# Taxonomic revision of the tarantula genus *Aphonopelma* Pocock, 1901 (Araneae, Mygalomorphae, Theraphosidae) within the United States

**DOI:** 10.3897/zookeys.560.6264

**Published:** 2016-02-04

**Authors:** Chris A. Hamilton, Brent E. Hendrixson, Jason E. Bond

**Affiliations:** 1 Department of Biological Sciences and Auburn University Museum of Natural History, Auburn University, Auburn, AL 36849, USA; 2 Department of Biology, Millsaps College, Jackson, MS 39210, USA

**Keywords:** Biodiversity, New species, Conservation, Molecular systematics, DNA taxonomy, DNA barcoding, Spider taxonomy

## Abstract

This systematic study documents the taxonomy, diversity, and distribution of the tarantula spider genus *Aphonopelma* Pocock, 1901 within the United States. By employing phylogenomic, morphological, and geospatial data, we evaluated all 55 nominal species in the United States to examine the evolutionary history of *Aphonopelma* and the group’s taxonomy by implementing an integrative approach to species delimitation. Based on our analyses, we now recognize only 29 distinct species in the United States. We propose 33 new synonymies (*Aphonopelma apacheum*, *Aphonopelma minchi*, *Aphonopelma rothi*, *Aphonopelma schmidti*, *Aphonopelma stahnkei* = *Aphonopelma chalcodes*; *Aphonopelma arnoldi* = *Aphonopelma armada*; *Aphonopelma behlei*, *Aphonopelma vogelae* = *Aphonopelma marxi*; *Aphonopelma breenei* = *Aphonopelma anax*; *Aphonopelma chambersi*, *Aphonopelma clarum*, *Aphonopelma cryptethum*, *Aphonopelma sandersoni*, *Aphonopelma sullivani* = *Aphonopelma eutylenum*; *Aphonopelma clarki*, *Aphonopelma coloradanum*, *Aphonopelma echinum*, *Aphonopelma gurleyi*, *Aphonopelma harlingenum*, *Aphonopelma odelli*, *Aphonopelma waconum*, *Aphonopelma wichitanum* = *Aphonopelma hentzi*; *Aphonopelma heterops* = *Aphonopelma moderatum*; *Aphonopelma jungi*, *Aphonopelma punzoi* = *Aphonopelma vorhiesi*; *Aphonopelma brunnius*, *Aphonopelma chamberlini*, *Aphonopelma iviei*, *Aphonopelma lithodomum*, *Aphonopelma smithi*, *Aphonopelma zionis* = *Aphonopelma iodius*; *Aphonopelma phanum*, *Aphonopelma reversum* = *Aphonopelma steindachneri*), 14 new species (*Aphonopelma atomicum* **sp. n.**, *Aphonopelma catalina* **sp. n.**, *Aphonopelma chiricahua* **sp. n.**, *Aphonopelma icenoglei* **sp. n.**, *Aphonopelma johnnycashi* **sp. n.**, *Aphonopelma madera* **sp. n.**, *Aphonopelma mareki* **sp. n.**, *Aphonopelma moellendorfi* **sp. n.**, *Aphonopelma parvum* **sp. n.**, *Aphonopelma peloncillo* **sp. n.**, *Aphonopelma prenticei* **sp. n.**, *Aphonopelma saguaro* **sp. n.**, *Aphonopelma superstitionense* **sp. n.**, and *Aphonopelma xwalxwal* **sp. n.**), and seven *nomina dubia* (*Aphonopelma baergi*, *Aphonopelma cratium*, *Aphonopelma hollyi*, *Aphonopelma mordax*, *Aphonopelma radinum*, *Aphonopelma rusticum*, *Aphonopelma texense*). Our proposed species tree based on Anchored Enrichment data delimits five major lineages: a monotypic group confined to California, a western group, an eastern group, a group primarily distributed in high-elevation areas, and a group that comprises several miniaturized species. Multiple species are distributed throughout two biodiversity hotspots in the United States (i.e., California Floristic Province and Madrean Pine-Oak Woodlands). Keys are provided for identification of both males and females. By conducting the most comprehensive sampling of a single theraphosid genus to date, this research significantly broadens the scope of prior molecular and morphological investigations, finally bringing a modern understanding of species delimitation in this dynamic and charismatic group of spiders.

## Introduction

The family Theraphosidae (tarantulas, baboon spiders, earth tigers) is the most diverse lineage (World Spider Catalog 2015) of spiders placed in the infraorder Mygalomorphae (Raven 1985, Hedin and Bond 2006, Bond et al. 2012, Bond et al. 2014). Residing within this group is the most species-rich lineage of tarantulas, the genus *Aphonopelma*, with 87 nominal species distributed throughout North and Central America, 55 of which are thought to occur in the United States (World Spider Catalog 2015). Despite their systematic appeal stemming from their diversity and charismatic nature as hairy, large-bodied spiders, the systematics and taxonomy of *Aphonopelma* have remained problematic. In the past 75 years, only four significant descriptive or revisionary works have examined the taxonomy and range of morphological character variation of *Aphonopelma* in the United States (Chamberlin and Ivie 1939, Chamberlin 1940, Smith 1995, Prentice 1997), none of which employed an explicit phylogenetic approach. The goal of this partial revision is to formally resolve the group’s species-level diversity by implementing an integrative approach to species delimitation that considers genomic, morphological, ecological, and geospatial data.

Morphology-based phylogenies of mygalomorph spiders have revealed widespread patterns of homoplasy among traditional taxonomic characters (Raven 1985, Goloboff 1993, Bond and Opell 2002, Hedin and Bond 2006, Bond and Hedin 2006, Hendrixson and Bond 2009, Bond et al. 2012). Furthermore, quantitative or meristic features often used to evaluate relationships among mygalomorphs have been found to be problematic (Bond and Beamer 2006, Hendrixson and Bond 2009, but see Goloboff et al. 2006). Generally, morphological approaches to species delimitation in groups with similar patterns of homoplasy or morphological conservatism have been shown to grossly oversimplify and underestimate diversity (Locke et al. 2010, Niemiller et al. 2011).

**Figure 1.**
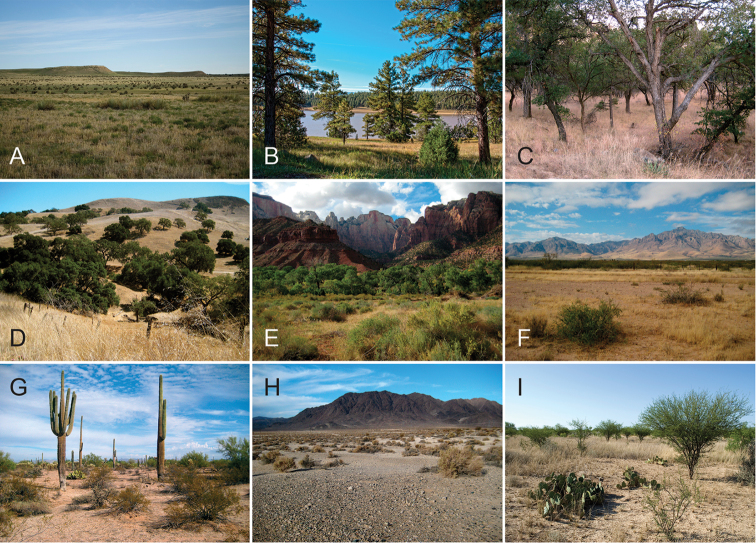
Breadth of diversity of *Aphonopelma* species habitat types across the United States. **A** grassland prairie, Otero Co., Colorado **B** high-elevation pine/conifer in Coconino Co., Arizona **C** mid-elevation oak woodland throughout the “sky islands” of southeastern Arizona (e.g. Madera Canyon in the Santa Rita Mountains) **D** grass/oak foothills of the Sierra Nevada Mountains, Mariposa Co., California **E** Zion National Park, Utah **F** Chihuahuan Desert below the Chiricahua Mountains in Cochise Co., Arizona **G** Sonoran Desert in Pinal Co., Arizona **H** Mojave Desert in San Bernardino Co., California **I** Tamaulipan thornscrub in Starr Co., Texas.

**Figure 2.**
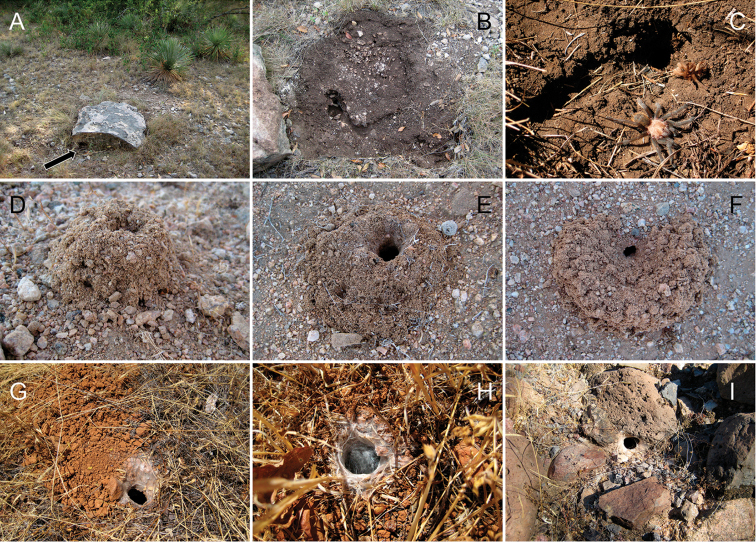
Representation of *Aphonopelma* burrows in different habitats across the United States. **A–C** a typical “scrape” (burrow under rock) of *Aphonopelma hentzi* in rocky habitat across their distribution **D–E** a turreted mound around the burrow of *Aphonopelma icenoglei* (also *Aphonopelma atomicum*, *Aphonopelma mojave*, and *Aphonopelma prenticei*) **F** the distinct crescent mound burrow of *Aphonopelma paloma* **G–I** typical free-standing burrows of *Aphonopelma chalcodes*, *Aphonopelma eutylenum*, *Aphonopelma iodius*, or *Aphonopelma johnnycashi* in desert, grassland, or rocky habitats.

Distributed across two major biogeographic realms (Nearctic and Neotropical), *Aphonopelma* species are distributed across the southern third of the United States, ranging west of the Mississippi River to California and south through Mexico and Central America (Fig. 1). The North American species of *Aphonopelma*, proposed to have rapidly diversified following expansion and adaptation into arid and desert environments ~5 Ma (Hamilton et al. 2011), can be found across a wide range of physical and climatic conditions. Most *Aphonopelma* live in silk-lined subterranean burrows and are found in nearly every habitat throughout their distribution (Fig. 2). Some species thrive in harsh environments, including hot and arid regions near Death Valley (California, USA), or in more temperate high-elevation forests along the Mogollon Rim and Madrean Archipelago (Arizona, USA) (Fig. 1). Perceived high diversity, complicated biogeography, morphological homogeneity between closely related taxa, and considerable variation within nominal species have posed significant problems for species delimitation (Prentice 1997) and higher-level classification (Raven 1985) in tarantulas. Not surprisingly, many arachnologists have expressed dismay toward the present state of theraphosid taxonomy (Raven 1985, Smith 1995, Pérez-Miles et al. 1996, Prentice 1997), with Raven (1990, p. 126) even declaring the group a “nomenclatural and taxonomic nightmare”.

The taxonomy of *Aphonopelma* is beset with poorly delimited species boundaries and very few specimens can be confidently identified using published keys (e.g., Chamberlin 1940, Smith 1995) or comparisons to original descriptions. With few exceptions (e.g., Prentice 1993, 1997, Warriner 2008), much of the descriptive work on *Aphonopelma* has been based upon only one or a few specimens, generally lacked consideration for the wide range of intraspecific and intrasexual variation, and was often subjective with respect to how characters were evaluated (Prentice 1997). Structure and variation of male and female genitalia has been a heavily weighted character in delimitation of spider species, but is of limited use in *Aphonopelma* due to morphological homogeneity across species and may only be useful for higher-level taxonomic groups (Prentice 1997). Male mating claspers – modifications on the first two pairs of legs in adult male mygalomorph spiders used in holding and stimulating females during copulation – have also been effective at delimiting species of mygalomorph spiders (e.g., Bond 2012), however, these are mostly homogeneous across *Aphonopelma* as well. Differences in somatic morphology have played an important role in delimiting species boundaries, but only a handful of described species have distinct color patterns or unique combinations of characters that readily facilitate identification. This has resulted in species boundaries delimited by highly variable morphological characters (e.g., leg article proportions, number and position of spines on appendages, eye patterns, etc.). But even when multiple samples of a given species are available, they commonly fall into two categories: (1) all specimens from the same general area; or (2) from the same sex (usually adult males because they are commonly collected after abandoning their burrows in search of females). The first issue poses problems for an obvious reason: we cannot assess geographic variation in characters that might be useful for diagnosing species. The second issue is problematic because male tarantulas undergo considerable changes upon reaching maturity. *Aphonopelma* often display sexual dimorphism in characters given heavy weight for delimiting species boundaries, making it especially difficult to associate male and female specimens of the same species, particularly in areas where two or more species occur in syntopy.

*Aphonopelma* has a complicated nomenclatural history. Pocock (1901) erected the genus during his dismemberment of *Eurypelma* to accommodate *Eurypelma seemanni* (type species) and other species from the United States, Mexico, and Central America. In the same paper, he described *Dugesiella* (type species *Dugesiella crinita*). *Aphonopelma* and *Dugesiella* were distinguished by the absence of a plumose scopula on the prolateral surface of the palpal trochanter, and presence of spiniform and thorn-like (basally-swollen) setae on prolateral coxa I. Petrunkevitch (1939) later described *Delopelma* (type species *Eurypelma marxi*) and differentiated it from both *Aphonopelma* (by the presence of simple, recumbent hairs on coxae and trochanters) and *Dugesiella* (by the complete absence of plumose hairs). Chamberlin (1940) demoted *Delopelma* to a subgenus of *Aphonopelma* when he recognized the presence of plumose setae in all genera and the similar form of setae on prolateral coxa I; described another subgenus (*Gosipelma*, type species *Gosipelma angusi*); and established a new genus *Chaunopelma* (type species *Delopelma radinum*) that differed from both *Aphonopelma* and *Dugesiella* by the presence of fine, soft prone hairs on the anterior coxa and trochanter of leg I and on the posterior palpal trochanter. The nomenclature and composition of these genera remained unchanged for 45 years until Raven (1985) conducted a large-scale revision of mygalomorph genera. Raven considered the differences between *Aphonopelma*, *Dugesiella*, and *Chaunopelma* insignificant and artificial, synonymizing all of them with *Rhechostica* Simon, 1892 (type species *Homoeomma texense*). However, because the name *Aphonopelma* had been cited more extensively than *Rhechostica*, the ICZN was petitioned to give *Aphonopelma* priority over *Rhechostica*. Through Opinion 1637, *Aphonopelma* was given precedence (ICZN 1991). Smith (1995) subsequently described the genus *Apachepelma* to accommodate *Aphonopelma paloma* Prentice, 1993, the first known miniature species in the genus. However, Prentice (1997) transferred the species back to *Aphonopelma* (thus synonymizing *Apachepelma* in the process). Presently, *Aphonopelma* is the only theraphosid genus native to the United States.

The discovery of species provides the crucial first step in the ongoing pursuit to understand the evolutionary patterns and processes shaping the biodiversity landscape. With over 250 years of taxonomic work behind us, ~1.2 million species have been described - with an estimated 7–10 million species on Earth still remaining to be described (Mora et al. 2011, Padial et al. 2010). Unfortunately, species are disappearing at an alarming rate (Barnosky et al. 2011, Vos et al. 2014), often without even being known to science, complicating our view of the Tree of Life. According to a new study, one-fifth of the world’s invertebrates are at risk for extinction, particularly species with limited vagility (Collen et al. 2012) – highlighting a concern for long-lived mygalomorph spiders. By conducting the most comprehensive sampling of a single theraphosid genus to date, this systematic revision aims to significantly broaden the scope of prior molecular (Graham et al. 2015, Hamilton et al. 2011, 2014, Hendrixson et al. 2013, 2015, Wilson et al. 2013) and morphological investigations (Prentice 1993, 1997, Smith 1995, Warriner 2008), finally bringing a modern understanding of the true species diversity in this dynamic and charismatic group of spiders, in particular the multiple species distributed throughout two biodiversity hotspots in the United States (i.e., California Floristic Province and Madrean Pine-Oak Woodlands) (Fig. 1B–D).

## Abbreviations

### Institutional

AMNH
American Museum of Natural History; New York, New York 


AUMNH
Auburn University Museum of Natural History; Auburn, Alabama 


BMNH
 The Natural History Museum, London; London, England 


MNHN
Muséum national d' Historie naturelle; Paris, France 


NHMW
Naturhistorisches Museum Wien; Vienna, Austria 


NMNH
 Smithsonian National Museum of Natural History; Washington D.C. 


### Quantitative morphological landmarks (Fig. 3)

Cl
 length of the carapace 


Cw
 width of the carapace 


LBl
 labial length 


LBw
 labial width 


F1
 femur I length (retrolateral aspect) 


F1w
 femur I width 


P1
 patella I length 


T1
 tibia I length 


M1
 metatarsus I length 


A1
 tarsus I length 


F3
 femur III length (prolateral aspect) 


F3w
 femur III width 


P3
 patella III length 


T3
 tibia III length 


M3
 metatarsus III length 


A3
 tarsus III length 


F4
 femur IV length (prolateral aspect) 


F4w
 femur IV width 


P4
 patella IV length 


T4
 tibia IV length 


M4
 metatarsus IV length 


A4
 tarsus IV length 


PTl
 palpal tibia length (retrolateral aspect) 


PTw
 palpal tibia width 


SC3
 ratio of the extent of metatarsus III scopulation (length of scopulation/ventral length of metatarsus III) 


SC4
 ratio of the extent of metatarsus IV scopulation (length of scopulation/ventral length of metatarsus IV) 


**Figure 3.**
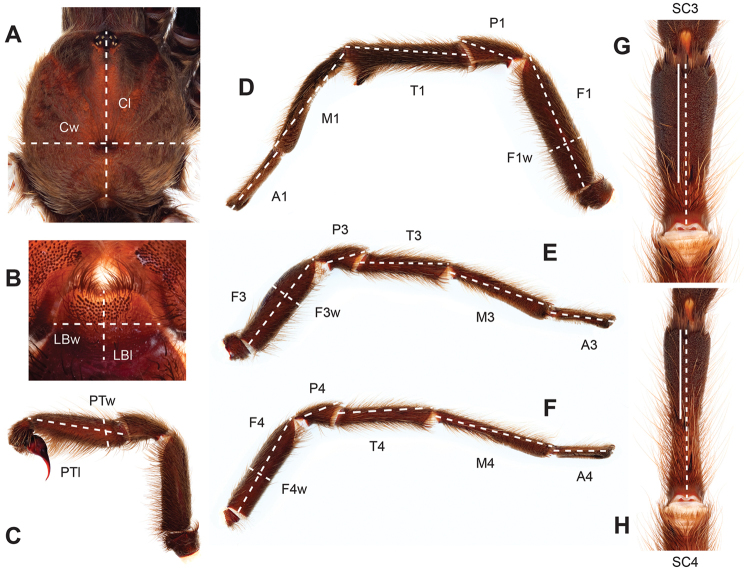
Diagrammatic representation of informative quantitative measurements. **A–B** carapace, labium length and width **C** retrolateral palpal tibia length and width **D** retrolateral lengths of leg 1 femur, patella, tibia, metatarsus, tarsus, and width of femur **E** prolateral lengths of leg 3 femur, patella, tibia, metatarsus, tarsus, and width of femur **F** prolateral lengths of leg 4 femur, patella, tibia, metatarsus, tarsus, and width of femur **G** ventral length of scopulation (line), ventral length of metatarsus III (dashed line) **H** ventral length of scopulation (line), ventral length of metatarsus IV (dashed line).

## Methods

### Measurement, characterization, and illustration of morphological features

All material was preserved in 80% ethanol and assigned a unique alphanumeric voucher number (APH_#### or AUMS_####) added to each vial and can be used to cross-reference all images, measurements, and locality data. Quantitative measurements are reported in millimeters and were made with a Leica M165C stereomicroscope using the Leica Application Suite software and a digital camera, or from a Mitutoyo ABSOLUTE Digimatic handheld digital caliper. Appendage measurements were based on left appendages (unless otherwise stated); palpal tibia & leg I - retrolateral, legs III & IV - prolateral, extent of metatarsal scopulation - ventral. Lengths of leg articles were taken from the mid-proximal point of articulation to the mid-distal point of the article (*sensu* Coyle 1995, Bond 2012, Bond and Godwin 2013, see Fig. 3). Individuals were selected for measurement from > 2,900 specimens collected during this project and museum specimens from the AMNH and the AUMNH (Suppl. material 1).

Quantitative measurements are based on a minimum of five individuals of each sex, when a sufficient number of specimens were available, that represent the geographic, molecular (based on the *CO1* data), and morphological breadth of each species across its distribution (i.e., every attempt was made to select specimens that represented the range of sizes available across the distribution). Material Examined sections were generated using the MATex Python script used in Bond (2012). Digital images of specimens were made using a Visionary Digital Imaging System (Visionary Digital^TM^, Richmond, VA) where images were recorded at multiple focal planes and then assembled into a single focused image using the computer program Zerene Stacker v.1.04 (Zerene Systems LLC, Richland, WA). The female genital region was removed from the abdominal wall and tissues dissolved using trypsin, incubated overnight at 37^o^C in a 1.5ml tube; spermathecae were examined and photographed in the manner described above. All images were cropped and toned using Adobe Photoshop (Adobe Systems, Inc.). All morphological measurement datasets and images, for all species, have been deposited in the Dryad Data Repository (doi: 10.5061/dryad.k6c82).

### Evaluation of quantitative morphological characters for species diagnoses

Morphometrics that were determined to have non-overlapping ranges were used as features for morphological diagnoses of species. The approach applied here is not without certain problems. For example, the inclusion of additional specimens, or in particular the case of species represented by only one or a few specimens (i.e., *Aphonopelma chiricahua* or *Aphonopelma saguaro* females), additional collecting could add specimens whose features expand the range of some characters and negate some of the measurements used to diagnose species. Building on the importance of certain morphological features found in Prentice (1997), we investigated 153 ratio combinations for males and 135 in females (Suppl. material 2).

**Figure 4.**
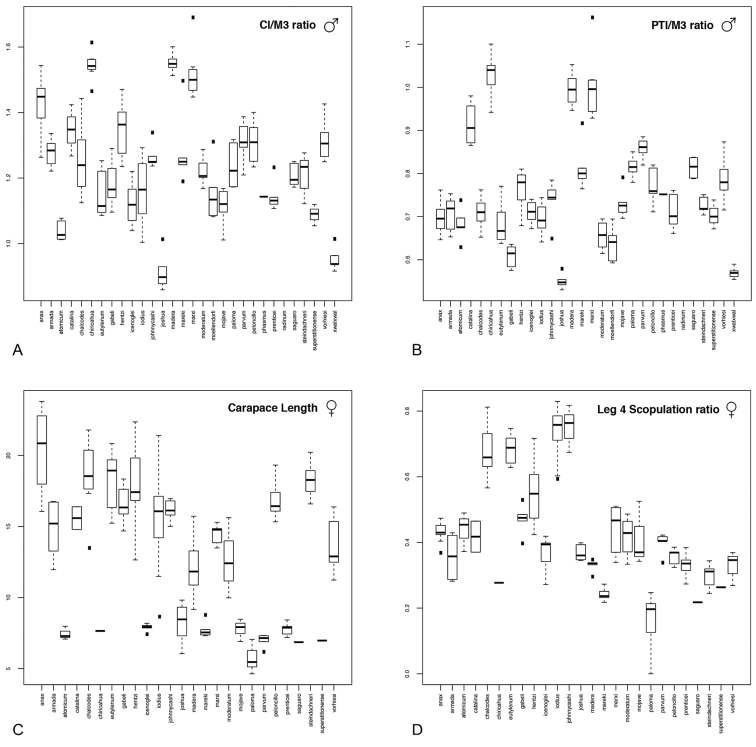
Examples of boxplots from male quantitative measurements used in species diagnosis (x-axis represents species). **A, B, D** are ratios: **A** carapace length/metatarsus III length **B** palpal tibia length/metatarsus III length **D** extent of scopulation on metatarsus IV **C** is carapace length (a proxy for body size), clearly showing the size differences between the miniature species and all other species (y-axis in mm). Additional boxplots can be viewed in Suppl. material 2.

**Figure 5.**
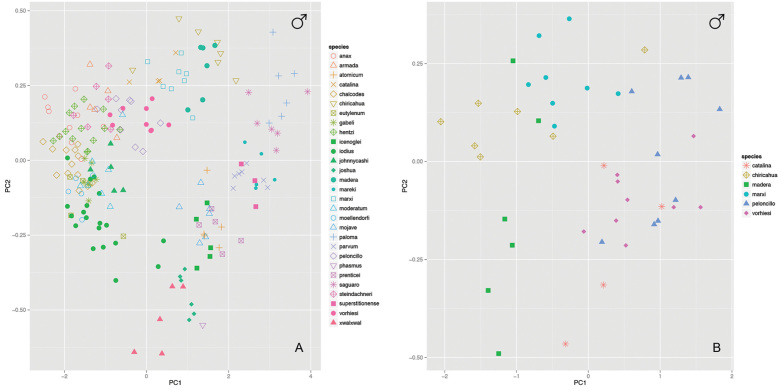
Examples of PCA morphospace plotted from quantitative measurements of males, used in species diagnosis. **A** a plot of males from all species in the United States **B** a plot of males in the *Marxi* species group.

To evaluate morphological variation, we examined the morphospace occupied by each species by plotting measurement ratios in boxplots (e.g., Fig. 4; Suppl. material 2), traditional PCA morphospace (e.g., Fig. 5; Suppl. material 2), and three-dimensional PCA morphospace (see GIF movies in Suppl. material 2). Simple R (R Core Team 2014) scripts, employing the packages “rgl”, “pca3d”, and “ggplot2”, have been created to evaluate new specimens either against our data or just the users data (Suppl. material 3). To evaluate PCA morphological space, all measurements (excluding ratios) were natural logarithmically transformed (i.e., ln(x)) to account for differences in body size. Traditional PCA morphospace was evaluated by plotting PC1~PC2, while three-dimensional PCA morphospace was evaluated by plotting PC1~PC2~PC3. All morphospace plots, associated scripts, and datasets have been deposited in the Dryad Data Repository (doi: 10.5061/dryad.k6c82).

### Taxon sampling, molecular techniques, and phylogenetic analyses

Through extensive fieldwork and a citizen-based science program (in association with the American Tarantula Society, see http://www.atshq.org/articles/found.html), we acquired nearly 1,800 specimens of *Aphonopelma* and closely related sister taxa, for DNA. Specimens were opportunistically collected throughout the southwestern United States, with every attempt made to gather topotypic material from (or near) the type localities of all nominal species of *Aphonopelma* in the United States. Of these, *Aphonopelma phasmus* Chamberlin, 1940 (type locality Phantom Ranch, Grand Canyon) was the only species for which we were unable to obtain fresh material. Legs were removed from all freshly collected material and preserved in ≥95% ethanol or RNA*later*^TM^ (Qiagen, Valencia, CA, USA) and stored at -80^o^C. Specimens are deposited at the AUMNH, with select duplicate specimens of novel species deposited at the AMNH.

Phylogenetic analyses of *Aphonopelma* relationships were conducted using molecular datasets (mitochondrial and nuclear) employing likelihood optimality criteria. A new mitochondrial dataset drawn from the taxa in Hamilton et al. (2014) and from newly collected data represent the most heavily DNA-barcoded spider genus to date (n = 1032 specimens). Genomic DNA was extracted from muscle tissues using the Qiagen DNeasy Tissue Kit^TM^ (Qiagen, Valencia, CA, USA). The concentration quality of the extracted DNA was quantified with a spectrophotometer (NanoDrop ND-1000, Thermo Scientific, Wilmington, DE, USA) or visualized via agarose gel electrophoresis. PCR and direct sequencing primers used for the cytochrome c oxidase subunit I (*CO1*) barcoding fragment are listed in Hamilton et al. (2011); PCR protocols follow Hendrixson et al. (2013). PCR products were purified using ExoSAP-IT (USB Corporation; Cleveland, OH, USA) and then sequenced with an ABI 3130 Genetic Analyzer (Applied Bio-systems, Foster City, CA, USA) using the ABI Big Dye Terminator version 3.2 Cycle Sequencing Ready Reaction Kit. All *CO1* sequences were manually edited using Sequencher (ver. 4.1.2, Genecodes, Madison, WI, USA). All *CO1* sequences were aligned with MUSCLE version 3.6 (Edgar 2004) using default parameters, followed by minor adjustment in MESQUITE version 3.03 (Maddison and Maddison 2015) if needed. Amino acid translations of the target gene region were examined to ensure the absence of stop codons in the alignment. The alignments were unambiguous and for consistency, sequences were trimmed to 900 basepairs. The program PartitionFinder v1.1.0 (Lanfear et al. 2014) was used to determine the appropriate model of DNA substitution. Each gene region was partitioned by codon position with separate models chosen for each partition. Maximum Likelihood (ML) analyses were carried out in RAxML-7.3.1 (Stamatakis 2006) on the CASIC HPC at Auburn University. Parameters for the analyses incorporated the GTRGAMMA model of evolution based on 10,000 random addition sequence replicates; branch support values were computed via 1000 non-parametric bootstrap replicates. The tree was rooted using divergent Central American species: *Sericopelma* sp. (APH_3003, 3004, 3020) from Panama; *Aphonopelma belindae* (APH_3001, 3008) from Panama; and Aphonopelma sp. xanthochromum (APH_3024) from Costa Rica. All previous *CO1* sequences were deposited in GenBank and can be found in Hamilton et al. (2011, 2014), Hendrixson et al. (2013, 2015), and Graham et al. (2015); all new *CO1* sequences have been deposited in GenBank (accession numbers: KU054417-KU055448). DNA sequence alignments and associated phylogenetic trees and data matrices, accompanying tree files, and scripts have been deposited in the Dryad Data Repository (doi: 10.5061/dryad.k6c82).

The analyses described above and recent smaller scale molecular studies (Hamilton et al. 2011, 2014, Hendrixson et al. 2013, 2015, Wilson et al. 2013, Graham et al. 2015) illustrate the potential that molecular data have for both separating and identifying known and unknown species within *Aphonopelma*. Unfortunately, all of these studies relied largely upon mitochondrial markers. The limitations of mtDNA have been documented extensively, namely, gene tree/species tree incongruence and the haploid, non-recombining nature of the molecule (Maddison 1997, Funk and Omland 2003, Knowles and Kubatko 2010). With mtDNA representing only one particular genealogy out of all possible within a genome, it is unlikely to fully resolve *Aphonopelma* phylogeny or demographic history accurately – though the use of mtDNA data represent an important first-step in untangling the complicated taxonomic problem that has developed for the tarantula fauna in the United States.

The use of nuclear loci generally has led to an enhanced understanding of the Tree of Life, but unfortunately, many non-model invertebrates (e.g., spiders) lack adequately developed loci for targeted sequencing. Until very recently, few genetic markers were available for inferring spider phylogenies (i.e., only 13 independent markers though 2013, see Gillespie et al. 1994, Hedin 1997, Agnarsson et al. 2007, Ayoub et al. 2007, Masta et al. 2009, Hamilton et al. 2011, Bond et al. 2012, Hendrixson et al. 2013, Satler et al. 2013). The poor resolution provided by traditional nuclear markers (e.g., 28S, 18S, H3) for inferring shallower relationships indicates many more loci need to be developed in order to overcome the stochasticity of sequence evolution and gene trees. Next-generation sequencing (NGS) has transformed molecular phylogenetics by enabling systematists to more confidently resolve major branches on the Tree of Life by gathering vast quantities of genomic data with relative ease. Given the difficulties in theraphosid systematics, novel genomic data is desperately needed to accurately delimit species and to reconstruct a robust phylogenetic framework for the group. To do this, a novel targeted sequencing approach was modified for use in spiders (Hamilton et al. *in prep*). Anchored Enrichment (AE) is a relatively new NGS methodology designed to recover hundreds of unique loci (i.e., single copy informative markers) from across the genome to resolve relationships at all taxonomic depths (Lemmon et al. 2012). Originally developed for vertebrates, Hamilton et al. (*in prep*) adapted this powerful approach to work in mygalomorph spiders by using six arachnid genomes and 17 transcriptomes to identify 585 loci. When possible, two specimens of each *CO1* species were sequenced, selecting specimens that encompassed the geographic and genetic breadth of each species. Unfortunately, at the time of our AE sequencing, some *CO1* species were singletons or we lacked good nuclear DNA for sequencing (i.e., old specimens whose tissue was not preserved for genomic sequencing). A total of 80 OTUs, including three outgroup taxa (*Aphonopelma belindae* (APH_3001), Aphonopelma sp. burica (APH_3012), and *Sericopelma* sp. (APH_3004)) were selected for sequencing and phylogenetic inference.

Anchored Enrichment data were collected through the Center for Anchored Phylogenomics at Florida State University (http://www.anchoredphylogeny.com) following the general methods of Lemmon et al. (2012), modified in Lemmon et al. (*in prep*), and applied to spiders in Hamilton et al. (*in prep*). Tissue preparation and DNA extraction are the same as above. Briefly, each genomic DNA sample was sonicated to a fragment size of ~300-800bp, library preparation and indexing were performed following a protocol modified from Meyer and Kircher (2010). Indexed samples were then pooled at equal quantities and enrichments were performed on each multi-sample pool using an Agilent Custom SureSelect Kit (Agilent Technologies; herein referred to as the Spider Probe Kit), which contained probes designed for anchored loci from multiple spider genomes and transcriptomes. After enrichment, each set of enrichment reactions were pooled in equal quantities for sequencing on PE150 Illumina HiSeq2000 lanes. Sequencing was performed in the Translational Science Laboratory in the College of Medicine at Florida State University.

Utilizing the bioinformatics pipeline described in Lemmon et al. (2012), modified in Lemmon et al. (*in prep*), and applied to spiders in Hamilton et al. (*in prep*), paired-end sequencing reads were filtered for quality. Reads were demultiplexed and pairs were identified and merged following Rokyta et al. (2012). Reads were assembled into contigs using an assembler (Lemmon et al. *in prep*) that makes use of both a divergent reference assembly approach to map reads to the probe regions and a de-novo assembly approach to extend the assembly into the flanks. Reads were mapped to the spider probes, consensus bases called, contamination filtered, consensus sequences were grouped by locus (across individuals) in order to produce sets of homologs, and orthology determined. From this, 455 loci (229,854bp) were selected for use in *Aphonopelma*. Sequences in each orthologous set were aligned using MAFFT v7.023b (Katoh 2013), with --genafpair and --maxiterate 1000 flags utilized. All alignments were visually investigated in Geneious Pro v5.6 (http://www.geneious.com, Kearse et al. 2012) for consistency. A concatenated supermatrix of all loci was constructed to infer relationships using Maximum Likelihood (ML) phylogenetic inference in RAxML v7.3.1 (Stamatakis 2006). Parameters for the concatenated RAxML analysis incorporated the GTRGAMMA model of evolution based on 1000 random addition sequence replicates, branch support values were computed via 1000 non-parametric bootstrap replicates, and partitions were set for each locus. *Aphonopelma belindae* (APH_3001) was designated as the outgroup. Prior analyses (Turner et al. *in review*), and our preliminary analyses have inferred *Sericopelma* to be more closely related to the *Aphonopelma* in the United States and Mexico, than to the Central American *Aphonopelma*. Phylogenies for each individual locus were inferred using RAxML under the same parameters as above, with subsequent species tree estimation performed in ASTRAL v4.7.6 (Mirarab et al. 2014). All investigations were carried out on the CASIC HPC at Auburn University and the Florida State University HPC.

### Locality data, georeferencing, generation of niche-based distribution models, and conservation status

**Figure 6.**
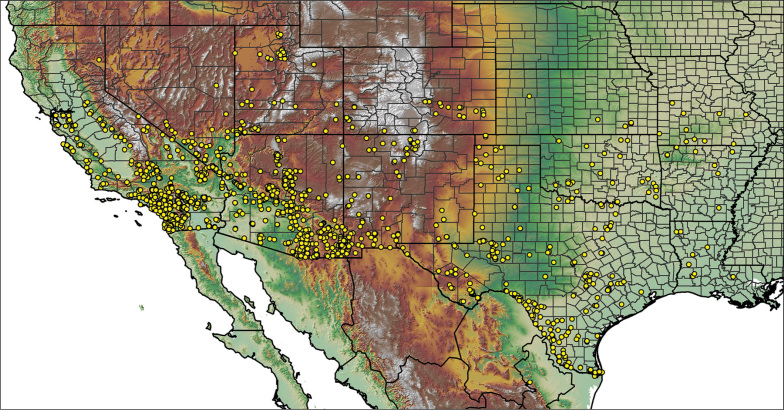
A generalized distribution map of all unique collecting localities from across the United States.

For all newly collected samples, latitude and longitude were recorded in the field using a Global Positioning System (GPS) receiver (WGS84 datum) in Decimal Degrees (DD). For previously collected museum specimens, locality data were manually georeferenced using Google Earth (Google, Mountain View, CA) or Topo North America (DeLorme, Yarmouth, ME) in Decimal Degrees (DD). All georeferenced and field recorded locality data (latitude, longitude, elevation) were crosschecked by hand in Google Earth, Topo North America, or ArcGIS (ESRI, Redlands, CA) prior to generating distribution maps, niche-based distribution models, and database entry. Distribution maps were constructed in ArcGIS. Because older locality labels often lack sufficient locality information, many georeferenced values are imprecise and should be interpreted with caution. As well, because these older labels are often faded or handwritten, there may be slight discrepancies in the spelling of localities, collectors, etc. Data for labels that document only county and/or town information were georeferenced to the approximate geographic center of the given locality. Precision for each georeferenced point is annotated as a superscript in the material examined section for each species using the confidence value scheme employed by Murphey et al. (2004) and Bond (2012) and modified herein: 1 = exact coordinates given; 2 = exact location given, validated in Google Earth; 3 = public land survey (or herein geographic place name); 4 = within 1 km radius (~.5 mile); 5 = within 5 km radius (~3 miles); 6 = within 10 km radius (~6 miles); 7 = to county or > 10 km; 8 = to state; 9 = to project region. Detailed locality and associated GIS information can be found in Suppl. material 1. See Figure 6 for a generalized map of all collecting sites of all species across the United States.

As an approach to facilitate species discovery and delimitation, niche-based distribution models (DMs) were constructed for species for which sufficient locality data were available (i.e., at least 10 different localities separated by at least 1 km). Niche-based DMs provide estimates for the probability of finding a species at a location on the landscape given the set of correlate ecological and climatic parameters used to construct the model. Locality coordinates for each specimen were imported into ArcMap (ESRI, Redlands, CA) and converted into shapefiles. Following the procedures outlined in Bond and Stockman (2008) and as previously implemented in *Aphonopelma* (Hendrixson et al. 2013, Graham et al. 2015), DMs were constructed using environmental layers thought to “likely influence the suitability of the environment for the species” (Phillips et al. 2006, p. 232). We selected eight environmental layers from the WORLDCLIM data set (Hijmans et al. 2005) based largely on the arguments presented by Bond and Stockman (2008): ALT (elevation), BIO4 (Temperature Seasonality), BIO5 (Max Temperature of Warmest Month), BIO6 (Min Temperature of Coldest Month), BIO12 (Annual Precipitation), BIO15 (Precipitation Seasonality), BIO16 (Precipitation of Wettest Quarter), and BIO17 (Precipitation of Driest Quarter). All layers were clipped to the same extent, cell size, and projection. DMs were created in the program Maxent ver. 3.3.3k (Phillips et al. 2006) using default parameters.

A hypothesized conservation status for each species has been included with each respective description. The designations provided herein are not based on any formal calculations and therefore should not be viewed as formal status declarations. These designations are based upon our own extensive fieldwork and the observations of fellow arachnologists (e.g., Tom Prentice and Wendell Icenogle). As such, our estimation of the conservation status for each species is likely conservative.

### Species delimitation and conceptualization

The species concept used throughout this taxonomic revision utilizes the ideas of de Queiroz’s Unified Species Concept (2005). Where possible, we employ a combination of molecular phylogenetic, morphological, behavioral, and biogeographic evidence to identify independently evolving lineages. If not possible, an alternative species concept is noted. Initial species hypotheses were based on the recognized morphological species hypotheses (World Spider Catalog 2015). To test these initial species hypotheses, we evaluated the agreement of mtDNA (*CO1*) with the morphological species hypotheses or putative new lineages, following the methodology laid out in Hamilton et al. (2014). Morphological species boundaries were then reevaluated based on *CO1* species boundaries. We then evaluated whether there was agreement of biogeographical and behavioral data with these species hypotheses. Finally, we employed the nuclear loci developed using Anchored Enrichment (Hamilton et al. *in prep*) to evaluate those species hypotheses and establish whether independent lineages have sorted. Following this final delimitation of species, a reciprocal illumination approach (see Lienau et al. 2006 and Padial et al. 2010) was employed to find informative morphological characters that can be used (absent of molecular data) across the group. This implementation of a tree-based approach attempts to identify specimens to species by phylogenetic association, providing evidence of common ancestry with specimens identified by other means (e.g., morphological or cohesion species criteria).

## Data resources

All data (molecular, morphological, geographic, and images) used to establish these species hypotheses have been deposited in the Dryad Data Repository (http://dx.doi.org/10.5061/dryad.k6c82). All locality data underpinning the analysis reported in this paper are deposited at GBIF, the Global Biodiversity Information Facility, http://ipt.pensoft.net/resource?r=aphonopelma. All morphological images have been deposited in Morphbank, and can be viewed by referencing the "APH-S" Specimen External Identifier. Additional specimen data, species plates, and morphological data can be found in the Suppl. materials.

## Results and discussion

### Summary of taxonomic diversity

Prior to our work leading up to this taxonomic revision (Hamilton et al. 2011, 2014, Hendrixson et al. 2013, 2015, Graham et al. 2015), only one other study had implemented a phylogenetic approach to understanding species diversity and evolutionary relationships in *Aphonopelma* (Wilson et al. 2013). A well-supported phylogeny is fundamental to addressing important questions regarding the role that biogeography, allopatry, ecological divergence, and ancestral interactions have played in the diversification of these lineages. Our results demonstrate that several species in the United States need to be synonymized (see Hamilton et al. 2011, 2014), novel species need to be named and described (Hamilton et al. 2011, 2014, Hendrixson et al. 2013, 2015, Graham et al. 2015), and a handful of species must be considered *nomina dubia*. Of the 55 nominal species of *Aphonopelma* reported from the United States prior to the present contribution, we only consider 15 of them valid (i.e., we propose 33 new synonymies and consider 7 species *nomina dubia*). Despite this significant reduction in the number of valid species, we also recognize 14 novel species (described herein), bringing the total diversity of *Aphonopelma* in the United States to 29 species. It is important to point out that geographic distribution is crucial to understanding *Aphonopelma* diversity within the United States.

### *Aphonopelma* phylogeny

**Figure 7.**
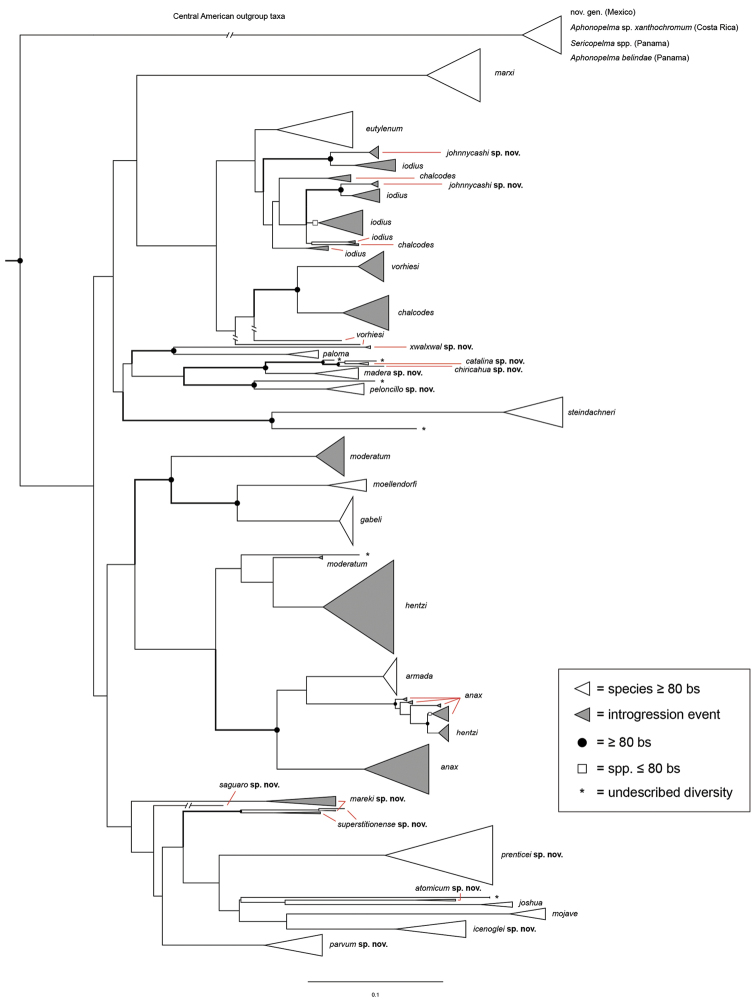
Maximum Likelihood inferred *CO1* gene tree phylogeny for the 1,032 *Aphonopelma* specimen dataset. Species delimitations followed the integrative methodological approach outlined in Hamilton et al. (2014). Black circles denote bootstrap support ≥ 80%; white squares denote bootstrap support ≤ 80%. White triangles indicate species clades supported with ≥ 80% bootstrap support; grey triangles indicate lineages with putative mitochondrial introgression events. Asterisks at the tips of branches indicate undescribed diversity.

Following the integrative approach for delimiting species within *Aphonopelma* outlined in Hamilton et al. (2014), we increased *CO1* taxon coverage (n = 1032) to further investigate mtDNA species boundaries in the group by including more OTUs per species and by adding new putative species to the analysis. With the exception of the newly added species, relationships are generally consistent with our previous analyses (see Hamilton et al. 2014) (e.g., the same cryptic species lineages are delimited and the same hypothesized species are highly supported with deeper support throughout the tree decreasing as more OTUs were added; Fig. 7). The mitochondrial data suggest that we should recognize 41 species in the United States (not including *Aphonopelma phasmus* because we did not have tissue for collecting *CO1* data), with 17 nominal species and 24 previously undescribed species.

**Figure 8.**
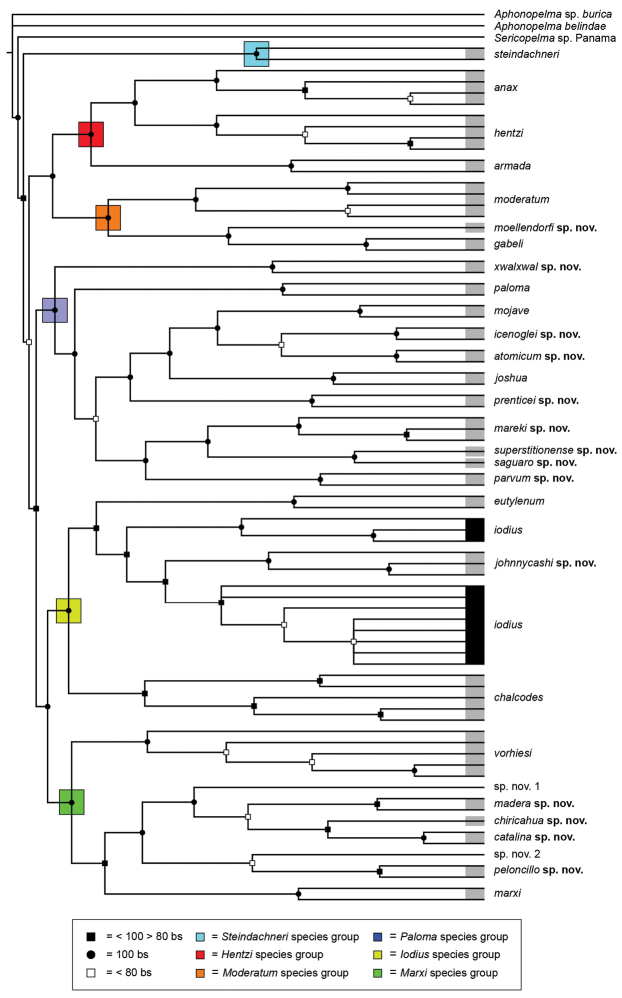
Species tree of all United States *Aphonopelma*, inferred from the 455 loci Anchored Enrichment dataset. Species delimitations correspond to our final integrative approach outlined herein. Black circles denote 100% bootstrap support; black squares denote bootstrap support between 99–80%; white squares denote bootstrap support less than 80%. Node support values = based on the RAxML bootstrap support from all trees and all loci. All genealogically exclusive species are identified with a grey bar; *Aphonopelma iodius*, a paraphyletic species as presently defined, is identified by the black boxes. All major species groups are identified by colored boxes.

To further assess species-level diversity and evaluate the associated hypothesized species boundaries delimited by the *CO1* data, we sequenced multiple specimens per putative species using Anchored Enrichment. A dataset of 455 loci (229,854 basepairs) across 80 OTUs produced a highly resolved species-level phylogeny of all the major species groups and regional clades within *Aphonopelma* (Fig. 8). These AE data identify 14 nominal species, 14 undescribed species, and 2 lineages that we will investigate in more depth in the future (labeled as sp. n. 1 and sp. n. 2 in Fig. 8; again, this analysis does not include *Aphonopelma phasmus*). All species are strongly supported as representing genealogically exclusive, independently evolving lineages, with one exception, *Aphonopelma iodius* (details below). Figure 8 summarizes the maximum likelihood inference of the AE molecular data and subsequent species tree inference, highlighting the monophyly and high support of the five major clades (see Fig. 8) of *Aphonopelma* within the United States: (1) a monotypic group that is confined to California; (2) a western group; (3) an eastern group; (4) a group primarily distributed in the high-elevation sky islands areas of Arizona and New Mexico; and (5) a group that comprises a diverse group of miniaturized species. The area that divides the western and eastern groups roughly corresponds with the Cochise Filter Barrier biogeographic region (see Pyron and Burbrink 2010) of southeastern Arizona and southwestern New Mexico, where the Chihuahuan and Sonoran deserts converge. The monotypic lineage includes the species *Aphonopelma steindachneri* (herein referred to as the *Steindachneri* species group); the western lineage includes the *iodius* species group (*Aphonopelma chalcodes*, *Aphonopelma eutylenum*, *Aphonopelma iodius*, and *Aphonopelma johnnycashi* sp. n.); the eastern lineage includes the *Hentzi* species group (*Aphonopelma anax*, *Aphonopelma armada*, and *Aphonopelma hentzi*) and *moderatum* species group (*Aphonopelma gabeli*, *Aphonopelma moderatum*, and *Aphonopelma moellendorfi* sp. n.). By far, the greatest amount of novel diversity is found in the two remaining clades: the *Marxi* species group, a lineage that includes predominately black species (both adult males and females) and often found in montane or other high-elevation habitats, in particular the sky islands region of southwestern New Mexico and southeastern Arizona (*Aphonopelma catalina* sp. n., *Aphonopelma chiricahua* sp. n., *Aphonopelma madera* sp. n., *Aphonopelma marxi*, *Aphonopelma peloncillo* sp. n., and *Aphonopelma vorhiesi*); and the *paloma* species group, a collection of closely-related species that have evolved a small size relative to most other species in the United States (*Aphonopelma atomicum* sp. n., *Aphonopelma icenoglei* sp. n., *Aphonopelma joshua*, *Aphonopelma mareki* sp. n., *Aphonopelma mojave*, *Aphonopelma paloma*, *Aphonopelma parvum* sp. n., *Aphonopelma phasmus*, *Aphonopelma prenticei* sp. n., *Aphonopelma saguaro* sp. n., *Aphonopelma superstitionense* sp. n., and *Aphonopelma xwalxwal* sp. n.). Each species is genealogically exclusive and strongly supported (≥80 bootstrap support) (Fig. 8).

While our methodological approach using *CO1* identified the broad effectiveness of this 900 basepair fragment of mtDNA to identify known species and help illuminate species boundaries across the most comprehensive molecular sampling of a single spider genus to date, the AE nuDNA data robustly highlight where deep mitochondrial divergences and introgression have obscured our understanding of the true evolutionary history of these lineages. It is important to point out that a number of the putative mitochondrial species corresponded to lineages that were considered cryptic species in Hamilton et al. (2014), yet when viewed in the light of the AE data, are no longer distinct. This is perhaps not very surprising given the large number of papers showing how the use of a single locus, like mtDNA, to delimit species can be very misleading (e.g., Rubinoff et al. 2006, Petit and Excoffier 2009).

### *Steindachneri* species group

The *Steindachneri* species group presently includes a single species (*Aphonopelma steindachneri*) from California. The *CO1* and AE datasets both support the “basal” position of *Aphonopelma steindachneri* as the sister taxon to all other species of *Aphonopelma* in the United States (Figs 7, 8). There are likely more species in northwestern Mexico that will be placed into this group, in particular the Baja Peninsula.

### Western species group diversity (*iodius* species group)

The western group of species (note: the western designation is only applied here in an informal sense because other lineages are distributed west of the Cochise Filter Barrier) is comprised entirely of the *iodius* species group (*Aphonopelma chalcodes*, *Aphonopelma eutylenum*, *Aphonopelma iodius*, and *Aphonopelma johnnycashi* sp. n.) (Fig. 8). The AE dataset identifies *Aphonopelma chalcodes* as the sister taxon to the remaining members of the *iodius* species group, with *Aphonopelma eutylenum* the sister lineage to the *Aphonopelma iodius* species complex. The *CO1* and AE datasets recognize *Aphonopelma eutylenum* as a distinct and genealogically exclusive species. The recognition of *Aphonopelma johnnycashi* sp. n. renders *Aphonopelma iodius* paraphyletic (see below).

Unfortunately, the mitochondrial data are complex. Results from the *CO1* analysis confounds our understanding of species boundaries and relationships in *Aphonopelma*, likely due to mitochondrial introgression or deep haplotype conservation (e.g., one mtDNA lineage of *Aphonopelma chalcodes* is sister to *Aphonopelma vorhiesi*, a species that belongs to the *Marxi* species group, while another mtDNA lineage of *Aphonopelma chalcodes* is sister to other *Aphonopelma iodius* lineages). Alternatively, the AE nuclear data provide support for these species (*Aphonopelma chalcodes*, *Aphonopelma vorhiesi*) as independently evolving and monophyletic lineages. In previous analyses (Hamilton et al. 2014), we would have considered the two mtDNA lineages of *Aphonopelma chalcodes* as separate cryptic species. We also see that *Aphonopelma iodius*, as presently defined, is a paraphyletic species with lineages in Utah comprising a highly supported, genealogically exclusive lineage that is sister to the new species *Aphonopelma johnnycashi* (from the western foothills of the Sierra Nevada). The Utah lineage is separate from other *Aphonopelma iodius* lineages that are currently lumped into one species (including the species *Aphonopelma iviei* and *Aphonopelma brunnius* which have been synonymized with *Aphonopelma iodius*). Unfortunately, at this time we do not have any additional evidence that can be used to separate the Utah lineage from these other lineages. Future work will need to focus on sequencing more individuals within this group, evaluating gene flow between populations, and looking more closely at the biogeographic history of these lineages to determine if/where the geographic and genetic split occurs.

### Eastern species group diversity (*Hentzi* and *moderatum* species groups)

The eastern group of species is designated as such because the six species are largely distributed east of the Cochise Filter Barrier. This monophyletic lineage is strongly supported and includes the *Hentzi* species group (*Aphonopelma anax*, *Aphonopelma armada*, and *Aphonopelma hentzi*) and *moderatum* species group (*Aphonopelma gabeli*, *Aphonopelma moderatum*, and *Aphonopelma moellendorfi* sp. n.) (Fig. 8). The three species in the *Hentzi* species group are phenotypically similar and are the only species in the United States that possess stout setae on the prolateral surface of coxa I; freshly molted specimens typically possess black legs, an abdomen with short black setae and longer reddish setae, and a copper, brown, or tan carapace. Within the highly supported, monophyletic *Hentzi* species group *Aphonopelma armada* is inferred as the sister lineage to *Aphonopelma hentzi* and *Aphonopelma anax*. The *moderatum* species group is composed of three species whose adult males undergo significant color changes upon reaching sexual maturity (i.e., usually becoming solid black and fading over the course of their respective breeding periods). Within the highly supported, monophyletic *moderatum* species group, *Aphonopelma moderatum* is the sister lineage to *Aphonopelma gabeli* and *Aphonopelma moellendorfi*.

When reviewing both the putative mitochondrial and cryptic species identified in Hamilton et al. (2014), we can see how the mtDNA analyses confound our understanding of species boundaries and relationships in *Aphonopelma* (e.g., several species delimited by the mtDNA data are no longer recognized by the AE data) (see Fig. 7 and Hamilton et al. 2014).

### Sky islands diversity (*Marxi* species group)

The *Marxi* species group includes *Aphonopelma catalina* sp. n., *Aphonopelma chiricahua* sp. n., *Aphonopelma madera* sp. n., *Aphonopelma marxi*, *Aphonopelma peloncillo* sp. n., and *Aphonopelma vorhiesi* (Fig. 8). Freshly molted specimens of these six species are generally black (adult males and females) and can often be found in the montane or other high-elevation habitats throughout the Madrean sky islands of southwestern New Mexico and southeastern Arizona; exceptions include *Aphonopelma vorhiesi* and some populations of *Aphonopelma peloncillo* which can be located in lower-elevation grassland or desert habitats, and *Aphonopelma marxi* which is found in the montane habitats of northern Arizona and New Mexico, as well as southwestern Colorado and southeastern Utah. Phylogenetic relationships are generally similar between the *CO1* and AE datasets, although the placement of the *Aphonopelma marxi* and *Aphonopelma vorhiesi* lineages within the *CO1* phylogeny has always been problematic (e.g., low support, suspect sister relationships). Outside of *Aphonopelma vorhiesi*, where both mitochondrial introgression and deep mitochondrial divergence obscure an understanding of species boundaries and relationships, there are no putative mitochondrial or cryptic species identified in Hamilton et al. (2014) that were invalidated by the nuclear and morphological data.

Despite our extensive collecting throughout this region, there likely still remains undescribed diversity in this species group (Figs 7–8). Our lack of specimens (particularly for the Madrean sky island species) is due to the difficulty of locating these spiders (see Hendrixson et al. 2015). The terrain throughout the sky islands makes it challenging to do field work because many of the mountain ranges are incredibly remote or otherwise difficult to access (due to rough roads or private land); this is confounded by the cryptic nature of these species (i.e., burrows are incredibly difficult to find throughout the rocky and forested habitats in these regions). A thorough understanding of the diversity across this unique biodiversity hotspot is going to require future funding and collaborative collecting efforts. Several other large mountain ranges in the region (e.g., the Dragoon, Galiuro, and Pinaleño Mountains) possess appropriate habitat but have not been rigorously searched for these tarantulas.

### Miniaturization and diversification in *Aphonopelma* (*paloma* species group)

The *paloma* species group includes a dozen miniaturized species primarily located in the Mojave and Sonoran deserts: *Aphonopelma atomicum* sp. n., *Aphonopelma icenoglei* sp. n., *Aphonopelma joshua*, *Aphonopelma mareki* sp. n., *Aphonopelma mojave*, *Aphonopelma paloma*, *Aphonopelma parvum* sp. n., *Aphonopelma phasmus*, *Aphonopelma prenticei* sp. n., *Aphonopelma saguaro* sp. n., *Aphonopelma superstitionense* sp. n., and *Aphonopelma xwalxwal* sp. n. (Fig. 8). The lineages within this group have a particularly interesting natural history. Wherever these species are located, they reside in syntopy with a larger species (generally from the *iodius* species group), yet are rarely found in syntopy with other members of the *paloma* species group. The larger species typically possess distributions that extend beyond that of individual members of the *paloma* species group; furthermore, these larger species can often be found in syntopy with multiple members of the *paloma* species group at different places across their distribution.

The *CO1* data can be used to confidently identify the species in this group (probably due to the relatively high mtDNA divergence between species), except *Aphonopelma mareki* and *Aphonopelma superstitionense* due to putative mitochondrial introgression. Unfortunately, interspecific relationships are not well supported and the placement of *Aphonopelma paloma* and *Aphonopelma xwalxwal* is problematic (i.e., not consistently recovered in the same place in the phylogeny). When the AE data are employed, the *paloma* species group is strongly supported and the placement of *Aphonopelma paloma* and *Aphonopelma xwalxwal* is confidently resolved. Most relationships within and between the remaining species are also strongly supported and highly resolved (Fig. 8). After reviewing the putative species identified in Hamilton et al. (2014), we see there are no mitochondrial or cryptic species that were invalidated by the nuclear and morphological data.

Body size is one of the most important determinants of an organism’s ecological role (Hanken and Wake 1993, and citations within). While the literature is replete with cases of miniaturization in sympatry due to character displacement (e.g., Bond and Sierwald 2002, Hendrixson and Bond 2005), these examples seemingly represent single events and do not appear to have led to subsequent lineage diversification within the smaller taxa. In *Aphonopelma*, a single origin of miniaturization is strongly supported and this event undoubtedly played a role in the diversification of these spiders in the United States (we recognize 13 species in this group, eight of which are new). We hypothesize that this miniaturization event (and associated changes in niche utilization), evolved as a consequence of ancestral character displacement. Following this shift in body size (a pre-zygotic isolating mechanism), members of the *paloma* species group may have been more able to capitalize on available microhabitats where the larger species were not as abundant (Prentice 1997, pers. obs.), allowing persistence in syntopy with larger taxa and leading to subsequent lineage diversification of these smaller taxa. In addition, these miniaturized species have limited dispersal capabilities and are probably more likely to undergo allopatric divergence than their larger counterparts (see Graham et al. 2015). Future research aims to investigate the processes that led to miniaturization and increased rates of diversification within the *paloma* species group.

### Species considered *nomina dubia*

Chamberlin (1940) described the species *Aphonopelma baergi* from Fayetteville, Arkansas. As noted by Smith (1995) and confirmed by our own examination of the holotype (AMNH), this species was likely mislabeled by the original collector. This large and robust tarantula is noticeably different from other species in the United States based on the morphology of its spermathecae (refer to Smith 1995, fig. 141); in addition, surveys of tarantulas in the region (i.e., Warriner 2008 and our own fieldwork) have failed to locate specimens that share diagnostic features with the holotype (all other tarantulas from Arkansas and surrounding states are referable to *Aphonopelma hentzi* (Girard, 1852)). Smith (1995) suggested that the species is most closely allied with members of the Mexican and Central American genus *Brachypelma* Simon, 1891 but did not propose any formal taxonomic changes (see Peters 2003, 2005, Rudloff 2008). Based on the holotype’s questionable origin, however, we do not recommend transferring this species to *Brachypelma* but instead consider the name *Aphonopelma baergi* a *nomen dubium*.

The species *Aphonopelma cratium* Chamberlin, 1940 is based on a male holotype and female allotype (both AMNH – examined) from an uncertain location in California (“California ?” in the locality information on the label). These specimens are morphologically similar to other valid species in the state (e.g., *Aphonopelma iodius* (Chamberlin & Ivie, 1939) and *Aphonopelma eutylenum* Chamberlin, 1940), but because molecular data and/or accurate locality information are needed to distinguish these species, we consider *Aphonopelma cratium* a *nomen dubium*.

Smith (1995) described *Aphonopelma hollyi* on the basis of a single adult male specimen from Lubbock, Texas. Efforts to locate the holotype in the Oklahoma State University collection were unsuccessful and the specimen is presumed lost (Richard Grantham 2013, pers. comm.). We were unable to locate new topotypic material from Lubbock for purposes of designating a neotype but our extensive fieldwork in surrounding parts of the Llano Estacado and Caprock Escarpment strongly suggests that only two species with thorn-like setae on the prolateral surface of coxa I can be found there: *Aphonopelma hentzi* and *Aphonopelma armada* (Chamberlin 1940). Our initial thinking on the identity of *Aphonopelma hollyi* was that it should be considered a junior synonym of *Aphonopelma hentzi*. However, the description of *Aphonopelma hollyi* is vague, preventing one from distinguishing it from either *Aphonopelma hentzi* or *Aphonopelma armada*. Because the holotype has been lost and its identity is uncertain, we consider *Aphonopelma hollyi* a *nomen dubium*.

The description of *Eurypelma mordax* Ausserer, 1871 was based on a single adult male but the holotype was likely destroyed during World War II (see Smith 1995). After several decades, Roewer (1942) eventually synonymized *Eurypelma mordax* with *Aphonopelma hentzi* (then *Dugesiella hentzi*), but as noted by Smith (1995), the synonymy was questionable because Roewer never examined the type specimen. Based upon his own examination of material in the Koch collection at the BMNH, Smith (1995) found a female specimen from Texas that was labeled *Eurypelma mordax* by Ausserer and removed the species from synonymy of *Aphonopelma hentzi*. We, however, consider the identity of *Aphonopelma mordax* suspect for the following reasons: (1) the collection site for the destroyed holotype male is unclear; (2) Smith (1995) did not formally designate the female specimen as a neotype; (3) the female’s collection site in Texas is also unclear; and (4) because we do not have precise locality information for either specimen, there is no compelling reason to conclude that they belong to the same species. In the absence of additional useful information, we consider *Eurypelma mordax* = *Aphonopelma mordax* a *nomen dubium*.

Two different type localities (Ft. Yuma and Williams, Arizona) were listed in the description of *Eurypelma rusticum* Simon, 1891 but it is unclear which specimen was actually used for the description (Prentice 1997). In subsequent attempts to determine the precise identity of this species, both Smith (1995) and Prentice (1997) failed to locate the specimen from Ft. Yuma. In the original description, Simon (1891) also mentioned that the species was common in northern Mexico and this prompted Smith (1995) to designate a lectotype from Simon-determined paratype material in the MNHN; curiously, Smith’s choice of a lectotype (MNHN No. 5873 – not examined) from Mazatlán, Mexico is located 1300 kilometers southeast of Ft. Yuma in strikingly different habitat (see Prentice 1997). Three years later, Prentice (1997) examined what he thought might be Simon’s original specimen from Williams in the NMNH (USNM No. 1585 – not examined). He noted that the badly fragmented specimen was not conspecific with Smith’s lectotype, but did not make any changes; instead, he stated “reexamination of all type material will be necessary before a lectotype can be objectively designated” (Prentice 1997, pg. 146). Based on this confusion, we consider *Eurypelma rusticum* = *Aphonopelma rusticum* a *nomen dubium* because: (1) Simon’s description does little to help diagnose the species; (2) the type specimen from Ft. Yuma is lost; (3) the type specimen from Williams is in poor condition; (4) the lectotype from Mazatlán is not conspecific with Simon’s specimen from Williams; and (5) we have serious doubts that the lectotype from Mazatlán is conspecific with material from Yuma, Arizona and surrounding areas. As also discussed by Smith (1995) and Prentice (1997), specimens of this species cited in Chamberlin (1940) from Apache Trail, Arizona were described as *Aphonopelma rothi* Smith, 1994 (= *Aphonopelma chalcodes* Chamberlin, 1940, see below).

The enigmatic species *Delopelma radinum* (Chamberlin & Ivie, 1939) was described on the basis of a single adult male collected from Manhattan Beach, California in November 1937. To our knowledge, no specimens comparable to the badly fragmented holotype (AMNH – examined; see supplemental morphology images in Suppl. material 4) have been collected or reported from the Los Angeles Basin since the original description. While it is possible that the species has gone extinct (see Bond et al. 2006 and Bond 2012 for further discussion of mygalomorph spider extinctions in California), we strongly suspect that the type locality is incorrect because the habitat is atypical for North American tarantulas (see Prentice 1997). Morphological data reported in Prentice (1997, pg. 142) and the November collection date of the holotype, suggest that the species is probably *Aphonopelma mojave* Prentice, 1997 or *Aphonopelma icenoglei* sp. n. from the western Mojave Desert. However, we consider *Delopelma radinum* = *Chaunopelma radinum* = *Aphonopelma radinum* a *nomen dubium* for the following reasons: (1) despite extensive fieldwork throughout the area, we have failed to locate any specimens; (2) the type locality is highly dubious; (3) without molecular data, we cannot definitively associate the species with *Aphonopelma mojave* or *Aphonopelma icenoglei*.

Simon (1891) also described *Homoeomma texense* on the basis of a single adult male from “Texas: Rio-Grande (Geo. Marx)”. Similar to the situation with *Aphonopelma baergi* discussed above, this species is markedly different from other species in the United States based on the morphology of its palpal bulb (Smith 1995, fig. 799, but see Prentice 1997, Smith 2010); in addition, the type locality is vague and may include any portion of the river from its origin in Texas near El Paso to its mouth in the Gulf of Mexico (a distance of approximately 2000 kilometers that passes through numerous physiographic provinces). No other specimens with this unique palpal bulb morphology have ever been found in Texas; furthermore, Smith (2010) suspects that the holotype was mislabeled (the collector, George Marx, was notorious for mislabeling specimens) and may belong to the South American genus *Paraphysa* Simon, 1892. For these reasons, we consider *Homoeomma texense* = *Rhechostica texensis* = *Rhechostica texense* = *Aphonopelma texensis* = *Aphonopelma texense* a *nomen dubium*.

## Taxonomy

### Family Theraphosidae Thorell, 1869 Subfamily Theraphosinae Thorell, 1870

#### Genus Aphonopelma

Taxon classificationAnimaliaAraneaeTheraphosidae

Pocock, 1901

Rhechostica Simon, 1892: 162 (type species by original designation *Homoeomma texense* Simon, 1891). Suppressed as a senior synonym of *Aphonopelma* by ICZN Opinion 1637.
Dugesiella Pocock, 1901: 551 (type species by original designation *Dugesiella crinita* Pocock, 1901). First synonymized with *Rhechostica* by Raven (1985: 152).
Aphonopelma Pocock, 1901: 553 (type species by original designation *Eurypelma seemanni* Pickard-Cambridge, 1897). First synonymized with *Rhechostica* by Raven (1985: 149).
Delopelma Petrunkevitch, 1939: 567 (type species by original designation *Eurypelma marxi* Simon, 1891). First synonymized with *Rhechostica* by Raven (1985: 151).
Gosipelma Chamberlin, 1940: 4 (type species by original designation *Gosipelma angusi* Chamberlin, 1940). Originally described as a subgenus of *Aphonopelma*, but never elevated to full generic status. First synonymized with *Rhechostica* by Raven (1985: 153).
Chaunopelma Chamberlin, 1940: 30 (type species by original designation *Delopelma radinum* Chamberlin & Ivie, 1939). First synonymized with *Rhechostica* by Raven (1985: 151).
Apachepelma Smith, 1994: 45 (type species by original designation *Aphonopelma paloma* Prentice, 1992). First synonymized with *Aphonopelma* by Prentice (1997: 147).

##### Type species.

*Eurypelma seemanni* F.O. Pickard-Cambridge, 1897; female holotype from Puerto Culebra, Pacific coast, W of Liberia, Guanacaste province, Costa Rica, coll. Dr. Seeman; deposited in BMNH. [examined]

*Eurypelma seemanni* F. O. Pickard-Cambridge, 1897: 26.

*Aphonopelma seemanni* Pocock, 1901: 553.

*Aphonopelma seemanni* Valerio, 1980: 274.

*Rhechostica seemanni* Raven, 1985: 149.

*Aphonopelma seemanni* Smith, 1995: 141.

##### Diagnosis.

From Prentice (1997, p. 147)

“The genus *Aphonopelma* is distinguished from all other theraphosid genera by the following combination of characters: (1) no known external stridulation organs; (2) hair-like or spiniform plumose setae on the prolateral surface of the trochanter and femur of leg I and on the retrolateral surface of the coxa and trochanter of the pedipalp; (3) type I urticating setae only; (4) corresponding segments of all legs approximately the same width in females (femur III in males sometimes laterally swollen); (5) scopula of tarsus IV usually entire, if divided then only partially and narrowly by line of setae; (6) setae on the prolateral surface of coxa I hair-like and not basally swollen, spiniform and basally swollen, or distinctly stout and thorn-like; (7) metatarsus I flexing against the lower process of the tibial spur, with either the apex of the spur contacting the ventral surface of the metatarsus or the outer edge of the spur in the apical half contacting the prolateral surface of the metatarsus; (8) and the lower process of the tibial spur curving prolaterodistally and widening apically, usually equipped with at least one apical or preapical megaspine, and the upper shorter process less stout basally, relatively uniform in diameter throughout its length, and equipped on its inner surface with at least one stout, basally articulated megaspine.”

**Future nomenclatural status of *Aphonopelma***

Due to our inability to conduct fieldwork in Mexico and Central America, a comprehensive taxonomic revision of *Aphonopelma* (and other closely related genera) is not feasible at this time. However, we do anticipate future genus-level nomenclatural changes for those species found in the United States. As presently defined, we have reason to believe that *Aphonopelma* is not monophyletic. Our choice of outgroups places the *Aphonopelma* species from the United States closer to the genus *Sericopelma* than to the Central American species of *Aphonopelma* (Fig. 8). This has nomenclatural implications because the type species (*Aphonopelma seemanni*) from Costa Rica is undoubtedly grouped with the other Central American *Aphonopelma* material (*pers. obs.*). Unfortunately, it is not possible to resurrect a synonymized generic name (e.g., *Dugesiella* or *Delopelma*) for the *Aphonopelma* species in the United States until researchers can more thoroughly sample material from Mexico and Central America (see Prentice 1997, Smith 2010).

***Aphonopelma***

##### within the United States

*Aphonopelma anax* (Chamberlin, 1940)

*Aphonopelma armada* (Chamberlin, 1940)

*Aphonopelma atomicum* Hamilton, sp. n.

*Aphonopelma catalina* Hamilton, Hendrixson & Bond, sp. n.

*Aphonopelma chalcodes* Chamberlin, 1940

*Aphonopelma chiricahua* Hamilton, Hendrixson & Bond, sp. n.

*Aphonopelma eutylenum* Chamberlin, 1940

*Aphonopelma gabeli* Smith, 1995

*Aphonopelma hentzi* (Girard, 1852)

*Aphonopelma icenoglei* Hamilton, Hendrixson & Bond, sp. n.

*Aphonopelma iodius* (Chamberlin & Ivie, 1939)

*Aphonopelma johnnycashi* Hamilton, sp. n.

*Aphonopelma joshua* Prentice, 1997

*Aphonopelma madera* Hamilton, Hendrixson & Bond, sp. n.

*Aphonopelma mareki* Hamilton, Hendrixson & Bond, sp. n.

*Aphonopelma marxi* (Simon, 1891)

*Aphonopelma moderatum* (Chamberlin & Ivie, 1939)

*Aphonopelma moellendorfi* Hamilton, sp. n.

*Aphonopelma mojave* Prentice, 1997

*Aphonopelma paloma* Prentice, 1993

*Aphonopelma parvum* Hamilton, Hendrixson & Bond, sp. n.

*Aphonopelma peloncillo* Hamilton, Hendrixson & Bond, sp. n.

*Aphonopelma phasmus* Chamberlin, 1940

*Aphonopelma prenticei* Hamilton, Hendrixson & Bond, sp. n.

*Aphonopelma saguaro* Hamilton, sp. n.

*Aphonopelma steindachneri* (Ausserer, 1875)

*Aphonopelma superstitionense* Hamilton, Hendrixson & Bond, sp. n.

*Aphonopelma vorhiesi* (Chamberlin & Ivie, 1939)

*Aphonopelma xwalxwal* Hamilton, sp. n.

### Key to the male *Aphonopelma* of the United States

**Table:** 

	Arizona	**A1**
	California	**B1**
	Colorado, Kansas, Oklahoma, Missouri, Arkansas, and Louisiana	**C1**
	Nevada and Utah	**D1**
	New Mexico	**E1**
	Texas	**F1**

#### A1. Key to the male *Aphonopelma* of Arizona

**Table:** 

1	Stout setae on the prolateral surface of coxa I; distribution restricted to southern Greenlee County	***Aphonopelma hentzi***
–	No stout setae on the prolateral surface of coxa I; widespread distribution	**2**
2	Small species (Cl ≤ 9 mm); generally found in desert, grassland, and/or chaparral habitats	**3**
–	Small to medium-sized species (Cl 6–12 mm); generally distributed throughout mid- to high-elevation forested habitats; possessing a black carapace – never blonde, tan, or brown	**5** ^1^
–	Medium to large-sized species (Cl ≥ 9 mm) found in a variety of habitats (but seldom found in higher-elevation forested habitats); carapace blonde, tan, brown, or black	**6**
3	Possessing a swollen or slightly swollen femur III	**4** ^1^
–	Lacks a swollen femur III; distributed east of Tucson in Cochise, Graham, and Greenlee Counties	***Aphonopelma parvum***
4	Distributed across the Mojave Desert and adjacent sections of the Sonoran Desert in western Arizona	***Aphonopelma prenticei***
–	Distributed across lower-elevation sections of the Sonoran Desert in southern Arizona	***Aphonopelma paloma***
–	Distributed across mid- to high-elevation chaparral and forested habitats north of the Phoenix Metropolitan Area	***Aphonopelma mareki***
–	Distributed at the bottom of Grand Canyon in the vicinity of Phantom Ranch	***Aphonopelma phasmus***
–	Distributed across the southern foothills and canyons of the Santa Catalina and Rincon Mountains east of Tucson	***Aphonopelma saguaro***
–	Distributed east of the Phoenix Metropolitan Area in the foothills and canyons of the Superstition Mountains	***Aphonopelma superstitionense***
5	Distributed north and east of the Phoenix Metropolitan Area across the Colorado Plateau and on isolated mountains (e.g., Four Peaks, Mount Ord)	***Aphonopelma marxi***
–	Distributed across the Santa Catalina Mountains	***Aphonopelma catalina***
–	Distributed across the Huachuca, Pajarito, Patagonia, and Santa Rita Mountains	***Aphonopelma madera***
–	Distributed across the Chiricahua Mountains	***Aphonopelma chiricahua***
6	Carapace blonde, tan, or brown	**7**
–	Carapace black	**8**
7	Distributed across the Colorado Plateau north of the Colorado River	***Aphonopelma iodius***
–	Widespread throughout Arizona but never north of the Colorado River	***Aphonopelma chalcodes***
8	PT1/M1 ≤ 0.68	***Aphonopelma gabeli***
–	PT1/M1 ≥ 0.74	***Aphonopelma peloncillo*** and ***Aphonopelma vorhiesi*** ^2^

#### B1. Key to the male *Aphonopelma* of California

**Table:** 

1	Metatarsus IV scopulation ≥ 60%	**2**
–	Metatarsus IV scopulation ≤ 60%	**4**
2	Possessing a black carapace; distributed across the plains and foothills of the western Sierra Nevada Mountains	***Aphonopelma johnnycashi***
–	Possessing a tan or brown carapace	**3** ^1^
3	Distributed west of the Mojave Desert, from the western part of the Transverse Range and down the southern California coast, along the Peninsular Ranges	***Aphonopelma eutylenum***
–	Distributed from the Bay Area south along the Coast Ranges, west of the Central Valley, across the Transverse Range and into the Mojave Desert	***Aphonopelma iodius***
4	Possessing stout setae on the medial surface of the sternum	**5**
–	Lacks stout setae on the medial surface of the sternum	**6**
5	F4/T4 ≤ 1.05; distributed across the mountains and foothills west of the Coachella Valley and south to the Borrego Springs area; fall breeding period	***Aphonopelma xwalxwal***
–	F4/T4 ≥1.07; distributed in and around Joshua Tree National Park; summer breeding period	***Aphonopelma joshua***
6	Medium to large-sized species (Cl ≥ 9 mm); distributed across southwestern California but not within the Mojave Desert	***Aphonopelma steindachneri***
–	Small species (Cl ≤ 9mm); distributed across the Mojave Desert	**7**
7	Femur III swollen or slightly swollen	**8**
–	Femur III not swollen	**9** ^1^
8	A1/F3 ≥ 0.58; distributed across the Panamint Range and eastern San Bernardino County	***Aphonopelma prenticei***
–	A1/F3 ≤0.56; narrowly distributed across the Amargosa Range in southeastern Inyo County	***Aphonopelma atomicum***
9	Distributed across the northwestern Mojave Desert in eastern Kern and northwestern San Bernardino Counties	***Aphonopelma mojave***
–	Distributed throughout the southern Mojave Desert along the foothills on the northern side of the San Gabriel and San Bernardino Mountains, east to the Coxcomb Mountains and south into Joshua Tree National Park, north to Kramer Junction and Barstow	***Aphonopelma icenoglei***

#### C1. Key to the male *Aphonopelma* of Colorado, Kansas, Oklahoma, Missouri, Arkansas, and Louisiana

**Table:** 

1	Carapace tan, brown, or copper; possessing stout setae on the prolateral surface of coxa I; distributed east of the Rocky Mountains across Colorado, Kansas, Oklahoma, Missouri, Arkansas, and Louisiana	***Aphonopelma hentzi***
–	Carapace black; lacks stout setae on the prolateral surface of coxa I; distributed west of the Rocky Mountains across southwestern Colorado	***Aphonopelma marxi***

#### D1. Key to the male *Aphonopelma* of Nevada and Utah

**Table:** 

1	Metatarsus IV scopulation ≥ 60%; widespread distribution	***Aphonopelma iodius***
–	Metatarsus IV scopulation ≤ 60%	**2**
2	Distributed across the Colorado Plateau in southeastern Utah	***Aphonopelma marxi***
–	Distributed across the Mojave Desert in southwestern Utah and/or southern Nevada	**3** ^1^
3	A1/F3 ≥ 0.58; widespread across the northeastern Mojave Desert	***Aphonopelma prenticei***
–	A1/F3 ≤ 0.58; distribution restricted to the Amargosa Valley and Nevada Test Site near Mercury, NV	***Aphonopelma atomicum***

#### E1. Key to the male *Aphonopelma* of New Mexico

**Table:** 

1	Medium to large-sized species (Cl ≥ 9 mm)	**2**
–	Small species (Cl ≤ 9 mm) distributed across the extreme southwestern part of the state (Hidalgo County)	***Aphonopelma parvum***
2	Possessing stout setae on the prolateral surface of coxa I; possessing a tan, brown, or copper carapace	***Aphonopelma hentzi***
–	Lacks stout setae on the prolateral surface of coxa I; possessing a black carapace	**3**
3	Distributed across the northern half of the state and western part of the Rockies, inhabiting high-elevation pine forest and sagebrush steppe	***Aphonopelma marxi***
–	Distributed across the southern half of the state	**4**
4	PT1/M1 ≤ 0.68	***Aphonopelma gabeli***
–	PT1/M1 ≥ 0.74	**5** ^3^
5	Distribution restricted to the southeastern Peloncillo Mountains; males active mostly during mid-summer	***Aphonopelma peloncillo***
–	Distribution widespread across southern New Mexico; males active mostly during late summer and early fall	***Aphonopelma vorhiesi***

#### F1. Key to the male *Aphonopelma* of Texas

**Table:** 

1	Possessing a short and stout embolus; distributed across South Texas	***Aphonopelma anax***
–	Possessing a long, thin, and tapering embolus; widespread	**2**
2	Possessing a tan, brown, or copper carapace	**3**
–	Possessing a black carapace	**4**
3	Metatarsus III scopulation ≤ 63%; stout setae on the prolateral surface of coxa I present along dorsal, posterior, and ventral margins, but lacking from the center and anterior margin	***Aphonopelma armada***
–	Metatarsus III scopulation ≥ 69%; stout setae abundant across the prolateral surface of coxa I	***Aphonopelma hentzi***
4	M1/M4 ≤ 0.74	***Aphonopelma gabeli***
–	M1/M4 ≥ 0.75	***Aphonopelma moderatum*** and ***Aphonopelma moellendorfi*** ^4^

^1^ Species that key here are morphologically indistinguishable for the most part but can be identified based on their localities and molecular data.

^2^ Mature males of these two species are morphologically indistinguishable and cannot be identified using morphological criteria when they co-occur in southeastern Cochise County, but can be differentiated using molecular data. *Aphonopelma vorhiesi* is likely the correct determination if the specimen originates from Graham, Pima, Pinal, Santa Cruz, or western Cochise Counties.

^3^ Species that key here are morphologically indistinguishable for the most part but can be identified based on their localities, the timing of their breeding periods, and molecular data.

^4^ Mature males of these two species are morphologically indistinguishable and cannot be identified using morphological criteria when they co-occur, but can be differentiated using molecular data. *Aphonopelma moderatum* reaches its northernmost distribution in Val Verde County but is largely distributed to the south along the Rio Grande Valley. *Aphonopelma moellendorfi* reaches its easternmost distribution in Val Verde County with other specimens having been located in extreme West Texas. When these two species come into syntopy (southeastern Val Verde County), males can easily be distinguished prior to their ultimate molt due to their phenotypic differences: *Aphonopelma moderatum* possess distinct alternating black and tan/orange bands on its legs whereas *Aphonopelma moellendorfi* is more uniformly brown.

### Key to the female *Aphonopelma* of the United States

**Table:** 

	Arizona	**A2**
	California	**B2**
	Colorado, Kansas, Oklahoma, Missouri, Arkansas, and Louisiana	**C2**
	Nevada and Utah	**D2**
	New Mexico	**E2**
	Texas	**F2**

#### A2. Key to the female *Aphonopelma* of Arizona ^1^

**Table:** 

1	Possessing stout setae on the prolateral surface of coxa I; distribution restricted to southern Greenlee County	***Aphonopelma hentzi***
–	Lacks stout setae on the prolateral surface of coxa I; widespread	**2**
2	Small species (Cl ≤ 9 mm); generally found in desert, grassland, and/or chaparral habitats	**3** ^2^
–	Small to large-sized species (Cl 7.5–16.5 mm); generally distributed throughout mid- to high-elevation forested habitats; possessing black or gray carapace – never blonde, tan, or brown	**4** ^2^
–	Medium to large-sized species (Cl ≥ 9 mm) found in a variety of habitats (but seldom found in higher-elevation forested habitats); carapace blonde, tan, brown, black, or gray	**6**
3	Distributed east of Tucson in Cochise, Graham, and Greenlee Counties	***Aphonopelma parvum***
–	Distributed across the Mojave Desert and adjacent sections of the Sonoran Desert in western Arizona	***Aphonopelma prenticei***
–	Distributed across lower-elevation sections of the Sonoran Desert in southern Arizona	***Aphonopelma paloma***
–	Distributed across mid- to high-elevation chaparral and forested habitats north of the Phoenix Metropolitan Area	***Aphonopelma mareki***
–	Distributed across the southern foothills and canyons of the Santa Catalina and Rincon Mountains east of Tucson	***Aphonopelma saguaro***
–	Distributed east of the Phoenix Metropolitan Area in the foothills and canyons of the Superstition Mountains	***Aphonopelma superstitionense***
4	T1/T4 ≥ 1.06; distributed north and east of the Phoenix Metropolitan Area across the Colorado Plateau and on isolated mountains (e.g., Four Peaks, Mount Ord)	***Aphonopelma marxi***
–	T1/T4 ≤1.02; distributed across the Madrean Sky Islands in southeastern Arizona	**5**
5	Distributed across the Santa Catalina Mountains	***Aphonopelma catalina***
–	Distributed across the Huachuca, Pajarito, Patagonia, and Santa Rita Mountains	***Aphonopelma madera***
–	Distributed across the Chiricahua Mountains	***Aphonopelma chiricahua***
6	Metatarsus IV scopulation ≥ 55%	**7**
–	Metatarsus IV scopulation ≤ 55%	**8**
7	Distributed across the Colorado Plateau north of the Colorado River	***Aphonopelma iodius***
–	Widespread throughout Arizona, but never north of the Colorado River	***Aphonopelma chalcodes***
8	Possessing spermathecae with capitate bulbs	***Aphonopelma peloncillo*** and ***Aphonopelma vorhiesi*** ^3^
–	Possessing short, wide, and slightly rounded spermathecae without capitate bulbs	***Aphonopelma gabeli***

#### B2. Key to the female *Aphonopelma* of California ^4^

**Table:** 

1	Metatarsus IV scopulation ≥ 55%	**2** ^2^
–	Metatarsus IV scopulation ≤ 55%	**3**
2	Distributed across the plains and foothills of the western Sierra Nevada Mountains	***Aphonopelma johnnycashi***
–	Distributed west of the Mojave Desert, from the western part of the Transverse Range and down the southern California coast, along the Peninsular Ranges	***Aphonopelma eutylenum***
–	Distributed from the Bay Area south along the Coast Ranges, west of the Central Valley, across the Transverse Range and into the Mojave Desert	***Aphonopelma iodius***
3	Large species (Cl ≥ 9 mm); distributed across southwestern California but not within the Mojave Desert	***Aphonopelma steindachneri***
–	Small species (Cl ≤ 9 mm); distributed across the Mojave and portions of the Sonoran (Colorado) Deserts	**4** ^2^
4	Distributed across the northwestern Mojave Desert in eastern Kern and northwestern San Bernardino Counties	***Aphonopelma mojave***
–	Distributed in and around Joshua Tree National Park; F1/M4 ≤ 1.04; L1/Cl ≤ 2.87	***Aphonopelma joshua*** ^5^
–	Distributed across the Panamint Range and eastern San Bernardino County	***Aphonopelma prenticei***
–	Distributed across the Amargosa Range in southeastern Inyo County	***Aphonopelma atomicum***
–	Distributed throughout the southern Mojave Desert along the foothills on the northern side of the San Gabriel and San Bernardino Mountains, east to the Coxcomb Mountains and south into Joshua Tree National Park, north to Kramer Junction and Barstow; F1/M4 ≥ 1.07; L1/Cl ≥ 2.92	***Aphonopelma icenoglei*** ^5^

#### C2. Key to the female *Aphonopelma* of Colorado, Kansas, Oklahoma, Missouri, Arkansas, and Louisiana

**Table:** 

1	Carapace tan or brown; possessing stout setae on the prolateral surface of coxa I; distributed east of the Rocky Mountains across Colorado, Kansas, Oklahoma, Missouri, Arkansas, and Louisiana	***Aphonopelma hentzi***
–	Carapace black; lacks stout setae on the prolateral surface of coxa I; distributed west of the Rocky Mountains across southwestern Colorado	***Aphonopelma marxi***

#### D2. Key to the female *Aphonopelma* of Nevada and Utah

**Table:** 

1	Metatarsus IV scopulation ≥ 60%; widespread distribution	***Aphonopelma iodius***
–	Metatarsus IV scopulation ≤ 50%	**2**
2	Distributed across the Colorado Plateau in southeastern Utah	***Aphonopelma marxi***
–	Distributed across the Mojave Desert in southwestern Utah and/or southern Nevada	**3** ^2^
3	Cl/M4 ≥ 1.31; widespread across the northeastern Mojave Desert	***Aphonopelma prenticei***
–	Cl/M4 ≤ 1.28; distribution restricted to the Amargosa Valley and Nevada Test Site near Mercury, NV	***Aphonopelma atomicum***

#### E2. Key to the female *Aphonopelma* of New Mexico

**Table:** 

1	Possessing spermathecae with capitate bulbs	**2**
–	Possessing short, wide, and slightly rounded spermathecae without capitate bulbs; anterior margin of carapace broad, associated with robust chelicerae	***Aphonopelma gabeli***
2	Possessing stout setae on the prolateral surface of coxa I; possessing a tan or brown carapace	***Aphonopelma hentzi***
–	Lacks stout setae on the prolateral surface of coxa I; possessing a black or gray carapace	**3**
3	Distributed across the northern half of the state and western part of the Rockies, inhabiting high-elevation pine forest and sagebrush steppe	***Aphonopelma marxi***
–	Distributed across the southern half of the state	**4**
4	Medium to large-sized species (Cl ≥ 10 mm)	**5** ^2^
–	Small species (Cl ≤ 9 mm); distributed in the extreme southwestern part of the state (Hidalgo County)	***Aphonopelma parvum***
5	Distribution restricted to the southeastern Peloncillo Mountains	***Aphonopelma peloncillo***
–	Distribution widespread across southern New Mexico	***Aphonopelma vorhiesi***

#### F2. Key to the female *Aphonopelma* of Texas ^6^

**Table:** 

1	Possessing spermathecae with capitate bulbs	**2**
–	Possessing short, wide, and slightly rounded spermathecae without capitate bulbs	**4**
2	Possessing stout setae on the prolateral surface of coxa I; all leg segments uniformly colored brown or black	**3**
–	Lacks stout setae on the prolateral surface of coxa I; legs distinctly colored with the femora and tibiae generally orange or tan and the patellae, metatarsi, and tarsi dark brown to black (if the leg segments are uniformly colored, they will be orange or tan, never brown or black)	***Aphonopelma moderatum***
3	Stout setae on the prolateral surface of coxa I present along dorsal, posterior, and ventral margins, but lacking from the center and anterior margin; metatarsi I, II, and III distinctly flared; species with overall shiny, lustrous appearance	***Aphonopelma armada***
–	Stout setae abundant across the prolateral surface of coxa I; metatarsi not distinctly flared; species with overall hirsute appearance	***Aphonopelma hentzi***
4	Distributed across South Texas; possessing a tan or brown carapace with a large robust appearance	***Aphonopelma anax***
–	Distributed across the Chihuahuan Desert and southwestern High Plains in West Texas; possessing a brownish-gray carapace; anterior margin of carapace broad, associated with robust chelicerae	***Aphonopelma gabeli***

^1^ Adult females of *Aphonopelma phasmus* remain unknown.

^2^ Species that key here are morphologically indistinguishable for the most part but can be identified based on their localities and molecular data.

^3^ Mature females of these two species can be morphologically indistinguishable when they co-occur in southeastern Cochise County. *Aphonopelma vorhiesi* is likely the correct determination if the specimen originates from Graham, Pima, Pinal, Santa Cruz, or western Cochise Counties.

^4^ Adult females of *Aphonopelma xwalxwal* remain unknown.

^5^ *Aphonopelma joshua* and *Aphonopelma icenoglei* are syntopic at various locations in and around Joshua Tree National Park. Females of *Aphonopelma joshua* generally can be diagnosed by possessing tarsi IV divided by setae (Prentice 1997) but this characteristic can be difficult to assess. Molecular data should be used to confirm the identity of specimens from this area.

^6^ Adult females of *Aphonopelma moellendorfi* remain unknown.

### Aphonopelma anax

Taxon classificationAnimaliaAraneaeTheraphosidae

(Chamberlin, 1940)

**Figure 9.**
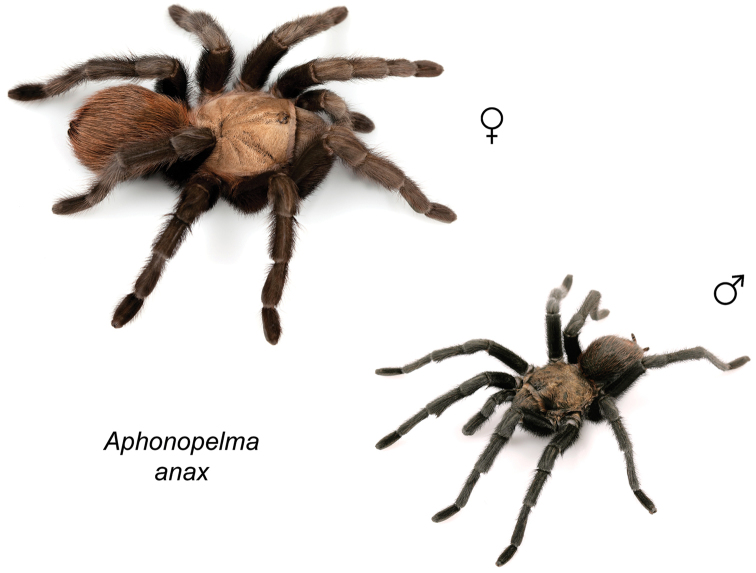
*Aphonopelma anax* (Chamberlin, 1940) specimens, live photographs. Female (L) - APH_0524; Male (R) - APH_3122.

**Figure 10.**
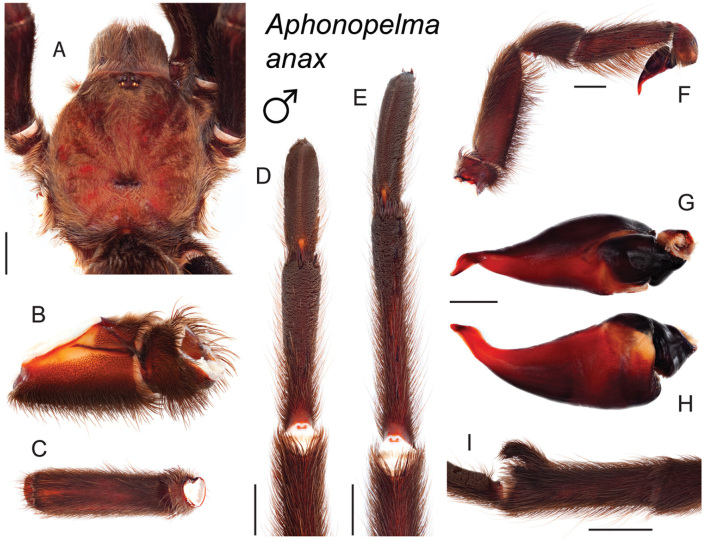
*Aphonopelma anax* (Chamberlin, 1940). **A–I** male specimen, APH_0924 **A** dorsal view of carapace, scale bar = 5mm **B** prolateral view of coxa I **C** dorsal view of femur III **D** ventral view of metatarsus III, scale bar = 4mm **E** ventral view of metatarsus IV, scale bar = 4mm **F** prolateral view of L pedipalp and palpal tibia, scale bar = 3mm **G** dorsal view of palpal bulb **H** retrolateral view of palpal bulb, scale bar = 1mm **I** prolateral view of tibia I (mating clasper), scale bar = 6mm.

**Figure 11.**
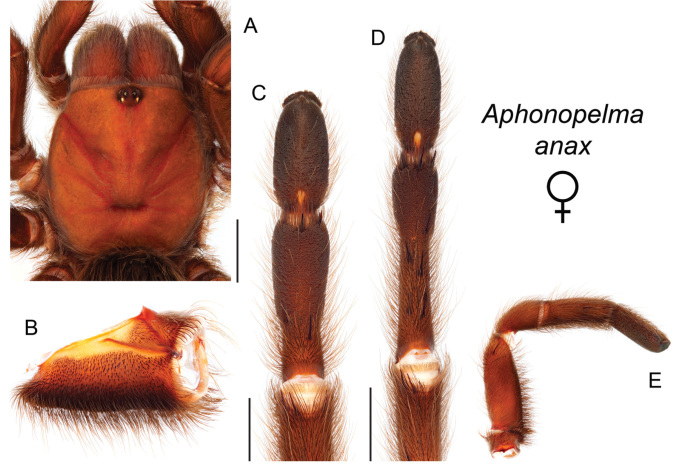
*Aphonopelma anax* (Chamberlin, 1940). **A–E** female specimen, APH_0857 **A** dorsal view of carapace, scale bar = 8mm **B** prolateral view of coxa I **C** ventral view of metatarsus III, scale bar = 4mm **D** ventral view of metatarsus IV, scale bar = 5.5mm **E** prolateral view of L pedipalp and palpal tibia.

**Figure 12.**
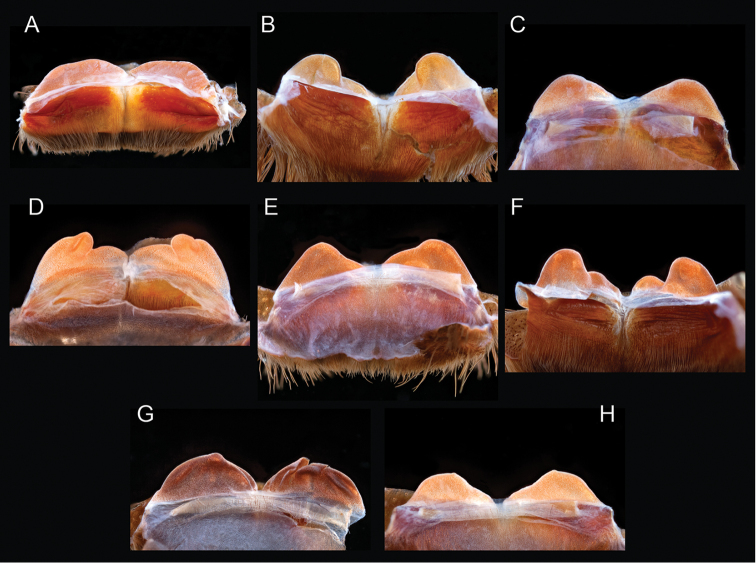
*Aphonopelma anax* (Chamberlin, 1940). **A–H** cleared spermathecae **A** *anax* allotype **B** *breenei* holotype **C** *harlingenum* paratype **D** APH_0056 **E** APH_0529 **F** APH_0857 **G** APH_0858 **H** APH_0859.

**Figure 13.**
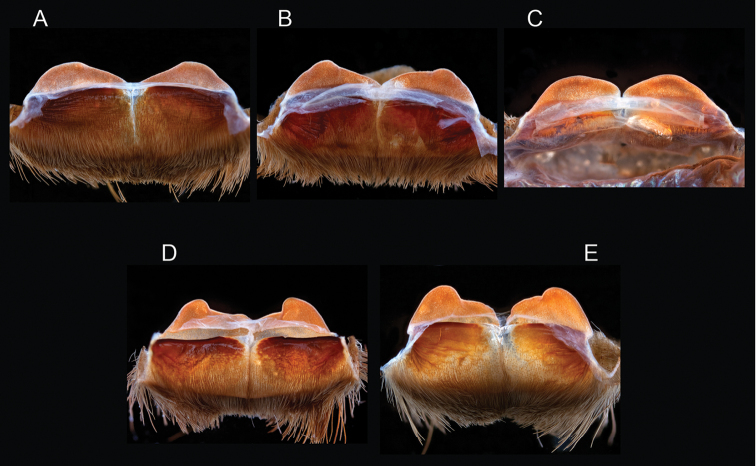
*Aphonopelma anax* (Chamberlin, 1940). **A–E** cleared spermathecae **A** APH_0871 **B** APH_0899 **C** APH_0902 **D** APH_1278 **E** APH_1280.

**Figure 14.**
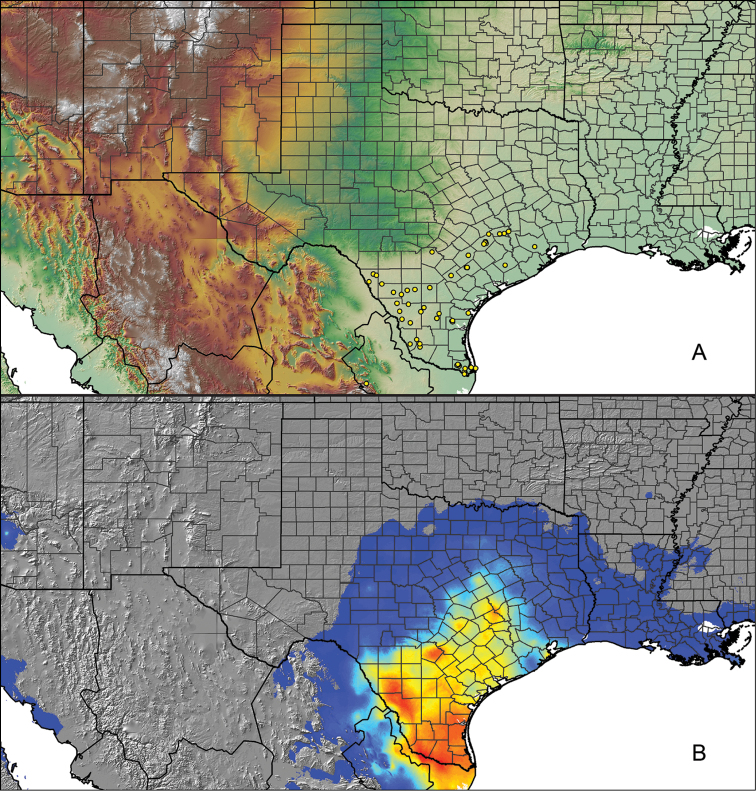
*Aphonopelma anax* (Chamberlin, 1940). **A** distribution of known specimens **B** predicted distribution; warmer colors (red, orange, yellow) represent areas of high probability of occurrence, cooler colors (blue shades) represent areas of low probability of occurrence.

Figures 9 , 10 , 11 , 12 , 13 , 14 , Suppl. material 4

Dugesiella anax Chamberlin, 1940: 34; male holotype and female allotype from Kingsville, Kleberg Co., Texas, 27.515869 -97.856109^5^, elev. 58ft., no collecting date, coll. Prof. J.C. Cross; 3 female paratypes from Harlingen, Cameron Co., Texas, 26.190631 -97.696103^5^, elev. 40ft., 1939, coll. Bryce Brown; deposited in AMNH. [examined]
Rhechostica anax Raven, 1985: 149.
Aphonopelma anax Smith, 1995: 71.
Aphonopelma breenei Smith, 1995: 78; female holotype from Harlingen, Cameron Co., Texas, 26.190631 -97.696103^5^, elev. 40ft., 1939, coll. Bryce Brown; deposited in AMNH. [examined] **syn. n.**

#### Diagnosis.

*Aphonopelma anax* (Fig. 9) is a member of the *Hentzi* species group and can be identified by a combination of morphological, molecular, and geographic characteristics. Nuclear DNA identifies *Aphonopelma anax* as a phylogenetically distinct monophyletic lineage (Fig. 8), supported as the sister lineage to *Aphonopelma hentzi*, and closely related to *Aphonopelma armada*. Male *Aphonopelma anax* can be distinguished from geographically proximate species by their unique palpal bulbs - stout and wide with distinct keels on the embolus (Fig. 10). Females can be distinguished from geographically proximate species by their unique spermathecae - short, wide, slightly rounded, without capitate bulbs (Figs 12–13). Significant measurements that distinguish male *Aphonopelma anax* from its closely related phylogenetic and syntopic species are Cl, M3, and F4. Male *Aphonopelma anax* can be distinguished by possessing a larger F4/M4 (≥0.94; 0.94–1.04) than *Aphonopelma armada* (≤0.92; 0.86–0.92); a larger Cl/M1 (≥1.36; 1.36–1.63) than *moderatum* (≤1.30; 1.13–1.30) and *Aphonopelma moellendorfi* sp. n. (≤1.31; 1.10–1.31); and a larger F1/M3 (≥1.28; 1.28–1.43) than *Aphonopelma gabeli* (≤1.24; 1.18–1.24). There are no significant measurements that separate male *Aphonopelma anax* from *Aphonopelma hentzi*. Significant measurements that distinguish female *Aphonopelma anax* from its closely related phylogenetic and syntopic species are P1 and T3. Female *Aphonopelma anax* can be distinguished by possessing a larger P1/T3 (≥13.88; 13.88–19.15) than *Aphonopelma armada* (≤13.84; 9.93–13.84) and *Aphonopelma moderatum* (≤13.12; 8.14–13.12). There are no significant measurements that separate female *Aphonopelma anax* from *Aphonopelma gabeli* or *Aphonopelma hentzi*. Females of *Aphonopelma moellendorfi* are unknown and cannot be compared.

#### Descriptions.

Male and female originally described by Chamberlin (1940).

#### Redescription of male exemplar

(APH_0924; Fig. 10). *Specimen preparation and condition*: Specimen collected live crossing road, preserved in 80% ethanol; deposited in AUMNH; original coloration faded due to preservation. Left legs I, III, IV, and left pedipalp removed for measurements and photographs; stored in vial with specimen. Right leg III removed for DNA and stored at -80°C in the AUMNH (Auburn, AL). *General coloration*: Generally black or faded to brown. *Cephalothorax*: Carapace 19.10 mm long, 18.28 mm wide; densely clothed with brown/golden iridescent pubescence appressed to surface; fringe covered in long setae not closely appressed to surface; foveal groove medium deep and straight to slightly procurved; pars cephalica region rises gradually from foveal groove, gently arching anteriorly toward ocular area; AER slightly procurved, PER recurved; normal sized chelicerae; clypeus extends forward on a very slight curve; LBl 2.96, LBw 2.94; sternum hirsute, clothed with short length black, densely packed setae. *Abdomen*: Densely clothed in short black pubescence with numerous longer, lighter setae interspersed (generally red or orange *in situ*); dense dorsal patch of black Type I urticating bristles (Cooke et al. 1972). *Legs*: Thick and hirsute; densely clothed in short black/brown pubescence. Metatarsus I slightly curved. F1 18.77; F1w 4.74; P1 8.10; T1 14.69; M1 13.41; A1 9.23; F3 15.19; F3w 4.61; P3 6.75; T3 12.07; M3 13.77; A3 9.42; F4 18.58; F4w 4.8; P4 8.16; T4 14.50; M4 19.51; A4 11.15; femur III is normal - not noticeably swollen or wider than other legs. All tarsi fully scopulate. Extent of metatarsal scopulation: leg III (SC3) = 68.9%; leg IV (SC4) = 43.4%. Two ventral spinose setae on metatarsus III; six ventral spinose setae on metatarsus IV. *Coxa I*: Prolateral surface a mix of fine, hair-like and medium tapered setae. The interior face of the retrolateral branch of the tibial apophyses possesses a very large megaspine that projects anteriorly. *Pedipalps*: Hirsute; densely clothed in the same setal color as the other legs, with numerous longer ventral setae; one spinose seta at the apical, prolateral femur and three spinose setae on the prolateral tibia; PTl 9.45, PTw 2.90. When extended, embolus tapers and rapidly curves up and to the retrolateral side near apex; embolus wide and thick, with smooth dorsal and ventral keels.

**Variation (11).** Cl 14.371–21.97 (17.885±0.7), Cw 13.32–19.84 (16.557±0.62), LBl 1.96–2.96 (2.341±0.09), LBw 2.16–3.02 (2.711±0.08), F1 14.1–19.3 (16.839±0.55), F1w 3.52–5.1 (4.392±0.15), P1 6.12–8.1 (7.105±0.22), T1 11.32–15.71 (13.314±0.39), M1 9.69–13.43 (11.878±0.4), A1 6.9–9.6 (8.499±0.28), L1 length 48.47–65.97 (57.635±1.73), F3 11.39–16.14 (13.868±0.48), F3w 3.41–5.03 (4.132±0.16), P3 4.86–7.65 (6.035±0.27), T3 7.91–12.16 (10.428±0.43), M3 9.89–14.89 (12.572±0.49), A3 6.93–9.45 (8.291±0.27), L3 length 41.32–59.2 (51.194±1.82), F4 13.52–19.31 (16.56±0.58), F4w 3.22–4.83 (4.094±0.17), P4 5.5–8.16 (6.647±0.26), T4 11.15–15.56 (13.676±0.41), M4 13.66–19.69 (17.13±0.58), A4 8.1–11.15 (9.504±0.3), L4 length 52.79–72.08 (64.171±1.92), PTl 7.187–10.136 (8.709±0.27), PTw 2.246–3.32 (2.882±0.09), SC3 ratio 0.607–0.805 (0.713±0.02), SC4 ratio 0.351–0.524 (0.44±0.02), Coxa 1 setae = thick tapered, F3 condition = normal/slightly swollen.

#### Redescription of female exemplar

(APH_0857; Figs 11–13). *Specimen preparation and condition*: Specimen collected live from burrow, preserved in 80% ethanol; deposited in AUMNH; original coloration faded due to preservation. Left legs I, III, IV, and pedipalp removed for photographs and measurements; stored in vial with specimen. Right leg III removed for DNA and stored at -80°C in the AUMNH (Auburn, AL). Genital plate with spermathecae removed and cleared, stored in vial with specimen. *General coloration*: Faded black and light brown, medium length, dense setae cover body. *Cephalothorax*: Carapace 21.37 mm long, 18.26 mm wide; densely clothed with light brown pubescence closely appressed to surface; fringe densely covered in long setae; foveal groove medium deep and straight; pars cephalica region rises from thoracic furrow more steeply than male, gently arching anteriorly toward ocular area; AER procurved, PER strongly recurved; clypeus extends forward on a slight curve; LBl 2.67, LBw 3.35; sternum hirsute, clothed with light black/dark brown, medium length dense setae. *Abdomen*: Densely clothed dorsally in short black setae with numerous longer, lighter setae interspersed (generally red or orange *in situ*); dense dorsal patch of black Type I urticating bristles (Cooke et al. 1972); ventral side with short, dense black setae. *Spermathecae*: Paired and separate, possessing dual bulges with the smaller near the interior, with wide bases that are not fused. *Legs*: Thick and hirsute; densely clothed in a mix of short black/brown pubescence setae. *Coxa I*: Prolateral surface a mix of fine, hair-like and thick tapered setae. F1 16.02; F1w 4.88; P1 7.53; T1 12.06; M1 9.18; A1 7.54; F3 12.72; F3w 4.56; P3 6.76; T3 9.29; M3 10.09; A3 7.54; F4 16.53; F4w 4.73; P4 7.08; T4 13.31; M4 13.26; A4 8.79. All tarsi fully scopulate. Extent of metatarsal scopulation: leg III (SC3) = 64.4%; leg IV (SC4) = 47.3%. Two ventral spinose setae on metatarsus III; seven ventral spinose setae on metatarsus IV. *Pedipalps*: Densely clothed in the same setal color as the other legs; one spinose seta on the apical, prolateral femur and five spinose setae on the prolateral tibia.

**Variation (13).** Cl 16.06–23.8 (20.27±0.79), Cw 14.9–21.73 (17.951±0.62), LBl 2.45–3.79 (2.878±0.1), LBw 2.71–4.3 (3.478±0.15), F1 13.12–18.14 (15.181±0.45), F1w 3.87–5.8 (4.945±0.18), P1 5.61–8.59 (7.196±0.26), T1 9.76–13.56 (11.616±0.35), M1 7.0–10.98 (8.911±0.31), A1 5.73–8.34 (7.138±0.19), L1 length 42.75–59.3 (50.042±1.47), F3 10.94–14.63 (12.375±0.37), F3w 3.36–5.13 (4.268±0.17), P3 5.1–7.72 (6.179±0.23), T3 7.45–10.56 (8.816±0.28), M3 8.27–12.05 (9.918±0.34), A3 6.52–8.84 (7.398±0.18), L3 length 38.67–53.7 (44.687±1.26), F4 13.27–18.66 (15.654±0.49), F4w 3.61–5.31 (4.514±0.17), P4 5.34–8.4 (6.55±0.28), T4 9.72–13.98 (12.038±0.35), M4 10.37–16.15 (13.621±0.46), A4 7.01–9.83 (8.373±0.25), L4 length 45.86–66.63 (55.723±1.74), SC3 ratio 0.644–0.763 (0.706±0.01), SC4 ratio 0.368–0.474 (0.433±0.01), Coxa 1 setae = thick tapered. Spermathecae variation can be seen in Figures 12–13.

#### Material examined.

**United States: Texas: Bexar**: Hollywood Park, 220 Mecca, 29.59413 -98.47946 ^2^, 934ft., [APH_0033, 2/6/2006, 1♂, Connor Shannon, Ryan Tubbesing, AUMNH]; **Cameron**: 2100 W. San Marcelo Blvd #158, Brownsville, 25.95835 -97.500489 ^2^, 21ft., [APH_0523, 20/5/2009, 1♂, Lilia Perez, AUMNH]; Brownsville, 25.901747 -97.497484 ^5^, 26ft., [APH_2045, 4/1963, 1♀, 1♂, Ted Beimler, AMNH]; Brownsville, field NE Coffeeport Rd, 25.948055 -97.480915 ^1^, 19ft., [APH_0459-0462, 9/4/09, 1♀, 3 juv, Brent E. Hendrixson, AUMNH]; Harlingen, 26.190631 -97.696103 ^5^, 39ft., [APH_2043, 15/11/1939, 1♀, B. Brown, AMNH]; [APH_2044, 1939, 1♀, Bryce Brown, AMNH]; Harlingen, Dixieland Park, 26.16825 -97.72063 ^1^, 43ft., [APH_1273-1275, 12/5/11, 3 juv, Brent E. Hendrixson, Kate Hall, Austin Deskewies, Alexis Guice, AUMNH]; Harlingen, McKelvey Park, 26.180254 -97.679291 ^1^, 35ft., [APH_0463, 9/4/09, 1 juv, Brent E. Hendrixson, AUMNH]; [APH_1271-1272, 11/5/11, 2 juv, Brent E. Hendrixson, Kate Hall, Austin Deskewies, Alexis Guice, AUMNH]; Harlingen, S of town, 26.145617 -97.661717 ^5^, 41ft., [APH_0924, 2006, 1♂, Dave Moellendorf, AUMNH]; Laguna Vista, 26.100864 -97.290234 ^5^, 12ft., [AUMS_2355, 4/1998, 1♂, R.G. Breene, AUMNH]; [AUMS_2583, 4/1998, 1♂, R.G. Breene, AUMNH]; [AUMS_2609, 1998, 1♂, R.G. Breene, AUMNH]; [AUMS_2613, unknown, 1♀, R.G. Breene, AUMNH]; [AUMS_2690, 4/1998, 2♂, R.G. Breene, AUMNH]; [AUMS_2695, 4/1998, 1♀, R.G. Breene, AUMNH]; [AUMS_3268-3269, 3/1998, 2♂, R.G. Breene, AUMNH]; [AUMS_3271, 4/1998, 1♂, R.G. Breene, AUMNH]; [AUMS_3274, 4/1998, 1♂, R.G. Breene, AUMNH]; [AUMS_3315, 4/1998, 1♂, R.G. Breene, AUMNH]; Laguna Vista, Roloff Park, 26.10243 -97.291 ^1^, 3ft., [APH_0455-0458, 9/4/09, 2♀, 2 juv, Brent E. Hendrixson, AUMNH]; Los Fresnos, 26.071744 -97.476373 ^5^, 23ft., [AUMS_2688, 1999, 1♀, R.G. Breene, AUMNH]; South Padre Island, 26.076567 -97.16315 ^5^, 4ft., [APH_0858, 2006, 1♀, Dave Moellendorf, AUMNH]; **DeWitt**: between Cuero and Westhoff on Hwy 87, 29.1221 -97.410817 ^1^, 303ft., [APH_0859-0861, 9/2008, 3♀, Chris A. Hamilton, AUMNH]; [APH_0947, 9/2008, 1♂, Chris A. Hamilton, AUMNH]; **Dimmit**: 6.7 miles E Maverick County Line on US-277, 28.625443 -100.004038 ^1^, 659ft., [APH_0473, 11/4/2009, 1 juv, Brent E. Hendrixson, AUMNH]; Chaparral Wildlife Management Area, Blocker Pond, 28.3125 -99.40694 ^1^, 570ft., [APH_0025, 12/3/2002, 1♂, Brent E. Hendrixson, WTAMU Herp Class, AUMNH]; 3 miles NW Catarina on US-83, 28.37472 -99.64806 ^1^, 560ft., [APH_0042, 12/3/2000, 1 juv, Brent E. Hendrixson, AUMNH]; **Duval**: 4.4 miles E Hwy-339 on FM-2295, 27.58835 -98.33743 ^1^, 340ft., [APH_1270, 11/5/11, 1 juv, Brent E. Hendrixson, Kate Hall, Austin Deskewies, Alexis Guice, AUMNH]; 5.3 miles SE Webb County Line on Hwy-44, 27.907752 -98.723635 ^1^, 497ft., [APH_0529, 3/6/09, 1♀, Brent E. Hendrixson, Courtney Dugas, Sloan Click, AUMNH]; S of San Diego, TX on Hwy-359, 27.733177 -98.262366 ^1^, 339ft., [APH_0584, 14/6/2009, 1 juv, Brent E. Hendrixson, Courtney Dugas, Sloan Click, AUMNH]; **Fayette**: La Grange, 29.914417 -96.866267 ^1^, 330ft., [APH_0804-0806, 5/2008, 3♀, Chris A. Hamilton, AUMNH]; [APH_0808, 5/2008, 1♀, Chris A. Hamilton, AUMNH]; [APH_0809, 5/2008, 1♀, Chris A. Hamilton, AUMNH]; [APH_0898, 5/2008, 1♂, Chris A. Hamilton, AUMNH]; La Grange, S side of river, 29.874417 -96.850383 ^1^, 345ft., [APH_0811, 7/2008, 1♀, Chris A. Hamilton, AUMNH]; [APH_0810, 5/2008, 1♀, Chris A. Hamilton, AUMNH]; [APH_0899, 5/2008, 1♂, Chris A. Hamilton, AUMNH]; [APH_0901, 5/2008, 1♂, Chris A. Hamilton, AUMNH]; La Grange, on FM155, 29.859333 -96.848033 ^1^, 348ft., [APH_0900, 5/2008, 1♂, Chris A. Hamilton, AUMNH]; La Grange, rest stop on Hwy 77, 29.843383 -96.9091 ^1^, 377ft., [APH_0902-0903, 5/2008, 1♀, 1♂, Chris A. Hamilton, AUMNH]; **Gonzales**: 6.1 miles SE of Gonzales, 29.497194 -97.380048 ^5^, 328ft., [APH_2658, 18/6/1956, 1♂, W. McAlister, AMNH]; **Harris**: Houston, 29.760193 -95.36939 ^6^, 43ft., [APH_2650, 6/1959, 1♂, Mrs. Emilie Steude, AMNH]; **Jim Hogg**: 0.9 miles E Zapata County Line on FM-2687, 26.939665 -98.941918 ^1^, 526ft., [APH_1131, 15/3/2010, 1 juv, Brent E. Hendrixson, Gerri Wilson, Thomas Martin, AUMNH]; 2.0 miles N Starr County Line on FM-649, 26.81392 -98.855998 ^1^, 625ft., [APH_1130, 15/3/2010, 1 juv, Brent E. Hendrixson, Gerri Wilson, Thomas Martin, AUMNH]; **Karnes**: Karnes City, 28.885483 -97.902683 ^1^, 411ft., [APH_0864-0866, 9/2008, 2♀, 1 juv, Chris A. Hamilton, AUMNH]; [APH_0952, 9/2008, 1♂, Chris A. Hamilton, AUMNH]; **Kleberg**: Kingsville, S Brahma Blvd, 27.4806 -97.855767 ^1^, 77ft., [APH_0856-0857, 9/2008, 2♀, Chris A. Hamilton, AUMNH]; Kingsville, 27.515869 -97.856109 ^5^, 56ft., [APH_2046, 27/6/1970, 1♂, Gillaspy, AMNH]; Kingsville, field at high school, along Caesar Ave across street from cemetery, 27.506531 -97.879785 ^1^, 65ft., [APH_0579-0583, 13/6/2009, 4 juv, Brent E. Hendrixson, Courtney Dugas, Sloan Click, AUMNH]; **La Salle**: Cotulla, 28.436934 -99.235032 ^1^, 437ft., [APH_3129, 2013, 1♂, Roger Birkhead, AUMNH]; Los Angeles, 28.460017 -98.9999 ^1^, 391ft., [APH_0871, 2008, 1♀, Sky Stevens, AUMNH]; **Maverick**: 4.2 miles SW Zavala County Line on Hwy-57, 28.88947 -100.16539 ^2^, 711ft., [APH_1458, 24/1/2012, 1 juv, Stanley A. Schultz, AUMNH]; 4.6 miles E/SE Eagle Pass (jct US-57) on US-277, 28.69567 -100.39303 ^1^, 833ft., [APH_0055, 17/7/2006, 1♂, Brent E. Hendrixson, AUMNH]; [APH_0058, 17/7/2006, 1♀, Brent E. Hendrixson, AUMNH]; [APH_0084, 17/7/2006, 1♀, Brent E. Hendrixson, AUMNH]; 9.0 miles NE US-57 on FM-481, 28.928198 -100.262798 ^1^, 787ft., [APH_1149-1150, 17/3/2010, 2 juv, Brent E. Hendrixson, Gerri Wilson, Thomas Martin, AUMNH]; **McMullen**: 1.25 miles N FM-3445 on TX-16, 28.520536 -98.547662 ^1^, 300ft., [APH_1117, 14/3/2010, 1 juv, Brent E. Hendrixson, Gerri Wilson, Thomas Martin, AUMNH]; **Nueces**: Corpus Christi, Meldo Park, approx. 2 blocks SW Santa Fe St on Brawner Parkway, 27.747437 -97.379802 ^1^, 41ft., [APH_0577-0578, 13/6/2009, 2 juv, Brent E. Hendrixson, Courtney Dugas, Sloan Click, AUMNH]; **Starr**: San Carlos, Hwy 649 ~ 2 miles N of 2686 junction, 26.720783 -98.8468 ^1^, 411ft., [APH_0889, 18/4/2008, 1♀, Christian Cox, Corey Roelke, AUMNH]; **Washington**: 2301 Running Valley Lane, Washington, 30.225692 -96.160138 ^1^, 224ft., [APH_3123, 13/6/2013, 1♂, Chuck Matula, AUMNH]; 5 miles west of Carmine, 30.140828 -96.759342 ^5^, 433ft., [APH_2660, 27/6/1960, 2♀, W. McAlister, AMNH]; Brenham, 30.145467 -96.43435 ^5^, 323ft., [APH_0968, 6/2006, 1♂, Dave Moellendorf, AUMNH]; Chappell Hill, 1/4 mile S of intersection of Old Chappell Hill Rd and Pulaski School Rd, 30.1577 -96.288344 ^1^, 282ft., [APH_3121, 20/5/2013, 1♂, Todd Burch, AUMNH]; [APH_3122, 30/5/2013, 1♂, Todd Burch, AUMNH]; **Webb**: 0.8 miles NW Hwy-44 on US-83, 28.03711 -99.54681 ^1^, 820ft., [APH_1285, 13/5/2011, 1♂, Brent E. Hendrixson, Kate Hall, Austin Deskewies, Alexis Guice, AUMNH]; 1.1 miles SW Duval County Line on US-59, 27.786023 -98.818769 ^1^, 451ft., [APH_0524-0528, 3/6/09, 3♀, 2 juv, Brent E. Hendrixson, Courtney Dugas, Sloan Click, AUMNH]; 3.2 miles W FM-2895 on TX-359, 27.454855 -99.136257 ^1^, 511ft., [APH_1125, 15/3/2010, 1 juv, Brent E. Hendrixson, Gerri Wilson, Thomas Martin, AUMNH]; 3.6 miles NW I-35 on US-83, 27.80051 -99.46291 ^1^, 760ft., [APH_1281-1282, 13/5/2011, 2 juv, Brent E. Hendrixson, Kate Hall, Austin Deskewies, Alexis Guice, AUMNH]; 4.3 miles E Loop-20 on US-59 at Los Tios Creek, 27.56159 -99.39045 ^1^, 470ft., [APH_1280, 13/5/2011, 1 juv, Brent E. Hendrixson, Kate Hall, Austin Deskewies, Alexis Guice, AUMNH]; 5.1 miles SE La Salle County Line on Hwy-44, 28.012456 -99.195109 ^1^, 454ft., [APH_0530-0531, 3/6/2009, 2 juv, Brent E. Hendrixson, Courtney Dugas, Sloan Click, AUMNH]; **Wharton**: El Campo, 29.19405 -96.2617 ^1^, 90ft., [APH_0800-0803, 7/2008, 4♀, Chris A. Hamilton, AUMNH]; **Zapata**: 2.0 miles NW FM-2687 on US-83, 26.80362 -99.142 ^1^, 410ft., [APH_1277-1279, 13/5/2011, 1♂, 2 juv, Brent E. Hendrixson, Kate Hall, Austin Deskewies, Alexis Guice, AUMNH]; **Mexico: Nuevo Leon**: Cañon de la Huasteca, 25.61484 -100.47585 ^1^, 2600ft., [APH_0056, 24/7/2006, 1♀, Brent E. Hendrixson, AUMNH].

#### Distribution and natural history.

In the United States, *Aphonopelma anax* is widely distributed throughout South Texas (Fig. 14). We only sampled a single individual from western Nuevo Leon but the species distribution model predicts suitable habitat for this species throughout northeastern Mexico in Coahuila, Nuevo Leon, and Tamaulipas. The vast majority of specimens were collected at elevations between sea level and 300 meters along the Western Gulf Coastal Plain (Fig. 1I) or Southern Texas Plains Level III Ecoregions, but other samples were obtained from the Texas Blackland Prairies, East Central Texas Plains, South Central Plains, and the very southern edge of the Edwards Plateau. The specimen from Nuevo Leon (at Huasteca Canyon near Monterrey) was collected at an elevation of approximately 850 meters. This species has been observed in syntopy with *Aphonopelma moderatum* (burrows located fewer than 50 centimeters from each other) at several different locations near the Rio Grande from Eagle Pass (Maverick County) to Rio Grande City (Starr County). *Aphonopelma anax* may also be found alongside *Aphonopelma armada* and *Aphonopelma hentzi*. Burrows are typical of that for North American tarantulas (i.e., circular and generally covered by a thin veil of silk) and specimens can be readily collected by pouring a small amount of water into their burrows. In extreme South Texas, *Aphonopelma anax* is probably active year-round; however, in more northern populations, spiders likely plug their burrows for short periods of time during the winter. The breeding period appears to be restricted to spring and early summer (April-July); adult males have been observed in large numbers at night along roads following heavy bouts of rain (United States Border Patrol 2009, pers. comm.).

#### Conservation status.

*Aphonopelma anax* is very common throughout its distribution. Extensive fieldwork near Edinburg and McAllen (Hidalgo County) suggests that some local populations of *Aphonopelma anax* have probably been extirpated due to extensive agriculture in the Lower Rio Grande Valley, but overall the species is fairly abundant throughout South Texas. These spiders appear to thrive in a variety of anthropogenic settings including golf courses, residential lawns, city parks, roadside picnic areas, and mowed highway shoulders. The status of *Aphonopelma anax* is likely secure.

#### Remarks.

*Aphonopelma anax* is one of the largest and most robust *Aphonopelma* within the United States. This species exhibits size variation within males and females across their distribution, with northern populations generally smaller and the southern populations representing the largest tarantulas in the United States. Other important ratios that distinguish males: *Aphonopelma anax* possess a smaller M1/F4 (≤0.75; 0.69-0.75) than *Aphonopelma moderatum* (≥0.80; 0.80-0.88) and *Aphonopelma moellendorfi* (≥0.81; 0.81-0.88). No other ratios distinguish female *Aphonopelma anax* from their syntopic or closely related phylogenetic species. For both males and females, certain morphometrics have potential to be useful though due to the amounts of variation, small number of specimens, and the small differences between species none are claimed to be significant at this time (see Suppl. material 2). During evaluation of traditional PCA morphospace, males of *Aphonopelma anax* separate from *Aphonopelma moderatum*, *Aphonopelma moellendorfi*, and *Aphonopelma gabeli* along PC1~2, but do not separate from *Aphonopelma armada* or *Aphonopelma hentzi*. Females separate from *moderatum* along PC1~2, but do not separate from *Aphonopelma armada*, *Aphonopelma gabeli*, *Aphonopelma hentzi*. Females of *Aphonopelma moellendorfi* are unknown at this time and cannot be compared. Interestingly, *Aphonopelma anax* males separate from *Aphonopelma gabeli*, *Aphonopelma moderatum*, and *Aphonopelma moellendorfi* in three-dimensional PCA morphospace (PC1~PC2~PC3), but do not separate from *Aphonopelma armada* and *Aphonopelma hentzi*. *Aphonopelma anax* females separate from *Aphonopelma armada* and *Aphonopelma moderatum*, but do not separate from *Aphonopelma gabeli* and *Aphonopelma hentzi*. PC1, PC2, and PC3 explain ≥87% of the variation in male analyses and ≥96% of the variation in female analyses.

It is important to note the tremendous amount of variation that can be observed in the shape of the spermathecae from numerous populations of *Aphonopelma anax* (Figs 12–13). Previous taxonomic work considered this amount of variation to represent differences between species (e.g., Smith 1995 differentiated his new species *Aphonopelma breenei* from *Aphonopelma anax* on the basis of its spermathecae) (Smith 1995) but our samples demonstrate that the shape of spermathecae is quite variable and is not useful for differentiating these two species. The spermathecae are however useful for distinguishing *Aphonopelma anax* from other members of the *Hentzi* species complex (*Aphonopelma armada* and *Aphonopelma hentzi*). This information was used to place *Aphonopelma harlingenum* (whose type locality is surrounded by *Aphonopelma anax*) into synonymy with *Aphonopelma hentzi* and not *Aphonopelma anax*. It is also important to note that the paratype of *Aphonopelma harlingenum* that is found in the same jar as the *Aphonopelma harlingenum* holotype is a female of *Aphonopelma anax* (see spermathecae in Fig. 12C). Additionally, as seen in the spermathecae, we find that palpal bulb variation is extreme both across and within *Aphonopelma* species - an unfortunate finding that further enforces our idea that these traditionally used morphological characters are ineffective species delimiters. Unless these characters are extreme autapomorphies (i.e. *Aphonopelma anax* spermathcae and palpal bulbs, *Aphonopelma gabeli* spermathecae) that can be used in conjunction with other evidence to determine species boundaries in this group of spider, they should not be regarded as taxonomically informative.

Mitochondrial DNA (*CO1*) identifies *Aphonopelma anax* as a polyphyletic group with respect to *Aphonopelma armada* and *Aphonopelma hentzi* (Fig. 7). A second lineage of *Aphonopelma anax* is sister to a lineage of *Aphonopelma hentzi*; both of these lineages were previously identified as putative cryptic species (Hamilton et al. 2014). The AE data demonstrate that *CO1* is not effective at accurately delimiting species boundaries within this group. Finally, we examined the holotype and freshly collected topotypic material of *Aphonopelma breenei*. Our morphological and molecular analyses fail to recognize this species as a separate, independently evolving lineage. As a consequence, we consider *Aphonopelma breenei* a junior synonym of *Aphonopelma anax*.

### Aphonopelma armada

Taxon classificationAnimaliaAraneaeTheraphosidae

(Chamberlin, 1940)

**Figure 15.**
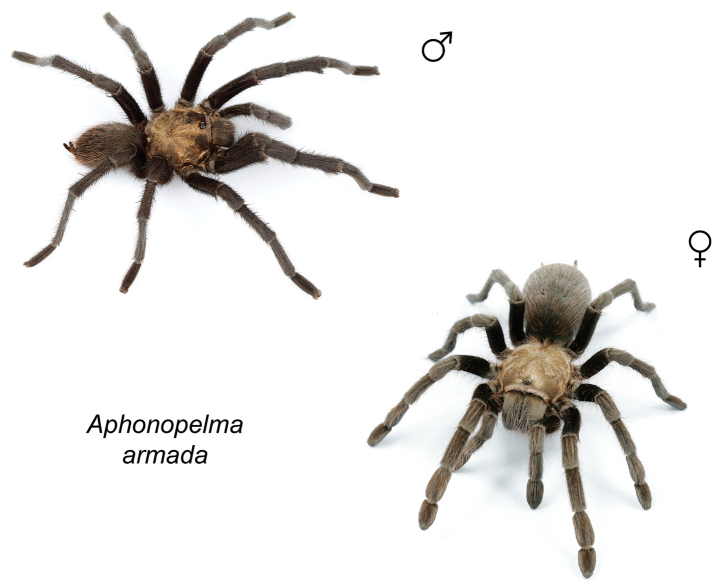
*Aphonopelma armada* (Chamberlin, 1940) specimens, live photographs. Male (L) - APH_1064; Female (R) - APH_3214.

**Figure 16.**
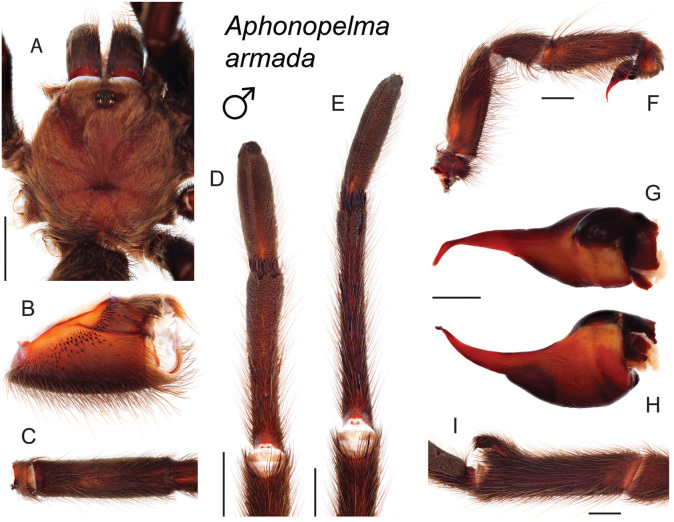
*Aphonopelma armada* (Chamberlin, 1940). **A–I** male specimen, APH_0950 **A** dorsal view of carapace, scale bar = 6.5mm **B** prolateral view of coxa I **C** dorsal view of femur III **D** ventral view of metatarsus III, scale bar = 4mm **E** ventral view of metatarsus IV, scale bar = 3mm **F** prolateral view of L pedipalp and palpal tibia, scale bar = 3mm **G** dorsal view of palpal bulb **H** retrolateral view of palpal bulb, scale bar = 1mm **I** prolateral view of tibia I (mating clasper), scale bar = 2mm.

**Figure 17.**
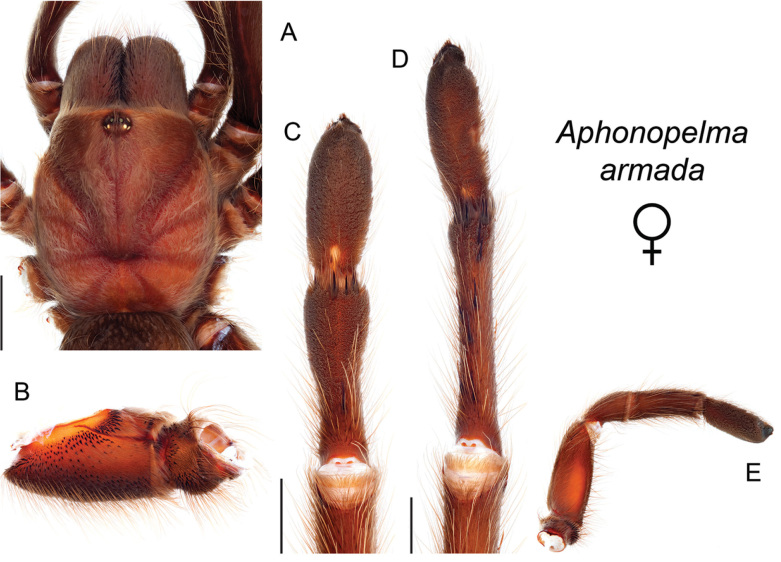
*Aphonopelma armada* (Chamberlin, 1940). **A–E** female specimen, APH_0848 **A** dorsal view of carapace, scale bar = 6mm **B** prolateral view of coxa I **C** ventral view of metatarsus III, scale bar = 3mm **D** ventral view of metatarsus IV, scale bar = 3mm **E** prolateral view of L pedipalp and palpal tibia.

**Figure 18.**
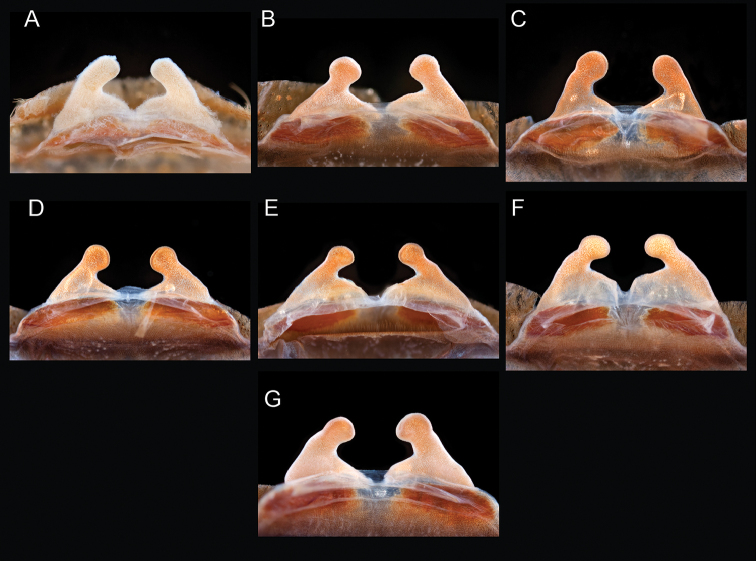
*Aphonopelma armada* (Chamberlin, 1940). **A–G** cleared spermathecae **A** *armada* holotype **B** APH_0547 **C** APH_0548 **D** APH_0807 **E** APH_0848 **F** APH_1049 **G** APH_1068.

**Figure 19.**
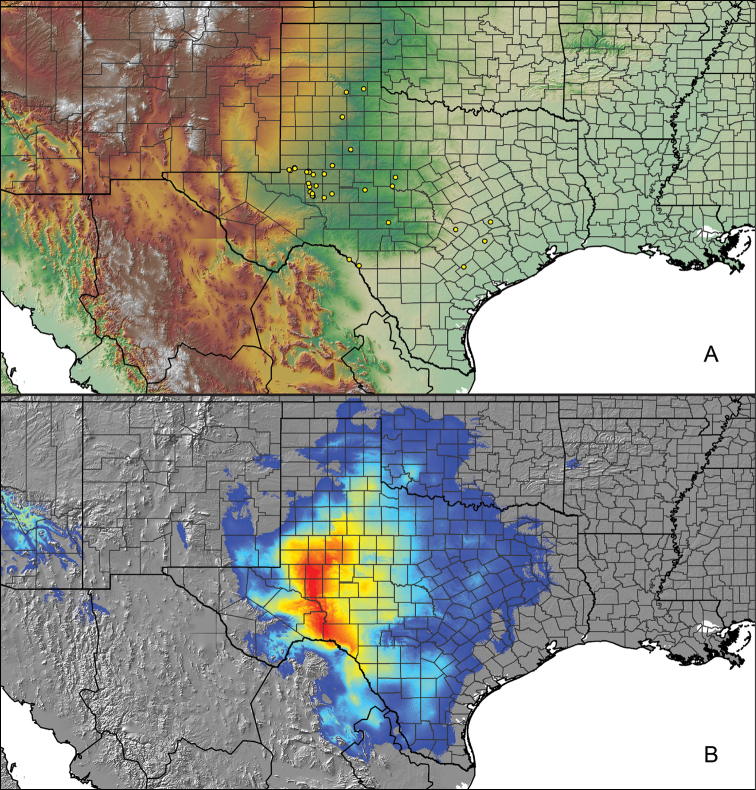
*Aphonopelma armada* (Chamberlin, 1940). **A** distribution of known specimens **B** predicted distribution; warmer colors (red, orange, yellow) represent areas of high probability of occurrence, cooler colors (blue shades) represent areas of low probability of occurrence.

Figures 15 , 16 , 17 , 18 , 19

Dugesiella armada Chamberlin, 1940: 32; female holotype from Austin, Travis Co., Texas, 30.267153 -97.743061^6^, elev. 461ft., ix.1909, coll. A. Petrunkevitch; deposited in AMNH. [examined]
Rhechostica armada Raven, 1985: 149.
Aphonopelma armada Smith, 1995: 71.
Aphonopelma arnoldi Smith, 1995: 74; male holotype from Hwy 82 near Crosbyton, Crosby Co., Texas, 33.660017 -101.294644^5^, elev. 3063ft., 17.vi.1963, coll. P. Keathley; deposited in Oklahoma State University collection. [not examined] **syn. n.**

#### Diagnosis.

*Aphonopelma armada* (Fig. 15) is a member of the *Hentzi* species group and can be identified by a combination of morphological, molecular, and geographic characteristics. Nuclear and mitochondrial DNA identifies *Aphonopelma armada* as a phylogenetically distinct monophyletic lineage (Figs 7–8), supported as a sister lineage to *Aphonopelma anax* and *Aphonopelma hentzi*. Female *Aphonopelma armada* can be distinguished by their noticeably large urticating hair patch (black spot), a less hirsute and more lustrous appearance, and metatarsi I, II, and III that are distinctly flared and wider than other syntopic species (Fig. 15). Male *Aphonopelma armada* are generally smaller and less hirsute than other similar looking syntopic species. Setae on the prolateral face of coxa I have a unique pattern that help distinguish *Aphonopelma armada* from all other *Aphonopelma* in the United States (Figs 16–17). Significant measurements that distinguish male *Aphonopelma armada* from its closely related phylogenetic and syntopic species are F4, PTl, and extent of metatarsus III scopulation. Male *Aphonopelma armada* can be distinguished by possessing a larger PTl/M1 (≥0.73; 0.73–0.83) than *Aphonopelma gabeli* (≤0.68; 0.61–0.68), *Aphonopelma moderatum* (≤0.69; 0.61–0.69), and *Aphonopelma moellendorfi* sp. n. (≤0.69; 0.60–0.69); by possessing a smaller F4/M4 (≤0.92; 0.86–0.92) than *Aphonopelma anax* (≥0.94; 0.94–1.04); and smaller L3 scopulation extent (48%–63%) than *Aphonopelma hentzi* (69%–86%). Significant measurements that distinguish female *Aphonopelma armada* from its closely related phylogenetic and syntopic species are P1, Cl, and extent of metatarsus IV scopulation. Female *Aphonopelma armada* can be distinguished by possessing a larger Cl/A3 (≥2.48; 2.48–2.69) than *Aphonopelma moderatum* (≤2.46; 2.25–2.46); a larger P1/F4 (≥0.47; 0.47–0.52) than *Aphonopelma gabeli* (≤0.46; 0.42–0.46); by possessing a smaller P1/T3 (≤13.84; 9.93–13.84) than *Aphonopelma anax* (≥13.88; 13.88–19.15); and a smaller L4 scopulation extent (28%-43%) than *Aphonopelma hentzi* (42%-72%, with slight overlap). Females of *Aphonopelma moellendorfi* are unknown and cannot be compared.

#### Description.

Female originally described by Chamberlin (1940).

#### Description of male exemplar

(APH_0950; Fig. 16). *Specimen preparation and condition*: Specimen collected live crossing road, preserved in 80% ethanol; deposited in AUMNH; original coloration faded due to preservation. Left legs I, III, IV, and left pedipalp removed for measurements and photographs; stored in vial with specimen. Right leg III removed for DNA and stored at -80°C in the AUMNH (Auburn, AL). *General coloration*: Generally black or faded to brown. *Cephalothorax*: Carapace 14.59 mm long, 13.42 mm wide; Very hirsute, densely clothed with brown/golden iridescent pubescence appressed to surface; fringe covered in long setae not closely appressed to surface; foveal groove medium deep and straight; pars cephalica region rises gradually from foveal groove, gently arching anteriorly toward ocular area; AER slightly procurved, PER slightly recurved; normal sized chelicerae; clypeus extends forward on a slight curve; LBl 2.09, LBw 2.42; sternum hirsute, clothed with medium length black/brown, densely packed setae. *Abdomen*: Densely clothed in short black/brown pubescence with numerous longer, lighter setae interspersed (generally red or orange *in situ*); possessing a dense dorsal patch of black Type I urticating bristles (Cooke et al. 1972). *Legs*: Hirsute, densely clothed in a mix of short black/brown pubescence with longer ventral setae. Metatarsus I slightly curved. F1 15.16; F1w 3.72; P1 6.29; T1 11.1; M1 10.31; A1 7.88; F3 12.04; F3w 3.49; P3 5.26; T3 8.89; M3 11.36; A3 7.68; F4 14.12; F4w 3.58; P4 5.76; T4 13.16; M4 15.28; A4 8.03; femur III is normal - not noticeably swollen or wider than other legs. All tarsi fully scopulate. Extent of metatarsal scopulation: leg III (SC3) = 63.4%; leg IV (SC4) = 37.1%. Three ventral spinose setae on metatarsus III; six ventral spinose setae on metatarsus IV. *Coxa I*: Prolateral surface a mix of fine, hair-like, very thick tapered, and stout setae with a unique placement around the anterior, prolateral margin. *Pedipalps*: Hirsute; densely clothed in the same setal color as the other legs, with numerous longer ventral setae; two spinose setae on the prolateral tibia; PTl 8.554, PTw 2.668. When extended, embolus tapers with a gentle curve to the retrolateral side near apex; embolus very slender, no keels; distinct ventral shift from the bulb to the embolus.

**Variation (5).** Cl 13.19–16.38 (14.614±0.61), Cw 11.37–13.88 (12.912±0.48), LBl 1.79–2.09 (1.956±0.06), LBw 2.04–2.51 (2.28±0.09), F1 13.7–15.67 (14.656±0.4), F1w 3.27–3.97 (3.688±0.13), P1 4.61–6.91 (6.056±0.38), T1 11.1–12.48 (11.724±0.23), M1 9.28–11.36 (10.386±0.39), A1 7.13–8.51 (7.76±0.25), L1 length 46.14–53.64 (50.582±1.41), F3 10.81–12.46 (11.742±0.31), F3w 3.18–3.97 (3.546±0.14), P3 4.99–5.48 (5.236±0.08), T3 8.26–9.35 (8.806±0.22), M3 10.78–12.26 (11.414±0.29), A3 6.68–7.72 (7.25±0.21), L3 length 41.97–46.59 (44.448±1.02), F4 12.85–14.12 (13.4±0.24), F4w 3.17–3.66 (3.474±0.09), P4 4.73–5.96 (5.476±0.22), T4 10.95–13.16 (11.896±0.36), M4 14.18–15.5 (14.95±0.23), A4 7.37–8.77 (8.15±0.24), L4 length 51.3–56.35 (53.872±1.03), PTl 7.053–8.554 (8.061±0.28), PTw 2.372–2.756 (2.578±0.07), SC3 ratio 0.483–0.634 (0.566±0.03), SC4 ratio 0.292–0.371 (0.328±0.01), Coxa I setae = very thick tapered & stout, F3 condition = normal.

#### Redescription of female exemplar

(APH_0848; Figs 17–18). *Specimen preparation and condition*: Specimen collected live from burrow, preserved in 80% ethanol; deposited in AUMNH; original coloration faded due to preservation. Left legs I, III, IV, and pedipalp removed for photographs and measurements; stored in vial with specimen. Right leg III removed for DNA and stored at -80°C in the AUMNH (Auburn, AL). Genital plate with spermathecae removed and cleared, stored in vial with specimen. *General coloration*: Faded black and brown, medium length setae cover body. *Cephalothorax*: Carapace 16.71 mm long, 14.08 mm wide; densely clothed with light brown pubescence closely appressed to surface; fringe densely covered in medium setae; foveal groove medium deep and slightly procurved; pars cephalica region rises from thoracic furrow more steeply than male, gently arching anteriorly toward ocular area; AER slightly procurved, PER recurved; clypeus mostly straight; LBl 2.4, LBw 2.4; sternum hirsute, clothed with brown, medium length and shorter, dense setae. *Abdomen*: Densely clothed dorsally in short black/brown setae with numerous longer, lighter setae interspersed (generally red or orange *in situ*); dense dorsal patch of black Type I urticating bristles (Cooke et al. 1972); ventral side with shorter brown setae. *Spermathecae*: Paired and separate, tapering to capitate bulbs, possessing a secondary bulge near the interior of the base, with wide bases that are not fused. *Legs*: Hirsute, particularly ventrally; densely clothed in a mix of short, brown pubescence with longer setae interspersed. F1 12.9; F1w 4.36; P1 6.08; T1 9.69; M1 7.27; A1 5.97; F3 10.68; F3w 3.65; P3 5.22; T3 6.69; M3 7.79; A3 6.21; F4 12.46; F4w 4.03; P4 5.82; T4 10.34; M4 10.63; A4 7.12. All tarsi fully scopulate. Extent of metatarsal scopulation: leg III (SC3) = 60.5%; leg IV (SC4) = 28.2%. Two ventral spinose setae on metatarsus III; seven ventral spinose setae on metatarsus IV. *Coxa I*: Prolateral surface a mix of fine, hair-like, very thick tapered, and stout setae with a unique placement around the anterior and ventral, prolateral margin. *Pedipalps*: Densely clothed in the same setal color as the other legs; one spinose seta on the apical, prolateral femur and five spinose setae on the prolateral tibia.

**Variation (6).** Cl 11.96–16.76 (14.852±0.78), Cw 9.64–14.64 (12.702±0.78), LBl 1.7–2.4 (2.107±0.11), LBw 1.93–2.97 (2.45±0.14), F1 9.847–13.69 (11.846±0.56), F1w 3.31–4.45 (3.908±0.19), P1 4.427–6.51 (5.481±0.33), T1 7.642–10.33 (9.015±0.4), M1 5.741–7.44 (6.824±0.27), A1 4.659–6.84 (5.905±0.3), L1 length 32.316–44.74 (39.071±1.79), F3 7.03–11.18 (9.385±0.63), F3w 2.62–3.72 (3.192±0.19), P3 3.31–5.63 (4.588±0.33), T3 5.51–7.33 (6.422±0.27), M3 5.66–8.73 (7.21±0.41), A3 4.81–6.5 (5.732±0.26), L3 length 26.32–39.37 (33.337±1.84), F4 9.11–12.55 (11.117±0.56), F4w 2.72–4.03 (3.41±0.21), P4 3.85–5.82 (4.963±0.3), T4 7.67–10.35 (9.172±0.43), M4 7.81–11.74 (10.153±0.58), A4 5.58–7.59 (6.528±0.32), L4 length 34.02–47.93 (41.933±2.09), SC3 ratio 0.566–0.737 (0.629±0.02), SC4 ratio 0.282–0.429 (0.356±0.03), Coxa 1 setae = very thick tapered & stout. Spermathecae variation can be seen in Figure 18.

#### Material examined.

**United States: Texas: Andrews**: SW4001 and SW7000, 32.131369 -102.621689 ^1^, 3152ft., [APH_1049, 6/7/2010, 1♀, Skyler Stevens, AUMNH]; SW4001, 32.113981 -102.615814 ^1^, 3140ft., [APH_1052, 6/7/2010, 1♂, Skyler Stevens, AUMNH]; **Briscoe**: Caprock Canyons State Park, Honey Flat camping area (site 4), 34.419514 -101.056081 ^1^, 2611ft., [APH_0551-0554, 7/6/2009, 4 juv, Brent E. Hendrixson, Courtney Dugas, Sloan Click, AUMNH]; **Burleson**: Caldwell, 30.49425 -96.6921 ^5^, 373ft., [APH_0944, 7/2008, 1♂, Dave Moellendorf, AUMNH]; **Coleman**: O.H. Ivie reservoir, Coleman Lake at Hords Creek, 31.842967 -99.5696 ^1^, 1936ft., [APH_0840, 9/2008, 1♀, Chris A. Hamilton, AUMNH]; O.H. Ivie reservoir, 31.57695 -99.66065 ^1^, 1571ft., [APH_0950, 9/2008, 1♂, Chris A. Hamilton, AUMNH]; **Crosby**: 0.2 miles N US-82 on FM 2591 (E of Crosbyton), 33.669122 -101.175656 ^1^, 2802ft., [APH_0547-0550, 7/6/2009, 4 juv, Brent E. Hendrixson, Courtney Dugas, Sloan Click, AUMNH]; **DeWitt**: Westhoff, Meyer Rd off Hwy 240 and County Rd 142, 29.136983 -97.497033 ^1^, 342ft., [APH_0922-0923, 9/2008, 2♂, Dan Lewis, AUMNH]; **Ector**: Cowden H Ranch, 32.076633 -102.789983 ^5^, 3320ft., [APH_0855, 2006, 1♀, Dave Moellendorf, AUMNH]; [APH_0965-0967, 2006, 3♂, Dave Moellendorf, AUMNH]; [APH_0977, 2006, 1♀, Dave Moellendorf, AUMNH]; **Fayette**: La Grange, 29.914433 -96.866317 ^1^, 321ft., [APH_0807, 5/2008, 1♀, Chris A. Hamilton, AUMNH]; **Glasscock**: E of Midland, off Hwy 137, 31.94904 -101.72346 ^2^, 2569ft., [APH_1468, 19/6/2012, 1♂, Darryl Burton, AUMNH]; **Hall**: Hulver Cemetery, 5.9 miles W US-287 on Hwy-86, 34.522756 -100.534382 ^2^, 1955ft., [APH_1460, 27/5/2012, 1♀, Brent E. Hendrixson, AUMNH]; **Howard**: Big Spring, at miniature golf course, 32.200567 -101.47715 ^1^, 2701ft., [APH_0841-0844, 9/2008, 4♀, Chris A. Hamilton, AUMNH]; [APH_0945, 9/2008, 1♀, Chris A. Hamilton, AUMNH]; **Kimble**: Texas Tech Field Station, Junction, 30.472222 -99.780833 ^1^, 1718ft., [APH_1067, 24/6/2010, 1♂, Skyler Stevens, AUMNH]; [APH_1068, 16/6/2010, 1♀, Bryce Hubbell, AUMNH]; [APH_1069, 17/6/2010, 1♀, Travis Fisher, AUMNH]; **Kinney**: 0.59 miles E US-277 on FM-693, 29.17044 -100.673259 ^1^, 972ft., [APH_1164, 17/3/2010, 4 juv, Brent E. Hendrixson, Gerri Wilson, Thomas Martin, AUMNH]; **Midland**: Midland, 31.924217 -102.0583 ^1^, 2784ft., [APH_0953, 9/2008, 1♀, Chris A. Hamilton, AUMNH]; W County Rd 54, 32.027933 -102.206853 ^1^, 2883ft., [APH_1056-1059, 5/7/2010, 4♂, Skyler Stevens, AUMNH]; CR60, 32.010556 -102.2275 ^1^, 2888ft., [APH_1061-1062, 2/7/2010, 2♂, Skyler Stevens, AUMNH]; near Midland, along Hwy-158, 31.999899 -102.180216 ^1^, 2862ft., [APH_1172-1173, 21/7/2010, 1♀, 1♂, Brent E. Hendrixson, Brendon Barnes, Nate Davis, AUMNH]; near Midland, along CR-60, 32.003836 -102.256184 ^1^, 2890ft., [APH_1174, 21/7/2010, 1♂, Brent E. Hendrixson, Brendon Barnes, Nate Davis, AUMNH]; 0.5 miles S CR 118W on Hwy-349, 31.58789 -101.9704 ^2^, 2793ft., [APH_1462, 13/6/2012, 1♂, Darryl Burton, AUMNH]; 0.3 miles S FM-1787 on FM-1492, 31.66265 -102.20689 ^2^, 2876ft., [APH_1466, 17/6/2012, 1♂, Darryl Burton, AUMNH]; **Reagan**: Hwy 137, 31.343611 -101.495833 ^1^, 2617ft., [APH_1064-1065, 30/6/2010, 2♂, Skyler Stevens, AUMNH]; off Hwy 67, 31.23071389 -101.7264444 ^2^, 2715ft., [APH_1385, 19/9/2011, 1 juv, Darryl Burton, AUMNH]; **Scurry**: Snyder, 32.682133 -100.925483 ^1^, 2353ft., [APH_0845-0849, 9/2008, 5♀, Chris A. Hamilton, AUMNH]; **Tom Green**: San Angelo, approx. 1 miles N of W Hwy-67, 31.46431 -100.49511 ^4^, 1922ft., [APH_0010, 23/6/2005, 1♂, Kati and Martha Mayfield, AUMNH]; **Upton**: oil fields W of Hwy 329, 31.28048056 -102.0802 ^2^, 2605ft., [APH_1374, 17/6/2011, 1♀, Darryl Burton, AUMNH]; [APH_1375, 16/6/2011, 1 juv, Darryl Burton, AUMNH]; [APH_1377, 19/7/2011, 1 juv, Darryl Burton, AUMNH]; [APH_1379, 6/9/2011, 1 juv, Darryl Burton, AUMNH]; [APH_1381, 10/9/2011, 1♀, Darryl Burton, AUMNH]; FM 1492, 31.56408333 -102.1742556 ^2^, 2855ft., [APH_1384, 11/9/2011, 1♀, Darryl Burton, AUMNH]; [APH_1387, 31/8/2011, 1 juv, Darryl Burton, AUMNH]; [APH_1388, 11/9/2011, 1 juv, Darryl Burton, AUMNH]; oil fields E of Hwy 329, 31.35340556 -102.0877333 ^2^, 2695ft., [APH_1389, 30/8/2011, 1♀, Darryl Burton, AUMNH]; FM 1492, 31.56408333 -102.1742556 ^2^, 2855ft., [APH_1391, 23/8/2011, 1♀, Darryl Burton, AUMNH]; **Val Verde**: Del Rio, near River Rd, 29.35553 -100.972297 ^5^, 885ft., [APH_0593-0594, Spring 2009, 2 juv, unknown, AUMNH]; 2.2 miles off Hwy 90 on spur 406, N of Del Rio, 29.574484, -101.041898 ^1^, 1195ft., [APH_3124, 15/6/2014, 1♀, Dave Moellendorf, AUMNH].

#### Distribution and natural history.

*Aphonopelma armada* has a wide distribution across Texas and can be found inhabiting these Level III Ecoregions: Chihuahuan Deserts, High Plains, Southwestern Tablelands, Central Great Plains, Edwards Plateau, Southern Texas Plains, Texas Blackland Prairies, and East Central Texas Plains (Fig. 19). *Aphonopelma armada* can be found in syntopy with a number of other species across its distribution: *Aphonopelma anax*, *Aphonopelma gabeli*, *Aphonopelma hentzi*, *Aphonopelma moderatum*, and *Aphonopelma moellendorfi*. Mating season, when mature males emerge, is similar to the other syntopic species (i.e., males are more active during the evenings and early mornings from late spring to early summer).

#### Conservation status.

*Aphonopelma armada* is very common throughout its distribution in South and West Texas. The species is likely secure.

#### Remarks.

The type locality for *Aphonopelma armada* is vague (Austin, Texas) and despite much fieldwork in the region, we have never been able to find specimens referable to *Aphonopelma armada* in or around the Austin city limits (this area is dominated by *Aphonopelma hentzi*). We have, however, found the species in counties to the south and east of Austin and note that it has a crescent-like distribution around Austin and becomes more common to the west (Fig. 19). Other important ratios that distinguish males: *Aphonopelma armada* possess a larger CL/M1 (≥1.36; 1.36–1.47) than *Aphonopelma moderatum* (≤1.30; 1.13–1.30), and *Aphonopelma moellendorfi* (≤1.31; 1.10–1.31); as well as possessing a smaller F4/M4 (≤0.92; 0.86–0.92) than *Aphonopelma hentzi* (≥0.92; 0.92–1.03). Other important ratios distinguish females: *Aphonopelma armada* possess a smaller T3/T4 (≤0.73; 0.64–0.73) than *Aphonopelma gabeli* (≥0.73; 0.73–0.78); as well as possessing a smaller L4/Cl (≤2.89; 2.73–2.89) than *Aphonopelma moderatum* (≥2.89; 2.89–3.09). Due to the amounts of variation, small number of specimens, and/or the small differences between species no others are claimed to be significant at this time (see Suppl. material 2). During evaluation of traditional PCA morphospace, males of *Aphonopelma armada* separate from *Aphonopelma gabeli*, *Aphonopelma moderatum*, *Aphonopelma moellendorfi* along PC1~2, but do not separate from *Aphonopelma hentzi* or *Aphonopelma anax*. Females do not separate from *Aphonopelma anax*, *Aphonopelma gabeli*, *Aphonopelma hentzi*, and *Aphonopelma moderatum*. Females of *Aphonopelma moellendorfi* are unknown and cannot be compared. Interestingly, *Aphonopelma armada* males separate from *Aphonopelma gabeli*, *Aphonopelma moderatum*, and *Aphonopelma moellendorfi* in three-dimensional PCA morphospace (PC1~PC2~PC3), but do not separate from *Aphonopelma anax* and *Aphonopelma hentzi*. *Aphonopelma armada* females separate from *Aphonopelma anax*, but do not separate from *Aphonopelma gabeli*, *Aphonopelma hentzi*, and *Aphonopelma moderatum*. PC1, PC2, and PC3 explain ≥87% of the variation in male analyses and ≥96% of the variation in female analyses.

We did not examine the holotype of *Aphonopelma arnoldi* but we did have the opportunity to study freshly collected topotypic material of the species from Crosbyton, Texas. Our morphological and molecular analyses fail to recognize this species as a separate, independently evolving lineage. As a consequence, we consider *Aphonopelma arnoldi* a junior synonym of *Aphonopelma armada*.

### Aphonopelma atomicum

Taxon classificationAnimaliaAraneaeTheraphosidae

Hamilton sp. n.

http://zoobank.org/A6BA5213-C063-460A-B246-7DBB940A016A

**Figure 20.**
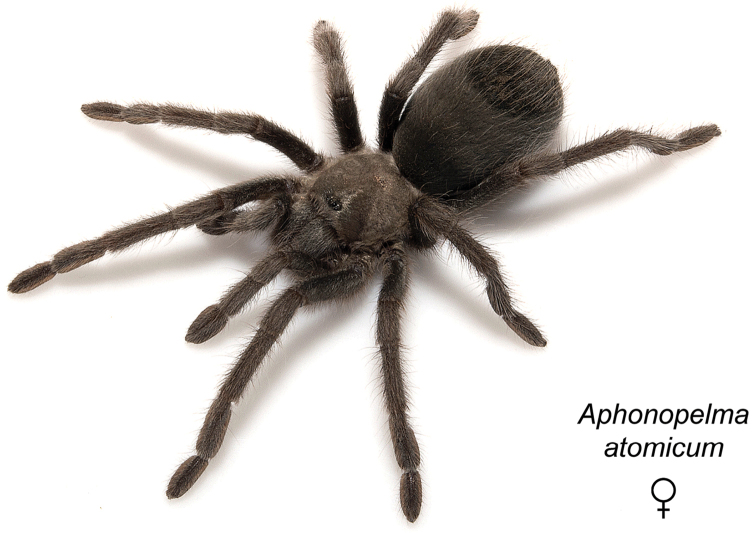
*Aphonopelma atomicum* sp. n. live photograph. Female - APH_1478. Do not have a photograph of a live male specimen.

**Figure 21.**
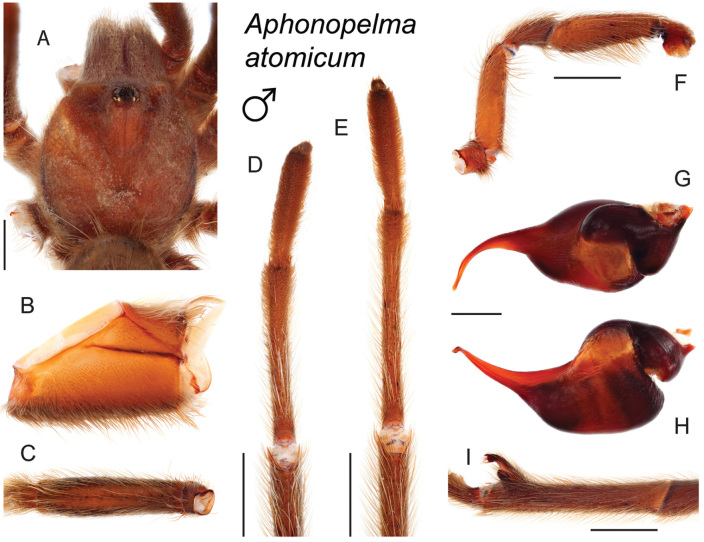
*Aphonopelma atomicum* sp. n. **A–I** male holotype, APH_2727-2 **A** dorsal view of carapace, scale bar = 2mm **B** prolateral view of coxa I **C** dorsal view of femur III **D** ventral view of metatarsus III, scale bar = 3mm **E** ventral view of metatarsus IV, scale bar = 3mm **F** prolateral view of L pedipalp and palpal tibia, scale bar = 3mm **G** dorsal view of palpal bulb **H** retrolateral view of palpal bulb, scale bar = 0.5mm **I** prolateral view of tibia I (mating clasper), scale bar = 2.5mm.

**Figure 22.**
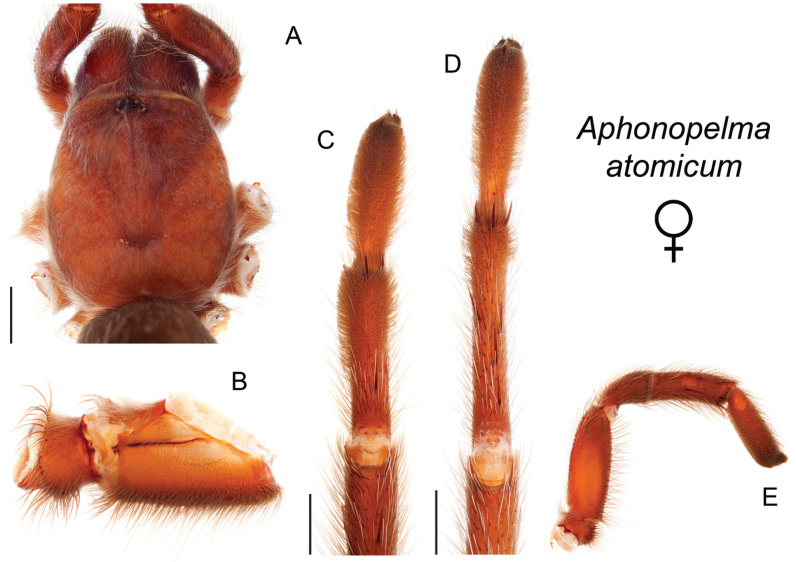
*Aphonopelma atomicum* sp. n. **A–E** female paratype, APH_3267-2 **A** dorsal view of carapace, scale bar = 2mm **B** prolateral view of coxa I **C** ventral view of metatarsus III, scale bar = 1.5mm **D** ventral view of metatarsus IV, scale bar = 1.5mm **E** prolateral view of L pedipalp and palpal tibia.

**Figure 23.**
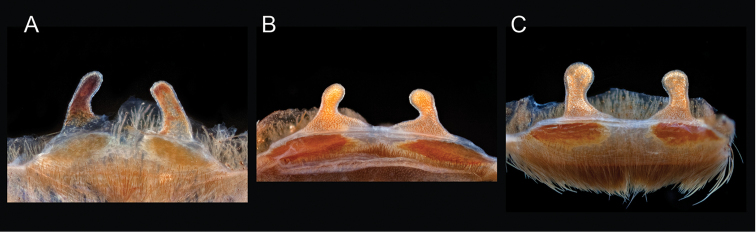
*Aphonopelma atomicum* sp. n. **A–C** cleared spermathecae **A** APH_2727 **B** APH_3267 **C** APH_3267-2.

**Figure 24.**
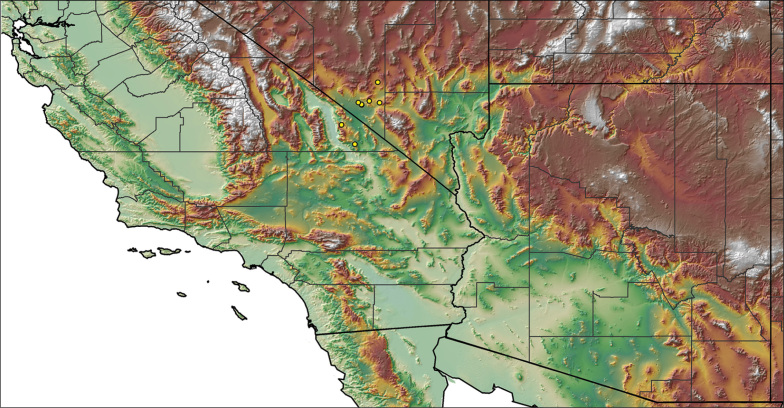
*Aphonopelma atomicum* sp. n. distribution of known specimens. There is no predicted distribution map due to the limited number of sampling localities and restricted distribution this species possesses.

Figures 20 , 21 , 22 , 23 , 24

Aphonopelma mojave Prentice, 1997 (in part): 161.

#### Types.

Male holotype (APH_2727-2) collected at the Nevada Testing Site, Nye Co., Nevada, 37.025579 -116.023865 ^6^, elev. 3990ft., viii.1961, coll. Gertsch; deposited in AMNH. Paratype female (AUMS_3267-2) from 10 miles W of Mercury, Mercury Valley, Nye Co., Nevada, 36.691928 -116.174483 ^5^, elev. 3470ft., 3.xi.1972, coll. Wendell Icenogle; deposited in AUMNH. Paratype male (AUMS_2638) from Nros Rd., 20 miles W of Mercury, Nye Co., Nevada, 36.66182 -116.368 ^5^, elev. 2744ft., 3.xi.1972, coll. C.S.L.; deposited in AUMNH.

#### Etymology.

The specific epithet is a neuter noun meaning “of or relating to atoms”. The name references the Nevada Test Site, constructed by the Atomic Energy Commission for the testing of nuclear devices, where this species was originally collected. The name is in homage to the famous sci-fi B movies of the 1950’s, of which *Tarantula* (1955) was the most entertaining, and slightly ironic given that this species is one of the smallest tarantulas in the United States.

#### Diagnosis.

*Aphonopelma atomicum* (Fig. 20) is a member of the *paloma* species group and can be distinguished by a combination of morphological, molecular, and geographic characteristics. Nuclear and mitochondrial DNA identifies *Aphonopelma atomicum* as a strongly supported monophyletic lineage (Figs 7–8) that is a sister lineage to *Aphonopelma mojave*, *Aphonopelma icenoglei* sp. n., *Aphonopelma prenticei* sp. n., and *Aphonopelma joshua*. *Aphonopelma atomicum* can easily be differentiated from larger syntopic species (e.g., *Aphonopelma iodius*) due to their much smaller size and the extent of metatarsal scopulation on legs III and IV, and from all other miniaturized species of *Aphonopelma* by their locality (with the exception of *Aphonopelma prenticei*). Molecular data is needed to differentiate this species from *Aphonopelma prenticei* (see Figs 7, 8). The most significant measurements that distinguish male *Aphonopelma atomicum* from its closely related phylogenetic and syntopic species are F3 and the extent of metatarsus IV scopulation. Male *Aphonopelma atomicum* can be distinguished by possessing a larger F3/M3 (≥0.99; 0.99–1.03) than *Aphonopelma joshua* (≤0.98; 0.91–0.98) and *Aphonopelma xwalxwal* sp. n. (≤0.95; 0.91–0.95); a smaller F3L/W (≤3.40; 2.92–3.40) than *Aphonopelma icenoglei* (≥3.51; 3.51–3.90); and a smaller L4 scopulation extent (26%-41%) than *Aphonopelma iodius* (62%-88%). There are no significant measurements that separate male *Aphonopelma atomicum* from *Aphonopelma mojave* or *Aphonopelma prenticei* (*Aphonopelma atomicum* males possess a swollen F3 femur like *Aphonopelma prenticei*, whereas *Aphonopelma mojave* males do not). The most significant measurements that distinguish female *Aphonopelma atomicum* from its closely related phylogenetic and syntopic species are F3 and the extent of metatarsus IV scopulation. Female *Aphonopelma atomicum* can be distinguished by possessing a smaller A1/F3 (≤0.56; 0.53–0.56) than *Aphonopelma mojave* (>0.56; 0.56–0.67), *Aphonopelma icenoglei* (≥0.59; 0.59–0.67), and *Aphonopelma joshua* (≥0.57; 0.57–0.64); a smaller Cl/F3 (≤1.38; 1.31–1.38) than *Aphonopelma prenticei* (≥1.39; 1.39–1.64); and smaller L4 scopulation extent (37%–49%) than *Aphonopelma iodius* (59%–83%).

#### Description of male holotype

(AUMS_2727-2; Fig. 21). *Specimen preparation and condition*: Specimen originally preserved in unknown percentage of ethanol; original coloration faded due to preservation. Missing leg IV right side. Left legs I, III, IV, and left pedipalp removed for measurements and photographs; stored in vial with specimen. No tissue for DNA. *General coloration*: Brown/faded brown. *Cephalothorax*: Carapace 6.523 mm long, 6.382 mm wide; densely clothed with brown/faded brown pubescence, appressed to surface; fringe covered in long setae not closely appressed to surface; foveal groove medium deep and straight; pars cephalica region rises gradually from foveal groove on a straight plane towards the ocular area; AER slightly procurved, PER slightly recurved; normal sized chelicerae; clypeus mostly straight; LBl 0.918, LBw 1.107; sternum hirsute, clothed with faded brown, densely packed, short setae. *Abdomen*: Densely clothed in short brown pubescence with numerous longer, lighter setae interspersed (generally red or orange *in situ*); dense dorsal patch of black Type I urticating bristles (Cooke et al. 1972) - smaller and distinct from large species. *Legs*: Hirsute; densely clothed in faded brown pubescence. Metatarsus I straight/very slightly curved. F1 7.691; F1w 1.62; P1 3.012; T1 6.618; M1 5.767; A1 3.83; F3 6.665; F3w 1.958; P3 2.436; T3 5.294; M3 6.438; A3 4.207; F4 7.922; F4w 1.596; P4 2.656; T4 6.615; M4 8.407; A4 4.358; femur III is swollen/slightly swollen. All tarsi fully scopulate. Extent of metatarsal scopulation: leg III (SC3) = 61.4%; leg IV (SC4) = 37.3%. No distinct ventral spinose setae on metatarsus III; three ventral spinose setae on metatarsus IV; two prolateral spinose setae on tibia I; one megaspine present on the retrolateral tibia, at the apex of the mating clasper; three megaspines on the apex of the retrolateral branch of the tibial apophyses. *Coxa I*: Prolateral surface covered by fine, hair-like setae. *Pedipalps*: Hirsute; densely clothed in the same setal color as the other legs, with numerous longer ventral setae; one spinose seta at the apical, prolateral femur and two prolateral spinose setae on the palpal tibia; PTl 4.486, PTw 1.51. When extended, embolus tapers with a curve to the retrolateral side; embolus slender, no keels; distinct dorsal and ventral transition from bulb to embolus.

**Variation (5).** Cl 6.523–7.917 (7.157±0.23), Cw 6.382–6.798 (6.64±0.09), LBl 0.832–0.988 (0.927±0.03), LBw 1.098–1.394 (1.231±0.06), F1 7.434–8.185 (7.761±0.12), F1w 1.565–1.914 (1.783±0.08), P1 2.859–3.481 (3.12±0.11), T1 6.618–7.496 (7.053±0.16), M1 5.767–6.402 (6.083±0.12), A1 3.768–4.149 (3.976±0.08), L1 length 26.653–28.926 (27.993±0.5), F3 6.665–7.388 (6.962±0.12), F3w 1.958–2.454 (2.223±0.09), P3 2.355–2.84 (2.562±0.08), T3 5.294–5.812 (5.58±0.1), M3 6.438–7.349 (6.88±0.15), A3 4.204–4.298 (4.252±0.02), L3 length 25.04–27.374 (26.236±0.39), F4 7.773–8.406 (8.073±0.11), F4w 1.596–2.045 (1.816±0.08), P4 2.621–2.9 (2.786±0.06), T4 6.615–7.533 (7.11±0.17), M4 8.197–9.072 (8.573±0.15), A4 4.358–4.937 (4.64±0.1), L4 length 29.958–32.832 (31.181±0.54), PTl 4.486–4.966 (4.689±0.08), PTw 1.433–1.736 (1.564±0.06), SC3 ratio 0.552–0.725 (0.626±0.03), SC4 ratio 0.264–0.409 (0.356±0.03), Coxa I setae = very thin tapered, F3 condition = slightly swollen/swollen.

#### Description of female paratype

(AUMS_3267-2; Figs 22–23). *Specimen preparation and condition*: Specimen originally preserved in unknown percentage of ethanol; original coloration faded due to preservation. Left legs I, II, III, IV, and pedipalp removed for photographs and measurements; stored in vial with specimen. No tissue for DNA. Genital plate with spermathecae removed and cleared, stored in vial with specimen. *General coloration*: Brown and faded brown. *Cephalothorax*: Carapace 7.291 mm long, 6.814 mm wide; Hirsute, densely clothed with short faded brown pubescence closely appressed to surface; fringe densely covered in slightly longer setae; foveal groove medium deep and procurved; pars cephalica region gently rises from thoracic furrow, arching anteriorly toward ocular area; AER slightly procurved, PER very slightly recurved - mostly straight; chelicerae robust, clypeus extends forward on a slight curve; LBl 1.058, LBw 1.416; sternum hirsute, clothed with short faded brown setae. *Abdomen*: Densely clothed dorsally in short faded brown setae with longer, lighter setae (generally red or orange *in situ*) focused near the urticating patch; dense dorsal patch of black Type I urticating bristles (Cooke et al. 1972) - smaller and distinct from large species. *Spermathecae*: Paired and separate, short, with capitate bulbs widening towards the bases; not fused. *Legs*: Hirsute; densely clothed in short faded brown pubescence; F1 6.49; F1w 1.891; P1 2.912; T1 5.232; M1 3.952; A1 3.102; F3 5.539; F3w 1.789; P3 2.418; T3 4.188; M3 4.363; A3 3.418; F4 6.898; F4w 1.766; P4 2.753; T4 5.716; M4 5.993; A4 3.839. All tarsi fully scopulate. Extent of metatarsal scopulation: leg III (SC3) = 68.3%; leg IV (SC4) = 37.2%. One ventral spinose setae on metatarsus III; four ventral spinose setae on metatarsus IV, with numerous thickened setae throughout. *Coxa I*: Prolateral surface covered by very thin tapered and fine, hair-like setae. *Pedipalps*: Densely clothed in the same setal color as the other legs; one spinose seta on the apical, prolateral femur, five prolateral (three at the apical, prolateral border with the tarsus) spinose setae and one ventral spinose seta on the tibia.

**Variation (3).** Cl 7.081–7.978 (7.45±0.27), Cw 5.951–7.105 (6.623±0.35), LBl 0.922–1.218 (1.066±0.09), LBw 1.105–1.621 (1.381±0.15), F1 5.656–7.25 (6.465±0.46), F1w 1.663–2.032 (1.862±0.11), P1 2.912–3.253 (3.026±0.11), T1 5.232–5.714 (5.394±0.16), M1 3.461–4.532 (3.982±0.31), A1 2.787–3.179 (3.023±0.12), L1 length 20.052–23.928 (21.889±1.12), F3 5.11–5.984 (5.544±0.25), F3w 1.493–1.907 (1.73±0.12), P3 2.046–2.81 (2.425±0.22), T3 3.603–4.223 (4.005±0.2), M3 3.981–4.61 (4.318±0.18), A3 3.305–3.641 (3.455±0.1), L3 length 18.045–21.268 (19.746±0.93), F4 6.234–7.413 (6.848±0.34), F4w 1.473–1.956 (1.732±0.14), P4 2.541–2.92 (2.738±0.11), T4 5.234–6.039 (5.663±0.23), M4 5.687–6.245 (5.975±0.16), A4 3.446–3.86 (3.715±0.13), L4 length 23.142–26.477 (24.939±0.97), SC3 ratio 0.683–0.755 (0.726±0.02), SC4 ratio 0.372–0.49 (0.439±0.03), Coxa I setae = thin tapered. Spermathecae variation can be seen in Figure 23.

#### Material examined.

**United States: California: Inyo**: Death Valley National Park, Hwy-178, Salsberry Pass, 35.923118 -116.431747 ^1^, 3204ft., [APH_1478, 30/7/2012, 1♀, Brent E. Hendrixson, Brendon Barnes, Austin Deskewies, AUMNH]; Death Valley National Park, ~7.5 miles S Hwy-190 on Dante’s View Rd, 36.267976 -116.665576 ^1^, 3242ft., [APH_1479, 30/7/2012, 1 juv, Brent E. Hendrixson, Brendon Barnes, Austin Deskewies, AUMNH]; **Nevada: Nye**: Rock Valley Wash, off Hwy-95, 36.622963 -116.309464 ^1^, 2784ft., [APH_1549, 24/10/2012, 1♀, Brent E. Hendrixson, AUMNH]; Rock Valley, 36.632732 -116.313933 ^5^, 2884ft., [AUMS_2637, 20/10/1972, 1♀, E.L. Sleeper, AUMNH]; Mercury, 36.66051 -115.994475 ^5^, 3796ft., [APH_2725-1, 6/9/1960, 2♀, 14♂, Gertsch, AMNH]; Nevada Testing Site, 37.025579 -116.023865 ^6^, 3990ft., [APH_2725-2, 1/10/1959, 3♂, JRM, AMNH]; [APH_2727, 29/6/1961, 3♀, 15♂, Gertsch, AMNH]; [APH_2727-2, 8/1961, 1♂, Gertsch, AMNH]; [APH_2730, 16/8/1964, 1♀, unknown, AMNH]; Nros Rd., 20 miles W of Mercury, 36.66182 -116.368 ^5^, 2744ft., [AUMS_2638, 3/11/1972, 1♂, C. S. L., AUMNH]; 10 miles W of Mercury, Mercury Valley, 36.691928 -116.174483 ^5^, 3470ft., [AUMS_3267 & 3267-2, 3/11/1972, 2♀, W. Icenogle, AUMNH].

#### Distribution and natural history.

*Aphonopelma atomicum* is only known from a handful of specimens surrounding the Amargosa Desert in southern Nye County (Nevada) and southeastern Inyo County (California), including the Amargosa Range and Nevada Test Site (Fig. 24). Specimens have been collected at elevations between 850 and 1220 meters, inhabiting the Mojave Basin and Range Level III Ecoregion. This species is likely syntopic with *Aphonopelma iodius* throughout its range and may be found near populations of *Aphonopelma prenticei*. Burrow entrances are generally surrounded by a distinct mound or turret made of excavated soil and silk (Fig. 2D–E). Mating most likely occurs during daylight hours in autumn (October-November).

#### Conservation status.

*Aphonopelma atomicum* has a highly restricted distribution limited to the mountains and foothills surrounding the Amargosa Desert and Death Valley. While this species is not dramatically different from *Aphonopelma prenticei*, it is genetically unique and should be considered important. The species is most likely secure.

#### Remarks.

*Aphonopelma atomicum* is unique because it was quite possibly the first miniature tarantula species collected in the United States (although it was never described and sat on a shelf in the AMNH collection). It is somewhat puzzling that Gertsch never described this species given the number of individuals he collected and how radically different (i.e., small in size) specimens are from all other *Aphonopelma* in that region. Other important ratios that distinguish males: *Aphonopelma atomicum* possess a smaller A1/F3 (≤0.58; 0.55–0.58) than *Aphonopelma mojave* (≥0.58; 0.58–0.63) and *Aphonopelma prenticei* (≥0.58; 0.58–0.65). Certain morphometrics have potential to be useful, though due to the amounts of variation, small number of specimens, and the small differences between species, no other are claimed to be significant at this time (see Suppl. material 2). During evaluation of traditional two-dimensional PCA morphospace and three-dimensional PCA morphospace (PC1~PC2~PC3), males of *Aphonopelma atomicum* separate from *Aphonopelma iodius*, *Aphonopelma joshua*, and *Aphonopelma xwalxwal* along PC1~2, but do not separate from *Aphonopelma mojave*, *Aphonopelma icenoglei*, or *Aphonopelma prenticei*. Female *atomicum* separate from *Aphonopelma iodius* in morphological space, but do not separate from the other miniature species (*Aphonopelma mojave*, *Aphonopelma icenoglei*, and *Aphonopelma prenticei*). There are no known female *Aphonopelma xwalxwal* at this time to compare. PC1, PC2, and PC3 explain ≥97% of the variation in all analyses.

### Aphonopelma catalina

Taxon classificationAnimaliaAraneaeTheraphosidae

Hamilton, Hendrixson & Bond sp. n.

http://zoobank.org/A0661186-E6EE-47D6-8C99-8496ECE41DDE

**Figure 25.**
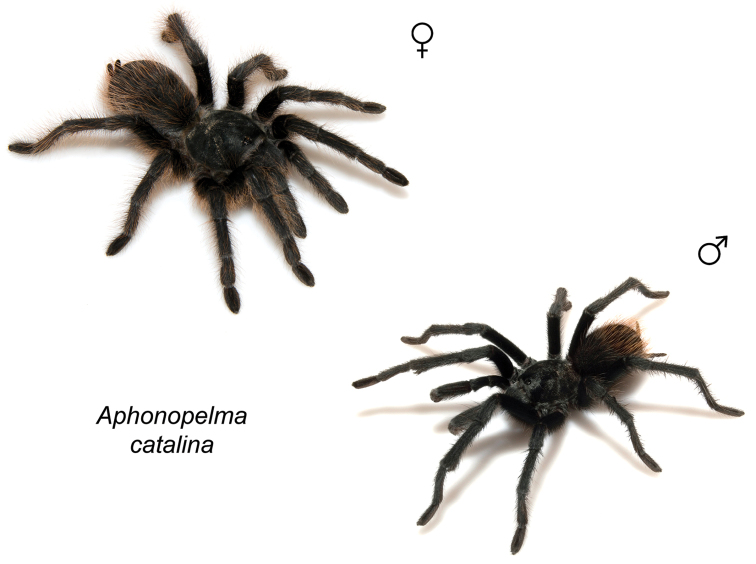
*Aphonopelma catalina* sp. n. specimens, live photographs. Female paratype (L) - APH_1602; Male (R) - APH_1438.

**Figure 26.**
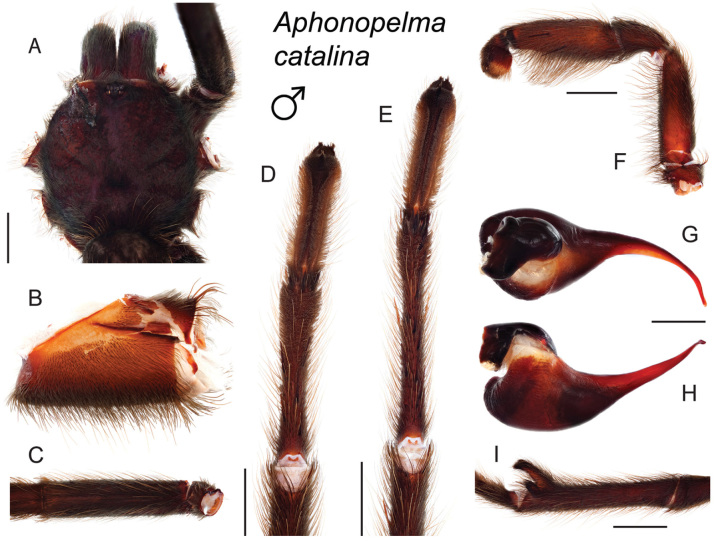
*Aphonopelma catalina* sp. n. **A–I** male holotype, APH_1440 **A** dorsal view of carapace, scale bar = 4mm **B** prolateral view of coxa I **C** dorsal view of femur III **D** ventral view of metatarsus III, scale bar = 3mm **E** ventral view of metatarsus IV, scale bar = 4mm **F** prolateral view of L pedipalp and palpal tibia, scale bar = 4mm **G** dorsal view of palpal bulb **H** retrolateral view of palpal bulb, scale bar = 1mm **I** prolateral view of tibia I (mating clasper), scale bar = 4.5mm.

**Figure 27.**
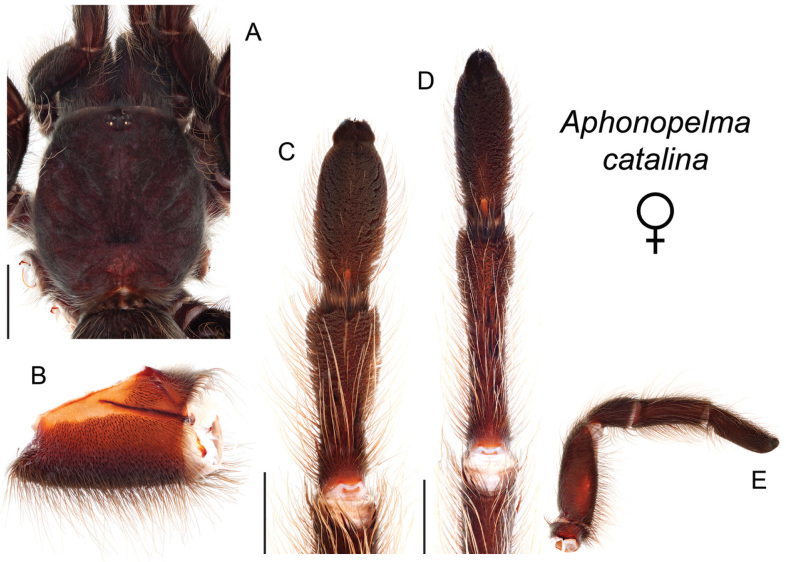
*Aphonopelma catalina* sp. n. **A–E** female paratype, APH_1602 **A** dorsal view of carapace, scale bar = 6mm **B** prolateral view of coxa I **C** ventral view of metatarsus III, scale bar = 3mm **D** ventral view of metatarsus IV, scale bar = 3mm **E** prolateral view of L pedipalp and palpal tibia.

**Figure 28.**
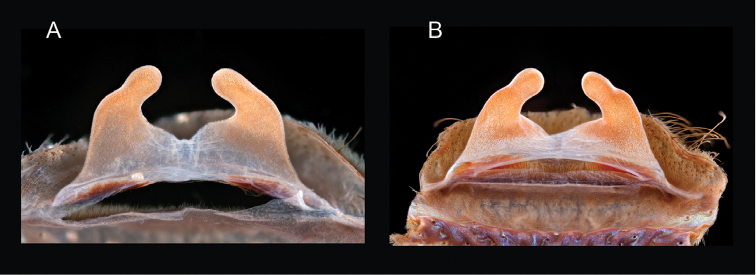
*Aphonopelma catalina* sp. n. cleared spermathecae. **A** APH_1602 **B** AUMS_2615.

**Figure 29.**
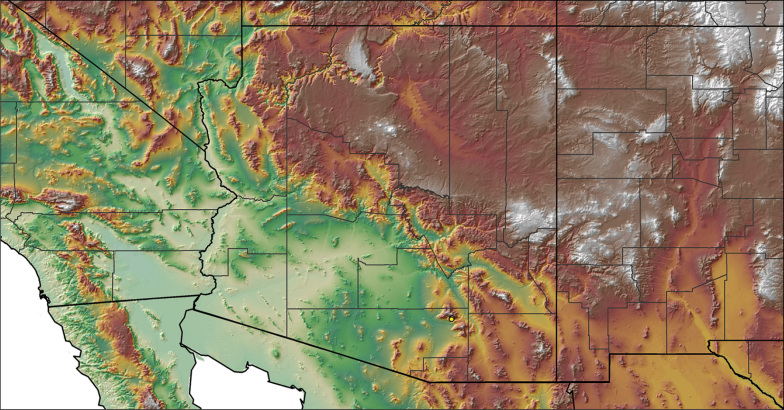
*Aphonopelma catalina* sp. n. distribution of known specimens. There is no predicted distribution map due to the limited number of sampling localities and restricted distribution this species possesses.

Figures 25 , 26 , 27 , 28 , 29

#### Types.

Male holotype (APH_1440) from Coronado National Forest, along Bug Spring Trail, Pima Co., Arizona, 32.34544 -110.71602 ^4^, elev. 5255ft., 17.xii.2011, coll. Brent E. Hendrixson and Thomas Martin; deposited in AUMNH. Paratype female (APH_1602) from Coronado National Forest, along Bug Spring Trail, Pima Co., Arizona, 32.34544 -110.71602 ^4^, elev. 5255ft., 9.xi.2012, coll. Brent E. Hendrixson; deposited in AUMNH. Paratype male (APH_1439) from Coronado National Forest, along Bug Spring Trail, Pima Co., Arizona, 32.34544 -110.71602 ^4^, elev. 5255ft., 17.xii.2011, coll. Brent E. Hendrixson and Thomas Martin; deposited in AMNH.

#### Etymology.

The specific epithet is a noun in apposition taken from type locality, the Santa Catalina Mountains near Tucson, Arizona, where this new species appears to be endemic.

#### Diagnosis.

*Aphonopelma catalina* (Fig. 25) is a member of the *Marxi* species group and can be distinguished by a combination of morphological, molecular, and geographic characteristics. Nuclear and mitochondrial DNA identifies *Aphonopelma catalina* as a phylogenetically distinct monophyletic lineage (Figs 7–8), supported as a sister lineage to *Aphonopelma chiricahua* sp. n. (a species endemic to the Chiricahua Mountains). The significant measurement that distinguishes male *Aphonopelma catalina* from its closely related phylogenetic and syntopic species is F1. Male *Aphonopelma catalina* can be distinguished by possessing a larger F1/A3 (≥2.25; 2.25–2.49) than *Aphonopelma chiricahua* (≤1.86; 1.55–1.86), *Aphonopelma madera* sp. n. (≤2.14; 2.02–2.14), *Aphonopelma parvum* sp. n. (≤1.98; 1.79–1.98), *Aphonopelma peloncillo* sp. n. (≤2.23; 1.86–2.23), *Aphonopelma saguaro* sp. n. (1.96 ± (only 1 specimen)), and *Aphonopelma vorhiesi* (≤2.17; 1.71–2.17). Significant measurements that distinguish female *Aphonopelma catalina* from its closely related phylogenetic and syntopic species are Cl and M3. Female *Aphonopelma catalina* can be distinguished by possessing a larger M3/A4 (≥1.07; 1.07–1.10) than *Aphonopelma chiricahua* (0.80 ± (only 1 specimen)), *Aphonopelma madera* (≤1.07; 0.96–1.07), *Aphonopelma parvum* (≤0.92; 0.86–0.92), and *Aphonopelma saguaro* (0.92 ± (only 1 specimen)), but smaller than *Aphonopelma peloncillo* (≥1.11; 1.11–1.23); and by possessing a smaller Cl/F4 (≤1.18; 1.17–1.18) than *Aphonopelma chiricahua* (1.21 ± (only 1 specimen)), *Aphonopelma madera* (≥1.20; 1.20–1.27), *Aphonopelma peloncillo* (≥1.22; 1.22–1.34), and *Aphonopelma vorhiesi* (≥1.23; 1.23–1.37).

#### Description of male holotype

(APH_1440; Fig. 26). *Specimen preparation and condition*: Specimen collected live crossing trail, preserved in 80% ethanol; original coloration faded due to preservation. Left legs I, III, IV, and left pedipalp removed for measurements and photographs; stored in vial with specimen. Right leg III removed for DNA and stored at -80°C in the AUMNH (Auburn, AL). *General coloration*: Generally black or faded black. *Cephalothorax*: Carapace 12.39 mm long, 11.33 mm wide; densely clothed with black/faded black pubescence, appressed to surface; fringe covered in long setae not closely appressed to surface; foveal groove medium deep and procurved; pars cephalica region rises gradually from foveal groove, gently arching anteriorly toward ocular area; AER procurved, PER recurved; normal sized chelicerae; clypeus straight; LBl 1.18, LBw 1.56; sternum hirsute, clothed with short black/brown, densely packed setae. *Abdomen*: Densely clothed in short black/brown pubescence with numerous longer, lighter setae interspersed (generally red or orange *in situ*); dense dorsal patch of black Type I urticating bristles (Cooke et al. 1972); ventral setae same as dorsal. *Legs*: Hirsute; densely clothed with short, similar length black/brown setae, and longer setae dorsally. Metatarsus I very slightly curved. F1 14.64; F1w 2.83; P1 5.31; T1 12.78; M1 8.91; A1 5.95; F3 10.40; F3w 2.88; P3 3.89; T3 8.63; M3 9.18; A3 5.89; F4 12.90; F4w 2.78; P4 4.54; T4 10.96; M4 12.34; A4 7.11; femur III is normal - not noticeably swollen or wider than other legs. All tarsi fully scopulate. Extent of metatarsal scopulation: leg III (SC3) = 53.0%; leg IV (SC4) = 30.6%. Five ventral spinose setae on metatarsus III; eleven ventral spinose setae, one prolateral and one retrolateral spinose seta on metatarsus IV; one large megaspine is present on the retrolateral tibia at the apex of the mating clasper - this can be seen when viewing the prolateral face of the mating clasper; the prolateral branch of the tibial apophyses possesses a very large megaspine that projects anteriorly. *Coxa I*: Prolateral surface a mix of fine, hair-like and tapered/thin tapered setae. *Pedipalps*: Hirsute; densely clothed in the same setal color as the other legs, with numerous longer ventral setae; one spinose seta at the apical, prolateral femur and two spinose setae on the prolateral tibia; PTl 8.058, PTw 2.911. When extended, embolus tapers with a gentle curve to the retrolateral side near apex; embolus slender, no keels.

**Variation (4).** Cl 9.57–12.39 (10.595±0.62), Cw 8.88–11.33 (9.708±0.55), LBl 0.967–1.237 (1.114±0.06), LBw 1.102–1.56 (1.354±0.11), F1 11.28–14.64 (12.765±0.7), F1w 2.18–2.83 (2.467±0.14), P1 3.73–5.31 (4.483±0.32), T1 9.89–12.78 (11.132±0.61), M1 6.42–8.91 (7.446±0.54), A1 5.0–5.95 (5.392±0.2), L1 length 36.32–47.59 (41.217±2.34), F3 7.97–10.4 (9.098±0.5), F3w 2.1–2.88 (2.465±0.16), P3 3.04–4.084 (3.551±0.26), T3 5.74–8.63 (7.039±0.6), M3 6.72–9.18 (7.882±0.51), A3 4.82–5.89 (5.37±0.23), L3 length 28.29–37.99 (32.94±2.02), F4 9.75–12.9 (11.077±0.66), F4w 2.05–2.78 (2.357±0.15), P4 3.26–4.54 (3.902±0.26), T4 7.82–10.96 (9.444±0.64), M4 9.26–12.34 (10.762±0.63), A4 5.41–7.11 (6.095±0.4), L4 length 35.5–47.85 (41.279±2.53), PTl 6.587–8.058 (7.173±0.32), PTw 2.328–2.911 (2.572±0.12), SC3 ratio 0.481–0.53 (0.514±0.01), SC4 ratio 0.228–0.359 (0.286±0.03), Coxa I setae = tapered/thin tapered, F3 condition = normal.

#### Description of female paratype

(APH_1602; Figs 27–28). *Specimen preparation and condition*: Specimen collected live from burrow, preserved in 80% ethanol; original coloration faded due to preservation. Left legs I, III, IV, and pedipalp removed for photographs and measurements; stored in vial with specimen. Right leg III removed for DNA and stored at -80°C in the AUMNH (Auburn, AL). Genital plate with spermathecae removed and cleared, stored in vial with specimen. *General coloration*: Black/faded black and brown. *Cephalothorax*: Carapace 14.79 mm long, 13.74 mm wide; Hirsute, densely clothed with black/faded black, pubescence closely appressed to surface; fringe densely covered in longer setae; foveal groove medium deep and straight; pars cephalica region gently rises from thoracic furrow, arching anteriorly toward ocular area; AER very slightly procurved, PER straight; robust chelicerae, clypeus extends forward on a slight curve; LBl 1.81, LBw 1.97; sternum hirsute, clothed with shorter black/faded black setae. *Abdomen*: Densely clothed dorsally in short black setae with numerous longer, lighter setae interspersed (generally red or orange *in situ*); dense dorsal patch of black Type I urticating bristles (Cooke et al. 1972); ventral setae same as dorsal. *Spermathecae*: Paired and separate, tapering and slightly curving medially towards capitate bulbs, with wide bases that appear fused. *Legs*: Very hirsute, particularly ventrally; densely clothed in short and medium black pubescence, with longer setae colored similarly as the long abdominal setae; F1 11.87; F1w 3.84; P1 5.36; T1 9.77; M1 6.92; A1 6.04; F3 9.74; F3w 3.32; P3 4.52; T3 7.26; M3 7.29; A3 6.07; F4 12.52; F4w 3.41; P4 4.92; T4 10.00; M4 10.98; A4 6.64. All tarsi fully scopulate. Extent of metatarsal scopulation: leg III (SC3) = 68.6%; leg IV (SC4) = 46.5%. Six ventral spinose setae on metatarsus III; nine ventral spinose setae and one prolateral spinose seta on metatarsus IV. *Coxa I*: Prolateral surface a mix of fine, hair-like and tapered setae. *Pedipalps*: Densely clothed in the same setal color as the other legs; two prolateral spinose setae and one ventral spinose seta on the tibia.

**Variation (2).** Cl 14.79–16.39 (15.59±0.8), Cw 13.74–15.06 (14.4±0.66), LBl 1.81–2.03 (1.92±0.11), LBw 1.97–2.67 (2.32±0.35), F1 11.87–13.85 (12.86±0.99), F1w 3.84–4.24 (4.04±0.2), P1 5.36–5.58 (5.47±0.11), T1 9.77–11.33 (10.55±0.78), M1 6.92–7.57 (7.245±0.33), A1 6.04–6.05 (6.045±0), L1 length 39.96–44.38 (42.17±2.21), F3 9.74–11.31 (10.525±0.79), F3w 3.32–3.78 (3.55±0.23), P3 4.52–5.33 (4.925±0.41), T3 7.26–8.35 (7.805±0.55), M3 7.29–8.42 (7.855±0.57), A3 6.07–6.77 (6.42±0.35), L3 length 34.88–40.18 (37.53±2.65), F4 12.52–13.95 (13.235±0.72), F4w 3.41–3.86 (3.635±0.23), P4 4.92–5.54 (5.23±0.31), T4 10.0–11.17 (10.585±0.59), M4 10.98–11.74 (11.36±0.38), A4 6.64–7.84 (7.24±0.6), L4 length 45.06–50.24 (47.65±2.59), SC3 ratio 0.629–0.686 (0.657±0.03), SC4 ratio 0.37–0.465 (0.418±0.05), Coxa I setae = medium tapered. Spermathecae variation can be seen in Figure 28.

#### Material examined.

**United States: Arizona: Pima**: Coronado National Forest, along Bug Spring Trail, 32.34544 -110.71602 ^4^, 5255ft., [APH_0454, 28/12/2008, 1♂, Paul E. Marek, Charity Hall, AUMNH]; [APH_1438, 11/12/2011, 1♂, Jillian Cowles, Bill Savary, AUMNH]; [APH_1439-1440, 17/12/2011, 2♂, Brent E. Hendrixson, Thomas Martin, AUMNH & AMNH]; [APH_1602, 9/11/2012, 1♀, Brent E. Hendrixson, AUMNH]; .5 miles W of Catalina Hwy, 2 miles N of Molino Basin, Santa Catalina Mtns, 32.336526 -110.718179 ^4^, 4886ft., [AUMS_2615, 15/9/1974, 1♀, W. Icenogle, AUMNH].

#### Distribution and natural history.

*Aphonopelma catalina* is known from only six individuals collected within a few kilometers of each other but this species appears to be a sky island endemic found in the Santa Catalina Mountains in Pima County, Arizona at elevations above 1480 meters in oak-grassland communities (Figs 1, 29); it is possible that this species is also found in bordering sections of the Rincon Mountains but the range has not been thoroughly sampled for tarantulas. *Aphonopelma catalina* can be found inhabiting the Madrean Archipelago Level III Ecoregion; the upper elevational limit for *Aphonopelma catalina* remains unknown but the species can likely be found in adjacent oak and pine-oak woodlands at higher elevations similar to its close relatives *Aphonopelma chiricahua* and *Aphonopelma madera*. This species has not been observed in syntopy with other *Aphonopelma* spp. but can probably be found alongside *Aphonopelma chalcodes* and *Aphonopelma vorhiesi* in oak-grassland communities along the lower elevations of the Santa Catalina Mountains. Only a single burrow (of an adult female, APH_1602, Fig. 25) of this species has been observed, located a few meters from a hiking trail surrounded by grasses and large rocks. The structure of the burrow was fairly typical for a North American theraphosid (i.e., circular and covered by a thin veil of silk) and did not appear too deep or well drained; the specimen was easily flooded from its burrow with less than a liter of water. Each adult male of *Aphonopelma catalina* examined in this study was found during daylight hours in December, suggesting that the breeding period for this species is restricted to late autumn and early winter; one of these males was found wandering along a snow bank on a sunny afternoon (APH_0454, P. Marek 2008, pers. comm.). An unconfirmed adult male (no voucher specimen available, identification tentatively assigned based on a photograph and locality data) was found above 1760 meters in an oak woodland community during early February (P. MacDuff 2015, pers. comm.).

#### Conservation status.

It is difficult to fully assess the distribution and abundance (and therefore the conservation status) of *Aphonopelma catalina* due to a lack of specimens and thorough sampling; however, as previously mentioned, the species appears to be narrowly endemic to the Santa Catalina Mountains, which may put the species at some risk. This mountain range is entirely contained within the Coronado National Forest (Santa Catalina Ranger District) which is afforded some degree of protection; however, increased urbanization of the Tucson Metropolitan Area (one of the most rapidly growing areas in the United States), increased recreation in the mountains, and climate change have impacted these habitats (Coronado Planning Partnership 2008, Brusca et al. 2013, Moore et al. 2013, Hendrixson et al. 2015) and pose additional threats to *Aphonopelma catalina*.

#### Remarks.

As noted in Hendrixson et al. (2015), *Aphonopelma catalina* is morphologically very similar to other Madrean sky island endemics in the *Marxi* species group, although generally larger than *Aphonopelma chiricahua* and *Aphonopelma madera* and possess a more hirsute appearance than *Aphonopelma vorhiesi*. Other important ratios that distinguish males: *Aphonopelma catalina* possess a larger T1/M3 (≥1.39; 1.39–1.47) than *Aphonopelma parvum* (≤1.34; 1.16–1.34) and *Aphonopelma saguaro* (1.16 ± (only 1 specimen)); by possessing a smaller Cl/T1 (≤0.97; 0.90–0.97) than *Aphonopelma chiricahua* (≥1.00; 1.00–1.17), *Aphonopelma madera* (≥1.06; 1.06–1.12), *Aphonopelma peloncillo* (≥1.01; 1.01–1.22), and *Aphonopelma vorhiesi* (≥0.99; 0.99–1.34). Other important ratios that distinguish females: *Aphonopelma catalina* possess a smaller A3/M4 (≤0.58; 0.55–0.58) than *Aphonopelma chiricahua* (0.71 ± (only 1 specimen)), *Aphonopelma parvum* (≥0.60; 0.60–0.69), and *Aphonopelma saguaro* (0.67 ± (only 1 specimen)); by possessing a smaller Cl/M3 (≤2.03; 1.94–2.03) than *Aphonopelma chiricahua* (2.36 ± (only 1 specimen)), *Aphonopelma madera* (≥2.06; 2.06–2.31), and *Aphonopelma saguaro* (2.10 ± (only 1 specimen)); by possessing a smaller Cl/Cw (≤1.09; 1.07–1.09) than *Aphonopelma chiricahua* (1.02 ± (only 1 specimen)), *Aphonopelma peloncillo* (≥1.11; 1.11–1.17), and *Aphonopelma vorhiesi* (≥1.11; 1.11–1.21). For both males and females, certain morphometrics have potential to be useful, though due to the amounts of variation, small number of specimens, and the small differences between species, no other are claimed to be significant at this time (see Suppl. material 2). During evaluation of PCA morphospace, males of *Aphonopelma catalina* separate from *Aphonopelma chalcodes*, *Aphonopelma peloncillo*, *Aphonopelma vorhiesi*, and all miniature species along PC1~2, but do not separate from *Aphonopelma chiricahua*, *Aphonopelma madera*, and *Aphonopelma marxi*. Females of *Aphonopelma catalina* separate from *Aphonopelma chalcodes*, *Aphonopelma chiricahua* and all miniature species along PC1~2, but do not separate from *Aphonopelma madera*, *Aphonopelma marxi*, *Aphonopelma peloncillo*, and *Aphonopelma vorhiesi*. Interestingly, *Aphonopelma catalina* males separate from *Aphonopelma chalcodes*, *Aphonopelma peloncillo*, *Aphonopelma saguaro*, and *Aphonopelma vorhiesi* in three-dimensional PCA morphospace (PC1~PC2~PC3), but do not separate from *Aphonopelma chiricahua*, *Aphonopelma madera*, and *Aphonopelma marxi*. *Aphonopelma catalina* females separate from *Aphonopelma chalcodes*, *Aphonopelma chiricahua*, *Aphonopelma marxi*, and *Aphonopelma saguaro* but do not separate from *Aphonopelma madera*, *Aphonopelma peloncillo*, and *Aphonopelma vorhiesi*. PC1, PC2, and PC3 explain ≥96% of the variation in all analyses.

### Aphonopelma chalcodes

Taxon classificationAnimaliaAraneaeTheraphosidae

Chamberlin, 1940

**Figure 30.**
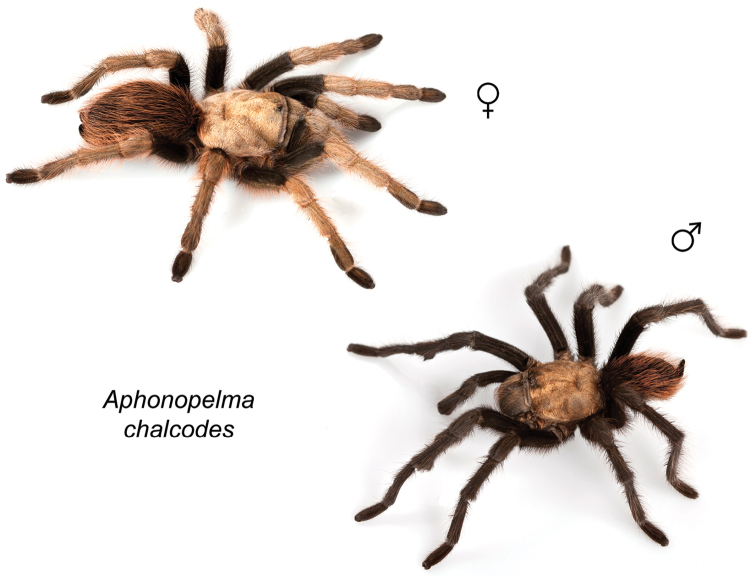
*Aphonopelma chalcodes* Chamberlin, 1940 specimens, live photographs. Female (L) - APH_0697; Male (R) - APH_0600.

**Figure 31.**
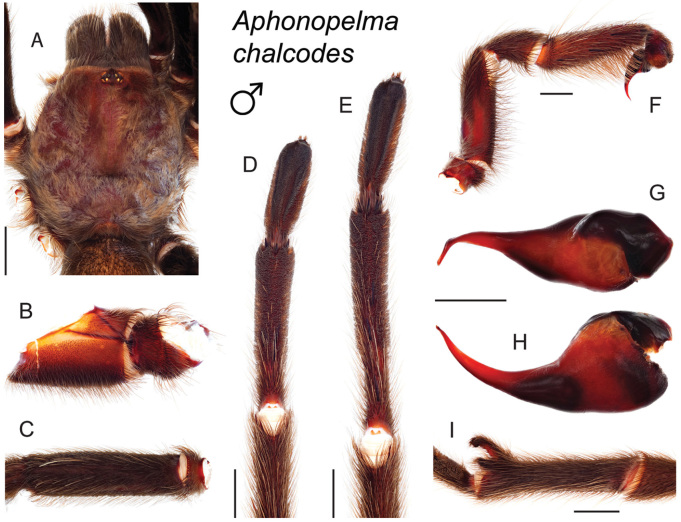
*Aphonopelma chalcodes* Chamberlin, 1940. **A–I** male specimen, APH_0954 **A** dorsal view of carapace, scale bar = 5mm **B** prolateral view of coxa I **C** dorsal view of femur III **D** ventral view of metatarsus III, scale bar = 4mm **E** ventral view of metatarsus IV, scale bar = 3.5mm **F** prolateral view of L pedipalp and palpal tibia, scale bar = 3mm **G** dorsal view of palpal bulb **H** retrolateral view of palpal bulb, scale bar = 1.5mm **I** prolateral view of tibia I (mating clasper), scale bar = 4.5mm.

**Figure 32.**
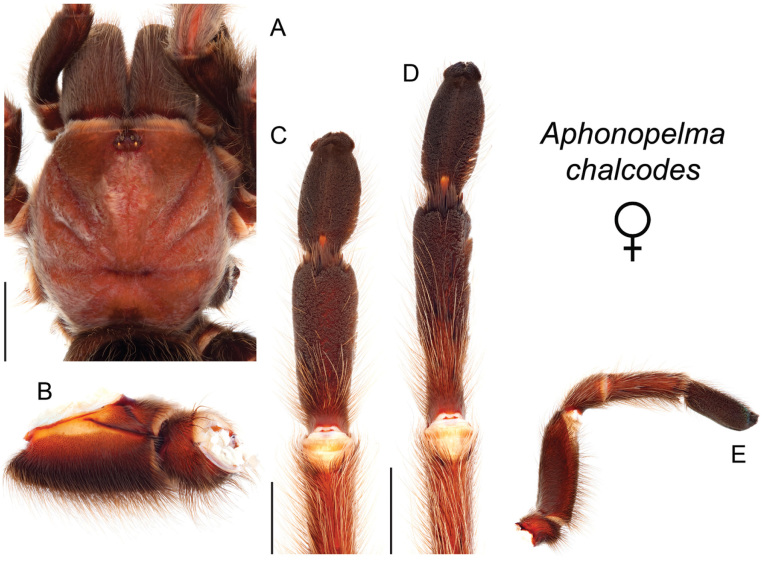
*Aphonopelma chalcodes* Chamberlin, 1940. **A–E** female specimen, APH_0887 **A** dorsal view of carapace, scale bar = 8mm **B** prolateral view of coxa I **C** ventral view of metatarsus III, scale bar = 4.5mm **D** ventral view of metatarsus IV, scale bar = 5mm **E** prolateral view of L pedipalp and palpal tibia.

**Figure 33.**
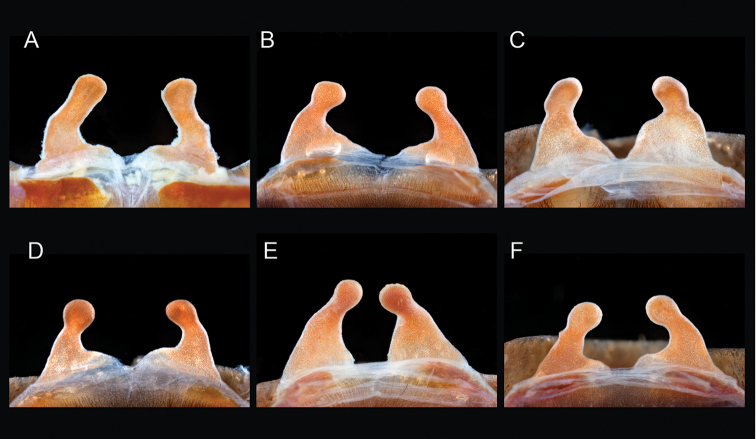
*Aphonopelma chalcodes* Chamberlin, 1940. **A–F** cleared spermathecae. **A** *chalcodes* allotype **B** APH_0608 **C** APH_0887 **D** APH_1485 **E** AUMS_2605 **F** AUMS_3282.

**Figure 34.**
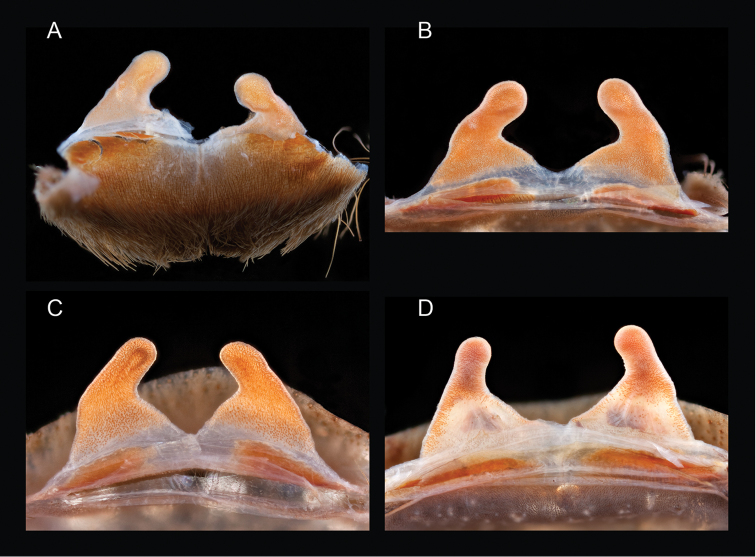
*Aphonopelma chalcodes* Chamberlin, 1940. **A–D** cleared spermathecae **A** APH_0168 **B** APH_0500 **C** AUMS_2339 **D** AUMS_2692.

**Figure 35.**
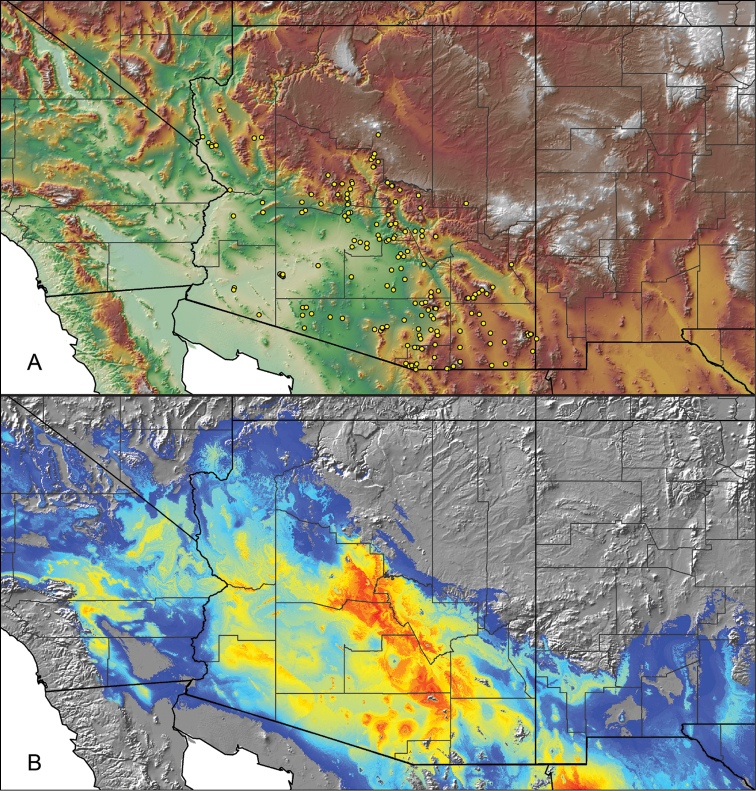
*Aphonopelma chalcodes* Chamberlin, 1940. **A** distribution of known specimens **B** predicted distribution; warmer colors (red, orange, yellow) represent areas of high probability of occurrence, cooler colors (blue shades) represent areas of low probability of occurrence.

Figures 30 , 31 , 32 , 33 , 34 , 35 ; Suppl. material 4

Aphonopelma chalcodes Chamberlin, 1940: 7; male holotype, male paratype, and two female paratypes from Tucson, Pima Co., Arizona, 32.221743 -110.926479^6^, elev. 2473ft., 27.vii.1936, coll. Prof. C.T. Vorhies; deposited in AMNH. [examined]
Rhechostica chalcodes Raven, 1985: 149.
Aphonopelma chalcodes Smith, 1995: 82.
Aphonopelma apacheum Chamberlin, 1940: 15; male holotype from Tucson, Pima Co., Arizona, 32.221743 -110.926479^6^, elev. 2473ft., elev. ft., no collecting date, coll. unknown; deposited in AMNH. Paratype male from the Santa Catalina Mountains, Pima Co., Arizona, 32.315500 -110.711168^5^, elev. 3800ft., 8-12.vii.1916, coll. Dr. F.E. Lutz; deposited in AMNH. [examined]
Rhechostica apacheum Raven, 1985: 149.
Aphonopelma apacheum Smith, 1995: 73. **syn. n.**
Aphonopelma minchi Smith, 1995: 121; male holotype from Usery Pass Rd., near Usery Mountain Regional Park, Maricopa Co., Arizona, 33.482543 -111.623178^4^, elev. 2033ft., no collecting date, coll. A. Smith and M. Sullivan; deposited in BMNH. Paratype male from western end of Apache Trail, 33.444378 -111.510491^5^, elev. 1937ft., no collecting date, coll. A. Smith and M. Sullivan; deposited in BMNH. [examined] **syn. n.**
Aphonopelma schmidti Smith, 1995: 140; male holotype and female paratype from Mineral Mountain, near Florence Junction on Hwy 60, Pinal Co., Arizona, 33.265044 -111.348427^5^, elev. 1916ft., 10.viii.1992, coll. A. Smith and M. Sullivan; deposited in BMNH. [examined] **syn. n.**
Aphonopelma stahnkei Smith 1995: 146; male holotype and male paratype from Tempe, Maricopa Co., Arizona, 33.425510 -111.940005^6^, elev. 1176ft., no collecting date, coll. Dr. H.L. Stahnke; deposited in BMNH. [examined] **syn. n.**

#### Diagnosis.

*Aphonopelma chalcodes* (Fig. 30) is a member of the *iodius* species group and can be identified by a combination of morphological, molecular, and geographic characteristics. Nuclear DNA identifies *Aphonopelma chalcodes* as a strongly supported lineage that is sister to the rest of the *iodius* species group (*Aphonopelma eutylenum*, *Aphonopelma iodius*, and *Aphonopelma johnnycashi* sp. n.) (Fig. 8). Aside from distribution, there are no pronounced measurements or characters that help discriminate *Aphonopelma chalcodes* from the phenotypically similar species in the *iodius* species group. Females and non-mature males of *Aphonopelma chalcodes* can easily be differentiated from *Aphonopelma catalina* sp. n., *Aphonopelma chiricahua* sp. n., *Aphonopelma madera* sp. n., *Aphonopelma marxi*, and *Aphonopelma vorhiesi* by possessing a more lightly colored carapace (the other species are generally black or dark brown). Males and females of *Aphonopelma chalcodes* can easily be differentiated from syntopic members of the *paloma* species group (*mareki* sp. n., *paloma*, *parvum* sp. n., *prenticei* sp. n., *saguaro* sp. n., and *superstitionense* sp. n.) by their greater extent of metatarsal scopulation on legs III and IV and their much larger size. The most significant measurements that distinguish male *Aphonopelma chalcodes* from its syntopic species are M3 and extent of metatarsus IV scopulation. Male *Aphonopelma chalcodes* can be distinguished by possessing a larger PTl/M3 (≥0.65; 0.65–0.76) than *Aphonopelma gabeli* (≤0.64; 0.58–0.64); by possessing a larger L4 scopulation extent (42%–74%) than *Aphonopelma catalina* (22%–36%), *Aphonopelma chiricahua* (33%–40%), and *Aphonopelma vorhiesi* (20%–36%); and by possessing a smaller F1/M3 (≤1.31; 1.13–1.31) than *Aphonopelma catalina* (≥1.57; 1.57–1.68), *Aphonopelma chiricahua* (≥1.40; 1.40–1.75), *Aphonopelma madera* (≥1.51; 1.51–1.69), *Aphonopelma marxi* (≥1.58; 1.58–1.87), and *Aphonopelma peloncillo* sp. n. (≥1.33; 1.33–1.49). The most significant measurement that distinguishes female *Aphonopelma chalcodes* from its syntopic species is extent of metatarsus IV scopulation. Female *Aphonopelma chalcodes* can be distinguished by possessing a larger L4 scopulation extent (56%–81%) than *Aphonopelma catalina* (37%–46%), *Aphonopelma chiricahua* (27% ± (only 1 specimen)), *Aphonopelma gabeli* (39%–53%), *Aphonopelma madera* (29%–35%), *Aphonopelma marxi* (33%–51%), *Aphonopelma peloncillo* (32%-39%), and *Aphonopelma vorhiesi* (29%–37%).

#### Redescription of male exemplar

(APH_0954; Fig. 31). *Specimen preparation and condition*: Specimen collected live crossing road, preserved in 80% ethanol; deposited in AUMNH; original coloration faded due to preservation. Left legs I, III, IV, and left pedipalp removed for measurements and photographs; stored in vial with specimen. Right leg III removed for DNA and stored at -80°C in the AUMNH (Auburn, AL). *General coloration*: Black and faded brown. *Cephalothorax*: Carapace 16.33 mm long, 15.71 mm wide; Hirsute; densely clothed with light brown iridescent pubescence mostly appressed to surface; fringe covered in long setae not closely appressed to surface; foveal groove medium deep and straight; pars cephalica region rises gradually from foveal groove, gently arching anteriorly toward ocular area; AER procurved, PER recurved; normal sized chelicerae; clypeus very slightly extends forward on a curve - mostly straight; LBl 2.15, LBw 2.76; sternum hirsute, clothed with shorter black/dark brown, densely packed setae. *Abdomen*: Densely clothed in short black/brown pubescence with numerous longer red/orange setae interspersed; possessing a dense dorsal patch of black Type I urticating bristles (Cooke et al. 1972). *Legs*: Hirsute, particularly ventrally; densely clothed in a mix of short/medium length black or faded black pubescence, femurs are darker. Metatarsus I slightly curved. F1 16.51; F1w 3.99; P1 7.12; T1 14.12; M1 13.37; A1 8.19; F3 13.73; F3w 4.15; P3 5.67; T3 10.59; M3 13.16; A3 7.95; F4 16.44; F4w 3.78; P4 6.16; T4 13.74; M4 16.84; A4 8.55; femur III is slightly swollen. All tarsi fully scopulate. Extent of metatarsal scopulation: leg III (SC3) = 81.3%; leg IV (SC4) = 76.4%. Three ventral spinose setae on metatarsus III; four ventral spinose setae on metatarsus IV. *Coxa I*: Prolateral surface a mix of fine, hair-like and tapered/thin tapered setae. *Pedipalps*: Hirsute; densely clothed in the same setal color as the other legs, with numerous longer ventral setae; one spinose seta on the apical, prolateral femur and three spinose setae on the prolateral tibia; PTl 9.54, PTw 3.15. When extended, embolus tapers and curves rapidly to the retrolateral side near apex; transition area from bulb to embolus is longer and wider than other species in the *iodius* group, no keels.

**Variation (13).** Cl 14.43–21.07 (16.98±0.51), Cw 13.23–18.32 (15.675±0.45), LBl 1.44–2.6 (2.07±0.08), LBw 1.69–2.91 (2.401±0.08), F1 14.56–19.13 (16.687±0.38), F1w 3.65–5.05 (4.119±0.12), P1 6.0–8.11 (6.852±0.16), T1 13.29–15.65 (14.136±0.19), M1 12.08–15.77 (13.313±0.3), A1 7.49–9.02 (8.219±0.14), L1 length 53.99–67.39 (59.208±1.09), F3 12.86–15.58 (14.193±0.25), F3w 3.97–5.5 (4.369±0.13), P3 5.08–6.86 (5.769±0.15), T3 10.26–12.33 (11.244±0.21), M3 12.46–14.84 (13.615±0.24), A3 7.16–9.09 (8.096±0.15), L3 length 48.04–58.45 (52.917±0.92), F4 15.25–18.59 (16.683±0.3), F4w 3.53–5.09 (4.012±0.12), P4 5.09–7.09 (6.138±0.15), T4 12.11–15.53 (13.848±0.28), M4 14.9–20.1 (17.587±0.39), A4 8.03–10.05 (8.951±0.17), L4 length 56.11–70.85 (63.208±1.17), PTl 8.835–11.026 (9.601±0.19), PTw 2.79–3.48 (3.065±0.06), SC3 ratio 0.651–0.86 (0.773±0.02), SC4 ratio 0.428–0.764 (0.647±0.03), Coxa I setae = tapered, F3 condition = normal/slightly swollen.

#### Redescription of female exemplar

(APH_0887; Figs 32–34). *Specimen preparation and condition*: Specimen collected live from burrow, preserved in 80% ethanol; deposited in AUMNH; original coloration faded due to preservation. Left legs I, III, IV, and pedipalp removed for photographs and measurements; stored in vial with specimen. Right leg III removed for DNA and stored at -80°C in the AUMNH (Auburn, AL). Genital plate with spermathecae removed and cleared, stored in vial with specimen. *General coloration*: Faded brown/black. *Cephalothorax*: Carapace 20.37 mm long, 18.10 mm wide; Hirsute, densely clothed with light brown pubescence closely appressed to surface; fringe densely covered in longer setae; foveal groove medium deep and straight; pars cephalica region gently rises from thoracic furrow, arching anteriorly toward ocular area; AER slightly procurved, PER slightly recurved; large chelicerae, clypeus mostly straight; LBl 2.45, LBw 3.01; sternum very hirsute, clothed with dark brown setae. *Abdomen*: Densely clothed dorsally in short black setae with numerous longer, lighter setae interspersed (generally red or orange *in situ*); dense dorsal patch of black Type I urticating bristles (Cooke et al. 1972); ventral side with shorter black/dark brown setae. *Spermathecae*: Paired and separate with wide bases, tapering and slightly curving medially towards capitate bulbs, with secondary medial bulges. *Legs*: Very hirsute, particularly ventrally; densely clothed in medium and long light brown pubescence, femurs much darker. F1 14.74; F1w 4.24; P1 6.40; T1 10.41; M1 8.93; A1 7.36; F3 12.95; F3w 4.27; P3 5.67; T3 9.23; M3 9.96; A3 7.32; F4 15.91; F4w 4.55; P4 6.66; T4 12.45; M4 13.79; A4 7.72. All tarsi fully scopulate. Extent of metatarsal scopulation: leg III (SC3) = 89.7%; leg IV (SC4) = 64.8%. Two ventral spinose setae on metatarsus III; six ventral spinose setae on metatarsus IV. *Coxa I*: Prolateral surface a mix of fine, hair-like and tapered/thin tapered setae. *Pedipalps*: Densely clothed in the same setal color as the other legs; one spinose seta on the apical, prolateral femur and six spinose setae on the prolateral tibia.

**Variation (10).** Cl 13.49-21.79 (18.548±0.72), Cw 11.72-19.71 (16.589±0.68), LBl 1.90-2.61 (2.375±0.07), LBw 2.21-3.02 (2.773±0.1), F1 11.39-16.37 (14.684±0.42), F1w 3.34–4.76 (4.397±0.14), P1 5.03–7.26 (6.554±0.21), T1 9.03–12.42 (11.224±0.31), M1 6.81–11.81 (9.35±0.41), A1 5.68–7.39 (7.012±0.16), L1 length 37.94–55.02 (48.824±1.42), F3 9.38–13.96 (12.093±0.38), F3w 3.15–4.58 (4.028±0.13), P3 3.92–6.25 (5.575±0.21), T3 6.78–9.67 (8.581±0.25), M3 7.83–11.6 (9.925±0.34), A3 5.89–7.32 (7.035±0.14), L3 length 33.8–48.7 (43.209±1.2), F4 12.15–16.3 (14.699±0.36), F4w 3.11–4.73 (4.134±0.14), P4 4.25–6.98 (6.134±0.24), T4 9.59–12.45 (11.334±0.25), M4 11.16–15.35 (13.498±0.37), A4 6.51–8.28 (7.676±0.15), L4 length 43.66–57.92 (53.341±1.24), SC3 ratio 0.728–0.926 (0.843±0.02), SC4 ratio 0.566–0.812 (0.682±0.03), Coxa I setae = medium tapered. Spermathecae variation can be seen in Figures 33–34.

#### Material examined.

**United States: Arizona: Cochise**: 0.6 miles E of Portal, 31.913718 -109.130089 ^4^, 4700ft., [AUMS_2676, 28/3/1990, 1♂, T.R. Prentice, AUMNH]; 1.4 miles NW Portal Rd on FR 42B (San Simon Rd); 31.926949 -109.169118 ^1^, 5126ft., [APH_1229, 7/8/10, 1♂, Brent E. Hendrixson, Ashley Bailey, Andrea Reed, AUMNH]; 10 miles east of Dos Cabezas, 31.93051 -109.794753 ^5^, 4281ft., [APH_2062, 4/8/71, 1♂, A. Jung, AMNH]; 2.4 miles NW Portal Rd on FR 42B (San Simon Rd); 31.932782 -109.183094 ^1^, 5293ft., [APH_1230, 7/8/10, 1♂, Brent E. Hendrixson, Ashley Bailey, Andrea Reed, AUMNH]; 2.6 miles NW Airport Rd on Muleshoe Ranch Rd, 32.259698 -110.103465 ^1^, 4967ft., [APH_0710, 19/7/2009, 1♂, Brent E. Hendrixson, Nate Davis, AUMNH]; 5 miles north of Douglas, 31.427339 -109.545202 ^5^, 4163ft., [APH_2069, 18/8/1964, 2♂, W.J. Gertsch, AMNH]; 5 miles south of Apache, 31.631987 -109.093064 ^5^, 4528ft., [APH_2074, 22/7/1964, 2♂, W.J. Gertsch and J.A. Woods, AMNH]; 5.1 miles NW AZ/NM state line on Portal Rd, 31.91264458 -109.1192604 ^2^, 4620ft., [APH_0386, 31/7/2008, 1♂, Alice Abela, AUMNH]; Airport Rd, W of Wilcox, 32.23908 -109.975427 ^1^, 4424ft., [APH_0709, 19/7/2009, 1♂, Brent E. Hendrixson, Nate Davis, AUMNH]; along Montezuma Canyon Rd, 31.385646 -110.406385 ^1^, 5650ft., [APH_1485, 2/8/12, 1♀, Brent E. Hendrixson, Brendon Barnes, Austin Deskewies, AUMNH]; along Ramsey Canyon Rd, 31.460005 -110.295399 ^1^, 5171ft., [APH_1225, 6/8/10, 1♂, Brent E. Hendrixson, Ashley Bailey, Andrea Reed, AUMNH]; Benson, 31.967008 -110.29502 ^5^, 3589ft., [APH_2579, 8/1/54, 1♂, George Bredt, AMNH]; Cochise Cemetery (along Hwy-191); 32.093827 -109.910145 ^1^, 4180ft., [APH_0619-621, 11/7/09, 2♀, 1 juv, Brent E. Hendrixson, Nate Davis, AUMNH]; Courtland Rd, near Jct. Ghost Town Trail, 31.758972 -109.796338 ^1^, 4596ft., [APH_0715, 20/7/2009, 1♀, Brent E. Hendrixson, Nate Davis, AUMNH]; Oro Rd, just NE of Hwy-80, 31.402388 -109.773026 ^1^, 4277ft., [APH_0686, 17/7/2009, 1 juv, Brent E. Hendrixson, Nate Davis, AUMNH]; Portal, 31.913703 -109.14145 ^5^, 4770ft., [APH_2057, 22/7/1976, 1♀, Mark Russel, AMNH]; [APH_2058, 1/9/64, 1♂, W.J. Gertsch, AMNH]; [APH_2060, 7/1/67, 1♂, W.J. Gertsch, AMNH]; [APH_2066, 27/8/1970, 1♀, 1♂, Chamberlin, AMNH]; [APH_2067-2068, 10/7/68, 2♂, W.J. Gertsch, AMNH]; [APH_2078, 8/1/65, 1♀, W.J. Gertsch, AMNH]; Rucker Canyon Rd, 31.784784 -109.557742 ^1^, 4575ft., [APH_0713-0714, 20/7/2009, 1♀, 1 juv, Brent E. Hendrixson, Nate Davis, AUMNH]; S of Sierra Vista at mouth of Ramsey Canyon, 31.463612 -110.291459 ^5^, 5095ft., [AUMS_2396, 14/7/1993, 1♂, V. Roth, AUMNH]; Sulphur Canyon, near Portal , 31.892604 -109.167082 ^5^, 5860ft., [APH_2059, 26/7/1955, 1♀, Guy Miller, AMNH]; Truman Rd, just off Hwy-82, 31.700137 -110.296948 ^1^, 4242ft., [APH_0687-0690, 17/7/2009, 1♀, 3 juv, Brent E. Hendrixson, Nate Davis, AUMNH]; **Coconino**: Flagstaff, 35.200524 -111.639452 ^6^, 6929ft., [APH_2586, unknown, 1♂, unknown, AMNH]; Mayer, just S of Prescott, 34.395374 -112.237457 ^5^, 4488ft., [AUMS_2390, 6/20/09, 1♂, Tom Roberts, AUMNH]; Schnebly Hill Road NE of Sedona, 34.887682 -111.703022 ^5^, 5810ft., [APH_2497, 6/8/77, 1♂, Barbara and M.W. Sanderson, AMNH]; [APH_2483, 6/8/77, 1♂, Barbara and M.W. Sanderson, AMNH]; **Gila**: 0.55 miles N Strawberry on Hwy-87, 34.41409752 -111.4912396 ^4^, 6046ft., [APH_0388, 2/8/08, 1♂, Alice Abela, AUMNH]; 0.75 miles E AZ-87 on Control Rd, 34.360317 -111.41347 ^1^, 5555ft., [APH_0505-0508, 20/5/2009, 2♀, 2 juv, Brent E. Hendrixson, Bernadette DeRussy, Sloan Click, AUMNH]; 1.1 miles NW AZ-87 on Barnhardt Trail Rd (FR 419); 34.09815 -111.36165 ^2^, 3200ft., [APH_0344, 10/5/08, 1 juv, Brent E. Hendrixson, Zach Valois, Josh Richards, AUMNH]; 1.4 miles E US-87 on Conrol Rd, 34.35949 -111.40302 ^2^, 5430ft., [APH_0346, 10/5/08, 1 juv, Brent E. Hendrixson, Zach Valois, Josh Richards, AUMNH]; 10 miles NW of Globe, 33.527502 -110.694934 ^5^, 4337ft., [APH_2064, 10/9/62, 1♂, Roth and Roth, AMNH]; 8 miles S of Young-dirt road Hwy 288, 33.997859 -110.953748 ^5^, 6170ft., [AUMS_2319, 6/8/92, 1♂, T.R. Prentice, AUMNH]; along Control Rd, east of Hwy-87, 34.3602 -111.415521 ^1^, 5855ft., [APH_1531-1532, 4/10/12, 1♀, 1 juv, Brent E. Hendrixson, AUMNH]; Along Houston-Mesa Rd, 34.29263192 -111.2868479 ^4^, 5168ft., [APH_0387, 2/8/08, 1♂, Alice Abela, AUMNH]; Apache Trail, 33.532562 -110.92045 ^6^, 3711ft., [APH_2456, 25/3/1936, 1♂, Edith M. Patel, AMNH]; Mazatzal Mountains, Tonto National Forest, El Oso Rd, 33.74139 -111.35831 ^1^, 5900ft., [APH_0191, 6/9/07, 1♀, Lorenzo Prendini, Jeremy Huff, AUMNH]; Payson, .41 miles E of Nf-200, 34.203644 -110.980578 ^1^, 5297ft., [APH_3127, 9/8/13, 1♂, Tyler P. McKee, AUMNH]; Salt River Canyon, 33.619952 -110.921017 ^5^, 2503ft., [APH_2065, 6/1/56, 1♂, W.D. Allison, AMNH]; Tonto National Forest, near jct. FR-449 off Hwy-188, 33.61465 -111.034975 ^1^, 2474ft., [APH_1301-1303, 26/7/2011, 3 juv, Brent E. Hendrixson, Brendon Barnes, Nate Davis, Jake Storms, AUMNH]; **Graham**: 11.2 miles W Hwy-191 on Hwy-266, 32.582263 -109.852113 ^1^, 5592ft., [APH_1235, 8/8/10, 1♀, Brent E. Hendrixson, Ashley Bailey, Andrea Reed, AUMNH]; 3.6 miles SW Hwy-191 along Swift Trail (Hwy-366); 32.6896 -109.754556 ^1^, 3950ft., [APH_1509, 7/9/12, 1♂, Brent E. Hendrixson, AUMNH]; along Ash Creek Rd, 32.51432 -110.077472 ^1^, 4450ft., [APH_1234, 8/8/10, 1♂, Brent E. Hendrixson, Ashley Bailey, Andrea Reed, AUMNH]; along High Creek Rd, 32.570316 -110.048141 ^1^, 4557ft., [APH_1232, 8/8/10, 1 juv, Brent E. Hendrixson, Ashley Bailey, Andrea Reed, AUMNH]; along Sunset Loop Rd, 32.512166 -110.159031 ^1^, 4747ft., [APH_1233, 8/8/10, 1♂, Brent E. Hendrixson, Ashley Bailey, Andrea Reed, AUMNH]; Bonita-Klondyke Rd, W of Bonita, 32.600171 -109.983213 ^1^, 4571ft., [APH_0695-0697, 18/7/2009, 3♀, Brent E. Hendrixson, Nate Davis, AUMNH]; crossing Sunset Rd, 32.506667 -110.180056 ^1^, 4711ft., [APH_0391, 24/7/2008, 1♂, Kari, Hunter McWest, AUMNH]; Lower Canyon of Mount Graham, 32.64023 -109.931671 ^6^, 5281ft., [APH_2071, 15/7/1956, 1♂, V. Roth and W.J. Gertsch, AMNH]; [APH_2583, 15/7/1956, 1♂, W. Gertsch and V. Roth, AMNH]; W Ash Creek Rd, 32.513833 -110.05858 ^1^, 4420ft., [APH_0392, 24/7/2008, 1♂, Kari, Hunter McWest, AUMNH]; **Greenlee**: 14 miles west of Clifton, 33.06165 -109.44632 ^5^, 3921ft., [APH_2493, 28/7/1962, 2♂, G. Bradt, AMNH]; **Groom**: Bradshaw Mountains near Prescott in pine forest, 34.385392 -112.368863 ^6^, 6440ft., [APH_2486, 15/9/1964, 1♂, Lowe, Soule, Wright, AMNH]; **La Paz**: 6.9 miles N US-60 on Alamo Rd (near Base of Harcuvar Mtns N of Wenden); 33.92479 -113.54419 ^1^, 2285ft., [APH_0340-0341, 9/5/08, 1♀, 1 juv, Brent E. Hendrixson, Zach Valois, AUMNH]; Alano Road, 34.08542 -113.5515 ^4^, 1982ft., [APH_0038, 12/8/06, 1♂, Alice Abela, AUMNH]; Bill Williams River NWA, E of Parker Dam (Buckskin Mtns); 34.29172 -114.08915 ^1^, 560ft., [APH_1003, 8/5/10, 1♀, Chris A. Hamilton, AUMNH]; S of Hwy-72 on Bouse-Quartzsite Rd, 33.86255 -114.02868 ^1^, 1130ft., [APH_0450, 17/11/2008, 1♀, June Olberding, AUMNH]; [APH_0451, 30/11/2008, 1♂, June Olberding, AUMNH]; **Maricopa**: Palomas Plain, 2.5 miles N of Sentinel, 32.886722 -113.238144 ^5^, 667ft., [AUMS_2604, 11/8/90, 1♂, T.R. Prentice, AUMNH]; Palomas Plain, 3 miles N of Sentinel, 32.90163 -113.212893 ^5^, 662ft., [AUMS_2647, 22/11/1992, 1♂, unknown, AUMNH]; Rio Verde, 1 mile S of Forest Rd and Rio Verde Dr. jct, off Forest Rd, 33.727441 -111.670456 ^4^, 1586ft., [AUMS_2673, 13/5/1991, 1♀, Chris Baptista, AUMNH]; Sentinel, 1.5 miles N of I-8, 32.879995 -113.223031 ^5^, 663ft., [AUMS_2620, 7/1/92, 1♂, unknown, AUMNH]; 1.35 miles N McDowell Rd on Usery Pass Rd, 33.480479 -111.624831 ^1^, 1989ft., [APH_1497, 5/9/12, 1 juv, Brent E. Hendrixson, AUMNH]; 3.0 miles N I-8 (Sentinel) on Agua Caliente Rd, 32.890757 -113.24223 ^1^, 656ft., [APH_0428, 16/11/2008, 1 juv, Brent E. Hendrixson, AUMNH]; 4.65 miles N I-8 (Sentinel) on Agua Caliente Rd, 32.90721 -113.262703 ^1^, 592ft., [APH_0429, 16/11/2008, 1 juv, Brent E. Hendrixson, AUMNH]; about 9-10 miles E AZ-87 on NF-143, 33.7285 -111.43288 ^4^, 3719ft., [APH_0134, 15/6/2007, 1♀, Austin Spears, AUMNH]; [APH_0135, 15/6/2007, 1 juv, Gilbert Quintana, AUMNH]; along Apache Trail, 33.606639 -111.19778 ^1^, 2372ft., [APH_1492-1496, 4/9/12, 4♂, 1 juv, Brent E. Hendrixson, AUMNH]; [APH_1502-1505, 6/9/12, 2♀, 2♂, Brent E. Hendrixson, AUMNH]; east of Tortilla Flat off of Hwy 88, 33.5336 -111.37225 ^1^, 1376ft., [APH_0954, 6/8/14, 1♂, Ben Allen, AUMNH]; in the Tonto National Forest, off Road 78 that splits from Apache Trail (88); E of Apache Junction, 33.4701 -111.47296 ^1^, 2082ft., [APH_3199, 15/11/2013, 1 juv, Chris A. Hamilton, Brent E. Hendrixson, AUMNH]; Mazatzal Mountains, Four Peaks, Cline Cabin Rd./F.R. 143, 33.71667 -111.45509 ^4^, 3080ft., [APH_0298, 6/10/07, 1 juv, Zach Valois, AUMNH]; Mesa, 33.414255 -111.831101 ^5^, 1220ft., [APH_2591, unknown, 1♂, Michael Soleglad, AMNH]; [APH_2595, unknown, 1♂, Michael Soleglad, AMNH]; N of Gila Bend along Hwy-85, 0.6 miles E of Hwy, 33.043715 -112.633476 ^1^, 807ft., [APH_0419, 15/11/2008, 1 juv, Brent E. Hendrixson, AUMNH]; N side of Cline Cabin Rd, 33.72875 -111.43244 ^4^, 3750ft., [APH_0360, 17/4/2008, 1♀, Zach Valois, AUMNH]; Palomas Plain, Sentinel, 32.861687 -113.21082 ^5^, 683ft., [AUMS_2323, 18/7/1989, 1♂, T.R. Prentice, AUMNH]; Phoenix, Usery Pass Rd off N Bush Hwy, 33.5468 -111.645 ^1^, 1346ft., [APH_0887, 6/8/14, 1♀, Ben Allen, AUMNH]; Phoenix, 33.460362 -112.039455 ^6^, 1119ft., [APH_2075, unknown, 1♀, unknown, AMNH]; South Mountain Park, 33.35257 -112.072563 ^6^, 1332ft., [APH_2501, 21/11/1965, 1♂, S. C. Williams, AMNH]; South of Geronimo Head Mountain, 33.498275 -111.387253 ^6^, 2933ft., [APH_2070, 21/12/1963, 1♂, Chamberlin, AMNH]; Sunflower, Sycamore Creek Rd., 33.88512 -111.47874 ^4^, 3526ft., [APH_0300-301, 6/10/07, 2 juv, Zach Valois, AUMNH]; E/NE of Pyramid Peak, E of New River Rd., 33.921335 -112.101997 ^5^, 2184ft., [AUMS_2631, 18/11/2003, 1♂, C. Shipley, AUMNH]; New River, 33.915059 -112.136776 ^5^, 2022ft., [AUMS_2687, 8/1/98, 1♂, Tom Roberts, AUMNH]; 6 miles N AZ-74 on New River Rd (near Lake Pleasant); 33.86502 -112.18967 ^1^, 1799ft., [APH_0007, 5/10/01, 1♂, Brent E. Hendrixson, Darrin Vernier, AUMNH]; [APH_0046, 5/10/01, 1 juv, Brent E. Hendrixson, Darrin Vernier, AUMNH]; 6.0 miles N AZ-74 on New River Rd (near Lake Pleasant); 33.86307 -112.19106 ^1^, 1773ft., [APH_0127-0130, 13/6/2007, 1♀, 1♂, 2 juv, Brent E. Hendrixson, AUMNH]; [APH_0142-0143, 13/6/2007, 1♀, 1 juv, Brent E. Hendrixson, AUMNH]; 6.6 miles W jct. US-93 on US-60, 33.956369 -112.840469 ^1^, 2582ft., [APH_0498-0501, 18/5/2009, 1♀, 3 juv, Brent E. Hendrixson, Bernadette DeRussy, Sloan Click, AUMNH]; New River, ~0.5 miles W I-17 on AZ-74, 33.79765 -112.14307 ^4^, 1655ft., [APH_0164-0165, unknown, 1♂, 1♀, Brandon Anderson, AUMNH]; Wickenburg, about 2.84 miles south of Interstate 60, 33.922158 -112.903511 ^1^, 2700ft., [APH_3128, 1/9/13, 1♀, Tyler P. McKee, AUMNH]; **Maricopa/Yavapai**: between Prescott and Phoenix, 34.052118 -112.147482 ^7^, 2047ft., [APH_2076, 31/12/1933, 1♂, McCanly, AMNH]; **Mohave**: 0.9 miles S I-40 on Hwy-93, 35.14879 -113.689479 ^1^, 3646ft., [APH_1221-1222, 2/8/10, 2 juv, Brent E. Hendrixson, Brendon Barnes, Nate Davis, AUMNH]; 7.4 miles NE Hwy-93 on CR-25 in Dolan Springs, 35.60418 -114.253563 ^1^, 3638ft., [APH_1309, 28/7/2011, 1 juv, Brent E. Hendrixson, Brendon Barnes, Nate Davis, Jake Storms, AUMNH]; Bullhead City, ~2 miles S Hwy-68, 35.16973 -114.53414 ^4^, 1003ft., [APH_0048, 22/8/2001, 1♀, Tom and Tracy Vezie, AUMNH]; Cool Spring on Oatman Rd, off Rt. 66 (Black Mtns); 35.02957 -114.31629 ^1^, 2772ft., [APH_0999-1001, 6/5/10, 3♀, Chris A. Hamilton, Rick West, AUMNH]; just N I-40 on Silver Springs Rd, 35.161716 -113.564676 ^1^, 3927ft., [APH_1305, 28/7/2011, 1♂, Brent E. Hendrixson, Brendon Barnes, Nate Davis, Jake Storms, AUMNH]; NW of Oatman on Rt. 10, 35.07184 -114.43861 ^1^, 2191ft., [APH_0310-0312, 11/10/07, 3♂, Rick C. West, AUMNH]; Oatman Rd., Black Mountains, 35.009671 -114.389097 ^6^, 2420ft., [AUMS_2682, 27/3/1988, 1♀, T.R. Prentice, AUMNH]; Oatman, N of town off Rt. 66 (Black Mtns); 35.03484 -114.39156 ^1^, 2772ft., [APH_1002, 7/5/10, 1♀, Chris A. Hamilton, AUMNH]; **Navajo**: Hwy 60 (77); N of Carrizo, mile marker 324, 34.070957 -110.193557 ^5^, 5609ft., [AUMS_2477, 31/8/1986, 1♂, T.R. Prentice, AUMNH]; **Pima**: 0.18 miles S Hwy-86 on Hwy-386, 32.02419162 -111.5769608 ^2^, 3251ft., [APH_0739, 23/8/2009, 1♂, Alice Abela, AUMNH]; 0.2 miles W SR-83 on Sahuarita Rd, 31.963159 -110.675585 ^1^, 3679ft., [APH_0616, 9/7/09, 1♂, Brent E. Hendrixson, Jon Davenport, Nate Davis, AUMNH]; 0.75 miles S Ball Rd in Why along Hwy-85, 32.252063 -112.7449 ^1^, 1787ft., [APH_0425, 15/11/2008, 1 juv, Brent E. Hendrixson, AUMNH]; 1.02 miles S Hwy-86 on Hwy-386, 32.01220214 -111.5747721 ^2^, 3372ft., [APH_0738, 23/8/2009, 1♂, Alice Abela, AUMNH]; 1.75 miles N Sahuarita Rd on Houghton Rd, 31.987937 -110.772369 ^1^, 3201ft., [APH_0617, 9/7/09, 1♂, Brent E. Hendrixson, Jon Davenport, Nate Davis, AUMNH]; 10560 E Wildfire Drive, Tucson, 32.2213 -110.76858 ^2^, 2757ft., [APH_0014, 8/2/14, 1♂, Alex Binford, AUMNH]; 2 miles N AZ-86 (W Ajo Hwy) on Sandario Rd, near jct. with Dusty Mesquite Trail, 32.14446246 -111.2177859 ^2^, 2350ft., [APH_0370, 24/7/2008, 1♂, Alice Abela, AUMNH]; 5 miles west on St.289 from Jef.289 and US 89- north of Nogales, 31.426474 -111.008278 ^5^, 3665ft., [APH_2526, 20/7/1966, 1♂, E. Brown and J. Cole, AMNH]; along Hwy-86, 32.1992 -112.43633 ^5^, 1980ft., [APH_0174, 8/7/14, 1♂, Alice Abela, AUMNH]; [APH_0176, 8/7/14, 1♂, Alice Abela, AUMNH]; [APH_0740-0742, 23/8/2009, 3♂, Alice Abela, AUMNH]; along Hwy-86, W of San Pedro, 32.03889549 -111.5014244 ^4^, 2943ft., [APH_0737, 22/8/2009, 1♂, Alice Abela, AUMNH]; along Kitt Peak Rd, 31.97428845 -111.604144 ^4^, 4625ft., [APH_0371-0372, 24/7/2008, 2♂, Alice Abela, AUMNH]; [APH_0374-0375, 25/7/2008, 2♂, Alice Abela, AUMNH]; along Madera Canyon Rd, 31.836971 -110.946796 ^1^, 3083ft., [APH_1344-1345, 4/8/11, 2♀, Brent E. Hendrixson, Brendon Barnes, Nate Davis, Jake Storms, AUMNH]; along SR-83, 31.896859 -110.665638 ^1^, 4045ft., [APH_0611-0615, 9/7/09, 5♂, Brent E. Hendrixson, Jon Davenport, Nate Davis, AUMNH]; AZ-86, 0.06 miles SW jct Indian Rt 30, 31.99315318 -111.7053503 ^2^, 2924ft., [APH_0373, 25/7/2008, 1♂, Alice Abela, AUMNH]; Batamote Rd, 31.729183 -111.161558 ^1^, 3387ft., [APH_0610, 9/7/09, 1 juv, Brent E. Hendrixson, Jon Davenport, Nate Davis, AUMNH]; Bates Well Rd., 2 miles N of OPCN monument (Organ Pipe Cactus National Monument); 32.226852 -112.890815 ^5^, 1545ft., [AUMS_3329, 10/9/90, 1♂, W. Icenogle and G. Lowe, AUMNH]; Catalina Mountains, Bug Spring Trail, 32.341135 -110.714921 ^1^, 3624ft., [APH_0603-0604, 9/7/09, 1♀, 1 juv, Brent E. Hendrixson, Jon Davenport, Nate Davis, Paul Marek, Charity Hall, AUMNH]; Catalina State Park, campground, 32.4238 -110.9233 ^2^, 2700ft., [APH_1487, 2/8/12, 1♂, Brent E. Hendrixson, Brendon Barnes, Austin Deskewies, AUMNH]; Catalina State Park, trailhead parking lot and nature trail, 32.425002 -110.909336 ^1^, 2635ft., [APH_0598-0600, 8/7/09, 1♀, 2♂, Brent E. Hendrixson, Jon Davenport, Nate Davis, AUMNH]; [APH_0602, 8/7/09, 1♂, Brent E. Hendrixson, Jon Davenport, Nate Davis, AUMNH]; Fish Canyon Rd, 31.742778 -110.707871 ^1^, 4888ft., [APH_0691-0693, 17/7/2009, 1♀, 2 juv, Brent E. Hendrixson, Nate Davis, AUMNH]; NFWL Campsite, Cabeza Prieta Game Refuge, 32.232313 -113.608052 ^5^, 932ft., [APH_2585, 7/1/76, 1♀, B. Woodward and R. Jones, AMNH]; Organ Pipe National Monument, 32.016369 -112.858922 ^5^, 1988ft., [APH_2498, 5/22/09, 1♂, Vogt, AMNH]; Quail Creek-Veterans Municipal Park, 31.898272 -110.964712 ^1^, 2794ft., [APH_0605-0609, 9/7/09, 5♀, Brent E. Hendrixson, Jon Davenport, Nate Davis, AUMNH]; Redington, 32.42157 -110.5037 ^5^, 2890ft., [APH_2072, 25/5/1970, 1♀, Mary Smallhouse, AMNH]; Sabino Canyon, Tucson, 32.315852 -110.81935 ^5^, 2782ft., [APH_2474, 11/9/35, 1♂, W.J. Baerg, AMNH]; [APH_2588, 9/1/35, 1♂, unknown, AMNH]; Saguaro National Park, eastern portion of park, off Cactus Forest Loop Dr. at Cactus Forest south hiking trail, 32.171204 -110.730429 ^1^, 3061ft., [APH_3174-3175, 12/11/13, 1♂, 1 juv, Chris A. Hamilton, Brent E. Hendrixson, AUMNH]; San Vicente, 33.336256 -111.831893 ^5^, 1220ft., [APH_2061, 19/8/1957, 2♂, V. Roth, AMNH]; Santa Catalina Mountains, 32.44193 -110.860732 ^6^, 3806ft., [APH_2073, 8/7/16, 1♂, T.E. Luty, AMNH]; Santa Catalina Mountains, Catalina Hwy, 32.337262 -110.6959 ^5^, 4501ft., [AUMS_3282, 26/8/1989, 1♂, T.R. Prentice, AUMNH]; SE of Ajo, mile marker 46, 32.357132 -112.826569 ^5^, 1710ft., [AUMS_2605, 27/8/1989, 1♀, T.R. Prentice, AUMNH]; SW of Ajo, Little Ajo Mountain, Darby Well Road, 32.35451 -112.886495 ^4^, 2090ft., [APH_0309, 15/10/2005, 1♂, Lennart Pettersson, Dave Kandiyeli, Sheri Monk, AUMNH]; Tucson, 32.221743 -110.926479 ^6^, 2470ft., [APH_2088, 27/7/1936, 1♀, 1♂, unknown, AMNH]; [APH_2089, 7/1/35, 1♀, P. Stichler, AMNH]; [APH_2063, 23/8/1973, 1♂, A. Jung, AMNH]; [APH_2205, 9/1/35, 1♂, W.J. Baerg, AMNH]; Tucson area, near Catalina State Park, N side of Tangerine Rd and E of Tangerine Crossing, E of Marana, 32.424545 -111.03393 ^1^, 2731ft., [APH_3173, 12/11/13, 1 juv, Brent E. Hendrixson, Chris A. Hamilton, AUMNH]; Ajo, SE on Hwy 85, 32.35653 -112.826776 ^5^, 1711ft., [AUMS_2384, 27/8/1989, 1♀, T.R. Prentice, AUMNH]; near Sahuarita off I19 (Hwy 89); 32.002043 -110.981937 ^5^, 2702ft., [AUMS_2324, 15/10/1992, 1♂, Barney Tomberlin, AUMNH]; Papago Indian Reservation, Hwy 86, 7.2 miles W of Indian Rte 15, 32.176101 -112.232872 ^4^, 2418ft., [AUMS_2382, 18/11/1989, 1♂, T.R. Prentice, AUMNH]; **Pinal**: 0.1 miles S Apache Trail on Mountain View Rd, 33.445508 -111.502834 ^1^, 1935ft., [APH_0503, 19/5/2009, 1 juv, Brent E. Hendrixson, Bernadette DeRussy, Sloan Click, AUMNH]; 1.5 miles S US-60 on Mineral Mountain Rd, 33.242909 -111.270678 ^1^, 2067ft., [APH_0502, 19/5/2009, 1 juv, Brent E. Hendrixson, Bernadette DeRussy, Sloan Click, AUMNH]; 2.4 miles E Hwy-79 on Cottonwood Canyon Rd, 33.187604 -111.313416 ^1^, 1901ft., [APH_1304, 26/7/2011, 1 juv, Brent E. Hendrixson, Brendon Barnes, Nate Davis, Jake Storms, AUMNH]; 3.4 miles SE AZ-77 on SR-76, near San Manuel, 32.628709 -110.648951 ^1^, 3392ft., [APH_0596, 8/7/09, 1♀, Brent E. Hendrixson, Jon Davenport, Nate Davis, AUMNH]; 7 miles south of Oracle-Santa Catalina Mountains, 32.531565 -110.773332 ^5^, 4678ft., [APH_2529, 25/7/1949, 2♂, unknown, AMNH]; in desert NW jct of Hwy-8 4and Amarillo Valley Rd (3.2 miles NW I-8); 32.856009 -112.085251 ^1^, 1517ft., [APH_0420, 15/11/2008, 1♀, Brent E. Hendrixson, Paul Marek, Charity Hall, Kojun, AUMNH]; near Cogburn Ostrich Ranch, 32.64396 -111.39235 ^4^, 1840ft., [APH_0369, 5/4/08, 1 juv, Shasta Michaels, AUMNH]; off Florence-Kelvin Hwy, BLM land E of Florence, 32.99965 -111.26476 ^1^, 1970ft., [APH_3196, 15/11/2013, 1 juv, Chris A. Hamilton, Brent E. Hendrixson, AUMNH]; off Hwy 79 around 1/2 way between 77 split and Florence at Tom Mix Rest Area, 32.82084 -111.20621 ^1^, 2333ft., [APH_3192, 15/11/2013, 1 juv, Chris A. Hamilton, Brent E. Hendrixson, AUMNH]; off Hwy 79 around 1/2 way between 77 split and Florence at Tom Mix Rest Area, 32.820504 -111.20632 ^1^, 2370ft., [APH_3193, 15/11/2013, 1 juv, Brent E. Hendrixson, Chris A. Hamilton, AUMNH]; OHV trail area, near Kearny, 33.065521 -110.888678 ^1^, 2163ft., [APH_0595, 8/7/09, 1♀, Brent E. Hendrixson, Jon Davenport, Nate Davis, AUMNH]; Oracle, 32.610602 -110.771115 ^5^, 4537ft., [APH_2593, 11/7/40, 1♂, Gertsch and Hook, AMNH]; Picacho, just off Camino Adelante Dr, 32.71453 -111.4975 ^2^, 1612ft., [APH_0716, 7/9/14, 1♀, Warren Burke, AUMNH]; Robert Engstrom’s property, 0.2 miles S East Cactus Forest Rd on North Coolidge Airport Rd, 32.97029 -111.42175 ^2^, 1541ft., [APH_0198, 14/6/2007, 1♂, Robert Engstrom, AUMNH]; Superior, Boyce Thompson Arboretum, 33.279265 -111.160952 ^2^, 2440ft., [APH_1370, 3/9/11, 1♂, Benjamin Curtis, Mason McWest, AUMNH]; W of Chuck’s Corner off S. Amarillo Valley Rd, off Hwy 84, N of I-8, 32.8572 -112.08641 ^1^, 1517ft., [APH_3167, 11/11/13, 1♂, Chris A. Hamilton, Brent E. Hendrixson, AUMNH]; [APH_3169, 11/11/13, 1 juv, Brent E. Hendrixson, Chris A. Hamilton, AUMNH]; **Santa Cruz**: 0.65 miles W Mount Hopkins Rd on Forest Service Rd 184, 31.69646 -111.00164 ^2^, 3512ft., [APH_0175, 8/7/14, 1♂, Alice Abela, AUMNH]; 0.95 miles W Mount Hopkins Rd on Forest Service Rd 184, 31.69671 -111.00641 ^2^, 3497ft., [APH_0173, 8/7/14, 1♂, Alice Abela, AUMNH]; along Duquesne Rd, 31.363711 -110.792456 ^1^, 4146ft., [APH_1340, 4/8/11, 1♀, Brent E. Hendrixson, Brendon Barnes, Nate Davis, Jake Storms, AUMNH]; along Duquesne Rd (FR-61]; 31.347952 -110.509098 ^1^, 5057ft., [APH_1339, 4/8/11, 1♀, Brent E. Hendrixson, Brendon Barnes, Nate Davis, Jake Storms, AUMNH]; along FR-61, 31.34832 -110.508551 ^1^, 5088ft., [APH_1224, 6/8/10, 1♀, Brent E. Hendrixson, Ashley Bailey, Andrea Reed, AUMNH]; along Montosa Canyon Rd (FR-184); 31.701063 -111.039066 ^1^, 3181ft., [APH_1341, 4/8/11, 1♀, Brent E. Hendrixson, Brendon Barnes, Nate Davis, Jake Storms, AUMNH]; Along Mt Hopkins Rd, 31.67354048 -110.9229983 ^4^, 5047ft., [APH_0383-0384, 28/7/2008, 2♂, Alice Abela, AUMNH]; [APH_0730, 20/8/2009, 1♂, Alice Abela, AUMNH]; Along Ruby Rd, 31.43198526 -111.188291 ^4^, 4018ft., [APH_0376, 26/7/2008, 1♂, Alice Abela, AUMNH]; [APH_0377-0379, 27/7/2008, 3♂, Alice Abela, AUMNH]; [APH_0380-0381, 28/7/2008, 2♂, Alice Abela, AUMNH]; Coronado National Forest, along FR-61, 31.343749 -110.485365 ^1^, 5083ft., [APH_1195-1196, 28/7/2010, 2♀, Brent E. Hendrixson, Brendon Barnes, Nate Davis, AUMNH]; Coronado National Forest, Pena Blanca Lake, Hwy 289, 31.39225 -111.13153 ^1^, 4290ft., [APH_0193, 5/9/07, 1♂, Lorenzo Prendini, Jeremy Huff, AUMNH]; Harshaw Rd, 31.531637 -110.718517 ^4^, 4187ft., [APH_0389, unknown, 1♂, Alice Abela, AUMNH]; [APH_0694, 17/7/2009, 1 juv, Brent E. Hendrixson, Nate Davis, AUMNH]; Oro Blanco Mountains-12 miles from Nogales, 31.333796 -111.023962 ^5^, 4472ft., [APH_2492, 7/1/37, 3♂, Steckler, AMNH]; Ruby Road, 31.3948 -111.13614 ^4^, 4487ft., [APH_0037, 6/8/06, 1♂, Alice Abela, AUMNH]; Ruby Road near Pena Lake, 31.400702 -111.090398 ^5^, 3871ft., [APH_2487, 19/7/1962, 1♂, unknown, AMNH]; Ruby Road-10 miles northwest of Nogales, 31.402097 -111.027724 ^5^, 3865ft., [APH_2505, 8/1/62, 1♂, W.J. Gerstch, AMNH]; **Yavapai**: 0.2 miles E I-17 on Dugas Rd, 34.39945 -112.07044 ^1^, 3800ft., [APH_0131-0132, 13/6/2007, 1♂, 1 juv, Brent E. Hendrixson, AUMNH]; 0.8 miles E I-17, 0.9 miles S Stoneman Lake Road [near Exit 306]; 34.76055 -111.64488 ^1^, 5472ft., [APH_0049, 3/10/01, 1 juv, Brent E. Hendrixson, Darrin Vernier, AUMNH]; 3.4 miles SE AZ-71 on US-93, 34.09027 -112.90285 ^1^, 2717ft., [APH_0133, 15/6/2007, 1 juv, Brent E. Hendrixson, Shasta Michaels, AUMNH]; Beaver Creek Trail at trailhead, 40.4 miles S of jct I-40 and I-17 on 17, 34.674376 -111.713738 ^5^, 3875ft., [AUMS_2339, 31/3/1990, 1♂, T.R. Prentice, AUMNH]; Beaver Creek, E of I-17, E of interstate, N of Hwy 260, 34.671522 -111.724763 ^5^, 3920ft., [AUMS_3264, 31/3/1990, 1♀, T.R. Prentice, AUMNH]; Black Canyon City, 0.85 miles E I-17 on South Mud Springs Rd, 34.073 -112.13204 ^4^, 2044ft., [APH_0168-0170, unknown, 1♀, 2♂, Brandon Anderson, AUMNH]; Bumble Bee Ranch Rd. (Bumble Bee, AZ Ghost Town off Co. Rd. 59); 34.152931 -112.156127 ^5^, 2640ft., [AUMS_2325, 10/8/90, 1♂, T.R. Prentice, AUMNH]; Bumblebee Ranch Rd (Crown King Rd); 1.5 miles E of Crown King, 34.217448 -112.307279 ^5^, 5477ft., [AUMS_2692, 30/8/1990, 2♂, T.R. Prentice, AUMNH]; Bumblebee Ranch Road, 34.227934 -112.154007 ^5^, 2800ft., [AUMS_2383, 30/8/1990, 1♂, T.R. Prentice, AUMNH]; [AUMS_2320, 11/8/90, 1♂, T.R. Prentice, AUMNH]; [AUMS_2338, 12/8/90, 1♀, T.R. Prentice, AUMNH]; [AUMS_3272, 16/8/1994, 1♀, T.R. Prentice, AUMNH]; [AUMS_3324, 11/8/90, 1♂, T.R. Prentice, AUMNH]; [AUMS_3339, 10/8/90, 1♂, T.R. Prentice, AUMNH]; [AUMS_2379, 16/8/1994, 1♂, Enza Prentice, AUMNH]; Constellation, 34.064896 -112.585608 ^5^, 3446ft., [AUMS_2366, 8/1/91, 1♂, T.R. Prentice, AUMNH]; Cordes Lake Development, Cordes jct, SE of Mayer, 34.307807 -112.103491 ^5^, 3701ft., [AUMS_3342, 21/8/1998, 1♂, Stephen Roy, AUMNH]; Dugas Road, approx. 2.0 miles W I-17 on Orme Ranch Road, 34.42758 -112.07979 ^4^, 3914ft., [APH_0013, 24/3/2002, 1♂, John Bell, AUMNH]; N of Village of Oak Creek, Courthouse Butte/Big Park Trail, 34.79569 -111.758817 ^1^, 4233ft., [APH_1543, 22/10/2012, 1 juv, Brent E. Hendrixson, AUMNH]; near Deer Pass Crossing on Oak Creek about 8 miles southeast Sedona, 34.838954 -111.721245 ^5^, 6411ft., [APH_2485, 15/6/1977, 1♂, M.W. Sanderson, AMNH]; Prescott, 34.53923 -112.468695 ^5^, 5387ft., [APH_2504, 28/7/1948, 1♂, C. and P. Vaurie, AMNH]; Yarnell, 34.221331 -112.747488 ^5^, 4806ft., [APH_2587, 4/9/61, 1♂, Roth and Roth, AMNH]; **Yuma**: Rd. 36 E off I-8, S side of I-8, E field, 32.679615 -114.015636 ^5^, 358ft., [AUMS_2362, 12/8/90, 1♂, T.R. Prentice, AUMNH]; Rd. 36 E, south, W side of rd., 32.645663 -114.02637 ^5^, 449ft., [AUMS_2363, 16/11/1986, 1♂, T.R. Prentice, AUMNH]; **New Mexico: Hidalgo**: Rodeo, 31.835372 -109.03117 ^5^, 4131ft., [APH_2090, 20/1/1969, 1♀, V. Roth, AMNH].

#### Distribution and natural history.

*Aphonopelma chalcodes* is widely distributed across the southern two-thirds of Arizona south of the Grand Canyon, bound to the west by the Colorado River and barely making its way into southwestern New Mexico (Fig. 35). The species is undoubtedly widespread throughout northern Sonora, Mexico as well. This species inhabits a large diversity of habitats including the following Level III Ecoregions: Arizona/New Mexico Mountains, Arizona/New Mexico Plateau, Madrean Archipelago, and the Sonoran Basin and Range (Fig. 1G). *Aphonopelma chalcodes* can be found in syntopy with a number of species across its distribution including: *Aphonopelma catalina* , *Aphonopelma chiricahua* , *Aphonopelma gabeli*, *Aphonopelma madera* , *Aphonopelma mareki* , *Aphonopelma marxi*, *Aphonopelma paloma*, *Aphonopelma parvum* , *Aphonopelma peloncillo*, *Aphonopelma prenticei* , *Aphonopelma saguaro* , *Aphonopelma superstitionense* , and *Aphonopelma vorhiesi*. The breeding season, when mature males abandon their burrows in search of females, occurs during the summer (generally July–September), particularly during the summer monsoon season. Additional information about the natural history of this species can be found in Smith (1995).

#### Conservation status.

*Aphonopelma chalcodes* is the most widespread and abundant tarantula species in Arizona. The species is secure.

#### Remarks.

*Aphonopelma chalcodes* is herein considered a member of the problematic *iodius* species group. Morphological and molecular data confirm that *Aphonopelma chalcodes* is the sister lineage to the remaining species in the group (*Aphonopelma iodius*, *Aphonopelma eutylenum*, and *Aphonopelma johnnycashi*). There are no major morphological features that can be used to distinguish *Aphonopelma chalcodes* from these species so we must rely on molecular data and distributional information (*Aphonopelma chalcodes* is largely restricted to Arizona south of the Grand Canyon). Other important ratios that distinguish males: *Aphonopelma chalcodes* possess a larger M1/A1 (≥1.51; 1.51–1.79) than *Aphonopelma johnnycashi* (≤1.43; 1.29–1.43). Other important ratios that distinguish females: *Aphonopelma chalcodes* possess a smaller F1/M1 (≤1.68; 1.38–1.68) than *Aphonopelma catalina* (≥1.71; 1.71–1.83), *Aphonopelma chiricahua* (1.84 ± (only 1 specimen)), *Aphonopelma madera* (≥1.73; 1.73; 1.73–2.15), and *Aphonopelma marxi* (≥1.77; 1.77–1.88); by possessing a smaller P1/M1 (≤0.75; 0.61–0.75) than *Aphonopelma vorhiesi* (≥0.75; 0.75–0.85). For both males and females, certain morphometrics have potential to be useful, though due to the amounts of variation, small number of specimens, and the small differences between species, no others are claimed to be significant at this time (see Suppl. material 2). During evaluation of traditional two-dimensional PCA morphospace and three-dimensional PCA morphospace (PC1~PC2~PC3), male and female *Aphonopelma chalcodes* do not separate from the other species in the *iodius* species group. Male *Aphonopelma chalcodes* separate from syntopic species *Aphonopelma catalina*, *Aphonopelma chiricahua*, *Aphonopelma madera*, and *Aphonopelma marxi* in two-dimensional morphospace as well as all syntopic miniature tarantulas (*Aphonopelma mareki*, *Aphonopelma paloma*, *Aphonopelma parvum*, *Aphonopelma prenticei*, *Aphonopelma saguaro*, and *Aphonopelma superstitionense*), but do not separate from *Aphonopelma gabeli* or *Aphonopelma peloncillo*; when three-dimensional morphospace is evaluated, *Aphonopelma chalcodes* separates from all of their syntopic species. Female *Aphonopelma chalcodes* separate from their syntopic species *Aphonopelma chiricahua*, *Aphonopelma madera*, and *Aphonopelma marxi* in traditional two-dimensional morphospace as well as all syntopic miniature tarantulas, but do not separate from *Aphonopelma catalina*, *Aphonopelma gabeli*, *Aphonopelma peloncillo* or *Aphonopelma vorhiesi*; when three-dimensional morphospace is evaluated, *Aphonopelma chalcodes* separates from *Aphonopelma chiricahua*, *Aphonopelma madera*, *Aphonopelma marxi*, *Aphonopelma peloncillo*, and *Aphonopelma vorhiesi*, but not *Aphonopelma catalina* or *Aphonopelma gabeli*. PC1, PC2, and PC3 explain ≥95% of the variation in all analyses. We examined the holotypes and freshly collected topotypic material of *Aphonopelma apacheum*, *Aphonopelma minchi*, *Aphonopelma schmidti*, and *Aphonopelma stahnkei*. Our morphological and molecular analyses fail to recognize these four species as separate, independently evolving lineages. As a consequence, we consider *Aphonopelma apacheum*, *Aphonopelma minchi*, *Aphonopelma schmidti*, and *Aphonopelma stahnkei* junior synonyms of *Aphonopelma chalcodes*.

Mitochondrial DNA (*CO1*) identifies *Aphonopelma chalcodes* as a polyphyletic group with some samples more closely related to specimens of *Aphonopelma iodius* (Fig. 7); these latter samples of *Aphonopelma chalcodes* were previously considered a putative cryptic species by Hamilton et al. (2014). The mtDNA also identifies *Aphonopelma vorhiesi* as the sister lineage to *Aphonopelma chalcodes*. The AE nuclear DNA on the other hand shows that *Aphonopelma chalcodes* is a single lineage that is sister to the rest of the *iodius* species group. Again, these results highlight how *CO1* is not effective at accurately delimiting species boundaries within this group.

### Aphonopelma chiricahua

Taxon classificationAnimaliaAraneaeTheraphosidae

Hamilton, Hendrixson & Bond sp. n.

http://zoobank.org/4E7A530F-C6B5-4E19-908B-81BB4F14E4A2

**Figure 36.**
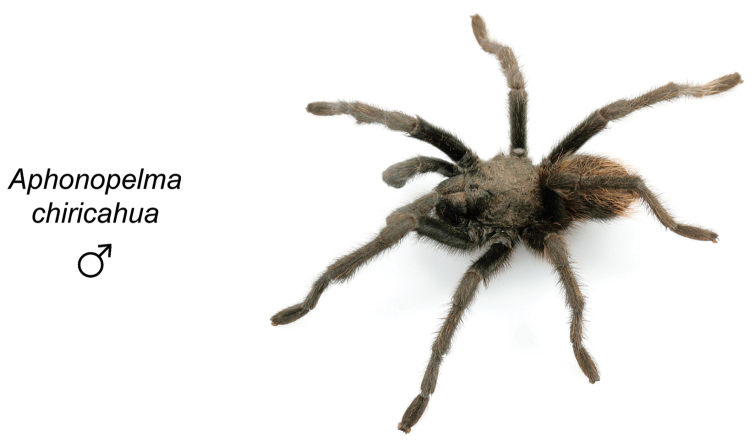
*Aphonopelma chiricahua* sp. n. live photograph. Male holotype - APH_3191. Do not have a photograph of a live female specimen.

**Figure 37.**
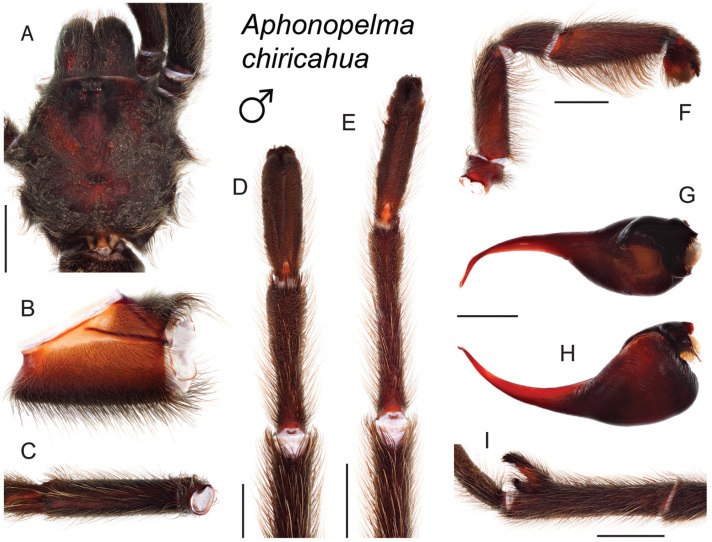
*Aphonopelma chiricahua* sp. n. **A–I** male holotype, APH_3191 **A** dorsal view of carapace, scale bar = 5mm **B** prolateral view of coxa I **C** dorsal view of femur III **D** ventral view of metatarsus III, scale bar = 3mm **E** ventral view of metatarsus IV, scale bar = 4mm **F** prolateral view of L pedipalp and palpal tibia, scale bar = 3.5mm **G** dorsal view of palpal bulb **H** retrolateral view of palpal bulb, scale bar = 1mm **I** prolateral view of tibia I (mating clasper), scale bar = 5mm.

**Figure 38.**
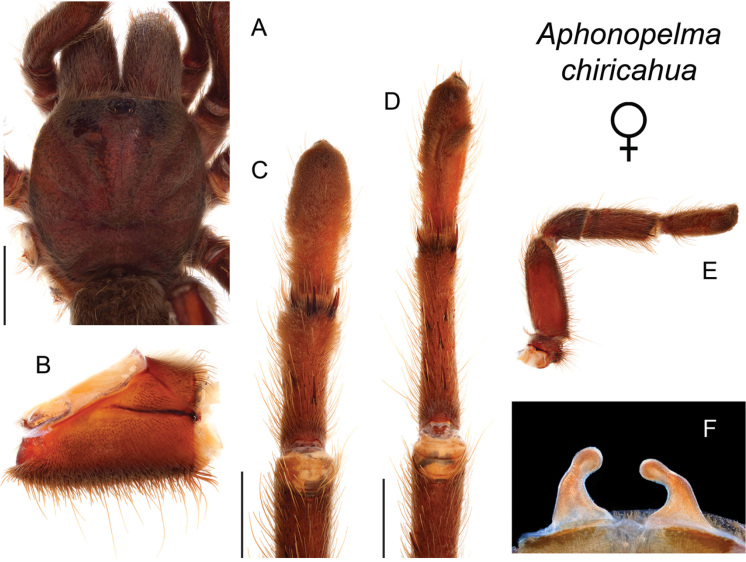
*Aphonopelma chiricahua* sp. n. **A–F** female paratype, APH_2097 **A** dorsal view of carapace, scale bar = 3.5mm **B** prolateral view of coxa I **C** ventral view of metatarsus III, scale bar = 2mm **D** ventral view of metatarsus IV, scale bar = 2mm **E** prolateral view of L pedipalp and palpal tibia **F** cleared spermathecae.

**Figure 39.**
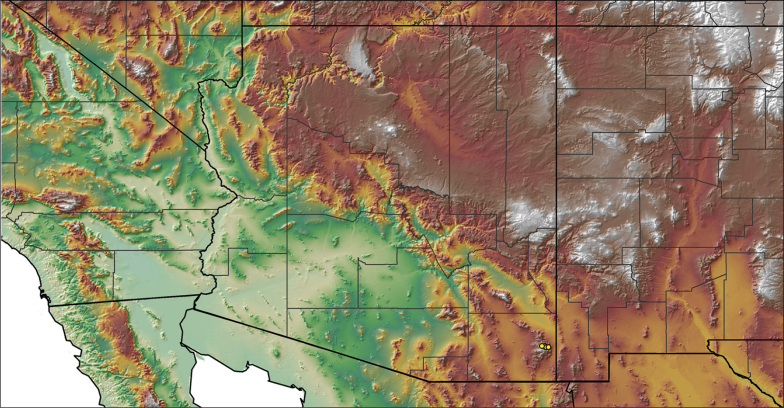
*Aphonopelma chiricahua* sp. n. distribution of known specimens. There is no predicted distribution map due to the limited number of sampling localities and restricted distribution this species possesses.

Figures 36 , 37 , 38 , 39

#### Types.

Male holotype (APH_3191) collected 1 mile up the road (42 Forest Rd.) from the lookout trail, Cochise Co., Arizona, 31.886417 -109.173356 ^1^, elev. 5083ft., 14.xi.2013, coll. Helen Snyder; deposited in AUMNH. Paratype female (APH_2097) from SWRS (Southwest Research Station, 5 miles W of Portal), Cochise Co., Arizona, 31.884056 -109.208261 ^5^, elev. 5436ft., 30.xi.1965, coll. Jon Jenson; deposited in AMNH. Paratype male (APH_2105) from SWRS, Cochise Co., Arizona, 31.883356 -109.207107 ^5^, elev. 5404ft., 31.x.1956, coll. E. Ordway; deposited in AMNH.

#### Etymology.

The specific epithet is a noun in apposition taken from type locality, the Chiricahua Mountains outside of Portal, Arizona, where this new species appears to be endemic.

#### Diagnosis.

*Aphonopelma chiricahua* (Fig. 36) is a member of the *Marxi* species group and can be distinguished by a combination of morphological, molecular, and geographic characteristics. Nuclear and mitochondrial DNA identifies *Aphonopelma chiricahua* as a phylogenetically distinct lineage (Figs 7–8), supported as a sister lineage to *Aphonopelma catalina* sp. n. (a species endemic to the Santa Catalina Mountains). The significant measurement that distinguishes male *Aphonopelma chiricahua* from its closely related phylogenetic and syntopic species is A3. Male *Aphonopelma chiricahua* can be distinguished by possessing a larger A3/M4 (≥0.65; 0.65–0.72) than *Aphonopelma catalina* (≤0.52; 0.47–0.52), *Aphonopelma madera* sp. n. (≤0.60; 0.54–0.60), *Aphonopelma parvum* sp. n. (≤0.64; 0.53–0.64), *Aphonopelma peloncillo* sp. n. (≤0.58; 0.45–0.58), and *Aphonopelma vorhiesi* (≤0.57; 0.46–0.57). The significant measurement that distinguishes female *Aphonopelma chiricahua* from its closely related phylogenetic and syntopic species is P1. Female *Aphonopelma chiricahua* can be distinguished by possessing a smaller Cl/P1 (2.21 ± (only 1 specimen)) than *Aphonopelma catalina* (≥2.75; 2.75–2.94), *Aphonopelma madera* (≥2.71; 2.71–3.01), *Aphonopelma parvum* (≥2.69; 2.69–3.04), *Aphonopelma peloncillo* (≥2.71; 2.71–3.02), and *Aphonopelma vorhiesi* (≥2.59; 2.59–2.88).

#### Description of male holotype

(APH_3191; Fig. 37). *Specimen preparation and condition*: Specimen collected live crossing road, preserved in 80% ethanol; original coloration faded due to preservation. Left legs I, III, IV, and left pedipalp removed for measurements and photographs; stored in vial with specimen. Right legs III & IV removed for DNA and stored at -80°C in the AUMNH (Auburn, AL). *General coloration*: Generally black or faded black. *Cephalothorax*: Carapace 11.42 mm long, 11.22 mm wide; densely clothed with black/faded black pubescence, slightly appressed to surface and longer than lower elevation species, slight iridescence; fringe covered in long setae not closely appressed to surface; foveal groove medium deep and straight; pars cephalica region rises gradually from foveal groove, gently arching anteriorly toward ocular area; AER slightly procurved, PER very slightly recurved; normal sized chelicerae; clypeus slightly extends forward on a curve; LBl 1.37, LBw 1.61; sternum hirsute, clothed with medium black, densely packed setae. *Abdomen*: Densely clothed in short black/brown pubescence with numerous longer, lighter setae interspersed (generally red or orange *in situ*), longer with a more hirsute appearance than lower elevation species; dense dorsal patch of black Type I urticating bristles (Cooke et al. 1972); ventral setae same as dorsal. *Legs*: Hirsute; densely clothed with medium length black/brown setae, and longer setae ventrally. Metatarsus I slightly curved. F1 12.72; F1w 3.28; P1 4.95; T1 11.37; M1 7.61; A1 6.16; F3 9.53; F3w 2.98; P3 4.11; T3 7.60; M3 7.79; A3 6.84; F4 11.41; F4w 3.20; P4 4.41; T4 9.67; M4 10.28; A4 7.78; femur III is normal - not noticeably swollen or wider than other legs. All tarsi fully scopulate. Extent of metatarsal scopulation: leg III (SC3) = 65.5%; leg IV (SC4) = 37.9%. Three ventral spinose setae and one retrolateral spinose seta on metatarsus III; nine ventral spinose setae, one prolateral spinose seta on metatarsus IV; two ventral spinose setae on tibia I. *Coxa I*: Prolateral surface a mix of fine, hair-like and thin/very thin tapered setae. *Pedipalps*: Hirsute; densely clothed in the same setal color as the other legs, with numerous longer ventral setae; one spinose seta at the apical, prolateral femur and four spinose setae on the prolateral tibia; PTl 7.34, PTw 2.82. When extended, embolus tapers with a gentle curve to the retrolateral side near apex; embolus slender, no keels.

**Variation (7).** Cl 6.837–11.42 (8.18±0.62), Cw 6.254–11.22 (8.269±0.86), LBl 0.684–1.368 (0.959±0.11), LBw 0.985–1.765 (1.292±0.11), F1 6.145–12.718 (8.731±0.77), F1w 1.898–3.281 (2.309±0.19), P1 2.859–4.947 (3.517±0.27), T1 5.851–11.372 (7.397±0.7), M1 4.09–7.61 (5.06±0.46), A1 3.572–6.165 (4.542±0.31), L1 length 22.568–42.812 (29.248±2.48), F3 5.591–9.531 (6.823±0.5), F3w 1.688–2.982 (2.147±0.18), P3 2.304–4.112 (2.896±0.23), T3 4.162–7.603 (5.286±0.43), M3 4.379–7.794 (5.317±0.45), A3 3.955–6.838 (5.003±0.35), L3 length 20.391–35.878 (25.325±1.95), F4 6.648–11.414 (8.181±0.62), F4w 1.74–3.205 (2.174±0.2), P4 2.524–4.414 (3.141±0.25), T4 5.784–9.674 (7.104±0.48), M4 5.772–10.277 (7.342±0.56), A4 4.944–7.78 (5.781±0.38), L4 length 25.672–43.559 (31.549±2.26), PTl 4.42–7.341 (5.424±0.36), PTw 1.888–2.82 (2.241±0.12), SC3 ratio 0.48–0.656 (0.556±0.02), SC4 ratio 0.33–0.404 (0.376±0.01), Coxa I setae = thin/very thin tapered, F3 condition = normal.

#### Description of female paratype

(APH_2097; Fig. 38). *Specimen preparation and condition*: Specimen collected live, preserved in unknown percentage of ethanol; original coloration faded due to preservation. Left legs I, III, IV, and pedipalp removed for photographs and measurements; stored in vial with specimen. No tissue for DNA. Genital plate with spermathecae removed and cleared, stored in vial with specimen. *General coloration*: Brown and faded black. *Cephalothorax*: Carapace 7.651 mm long, 7.479 mm wide; Hirsute, densely clothed with brown pubescence closely appressed to surface; fringe densely covered in longer setae; foveal groove medium deep and slightly procurved; pars cephalica region gently rises from thoracic furrow, arching anteriorly toward ocular area; AER slightly procurved, PER slightly recurved; robust chelicerae, clypeus extends forward on a slight curve; LBl 1.194, LBw 1.218; sternum hirsute, clothed with medium short brown setae. *Abdomen*: Densely clothed dorsally in brown setae with numerous longer, lighter setae interspersed (generally red or orange *in situ*); dense dorsal patch of black Type I urticating bristles (Cooke et al. 1972); ventral setae shorter than dorsal. *Spermathecae*: Paired and separate, tapering and curving medially towards capitate bulbs, with wide bases that are not fused. *Legs*: Hirsute; densely clothed in short and medium brown pubescence; F1 6.261; F1w 2.223; P1 3.453; T1 4.952; M1 3.398; A1 3.011; L1 length 21.075; F3 4.989; F3w 1.645; P3 2.332; T3 3.341; M3 3.231; A3 3.681; L3 length 17.574; F4 6.292; F4w 1.897; P4 3.17; T4 5.134; M4 5.114; A4 3.995; L4 length 23.705. All tarsi fully scopulate. Extent of metatarsal scopulation: leg III (SC3) = 56.2%; leg IV (SC4) = 27.7%. Two ventral spinose setae and one prolateral spinose seta on metatarsus III; six ventral spinose setae and one prolateral spinose seta on metatarsus IV. *Coxa I*: Prolateral surface a mix of fine, hair-like and tapered/thin tapered setae. *Pedipalps*: Densely clothed in the same setal color as the other legs; one spinose seta at the apical, prolateral femur, two prolateral spinose setae on the tibia.

#### Material examined.

**United States: Arizona: Cochise**: SWRS (5 miles W of Portal), 31.884056 -109.208261 ^5^, 5436ft., [APH_2097, 30/11/1965, 1♀, Jon Jenson, AMNH]; Cave Creek Canyon, 31.885338 -109.175462 ^5^, 5105ft., [APH_2101, 30/11/1963, 1♂, V. Roth, AMNH]; Upper Cave Creek Canyon, 31.900796 -109.229328 ^5^, 5997ft., [APH_2102, 1966, 1♂, Marlene Posedly, AMNH]; South West Research Station, 31.883356 -109.207107 ^5^, 5404ft., [APH_2105, 31/10/1956, 1♂, E. Ordway, AMNH]; Sunny Flat, 31.884963 -109.175964 ^4^, 5108ft., [APH_2480-A, 30/10/1971, 1♂, V. Roth, AMNH]; South West Research Station-Portal, 31.883372 -109.205727 ^5^, 5384ft., [APH_2480-B, 20/11/1971, 1♂, V. Roth, AMNH]; Chiricahua Mtns, 31.903946 -109.279016 ^6^, 8432ft., [APH_2548, 4/11/1970, 1♂, Rustler and Long Park, Joan Harper, AMNH]; Cave Creek Canyon, on Portal Rd/42A (road to SWRS), 1 mile up the road from the lookout trail, 31.886417 -109.173356 ^1^, 5083ft., [APH_3191, 14/11/2013, 1♂, Helen Snyder, AUMNH].

#### Distribution and natural history.

*Aphonopelma chiricahua* is a sky island endemic restricted to the Chiricahua Mountains in Cochise County, Arizona at elevations ranging from 1550 to 2700 meters in oak woodland, pine-oak woodland, and mixed conifer communities (Fig. 39). *Aphonopelma chiricahua* can be found inhabiting the Madrean Archipelago Level III Ecoregion. Very little is known about the natural history of this elusive species. No burrows or shelters have been observed but these spiders probably seek refuge under rocks and rarely place silk around their burrow entrances. Most specimens in natural history collections have been collected near the AMNH’s Southwest Research Station. *Aphonopelma chiricahua* is probably the only tarantula found at higher elevations in the Chiricahua Mountains but four other species are known from the area (including *Aphonopelma chalcodes*, *Aphonopelma gabeli*, *Aphonopelma parvum*, and *Aphonopelma vorhiesi*) and might be syntopic with *Aphonopelma chiricahua* at lower elevations near various canyon mouths. An unconfirmed adult female (no voucher specimen available, identification tentatively assigned based on a photograph and locality data) was found walking across a road in Cave Creek Canyon in August during the summer monsoon season (A. Abela 2006, pers. comm.). Vouchered adult males were collected between late October and late November; an adult male and female (no voucher specimens available, identification tentatively assigned based on video images and locality data) were found mating during daylight hours in early December along Echo Canyon Trail in Chiricahua National Monument (Chiricahua National Monument 2015, pers. comm.; https://www.facebook.com/video.php?v=785835144804065). The holotype male was collected wandering across 42 Forest Road in November, one year before this video was taken. These data indicate that the breeding season for this species is restricted to late autumn and early winter, similar to that of other high-elevation species in the region (*Aphonopelma catalina* and *Aphonopelma madera*).

#### Conservation status.

It is difficult to assess the conservation status of *Aphonopelma chiricahua* due to small sample sizes and the very cryptic nature of these spiders. This species does not occur outside of the Chiricahua Mountains so its narrow distribution is one factor that may threaten its future survival. These mountains have the advantage of being somewhat protected by their remoteness and management by the federal government (Coronado National Forest, Douglas Ranger District, Chiricahua National Monument); however, these habitats have also been subjected to habitat degradation from recent urban growth, human-caused forest fires, off-road driving, poorly managed livestock grazing, invasive species, recreational activities, human immigrants, and illegal drug trafficking (Coronado Planning Partnership 2008). Climate change in the sky island region (Brusca et al. 2013, Mitchell and Ober 2013, Moore et al. 2013, Hendrixson et al. 2015) also poses a potential threat to the future survival of *Aphonopelma chiricahua*.

#### Remarks.

*Aphonopelma chiricahua* is morphologically very similar to other Madrean sky island endemics in the *Marxi* species group, although generally smaller than *Aphonopelma catalina*, *Aphonopelma madera*, and *Aphonopelma vorhiesi*. Other important ratios that distinguish males: *Aphonopelma chiricahua* possess a larger Cl/M3 (≥1.46; 1.46–1.61) than *Aphonopelma catalina* (≤1.42; 1.26–1.42), *Aphonopelma parvum* (≤1.39; 1.20–1.39), *Aphonopelma peloncillo* (≤1.40; 1.20–1.40), and *Aphonopelma vorhiesi* (≤1.43; 1.24–1.43); by possessing a larger L3/Cl (≥2.98; 2.98–3.19) than *Aphonopelma madera* (≤2.95; 2.71–2.95). Other important ratios that distinguish females: *Aphonopelma chiricahua* possess a smaller M3/P4 (1.02 ± (only 1 specimen)) than *Aphonopelma catalina* (≥1.48; 1.48–1.52), *Aphonopelma madera* (≥1.39; 1.39–1.48), *Aphonopelma parvum* (≥1.32; 1.32–1.54), *Aphonopelma peloncillo* (≥1.39; 1.39–1.67), and *Aphonopelma vorhiesi* (≥1.27; 1.27–1.64). For both males and females, certain morphometrics have potential to be useful, though due to the amounts of variation, small number of specimens, and the small differences between species, no other are claimed to be significant at this time (see Suppl. material 2). During evaluation of PCA morphospace, males of *Aphonopelma chiricahua* separate from *Aphonopelma parvum* and all other miniature species along PC1~2, but do not separate from *Aphonopelma catalina*, *Aphonopelma madera*, *Aphonopelma peloncillo*, and *Aphonopelma vorhiesi*. Though we only have one female of *Aphonopelma chiricahua*, it appears to separate from all other sky island species (*Aphonopelma catalina*, *Aphonopelma madera*, *Aphonopelma peloncillo*, and *Aphonopelma vorhiesi*), grouping more closely with the miniature species in morphospace along PC1~2. Interestingly, *Aphonopelma chiricahua* males separate from *Aphonopelma parvum*, *Aphonopelma peloncillo*, and *Aphonopelma vorhiesi* in three-dimensional PCA morphospace (PC1~PC2~PC3), but do not separate from *Aphonopelma catalina*, *Aphonopelma madera*, *Aphonopelma marxi*. There is only one *Aphonopelma chiricahua* female, but she separates from all other phylogenetic sister species or syntopic species - *Aphonopelma catalina*, *Aphonopelma madera*, *Aphonopelma marxi*, *Aphonopelma parvum*, *Aphonopelma peloncillo*, and *Aphonopelma vorhiesi*. PC1, PC2, and PC3 explain ≥96% of the variation in all analyses.

This species was first identified as novel by Jung (1975) but was never formally described. Of particular note is the size of the holotype male and paratype female; the two specimens probably represent opposite extremes on the size spectrum for what is possible in this species. The rather large holotype male was chosen because it was a fresh specimen and could be associated with molecular data. The female, though small, is sexually mature (based on spermathecal development).

### Aphonopelma eutylenum

Taxon classificationAnimaliaAraneaeTheraphosidae

Chamberlin, 1940

**Figure 40.**
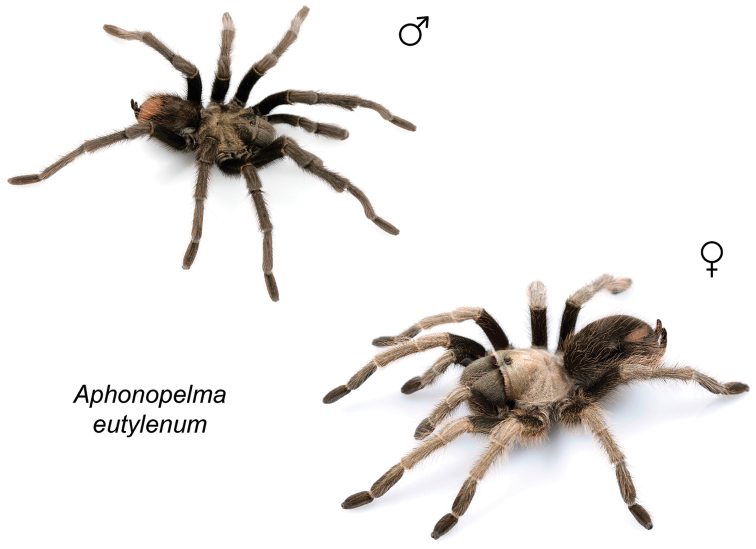
*Aphonopelma eutylenum* Chamberlin, 1940 specimens, live photographs. Male (L) - APH_3207; Female (R) - APH_3108.

**Figure 41.**
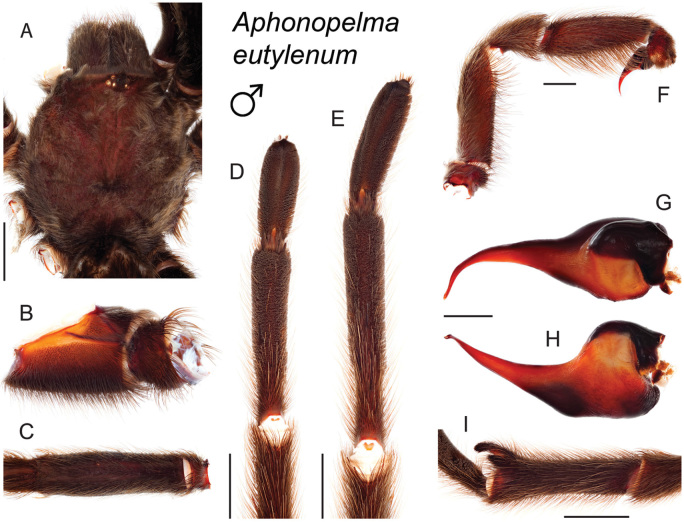
*Aphonopelma eutylenum* Chamberlin, 1940. **A–I** male specimen, APH_1088 **A** dorsal view of carapace, scale bar = 6mm **B** prolateral view of coxa I **C** dorsal view of femur III **D** ventral view of metatarsus III, scale bar = 5mm **E** ventral view of metatarsus IV, scale bar = 4.5mm **F** prolateral view of L pedipalp and palpal tibia, scale bar = 3mm **G** dorsal view of palpal bulb **H** retrolateral view of palpal bulb, scale bar = 1mm **I** prolateral view of tibia I (mating clasper), scale bar = 6mm.

**Figure 42.**
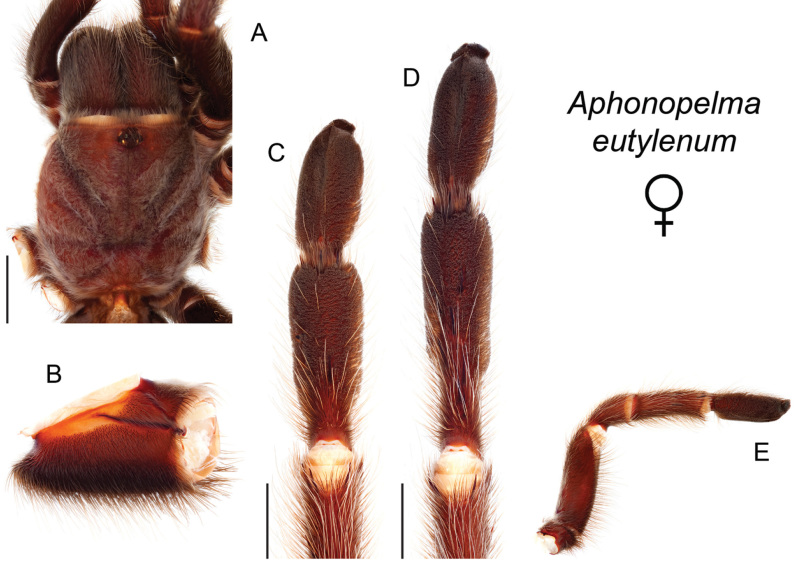
*Aphonopelma eutylenum* Chamberlin, 1940. **A–E** female specimen, APH_1031. **A** dorsal view of carapace, scale bar = 8mm **B** prolateral view of coxa I **C** ventral view of metatarsus III, scale bar = 4.5mm **D** ventral view of metatarsus IV, scale bar = 5mm **E** prolateral view of L pedipalp and palpal tibia.

**Figure 43.**
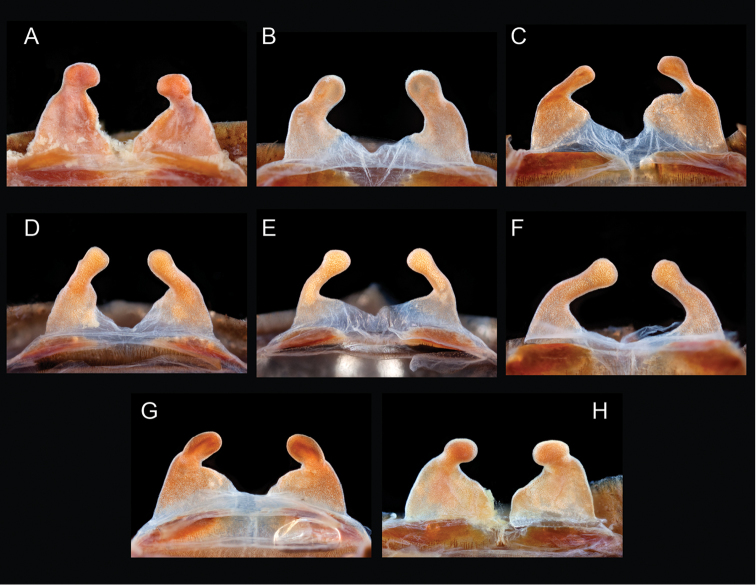
*Aphonopelma eutylenum* Chamberlin, 1940. **A–H** cleared spermathecae. **A** *eutylenum* allotype **B** *eutylenum* paratype **C** APH_1018 **D** APH_1031 **E** APH_1045 **F** APH_2035 **G** AUMS_3303 **H** *cryptethum* allotype.

**Figure 44.**
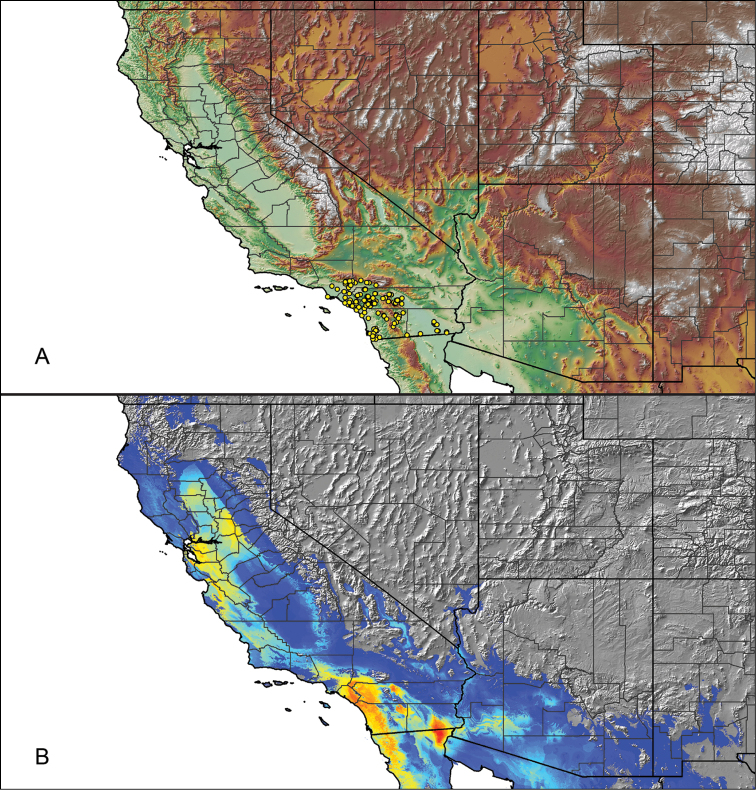
*Aphonopelma eutylenum* Chamberlin, 1940. **A** distribution of known specimens **B** predicted distribution; warmer colors (red, orange, yellow) represent areas of high probability of occurrence, cooler colors (blue shades) represent areas of low probability of occurrence.

Figures 40 , 41 , 42 , 43 , 44

Aphonopelma eutylenum Chamberlin, 1940: 9; male holotype and female allotype from San Diego, San Diego Co., California, 32.715738 -117.161085^6^, elev. 54ft., 1935, coll. unknown; deposited in AMNH. One male from San Diego, San Diego Co., California, 32.715738 -117.161085^6^, elev. 54ft., 20.vii.1925, coll. unknown; deposited in AMNH. One female paratype from San Diego, San Diego Co., California, 32.715738 -117.161085^6^, elev. 54ft., 28.v.1927, coll. unknown; deposited in AMNH. [examined]
Rhechostica eutylenum Raven, 1985: 149.
Aphonopelma eutylenum Smith, 1995: 99.
Aphonopelma chambersi Smith, 1995: 86; male holotype from Garner Valley, S of Idyllwild-Pine Cove, Riverside Co., California, 33.635459 -116.643974^5^, elev. 4475ft., no collecting date, coll. Aaron Chambers; deposited in BMNH. [examined] **syn. n.**
Aphonopelma clarum Chamberlin, 1940: 10; male holotype from mountains near Claremont, Los Angeles Co., California, 34.137812 -117.718194^4^, elev. 1571ft., no collecting date, coll. R.V. Chamberlin; deposited in AMNH. [examined]
Rhechostica clarum Raven, 1985: 149.
Aphonopelma clarum Smith, 1995: 89. **syn. n.**
Aphonopelma cryptethus Chamberlin, 1940: 16; male holotype from Los Angeles, Los Angeles Co., California, 34.052234 -118.243685^6^, elev. 309ft., 9.v.1908, coll. unknown; deposited in AMNH. Female allotype from Claremont, Los Angeles Co., California, 34.096676 -117.719778^5^, elev. 1166ft., 9.v.1908, coll. unknown; deposited in AMNH. [examined]
Rhechostica cryptethus Raven, 1985: 149.
Aphonopelma cryptethum Smith, 1995: 95. **syn. n.**
Aphonopelma sandersoni Smith, 1995: 138; male holotype and female allotype from San Bernardino Mountains., San Bernardino Co., California, 34.180742 -117.164638^7^, elev. 3137ft., x.1995, coll. R. Douglas; deposited in BMNH. [examined] **syn. n.**
Aphonopelma sullivani Smith, 1995: 149; male holotype from Coachella Valley, Palm Springs, Riverside Co., California, 33.767209 -116.359868^6^, elev. 277ft., ix.1991, coll. Michael Sullivan; deposited in BMNH. [examined] **syn. n.**

#### Diagnosis.

*Aphonopelma eutylenum* (Fig. 40) is a member of the *iodius* species group and can be identified by a combination of morphological, molecular, and geographic characteristics. Nuclear and mitochondrial DNA (*CO1*) identifies *Aphonopelma eutylenum* as a strongly supported phylogenetically distinct monophyletic lineage (Figs 7–8), supported as the sister lineage to *Aphonopelma iodius* and *Aphonopelma johnnycashi* sp. n. Aside from measurements, no pronounced morphological features are useful for distinguishing *Aphonopelma eutylenum* from closely related phylogenetic species. Significant measurements that distinguish male *Aphonopelma eutylenum* from its closely related phylogenetic and syntopic species are T3 and the extent of scopulation on metatarsus IV. Male *Aphonopelma eutylenum* can be distinguished by possessing a larger L4 scopulation extent (62%-77%) than *Aphonopelma steindachneri* (21%–31%) and *Aphonopelma xwalxwal* sp. n. (34%–48%); and by possessing a smaller T1/T3 (≤1.23; 1.16–1.23) than *Aphonopelma johnnycashi* (≥1.25; 1.25–1.31) and *Aphonopelma steindachneri* (≥1.28; 1.28–1.36). There are no significant measurements that separate male *Aphonopelma eutylenum* from *Aphonopelma chalcodes* and *Aphonopelma iodius*. Significant measurements that distinguish female *Aphonopelma eutylenum* from its closely related phylogenetic and syntopic species are F1 and the extent of scopulation on metatarsus IV. Female *Aphonopelma eutylenum* can be distinguished by possessing a larger L4 scopulation extent (62%–75%) than *Aphonopelma steindachneri* (24%–34%); and by possessing a smaller F1/M3 (≤1.52; 1.41–1.52) than *Aphonopelma johnnycashi* (≥1.52; 1.52–1.61). There are no significant measurements that separate female *Aphonopelma eutylenum* from *Aphonopelma chalcodes* and *Aphonopelma iodius*. Females of *Aphonopelma xwalxwal* are unknown at this time and cannot be compared.

#### Description.

Originally described by Chamberlin (1940).

#### Redescription of male exemplar

(APH_1088; Fig. 41). *Specimen preparation and condition*: Specimen collected live crossing road, preserved in 80% ethanol; deposited in AUMNH; original coloration faded due to preservation. Left legs I, III, IV, and left pedipalp removed for measurements and photographs; stored in vial with specimen. Right leg III removed for DNA and stored at -80°C in the AUMNH (Auburn, AL). *General coloration*: Black, grey, and faded brown. *Cephalothorax*: Carapace 16.30 mm long, 15.89 mm wide; Hirsute; densely clothed with light brown iridescent pubescence mostly appressed to surface; fringe covered in long setae not closely appressed to surface; foveal groove medium deep and straight; pars cephalica region rises gradually from foveal groove, gently arching anteriorly toward ocular area; AER and PER not in normal placement - looks to be developmental issues; normal sized chelicerae; clypeus extends forward on a curve; LBl 1.94, LBw 2.08; sternum hirsute, clothed with black, densely packed setae. *Abdomen*: Densely clothed in short black/brown pubescence with numerous longer red/orange setae interspersed; possessing a dense dorsal patch of black Type I urticating bristles (Cooke et al. 1972). *Legs*: Hirsute, particularly ventrally; densely clothed in a mix of black or faded black pubescence, femurs are darker. Metatarsus I slightly curved. F1 16.12; F1w 4.16; P1 6.93; T1 13.24; M1 13.41; A1 9.02; F3 14.19; F3w 4.41; P3 5.82; T3 10.82; M3 14.44; A3 8.30; F4 16.77; F4w 4.10; P4 6.71; T4 13.76; M4 18.16; A4 9.02; femur III is slightly swollen. All tarsi fully scopulate. Extent of metatarsal scopulation: leg III (SC3) = 83.5%; leg IV (SC4) = 71.6%. Two ventral spinose setae on metatarsus III; four ventral spinose setae on metatarsus IV; one large megaspine is present on the retrolateral tibia at the apex of the mating clasper - this can be seen when viewing the prolateral face of the mating clasper. *Coxa I*: Prolateral surface a mix of fine, hair-like and tapered setae. *Pedipalps*: Hirsute; densely clothed in the same setal color as the other legs, with numerous longer ventral setae; one spinose seta on the apical, prolateral femur and two spinose setae on the prolateral tibia; PTl 9.361, PTw 3.47. When extended, embolus tapers and gently curves to the retrolateral side near apex; embolus very slender, no keels; distinct ventral bulge at the shift from bulb to embolus.

**Variation (8).** Cl 12.01–18.42 (15.497±0.73), Cw 10.92–16.38 (14.213±0.68), LBl 1.53–2.14 (1.846±0.09), LBw 1.57–2.38 (2.113±0.11), F1 12.67–16.86 (15.34±0.5), F1w 2.85–4.51 (3.799±0.2), P1 4.94–7.22 (6.028±0.28), T1 10.52–13.62 (12.456±0.42), M1 10.08–14.57 (12.415±0.59), A1 6.71–9.18 (8.328±0.32), L1 length 45.83–61.45 (55.503±1.95), F3 10.75–14.19 (12.924±0.45), F3w 3.21–4.77 (3.944±0.19), P3 3.98–5.98 (5.186±0.28), T3 8.82–11.26 (10.247±0.36), M3 10.59–14.7 (13.179±0.67), A3 6.75–8.48 (7.771±0.26), L3 length 41.73–54.29 (49.353±2.08), F4 12.93–16.82 (15.241±0.54), F4w 2.85–4.41 (3.656±0.19), P4 4.61–6.71 (5.734±0.26), T4 10.79–14.21 (12.781±0.53), M4 13.84–18.71 (16.776±0.77), A4 7.42–9.47 (8.556±0.31), L4 length 49.65–65.06 (59.139±2.5), PTl 7.375–10.727 (8.984±0.35), PTw 2.319–3.47 (3.082±0.14), SC3 ratio 0.728–0.93 (0.827±0.02), SC4 ratio 0.626–0.772 (0.701±0.02), Coxa I setae = tapered, F3 condition = slightly swollen.

#### Description of female exemplar

(APH_1031; Figs 42–43). *Specimen preparation and condition*: Specimen collected live from burrow, preserved in 80% ethanol; deposited in AUMNH; original coloration faded due to preservation. Left legs I, III, IV, and pedipalp removed for photographs and measurements; stored in vial with specimen. Right leg III removed for DNA and stored at -80°C in the AUMNH (Auburn, AL). Genital plate with spermathecae removed and cleared, stored in vial with specimen. *General coloration*: Faded brown/black. *Cephalothorax*: Carapace 16.55 mm long, 15.05 mm wide; Hirsute, densely clothed with light brown pubescence closely appressed to surface; fringe densely covered in longer setae; foveal groove medium deep and straight; pars cephalica region gently rises from thoracic furrow, arching anteriorly toward ocular area; AER procurved, PER slightly recurved; large chelicerae, clypeus extends forward on a curve; LBl 2.05, LBw 2.38; sternum hirsute, clothed with dark brown setae. *Abdomen*: Densely clothed dorsally in short black setae with numerous longer, lighter setae interspersed (generally red or orange *in situ*); dense dorsal patch of black Type I urticating bristles (Cooke et al. 1972); ventral side with shorter black/dark brown setae. *Spermathecae*: Paired and separate with wide bases, tapering and curving medially towards capitate bulbs, with smaller bulges laterally and medially. *Legs*: Very hirsute, particularly ventrally; densely clothed in medium and long brown pubescence, femurs darker. F1 13.77; F1w 4.14; P1 5.95; T1 10.15; M1 8.89; A1 7.44; F3 10.84; F3w 3.83; P3 5.29; T3 8.95; M3 9.07; A3 7.29; F4 13.70; F4w 3.86; P4 5.51; T4 11.43; M4 12.70; A4 8.14. All tarsi fully scopulate. Extent of metatarsal scopulation: leg III (SC3) = 88.2%; leg IV (SC4) = 74.7%. Two ventral spinose setae on metatarsus III; seven ventral spinose setae on metatarsus IV. *Coxa I*: Prolateral surface a mix of fine, hair-like and tapered setae. *Pedipalps*: Densely clothed in the same setal color as the other legs; one spinose seta on the apical, prolateral femur and five spinose setae on the prolateral tibia.

**Variation (8).** Cl 15.23–20.84 (18.248±0.72), Cw 13.33–17.78 (16.206±0.61), LBl 1.95–2.81 (2.353±0.12), LBw 2.28–3.04 (2.675±0.1), F1 12.87–15.78 (14.599±0.5), F1w 3.62–4.94 (4.357±0.2), P1 5.38–7.34 (6.479±0.25), T1 9.82–13.05 (11.243±0.42), M1 8.36–10.75 (9.565±0.33), A1 6.6–7.92 (7.36±0.17), L1 length 43.43–54.02 (49.137±1.72), F3 10.53–13.35 (12.208±0.42), F3w 3.3–4.67 (4.044±0.16), P3 5.12–7.26 (5.776±0.27), T3 8.3–9.93 (8.82±0.2), M3 8.64–11.06 (9.958±0.36), A3 6.49–7.87 (7.084±0.16), L3 length 39.24–48.1 (43.845±1.2), F4 13.21–16.57 (14.909±0.53), F4w 3.42–4.73 (4.106±0.19), P4 5.51–7.40 (6.312±0.32), T4 10.5–12.84 (11.509±0.31), M4 11.84–15.26 (13.32±0.48), A4 6.94–8.82 (7.779±0.26), L4 length 48.62–60.13 (54.192±1.91), SC3 ratio 0.772–0.906 (0.851±0.02), SC4 ratio 0.628–0.747 (0.683±0.02), Coxa I setae = tapered. Spermathecae variation can be seen in Figure 43.

#### Material examined.

**United States: California: Imperial**: 1.5 miles N Gordons Well off of I-8, 32.7636 -114.966431 ^2^, 248ft., [APH_0152, unknown, 1 juv, T.R. Prentice, AUMNH]; E of Brawley, off of Hwy 78, 32.969167 -115.259444 ^5^, 3ft., [APH_3116, 10/1992, 1♀, T.R. Prentice, AUMNH]; East Mesa, 33.058096 -115.324147 ^5^, 46ft., [AUMS_3332, 30/5/1995, 1♂, unknown, AUMNH]; East Mesa Exit of I-8, 2.1 miles N of I-8, 32.73013 -114.955351 ^4^, 158ft., [AUMS_2358, 26/10/1993, 1♂, Greg Ballmer, AUMNH]; East Mesa, 1/2 mile E of Highline Canal and 3/10 mile S of Hwy 78, 32.968133 -115.290717 ^1^, 37ft., [APH_3211, 15/11/2013, 1♂, W. Icenogle, AUMNH]; East Mesa, 5.4 miles E of Hwy 115 E of Wander Linder Exit of I-8, 32.788365 -115.207479 ^4^, 50ft., [AUMS_3303, 30/5/1995, 1♀, unknown, AUMNH]; East Mesa, 5.4 miles E of Hwy 115 E of Wander Linder Exit, 2.1 miles N, 32.790359 -115.240569 ^4^, 63ft., [AUMS_3341, 30/5/1995, 1♂, T.R. Prentice, AUMNH]; Hwy 78, 1.0 miles east of Highline Canal, 32.970575 -115.28374 ^4^, 30ft., [AUMS_2356,-2357 3/11/1998, 2♂, Greg Ballmer, AUMNH]; Hwy 78, 3.35 miles E of East Highline Canal, N of 78 ~0.4 miles, 32.972797 -115.242665 ^4^, 90ft., [AUMS_2674, unknown, 1♀, T.R. Prentice, AUMNH]; off I-8 split, W of Devils Canyon, W of Ocotillo, 32.67371 -116.10147 ^1^, 2238ft., [APH_1028-1029, 18/5/2010, 1♂, 1♀, Chris A. Hamilton, Xavier Atkinson, AUMNH]; Pinto Wash, 32.708139 -115.715525 ^5^, 13ft., [APH_2690, 5/3/1961, 1♂, Laurie Breese, AMNH]; 15 miles west of Glamis , 33.008844 -115.321813 ^5^, -7ft., [APH_2154, 4/10/1961, 1♀, 1♂, Findlay E. Russell, AMNH]; **Los Angeles**: Claremont College - Frary Dining Hall on steps, 34.10018 -117.710527 ^4^, 1225ft., [AUMS_3308, 11/11/1991, 1♂, Martin Ramirez, AUMNH]; Claremont, Mt. Baldy Rd and Mills Ave, 34.13823 -117.7072 ^1^, 1660ft., [APH_1087, 8/10/2010, 1♂, Chris A. Hamilton, AUMNH]; Claremont, Thompson Creek Trail, 34.13716 -117.71389 ^1^, 1582ft., [APH_1088-1089, 8/10/2010, 3♂, Chris A. Hamilton, Dylan Burke, Warren Burke, AUMNH]; [APH_2023-2027, 8/10/2010, 5♂, Chris A. Hamilton, AUMNH]; Glendora Ridge Rd., Angeles National Forest, 34.221163 -117.739386 ^6^, 3666ft., [AUMS_2686, 17/11/1991, 1♂, John Adams, AUMNH]; Glendore, 34.136118 -117.865337 ^5^, 774ft., [APH_2687, 18/10/1963, 1♂, R. Ayre, AMNH]; Morris Lake, 34.192644 -117.865749 ^5^, 1207ft., [APH_2711, 8/1956, 1♂, Gay J. Kay, AMNH]; Palos Verde, 33.768911 -118.373032 ^5^, 1132ft., [AUMS_2496, unknown, 1♂, C. Grover, AUMNH]; Phillips Ranch Rd., N of Freeway, 34.028759 -117.772093 ^5^, 862ft., [AUMS_2498, 10/1992, 1♂, Dianna Hansen, AUMNH]; Pico Rivera, 33.983069 -118.096735 ^5^, 163ft., [AUMS_3336, 4/10/1963, 1♂, unknown, AUMNH]; San Gabriel Mtns (N of Azusa), 34.2175 -117.8553 ^1^, 1697ft., [APH_0895, 6/2007, 1♂, Chris A. Hamilton, AUMNH]; San Gabriel Mtns., near jct. Mt. Baldy Rd. and Glendora Ridge Rd., 0.8 miles up Glendora Ridge Rd. to dirt road winding around mtn., 34.228259 -117.670819 ^5^, 4532ft., [AUMS_2490, 24/6/1989, 1♂, T.R. Prentice, AUMNH]; **Orange**: 21263 Mohler Drive, Anaheim, 33.853135 -117.755736 ^2^, 617ft., [APH_2707, 1963, 2♂, F.W. Handsfield Jr., AMNH]; Brea, 33.916706 -117.899988 ^5^, 362ft., [AUMS_2487, 15/10/1975, 1♂, Jim Lawrence, AUMNH]; Caspers Wilderness Park (San Juan Capistrano), 33.5572 -117.556783 ^1^, 709ft., [APH_0981, 9/2009, 1♀, Kyle Dickerson, AUMNH]; El Toro (Marine Corps Air Station MCAS), 33.673927 -117.72924 ^5^, 340ft., [AUMS_2588, 26/9/1966, 1♂, 1st Lt Jim Hardy, AUMNH]; Hidden Hills area, (Laguna Nigel) near San Juan Capistrano, 33.529839 -117.685743 ^5^, 525ft., [AUMS_3309, 20/11/1991, 1♂, Scott Elgnam, AUMNH]; Laguna Beach , 33.542248 -117.783109 ^5^, 16ft., [APH_2155, 26/7/1931, 1♀, R.V. Chamberlin, AMNH]; [APH_2472, 26/5/1931, 1 juv, Wilton Ivie, AMNH]; Laguna Hills, 33.599722 -117.699444 ^5^, 372ft., [AUMS_2481, 28/10/1967, 1♂, Tom Simms, AUMNH]; Laguna Niguel, 33.522526 -117.707553 ^5^, 407ft., [AUMS_2488, unknown, 1♀, unknown, AUMNH]; Lookout Point, Corona del Mar, 33.592518 -117.877554 ^5^, 0ft., [APH_2708, 16/10/1962, 1♂, Jerry Parrish, AMNH]; San Clemente, E of Avenida Pico, off The Christianitos Trail, 33.455 -117.57523 ^1^, 388ft., [APH_1035, 22/5/2010, 1♀, Chris A. Hamilton, Xavier Atkinson, AUMNH]; San Jauquin Hills, S of Lagunas Reservoir, off Hwy 133, 33.607836 -117.75685 ^5^, 397ft., [AUMS_3316, 7/5/1989, 1♂, T.R. Prentice, AUMNH]; San Juan Capistrano, 33.501693 -117.662551 ^5^, 127ft., [AUMS_3327, 23/8/1964, 2♀, 1♂, J.H. Menees, AUMNH]; Santa Ana, 33.745573 -117.867931 ^5^, 112ft., [APH_2153, 17/7/1931, 2♀, R.V. Chamberlin, AMNH]; Starr Ranch, between Caspers and Clev. Nat Forest, 33.632326 -117.555835 ^5^, 999ft., [AUMS_2399, 13/8/1997, 1♂, unknown, AUMNH]; Starr Ranch, N Perusker and Bell Canyon Rd, 33.626176 -117.556666 ^5^, 919ft., [AUMS_2404, 4/8/1997, 1♂, unknown, AUMNH]; **Riverside**: 1 mile N of Winchester, 33.72247 -117.081149 ^4^, 1558ft., [APH_2171, 13/10/1968, 1♂, W. Icenogle, AMNH]; 1 mile NW of Winchester, 33.71869 -117.096162 ^5^, 1745ft., [AUMS_2590, 3/9/1990, 1♀, Gary Larson, AUMNH]; 2 miles N of Winchester, 33.734766 -117.076361 ^5^, 1556ft., [AUMS_2491, 30/10/1995, 1♂, Ken Harbridge, AUMNH]; 2 miles NE of Winchester, 33.734476 -117.074678 ^5^, 1562ft., [AUMS_3301, 13/10/1995, 1♀, W. Icenogle, AUMNH]; 7 miles E of Hemet - off Hwy 74 in San Jacinto Mtns, 33.72003 -116.7916 ^1^, 2625ft., [APH_3114, 22/7/2012, 1 juv, Chris A. Hamilton, AUMNH]; at base of Double Butte, Washington Ave, 1/2 mile N of Winchester town center, 33.714 -117.085 ^1^, 1492ft., [APH_3207, 28/10/2013, 1♂, Patrick Parker, AUMNH]; [APH_3208, 6/11/2013, 1♂, Kim Johnson, AUMNH]; Box Spring Mtns, 33.972945 -117.29587 ^6^, 2351ft., [AUMS_2585, 7/1991, 2♂, T.R. Prentice, AUMNH]; Cajalco Canyon, 8.6 miles southeast of Corona at Lake Mathews, 33.829201 -117.462018 ^4^, 1368ft., [APH_2692, 3/11/1963, 1♂, W. A. Powder, AMNH]; Chino Canyon, on road to Palm Springs Aerial Tramway (Valley Station), 3 miles W of junction with Hwy 111 San Jacinto Mtns, 33.8447 -116.60125 ^1^, 1971ft., [APH_3138, 10/9/2013, 1♂, W. Icenogle, AUMNH]; Chino Canyon, Palm Springs Aerial Tramway (Valley Station), San Jacinto Mtns, 33.838433 -116.6132 ^1^, 2532ft., [APH_3137, 22/9/2013, 1♂, W. Icenogle, AUMNH]; [APH_3139, 4/9/2013, 1♂, W. Icenogle, AUMNH]; [APH_3140, 5/9/2013, 1♂, W. Icenogle, AUMNH]; Deep Canyon, 33.664423 -116.372819 ^5^, 764ft., [AUMS_2629, unknown, 1♂, unknown, AUMNH]; E of Perris Valley Airport, Perris, 33.760961 -117.202485 ^5^, 1417ft., [AUMS_2628, 19/7/1994, 1♀, unknown, AUMNH]; East of Sun City off I-215, Sun Mtns east, 33.702126 -117.171559 ^5^, 1849ft., [AUMS_3285, 5/2/1989, 1♂, T.R. Prentice, AUMNH]; Garner Valley, off Fobes Ranch Rd - off Hwy 74, S of Lake Hemet, 33.63184 -116.63173 ^1^, 4501ft., [APH_3111-3113, 22/7/2012, 2♂, 1♀, Chris A. Hamilton, Amy Skibiel, AUMNH]; just S of the Garner Valley, outside Nightingale, off Piñon Flats Transfer Station Rd - off Hwy 74, Sawmill Trailhead, 33.5802 -116.45131 ^1^, 4047ft., [APH_3106-3110, 21/7/2012, 2♂, 3♀, Chris A. Hamilton, Amy Skibiel, AUMNH]; Lake Elsinore, near J Bethlee home, 33.668144 -117.327394 ^5^, 1293ft., [AUMS_2503, 9/1998, 1♂, J. Bethlee, AUMNH]; Lake Mathews, 33.828903 -117.437512 ^5^, 1401ft., [AUMS_3284, 14/4/1968, 1♂, M. Cline, AUMNH]; Lake Mathews, heading N on Cajalco Rd., 0.5 mile E of junction La Sierra Ave., 33.827762 -117.451927 ^4^, 1397ft., [AUMS_2517, 12/11/1998, 1♂, T.R. Prentice, AUMNH]; Lake Skinner, at entrance gate, 33.590905 -117.07877 ^5^, 1408ft., [AUMS_2499, 26/11/1997, 1♂, Tom Ash, AUMNH]; Lake Skinner, below U3 on rd. above oaks, 33.604263 -117.038208 ^5^, 1567ft., [AUMS_2681, 29/10/1992, 1♂, T.R. Prentice, AUMNH]; Lake Skinner, below U5, above lake, 33.609043 -117.037303 ^5^, 1661ft., [AUMS_2507, 28/9/1997, 1♀, T.R. Prentice, AUMNH]; Lake Skinner, between road and B9, 33.580808 -117.088785 ^5^, 1353ft., [AUMS_2255, 16/8/1998, 1♂, T.R. Prentice, AUMNH]; Lake Skinner, by 90 deg. turn in dirt rd., S. of U7?, S side of lake, 33.575862 -117.066618 ^5^, 1539ft., [AUMS_2501, 3/9/1987, 1♀, T.R. Prentice, AUMNH]; Lake Skinner, found on N side of lake E of Borel rd, 33.588117 -117.028523 ^5^, 1517ft., [AUMS_2581, 18/8/1999, 1♂, T.R. Prentice, AUMNH]; Lake Skinner, Rowson Canyon, 1.5 miles N of middle gate, 0.8 miles S of Loop Rd., 33.573703 -117.066585 ^4^, 1519ft., [AUMS_2505, 28/9/1997, 1♀, T.R. Prentice, AUMNH]; Lake Skinner, S of B5, close to lake, 33.569155 -117.064281 ^5^, 1534ft., [AUMS_2506, 28/9/1997, 1♂, T.R. Prentice, AUMNH]; Lake Skinner, S side, 33.578777 -117.057225 ^5^, 1535ft., [AUMS_2518, 11/9/1997, 1♂, Adam Bucklin , AUMNH]; Lake Skinner, SE of H2O, 33.573965 -117.025814 ^5^, 1605ft., [AUMS_3325, 3/9/1997, 1♀, Adam Backlin, AUMNH]; Lamb Canyon, 33.855805 -117.015244 ^5^, 1590ft., [AUMS_2587, 23/7/1988, 1♂, T.R. Prentice, AUMNH]; Lamb Canyon - N of jct 79 and Gilman Springs Rd., 33.858779 -117.016279 ^5^, 1627ft., [AUMS_2401, 14/10/1989, 1♂, T.R. Prentice, AUMNH]; Lamb Canyon (S of Beaumont), off Hwy 79, 33.85507 -117.00342 ^1^, 1911ft., [APH_1041-1042, 27/5/2010, 2♀, Chris A. Hamilton, Tom Prentice, AUMNH]; Lamb Canyon, Hwy 79, 33.853149 -117.007918 ^5^, 1660ft., [AUMS_2407, 27/6/1987, 1♂, T.R. Prentice, AUMNH]; Mt Vernon Park, base of Sugarloaf Mtn, 33.988098 -117.295535 ^5^, 2078ft., [AUMS_2497, 30/9/1992, 1♀, James Adams, AUMNH]; Murrieta, 33.553914 -117.213923 ^5^, 1103ft., [AUMS_2655, 13/10/2001, 1♂, Bill, AUMNH]; [AUMS_3297, 8/9/1996, 1♂, C. Webster, AUMNH]; P.L. Boyd Deep Canyon Reserve, 0.1 miles above reserve gate, 33.66925 -116.372791 ^4^, 701ft., [AUMS_2337, 21/9/1995, 1♂, T.R. Prentice, AUMNH]; P.L. Boyd Deep Canyon Reserve, 0.1 miles below reserve gate, 33.672099 -116.372355 ^4^, 670ft., [AUMS_2335, 27/9/1995, 1♂, T.R. Prentice, AUMNH]; P.L. Boyd Deep Canyon Reserve, 0.55 miles N of reserve station, 33.655658 -116.373614 ^4^, 883ft., [AUMS_2336, 22/9/1995, 1♀, T.R. Prentice, AUMNH]; P.L. Boyd Deep Canyon Reserve, 0.6 miles below reserve gate, 33.677492 -116.370089 ^4^, 615ft., [AUMS_2334, 27/9/1995, 1♂, T.R. Prentice, AUMNH]; [AUMS_2368, 21/9/1995, 1♂, T.R. Prentice, AUMNH]; P.L. Boyd-Deep Canyon Reserve, 1.1 miles below reserve gate, 33.571489 -116.248672 ^4^, 540ft., [AUMS_3293, 21/9/1995, 1♂, T.R. Prentice, AUMNH]; Perris, 33.782519 -117.228648 ^5^, 1468ft., [AUMS_2400, 20/7/1994, 1♂, T.R. Prentice, AUMNH]; [AUMS_2409, 20/7/1994, 1♂, T.R. Prentice, AUMNH]; Perris, between I-215 and Perris Valley Airport, just N of Hwy 74 exit, 33.770547 -117.208584 ^5^, 1417ft., [AUMS_2486, 19/7/1994, 1♂, T.R. Prentice, AUMNH]; Perris, E of Perris Valley Airport, 33.774681 -117.210995 ^5^, 1419ft., [AUMS_4196, 20/7/1994, 1♂, T.R. Prentice, AUMNH]; Perris, E of Perris Valley Airport, W of I-215, 33.766645 -117.21044 ^5^, 1416ft., [AUMS_2403, 20/7/1993, 1♂, T.R. Prentice, AUMNH]; Perris, field between Perris Valley airport and 74G exit (north of exit), off I-215, 33.765075 -117.201682 ^5^, 1415ft., [AUMS_3311, 24/10/1994, 1♂, T.R. Prentice, AUMNH]; Perris, Rd 11 to I-215 between 74E exit and Perris exit, W of I-215, 33.775744 -117.239759 ^5^, 1529ft., [AUMS_2405, 20/9/1993, 1♀, W. Icenogle, AUMNH]; [AUMS_2483, 23/9/1993, 1♀, T.R. Prentice, AUMNH]; Pinyon Pines area, Palm Canyon Dr, 2 mils N of junction with Hwy 74, Santa Rosa Mtns, 33.609067 -116.471767 ^1^, 4054ft., [APH_3142, 16/8/2013, 1♂, W. Icenogle, AUMNH]; Pinyon Pines, Piñon Rd just N of junction with Hwy 74, Santa Rosa Mtns, 33.583867 -116.455383 ^1^, 4036ft., [APH_3141, 16/8/2013, 1♂, W. Icenogle, AUMNH]; S of Corona, off I-15 west side, Weirick Rd exit (Temescal Canyon), 1.5 miles W, 33.796868 -117.531405 ^5^, 1732ft., [AUMS_2685, 4/9/1995, 1♀, John Hermesman, AUMNH]; S of Hemet, NE of Lake Skinner, off De Portola Rd and Crown Valley Rd, 33.64276 -116.992 ^1^, 2307ft., [APH_1038, 27/5/2010, 1♀, Chris A. Hamilton, Tom Prentice, AUMNH]; [APH_1040, 27/5/2010, 1♀, Chris A. Hamilton, Tom Prentice, AUMNH]; S side of Lake Skinner, 33.574388 -117.054156 ^5^, 1533ft., [AUMS_2504, 11/9/1997, 1♂, Adam Bucklin, AUMNH]; San Jacinto mountains, Palms to Pines Hwy 74, above Palm Desert, 33.664336 -116.398965 ^5^, 1240ft., [AUMS_2480, 2/4/1988, 1♂, T.R. Prentice, AUMNH]; Santa Rosa Mtns, W side of Deep Canyon, Hwy 74, 1 mile S of jct with Carrizo Road, 33.5947 -116.422467 ^1^, 3733ft., [APH_3206, 17/10/2013, 1♂, W. Icenogle, AUMNH]; Santa Rosa Plateau, 33.545816 -117.270534 ^5^, 1779ft., [AUMS_2314, 25/10/1998, 1♂, Connell Dunning, AUMNH]; Santa Rosa Plateau (reserve), jct of Clinton Keith and Tenaja Rd. (bend), 33.52847 -117.273132 ^5^, 1800ft., [AUMS_2509, 29/9/1998, 1 juv, Connell Dunning, AUMNH]; Santa Rosa Plateau Ecological Reserve, Lomas Trail 1.2 miles E of Clinton Keith Rd, 33.522687 -117.271312 ^4^, 1949ft., [AUMS_2679, 29/9/1998, 1♂, C. Dunning, AUMNH]; Sun Mtns., Sun City, 33.717019 -117.216043 ^5^, 1623ft., [AUMS_2493, unknown, 1♀, T.R. Prentice, AUMNH]; Temecula, 33.493639 -117.148365 ^5^, 1047ft., [APH_2686, 8/6/1965, 1♂, Helen Wheeler, AMNH]; Temescal Canyon (Valley), N of Weirick Rd, exit off I-15, Bedford Motorway, 33.807554 -117.513595 ^5^, 1008ft., [AUMS_3321, 24/9/1995, 1♂, John Hermesman, AUMNH]; UC Riverside - backside between Ent. Annex I and Boden Lab, 33.971765 -117.325971 ^5^, 1091ft., [AUMS_2519, 13/12/1998, 1♂, T.R. Prentice, AUMNH]; Winchester, 33.706966 -117.084473 ^5^, 1486ft., [APH_2167, 18/11/1967, 1♂, W. Icenogle, AMNH]; [APH_2168, 1/10/1967, 1♂, W. Icenogle, AMNH]; [AUMS_2402, unknown, 1♀, W. Icenogle, AUMNH]; [AUMS_2478, 11/9/1990, 2♂, W. Icenogle, AUMNH]; Arabian Gardens Mobile Estates, Indio, 33.72995 -116.24115 ^4^, 9ft., [AUMS_2376, 9/1988, 1♂, Thomas R. Prentice, AUMNH]; **San Bernardino**: 1255 Colony Dr., Upland (base of Mt. Baldy), 34.1553 -117.672217 ^1^, 2102ft., [APH_2035, 7/17/2011, 1♀, Jolene Stewart, AUMNH]; 620 Greenwood Avenue, Devore, 34.242532 -117.419506 ^2^, 2690ft., [APH_0189, 25/9/2007, 1♂, Joshua Gutierrez, AUMNH]; Hwy 38, 1.8 miles W jct of Forest Falls, 34.099825 -116.98744 ^4^, 3926ft., [AUMS_2484, 24/8/1990, 1♂, W. Icenogle, AUMNH]; N of Rancho Cucamonga, off Hermosa, 34.16862 -117.58182 ^1^, 2565ft., [APH_1085, 8/10/2010, 1♂, Chris A. Hamilton, AUMNH]; N of Rancho Cucamonga, off Hermosa, 34.16742 -117.58117 ^1^, 2477ft., [APH_1086, 8/10/2010, 1♂, Chris A. Hamilton, AUMNH]; San Bernardino (San Bernardino National Forest), in hills N of city, off Quail Canyon Rd and Del Rosa, 34.174 -117.252 ^1^, 2299ft., [APH_1045, 1/6/2010, 1♀, Chris A. Hamilton, AUMNH]; **San Diego**: 0.5 W of most SW shore of Otay Lake, 32.608062 -116.939954 ^2^, 491ft., [APH_0305, 27/10/2007, 1 juv, Dorian LaPaglia, AUMNH]; 12 miles south of San Diego, 32.556071 -117.075033 ^5^, 30ft., [APH_2476, 15/10/1935, 1♂, W.J. Baerg, AMNH]; 2/10 mile W of Yaqui Pass road (S3), at the Anza-Borrego State Park sign, 3 miles S of jct with Borrego Springs Rd, 33.170767 -116.337967 ^1^, 1207ft., [APH_3209, 8/11/2013, 1♂, W. Icenogle, AUMNH]; Anza Borrego desert, on S22 (Borrego Salton Seaway) W of Salton City, 33.30525 -116.208333 ^1^, 957ft., [APH_3144, 9/11/2013, 1♂, Chris A. Hamilton, Brent E. Hendrixson, Molly Taylor, AUMNH]; Anza Borrego desert, W outside Borrego Springs off of Hwy S22, 33.228106 -116.410867 ^1^, 1523ft., [APH_3143, 9/11/2013, 1♀, Chris A. Hamilton, Brent E. Hendrixson, Molly Taylor, AUMNH]; Anza-Borrego Desert State Park, Borrego Springs, 32.908056 -116.491047 ^6^, 5115ft., [APH_2165, 31/10/1961, 1♂, Dalton E. Merkel, AMNH]; Anza-Borrego State Park, 33.039933 -116.402333 ^1^, 2584ft., [APH_0982-0984, 9/2009, 2♀, 1♂, Kyle Dickerson, AUMNH]; [APH_2697, 12/1962, 1♂, Merkel, AMNH]; [APH_2704-2705, 12/1962, 3♂, Merkel, AMNH]; [APH_2716, 11/1962, 2♂, Merkel, AMNH]; [APH_2719, 10/1962, 1♂, Merkel, AMNH]; Anza-Borrego, Yaqui Pass, 33.151285 -116.346386 ^5^, 1800ft., [AUMS_2680, 10/5/1987, 1♂, T.R. Prentice, AUMNH]; Borrego Valley, Borrego Springs Road, 8/10 mile SE of jct with Jaqui Pass road (S3), 33.204 -116.318417 ^1^, 543ft., [APH_3210, 8/11/2013, 1♂, W. Icenogle, AUMNH]; Camp Pendleton, range 313A - HOLF, 33.313998 -117.314552 ^6^, 353ft., [AUMS_2610, 20/8/unknown, 1♀, unknown, AUMNH]; Camp Pendleton, HOLF, San Mateo Creek, 33.469175 -117.475198 ^6^, 415ft., [AUMS_2611, 20/8/1999, 1♀, unknown, AUMNH]; Camp Pendleton, plot C30, 33.313998 -117.314552 ^6^, 353ft., [AUMS_2675, 30/5/1996, 1♂, T.R. Prentice, AUMNH]; Camp Pendleton, San Mateo Creek, 33.434796 -117.523894 ^6^, 321ft., [AUMS_2317, 1990, 1♂, unknown, AUMNH]; [AUMS_2644, 1999, 1♂, Dan Holland, AUMNH]; [AUMS_3328, unknown, 1♂, Dan Holland, AUMNH]; Camp Pendleton, San Onofre Cr., 33.234926 -117.389044 ^6^, 146ft., [AUMS_2582, 29/9/1999, 1♀, Dan Holland, AUMNH]; [AUMS_2258, 9/1999, 2♀, Dan Holland, AUMNH]; [AUMS_2251, 29/9/1999, 1♂, Dan Holland, AUMNH]; [AUMS_2397, 9/1999, 1♀, Dan Holland, AUMNH]; [AUMS_3348, 12/9/1999, 1♂, Dan Holland, AUMNH]; [AUMS_2315, 9/1999, 1♂, Dan Holland, AUMNH]; Chula Vista, 32.640023 -117.084004 ^5^, 67ft., [AUMS_2502, 10/8/1997, 1♀, unknown, AUMNH]; De Luz, off De Luz Rd, NW of Fallbrook, 33.42858 -117.32127 ^1^, 384ft., [APH_1037, 24/5/2010, 1♀, Chris A. Hamilton, Xavier Atkinson, AUMNH]; El Cajon, 32.794773 -116.962527 ^5^, 433ft., [APH_2147, 4/5/1926, 1♀, unknown, AMNH]; Encanto, 32.711739 -117.061755 ^5^, 230ft., [APH_2146, 18/8/1928, 1♂, unknown, AMNH]; [APH_2170, 12/11/1931, 1♂, unknown, AMNH]; Growmont, 32.799285 -116.999051 ^5^, 715ft., [APH_2169, 17/8/1931, 1♂, unknown, AMNH]; La Mesa, 32.767829 -117.023084 ^5^, 541ft., [APH_2152, 1/8/1938, 1♂, H. Stredwick, AMNH]; [APH_2162, 20/7/1925, 1♂, unknown, AMNH]; [APH_2163, 4/8/1925, 1♀, unknown, AMNH]; [APH_2164, 25/4/1927, 1♀, unknown, AMNH]; Laguna Beach , 33.542248 -117.783109 ^5^, 16ft., [APH_2158, 20/7/1930, 1♂, Edward Lawrance, AMNH]; Lake Henshaw, on Hwy 76, 33.22337 -116.75292 ^1^, 2801ft., [APH_1016-1017, 15/5/2010, 1♀, 1♂, Chris A. Hamilton, Xavier Atkinson, AUMNH]; Lake Henshaw, on Hwy 76 at Lake Henshaw campground, 33.23073 -116.76336 ^1^, 2929ft., [APH_1014-1015, 15/5/2010, 2♀, Chris A. Hamilton, Xavier Atkinson, AUMNH]; Las Flores, 33.154632 -117.207187 ^5^, 568ft., [APH_2145, 30/8/1936, 1♂, unknown, AMNH]; Lemon Grove, 32.742552 -117.031417 ^5^, 453ft., [APH_2156, 10/10/1930, 1♂, unknown, AMNH]; [APH_2157, 5/8/1926, 1♀, unknown, AMNH]; off S6/Hwy 76, E of Rincon, W of S6 turnoff for Palomar Mtn, 33.29765 -116.92304 ^1^, 2468ft., [APH_1020-1021, 15/5/2010, 2♀, Chris A. Hamilton, Xavier Atkinson, AUMNH]; Oriflamme Canyon, 2 miles W of S2, W of Anza-Borrego state park boundary, 33.004274 -116.460837 ^5^, 2246ft., [AUMS_2612, 5/1990, 1♂, T.R. Prentice, AUMNH]; San Diego, 32.71533 -117.157253 ^6^, 62ft., [APH_2148, 1935, 1♂, unknown, AMNH]; [APH_2149, 7/1962, 1♂, Chris Parrish, AMNH]; [APH_2159-2160, unknown, 1♀, 2♂, unknown, AMNH]; [APH_2161, 20/8/1925, 1♂, unknown, AMNH]; San Diego, Mission Trails Regional Park, 32.8402 -117.04561 ^1^, 349ft., [APH_1031, 20/5/2010, 1♀, Chris A. Hamilton, AUMNH]; San Diego, Mission Trails Regional Park, Oak Canyon Trail and Grassland Trail intersect, 32.84467 -117.0437 ^1^, 324ft., [APH_1009-1010, 11/5/2010, 2♀, Chris A. Hamilton, Xavier Atkinson, Kyle Dickerson, AUMNH]; Santa Ysabel, on Hwy 79, next to Santa Ysabel Open Space Preserve, 33.12607 -116.67841 ^1^, 2938ft., [APH_1018-1019, 15/5/2010, 2♀, Chris A. Hamilton, Xavier Atkinson, AUMNH]; Santee, in hills N of where Carlton Hills Blvd ends, 32.86146 -116.99485 ^4^, 780ft., [APH_0144, 5/2007, 1 juv, Gilbert Quintana, AUMNH]; Sweetwater Reserve near San Diego, 32.602142 -116.879255 ^5^, 1345ft., [APH_2473, 21/9/1935, 2♀, W.J. Baerg, AMNH]; Wruck Canyon, San Ysidro, off Cactus Rd, 32.5519 -116.99642 ^1^, 351ft., [APH_1025, 16/5/2010, 1♀, Chris A. Hamilton, Xavier Atkinson, Jordan Satler, AUMNH].

#### Distribution and natural history.

*Aphonopelma eutylenum* has a distribution that stretches from the western part of the Transverse Ranges, south down the length of the California coast along the Peninsular Ranges, and west of the Mojave Desert (Fig. 44). *Aphonopelma eutylenum* inhabits the following Level III Ecoregions in California: Southern California/Northern Baja Coast, Sonoran Basin and Range, and Southern California Mountains. This species can be found in syntopy with a number of species across its distribution including *Aphonopelma iodius*, *Aphonopelma steindachneri*, and *Aphonopelma xwalxwal*. The breeding season, when mature males abandon their burrows in search of females, occurs during the fall (generally September–November).

#### Conservation status.

*Aphonopelma eutylenum* is widely distributed across Southern California and is very common. The species is likely secure although some localized populations in urbanized areas (e.g., Los Angeles and San Diego) are likely threatened by human encroachment and development.

#### Remarks.

*Aphonopelma eutylenum* can easily be differentiated from *Aphonopelma steindachneri* and *Aphonopelma xwalxwal* by the extent of scopulation on legs III and IV, and from *Aphonopelma xwalxwal* a larger body size. Female and immature male *Aphonopelma eutylenum* can be distinguished from *Aphonopelma steindachneri* and *Aphonopelma xwalxwal* by body color as well. Other important ratios that distinguish males: *Aphonopelma eutylenum* possess a smaller T1/F3 (≤1.00; 0.93–1.00) than *Aphonopelma steindachneri* (≥1.01; 1.01–1.11) and *Aphonopelma xwalxwal* (≥1.10; 1.10–1.17); by possessing a larger T3/A3 (≥1.30; 1.30–1.35) than *Aphonopelma johnnycashi* (≤1.27; 1.18–1.27), but smaller than *Aphonopelma xwalxwal* (≥1.41; 1.41–1.64). Other important ratios that distinguish females: *Aphonopelma eutylenum* possess a larger M1/M4 (≥0.67; 0.67–0.78) than *Aphonopelma steindachneri* (≤0.67; 0.62–0.67). For both males and females, certain morphometrics have potential to be useful, though due to the amounts of variation, small number of specimens, and the small differences between species, no others are claimed to be significant at this time (see Suppl. material 2). During evaluation of traditional two-dimensional PCA morphospace, male *Aphonopelma eutylenum* separate from their syntopic species *Aphonopelma steindachneri* and *Aphonopelma xwalxwal*, but do not separate from the other species in the *iodius* species group. Interestingly, when evaluating three-dimensional PCA morphospace (PC1~PC2~PC3), male *Aphonopelma eutylenum* separates from *Aphonopelma johnnycashi*, as well as *Aphonopelma steindachneri* and *Aphonopelma xwalxwal*. Female *Aphonopelma eutylenum* separate in two-dimensional and three-dimensional morphospace from their syntopic species *Aphonopelma steindachneri* and *Aphonopelma xwalxwal*, but do not separate from the other species in the *iodius* species group. PC1, PC2, and PC3 explain ≥95% of the variation in all analyses. We examined the holotypes and freshly collected topotypic material of *Aphonopelma chambersi*, *Aphonopelma clarum*, *Aphonopelma cryptethum*, *Aphonopelma sandersoni*, and *Aphonopelma sullivani*. Our morphological and molecular analyses fail to recognize these five species as separate, independently evolving lineages. As a consequence, we consider *Aphonopelma chambersi*, *Aphonopelma clarum*, *Aphonopelma cryptethum*, *Aphonopelma sandersoni*, and *Aphonopelma sullivani* junior synonyms of *Aphonopelma eutylenum*.

Mitochondrial DNA (*CO1*) is problematic in the *iodius* species group. While this locus identifies *Aphonopelma eutylenum* as a monophyletic group, sister relationships are unclear. Nuclear DNA reveals the true evolutionary history of the *Aphonopelma eutylenum* lineage and highlights the ineffectiveness of *CO1* for accurately delimiting species boundaries within this group.

### Aphonopelma gabeli

Taxon classificationAnimaliaAraneaeTheraphosidae

Smith, 1995

**Figure 45.**
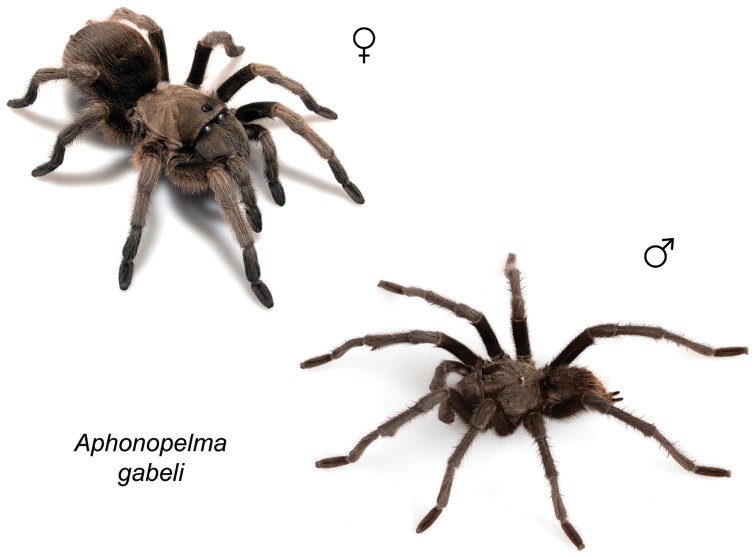
*Aphonopelma gabeli* Smith, 1995 specimens, live photographs. Female (L) - APH_1481; Male (R) - APH_0628.

**Figure 46.**
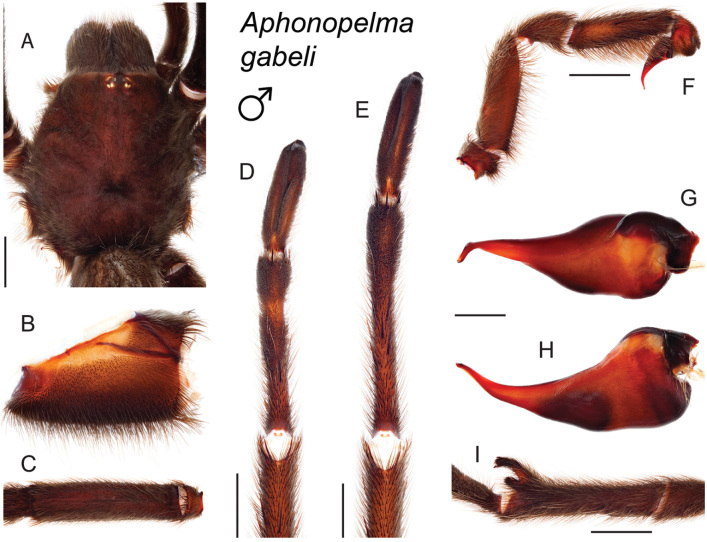
*Aphonopelma gabeli* Smith, 1995. **A–I** male specimen, APH_1054 **A** dorsal view of carapace, scale bar = 5mm **B** prolateral view of coxa I **C** dorsal view of femur III **D** ventral view of metatarsus III, scale bar = 4.5mm **E** ventral view of metatarsus IV, scale bar = 4mm **F** prolateral view of L pedipalp and palpal tibia, scale bar = 5mm **G** dorsal view of palpal bulb **H** retrolateral view of palpal bulb, scale bar = 1mm **I** prolateral view of tibia I (mating clasper), scale bar = 5mm.

**Figure 47.**
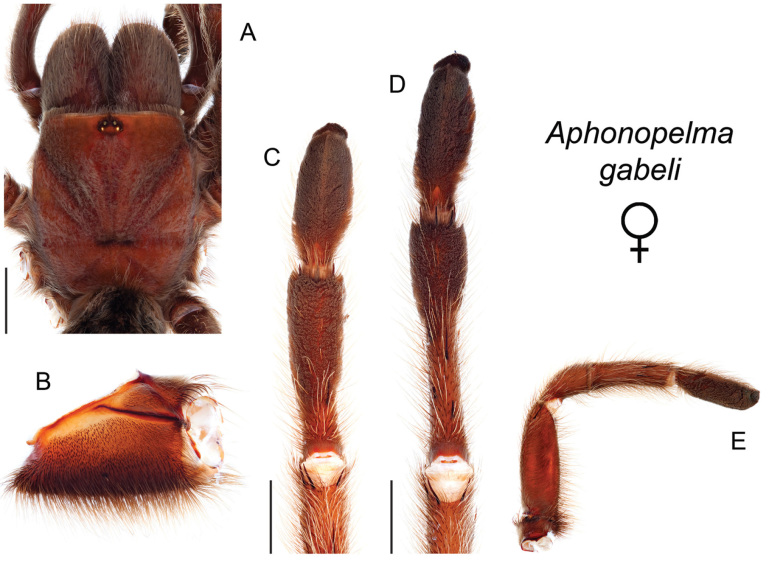
*Aphonopelma gabeli* Smith, 1995. **A–E** female specimen, APH_0680 **A** dorsal view of carapace, scale bar = 7mm **B** prolateral view of coxa I **C** ventral view of metatarsus III, scale bar = 4mm **D** ventral view of metatarsus IV, scale bar = 4mm **E** prolateral view of L pedipalp and palpal tibia.

**Figure 48.**
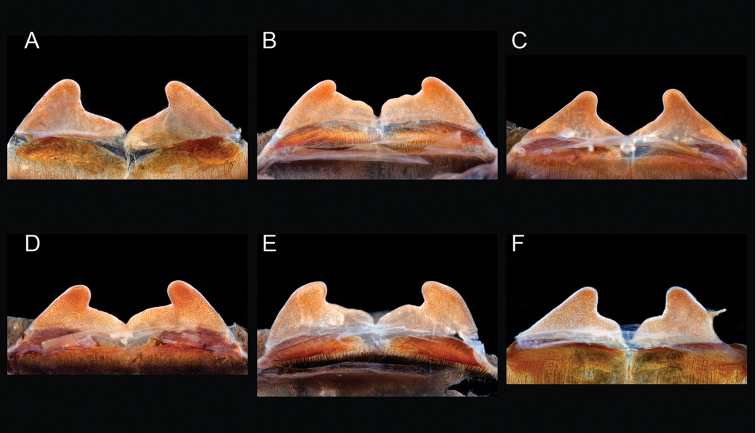
*Aphonopelma gabeli* Smith, 1995. **A–F** cleared spermathecae **A** APH_0044 **B** APH_0642 **C** APH_0680 **D** APH_0946 **E** APH_1338 **F** Jung’s “*portal*” paratype.

**Figure 49.**
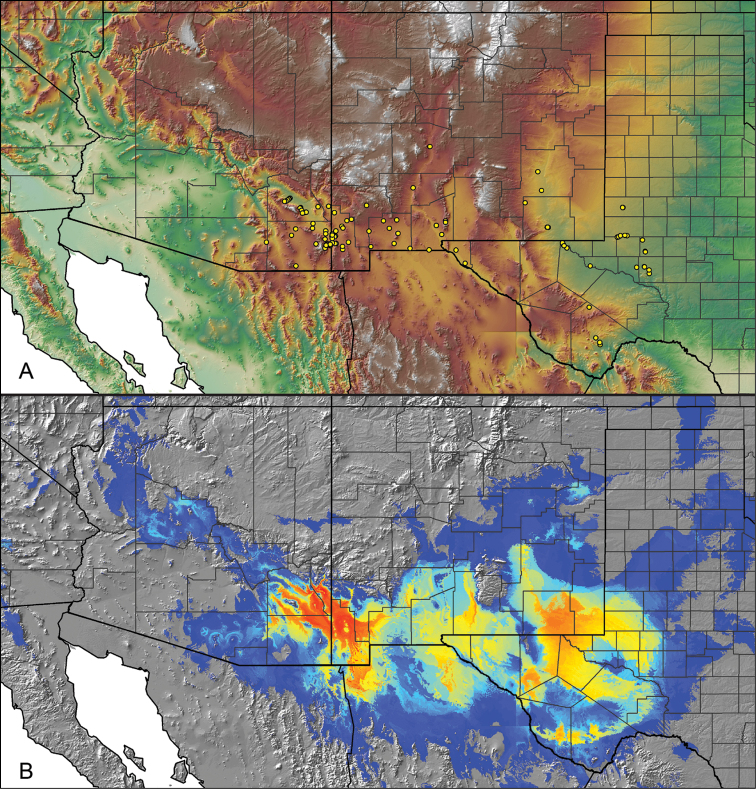
*Aphonopelma gabeli* Smith, 1995. **A** distribution of known specimens **B** predicted distribution; warmer colors (red, orange, yellow) represent areas of high probability of occurrence, cooler colors (blue shades) represent areas of low probability of occurrence.

Figures 45 , 46 , 47 , 48 , 49

Aphonopelma gabeli Smith, 1995: 100; male holotype from E of Tucson, Pima Co., Arizona, 31.956396 -110.339817^7^, elev. 4055ft., no collecting date, coll. Russ Gurley; deposited in BMNH. [examined]

#### Diagnosis.

*Aphonopelma gabeli* (Fig. 45) is a member of the *moderatum* species group and can be identified by a combination of morphological, molecular, and geographic characteristics. Nuclear and mitochondrial DNA identifies *Aphonopelma gabeli* as a phylogenetically distinct monophyletic lineage (Figs 7–8), supported as the sister lineage to *Aphonopelma moellendorfi* sp. n. and closely related to *Aphonopelma moderatum*. Female *Aphonopelma gabeli* can be distinguished from syntopic species by their unique spermathecae, noticeably large and robust chelicerae, and associated broad anterior carapace margin (Figs 45, 47). Male *Aphonopelma gabeli* have an overall black body appearance with very long, thin legs. Significant measurements that distinguish male *Aphonopelma gabeli* from its closely related phylogenetic and syntopic species are PTl and M4. Male *Aphonopelma gabeli* can be distinguished by possessing a smaller PTl/M3 (≤0.63; 0.57–0.63) than *Aphonopelma anax* (≥0.64; 0.64–0.76), *Aphonopelma armada* (≥0.65; 0.65–0.75), *Aphonopelma hentzi* (≥0.67; 0.67–0.81), *Aphonopelma chalcodes* (≥0.65; 0.65–0.75), *Aphonopelma peloncillo* sp. n. (≥0.71; 0.71–0.82), and *Aphonopelma vorhiesi* (≥0.71; 0.71–0.87); and a smaller M1/M4 (≤0.74; 0.70–0.74) than *Aphonopelma moderatum* (≥0.76; 0.76–0.81) and *Aphonopelma moellendorfi* (≥0.75; 0.75–0.82). Significant measurements that distinguish female *Aphonopelma gabeli* from its closely related phylogenetic and syntopic species are P1, M3, and extent of scopulation on metatarsus IV. Female *Aphonopelma gabeli* can be distinguished by possessing a larger M3/M4 (≥0.73; 0.73–0.78) than *Aphonopelma moderatum* (≤0.72; 0.67–0.72); a larger L4 scopulation extent (39%-53%) than *Aphonopelma peloncillo* (32%-38%) and *Aphonopelma vorhiesi* (26%-37%); a smaller L4 scopulation extent than *Aphonopelma chalcodes* (63%-81%); and by possessing a smaller P1/F4 (≤0.46; 0.42–0.46) than *Aphonopelma armada* (≥0.47; 0.47–0.51). Females of *Aphonopelma moellendorfi* are unknown and cannot be compared.

#### Description.

Male originally described by Smith (1995).

#### Redescription of male exemplar

(APH_1054; Fig. 46). *Specimen preparation and condition*: Specimen collected live crossing road, preserved in 80% ethanol; deposited in AUMNH; original coloration faded due to preservation. Left legs I, III, IV, and left pedipalp removed for measurements and photographs; stored in vial with specimen. Right leg III removed for DNA and stored at -80°C in the AUMNH (Auburn, AL). *General coloration*: Generally black or faded to brown. *Cephalothorax*: Carapace 16.79 mm long, 15.08 mm wide; densely clothed with black pubescence appressed to surface; fringe covered in long setae not closely appressed to surface; foveal groove deep and straight; pars cephalica region rises gradually from foveal groove, gently arching anteriorly toward ocular area; AER slightly procurved, PER recurved; normal sized chelicerae; clypeus extends slightly forward; LBl 1.90, LBw 2.05; sternum hirsute, clothed with short and medium length black, densely packed setae. *Abdomen*: Densely clothed in short black/brown pubescence with numerous longer red/orange setae interspersed; possessing a dense dorsal patch of black Type I urticating bristles (Cooke et al. 1972). *Legs*: Hirsute; densely clothed in a mix of short black/brown pubescence with longer ventral setae. Metatarsus I slightly curved. F1 16.15; F1w 3.65; P1 6.31; T1 12.7; M1 11.71; A1 7.98; F3 13.75; F3w 4.01; P3 5.53; T3 10.31; M3 13.01; A3 7.87; F4 16.26; F4w 3.75; P4 5.95; T4 12.76; M4 16.60; A4 8.55; femur III is normal - not noticeably swollen or wider than other legs. All tarsi fully scopulate. Extent of metatarsal scopulation: leg III (SC3) = 67.5%; leg IV (SC4) = 47.1%. Two ventral spinose setae on metatarsus III; five ventral spinose setae on metatarsus IV. *Coxa I*: Prolateral surface a mix of fine, hair-like and tapered setae. *Pedipalps*: Hirsute; densely clothed in the same setal color as the other legs, with numerous longer ventral setae; one spinose seta on the apical, prolateral femur and six spinose setae on the prolateral tibia; PTl 7.963, PTw 2.590. When extended, embolus tapers but quickly curves to the retrolateral side near apex; embolus very slender, no keels.

**Variation (6).** Cl 15.21–16.79 (15.667±0.24), Cw 13.4–15.08 (14.075±0.25), LBl 1.7–2.02 (1.872±0.06), LBw 1.91–2.27 (2.075±0.05), F1 15.63–17.21 (16.127±0.23), F1w 3.41–3.65 (3.518±0.04), P1 5.53–6.37 (6.022±0.13), T1 12.7–13.55 (13.067±0.12), M1 11.71–12.98 (12.328±0.18), A1 7.64–8.56 (7.953±0.14), L1 length 54.27–57.46 (55.497±0.44), F3 13.4–14.24 (13.665±0.13), F3w 3.36–4.01 (3.723±0.09), P3 4.67–5.53 (5.253±0.13), T3 10.02–10.89 (10.472±0.13), M3 12.86–13.88 (13.28±0.14), A3 7.33–7.91 (7.635±0.1), L3 length 48.39–51.87 (50.305±0.46), F4 14.81–17.47 (15.853±0.38), F4w 3.44–3.81 (3.592±0.06), P4 4.79–6.02 (5.423±0.2), T4 12.64–13.39 (12.985±0.12), M4 16.33–18.26 (17.022±0.28), A4 8.09–9.14 (8.673±0.15), L4 length 57.18–63.65 (59.957±0.88), PTl 7.677–8.48 (8.083±0.11), PTw 2.38–2.59 (2.525±0.03), SC3 ratio 0.591–0.721 (0.663±0.02), SC4 ratio 0.361–0.471 (0.416±0.02), Coxa I setae = tapered, F3 condition = normal.

#### Description of female exemplar

(APH_0680; Figs 47–48). *Specimen preparation and condition*: Specimen collected live from burrow, preserved in 80% ethanol; deposited in AUMNH; original coloration faded due to preservation. Left legs I, III, IV, and pedipalp removed for photographs and measurements; stored in vial with specimen. Right leg III removed for DNA and stored at -80°C in the AUMNH (Auburn, AL). Genital plate with spermathecae removed and cleared, stored in vial with specimen. *General coloration*: Faded brown, black, and grey, medium length setae cover body; brownish-grey, with green tint following a molt (*in situ*). *Cephalothorax*: Carapace 18.34 mm long, 15.81 mm wide; densely clothed with brown pubescence closely appressed to surface; fringe densely covered in medium setae; broad anterior margin of carapace; foveal groove medium deep and straight; pars cephalica region rises from thoracic furrow more steeply than male, arching anteriorly toward ocular area; AER very slightly procurved, PER recurved; large, robust chelicerae; clypeus extends forward on a slight curve; LBl 2.38, LBw 2.44; sternum hirsute, clothed with brown, medium length setae. *Abdomen*: Densely clothed dorsally in short black setae with numerous longer, lighter setae interspersed (generally red or orange *in situ*); dense dorsal patch of black Type I urticating bristles (Cooke et al. 1972); ventral side with shorter dark brown setae. *Spermathecae*: Uniquely shaped; paired and separate that taper to a pocket, with wide bases that are not fused. *Legs*: Hirsute, particularly ventrally; densely clothed in short, brown pubescence with longer setae interspersed. F1 15.24; F1w 4.26; P1 6.26; T1 12.11; M1 10.20; A1 7.57; F3 12.72; F3w 3.58; P3 6.26; T3 8.88; M3 10.71; A3 7.73; F4 14.9; F4w 3.83; P4 6.55; T4 12.03; M4 13.75; A4 8.49. All tarsi fully scopulate. Extent of metatarsal scopulation: leg III (SC3) = 79.2%; leg IV (SC4) = 52.9%. One ventral spinose seta on metatarsus III; four ventral spinose setae on metatarsus IV. *Coxa I*: Prolateral surface a mix of fine, hair-like and tapered setae. *Pedipalps*: Densely clothed in the same setal color as the other legs; one spinose seta on the apical, prolateral femur and two spinose setae on the prolateral tibia.

**Variation (6).** Cl 14.69–18.34 (16.535±0.53), Cw 12.65–15.81 (14.145±0.5), LBl 2.07–2.41 (2.29±0.06), LBw 2.29–2.82 (2.485±0.08), F1 12.13–15.24 (13.288±0.45), F1w 3.67–4.26 (3.917±0.09), P1 5.41–6.26 (5.772±0.15), T1 9.35–12.11 (10.285±0.4), M1 7.41–10.2 (8.33±0.43), A1 5.83–7.57 (6.647±0.24), L1 length 40.18–51.38 (44.322±1.6), F3 9.63–12.72 (10.783±0.44), F3w 3.44–3.76 (3.575±0.05), P3 4.27–6.26 (5.053±0.28), T3 7.08–8.88 (7.773±0.26), M3 7.78–10.71 (8.75±0.41), A3 6.09–7.73 (6.593±0.24), L3 length 35.81–46.30 (38.953±1.56), F4 12.21–14.90 (13.065±0.41), F4w 3.43–3.83 (3.642±0.06), P4 4.67–6.55 (5.553±0.27), T4 9.31–12.03 (10.187±0.4), M4 10.5–13.75 (11.663±0.48), A4 6.71–8.49 (7.168±0.28), L4 length 43.83–55.72 (47.637±1.7), SC3 ratio 0.725–0.805 (0.762±0.01), SC4 ratio 0.397–0.529 (0.471±0.02), Coxa 1 setae = tapered. Spermathecae variation can be seen in Figure 48.

#### Material examined.

**United States: Arizona: Cochise**: 0.1 mi. west of Portal, 31.913699 -109.143184 ^4^, 4780ft., [APH_2356, 2/7/1961, 1♂, J. Cole, AMNH]; 0.5 miles east of Portal, 31.914884 -109.149967 ^4^, 4846ft., [APH_2364, 1/7/1961, 1♂, J. Cole, AMNH]; 1 mile southwest of Portal, 31.903762 -109.152806 ^5^, 4918ft., [APH_2363, 2/9/1960, 1♀, R. Zweifel, AMNH]; 1 mile west of Portal, 31.9135 -109.158499 ^5^, 4961ft., [APH_2365, 4/7/1963, 1♂, Steve Aaron, AMNH]; 1.7 miles northeast of Portal on San Simon Rd., 31.930194 -109.12083 ^4^, 4570ft., [APH_2377, 21/7/1961, 1♂, J. Cole, AMNH]; 15.5 miles S I-10 on Noland Rd, 32.032719 -109.186732 ^1^, 4478ft., [APH_1337-1338, 2/8/2011, 2♀, Brent E. Hendrixson, Brendon Barnes, Nate Davis, Jake Storms, AUMNH]; 2 miles northeast of Portal, 31.934159 -109.117117 ^5^, 4534ft., [APH_2380, 10/6/1963, 1♀, Cazier and Mortenson, AMNH]; 2.5 miles southeast of Portal on Portal Rd., 31.902146 -109.10097 ^4^, 4478ft., [APH_2372, 6/7/1973, 1♂, R. Zweifel, AMNH]; 2.6 miles NW AZ/NM state line on Portal Rd, 31.89302489 -109.0856873 ^1^, 4349ft., [APH_0385, 31/7/2008, 1♂, Alice Abela, AUMNH]; 7.7 miles S I-10 on Noland Rd, 32.145844 -109.173364 ^1^, 3872ft., [APH_1336, 2/8/2011, 1♀, Brent E. Hendrixson, Brendon Barnes, Nate Davis, Jake Storms, AUMNH]; Apache Pass Rd, S of I-10, 32.267466 -109.464574 ^1^, 3854ft., [APH_0712, 20/7/2009, 1 juv, Brent E. Hendrixson, Nate Davis, AUMNH]; Cave Creek Canyon, 1.3 miles northeast of Ranger Station on Cave Creek Canyon Rd. near Portal, 31.896202 -109163672 ^4^, 4961ft., [APH_2383, 11/7/1973, 1♂, A. Bush, AMNH]; Chiricahua Mtns, 31.929812 -109.382285 ^6^, 6119ft., [APH_2367, 8/1972, 2♂, J.A.L. Cooke, AMNH]; Dos Cabezas, 32.114138 -109.920729 ^5^, 4219ft., [APH_2378, 19/9/1954, 1♂, G. Bradt, M. Oazier, AMNH]; Fan Road, 5.5 miles NE Bowie, 32.375306 -109.447667 ^1^, 3640ft., [APH_0393, 24/7/2008, 1♂, Kari and Hunter McWest, AUMNH]; Portal, 31.913699 -109.1414 ^5^, 4770ft., [APH_2357, 8/7/1964, 2♂, D. Rich, AMNH]; [APH_2359, 1/8/1965, 1♂, W.J. Gerstch, AMNH]; [APH_2360, 25/6/1962, 1♂, W.J. Gerstch, AMNH]; [APH_2362, 10/7/1962, 1♀, Melinda Stebbins, AMNH]; [APH_2368, 4/7/1961, 1♂, J. Cole, AMNH]; [APH_2369, 15/8/1962, 1♀, C. Parrish and W.J. Gertsch, AMNH]; [APH_2381, 14/6/1962, 1♀, W.J. Gerstch, AMNH]; Portal Rd, 31.884644 -109.071997 ^5^, 4250ft., [APH_0390, unknown, 1♂, Alice Abela, AUMNH]; S of I-10 on Noland Rd, 32.213729 -109.176179 ^1^, 3645ft., [APH_0711, 20/7/2009, 1 juv, Brent E. Hendrixson, Nate Davis, AUMNH]; Sulphur Canyon, 9 miles west of Rodeo, 31.836758 -109.188226 ^5^, 7060ft., [APH_2355, 23/7/1955, 1♂, Guy Miller, AMNH]; Wilcox, 32.252851 -109.83201 ^5^, 4170ft., [APH_2382, 12/7/1954, 1♂, W.J. Gerstch, AMNH]; **Graham**: 0.25 miles E Hwy-191 on Tanque Rd, 32.605235 -109.682418 ^1^, 3873ft., [APH_1329-1330, 1/8/2011, 2♀, Brent E. Hendrixson, Brendon Barnes, Nate Davis, Jake Storms, AUMNH]; [APH_1481-1482, 1/8/2012, 1♀, 1♂, Brent E. Hendrixson, Brendon Barnes, Austin Deskewies, AUMNH]; [APH_1489, 4/9/2012, 1♀, Brent E. Hendrixson, AUMNH]; 0.4 miles E Hwy-191 on Tanque Rd, 32.606204 -109.681524 ^1^, 3891ft., [APH_1184, 25/7/2010, 1♀, Brent E. Hendrixson, Brendon Barnes, Nate Davis, AUMNH]; 3.6 miles E Hwy-191 on Tanque Rd, 32.619274 -109.633622 ^1^, 3623ft., [APH_1231, 8/8/2010, 1♂, Brent E. Hendrixson, Ashley Bailey, Andrea Reed, AUMNH]; 5.8 miles E Hwy-191 on Tanque Rd, 32.621499 -109.596498 ^1^, 3481ft., [APH_1236, 8/8/2010, 1♂, Brent E. Hendrixson, Ashley Bailey, Andrea Reed, AUMNH]; along Hwy-191, 32.662275 -109.701131 ^1^, 3530ft., [APH_1179, 25/7/2010, 1♂, Brent E. Hendrixson, Brendon Barnes, Nate Davis, AUMNH]; dirt road S of US-70, 32.744869 -109.344099 ^1^, 4088ft., [APH_0641-0642, 12/7/2009, 2♀, Brent E. Hendrixson, Nate Davis, AUMNH]; Hwy-366, near Hwy-191, 32.726066 -109.71822 ^1^, 3266ft., [APH_0637-0638, 11/7/2009, 2♂, Brent E. Hendrixson, Nate Davis, AUMNH]; Klondyke Rd, SW of Hwy-70, 32.914146 -109.975734 ^1^, 3110ft., [APH_0698-0699, 18/7/2009, 2 juv, Brent E. Hendrixson, Nate Davis, AUMNH]; [APH_0701-0708, 18/7/2009, 2♀, 6♂, Brent E. Hendrixson, Nate Davis, AUMNH]; Tanque Rd, near Hwy-191, 32.604126 -109.681695 ^1^, 3887ft., [APH_0627-0632, 11/7/2009, 5♂, 1 juv, Brent E. Hendrixson, Nate Davis, AUMNH]; [APH_0635, 11/7/2009, 1♀, Brent E. Hendrixson, Nate Davis, AUMNH]; **Greenlee**: 0.4 miles N Hwy-75 on Goat Camp Rd, 32.755403 -109.110492 ^1^, 3726ft., [APH_1351, 5/8/2011, 1 juv, Brent E. Hendrixson, Brendon Barnes, Nate Davis, Jake Storms, AUMNH]; **New Mexico: Chaves**: N of Roswell, 33.50677 -104.52311 ^5^, 3626ft., [APH_0316, 10/7/2007, 1♀, Rick C. West, AUMNH]; rest area near Hagerman, near NM-249, 33.09694 -104.44167 ^2^, 3559ft., [APH_0044, 20/6/2002, 1♀, Shasta Michaels, JJ East, AUMNH]; **Dona Ana**: 0.9 miles NE I-10 on CR-B19 (I-10 Exit 155), 32.130859 -106.626039 ^1^, 4030ft., [APH_0538, 5/6/2009, 1♀, Brent E. Hendrixson, Courtney Dugas, Sloan Click, AUMNH]; 2711 Los Misioneros, Las Cruces, 32.336008 -106.749999 ^2^, 4170ft., [APH_1461, 27/6/2012, 1♂, Jesse Ortiz, AUMNH]; Aguirre Springs Rd, 32.43067 -106.547785 ^1^, 5258ft., [APH_0655, 13/7/2009, 1 juv, Brent E. Hendrixson, Nate Davis, AUMNH]; [APH_0662-0664, 14/7/2009, 3 juv, Brent E. Hendrixson, Nate Davis, AUMNH]; along Hwy-9, 31.79866 -106.907625 ^1^, 4113ft., [APH_0653, 13/7/2009, 1 juv, Brent E. Hendrixson, Nate Davis, AUMNH]; Just outside Las Cruces, on Aguirre Spring Road, 32.431202 -106.54921 ^5^, 5306ft., [APH_0002, 8/2003, 1♂, Roy Thibodeau, AUMNH]; **Eddy**: 0.5 miles W US-62/180 on CR 408 (Dark Canyon Rd), 32.28746 -104.28966 ^1^, 3330ft., [APH_0366-0368, 22/6/2008, 3♂, Shasta Michaels, AUMNH]; 1.2 miles W US-62/180 on CR 408 (Dark Canyon Rd), 32.28969 -104.30144 ^1^, 3350ft., [APH_0364-0365, 21/6/2008, 2♂, Shasta Michaels, AUMNH]; 1.7 miles W US-62/180 on CR-408, 32.293874 -104.309514 ^1^, 3392ft., [APH_0542-0543, 6/6/2009, 2 juv, Brent E. Hendrixson, Courtney Dugas, Sloan Click, AUMNH]; 2.3 miles W US-62/180 on CR 408, 32.29345 -104.31993 ^1^, 3434ft., [APH_0061, 14/6/2001, 1♀, Brent E. Hendrixson, AUMNH]; near jct. CR-1 and US-82, 32.830265 -104.797078 ^1^, 4228ft., [APH_0541, 6/6/2009, 1♀, Brent E. Hendrixson, Courtney Dugas, Sloan Click, AUMNH]; **Grant**: Silver City, pinyon pine habitat, 32.770075 -108.280326 ^4^, 5944ft., [APH_1293, 7/2011, 1 juv, Ken McNeil, AUMNH]; **Hidalgo**: 1.6 miles S I-10 on Hwy-80, 32.213781 -108.951553 ^1^, 4233ft., [APH_1180, 26/7/2010, 1♂, Brent E. Hendrixson, Brendon Barnes, Nate Davis, AUMNH]; 15.5 miles north of Rodeo, 32.049697 -109.030541 ^5^, 3934ft., [APH_2376, 11/7/1960, 1♂, Zweifel, AMNH]; 18 miles north of Rodeo, 32.089638 -109.036588 ^5^, 3976ft., [APH_2361, 7/7/1956, 1♂, H. Howden, AMNH]; 19.5 miles north of Rodeo, 32.11859 -109.03127 ^5^, 4042ft., [APH_2385, 11/7/1960, 1♂, Zweifel, AMNH]; 21.5 miles north of Rodeo, 32.141862 -109.028611 ^5^, 4124ft., [APH_2371, 11/7/1960, 1♂, Zweifel, AMNH]; 4.1 miles E Hwy-80 on Hwy-9, 31.936704 -108.970448 ^1^, 4187ft., [APH_0678, 16/7/2009, 1♀, Brent E. Hendrixson, Nate Davis, AUMNH]; 5 miles north of junction of Animas Rd. and rt. 80, 31.443254 -109.827763 ^5^, 4678ft., [APH_2374, 23/7/1960, 1♂, R. Zweifel, AMNH]; 5 miles south of Road Forks, 32.275308 -108.794078 ^5^, 4354ft., [APH_2384, 2/7/1962, 1♂, W.J. Gerstch, AMNH]; 6 miles north of Lordsburg, 32.436395 -108.707975 ^5^, 4432ft., [APH_2358, 28/7/1962, 1♂, unknown, AMNH]; 8 miles north of Rodeo, 31.952008 -109.030121 ^5^, 4078ft., [APH_2370, 14/7/1963, 1♂, C. Bagwell, AMNH]; along Hwy-338, 31.807343 -108.801127 ^1^, 4675ft., [APH_1192-1194, 26/7/2010, 3♂, Brent E. Hendrixson, Brendon Barnes, Nate Davis, AUMNH]; along Hwy-80 at CR-C078, 32.101572 -108.957714 ^1^, 4408ft., [APH_0676, 15/7/2009, 1 juv, Brent E. Hendrixson, Nate Davis, AUMNH]; along Hwy-80 at Granite Gap, 32.086958 -108.977019 ^1^, 4495ft., [APH_1483, 1/8/2012, 1♂, Brent E. Hendrixson, Brendon Barnes, Austin Deskewies, AUMNH]; along Hwy-9, just E of Continental Divide, 31.963316 -108.673824 ^1^, 4489ft., [APH_0680, 16/7/2009, 1♀, Brent E. Hendrixson, Nate Davis, AUMNH]; along Hwy-9, just west of Gas Line Rd, 31.935932 -108.940821 ^1^, 4355ft., [APH_0677, 15/7/2009, 1♂, Brent E. Hendrixson, Nate Davis, AUMNH]; along Hwy-90, NE of Lordsburg, 32.469585 -108.60797 ^1^, 5005ft., [APH_0646, 12/7/2009, 1 juv, Brent E. Hendrixson, Nate Davis, AUMNH]; along US-70, SE of Hwy-92, 32.612037 -108.984613 ^1^, 4151ft., [APH_0643-0644, 12/7/2009, 1♀, 1 juv, Brent E. Hendrixson, Nate Davis, AUMNH]; Cieanega Lake, 15.5 miles north of Rodeo, 32.049697 -109.030541 ^5^, 3934ft., [APH_2375, 14/7/1961, 1♂, J. Cole, AMNH]; Lordsburg, vicinity of Fraggle Rock, 32.31738 -108.81833 ^4^, 4250ft., [APH_1294, 7/2011, 1♂, Ken McNeil, AUMNH]; Rodeo, 31.950087 -109.031176 ^5^, 4085ft., [APH_2366, 17/7/1963, 2♂, V. Roth, AMNH]; Rt. 9, 2 miles east of juncture with US 80, 31.920498 -109.070014 ^4^, 4301ft., [APH_2387, 3/7/1958, 2♂, Robert Chew, AMNH]; **Luna**: 0.5 miles E Hidalgo Co. Line along I-10, 32.208983 -108.221098 ^1^, 4547ft., [APH_1178, 23/7/2010, 1♂, Brent E. Hendrixson, Brendon Barnes, Nate Davis, AUMNH]; along Hwy-11, N of Columbus, 31.935144 -107.670986 ^1^, 4220ft., [APH_0650-0651, 13/7/2009, 2 juv, Brent E. Hendrixson, Nate Davis, AUMNH]; along Hwy-180, 32.431967 -107.90736 ^1^, 4691ft., [APH_0647-0649, 13/7/2009, 3 juv, Brent E. Hendrixson, Nate Davis, AUMNH]; along Hwy-9, 31.828861 -107.320841 ^1^, 4141ft., [APH_0652, 13/7/2009, 1 juv, Brent E. Hendrixson, Nate Davis, AUMNH]; along Hwy-9, 2.3 miles SE Grant County Line, 31.868935 -108.182075 ^1^, 4605ft., [APH_0665, 14/7/2009, 1 juv, Brent E. Hendrixson, Nate Davis, AUMNH]; Cookes Canyon Rd, A019, 3.4 miles NW NM-26, 32.453472 -107.614333 ^1^, 4710ft., [APH_0395, 28/7/2008, 1♂, Kari and Hunter McWest, AUMNH]; Deming, on ramp to I-10, 32.267886 -107.780667 ^1^, 4817ft., [APH_1177, 23/7/2010, 1♂, Brent E. Hendrixson, Brendon Barnes, Nate Davis, AUMNH]; Little Florida Mtns, 1 miles SE on Bonita Rd from turnoff on Gap Rd, 32.15817 -107.58858 ^4^, 4480ft., [APH_0194, 3/9/2007, 1♀, Lorenzo Prendini, Jeremy Huff, AUMNH]; **Sierra**: near Truth or Consequences, 0.2 miles W I-25 off Exit 79, 33.157129 -107.257152 ^1^, 4491ft., [APH_1295, 24/7/2011, 1 juv, Brent E. Hendrixson, Shasta Michaels, AUMNH]; **Socorro**: Escondida, 34.05833 -106.89083 ^5^, 4607ft., [APH_0034, 7/8/2006, 1♀, Kristin Greene, AUMNH]; **Texas: Andrews**: SW4001, 32.113981 -102.615814 ^1^, 3139ft., [APH_1053-1054, 6/7/2010, 2♂, Skyler Stevens, AUMNH]; SW7000 and SW6601, 32.110219 -102.710939 ^1^, 3233ft., [APH_1055, 6/7/2010, 1♂, Skyler Stevens, AUMNH]; SW8000 and SW3001, 32.111511 -102.566667 ^1^, 3122ft., [APH_1050-1051, 6/7/2010, 2♂, Skyler Stevens, AUMNH]; **Brewster**: 1.85 miles N Ranch Rd 2627 on Hwy-385, 29.71843 -103.15894 ^2^, 2759ft., [APH_1469, 22/6/2012, 1♂, Darryl Burton, AUMNH]; 14 miles N Ranch Rd 2627 on Hwy-385, 29.86536 -103.24919 ^2^, 3215ft., [APH_1472, 24/6/2012, 1♂, Darryl Burton, AUMNH]; 14.3 miles NE jct US-90 on US-67, 30.53666 -103.39322 ^1^, 3831ft., [APH_0029, 18/6/2001, 1♂, Jeff Owens, AUMNH]; 5.5 miles N Ranch Rd 2627 on Hwy-385, 29.76887 -103.16619 ^2^, 2840ft., [APH_1471, 24/6/2012, 1♂, Darryl Burton, AUMNH]; **Crane**: off Hwy 385, N or Crane, 31.41690278 -102.3563111 ^2^, 2550ft., [APH_1386, 29/8/2011, 1 juv, Darryl Burton, AUMNH]; **Ector**: Cowden H Ranch, 32.07805 -102.780783 ^6^, 3316ft., [APH_0940, 2006, 1♀, Dave Moellendorf, AUMNH]; [APH_0943, 9/2008, 1♀, Chris A. Hamilton, AUMNH]; [APH_0946, 9/2008, 1♀, Chris A. Hamilton, AUMNH]; **El Paso**: 3457 Red Sails Drive, El Paso, 31.79305 -106.313143 ^1^, 3987ft., [APH_3126, 18/7/2013, 1♂, Jackie Ortegon, AUMNH]; Rest Area, 1.4 miles SE FM-793 (SE Fabens), 31.503951 -106.116307 ^1^, 3779ft., [APH_0537, 5/6/2009, 1♀, Brent E. Hendrixson, Courtney Dugas, Sloan Click, AUMNH]; **Gaines**: Seminole, 32.72695 -102.660533 ^1^, 3327ft., [APH_0850-0854, 9/2008, 5♀, Chris A. Hamilton, AUMNH]; [APH_0888, 9/2008, 1♀, Chris A. Hamilton, AUMNH]; Seminole, 0.4 miles NW 11th St on Hwy-214, 32.729362 -102.661669 ^1^, 3329ft., [APH_0545, 7/6/2009, 1♀, Brent E. Hendrixson, Courtney Dugas, Sloan Click, AUMNH]; **Midland**: CR60, 32.010556 -102.2275 ^1^, 2888ft., [APH_1060, 2/7/2010, 1♂, Skyler Stevens, AUMNH]; FM 1788 S, 31.762925 -102.16813 ^2^, 2895ft., [APH_1372-1373, 27/6/2011, 2♂, Darryl Burton, AUMNH]; **Reeves**: outside Carlsbad, 31.83806 -103.88556 ^1^, 2780ft., [APH_0018-0021, 20/6/2002, 4♂, ATS Conference, AUMNH]; **Upton**: just S of Hwy 329, before FM1492 jct, 31.42758333 -102.1889667 ^2^, 2800ft., [APH_1378, 10/8/2011, 1 juv, Darryl Burton, AUMNH]; oil fields E of Hwy 329, 31.35481944 -102.0862667 ^2^, 2715ft., [APH_1382, 10/9/2011, 1♀, Darryl Burton, AUMNH]; [APH_1383, 30/8/2011, 1♀, Darryl Burton, AUMNH]; oil fields W of Hwy 329, 31.42420833 -102.1828 ^2^, 2795ft., [APH_1376, 30/7/2011, 1 juv, Darryl Burton, AUMNH]; [APH_1380, 6/9/2011, 1 juv, Darryl Burton, AUMNH]; [APH_1390, 31/7/2011, 1 juv, Darryl Burton, AUMNH]; **Ward**: E of Pecos on I-20, 31.444196 -103.371451 ^1^, 2583ft., [APH_1175, 22/7/2010, 1♂, Brent E. Hendrixson, Brendon Barnes, Nate Davis, AUMNH.

#### Distribution and natural history.

*Aphonopelma gabeli* is distributed mostly throughout the Chihuahuan Desert in southeastern Arizona, southern New Mexico, and West Texas; this includes the northern finger-like extensions of the desert along the Rio Grande and Pecos Rivers into Socorro and Chaves Counties, New Mexico, respectively (Fig. 49A). This species is also known from adjacent sections of the High Plains and near canyon mouths of the Madrean sky islands. The species distribution model (Fig. 49B) predicts suitable habitat throughout most of Trans-Pecos Texas, the boot heel of New Mexico, and sections of southeastern Arizona, northeastern Sonora, and northwestern Chihuahua. *Aphonopelma gabeli* can be found inhabiting the following Level III Ecoregions: Chihuahuan Deserts, High Plains, Arizona/New Mexico Mountains, Southwestern Tablelands, and Madrean Archipelago (Fig. 1F). Specimens accompanied with precise georeferenced locality data have been collected at elevations ranging from 775 to 1620 meters in short grass prairie, desert grassland, and desert scrub communities; at higher elevations, habitats are sometimes associated with various oaks and junipers. *Aphonopelma gabeli* has been observed in syntopy (burrows located within a few meters of each other) with *Aphonopelma armada*, *Aphonopelma hentzi*, *Aphonopelma parvum* sp. n., and *Aphonopelma vorhiesi* and can probably be found alongside *Aphonopelma chalcodes* in Cochise and Graham Counties, Arizona. Burrows are typical of that for North American tarantulas (i.e., circular and generally covered by a thin veil of silk) and specimens can be readily collected by pouring a small amount of water into their burrows. Burrows are plugged during the winter months. The breeding period is late spring and early summer (June-August); adult males have been observed in large numbers at night along dirt roads in Graham County, Arizona and Eddy County, New Mexico. The data in Hamilton et al. (2011) and other preliminary mtDNA data suggests that *Aphonopelma gabeli* recently expanded its distribution into present-day portions of the Chihuahuan Desert, likely following the desert’s appearance in the United States as the climate warmed and dried during the late Holocene (see Van Devender 1990).

#### Conservation status.

Landscape fragmentation due to oil and natural gas production has raised concern about the conservation status of some species in the Permian Basin of West Texas and southeastern New Mexico (Leavitt and Fitzgerald 2013). *Aphonopelma gabeli*, however, is one of the most common and widely distributed tarantulas in the United States and is frequently encountered in areas that have been developed for such production (D. Burton 2011, pers. comm.). This species is secure.

#### Remarks.

*Aphonopelma gabeli* females and juvenile males are easily differentiated from *Aphonopelma armada* by the flared metatarsal scopulae and prolateral coxa I setae of *Aphonopelma armada*; from *Aphonopelma anax* by spermathecae and palpal bulbs, as well as prolateral coxa I setae; from *Aphonopelma hentzi* by its phenotypic appearance and prolateral coxa I setae; from *Aphonopelma moderatum* by their unique phenotypic color and banding; from *Aphonopelma peloncillo* by its phenotypic appearance; from *Aphonopelma vorhiesi* by their black appearance; and from *Aphonopelma parvum* due to the extreme small size of the miniature species. Other important ratios that distinguish males: *Aphonopelma gabeli* possess a larger F4L/W (≥4.29; 4.29–4.60) than *Aphonopelma hentzi* (≤4.24; 3.62–4.24); by possessing a smaller L1/L4 (≤0.95; 0.91–0.95) than *Aphonopelma moderatum* (>0.95; 0.95–0.99) and *Aphonopelma moellendorfi* (≥0.96; 0.96–1.00); by possessing a larger L4 scopulation extent (36%–47%) than *Aphonopelma vorhiesi* (20%-36%) and smaller than *Aphonopelma chalcodes* (66%–76%); by possessing a smaller F1/M3 (≤1.24; 1.18–1.24) than *Aphonopelma anax* (≥1.28; 1.28–1.43), *Aphonopelma armada* (≥1.26; 1.26–1.33), and *Aphonopelma peloncillo* (≥1.33; 1.33–1.49). Other important ratios distinguish females: *Aphonopelma gabeli* possess a larger T3/T4 (≥0.73; 0.73–0.79) than *Aphonopelma armada* (≤0.73; 0.64–0.73) and *Aphonopelma moderatum* (≤0.71; 0.63–0.71); by possessing a larger L3 scopulation extent (72%-80%) than *Aphonopelma peloncillo* (58%–68%) and *Aphonopelma vorhiesi* (49%–69%); by possessing a larger CL/CW (≥1.14; 1.14–1.20) than *Aphonopelma chalcodes* (≤1.14; 1.09–1.14). For both males and females, certain morphometrics have potential to be useful, though due to the amounts of variation, small number of specimens, and the small differences between species, no others are claimed to be significant at this time (see Suppl. material 2). During evaluation of traditional PCA morphospace, males of *Aphonopelma gabeli* separate from *Aphonopelma anax*, *Aphonopelma armada*, *Aphonopelma hentzi*, and *Aphonopelma vorhiesi* along PC1~2, but do not separate from *Aphonopelma moderatum*, *Aphonopelma moellendorfi*, *Aphonopelma chalcodes*, or *Aphonopelma peloncillo*. Female *Aphonopelma gabeli* do not separate from any of their syntopic species or phylogenetic sister lineages in PCA morphospace. Females of *Aphonopelma moellendorfi* are unknown and cannot be compared. Interestingly, *Aphonopelma gabeli* males separate from *Aphonopelma anax*, *Aphonopelma armada*, and *Aphonopelma hentzi* in three-dimensional PCA morphospace (PC1~PC2~PC3), but do not separate from *Aphonopelma moderatum* and *Aphonopelma moellendorfi*. *Aphonopelma gabeli* females separate from *Aphonopelma anax*, but do not separate from *Aphonopelma armada*, *Aphonopelma hentzi*, and *Aphonopelma moderatum*. PC1, PC2, and PC3 explain ≥87% of the variation in male analyses and ≥96% of the variation in female analyses. It is also important to note the tremendous variation in spermathecae shape that can be seen across *Aphonopelma gabeli* populations (Fig. 48). Previous taxonomic work considered this variation enough to split and describe separate species; this is clearly not an effective character due to the large amounts of subtle variation that is possible. This species was first identified as a new species by Jung (1975) but was not formally described until Smith (1995). The type locality for *Aphonopelma gabeli* (“east of Tucson”) is vague and is probably located closer to Willcox or Safford, Arizona.

### Aphonopelma hentzi

Taxon classificationAnimaliaAraneaeTheraphosidae

(Girard, 1852)

**Figure 50.**
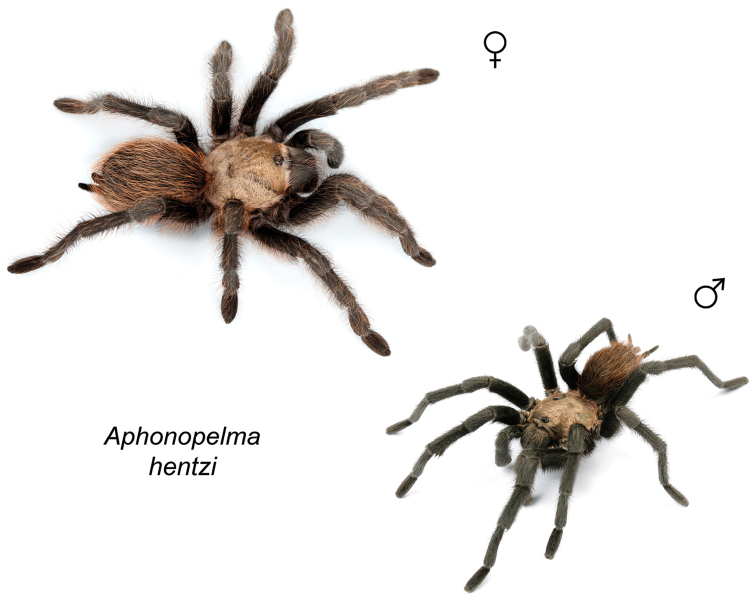
*Aphonopelma hentzi* (Girard, 1854) specimens, live photographs. Female (L) - APH_0576; Male (R) - APH_3216.

**Figure 51.**
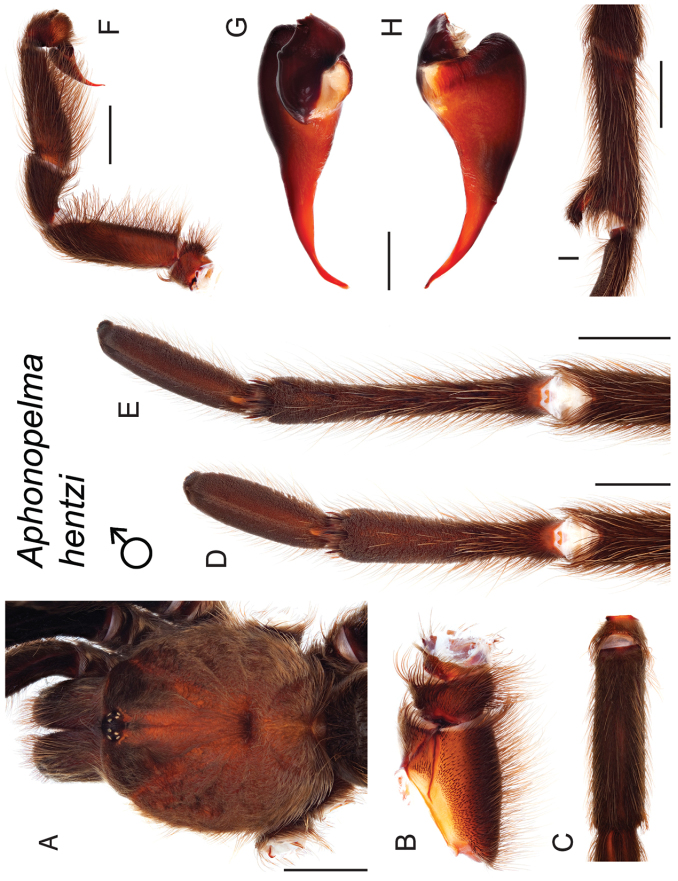
*Aphonopelma hentzi* (Girard, 1854). **A–I** male specimen, APH_0921 **A** dorsal view of carapace, scale bar = 6mm **B** prolateral view of coxa I **C** dorsal view of femur III **D** ventral view of metatarsus III, scale bar = 4mm **E** ventral view of metatarsus IV, scale bar = 5mm **F** prolateral view of L pedipalp and palpal tibia, scale bar = 4.5mm **G** dorsal view of palpal bulb **H** retrolateral view of palpal bulb, scale bar = 1mm **I** prolateral view of tibia I (mating clasper), scale bar = 5mm.

**Figure 52.**
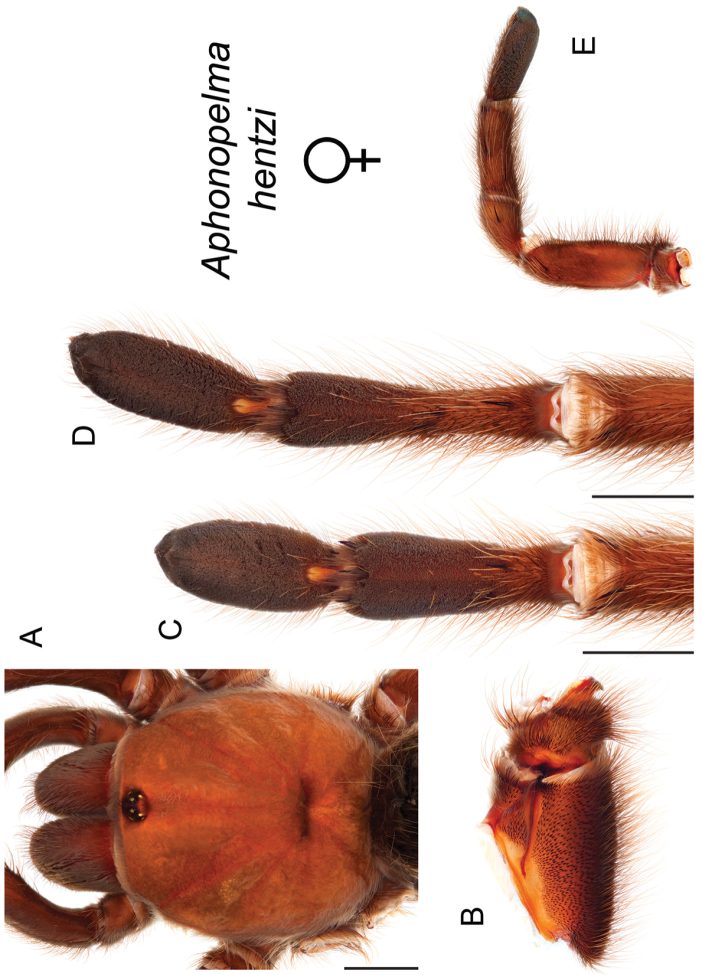
*Aphonopelma hentzi* (Girard, 1854). **A–E** female specimen, APH_0812 **A** dorsal view of carapace, scale bar = 6mm **B** prolateral view of coxa I **C** ventral view of metatarsus III, scale bar = 4.5mm **D** ventral view of metatarsus IV, scale bar = 4.5mm **E** prolateral view of L pedipalp and palpal tibia.

**Figure 53.**
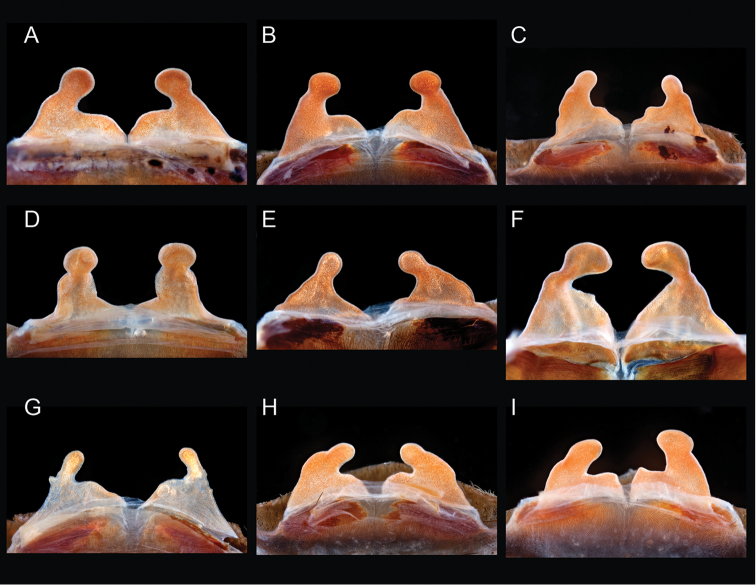
*Aphonopelma hentzi* (Girard, 1854). **A–I** cleared spermathecae **A** APH_0052 **B** APH_0571 **C** APH_0743 **D** APH_0812 **E** APH_0813 **F** APH_0833 **G** APH_0838 **H** APH_0862 **I** APH_0867.

**Figure 54.**
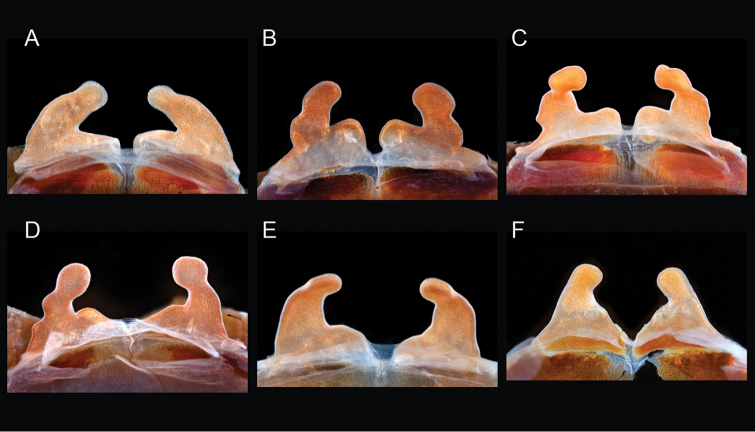
*Aphonopelma hentzi* (Girard, 1854). **A–F** cleared spermathecae **A** APH_0868 **B** APH_0927 **C** APH_0934 **D** APH_1063 **E** APH_1353 **F** *harlingenum* holotype.

**Figure 55.**
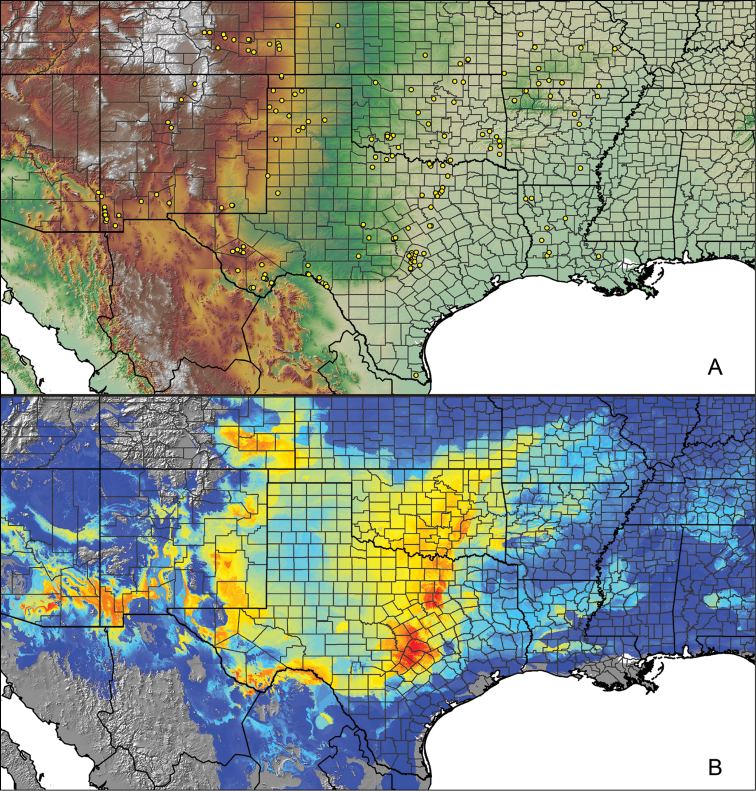
*Aphonopelma hentzi* (Girard, 1854). **A** distribution of known specimens **B** predicted distribution; warmer colors (red, orange, yellow) represent areas of high probability of occurrence, cooler colors (blue shades) represent areas of low probability of occurrence. Of note, the lone south Texas outlier specimen in A is the *harlingenum* holotype.

Figures 50 , 51 , 52 , 53 , 54 , 55 ; Suppl. material 4

Mygale hentzii Girard, 1852: 251; male holotype from southwestern Oklahoma, 34.309094 -98.396494^7^, elev. 968ft., coll. unknown, 1849-1852.
Eurypelma hentzi Simon, 1891: 322.
Rhechostica hentzi Raven, 1985: 149.
Aphonopelma hentzi Smith, 1995: 107; neotype male and female exemplar from Garfield Co., Oklahoma, 36.436139 -97.872160^7^, elev. 1264ft., summer 1975, coll. R.L. Lardie; deposited in Oklahoma State University collection. [not examined]
Aphonopelma clarki Smith, 1995: 87; female holotype from Dallas, Dallas Co., Texas, 32.780141 -96.800451^7^, elev. 421ft., 1968, coll. H.J. Berman; deposited in BMNH. [examined] **syn. n.**
Dugesiella coloradana Chamberlin, 1940: 35; female holotype from Sugar City, Crowley Co., Colorado, 38.231949 -103.663001^6^, elev. 4307ft., no collecting date, coll. unknown; deposited in AMNH. [examined]
Rhechostica coloradanum Raven, 1985: 149.
Aphonopelma coloradanum Smith, 1995: 90. **syn. n.**
Dugesiella echina Chamberlin, 1940: 36; male holotype from Arkansas Valley, Colorado, 38.055115 -103.621743^7^, elev. 4182ft., 15.xi.1938, coll. unknown; deposited in AMNH. [examined]
Rhechostica echinum Raven, 1985: 149.
Aphonopelma echinum Smith, 1995: 96. **syn. n.**
Aphonopelma gurleyi Smith, 1995: 104; male holotype from Interstate 35, near Moss Lake, Sherman, Grayson Co., Texas, 33.781417 -97.221633^5^, elev. 796ft., no collection date, coll. Russ Gurley; deposited in BMNH. [examined] **syn. n.**
Dugesiella harlingena Chamberlin, 1940: 37; female holotype from Harlingen, Cameron Co., Texas, 26.190631 -97.696103^6^, elev. 41ft., 1939, coll. Bryce Brown; deposited in AMNH. [examined]
Rhechostica harlingenum Raven, 1985: 149.
Aphonopelma harlingenum Smith, 1995: 106. **syn. n.**
Aphonopelma odelli Smith, 1995: 126; female holotype from Beavers Bend State Resort Park McCurtain Co., Oklahoma, 34.13104 -94.69004^5^, elev. 670ft., 13.vi.1979, coll. D.C. Arnold; deposited in Oklahoma State University collection. [not examined] **syn. n.**
Dugesiella wacona Chamberlin, 1940: 38; male holotype from Waco, McClennan Co., Texas, 31.549333 -97.146670^6^, elev. 498ft., 5.vii.1931, coll. unknown; deposited in AMNH. [examined]
Rhechostica waconum Raven, 1985: 149.
Aphonopelma waconum Smith, 1995: 156. **syn. n.**
Dugesiella wichitana Chamberlin, 1940: 35; male holotype from Wichita Mtns. National Wildlife Refuge, Comanche Co., Oklahoma, 34.772106 -98.601308^6^, elev. 1495ft., 5.vii.1928, coll. N.M. Newport; deposited in AMNH. [examined]
Rhechostica wichitanum Raven, 1985: 149.
Aphonopelma wichitanum Smith, 1995: 157. **syn. n.**

#### Diagnosis.

*Aphonopelma hentzi* (Fig. 50) is a member of the *Hentzi* species group and can be identified by a combination of morphological, molecular, and geographic characteristics. Nuclear DNA identifies *Aphonopelma hentzi* as a phylogenetically distinct monophyletic lineage (Fig. 8), supported as the sister lineage to *Aphonopelma anax*, closely related to *Aphonopelma armada*, and exclusive of *Aphonopelma moellendorfi* sp. n. (a new species previously confused with *Aphonopelma hentzi*). *Aphonopelma hentzi* can be distinguished from *Aphonopelma anax* by the shapes of its spermathecae in females and palpal bulbs in males; from *Aphonopelma armada* by the pattern of setae on coxa I and the flared metatarsal scopulae of females and juvenile males; from *Aphonopelma gabeli* by its large chelicerae and general phenotypic appearance; from *moderatum* by their unique phenotypic color and banding; from *parvum* sp. n. due to the extreme small size; from *peloncillo* by its phenotypic appearance; and from *vorhiesi* due to its black appearance. Significant measurements that distinguish male *Aphonopelma hentzi* from its closely related phylogenetic and syntopic species are PTl, M1, and the extent of scopulation on metatarsus III. Male *Aphonopelma hentzi* can be distinguished by possessing a larger PTl/M1 (≥0.74; 0.74–0.88) than *Aphonopelma gabeli* (≤0.68; 0.61–0.68), *Aphonopelma moderatum* (≤0.69; 0.61–0.69), and *Aphonopelma moellendorfi* (≤0.69; 0.60–0.69); a larger L3 scopulation extent (69%–86%) than *Aphonopelma armada* (48%-63%), *Aphonopelma peloncillo* sp. n. (52%-68%), and *Aphonopelma vorhiesi* (44%–62%); and by possessing a smaller M1/F4 (≤0.77; 0.68–0.77) than *Aphonopelma moderatum* (≥0.80; 0.80–0.87) and *Aphonopelma moellendorfi* sp. n. (≥0.81; 0.81–0.88). The significant measurement that distinguishes female *Aphonopelma hentzi* from syntopic species is the extent of scopulation on metatarsus IV. Female *Aphonopelma hentzi* can be distinguished by possessing a larger L4 scopulation extent (42%–72%) than *Aphonopelma peloncillo* (32%–38%) and *Aphonopelma vorhiesi* (26%–37%). There are no significant measurements that separate female *Aphonopelma hentzi* from *Aphonopelma anax*, *Aphonopelma armada*, *Aphonopelma gabeli*, or *Aphonopelma moderatum*. Females of *Aphonopelma moellendorfi* are unknown and cannot be compared.

#### Description.

Male and female described by Girard (1852); presumed lost. Neotype designated and redescribed by Smith (1995); new female specimen designated and redescribed by Smith (1995).

#### Redescription of male exemplar

(APH_0921; Fig. 51). *Specimen preparation and condition*: Specimen collected live crossing road, preserved in 80% ethanol; deposited in AUMNH; original coloration faded due to preservation. Left legs I, III, IV, and left pedipalp removed for measurements and photographs; stored in vial with specimen. Right leg III removed for DNA and stored at -80°C in the AUMNH (Auburn, AL). *General coloration*: Generally black or faded to brown. *Cephalothorax*: Carapace 14.89 mm long, 13.47 mm wide; Hirsute; densely clothed with brown/golden iridescent pubescence appressed to surface; fringe covered in long setae not closely appressed to surface; foveal groove deep and straight; pars cephalica region rises gradually from foveal groove, gently arching anteriorly toward ocular area; AER slightly procurved, PER recurved; normal sized chelicerae; clypeus extends forward on a slight curve; LBl 1.79, LBw 1.93; sternum very hirsute, clothed with long black/brown, densely packed setae. *Abdomen*: Densely clothed in short black/brown pubescence with numerous longer red/orange setae interspersed; possessing a dense dorsal patch of black Type I urticating bristles (Cooke et al. 1972). *Legs*: Very hirsute, particularly ventrally; densely clothed in a mix of medium and long black/brown pubescence. Metatarsus I slightly curved. F1 14.11; F1w 3.69; P1 5,94; T1 12.54; M1 10.48; A1 7.60; F3 12.06; F3w 3.71; P3 5.63; T3 9.32; M3 10.85; A3 7.56; F4 14.71; F4w 4.02; P4 5.86; T4 12.17; M4 14.99; A4 8.36; femur III is normal - not noticeably swollen or wider than other legs. All tarsi fully scopulate. Extent of metatarsal scopulation: leg III (SC3) = 80.9%; leg IV (SC4) = 54.3%. Two ventral spinose setae on metatarsus III; nine ventral spinose setae on metatarsus IV. *Coxa I*: Prolateral surface a mix of fine, hair-like and thick tapered setae. *Pedipalps*: Very hirsute; densely clothed in the same setal color as the other legs, with numerous longer ventral setae; one spinose seta on the apical, prolateral femur and four spinose setae on the prolateral tibia; PTl 8.525, PTw 2.77. When extended, embolus tapers but gently curves to the retrolateral side near apex; embolus very slender, no keels.

**Variation (14).** Cl 13.58–18.04 (16.008±0.38), Cw 12.45–17.28 (14.924±0.41), LBl 1.78–2.46 (2.09±0.07), LBw 1.92–2.79 (2.371±0.09), F1 13.1–17.31 (15.509±0.36), F1w 3.19–4.50 (3.991±0.1), P1 5.36–7.63 (6.494±0.18), T1 10.71–14.18 (12.986±0.28), M1 9.15–13.03 (11.118±0.29), A1 6.03–9.13 (7.836±0.26), L1 length 44.35–60.93 (53.943±1.31), F3 10.56–16.83 (13.026±0.42), F3w 3.23–4.71 (3.999±0.11), P3 4.16–7.57 (5.675±0.23), T3 7.89–12.61 (10.126±0.33), M3 9.69–13.93 (11.859±0.33), A3 5.92–8.88 (7.786±0.21), L3 length 38.72–59.23 (48.471±1.42), F4 12.88–17.50 (15.175±0.37), F4w 3.11–4.69 (3.924±0.12), P4 4.36–7.13 (6.046±0.2), T4 10.75–15.16 (12.809±0.32), M4 12.91–17.57 (15.406±0.33), A4 6.64–9.95 (8.803±0.26), L4 length 47.54–66.69 (58.239±1.42), PTl 7.762–10.81 (9.067±0.23), PTw 2.722–3.61 (3.003±0.07), SC3 ratio 0.691–0.866 (0.769±0.01), SC4 ratio 0.324–0.968 (0.579±0.05), Coxa 1 setae = stout/thick tapered, F3 condition = normal/slightly swollen.

#### Redescription of female exemplar

(APH_0812; Figs 52–54). *Specimen preparation and condition*: Specimen collected live from burrow, preserved in 80% ethanol; deposited in AUMNH; original coloration faded due to preservation. Left legs I, III, IV, and pedipalp removed for photographs and measurements; stored in vial with specimen. Right leg III removed for DNA and stored at -80°C in the AUMNH (Auburn, AL). Genital plate with spermathecae removed and cleared, stored in vial with specimen. *General coloration*: Faded brown/black, with a very hirsute appearance. *Cephalothorax*: Carapace 20.89 mm long, 19.82 mm wide; densely clothed with light brown pubescence closely appressed to surface; fringe densely covered in longer setae; foveal groove medium deep and straight; pars cephalica region gently rises from thoracic furrow, arching anteriorly toward ocular area; AER slightly procurved, PER recurved; clypeus extends forward on a slight curve; LBl 2.84, LBw 3.42; sternum very hirsute, clothed with black/brown, long setae. *Abdomen*: Densely clothed dorsally in short black setae with numerous longer, lighter setae interspersed (generally red or orange *in situ*); dense dorsal patch of black Type I urticating bristles (Cooke et al. 1972); ventral side with shorter dark brown/black setae. *Spermathecae*: Uniquely shaped; paired and separate, taper rapidly to a medium width with a secondary bulge and strongly capitate bulbs, with wide bases that are not fused. *Legs*: Very hirsute, particularly ventrally; densely clothed in long brown pubescence. F1 14.90; F1w 4.85; P1 6.91; T1 11.32; M1 7.95; A1 7.60; F3 12.65; F3w 4.33; P3 6.62; T3 8.82; M3 9.39; A3 7.48; F4 14.91; F4w 4.27; P4 6.34; T4 10.97; M4 13.48; A4 8.65. All tarsi fully scopulate. Extent of metatarsal scopulation: leg III (SC3) = 74.0%; leg IV (SC4) = 54.7%. Two ventral spinose setae on metatarsus III; four ventral spinose setae on metatarsus IV. *Coxa I*: Prolateral surface a mix of fine, hair-like and thick tapered setae. *Pedipalps*: Densely clothed in the same setal color as the other legs; one spinose seta on the apical, prolateral femur, one spinose seta on the prolateral patella, and three spinose setae on the prolateral tibia.

**Variation (15).** Cl 12.65–22.36 (17.892±0.68), Cw 11.57–19.87 (16.328±0.61), LBl 1.91–2.86 (2.399±0.08), LBw 2.13–3.42 (2.773±0.1), F1 10.41–17.13 (13.72±0.48), F1w 3.16–5.56 (4.413±0.17), P1 4.55–8.09 (6.4±0.25), T1 7.79–13.28 (10.669±0.32), M1 6.06–10.74 (7.945±0.3), A1 5.17–8.86 (6.671±0.23), L1 length 34.72–57.70 (45.404±1.51), F3 8.32–14.09 (11.281±0.43), F3w 2.79–4.99 (3.804±0.16), P3 3.94–8.06 (5.64±0.31), T3 6.11–10.56 (8.164±0.32), M3 6.46–11.65 (8.915±0.38), A3 5.46–9.03 (6.866±0.26), L3 length 30.84–53.39 (40.818±1.7), F4 10.43–17.15 (13.795±0.44), F4w 3.04–5.16 (4.072±0.15), P4 4.58–7.81 (5.906±0.23), T4 8.66–13.41 (10.851±0.31), M4 8.95–15.84 (11.924±0.47), A4 6.04–9.79 (7.672±0.27), L4 length 39.16–64.0 (50.132±1.68), SC3 ratio 0.671–0.953 (0.805±0.02), SC4 ratio 0.424–0.717 (0.547±0.02), Coxa 1 setae = stout/thick tapered. Spermathecae variation can be seen in Figures 53–54.

#### Material examined.

**United States: Arkansas: Carroll**: near Eureka Springs, 36.404273 -93.735646 ^5^, 1201ft., [APH_2600, unknown, 1♂, H.A. Clay, AMNH]; **Cleburne**: 6 miles S/SW of Drasco, 35.569318 -91.998288 ^5^, 843ft., [APH_2602, 9/1979, 1♀, D. Pearson, AMNH]; **Drew**: Monticello, 33.628997 -91.790964 ^5^, 292ft., [APH_2174, 1936, 1♀, unknown, AMNH]; **Lawrence**: Imboden, 36.202568 -91.174573 ^5^, 308ft., [APH_2196, 1931, 5♂, R.V. Chamberlin, AMNH]; [APH_2203, 1935, 1♀, 1♂, unknown, AMNH]; **Marion**: between Pyatt and Eros, near Jct Hwy 125 and MC 4054, 36.21402 -92.86224 ^4^, 912ft., [APH_0024, 9/2002, 1♂, Lewonna Nelson, AUMNH]; **Washington**: Fayetteville, 36.044074 -94.169349 ^5^, 1211ft., [APH_2172-2173, unknown, 2♂, 1♀, W.J. Baerg, AMNH]; [APH_2467, 1921, 2♀, 1♂, W.J. Baerg, AMNH]; **Arizona: Greenlee**: 0.4 miles N Hwy-75 on Goat Camp Rd, 32.755403 -109.110492 ^1^, 3726ft., [APH_1352-1353, 5/8/2011, 2♀, Brent E. Hendrixson, Brendon Barnes, Nate Davis, Jake Storms, AUMNH]; **Colorado: Bent**: John Martin Reservoir State Park, 38.07612 -102.957421 ^1^, 3839ft., [APH_0570, 10/6/2009, 1 juv, Brent E. Hendrixson, Courtney Dugas, Sloan Click, AUMNH]; **Crowley**: 1.5 miles N Hwy-96 on CR-20, S of Lake Henry (near Sugar City), 38.246723 -103.711337 ^1^, 4371ft., [APH_0565, 9/6/2009, 1 juv, Brent E. Hendrixson, Courtney Dugas, Sloan Click, AUMNH]; **Fremont**: Canon City, Hwy 64, 5 miles N of Junction with Hwy 50, 38.496596 -105.106624 ^5^, 5800ft., [AUMS_3334, 17/8/1987, 1♂, unknown, AUMNH]; Canon City, N toward Cripple Creek, 5 miles N off Hwy 50, 38.497302 -105.291414 ^5^, 6248ft., [AUMS_3338, 17/8/1986, 1♀, T.R. Prentice, AUMNH]; **Otero**: 1.5 miles SW Hwy-109 on CR-802, in field and bluffs N of road, 37.794004 -103.521272 ^1^, 4425ft., [APH_0560-0564, 9/6/2009, 5♀, Brent E. Hendrixson, Courtney Dugas, Sloan Click, AUMNH]; Commanche Grasslands, La Junta, 37.859983 -103.719267 ^6^, 4440ft., [APH_0917, 10/2009, 1♂, Ken Suzuki, AUMNH]; **Prowers**: Prowers Co. Rd., S. of Lamar, on Deahl Ranch, 37.892338 -102.60625 ^6^, 3914ft., [AUMS_2469, 4/7/1986, 1♂, T.R. Prentice, AUMNH]; S of Lamar, Deahl Ranch, 38.013237 -102.613274 ^6^, 3804ft., [AUMS_2455, 4/7/1986, 1♀, T.R. Prentice, AUMNH]; **Pueblo**: 1.5 miles E Boone on Hwy-96, 38.243146 -104.233761 ^2^, 4448ft., [APH_0749, 16/9/2009, 1♂, Jerry Smith, Mario Juvera, AUMNH]; I-25, approx. 25 miles S Pueblo, 37.93879 -104.81027 ^5^, 5908ft., [APH_0022, 7/9/2002, 1♂, Kristin Young, AUMNH]; Piñon Truck Stop, I-25 N of Pueblo, 38.433689 -104.610331 ^4^, 5030ft., [AUMS_2329, 28/6/1986, 1♂, T.R. Prentice, AUMNH]; Pueblo, 0.9 miles N of Hwy-47 on Baculite Mesa Rd, 38.302884 -104.553482 ^1^, 4814ft., [APH_0566-0569, 9/6/2009, 4 juv, Brent E. Hendrixson, Courtney Dugas, Sloan Click, AUMNH]; **Kansas: Elk**: 1.5 miles W of Longton, 37.377606 -96.108334 ^4^, 935ft., [AUMS_2321, 28/4/1994, 1♂, B. Cutler, AUMNH]; **Gove**: along Smokey Hill River just W of Hwy 23, 38.74457 -100.50428 ^4^, 2453ft., [APH_0027, 10/2002, 1♂, Alvis Wade, AUMNH]; **Wilson**: Fredonia, 37.532925 -95.826937 ^5^, 922ft., [APH_2603, 16/8/1964, 1♂, A.R. Moldenke, AMNH]; Fredonia, 2.5 miles SW Hwy 400 and Hwy 47, 37.50556 -95.83278 ^5^, 858ft., [APH_0003, 6/7/2003, 1♀, Jordan B. Johnson, AUMNH]; **Louisiana: Allen (Parish)**: near town of Harmony, off Hwy-1147, 30.59583 -92.91361 ^2^, 57ft., [APH_1171, 24/4/2010, 1 juv, Roland H. Delaune, AUMNH]; Reeves, 2250 Martin Tram Rd, 30.48063 -92.9884 ^2^, 25ft., [APH_0060, 29/10/2006, 1♂, Gary Bellon, AUMNH]; **Bossier (Parish)**: Haughton, 400 ft. N I-20, 1/2 mile W Hwy 614, 32.53917 -93.54944 ^4^, 230ft., [APH_0028, 20/10/2002, 1♂, Jimmy Landen, AUMNH]; **Caddo (Parish)**: Shreveport, 32.525152 -93.750179 ^6^, 159ft., [AUMS_2449, 16/5/1996, 1♂, R. Moore, AUMNH]; **East Baton Rouge (Parish)**: Baton Rouge, 30.457702 -91.14032 ^6^, 49ft., [APH_2610, 1958, 1♂, Leon Rody, AMNH]; **La Salle (Parish)**: Tullos, 31.819261 -92.329862 ^5^, 121ft., [APH_2612, 1/10/1950, 1♂, W.J. Gerstch, AMNH]; **Natchitoches (Parish)**: Kisatchie National Forest, Kisatchie District, along Longleaf Vista Trail, 31.47644 -92.99622 ^5^, 288ft., [APH_0403, 4/10/2008, 1♂, Brent E. Hendrixson, AUMNH]; **Vernon (Parish)**: Near Fort Polk, 31.029236 -93.176675 ^6^, 289ft., [APH_2202, 1957, 1♀, F.E. Potter, AMNH]; **Missouri: Greene**: Springfield, 37.208957 -93.292299 ^5^, 1280ft., [APH_2183, 1931, 1♂, Truman Smith, AMNH]; [APH_2190, 10/1931, 2♂, Truman Smith, AMNH]; **Henry**: Hartwell, 38.435258 -93.934662 ^5^, 761ft., [APH_2611, 1937, 1♂, J.K. Connolly, AMNH]; **Hickory**: 4 miles northwest of Wheatland, 37.969377 -93.427109 ^5^, 991ft., [APH_2609, 2/10/1967, 1♂, W. Ivie, AMNH]; **Newton**: Joplin , 37.031191 -94.52842 ^5^, 909ft., [APH_2191, 11/10/1942, 4♂, A.B. Gurney, AMNH]; **Ozark**: Caney Mountain Conservation Area, along Long Bald trail, 36.696831 -92.45143 ^1^, 1193ft., [APH_0591, 25/6/2009, 1♀, Brent E. Hendrixson, AUMNH]; **Phelps**: Rolla, 37.948543 -91.771535 ^5^, 1093ft., [APH_2175, 1/10/1954, 4♂, L. Bade and Walker, AMNH]; [APH_2177, 10/1960, 1♂, Lee Malone, AMNH]; **Ripley**: between Calm and Gatewood on Hwy 142, 36.560976 -91.117925 ^1^, 786ft., [APH_0874, 6/2008, 1 juv, Matt Ingrasci, AUMNH]; **St. Francois**: Bonne Terre, near city lake, 37.912189 -90.55567 ^4^, 866ft., [APH_0199, 30/9/2007, 1♂, Andrew Rieger, AUMNH]; **Taney**: Bradleyville, edge of floodplains of Breaver Creek, 36.7754 -92.920951 ^5^, 814ft., [APH_2608, 25/9/1955, 1♂, N. and J. Crenshaw, AMNH]; near Branson, Ruth and Paul Henning Conservation Area, 36.6627 -93.293056 ^1^, 1165ft., [APH_0586-0590, 24/6/2009, 5 juv, Brent E. Hendrixson, AUMNH]; **New Mexico: Bernalillo**: Albuquerque, base of Sandia Mtns at Copper Trail, 35.079247 -106.484022 ^1^, 5948ft., [APH_1437, 12/11/2011, 1♂, Jessica Ashley, AUMNH]; **Dona Ana**: Aguirre Springs Campground, 32.370504 -106.561475 ^1^, 5662ft., [APH_0539-0540, 5/6/2009, 2♀, Brent E. Hendrixson, Courtney Dugas, Sloan Click, AUMNH]; CR-72, just off I-25, 32.67635 -107.012973 ^1^, 4348ft., [APH_0654, 13/7/2009, 1 juv, Brent E. Hendrixson, Nate Davis, AUMNH]; **Eddy**: 0.3 miles W US-62/180 on CR 408 (Dark Canyon Rd), 32.28694 -104.28646 ^1^, 3320ft., [APH_0363, 21/6/2008, 1 juv, Shasta Michaels, AUMNH]; 1.7 miles W US-62/180 on CR-408, 32.293874 -104.309514 ^1^, 3392ft., [APH_0544, 6/6/2009, 1 juv, Brent E. Hendrixson, Courtney Dugas, Sloan Click, AUMNH]; Lincoln National Forest, just off Queens Hwy, 32.20002 -104.67976 ^1^, 5583ft., [APH_0522, 31/8/2008, 1♂, Rick C. West, AUMNH]; **Hidalgo**: 10 miles S I-10 on Hwy-338, 32.13267 -108.87908 ^2^, 4210ft., [APH_0795, 22/10/2009, 1♂, Clyde Mahan, AUMNH]; 2.64 miles W Hwy-338 on Hwy-9, 31.94331765 -108.8509352 ^2^, 4403ft., [APH_0728, 18/8/2009, 1♂, Alice Abela, AUMNH]; 4 miles west of Animas, 31.942746 -108.853622 ^5^, 4400ft., [APH_2628, 26/8/1960, 1♂, unknown, AMNH]; along Hwy-338, 32.226309 -108.879093 ^1^, 4078ft., [APH_1510-1511, 8/9/2012, 2♂, Brent E. Hendrixson, AUMNH]; [APH_1513-1515, 8/9/2012, 3♂, Brent E. Hendrixson, AUMNH]; along Hwy-9, W of Animas, 31.94337666 -108.8874883 ^2^, 4407ft., [APH_0726, 18/8/2009, 1♂, Alice Abela, AUMNH]; [APH_0729, 18/8/2009, 1♂, Alice Abela, AUMNH]; along US-70, SE of Hwy-92, 32.612037 -108.984613 ^1^, 4151ft., [APH_0645, 12/7/2009, 1 juv, Brent E. Hendrixson, Nate Davis, AUMNH]; E side of Animas Mtns, 31.560017 -108.499913 ^6^, 4430ft., [APH_0862, 2006, 1♀, Dave Moellendorf, AUMNH]; NM-9 at Little Hatchet Mtns Rd, W of Hachita, 31.926633 -108.363083 ^4^, 4480ft., [APH_0408-0410, 11/10/2008, 3♂, Kari McWest, Keisha Hendricks, AUMNH]; **Luna**: 15.5 miles NE Hwy-180 on Hwy-26, 32.430777 -107.566477 ^1^, 4584ft., [APH_1572, 29/10/2012, 1♂, Brent E. Hendrixson, AUMNH]; **Rio Arriba**: along CR 55, 36.084375 -106.12193 ^2^, 5715ft., [APH_1248, 1/11/2010, 1♂, Chis Tough, AUMNH]; **Sandoval**: Rt 44 (Rt 448), 3.7 miles west of Placitas, 35.269619 -106.605786 ^4^, 5046ft., [APH_2198, 16/11/1963, 2♂, William A. Shear, AMNH]; **Taos**: 70 miles N of Santa Fe, 36.650692 -105.636765 ^7^, 7832ft., [AUMS_2476, unknown, 1♂, unknown, AUMNH]; **Oklahoma: Bryan**: Calera Golf Center, 2.5 miles S Calera on Hwy-75, 33.9094 -96.462 ^4^, 755ft., [APH_0156, 22/7/2007, 1♂, Julianne Smith, AUMNH]; **Cimarron**: 6.6 miles S Colorado State line on US-287, 36.900429 -102.520409 ^1^, 3924ft., [APH_0555-0559, 8/6/2009, 2♀, 3 juv, Brent E. Hendrixson, Courtney Dugas, Sloan Click, AUMNH]; Oklahoma panhandle, Cimarron River, just N off Hwy 287-385, N of Boise City, 36.945676 -102.521756 ^6^, 3933ft., [AUMS_2472, 1/6/1986, 1♂, T.R. Prentice, AUMNH]; **Cleveland**: Lake Thunderbird State Park, 35.21208 -97.24742 ^5^, 1085ft., [APH_0052, 25/6/2004, 1♀, Brent E. Hendrixson, AUMNH]; [APH_0150, 25/6/2004, 1♀, Brent E. Hendrixson, AUMNH]; **Comanche**: In the Wichita Mtns, 3 miles north of Medicine Park, 34.798239 -98.511099 ^5^, 1411ft., [APH_2204, 19/5/1977, 1♀, J.C. Colkendolpher, AMNH]; N side of Wichita Mtns, 34.841533 -98.727542 ^1^, 1771ft., [APH_0571-0574, 11/6/2009, 1♀, 3 juv, Brent E. Hendrixson, Courtney Dugas, Sloan Click, AUMNH]; Wichita National Forest , 37.683896 -97.363593 ^5^, 1302ft., [APH_2176, unknown, 2♂, unknown, AMNH]; Wichita Mtns near Mount Scott, 34.767625 -98.724761 ^6^, 1955ft., [APH_2601, 16/9/1971, 1♀, R. Forbes, AMNH]; Wichita Mtns Wildlife Refuge, 34.709358 -98.623307 ^6^, 1476ft., [APH_2455, 6/9/1975, 1♂, F. Fryce, AMNH]; Wichita Mtns NWR - Comanche Rd., 34.780183 -98.598183 ^1^, 1520ft., [APH_0873, 5/2008, 1 juv, Christian Cox, AUMNH]; Wichita Mtns NWR - Taylor Ranch Rd., 34.841783 -98.728283 ^1^, 1712ft., [APH_0939, 5/2008, 1♀, Christian Cox, AUMNH]; **Cotton**: in cemetery NW of Walters, approx. 0.4 miles N Hwy-5 on CR-N2610, 34.367655 -98.329618 ^1^, 1029ft., [APH_0575, 11/6/2009, 1 juv, Brent E. Hendrixson, Courtney Dugas, Sloan Click, AUMNH]; **Creek**: SW of Tulsa, 35.99622 -96.33341 ^1^, 787ft., [APH_0918, 25/4/2008, 1♂, Christian Cox, John Morse, AUMNH]; SW of Tulsa, NE OK off route 33, 35.99622 -96.33341 ^1^, 787ft., [APH_0834, 25/4/2008, 1♀, Christian Cox, John Morse, AUMNH]; **Jackson**: 498 Honeysuckle ave, Altus, Altus Air Force Base, 34.664112 -99.296041 ^1^, 1372ft., [APH_3124, 15/6/2013, 1♂, David Welch, AUMNH]; **Latimer**: Along US-270, E of Red Oak, 34.950086 -95.054512 ^1^, 571ft., [APH_0585, 24/6/2009, 1 juv, Brent E. Hendrixson, AUMNH]; unknown, 34.835035 -95.31025 ^7^, 830ft., [APH_2182, 1931, 4♂, unknown, AMNH]; [APH_2193, 6/1931, 1♀, 2♂, 1 juv, unknown, AMNH]; **Le Flore**: Ouachita Mtns, S of Smithville on US 259, 34.418983 -94.676 ^1^, 736ft., [APH_0921, 10/2008, 1♂, Chris A. Hamilton, AUMNH]; Ouachita Mtns, S on Hwy 259 from Winding Stair Mountain, 34.600767 -94.6987 ^1^, 1701ft., [APH_0920, 10/2008, 1♂, Mike Logan, AUMNH]; Ouachita Mtns, Winding Stair Mountain, 34.7489 -94.8037 ^1^, 2185ft., [APH_0919, 10/2008, 1♂, Brian Fontenot, AUMNH]; **Logan**: Stillwater , 36.115607 -97.058368 ^5^, 876ft., [APH_2181, 15/9/1969, 1♀, N.V. Horner, AMNH]; **McCurtain**: 1 mile east of Signal Mountain, Fort Hill, 34.333306 -95.024261 ^4^, 1168ft., [APH_2201, 3/10/1952, 1♀, 2♂, T. Cohn , AMNH]; **Oklahoma**: Oklahoma City/Edmond at intersection of 150th St and Macarrthur, 35.623642 -97.620781 ^2^, 1100ft., [APH_0402, 21/9/2008, 1♂, Natalie Ellis, AUMNH]; **Osage**: between Pahuska and Bartlesville (10 miles west of Bartlesville), 36.740071 -96.299699 ^5^, 827ft., [APH_2606, 6/9/1940, 1♀, 1♂, A. Clay, AMNH]; **Pontotoc**: Ada, 34.773126 -96.678772 ^5^, 1007ft., [APH_2607, 10/1981, 1♂, unknown, AMNH]; **Washington**: 2 miles W Bartlesville, Circle Mountain, forested area, 36.71598 -95.99562 ^4^, 677ft., [APH_0026, 29/9/2002, 1♂, Jerry Tolbert, AUMNH]; **Woodward**: Alabaster Cavern- 9 miles south of Freedom, 36.698679 -99.147057 ^5^, 1742ft., [APH_2605, 12/10/1952, 1♂, unknown, AMNH]; **Texas: Bastrop**: Wolf Lane and Pearce Rd, 30.140783 -97.5673 ^5^, 555ft., [APH_0960-0964, 22/5/2006, 5♂, Dave Moellendorf, AUMNH]; **Baylor**: 7 miles north of Maybelle, 33.654334 -99.263208 ^5^, 1325ft., [APH_2657, 8/11/1964, 2♂, Karl W. Haller, AMNH]; **Brewster**: 3.5 miles S US-90 on US-395, 30.15626 -103.23608 ^1^, 4070ft., [APH_1288, 14/5/2011, 1♂, Brent E. Hendrixson, Kate Hall, Austin Deskewies, Alexis Guice, AUMNH]; Big Bend, 29.330733 -103.536367 ^6^, 2592ft., [APH_0933, 5/2008, 1♂, Corey Roelke, AUMNH]; Black Gap WMA, 29.467633 -102.837333 ^6^, 1833ft., [APH_0868, 2006, 1♀, Dave Moellendorf, AUMNH]; [APH_0973, 2006, 1♂, Dave Moellendorf, AUMNH]; [APH_0975, 2006, 1♂, Dave Moellendorf, AUMNH]; Big Bend National Park, 0.55 miles S park boundary on US-385, 29.67449 -103.17149 ^1^, 2910ft., [APH_1289, 15/5/2011, 1♂, Brent E. Hendrixson, Kate Hall, Austin Deskewies, Alexis Guice, AUMNH]; Black Gap WMA, 29.5058 -102.884817 ^1^, 2205ft., [APH_0932, 5/2008, 1♂, Corey Roelke, AUMNH]; [APH_0972, 6/2006, 1♂, Dave Moellendorf, AUMNH]; [APH_0974, 2006, 1♂, Dave Moellendorf, AUMNH]; Black Gap WMA, on FM2627, 29.4867 -102.862733 ^1^, 2009ft., [APH_0931, 5/2008, 1♂, Corey Roelke, AUMNH]; **Carson**: Pantex Plant, 35.302672 -101.561938 ^5^, 3534ft., [AUMS_2328, 7/10/1997, 1♀, Felix Chavez, AUMNH]; **Coleman**: O.H. Ivie reservoir, 31.60165 -99.5915 ^1^, 1684ft., [APH_0832, 9/2008, 1♀, Chris A. Hamilton, AUMNH]; **Collin**: Anna, 2 miles E of junction of FM455 and Hwy 75, 41 miles N of Dallas, 33.344388 -96.552215 ^5^, 659ft., [AUMS_2327, 7/1988, 1♂, SCJ, AUMNH]; [AUMS_2330, 7/1988, 1♂, SCJ, AUMNH]; **Cooke**: Moss Lake, Sherman, 33.781417 -97.221633 ^6^, 777ft., [APH_3035, unknown, 1♂, Russ Gurley, BMNH]; **Coryell**: Hwy 84, 14 miles W of Gatesville, 31.470143 -97.977296 ^5^, 1041ft., [AUMS_2616, 27/6/1999, 1♂, M. Buffington, AUMNH]; **Dallam**: Dalhart, 36.059477 -102.51325 ^5^, 3986ft., [AUMS_3291, unknown, 1♂, unknown, AUMNH]; just NE jct Thompson Grove Ln and Tex-Top Rd, 36.41505 -102.82264 ^1^, 4369ft., [APH_0744, 25/6/2009, 1 juv, Zach Valois, AUMNH]; **Dallas**: 1455 N. Joe Wilson Road, Cedar Hill, 32.62016 -96.925792 ^1^, 731ft., [APH_3125, 18/6/2013, 1♂, Nikki Miller, AUMNH]; Dallas, 32.802955 -96.769923 ^6^, 515ft., [APH_2200, 5/1938, 1♀, Ottys Sanders, AMNH]; Dallas, Dallas Zoo, 32.73665 -96.815917 ^1^, 484ft., [APH_0839, 5/2008, 1♂, Julie Post, AUMNH]; Dallas, Restland cemetery, 32.924625 -96.744856 ^1^, 593ft., [APH_0576, 12/6/2009, 1 juv, Brent E. Hendrixson, Courtney Dugas, Sloan Click, AUMNH]; [APH_0837-0838, 7/2008, 2♀, Chris A. Hamilton, AUMNH]; [APH_0915-0916, 7/2008, 2♂, Chris A. Hamilton, AUMNH]; [APH_0951, 7/2008, 1♂, Chris A. Hamilton, AUMNH]; Grand Prairie, 32.728783 -96.990383 ^1^, 477ft., [APH_0836, 3/2008, 1♀, Stephanie Rudy, AUMNH]; **Gaines**: Seminole, 0.4 miles NW 11th St on Hwy-214, 32.729362 -102.661669 ^1^, 3329ft., [APH_0546, 7/6/2009, 1 juv, Brent E. Hendrixson, Courtney Dugas, Sloan Click, AUMNH]; **Gray**: Pampa, approx. 12 miles S Pampa on US-70, 35.35843 -100.97436 ^5^, 3210ft., [APH_0023, 24/9/2002, 1♂, Kyle Harrison, AUMNH]; **Grayson**: 6 miles southwest of Pottsboro, 33.71582 -96.73669 ^5^, 666ft., [APH_2664, 1/11/1965, 1♂, Karl W. Haller, AMNH]; **Hartley**: Smith Ranch, N side of FM-767, 35.67812 -102.72925 ^1^, 3813ft., [APH_0745, 27/6/2009, 1 juv, Zach Valois, AUMNH]; **Hays**: Buda, Buda High School, 30.03265 -97.89275 ^4^, 848ft., [APH_0910, 7/2008, 1♂, Dave Moellendorf, AUMNH]; Kyle, 29.995 -97.893867 ^5^, 801ft., [APH_0835, 7/2006, 1♀, Dave Moellendorf, AUMNH]; [APH_0872, 2006, 1♀, Dave Moellendorf, AUMNH]; unknown rd N of FM-3296, 35.82407 -102.95766 ^1^, 4122ft., [APH_0743, 26/6/2009, 1 juv, Zach Valois, AUMNH]; **Hill**: 5 miles north of Itasca, 32.22622 -97.156988 ^5^, 659ft., [APH_2185, 16/7/1958, 1♂, P. Carne Jr., AMNH]; **Jeff Davis**: Davis Mtns, 30.8044 -103.8923 ^1^, 4087ft., [APH_0936, 31/9/2008, 1♂, Christian Cox, AUMNH]; Davis Mtns State Park, 30.592854 -103.940197 ^1^, 5045ft., [APH_0536, 4/6/2009, 1♀, Brent E. Hendrixson, Courtney Dugas, Sloan Click, AUMNH]; Davis Mtns, Hwy 118 between McDonald observatory and Davis Mtns State Park, 30.6638 -104.0317 ^1^, 6154ft., [APH_0934, 31/9/2008, 1♀, Christian Cox, AUMNH]; Davis Mtns, Hwy 118 between McDonald observatory and Hwy 166, 30.7049 -104.1118 ^1^, 5905ft., [APH_0935, 31/9/2008, 1♂, Christian Cox, AUMNH]; Davis Mtns, Hwy 166 at the foothills of the Davis Mtns, 30.63155 -104.278117 ^1^, 5393ft., [APH_0937, 31/9/2008, 1♂, Corey Roelke, AUMNH]; Fort Davis, 30.588211 -103.894625 ^5^, 4895ft., [APH_2466, 5/1938, 2♂, unknown, AMNH]; **Kimble**: CR182, Junction, 30.470278 -99.756389 ^1^, 1743ft., [APH_1066, 15/6/2010, 1♀, Skyler Stevens, AUMNH]; **McCulloch**: Brady, Brady Lake, 31.1235 -99.384517 ^1^, 1759ft., [APH_0827-0831, 7/2008, 4♀, 1 juv, Chris A. Hamilton, AUMNH]; **McLennan**: Waco, near Lake Waco, 31.553483 -97.203633 ^5^, 559ft., [APH_0914, 2006, 1♂, Dave Moellendorf, AUMNH]; **Oldham**: Boys Ranch area, Hwy 385, S of Canadian River, 35.500017 -102.25236 ^5^, 3222ft., [AUMS_2474, 25/10/1986, 1♂, T.R. Prentice, AUMNH]; **Parmer**: 2 miles north of Friona, 34.670063 -102.72741 ^4^, 4029ft., [APH_2184, 5/10/1961, 1♂, W.J. Gertsch, AMNH]; **Presidio**: Terlingua, 29.323867 -103.617117 ^6^, 3020ft., [APH_0863, 2006, 1♀, Dave Moellendorf, AUMNH]; unknown, 29.948067 -104.100133 ^7^, 4310ft., [APH_0870, 2006, 1♀, Dave Moellendorf, AUMNH]; **Randall**: Canyon, field due west of Canyon Crest Apts., 34.9703 -101.92336 ^2^, 3602ft., [APH_0041, 24/5/2002, 1♀, Brent E. Hendrixson, AUMNH]; FM Rd 1151 W jct. Whitaker Rd near playa lake, 35.09147 -101.75591 ^4^, 3573ft., [APH_0035, 26/9/2006, 1♂, Kari McWest, AUMNH]; **San Saba**: Colorado Bend State Park, 31.095817 -98.501567 ^1^, 1185ft., [APH_0822-0826, 6/2008, 5♀, Chris A. Hamilton, AUMNH]; [APH_0909, 6/2008, 1♀, Chris A. Hamilton, AUMNH]; [APH_0976, 6/2008, 1 juv, Chris A. Hamilton, AUMNH]; **Sherman**: 10 miles south of Texhoma, 36.399192 -101.803752 ^5^, 3510ft., [APH_2645, 9/1967, 1♂, W. Ivie, AMNH]; 6 miles south of Stratford, 36.284667 -102.049499 ^5^, 3655ft., [APH_2662, 9/1967, 1♂, W. Ivie, AMNH]; **Tarrant**: 537 Jakmar Rd, Benbrook, 32.6549 -97.47915 ^2^, 800ft., [APH_0361, 6/2008, 1♂, Laura Gleason, AUMNH]; **Travis**: 1907 Casa Grande, Austin, 30.34272 -97.86449 ^2^, 673ft., [APH_0015, 14/9/2002, 1♂, Mike Comai, AUMNH]; 5203 Rico Cove, Austin, 30.374853 -97.77368 ^2^, 885ft., [APH_0401, 5/10/2008, 1♂, Rhone McCall, AUMNH]; Austin, 30.303983 -97.839883 ^6^, 991ft., [APH_0875, 7/2008, 1♀, Dave Moellendorf, AUMNH]; Austin - City Park Rd and Pence Ln, 30.354083 -97.832567 ^1^, 843ft., [APH_0812, 6/2008, 1♀, Chris A. Hamilton, AUMNH]; [APH_0904, 6/2008, 1♂, Chris A. Hamilton, AUMNH]; Austin - Cuernavaca area in West Lake Hills, 30.34478 -97.85278 ^1^, 623ft., [APH_0159, Summer/2007, 1♂, Chris A. Hamilton, AUMNH]; [APH_0816-0819, 6/2008, 4♀, Chris A. Hamilton, AUMNH]; Austin - Red Bud trail in West Lake Hills, 30.2984 -97.80258 ^1^, 784ft., [APH_0158, Summer/2007, 1 juv, Chris A. Hamilton, AUMNH]; [APH_0813-0815, 6/2008, 3♀, Chris A. Hamilton, AUMNH]; [APH_0906-0907, 6/2008, 2♂, Chris A. Hamilton, AUMNH]; Austin area, along Wild Basin Ledge, 30.307721 -97.818971 ^2^, 774ft., [APH_0748, 5/9/2009, 1♂, Claudia Cobianchi, AUMNH]; Austin Baseball Field, 30.267153 -97.743061 ^6^, 459ft., [APH_2186, 29/5/1958, 1♀, W.H. McAlister, AMNH]; [APH_2187, 21/7/1958, 1♂, W.H. McAlister, AMNH]; Austin, Duvall and Jolleyville Rd, 30.417283 -97.750033 ^6^, 882ft., [APH_0911, 2006, 1♂, Dave Moellendorf, AUMNH]; Austin, near 290 and 71 split, 30.2425 -97.874367 ^6^, 941ft., [APH_0876, 7/2008, 1♀, Dave Moellendorf, AUMNH]; Jonestown - Reed Park, 30.4779 -97.94605 ^1^, 860ft., [APH_0160, Summer/2007, 1♀, Chris A. Hamilton, AUMNH]; [APH_0820-0821, 6/2008, 2♀, Chris A. Hamilton, AUMNH]; [APH_0908, 6/2008, 1♂, Chris A. Hamilton, AUMNH]; [APH_0949, 6/2008, 1♀, Chris A. Hamilton, AUMNH]; **Val Verde**: Comstock, 29.685683 -101.17145 ^1^, 1580ft., [APH_0833, 7/2008, 1♀, Chris A. Hamilton, AUMNH]; Del Rio - Lake Amistad, 29.496167 -101.04455 ^5^, 1147ft., [APH_0927, 2006, 1♀, Dave Moellendorf, AUMNH]; Del Rio, 29.370886 -100.895867 ^6^, 995ft., [APH_0971, 2006, 1♂, Dave Moellendorf, AUMNH]; Del Rio, on spur 454, 29.460165 -100.948547 ^5^, 1139ft., [APH_0957, 6/2006, 1♂, Dave Moellendorf, AUMNH]; Langtry, 29.809722 -101.5525 ^1^, 1272ft., [APH_1063, 18/6/2010, 1♀, Travis Fisher, AUMNH]; [APH_0958, 6/2006, 1♂, Dave Moellendorf, AUMNH]; Langtry, Hwy 90, 29.814633 -101.563317 ^1^, 1372ft., [APH_0867, 5/2008, 1♀, Corey Roelke, AUMNH]; N of Langtry on Pandale Dirt Rd, 29.828883 -101.59095 ^6^, 1425ft., [APH_0926, 2006, 1♂, Dave Moellendorf, AUMNH]; Pandale, 30.130017 -101.575733 ^1^, 1607ft., [APH_1070, 2/6/2010, 1♂, Lynn McCutchen, AUMNH]; [APH_1071, 26/6/2010, 1♂, Roxana Leija, AUMNH]; **Wichita**: 4 miles southeast of Burkburnett, 34.067561 -98.541374 ^5^, 994ft., [APH_2665, 4/7/1975, 1♂, J. Cokendolpher, AMNH]; unknown, 33.930965 -98.748117 ^7^, 1007ft., [APH_2199, 23/4/1975, 1♀, M. Nipper, AMNH]; **Wilbarger**: 12 miles west of Electra, 34.025734 -99.145057 ^5^, 1171ft., [APH_2179, 3/9/1998, 1♀, M.E. Janowski-Bell, AMNH]; [APH_2192, 3/9/1998, 1♂, M.E. Janowski-Bell, AMNH]; **Williamson**: Cedar Park, 30.5015 -97.847917 ^5^, 1018ft., [APH_0905, 2006, 1♂, Dave Moellendorf, AUMNH]; Core Hole Cave- 1 mile south Georgetown, 30.607738 -97.687453 ^5^, 787ft., [APH_2555, 3/11/1963, 1♀, J. Reddell, D. McKenzie, John Porter, AMNH]; Leander, 30.57295 -97.850467 ^5^, 972ft., [APH_0896-0897, 2006, 2♂, Dave Moellendorf, AUMNH]; Round Rock, Fern Bluff Elementary, 30.517917 -97.719917 ^4^, 806ft., [APH_0912, 6/2008, 1♂, Dave Moellendorf, AUMNH]; Taylor, 30.560167 -97.413333 ^5^, 518ft., [APH_0913, 6/2008, 1♂, Dave Moellendorf, AUMNH]; **Yoakum**: unknown rd N of Hwy-380, 33.35231 -103.01736 ^1^, 3857ft., [APH_0746, 17/6/2009, 1 juv, Zach Valois, AUMNH].

#### Distribution and natural history.

*Aphonopelma hentzi* is the most widely distributed species in the United States. Its distribution is bound to the east by the Mississippi River but these tarantulas are found in all or parts of Missouri, Arkansas, Louisiana, Kansas, Oklahoma, Texas, Colorado, New Mexico and Arizona (Fig. 55). *Aphonopelma hentzi* thrives in a variety of habitats and can be found inhabiting the following Level III Ecoregions: Interior River Valleys and Hills, Ozark Highlands, Central Irregular Plains, Alluvial Plain, South Central Plains, Ouachita Mountains, Arkansas Valley, Boston Mountains, Western Gulf Coastal Plains, Flint Hills, Central Oklahoma/Texas Plains, Western High Plains, Southwestern Tablelands, Central Great Plains, Cross Timbers, East Central Texas Plains, Arizona/New Mexico Mountains, Chihuahuan Deserts, Edwards Plateau, Southern Texas Plains, Blackland Prairies, Arizona/New Mexico Plateau, and Madrean Archipelago. *Aphonopelma hentzi* can be found in syntopy with a number of species across its distribution including *Aphonopelma armada*, *Aphonopelma gabeli*, *Aphonopelma moderatum*, *Aphonopelma moellendorfi* sp. n., *Aphonopelma parvum* sp. n., *Aphonopelma peloncillo* sp. n., and *vorhiesi*.

*Aphonopelma hentzi* exhibits significant variation in burrowing behavior across its distribution. This species is known to inhabit freestanding burrows or scrapes (burrows under rocks, wood, etc.; see Fig. 2A–C) depending on the habitat. *Aphonopelma hentzi* inhabit a wide range of habitats and elevations and exhibit phenotypic differences between those (e.g., large and robust in lower elevations or plains habitats and a smaller body with longer, thinner legs in rocky or higher elevation habitats). Depending on the locality, *Aphonopelma hentzi* can exhibit two different breeding periods: a spring/summer season (April-August) and a fall season (September-October), sometimes at the same location. Additional information about the natural history of *Aphonopelma hentzi* is provided by Smith (1995).

#### Conservation status.

*Aphonopelma hentzi* would be considered rare in Arizona due to its very limited distribution in the state but this species is probably the most abundant tarantula in the United States. These spiders thrive in many different habitats and are not uncommon in major metropolitan areas. More than 40 individuals were collected by one of the authors (BEH) from a single residential lawn in suburban Fort Worth, Texas (specimens not reported). Specimens have also been collected from well-maintained grounds at a cemetery just outside Downtown Dallas, Texas near a busy freeway. This species is secure.

#### Remarks.

Other important ratios that distinguish males: *Aphonopelma hentzi* possess a larger L3 scopulation extent (69%-86%) than *Aphonopelma armada* (48%-63%); by possessing a larger F4/M4 (≥0.92; 0.92–1.03) than *Aphonopelma armada* (≤0.92; 0.86–0.92); by possessing a larger PTl/T4 (>0.65; 0.65–0.77) than *Aphonopelma gabeli* (≤0.64; 0.58–0.64). Other important ratios distinguish females: *Aphonopelma hentzi* possess a larger L4 scopulation extent (42%-72%) than *Aphonopelma armada* (28%–43%, with slight overlap); by possessing a smaller L1/L3 (≤1.14; 1.07–1.14) than *Aphonopelma moderatum* (>1.14; 1.14–1.24, with slight overlap). For both males and females, certain morphometrics have potential to be useful, though due to the amounts of variation, small number of specimens, and the small differences between species, no others are claimed to be significant at this time (see Suppl. material 2). During evaluation of traditional PCA morphospace, males of *Aphonopelma hentzi* separate in PCA morphological space from *Aphonopelma gabeli*, *Aphonopelma moderatum*, and *Aphonopelma moellendorfi* along PC1~2, but do not separate from *Aphonopelma anax*, *Aphonopelma armada*, *Aphonopelma peloncillo*, or *Aphonopelma vorhiesi*. Female *Aphonopelma hentzi* do not separate from *Aphonopelma anax*, *Aphonopelma armada*, *Aphonopelma gabeli*, *Aphonopelma moderatum*, *Aphonopelma peloncillo*, or *Aphonopelma vorhiesi*. Females of *Aphonopelma moellendorfi* are unknown and cannot be compared. Interestingly, *Aphonopelma hentzi* males separate from *Aphonopelma gabeli*, *Aphonopelma marxi*, *Aphonopelma moderatum*, *Aphonopelma moellendorfi*, *Aphonopelma parvum*, and *Aphonopelma peloncillo* in three-dimensional PCA morphospace (PC1~PC2~PC3), but do not separate from *Aphonopelma anax*, *Aphonopelma armada*, *Aphonopelma chalcodes*, or *Aphonopelma vorhiesi*. *Aphonopelma hentzi* females separate from *Aphonopelma marxi* and *Aphonopelma parvum*, but do not separate from *Aphonopelma anax*, *Aphonopelma armada*, *Aphonopelma chalcodes*, *Aphonopelma gabeli*, *Aphonopelma moderatum*, *Aphonopelma peloncillo*, or *Aphonopelma vorhiesi*. PC1, PC2, and PC3 explain ≥87% of the variation in male analyses and ≥96% of the variation in female analyses.

Of particular note, Warriner (2008) determined that the type locality for *Aphonopelma hentzi* was near the Wichita Mountains.in Comanche County, Oklahoma where the Marcy Expedition likely camped in May 1852. Even though we have examined material from this area, we have chosen not to designate a new neotype at this time because Smith’s (1995) neotype is conspecific with this material and we were unable to secure adults. We examined most of the holotypes and/or freshly collected topotypic material of *Aphonopelma clarki*, *Aphonopelma coloradanum*, *Aphonopelma echinum*, *Aphonopelma gurleyi*, *Aphonopelma harlingenum*, *Aphonopelma odelli*, *Aphonopelma waconum*, and *Aphonopelma wichitanum*. Our morphological and molecular analyses fail to recognize these eight species as separate, independently evolving lineages. As a consequence, we consider *Aphonopelma clarki*, *Aphonopelma coloradanum*, *Aphonopelma echinum*, *Aphonopelma gurleyi*, *Aphonopelma harlingenum*, *Aphonopelma odelli*, *Aphonopelma waconum*, and *Aphonopelma wichitanum* junior synonyms of *Aphonopelma hentzi*. It is also important to note that the paratype of *Aphonopelma harlingenum* that is found in the same jar as the *Aphonopelma harlingenum* holotype is a female of *Aphonopelma anax* (see spermathecae in Fig. 12C).

Mitochondrial DNA (*CO1*) identifies *Aphonopelma hentzi* as a polyphyletic group with respect to *Aphonopelma moderatum*, *Aphonopelma anax*, and *Aphonopelma armada* (Fig. 7). A second lineage of *Aphonopelma hentzi* is embedded within a secondary lineage of *Aphonopelma anax*; both lineages were previously identified as putative cryptic species (Hamilton et al. 2014). Based on the AE results, it appears that *CO1* is not effective at delimiting species boundaries within this group.

### Aphonopelma icenoglei

Taxon classificationAnimaliaAraneaeTheraphosidae

Hamilton, Hendrixson & Bond sp. n.

http://zoobank.org/5D5AC92B-291D-4902-B7F1-918BD05B331D

**Figure 56.**
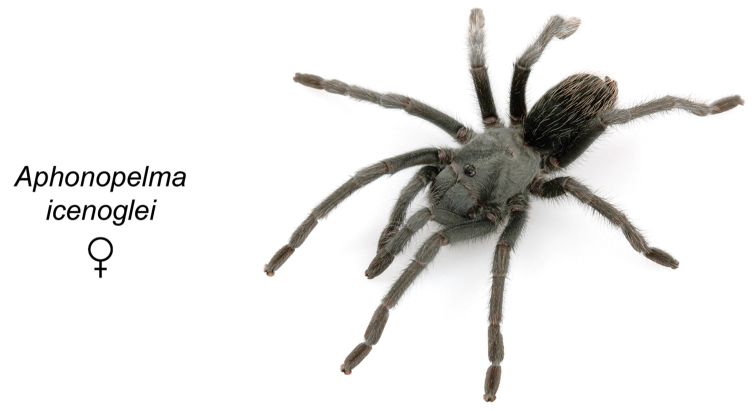
*Aphonopelma icenoglei* sp. n. live photograph. Female - APH_3146. Do not have a photograph of a live male specimen.

**Figure 57.**
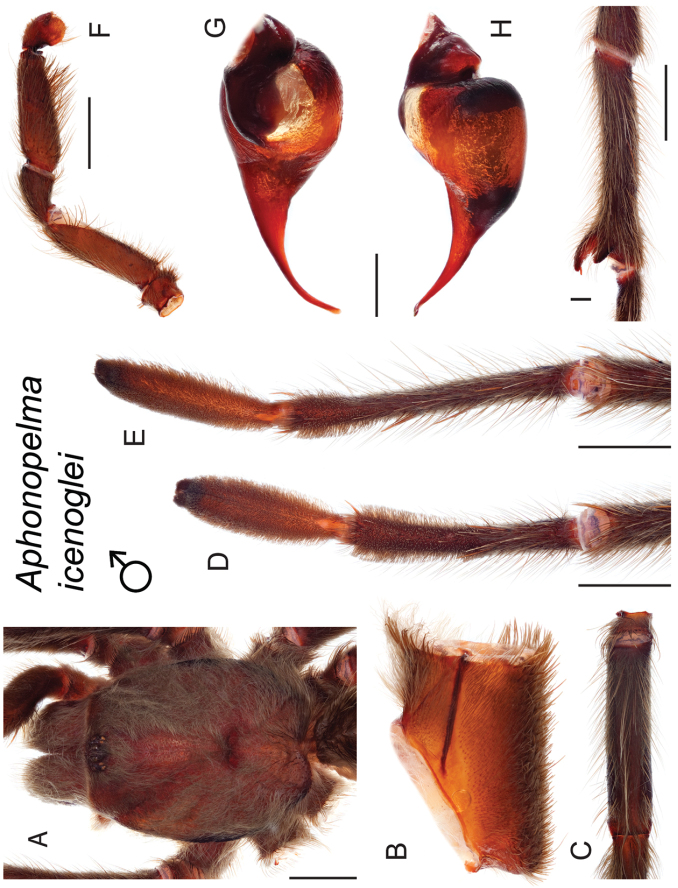
*Aphonopelma icenoglei* sp. n. **A–I** male holotype, APH_2396 **A** dorsal view of carapace, scale bar = 2.5mm **B** prolateral view of coxa I **C** dorsal view of femur III **D** ventral view of metatarsus III, scale bar = 2.5mm **E** ventral view of metatarsus IV, scale bar = 2.5mm **F** prolateral view of L pedipalp and palpal tibia, scale bar = 3mm **G** dorsal view of palpal bulb **H** retrolateral view of palpal bulb, scale bar = 0.5mm **I** prolateral view of tibia I (mating clasper), scale bar = 2.5mm.

**Figure 58.**
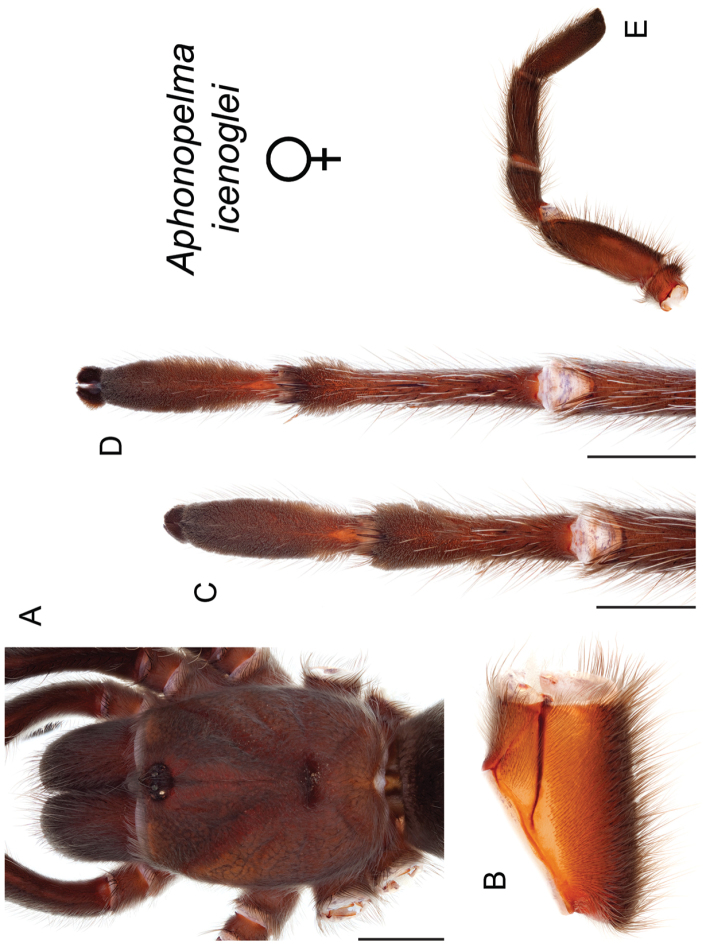
*Aphonopelma icenoglei* sp. n. **A–E** female paratype, APH_1562 **A** dorsal view of carapace, scale bar = 3mm **B** prolateral view of coxa I **C** ventral view of metatarsus III, scale bar = 2mm **D** ventral view of metatarsus IV, scale bar = 2.5mm **E** prolateral view of L pedipalp and palpal tibia.

**Figure 59.**
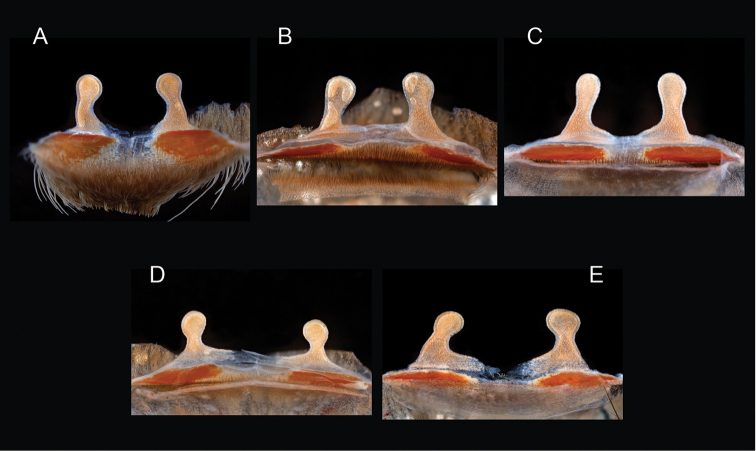
*Aphonopelma icenoglei* sp. n. **A–E** cleared spermathecae **A** APH_0761 **B** APH_0885 **C** APH_1562 **D** APH_2393 **E** APH_3118.

**Figure 60.**
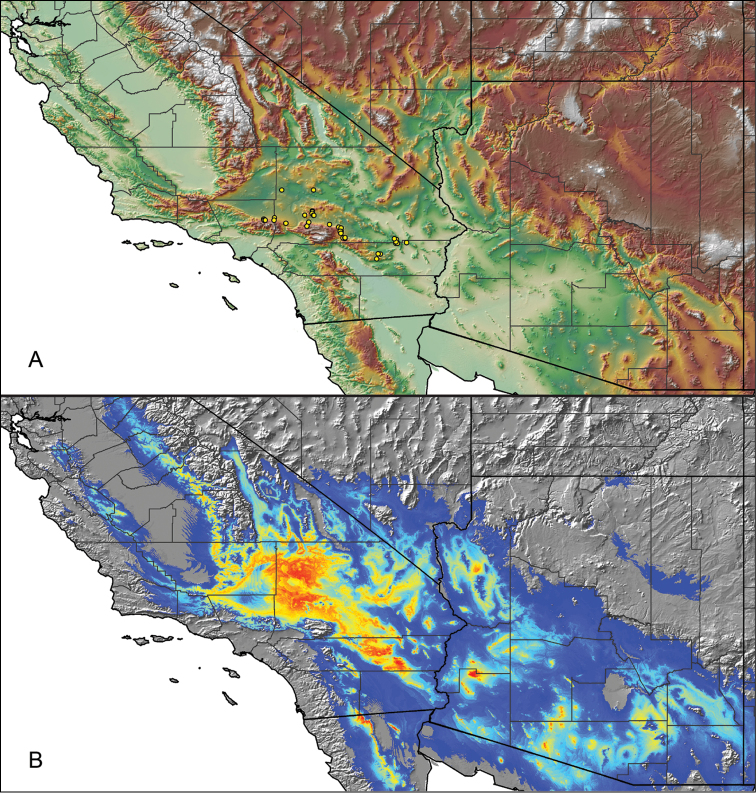
*Aphonopelma icenoglei* sp. n. **A** distribution of known specimens **B** predicted distribution; warmer colors (red, orange, yellow) represent areas of high probability of occurrence, cooler colors (blue shades) represent areas of low probability of occurrence.

Figures 56 , 57 , 58 , 59 , 60

Aphonopelma mojave Prentice, 1997 (in part): 161.

#### Types.

Male holotype (APH_2396) collected 7.5 miles north of Pipes Canyon Rd., San Bernardino Co., California, 34.303363 -116.440492 ^5^, elev. 3133ft., 14.iv.1991, coll. T.R. Prentice; deposited in AMNH. Paratype female (APH_1562) from 0.6 miles S Hwy-62 on La Contenta Rd., San Bernardino Co., California, 34.126565 -116.368852 ^1^, elev. 3170ft., 25.x.2012, coll. Brent E. Hendrixson; deposited in AUMNH. Paratype male (AUMS_2643) from near junction of Main Rd. and past Cottonwood Road by Eagle Mtn. mine, 33.828312 -115.756448 ^5^, elev. 2476ft., xii.1999, coll. unknown; deposited in AUMNH. Paratype female (APH_2393) from Apple Valley, 2 miles south of Highway 18, Milpas Dr., San Bernardino Co., California, 34.53717 -117.103251 ^4^, elev. 3140ft., 5.v.1992, coll. T.R. Prentice; deposited in AMNH.

#### Etymology.

The specific epithet is a patronym in recognition of Wendell Icenogle, an arachnologist and prolific collector of North American mygalomorph spiders. This work benefitted substantially from his help collecting specimens and his wealth of knowledge concerning tarantulas in the United States.

#### Diagnosis.

*Aphonopelma icenoglei* (Fig. 56) is a member of the *paloma* species group and can be distinguished by a combination of morphological, molecular, and geographic characteristics. Nuclear and mitochondrial DNA identifies *Aphonopelma icenoglei* as a strongly supported monophyletic lineage (Figs 7–8) that is a sister lineage to *Aphonopelma mojave*, *Aphonopelma atomicum* sp. n., *Aphonopelma prenticei* sp. n., and *Aphonopelma joshua*. *Aphonopelma icenoglei* can easily be differentiated from *Aphonopelma iodius* by its smaller size and limited extent of scopulation on metatarsus IV, and from *Aphonopelma atomicum* and *Aphonopelma prenticei* by locality. The most significant measurements that distinguish male *Aphonopelma icenoglei* from its closely related phylogenetic and syntopic species are F3 and the extent of scopulation on metatarsus IV. Male *Aphonopelma icenoglei* can be distinguished by possessing a larger F3L/W (≥3.51; 3.51–3.90) than *Aphonopelma atomicum* (≤3.40; 2.92–3.40) and *Aphonopelma prenticei* (≤3.27; 2.76–3.27); a larger PTl/F3 (≥0.67; 0.67–0.71) than *Aphonopelma joshua* (≤0.61; 0.55–0.61) and *Aphonopelma xwalxwal* sp. n. (≤0.63; 0.59–0.63); and a smaller L4 scopulation extent (31%-46%) than *Aphonopelma iodius* (62%-88%). There are no significant measurements that separate male *Aphonopelma icenoglei* from *Aphonopelma mojave*. The most significant measurements that distinguish female *Aphonopelma icenoglei* from its closely related phylogenetic and syntopic species are M4 and the extent of scopulation on metatarsus IV. Female *Aphonopelma icenoglei* can be distinguished by possessing a larger F1/M4 (≥1.07; 1.07–1.23) than *Aphonopelma joshua* (≤1.04; 0.98–1.04); a larger A3/M4 (≥0.63; 0.63–0.70) than *Aphonopelma atomicum* (≤0.58; 0.57–0.58) and *Aphonopelma joshua* (≤0.59; 0.50–0.59); a smaller Cl/F1 (≤1.16; 1.07–1.16) than *Aphonopelma prenticei* (≥1.17; 1.17–1.33); and a smaller L4 scopulation extent (27%–42%) than *Aphonopelma iodius* (59%–83%). There are no significant measurements that separate female *Aphonopelma icenoglei* from *Aphonopelma mojave*. *Aphonopelma icenoglei* is most similar (morphologically and geographically) to *Aphonopelma mojave* but these two species are phylogenetically distinct and probably are not sister taxa (Figs 7, 8).

#### Description of male holotype

(APH_2396; Fig. 57). *Specimen preparation and condition*: Specimen originally collected from burrow and preserved in unknown percentage of ethanol; original coloration faded due to preservation. Left legs I, III, IV, and left pedipalp removed for measurements and photographs; stored in vial with specimen. No tissue for DNA. *General coloration*: Faded black/brown. *Cephalothorax*: Carapace 8.228 mm long, 6.965 mm wide; densely clothed with faded pubescence, appressed to surface; fringe covered in long setae not closely appressed to surface, hirsute appearance; foveal groove medium deep and straight; pars cephalica region rises very gradually from foveal groove on a straight plane towards the ocular area; AER very slightly procurved, PER recurved; normal sized chelicerae; clypeus extends slightly on a curve; LBl 1.051, LBw 1.468; sternum hirsute, clothed with faded, densely packed, short setae. *Abdomen*: Densely clothed in short black/brown pubescence with numerous longer, lighter setae interspersed (generally red or orange *in situ*); dense dorsal patch of black Type I urticating bristles (Cooke et al. 1972) - smaller and distinct from large species. *Legs*: Hirsute; densely clothed in faded pubescence. Metatarsus I straight. F1 7.605; F1w 1.786; P1 2.994; T1 6.779; M1 5.848; A1 4.17; F3 7.202; F3w 1.851; P3 2.613; T3 5.625; M3 6.742; A3 4.464; F4 8.432; F4w 1.872; P4 2.869; T4 7.231; M4 8.368; A4 4.938; femur III is normal. All tarsi fully scopulate. Extent of metatarsal scopulation: leg III (SC3) = 62.6%; leg IV (SC4) = 35.1%. Two ventral spinose setae on metatarsus III; five ventral spinose setae on metatarsus IV; one prolateral spinose seta on tibia I; one megaspine on the apex on the retrolateral branch of the tibial apophyses. *Coxa I*: Prolateral surface covered by fine, hair-like setae. *Pedipalps*: Hirsute; densely clothed in the same setal color as the other legs, with numerous longer ventral setae; one spinose seta at the apical, prolateral femur and three prolateral spinose setae on the palpal tibia; PTl 4.938, PTw 1.77. When extended, embolus tapers with a curve to the retrolateral side; embolus slender, no keels; distinct dorsal and ventral transition from bulb to embolus.

**Variation (5).** Cl 7.326–8.228 (7.831±0.15), Cw 6.609–7.307 (6.976±0.11), LBl 1.031–1.069 (1.046±0.01), LBw 1.092–1.475 (1.271±0.08), F1 7.605–8.996 (8.292±0.24), F1w 1.703–1.938 (1.821±0.05), P1 2.909–3.301 (3.067±0.07), T1 6.679–7.727 (7.204±0.21), M1 5.848–7.032 (6.459±0.22), A1 4.012–4.818 (4.338±0.14), L1 length 27.396–31.441 (29.36±0.78), F3 6.881–7.558 (7.203±0.14), F3w 1.763–2.145 (1.963±0.08), P3 2.477–2.771 (2.579±0.05), T3 5.625–6.187 (5.88±0.12), M3 6.742–7.609 (6.986±0.16), A3 4.448–4.952 (4.569±0.1), L3 length 26.376–28.76 (27.216±0.48), F4 7.919–9.094 (8.46±0.22), F4w 1.691–1.902 (1.836±0.04), P4 2.549–2.869 (2.77±0.06), T4 6.914–7.825 (7.283±0.16), M4 8.368–9.267 (8.657±0.17), A4 4.702–5.595 (5.036±0.16), L4 length 30.775–33.887 (32.205±0.65), PTl 4.758–5.115 (4.953±0.07), PTw 1.548–1.77 (1.684±0.04), SC3 ratio 0.626–0.788 (0.704±0.03), SC4 ratio 0.312–0.456 (0.376±0.02), Coxa I setae = very thin tapered, F3 condition = normal.

#### Description of female paratype

(APH_1562; Figs 58–59). *Specimen preparation and condition*: Specimen collected live from burrow, preserved in 80% ethanol; original coloration faded due to preservation. Left legs I, III, IV, and pedipalp removed for photographs and measurements; stored in vial with specimen. Right legs III & IV removed for DNA and stored at -80°C in the AUMNH (Auburn, AL). Genital plate with spermathecae removed and cleared, stored in vial with specimen. *General coloration*: Faded black/brown. *Cephalothorax*: Carapace 7.943 mm long, 6.522 mm wide; Hirsute, densely clothed with short faded black/brown pubescence closely appressed to surface; fringe densely covered in slightly longer setae; foveal groove medium deep and slightly procurved; pars cephalica region gently rises from thoracic furrow, arching anteriorly toward ocular area; AER slightly procurved, PER very slightly recurved; chelicerae robust, clypeus extends forward on a curve; LBl 1.353, LBw 1.435; sternum hirsute, clothed with short faded setae. *Abdomen*: Densely clothed dorsally in short faded black setae with longer, lighter setae (generally red or orange *in situ*) focused near the urticating patch; dense dorsal patch of black Type I urticating bristles (Cooke et al. 1972) - smaller and distinct from large species. *Spermathecae*: Paired and separate, with capitate bulbs widening towards the bases; not fused. *Legs*: Hirsute; densely clothed in short faded black/brown pubescence; F1 6.983; F1w 1.974; P1 2.993; T1 5.757; M1 4.266; A1 3.976; F3 5.884; F3w 1.834; P3 2.608; T3 4.443; M3 4.682; A3 4.092; F4 7.463; F4w 1.906; P4 2.765; T4 5.853; M4 6.269; A4 4.678. All tarsi fully scopulate. Extent of metatarsal scopulation: leg III (SC3) = 63.5%; leg IV (SC4) = 27.1%. One ventral and one prolateral spinose seta on metatarsus III; four ventral spinose setae and one prolateral spinose seta on metatarsus IV. *Coxa I*: Prolateral surface covered by very thin tapered and fine, hair-like setae. *Pedipalps*: Densely clothed in the same setal color as the other legs; one spinose seta on the apical, prolateral femur, four prolateral (two at the apical, prolateral border with the tarsus) spinose setae and one ventral spinose seta on the tibia.

**Variation (5).** Cl 7.424–8.188 (7.89±0.13), Cw 6.522–7.385 (6.993±0.16), LBl 1.285–1.353 (1.322±0.01), LBw 1.368–1.615 (1.453±0.05), F1 6.565–7.644 (6.966±0.18), F1w 1.974–2.131 (2.049±0.03), P1 2.716–3.161 (2.988±0.08), T1 5.185–6.381 (5.695±0.2), M1 4.163–4.72 (4.406±0.1), A1 3.568–3.976 (3.708±0.08), L1 length 22.432–25.667 (23.763±0.54), F3 5.362–6.315 (5.807±0.16), F3w 1.766–1.979 (1.849±0.04), P3 2.217–2.711 (2.47±0.1), T3 3.995–4.755 (4.305±0.13), M3 4.216–4.864 (4.54±0.11), A3 3.90–4.389 (4.041±0.09), L3 length 20.101–22.92 (21.164±0.51), F4 6.84–8.227 (7.376±0.24), F4w 1.766–1.967 (1.897±0.03), P4 2.625–3.112 (2.836±0.09), T4 5.477–6.325 (5.799±0.14), M4 5.589–6.77 (6.14±0.19), A4 4.174–4.678 (4.436±0.1), L4 length 25.043–28.961 (26.587±0.68), SC3 ratio 0.636–0.726 (0.691±0.02), SC4 ratio 0.272–0.419 (0.365±0.03), Coxa I setae = very thin tapered. Spermathecae variation can be seen in Figure 59.

#### Material examined.

**United States: California: Los Angeles**: 0.66 miles N Ft Tejon Rd on Valyermo Rd, 34.468812 -117.860377 ^1^, 3532ft., [APH_0756-0757, 4/10/2009, 2 juv, Brent E. Hendrixson, Thomas Martin, AUMNH]; 0.67 miles S Hwy-18 on 263rd St, 34.48904 -117.661058 ^1^, 3461ft., [APH_1202, 30/7/2010, 1♀, Brent E. Hendrixson, Brendon Barnes, Nate Davis, AUMNH]; 1.15 miles N of Valyermo, Bobs Gap Rd, 34.448937 -117.82921 ^4^, 3920ft., [AUMS_2511, 4/10/1992, 1♂, T.R. Prentice, AUMNH]; Piñon Hills off Hwy 138, 34.448134 -117.668735 ^5^, 4002ft., [AUMS_2515, 1993, 1♂, unknown, AUMNH]; Valyermo, 34.446108 -117.852286 ^5^, 4403ft., [APH_2400, 28/10/1989, 1♂, T.R. Prentice, AMNH]; **Riverside**: Eagle Mountain area, 2 miles E of Cottonwood Springs, 33.733266 -115.784522 ^5^, 3485ft., [AUMS_2464, 4/4/1989, 1♂, T.R. Prentice, AUMNH]; Joshua Tree National Park, 33.82491 -115.720588 ^6^, 2423ft., [APH_0885, 2007, 1♀, Josh Richards, AUMNH]; near junction of Main Rd. and past Cottonwood Road by Eagle Mt. mine, 33.828312 -115.756448 ^5^, 2476ft., [AUMS_2643, 12/1999, 1♂, unknown, AUMNH]; **San Bernardino**: 0.5 miles SE Mikiska Blvd along Hwy-247 (NW of Landers), 34.319594 -116.48102 ^1^, 3406ft., [APH_0761-0762, 5/10/2009, 2 juv, Brent E. Hendrixson, Thomas Martin, AUMNH]; 0.6 miles S Hwy-62 on La Contenta Rd, 34.126565 -116.368852 ^1^, 3170ft., [APH_1562, 25/10/2012, 1♀, Brent E. Hendrixson, AUMNH]; 1 miles W Ft. Irwin Rd on Irwin Rd, 34.998665 -116.945203 ^1^, 2658ft., [APH_1320, 30/7/2011, 1♀, Brent E. Hendrixson, Brendon Barnes, Nate Davis, Jake Storms, AUMNH]; 1.1 miles NE Lucerne Valley Cutoff along Hwy-247 (Barstow Rd), 34.614513 -116.968992 ^1^, 3239ft., [APH_0760, 5/10/2009, 1 juv, Brent E. Hendrixson, Thomas Martin, AUMNH]; 12.2 miles west of junction of 177 and Highway 62, 34.046346 -115.432997 ^4^, 2205ft., [APH_2391, 12/11/1989, 1♀, T.R. Prentice, AMNH]; [APH_2398, 24/11/1989, 1♂, T.R. Prentice, AMNH]; [APH_2402, 24/11/1989, 1♂, T.R. Prentice, AMNH]; 2.6 miles S Phelan Rd on Baldy Mesa Rd, 34.389404 -117.454546 ^1^, 3904ft., [APH_1577-1578, 6/11/2012, 1♀, 1 juv, Brent E. Hendrixson, AUMNH]; 3.7 miles north of Pipes Canyon Rd. Highway 247 Access Rd., 34.243922 -116.440137 ^4^, 3484ft., [APH_2395, 18/4/1991, 1♀, T.R. Prentice, AMNH]; 7.5 miles north of Pipes Canyon Rd., 34.303363 -116.440492 ^5^, 3133ft., [APH_2396, 14/4/1991, 1♂, T.R. Prentice, AMNH]; along Hwy-62, west of Coxcomb Mtns, 34.090719 -115.424593 ^1^, 1767ft., [APH_1584-1585, 7/11/2012, 2♀, Brent E. Hendrixson, AUMNH]; Apple Valley, 2 miles south of Highway 18, Milpas Dr., 34.53717 -117.103251 ^4^, 3140ft., [APH_2393, 5/5/1992, 1♀, T.R. Prentice, AMNH]; Cadiz Valley, 34.03779 -115.238314 ^5^, 1247ft., [APH_2389, 24/11/1989, 1♂, T.R. Prentice, AMNH]; Coxcomb Mtns, Highway 177 and Highway 62 - 12.2 miles west of junction, 34.026317 -115.431625 ^5^, 2743ft., [APH_2397, 31/1/1991, 1♀, T.R. Prentice, AMNH]; E of Apple Valley, High Rd and Castle Rock Rd, 34.40636 -117.033581 ^1^, 3551ft., [APH_1201, 30/7/2010, 1 juv, Brent E. Hendrixson, Brendon Barnes, Nate Davis, AUMNH]; Hondo Rd. between Pipes canyon Rd. and New Dixie Mine Rd., 1 mile west of highway 247, 34.244106 -116.456685 ^5^, 3681ft., [APH_2392, 14/4/1990, 1♀, T.R. Prentice, AMNH]; Hwy 177 and 62 Jct, 12.2 miles west, 34.105603 -115.45904 ^5^, 2100ft., [AUMS_2541, 31/1/1991, 1♂, T.R. Prentice, AUMNH]; Joshua Tree National Park, northeast corner west of Coxcomb Mtns, off Highway 62, 34.026111 -115.404444 ^5^, 2940ft., [APH_2401, 2/11/1991, 1♀, T.R. Prentice, AMNH]; Kramer Junction (near jct. Hwy-58 and Hwy-395), 34.999749 -117.528159 ^1^, 2443ft., [APH_1321-1322, 30/7/2011, 1♀, 1 juv, Brent E. Hendrixson, Brendon Barnes, Nate Davis, Jake Storms, AUMNH]; N of Lucerne Valley, off Hwy 247 (Barstow Rd.), 34.5345 -116.943833 ^1^, 2864ft., [APH_3117-3118, 3/12/2012, 1♀, 1 juv, Chris A. Hamilton, Jason Bond, AUMNH]; North Lucerne Valley, Highway 247, 34.573636 -116.970782 ^5^, 3077ft., [APH_2419, 26/10/1991, 1♂, T.R. Prentice, AMNH]; North Lucerne Valley, Highway 247, 20 miles south of Barstow, 34.598835 -116.966024 ^5^, 3130ft., [APH_2394, 16/10/1990, 1♂, S. Kutcher, AMNH]; [APH_2417, 16/10/1990, 1♂, S. Kutcher, AMNH]; North of pipes Canyon Rd., Hondo Rd., 34.244106 -116.456685 ^5^, 3681ft., [APH_2399, 14/4/1991, 1♀, T.R. Prentice, AMNH]; off Hwy 247, N of Lucerne Valley, 34.53234 -116.93977 ^1^, 2848ft., [APH_3146-3150, 10/11/2013, 4♀, 1 juv, Chris A. Hamilton, Brent E. Hendrixson, Molly Taylor, AUMNH]; off Hwy 247, west side of road, S of La Brisa Dr. and N of Pipes Canyon Rd, 34.20272 -116.44434 ^1^, 3585ft., [APH_3151-3153, 10/11/2013, 1♀, 2 juv, Chris A. Hamilton, Brent E. Hendrixson, Molly Taylor, AUMNH]; Rattlesnake Spring Rd., off highway 247 toward Rattlesnake Rd and Two Hole Spring, 34.367051 -116.652323 ^5^, 3153ft., [APH_2416, 1/11/1989, 1♀, T.R. Prentice, AMNH]; South of Apple Valley, up Coxey Rd. into San Bernardino Mtns, 34.338472 -117.065683 ^5^, 5761ft., [APH_2418, 4/11/1989, 1♀, T.R. Prentice, AMNH]; W side of La Contenta Rd, off Hwy 62, E of Yucca Valley, 34.126055 -116.368585 ^1^, 3297ft., [APH_3154-3156, 10/11/2013, 2♀, 1 juv, Chris A. Hamilton, Brent E. Hendrixson, Molly Taylor, AUMNH]; Yucca Trail (Road), 3 miles east of Yucca Valley, 34.120528 -116.404976 ^5^, 3301ft., [APH_2710, 2/8/1973, 1♂, W. Icenogle, AMNH]; Yucca Valley - La Contenta - west side, 34.11243 -116.370359 ^5^, 3481ft., [APH_2415, 2/11/1991, 1♀, T.R. Prentice, AMNH].

#### Distribution and natural history.

*Aphonopelma icenoglei* is known from portions of the southern Mojave (Fig. 1 H) and northwestern Sonoran deserts (Colorado Desert subdivision) in California along the northern foothills of the San Gabriel and San Bernardino Mountains, the southwestern quadrant of San Bernardino County, and east to the northeastern corner of Joshua Tree National Park near the Coxcomb Mountains (Fig. 60). *Aphonopelma icenoglei* can be found inhabiting the Mojave Basin and Range Level III Ecoregion, and are likely restricted by the lower elevation Sonoran Basin and Range. The species distribution model suggests that suitable habitat for this species is present throughout the Mojave Desert (Fig. 60B) in areas already occupied by closely-related species; more extensive sampling along the bajadas of eastern Riverside County is required to determine the southern extent of this spider’s range in the Colorado Desert. Specimens have been collected at elevations between 545 and 1220 meters. *Aphonopelma icenoglei* can be found in syntopy with *Aphonopelma iodius* throughout much of its range, and may also co-occur with *Aphonopelma mojave* near Kramer Junction and *Aphonopelma joshua* in areas around Joshua Tree National Park. Burrow entrances are generally surrounded by a distinct mound or turret made of excavated soil and silk (Fig. 2D–E). Mating occurs during daylight hours in autumn (October–November).

#### Conservation status.

*Aphonopelma icenoglei* is not morphologically distinct from *Aphonopelma mojave* but is genetically unique and should be considered important. The species is moderately common but may experience threats to some populations due to human encroachment and development in portions of the Mojave Desert closest to Los Angeles.

#### Remarks.

Other important ratios that distinguish *Aphonopelma icenoglei* males: *Aphonopelma icenoglei* possess a larger F1/M4 (≥0.90; 0.90–1.02) than *Aphonopelma joshua* (≤0.84; 0.78–0.84) and *Aphonopelma xwalxwal* (≤0.84; 0.80–0.84); important ratios that distinguish females: *Aphonopelma icenoglei* possess a larger A3/T4 (≥0.68; 0.68–0.71) than *Aphonopelma atomicum* (≤0.63; 0.59–0.63) and *Aphonopelma joshua* (≤0.63; 0.55–0.63). Certain morphometrics have potential to be useful, though due to the amounts of variation, small number of specimens, and the small differences between species, no other are claimed to be significant at this time (see Suppl. material 2). During evaluation of traditional two-dimensional PCA morphospace and three-dimensional PCA morphospace (PC1~PC2~PC3), males of *Aphonopelma icenoglei* separate from *Aphonopelma iodius*, *Aphonopelma joshua*, and *Aphonopelma xwalxwal* along PC1~2, but do not separate from *Aphonopelma atomicum*, *Aphonopelma mojave*, or *Aphonopelma prenticei*. Female *Aphonopelma icenoglei* do not separate from *Aphonopelma iodius* or any other miniature species (*Aphonopelma atomicum*, *Aphonopelma mojave*, or *Aphonopelma prenticei*) in two-dimensional morphological space, but do separate from *iodius* in three-dimensional morphospace. There are no known female *Aphonopelma xwalxwal* at this time to compare. PC1, PC2, and PC3 explain ≥97% of the variation in all analyses.

### Aphonopelma iodius

Taxon classificationAnimaliaAraneaeTheraphosidae

(Chamberlin & Ivie, 1939)

**Figure 61.**
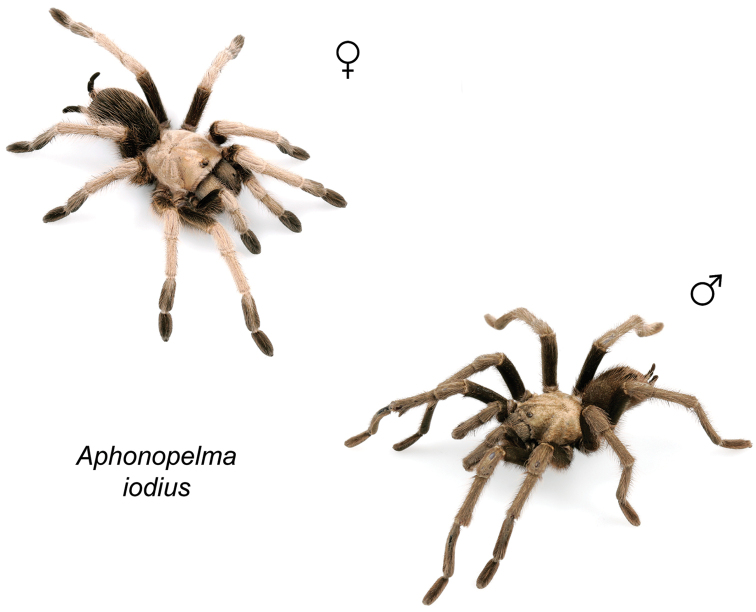
*Aphonopelma iodius* (Chamberlin & Ivie, 1939) specimens, live photographs. Female (L) - APH_3201; Male (R) - APH_3202.

**Figure 62.**
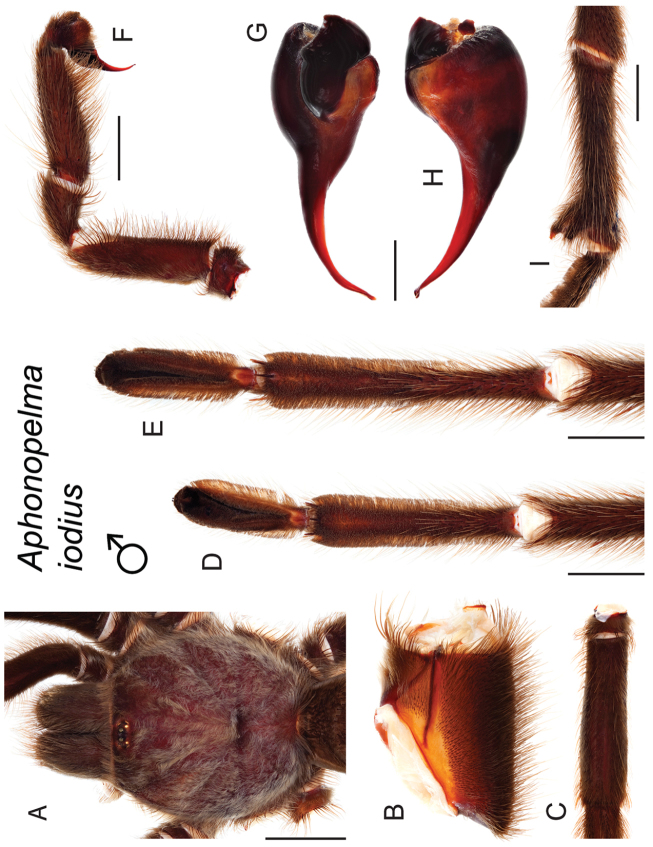
*Aphonopelma iodius* (Chamberlin & Ivie, 1939). **A–I** male specimen, APH_2015 **A** dorsal view of carapace, scale bar = 7mm **B** prolateral view of coxa I **C** dorsal view of femur III **D** ventral view of metatarsus III, scale bar = 5mm **E** ventral view of metatarsus IV, scale bar = 4.5mm **F** prolateral view of L pedipalp and palpal tibia, scale bar = 4.5mm **G** dorsal view of palpal bulb **H** retrolateral view of palpal bulb, scale bar = 1mm **I** prolateral view of tibia I (mating clasper), scale bar = 4.5mm.

**Figure 63.**
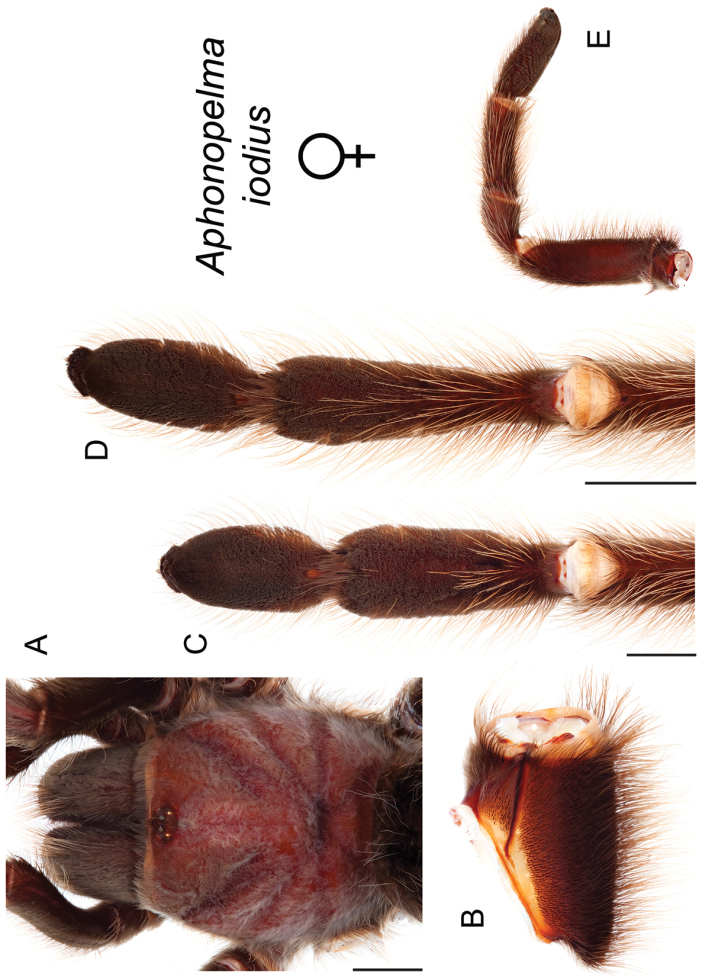
*Aphonopelma iodius* (Chamberlin & Ivie, 1939). **A–E** female specimen, APH_2016 **A** dorsal view of carapace, scale bar = 5.5mm **B** prolateral view of coxa I **C** ventral view of metatarsus III, scale bar = 4mm **D** ventral view of metatarsus IV, scale bar = 5mm **E** prolateral view of L pedipalp and palpal tibia.

**Figure 64.**
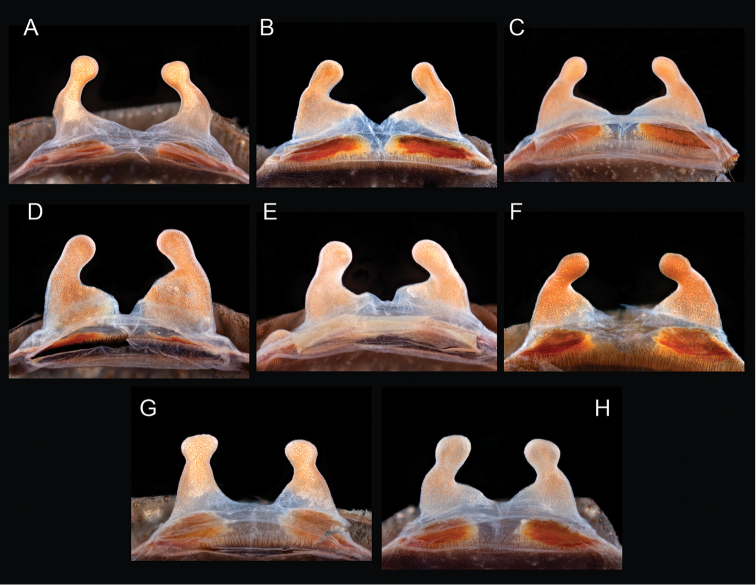
*Aphonopelma iodius* (Chamberlin & Ivie, 1939). **A–H** cleared spermathecae **A** APH_0313 **B** APH_0985 **C** APH_0996 **D** APH_0997 **E** APH_1004 **F** APH_1006 **G** APH_1082 **H** APH_1083.

**Figure 65.**
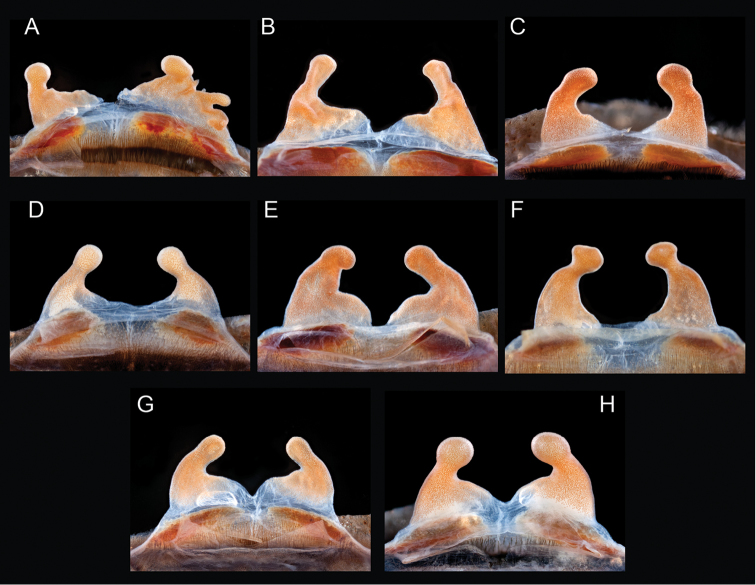
*Aphonopelma iodius* (Chamberlin & Ivie, 1939). **A–H** cleared spermathecae **A** APH_1091 **B** APH_1094 **C** APH_1220 **D** APH_2010 **E** APH_2016 **F** APH_2018 **G** APH_2030 **H** APH_3201.

**Figure 66.**
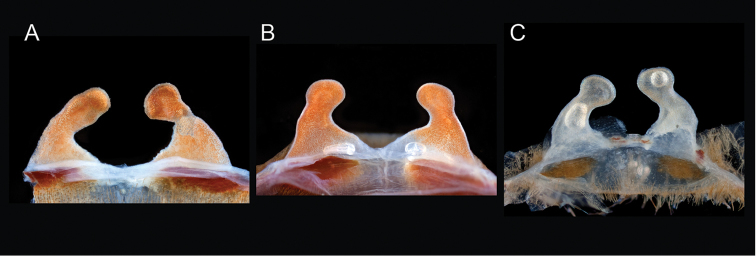
*Aphonopelma iodius* (Chamberlin & Ivie, 1939). **A–C** cleared spermathecae **A** AUMS_2386 **B** AUMS_3310 **C** *angusi* allotype.

**Figure 67.**
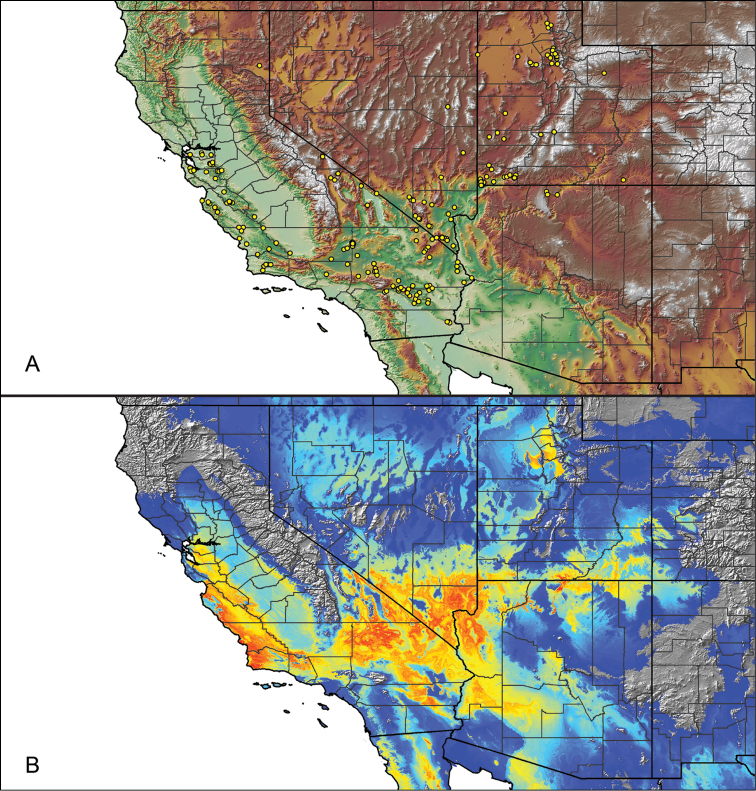
*Aphonopelma iodius* (Chamberlin & Ivie, 1939). **A** distribution of known specimens **B** predicted distribution; warmer colors (red, orange, yellow) represent areas of high probability of occurrence, cooler colors (blue shades) represent areas of low probability of occurrence.

Figures 61 , 62 , 63 , 64 , 65 , 66 , 67 ; Suppl. material 4

Delopelma iodius Chamberlin & Ivie, 1939: 6; male holotype from 2 miles W of Castle Cliffs, Washington Co., Utah, 37.072028 -113.919773^4^, elev. 3663ft., 27.xi.1936, coll. unknown. Paratype males from Zion National Park, Washington Co., Utah, 37.205521 -112.983664^5^, elev. 3973ft., no collecting date, coll. unknown; deposited in AMNH. [examined]
Rhechostica iodius Raven, 1985: 149.
Aphonopelma iodium Smith, 1995: 115.
Aphonopelma iodium Prentice, 1997: 162.
Aphonopelma iodius (spelling change; Platnick, World Spider Catalog)
Aphonopelma angusi Chamberlin, 1940: 21; male holotype from two miles west of Beaver Dam Mts., on Beaver Dam slope, Washington Co., Utah, 37.150885 -113.917096^6^, elev. 6332ft., 7.x.1939, coll. Dr. Angus M. Woodbury, Ross Hardy, Harold Higgins, and Robert Pendleton; deposited in AMNH. [examined]
Aphonopelma angusi Smith, 1995: 72. **previously synonymized by Prentice, 1997: 162.**
Delopelma melanius Chamberlin & Ivie, 1939: 5; male holotype and female allotype from Salt Lake City, Salt Lake Co., Utah, 40.760779 -111.891047^6^, elev. 4284ft., no collecting date, coll. unknown; deposited in AMNH. [examined]
Aphonopelma melanium Smith, 1995: 120. **previously synonymized by Prentice, 1997: 162.**
Aphonopelma nevadanum Chamberlin, 1940: 12; male holotype from Searchlight, Clark Co., Nevada, 35.465269 -114.919701^5^, elev. 3590ft., 2.xii.1930, coll. Geo. Carter; deposited in AMNH. [examined]
Aphonopelma nevadanum Smith, 1995: 125. **previously synonymized by Prentice, 1997: 162.**
Aphonopelma brunnius Chamberlin, 1940: 11; male holotype from Jasper Ridge Biological Preserve, W of Stanford University, Palo Alto, Santa Clara Co., California, 37.405240 -122.242100^4^, elev. 378ft., 13.xi.1921, coll. C.D. Duncan; deposited in AMNH. [examined]
Rhechostica brunnius Raven, 1985: 149.
Aphonopelma brunnium Smith, 1995: 79. **syn. n.**
Aphonopelma chamberlini Smith, 1995: 86; female holotype from Camp Roberts, outside of Paso Robles, Co., California, 35.790969 -120.743364^5^, elev. 642ft., no collecting date, coll. Rupert Hazen; deposited in BMNH. [examined] **syn. n.**
Aphonopelma iviei Smith, 1995: 115; male holotype from Death Valley, Inyo Co., California, 36.735992 -116.970188^6^, elev. 2761ft., 30.ix.1883, coll. Chalmers-Hunt; deposited in BMNH. [examined] **syn. n.**
Aphonopelma lithodomum Chamberlin, 1940: 14; male holotype from House Rock, Coconino Co., Arizona, 36.703978 -111.947171^5^, elev. 5168ft., 9.ix.1939, coll. D. and S. Mulaik; deposited in AMNH. [examined]
Rhechostica lithodomum Raven, 1985: 149.
Aphonopelma lithodomum Smith, 1995: 119. **syn. n.**
Aphonopelma smithi Smith, 1995: 145; male holotype from Frank Raines County Park, off Del Puerto Canyon Rd, ~20km W of Patterson, Stanislaus Co., California, 37.422734 -121.380697^5^, elev. 1153ft., coll. Russell Smith, x.1992; deposited in BMNH. Two male paratypes from Patterson, Stanislaus Co., California, 37.471600 -121.129656^5^, elev. 107ft., x.1992, coll. Russell Smith; deposited in BMNH. [examined] **syn. n.**
Aphonopelma zionis Chamberlin, 1940: 24; male holotype from Zion National Park, near entrance, Washington Co., Utah, 37.205521 -112.983664^4^, elev. 3973ft., 16.viii.1925, coll. Dr. A.M. Woodbury; deposited in AMNH. [examined]
Rhechostica zionis Raven, 1985: 149.
Aphonopelma zionis Smith, 1995: 157. **syn. n.**

#### Diagnosis.

*Aphonopelma iodius* (Fig. 61) is a member of the *iodius* species group and can be identified by a combination of morphological, molecular, and geographic characteristics. Nuclear DNA identifies *Aphonopelma iodius* as a strongly supported lineage that is the sister lineage to *Aphonopelma eutylenum*, and paraphyletic with regards to the incipient species lineage *Aphonopelma johnnycashi* sp. n. (Fig. 8). There are no pronounced measurements or characters that help discriminate male or female *Aphonopelma iodius* from closely related phylogenetic species in the *iodius* species group, except for *Aphonopelma johnnycashi* sp. n., which is geographically and morphologically distinct. Males and females of *Aphonopelma iodius* can easily be differentiated from *Aphonopelma steindachneri* by their lighter color and greater extent of scopulation on metatarsi III and IV, and from syntopic members of the *paloma* species group (*Aphonopelma atomicum* sp. n., *Aphonopelma icenoglei* sp. n., *Aphonopelma joshua*, *Aphonopelma mojave*, and *Aphonopelma prenticei* sp. n.) by their larger size. Male *Aphonopelma iodius* possess a larger L3 scopulation extent (78%–96%) than *Aphonopelma steindachneri* (40%–54%), and a larger L4 scopulation extent (62%-88%) than *Aphonopelma steindachneri* (21%–31%). Female *Aphonopelma iodius* possess a larger L3 scopulation extent (72%–95%) than *Aphonopelma steindachneri* (51%–61%), and a larger L4 scopulation extent (59%–83%) than *Aphonopelma steindachneri* (24%–34%).

#### Description.

Originally described by Chamberlin and Ivie (1939).

#### Redescription of male exemplar

(APH_2015; Fig. 62). *Specimen preparation and condition*: Specimen collected live crossing road, preserved in 80% ethanol; deposited in AUMNH; original coloration faded due to preservation. Left legs I, III, IV, and left pedipalp removed for measurements and photographs; stored in vial with specimen. Right leg III removed for DNA and stored at -80°C in the AUMNH (Auburn, AL). *General coloration*: Black and faded brown. *Cephalothorax*: Carapace 16.14 mm long, 15.02 mm wide; Hirsute; densely clothed with light brown iridescent pubescence mostly appressed to surface; fringe covered in long setae not closely appressed to surface; foveal groove medium deep and straight; pars cephalica region rises gradually from foveal groove, gently arching anteriorly toward ocular area; AER slightly procurved, PER very slightly recurved; normal sized chelicerae; clypeus slightly extends forward on a curve; LBl 2.04, LBw 2.56; sternum hirsute, clothed with black, densely packed setae. *Abdomen*: Densely clothed in short black/brown pubescence with numerous longer red/orange setae interspersed; possessing a dense dorsal patch of black Type I urticating bristles (Cooke et al. 1972). *Legs*: Hirsute, particularly ventrally; densely clothed in a mix of black or faded black pubescence, femurs are darker. Metatarsus I slightly curved. F1 16.56; F1w 4.02; P1 6.57; T1 14.65; M1 13.29; A1 8.96; F3 13.95; F3w 4.46; P3 5.58; T3 11.42; M3 13.91; A3 8.33; F4 16.27; F4w 3.86; P4 5.79; T4 13.82; M4 17.32; A4 9.16; femur III is slightly swollen. All tarsi fully scopulate. Extent of metatarsal scopulation: leg III (SC3) = 93.1%; leg IV (SC4) = 72.1%. One ventral spinose seta on metatarsus III; six ventral spinose setae on metatarsus IV; two prolateral spinose setae on tibia I. *Coxa I*: Prolateral surface a mix of fine, hair-like and tapered setae. *Pedipalps*: Hirsute; densely clothed in the same setal color as the other legs, with numerous longer ventral setae; one spinose seta on the apical, prolateral femur; two spinose setae on the prolateral patella; four spinose setae on the prolateral tibia; PTl 9.033, PTw 2.97. When extended, embolus tapers and gently curves to the retrolateral side near apex; embolus very slender, no keels.

**Variation (23).** Cl 9.69–17.75 (13.959±0.45), Cw 8.51–16.49 (13.019±0.43), LBl 1.21–2.23 (1.734±0.06), LBw 1.397–2.56 (2.079±0.06), F1 10.336–16.69 (14.442±0.38), F1w 2.13–4.38 (3.326±0.12), P1 3.916–6.92 (5.704±0.16), T1 9.343–14.65 (12.294±0.28), M1 7.918–14.62 (11.76±0.34), A1 5.204–9.03 (7.508±0.21), L1 length 36.717–60.79 (51.709±1.33), F3 8.88–14.81 (12.472±0.34), F3w 2.27–4.51 (3.525±0.12), P3 3.248–6.39 (4.935±0.17), T3 6.934–11.84 (9.943±0.28), M3 8.446–14.28 (12.058±0.35), A3 5.067–8.63 (7.204±0.21), L3 length 32.69–54.63 (46.665±1.35), F4 10.308–17.21 (14.341±0.39), F4w 2.127–4.27 (3.258±0.12), P4 3.614–6.59 (5.277±0.17), T4 8.674–14.44 (12.274±0.31), M4 10.951–18.26 (15.385±0.4), A4 5.39–9.58 (7.96±0.24), L4 length 39.514–63.97 (55.346±1.51), PTl 6.198–10.293 (8.387±0.22), PTw 2.04–3.30 (2.687±0.07), SC3 ratio 0.789–0.961 (0.896±0.01), SC4 ratio 0.623–0.876 (0.746±0.01), Coxa I setae = thin tapered/tapered, F3 condition = normal/slightly swollen.

#### Description of female exemplar

(APH_2016; Figs 63–66). *Specimen preparation and condition*: Specimen collected live from burrow, preserved in 80% ethanol; deposited in AUMNH; original coloration faded due to preservation. Left legs I, III, IV, and pedipalp removed for photographs and measurements; stored in vial with specimen. Right leg III removed for DNA and stored at -80°C in the AUMNH (Auburn, AL). Genital plate with spermathecae removed and cleared, stored in vial with specimen. *General coloration*: Faded brown/black. *Cephalothorax*: Carapace 18.71 mm long, 17.01 mm wide; Hirsute, densely clothed with light brown pubescence closely appressed to surface; fringe densely covered in longer setae; foveal groove medium deep and straight; pars cephalica region gently rises from thoracic furrow, arching anteriorly toward ocular area; AER slightly procurved, PER very slightly recurved; large chelicerae, clypeus extends forward on a curve; LBl 2.48, LBw 3.25; sternum very hirsute, clothed with dark brown setae. *Abdomen*: Densely clothed dorsally in short black setae with numerous longer, lighter setae interspersed (generally red or orange *in situ*); dense dorsal patch of black Type I urticating bristles (Cooke et al. 1972); ventral side with shorter black/dark brown setae. *Spermathecae*: Paired and separate with wide bases, tapering and curving medially towards capitate bulbs. *Legs*: Very hirsute, particularly ventrally; densely clothed in medium and long brown pubescence, femurs darker. F1 15.37; F1w 4.45; P1 6.35; T1 11.57; M1 9.73; A1 7.40; F3 12.78; F3w 3.84; P3 5.79; T3 9.39; M3 9.85; A3 7.72; F4 15.23; F4w 4.28; P4 6.54; T4 11.62; M4 12.71; A4 8.91. All tarsi fully scopulate. Extent of metatarsal scopulation: leg III (SC3) = 87.8%; leg IV (SC4) = 81.4%. Three ventral spinose setae on metatarsus III; five ventral spinose setae on metatarsus IV. *Coxa I*: Prolateral surface a mix of fine, hair-like and medium tapered setae. *Pedipalps*: Densely clothed in the same setal color as the other legs; two spinose setae on the apical, prolateral femur, one spinose seta on the prolateral patella, and three spinose setae on the prolateral tibia.

**Variation (19).** Cl 8.657–21.4 (15.619±0.68), Cw 7.51–18.6 (14.079±0.6), LBl 1.322–2.73 (2.086±0.09), LBw 1.578–3.25 (2.414±0.11), F1 7.493–16.71 (12.928±0.54), F1w 2.162–4.72 (3.69±0.16), P1 3.155–7.60 (5.581±0.26), T1 6.154–13.02 (10.428±0.41), M1 4.613–11.89 (8.701±0.43), A1 3.861–7.94 (6.577±0.24), L1 length 25.276–56.01 (44.214±1.84), F3 6.452–13.52 (11.003±0.42), F3w 1.923–4.02 (3.281±0.13), P3 2.599–6.65 (4.898±0.22), T3 4.381–10.07 (8.138±0.32), M3 4.766–11.50 (8.808±0.36), A3 3.978–7.72 (6.494±0.22), L3 length 22.176–48.63 (39.341±1.49), F4 7.32–17.23 (13.256±0.53), F4w 1.86–4.28 (3.405±0.14), P4 3.187–6.67 (5.289±0.21), T4 6.243–12.71 (10.466±0.39), M4 7.325–15.1 (12.038±0.43), A4 4.46–8.91 (7.111±0.22), L4 length 28.535–58.40 (48.161±1.72), SC3 ratio 0.724–0.949 (0.855±0.02), SC4 ratio 0.593–0.829 (0.739±0.02), Coxa I setae = tapered. Spermathecae variation can be seen in Figures 64–66.

#### Material examined.

**United States: Arizona: Coconino**: House Rock - off 89A, 36.729414 -112.037386 ^5^, 5337ft., [AUMS_3299, 3/7/89, 1♂, T.R. Prentice, AUMNH]; [AUMS_2386, 2/7/89, 1♂, T.R. Prentice, AUMNH]; off US-89A, Soap Creek Rd, 36.72439 -111.7556 ^1^, 4211ft., [APH_0303, 8/10/07, 1 juv, Zach Valois, AUMNH]; **Mohave**: Road to Summit Springs, .25 miles from the UT/AZ border, 36.99566 -113.917793 ^4^, 2533ft., [APH_2216, 19/10/1993, 1♂, T.R. Prentice, AMNH]; Beaver Dam Mountains, 0.3 miles S of UT/AZ border, 36.991946 -113.93678 ^4^, 2450ft., [AUMS_3286, 6/10/93, 1♂, T.R. Prentice, AUMNH]; Beaver Dam Mountains, Rd to Summit Springs, Old Hwy 91, 36.985176 -113.920017 ^5^, 2398ft., [APH_2218, 20/10/1993, 1♂, T.R. Prentice, AMNH]; Beaver Dam Mountains, Rd to Summit Springs, Old How 91, .2 miles south of UT/AZ line, 36.996269 -113.918282 ^5^, 2520ft., [APH_2258, 6/10/93, 1♂, T.R. Prentice, AMNH]; **California: Alameda**: 5 miles SE of Livermore at jct. of Mines Rd. and Del Valle Rd., 37.62253 -121.7028 ^4^, 784ft., [APH_0180-0182, 5/9/07, 1♀, 2♂, Gilbert Quintana, AUMNH]; Berkeley, 37.871593 -122.272747 ^5^, 164ft., [APH_2459, 24/9/1935, 1♂, unknown, AMNH]; [APH_2460, 15/4/1936, 1♀, W.J. Baerg, AMNH]; Mines Rd, SE of Livermore, 37.5896 -121.6233 ^4^, 1860ft., [APH_0404, 10/2008, 1♂, Mike Dame, AUMNH]; open area just SW jct Del Valle Rd and Mendenhall Rd [on trail along side of hill]; 37.604004 -121.686283 ^4^, 1396ft., [APH_0104-0106, 6/5/07, 3♀, Mike Dame, AUMNH]; **Contra Costa**: Mt. Diablo State Park, 37.920733 -121.941483 ^1^, 589ft., [APH_0985-0986, 9/2009, 1♀, 1♂, Kyle Dickerson, AUMNH]; [APH_0988-0989, 10/2009, 1♀, 1♂, Cody Will, AUMNH]; [APH_2150, 10/1966, 3♀, Pat Warner, AMNH]; [APH_2151, 10/1967, 1♂, Pat Warner, AMNH]; Mt. Diablo State Park, 1.35 miles E Mt Diablo Scenic Blvd on Summit Rd, 37.868743 -121.923839 ^2^, 2640ft., [APH_0102-0103, 15/4/2007, 2♀, Mike Dame, AUMNH]; S of Antioch/Brentwood, Camino Diablo Rd and Vasco Rd, 37.865167 -121.668233 ^1^, 114ft., [APH_2031, 10/2010, 1♂, Mike Marciano, AUMNH]; **Fresno**: Coalinga, 36.09221 -120.42766 ^5^, 904ft., [APH_0307-0308, 11/9/07, 2♀, Gilbert Quintana, AUMNH]; **Imperial**: E of 78/N of Picacho State Recreation Area, on Black Mountain Rd, 33.08765 -114.85645 ^1^, 1564ft., [APH_2028, 9/10/10, 1♂, Peter Scott, AUMNH]; E of 78/N of Picacho State Recreation Area, on Black Mountain Rd, S of 1128, 33.06294 -114.83999 ^1^, 1849ft., [APH_2029, 9/10/10, 1♂, Peter Scott, AUMNH]; Hwy 78, 2/10 mile E of jct with Ogilby Road (S34); 33.088327 -114.907025 ^1^, 1025ft., [APH_3212, 15/11/2013, 1♂, W. Icenogle, AUMNH]; **Inyo**: along Waucoba Rd, 37.14549 -118.13074 ^2^, 6034ft., [APH_1242-1243, 15/10/2010, 2♂, Anette Pillau, AUMNH]; Death Valley National Park, Daylight Pass Rd, near mile 11 marker, Amargosa Mtns, 36.75975 -116.9308 ^1^, 3660ft., [APH_1080, 3/10/10, 1♂, Chris A. Hamilton, AUMNH]; [APH_1083, 4/10/10, 1♀, Chris A. Hamilton, AUMNH]; Death Valley National Park, Emigrant Canyon Rd, Panamint Mtns, 36.42383 -117.19048 ^1^, 4064ft., [APH_1078-1079, 3/10/10, 2♂, Chris A. Hamilton, AUMNH]; [APH_1081-1082, 4/10/10, 2♀, Chris A. Hamilton, AUMNH]; Death Valley National Park, Mesquite Spring Campground Rd, Amargosa Mtns, 36.97597 -117.36693 ^1^, 1930ft., [APH_1084, 4/10/10, 1♂, Chris A. Hamilton, AUMNH]; Deep Springs Valley, E of summit of Westgard Pass, 37.331895 -118.037264 ^5^, 5110ft., [AUMS_2351, 16/10/1976, 1♂, Frank Hovore, AUMNH]; Westgard Pass Road, 2.3 miles E of Big Pine, 37.204734 -118.242688 ^4^, 4318ft., [AUMS_2450, 20/10/1993, 1♂, T.R. Prentice, AUMNH]; **Kern**: 1 mile S of Maricopa, 35.051693 -119.410088 ^5^, 1000ft., [AUMS_2370, 7/12/69, 1♂, E.K. Slope, AUMNH]; 12 miles WNW McKittrick, Temblor Range, 35.34526 -119.80899 ^4^, 3258ft., [APH_0045, 16/4/2000, 1 juv, James Pitts, AUMNH]; outside Rosamond, in hills N or Tropico; off Mojave-Tropico Rd, 34.88133 -118.23144 ^1^, 2637ft., [APH_3103, 19/7/2012, 1♂, Chris Hamilton, Amy Skibiel, AUMNH]; Red Mountain, 20 Mule Team Rd., 9.5 miles E of Cal City, 35.194924 -117.774276 ^5^, 2867ft., [AUMS_2380, 20/10/1994, 1♂, unknown, AUMNH]; 7.6 miles east of California City on 20 Mule Team Parkway, 35.161453 -117.863179 ^4^, 2539ft., [APH_2223, 20/10/1994, 1♂, Thomas R. Prentice, AMNH]; **Lassen**: 0.5 miles S of Hwy 395, N of Honey Lake, where Smoke Creek Ranch Rd. joins Hwy 395, 40.425413 -120.279483 ^4^, 4539ft., [AUMS_2672, 9/9/00, 1♀, Stephan Wiley, AUMNH]; **Los Angeles**: Lang, 34.44549 -118.37372 ^5^, 1862ft., [APH_0592, 13/6/2009, 1 juv, Anette Pillau, AUMNH]; E of Hi Vista, Avenue G, 34.734973 -117.765229 ^5^, 3028ft., [APH_2720, 10/11/1963, 1♂, Geo. R. Wilson, AMNH]; **Mono**: Benton, 37.819199 -118.474701 ^5^, 5410ft., [APH_2676, 27/9/1941, 1♂, L.A. Hart, AMNH]; [APH_2679, 7/10/43, 1♂, W.M. Pearce, AMNH]; **Monterey**: Arroyo Seco, foothills of Santa Lucia Range, 36.238772 -121.480529 ^5^, 977ft., [AUMS_2489, 6/11/87, 1♀, T.R. Prentice, AUMNH]; Carmel, 36.555239 -121.923288 ^5^, 233ft., [APH_2815, 6/1/54, 1♂, unknown, AMNH]; Carmel Valley, 36.479925 -121.7328 ^5^, 410ft., [APH_2713, 11/1944, 1♂, Fred E. Samson, AMNH]; [APH_2718, 6/10/53, 1♂, unknown, AMNH]; dry valley 14 miles southeast of Monterey, 36.471026 -121.702876 ^5^, 850ft., [APH_2471, unknown, 1♂, E. Ricketts, AMNH]; Hastings Natural History Reservation, Robles del Rio, 36.367998 -121.563616 ^5^, 1542ft., [APH_2698, unknown, 1♂, Linsdale, AMNH]; [APH_2714, unknown, 1♂, Linsdale, AMNH]; [APH_2721, unknown, 1♂, Linsdale, AMNH]; Los Laureles Grade Road, southeast of Monterey, 36.518052 -121.756652 ^5^, 768ft., [APH_2696, 8/10/66, 1♂, P. Warner, AMNH]; Santa Lucia Range - Los Padres NF, Arroyo Seco (Campground); 36.231711 -121.485187 ^5^, 944ft., [AUMS_3306, 6/11/87, 1♂, T.R. Prentice, AUMNH]; Lake Nacimiento, along Nacimiento Lake Dr. (Co. Hwy G14); west of Camp Roberts, W of San Miguel, 35.69067 -120.79972 ^1^, 1007ft., [APH_2041, 7/12/11, 1 juv, Chris A. Hamilton, Jason Bond, AUMNH]; Lake Nacimiento, on Vista Rd at junction with Nacimiento Lake Dr. (Co. Road 19); west of Camp Roberts, W of San Miguel, 35.79395 -120.8727 ^1^, 646ft., [APH_2042, 7/12/11, 1♀, Chris A. Hamilton, Jason Bond, AUMNH]; **Riverside**: 3 miles W of I-10 off Hwy 177, 33.705262 -115.451685 ^5^, 1110ft., [AUMS_3326, 8/10/89, 1♀, T.R. Prentice, AUMNH]; Hexie Mountains, 33.830162 -115.983601 ^6^, 3812ft., [APH_2220, 16/4/1989, 1♀, T.R. Prentice, AMNH]; Joshua Tree National Monument, Pinto Basin, 33.925009 -115.700823 ^5^, 1538ft., [AUMS_2371, 10/1962, 1♂, Van Hoose and Rainey, AUMNH]; Joshua Tree National Monument, west of Hidden Valley picnic area, 34.010251 -116.173413 ^5^, 4163ft., [APH_2228, 5/8/87, 1♀, T.R. Prentice, AMNH]; Joshua Tree National Park, Cottonwood Springs area, 33.74139 -115.811471 ^1^, 3112ft., [APH_1500-1501, 5/9/12, 2♂, Brent E. Hendrixson, AUMNH]; [APH_2235, 25/2/1985, 1♂, T.R. Prentice, AMNH]; [APH_2240, 4/4/89, 1♀, T.R. Prentice, AMNH]; Joshua Tree National Park, first roadside pullout stop from west entrance along Quail Springs Rd, along trail, 34.07156 -116.23954 ^4^, 4040ft., [APH_0332-0334, 8/5/08, 3 juv, Brent E. Hendrixson, Zach Valois, AUMNH]; Joshua Tree National Park, near Cottonwood Campground, 33.740358 -115.812362 ^1^, 3046ft., [APH_1327, 31/7/2011, 1♀, Brent E. Hendrixson, Brendon Barnes, Nate Davis, Jake Storms, AUMNH]; Joshua Tree National Park, off El Dorado Mine Rd/Cottonwood Springs Rd, 1/2 mile N of Porcupine Wash, 33.84946 -115.78205 ^1^, 2386ft., [APH_1008, 10/5/10, 1♀, Chris A. Hamilton, AUMNH]; Joshua Tree National Park, on service rd to maintenance area, off El Dorado Mine Rd, 34.02212 -116.01233 ^1^, 3624ft., [APH_1005-1006, 10/5/10, 2♀, Chris A. Hamilton, AUMNH]; Joshua Tree National Park, picnic area, Covington Flats area, 34.027454 -116.30194 ^1^, 4656ft., [APH_0491, 16/5/2009, 1 juv, Brent E. Hendrixson, Bernadette DeRussy, Sloan Click, Jason Bond, AUMNH]; [APH_1198, 29/7/2010, 1♂, Brent E. Hendrixson, Brendon Barnes, Nate Davis, AUMNH]; Joshua Tree National Park, S border, 3.4 miles E of Cottonwood Springs Rd, 33.66753 -115.726563 ^4^, 1750ft., [AUMS_2684, 23/8/1993, 1♀, T.R. Prentice, AUMNH]; near Red Cloud Mine, 33.621745 -115.463815 ^6^, 2068ft., [AUMS_2369, 25/10/1963, 1♂, unknown, AUMNH]; Orocopia Mtns, Joshua Tree National Monument exit, 33.613298 -115.853258 ^5^, 2108ft., [AUMS_3288, 16/12/1989, 1♂, T.R. Prentice, AUMNH]; Pleasant Valley, Joshua Tree National Park, 33.914434 -116.052056 ^5^, 3292ft., [AUMS_2683, 27/8/1966, 1♂, unknown, AUMNH]; Quail Springs Rd (National Park Drive); 3 miles NW of picnic area, 2.7 miles SE of monument entrance, Joshua Tree National Park, 34.068483 -116.235733 ^4^, 3976ft., [APH_2247, 3/8/89, 1♂, T.R. Prentice, AMNH]; West Palm Springs Village, Cottonwood Rd, 1.4 miles N of I-10, San Bernardino Mtns, 33.938417 -116.697217 ^1^, 1621ft., [APH_3136, 20/9/2013, 1♂, W. Icenogle, AUMNH]; Whitewater Canyon Rd area NW of Palm Springs, 33.965967 -116.651917 ^1^, 1896ft., [APH_2036-2037, 7/27/11, 1♂, 1♀, Warren Burke, AUMNH]; Whitewater Canyon Rd, 3 miles N of junction with I-10, San Bernardino Mtns, 33.96385 -116.65105 ^1^, 1878ft., [APH_3135, 8/9/13, 1♂, W. Icenogle, AUMNH]; **San Benito**: E of Hollister, off Quien Sabe Rd, E of Tres Pinos, 36.80288 -121.30323 ^1^, 805ft., [APH_2001-2003, 10/10/10, 3♂, Chris A. Hamilton, RJ Adams, AUMNH]; Hwy 25, 1.5 miles N of Pinnacles turn off, old Horace Bacon Ranch, 36.535996 -121.148339 ^5^, 1309ft., [AUMS_2495, 8/11/87, 1♀, T.R. Prentice, AUMNH]; just E of Hwy-25, vicinity of Pinnacles National Monument, 36.49367 -121.06376 ^2^, 1842ft., [APH_0151, unknown, 1 juv, T.R. Prentice, AUMNH]; Pinnacles N. Mon., .25 miles S of Hwy 25, 36.517091 -121.13235 ^4^, 1197ft., [AUMS_2500, 7/11/87, 1♀, T.R. Prentice, AUMNH]; Pinnacles N. Mon., 8 miles N of turnoff from Hwy 25, 36.48708 -121.21392 ^4^, 1458ft., [AUMS_2508, 7/11/87, 2♂, T.R. Prentice, AUMNH]; Pinnacles, old Horace Bacon Ranch, 1.5 miles NE of Pinnacles, 36.535699 -121.148194 ^5^, 1310ft., [AUMS_2494, 8/11/87, 1♀, T.R. Prentice, AUMNH]; **San Bernardino**: 10 miles south of Barstow on Hwy 247, 34.754995 -117.007841 ^5^, 2877ft., [APH_2252, 7/11/91, 1♂, T.R. Prentice, AMNH]; Coxcomb Mountains, 8 miles west of Hwy 62, 33.990731 -115.430123 ^5^, 3402ft., [APH_2230, 4/2/90, 1♀, T.R. Prentice, AMNH]; NE slope of Coxcomb Mountains, 8 miles west of Hwy 62, 34.048671 -115.335004 ^5^, 1614ft., [APH_2227, 4/2/90, 1♀, T.R. Prentice, AMNH]; Reche Rd, 4.5 miles east of Landers, 34.265929 -116.304316 ^4^, 2730ft., [APH_2242, 18/10/1981, 1♂, W. Icenogle, AMNH]; 1.1 miles NE Lucerne Valley Cutoff along Hwy-247 (Barstow Rd); 34.614513 -116.968992 ^1^, 3239ft., [APH_0758-0759, 5/10/09, 2 juv, Brent E. Hendrixson, Thomas Martin, AUMNH]; 11 miles north of Buelton, 34.677234 -120.157748 ^5^, 745ft., [APH_2701, 23/9/1965, 1♂, Jean and Wilton Ivie, AMNH]; 2.6 miles S Phelan Rd on Baldy Mesa Rd, 34.389973 -117.454903 ^1^, 3974ft., [APH_1575-1576, 6/11/12, 2 juv, Brent E. Hendrixson, AUMNH]; 3-4 miles E of Milpas Dr. Hwy, 18 miles S of Hwy by power lines, 34.525984 -116.984374 ^5^, 3028ft., [AUMS_2521, 28/10/1989, 1♂, T.R. Prentice, AUMNH]; 36 miles E of Joshua Tree National Monument headquarters, 34.124371 -115.408958 ^5^, 1548ft., [AUMS_2540, 12/11/89, 1♂, T.R. Prentice, AUMNH]; along Hwy-62, west of Coxcomb Mountains, 34.113767 -115.496503 ^1^, 2252ft., [APH_1582-1583, 7/11/12, 1♂, 1 juv, Brent E. Hendrixson, AUMNH]; Apple Valley, 34.500666 -117.172078 ^5^, 2943ft., [AUMS_3312, 07/1973, 1♂, R. Estrada, AUMNH]; Coxcomb Mountains, 0.3 miles N of JTNM Boundary, 34.002853 -115.426675 ^4^, 1892ft., [AUMS_2537, 24/11/1989, 1♂, T.R. Prentice, AUMNH]; E of Twentynine Palms, off Hwy 62 - N side of road, near Joshua Tree National Park, 34.11838 -115.89557 ^1^, 1660ft., [APH_3157-3160, 10/11/13, 2♂, 2 juv, Chris A. Hamilton, Brent E. Hendrixson, Molly Taylor, AUMNH]; Hesperia, 2.5 miles S Phelan Rd on Baldy Mesa Rd, 34.389771 -117.453406 ^1^, 3692ft., [APH_1326, 31/7/2011, 1 juv, Brent E. Hendrixson, Brendon Barnes, Nate Davis, Jake Storms, AUMNH]; Honda Road, 34.111948 -116.461021 ^6^, 3403ft., [AUMS_2524, 14/4/1991, 1♀, T.R. Prentice, AUMNH]; Joshua Tree National Monument, W of small picnic area, Quail Mtn. area, 33.97058 -116.299793 ^5^, 3977ft., [AUMS_2467, 10/8/89, 1♂, T.R. Prentice, AUMNH]; Lucerne Valley, Hwy 18 - 3 miles SE Hwy 247, High Desert, 34.423672 -116.918691 ^5^, 3077ft., [AUMS_2381, 1/11/89, 1♂, T.R. Prentice, AUMNH]; off Hwy 247, N of Lucerne Valley, 34.53291 -116.9397 ^1^, 2850ft., [APH_3145, 10/11/13, 1♀, Chris A. Hamilton, Brent E. Hendrixson, Molly Taylor, AUMNH]; 3.6 miles W of Nipton on Nipton Rd., 35.45746 -115.3346 ^1^, 2665ft., [APH_0318, 6/5/08, 1 juv, Brent E. Hendrixson, Zach Valois, Stephen Rash, AUMNH]; Halloran Summit Road, 0.3 miles N I-15, 35.407038 -115.795374 ^1^, 4079ft., [APH_0515, 21/5/2009, 1♀, Brent E. Hendrixson, Bernadette DeRussy, Sloan Click, AUMNH]; Kingston Range, Excelsior Mine Rd, 35.71268 -115.79702 ^2^, 3601ft., [APH_1642, 26/11/2012, 1 juv, Matt Graham, AUMNH]; 100 yards N Havasu Lake Road, 2 miles SE of jct with Hwy 95, 34.532801 -114.624968 ^1^, 1670ft., [APH_3201, 11/11/13, 1♀, W. Icenogle, AUMNH]; Eastern slope of Whipple Mountains, Black Landing road across from Gene Wash reservoir, 34.346101 -114.205737 ^5^, 588ft., [AUMS_3310, 4/2/90, 1♀, T.R. Prentice, AUMNH]; Hwy 95, ~8 miles N of jct with Havasu lake Road, S of Lobecks Pass, 34.672525 -114.625747 ^1^, 1578ft., [APH_3202, 16/11/2013, 1♂, W. Icenogle, AUMNH]; near Needles, on River Road, 2.5 miles S of I-40, 34.794722 -114.608835 ^5^, 725ft., [AUMS_2349, 22/10/1976, 1♂, W. Icenogle, AUMNH]; off Hwy 62, W of Parker (Whipple Mtns); 34.2131 -114.42914 ^1^, 987ft., [APH_1004, 9/5/10, 1♀, Chris A. Hamilton, AUMNH]; 3.1 miles east of Kramer Jct on Hwy 56, 34.983798 -117.484214 ^4^, 2414ft., [APH_2250, 8/11/92, 1♂, Thomas R. Prentice, AMNH]; East Mojave Desert, Mid Hills near Providence Mountain , 35.22291 -115.457469 ^6^, 4429ft., [APH_2222, 13/5/1989, 1♂, Thomas R. Prentice, AMNH]; Kelso-Cina Rd, 11.5 miles north of Kelso, 35.135929 -115.530791 ^4^, 3382ft., [APH_2236, 1/11/92, 1♂, Thomas R. Prentice, AMNH]; Kelso-Cina Rd, 6.3 miles north of Kelso, 35.083174 -115.567666 ^4^, 2926ft., [APH_2251, 1/11/92, 1♂, Thomas R. Prentice, AMNH]; Red Mountain, 1 mile west of Powerline Rd. , 35.357912 -117.623138 ^5^, 3664ft., [APH_2233, 26/10/1991, 1♂, Thomas R. Prentice, AMNH]; [APH_2234, 13/10/1992, 1♂, Thomas R. Prentice, AMNH]; Red Mountain, 1.5 miles west of Hwy 395, 35.358458 -117.640062 ^4^, 3937ft., [APH_2237, 20/10/1991, 1♂, Thomas R. Prentice, AMNH]; Red Mountain, 1.5 miles west of Powerline rd., 35.352194 -117.606607 ^4^, 3802ft., [APH_2221, 13/10/1991, 1♀, Thomas R. Prentice, AMNH]; Red Mountain, 19 miles north of Kramer Junction, 1.5 miles west of Hwy 395, 35.361421 -117.621001 ^5^, 3694ft., [APH_2255, 13/10/1992, 1♀, Thomas R. Prentice, AMNH]; Red Mountain, 19 miles north of Kramer Junction, off Hwy 395, 35.353665 -117.617923 ^5^, 3589ft., [APH_2238, 14/10/1991, 1♀, Thomas R. Prentice, AMNH]; [APH_2239, 12/10/1991, 1♀, Thomas R. Prentice, AMNH]; [APH_2241, 26/10/1991, 1♂, Thomas R. Prentice, AMNH]; [APH_2246, 20/10/1991, 1♂, Thomas R. Prentice, AMNH]; Red Mountain, 20 miles north of Kramer Jct, 1.5 miles west of Hwy 395, 35.363666 -117.623444 ^5^, 3694ft., [APH_2256, 22/1/1994, 1♀, Thomas R. Prentice, AMNH]; Red Mountain, 20 miles north of Kramer Jct, on Power Rd, 35.361066 -117.615155 ^5^, 3602ft., [APH_2243, 26/10/1991, 1♂, Thomas R. Prentice, AMNH]; Red Mountain, off Hwy 395, 35.36059 -117.615889 ^6^, 3664ft., [APH_2231, 14/10/1991, 1♀, Thomas R. Prentice, AMNH]; East Mojave, Black Canyon Rd., 2 miles N of Essex Rd. jct., 34.937755 -115.413054 ^4^, 3180ft., [AUMS_2372, 25/10/1992, 1♂, Thomas R. Prentice, AUMNH]; Red Mountain, 35.316678 -117.591078 ^6^, 3164ft., [AUMS_2353, 14/10/1991, 1♀, Tom Prentice, AUMNH]; Red Mountain, ~19 miles N of Kramer Junction, 35.271452 -117.630077 ^5^, 3220ft., [AUMS_2373, 3/10/92, 1♀, Thomas R. Prentice, AUMNH]; [AUMS_2375, 13/10/1991, 1♂, Thomas R. Prentice, AUMNH]; Red Mountain, ~19 miles N of Kramer Junction - Hwy 395, power line rd., 35.262031 -117.612309 ^5^, 3059ft., [AUMS_2374, 3/10/92, 1♂, Thomas R. Prentice, AUMNH]; Red Mountain, ~19 miles N of Kramer Junction, 0.4 W of powerline, 35.250499 -117.616993 ^5^, 3000ft., [AUMS_2378, 20/10/1991, 1♂, Thomas R. Prentice, AUMNH]; Red Mountain, 0.6 miles 20 Mule Team Rd., 35.251471 -117.617676 ^4^, 3011ft., [AUMS_2377, 20/10/1991, 1♂, Thomas R. Prentice, AUMNH]; Red Mountain, 1 mile W of power line rd., 35.296474 -117.6267 ^5^, 3230ft., [AUMS_2346, 26/10/1991, 1♂, Thomas R. Prentice, AUMNH]; Red Mountain, 19 miles N of Kramer Junction off Hwy 395, power line rd., 35.266359 -117.612937 ^5^, 3081ft., [AUMS_2343, 17/10/1992, 1♂, Thomas R. Prentice, AUMNH]; Red Mountain, 19 miles N of Kramer Junction with 20 Mule Team Rd, 35.265871 -117.613745 ^5^, 3081ft., [AUMS_2365, 19/10/1994, 1♂, Thomas R. Prentice, AUMNH]; Red Mountain, 3.6 miles W of power line road, on 20 Mule Team Rd., 35.25059 -117.620344 ^4^, 3008ft., [AUMS_2342, 10/10/92, 1♂, Thomas R. Prentice, AUMNH]; Red Mountain, power line rd., mining mounds, 35.329506 -117.611623 ^5^, 3364ft., [AUMS_2341, 20/10/1991, 1♂, Thomas R. Prentice, AUMNH]; 0.4 miles north of Covington Flats entrance, 34.04764 -116.316153 ^4^, 4619ft., [APH_2229, 10/8/89, 1♂, T.R. Prentice, AMNH]; **San Joaquin**: Tracy - Carnegie SVRA, 37.653867 -121.627067 ^1^, 1553ft., [APH_0990, 10/2009, 1♂, Cody Will, AUMNH]; **San Luis Obispo**: 7 miles northeast of Cholame, 35.774731 -120.181254 ^5^, 1693ft., [APH_2671, 31/8/1958, 1♂, V. Roth, AMNH]; 7 miles NW of Paso Robles - Lake Nacimiento, 35.690839 -120.801114 ^5^, 1108ft., [AUMS_2579, 10/1996, 1♂, Andrew Walters, AUMNH]; Chimineas Ranch, 35.17554 -120.01147 ^4^, 2383ft., [APH_0078-0083, 25/3/2007, 1♂, 2♀, 3 juv, Alice Abela, AUMNH]; Cayucos, approx. 4 miles E on Old Creek Road, 35.47548 -120.85584 ^4^, 297ft., [APH_0016, 15/8/2002, 1♂, Kelly C. Carroll, AUMNH]; E of Santa Maria, Hwy 166, 35.01671 -120.33505 ^1^, 722ft., [APH_1092-1094, 9/10/10, 2♂, 1♀, Chris A. Hamilton, AUMNH]; Poly Canyon, California Polytechnic Univ., San Luis Obispo, 35.31598 -120.65632 ^4^, 701ft., [APH_0077, 22/10/2006, 1 juv, Alice Abela, AUMNH]; **San Mateo**: 1000 block of Porto Marino Dr, San Carlos, 37.488576 -122.274238 ^2^, 254ft., [APH_1371, 9/2011, 1♂, Julie Pitre, AUMNH]; Jasper Ridge, 37.40524 -122.2421 ^5^, 417ft., [APH_2477, 13/11/1921, 1♀, 4♂, 1 juv, C.D. Duncan, AMNH]; **Santa Barbara**: 12 miles northeast of Los Olivos near Zaca Park, 34.722266 -120.035831 ^5^, 1296ft., [APH_2709, 29/7/1961, 1♂, Roth and Roth, AMNH]; 5 miles south of Buelton, 34.560664 -120.190635 ^5^, 564ft., [APH_2682, 23/9/1965, 1♂, Jean and Wilton Ivie, AMNH]; Figueroa Mountain Road, 34.72249 -119.96665 ^4^, 4043ft., [APH_0036, 4/9/06, 1♂, Alice Abela, AUMNH]; [APH_0039-0040, 4/9/06, 2 juv, Alice Abela, AUMNH]; NE of Buellton, E on 154 at Hwy 101 split, 34.68066 -120.15461 ^1^, 785ft., [APH_1091, 9/10/10, 1♀, Chris A. Hamilton, AUMNH]; Sycamore Canyon area, along Figueroa Mountain Rd, 34.727447 -120.098169 ^4^, 1160ft., [APH_0418, 20/9/2008, 1♂, Anette Pillau, AUMNH]; **Santa Clara**: 0.37 miles SW jct Mines Rd on San Antonio Valley Rd, 37.4005 -121.4918 ^2^, 2240ft., [APH_0407, 10/2008, 1♀, Mike Dame, AUMNH]; Mount Hamilton Area, 37.37888 -121.81057 ^4^, 526ft., [APH_0051, 24/8/2001, 1 juv, Katrina Couch, Jerami Myers, AUMNH]; Palo Alto, 37.441872 -122.143006 ^5^, 30ft., [APH_2695, Spring 1922, 1♂, R.W. Doane, AMNH]; Stanford, 37.424106 -122.166076 ^5^, 92ft., [APH_2672, 1905, 1♀, unknown, AMNH]; **Stanislaus**: 0.06 miles W jct Adobe Canyon Rd on Del Puerto Canyon Rd, 37.4085 -121.4102 ^2^, 1340ft., [APH_0405, 10/2008, 1♂, Mike Dame, AUMNH]; Del Puerto Canyon Rd, 37.4 -121.4438 ^4^, 2050ft., [APH_0406, 10/2008, 1♂, Mike Dame, AUMNH]; Del Puerto Canyon, 25 miles west of Patterson, 37.42119 -121.374632 ^5^, 1188ft., [APH_2677, 23/9/1967, 1♂, W.J. Turner, AMNH]; Frank Raines Park, 37.419831 -121.370933 ^1^, 1200ft., [APH_2030, 10/2010, 1♀, Mike Dame, AUMNH]; Henry W Coe State Park, 37.18823 -121.54585 ^1^, 2749ft., [APH_1095-1099, 10/10/10, 3♂, 2♀, Chris A. Hamilton, RJ Adams, AUMNH]; [APH_2000, 10/10/10, 1♂, Chris A. Hamilton, RJ Adams, AUMNH]; **Nevada: Clark**: 0.3 miles N Hwy-161 along dirt road, between Jean and Goodsprings, 35.81268 -115.38099 ^1^, 3370ft., [APH_0358-0359, 12/5/08, 2 juv, Brent E. Hendrixson, Zach Valois, AUMNH]; 1.9 miles E Hwy-160 on Trout Canyon Rd, 36.122811 -115.817589 ^1^, 3463ft., [APH_1208-1210, 31/7/2010, 3 juv, Brent E. Hendrixson, Brendon Barnes, Nate Davis, AUMNH]; 10 miles east of Las Vegas, 36.178731 -114.873725 ^5^, 1447ft., [APH_2274, 07/1955, 1♂, W.J. Gertsch, AMNH]; 17-19 miles SE of Pahrump on Hwy 160, 36.029949 -115.702158 ^5^, 3566ft., [APH_2224, 11/10/74, 1♂, W. Icenogle, AMNH]; 4.6 miles W US-95 (Searchlight) on Hwy-164, 35.488073 -114.996602 ^1^, 3690ft., [APH_1220, 2/8/10, 1♀, Brent E. Hendrixson, Brendon Barnes, Nate Davis, AUMNH]; 7.7 miles W Searchlight on Hwy-164, 35.50444 -115.04914 ^1^, 4057ft., [APH_0751, 3/10/09, 1♂, Brent E. Hendrixson, Thomas Martin, AUMNH]; 8.2 miles west of Searchlight, 2 miles north of Hwy 164, 35.496212 -115.213376 ^4^, 4665ft., [APH_2248, 7/10/89, 1♀, T.R. Prentice, AMNH]; 8.2 miles west of Searchlight, on Hwy 164, 35.508562 -115.066862 ^4^, 4249ft., [APH_2253, 7/10/89, 1♂, T.R. Prentice, AMNH]; along dirt road S of Hwy-95, 36.56321 -115.881585 ^1^, 3701ft., [APH_1215, 1/8/10, 1♀, Brent E. Hendrixson, Brendon Barnes, Nate Davis, AUMNH]; Boulder City and Hemenway Wash, 36.020585 -114.80798 ^5^, 1575ft., [APH_2458, 10/1939, 5♂, B.E. Rees, AMNH]; Desert National Wildlife Refuge, open desert N of Mormon Well Rd, approx. 3 miles W Alamo Rd, 36.435992 -115.351558 ^2^, 2968ft., [APH_0476, 13/5/2009, 1 juv, Brent E. Hendrixson, Bernadette DeRussy, Sloan Click, Jason Bond, AUMNH]; Goodsprings, 35.832479 -115.434165 ^5^, 3707ft., [APH_2731, 22/12/1946, 1♂, J.C. Madsen, AMNH]; Indian Springs, 1.5 miles WSW US-95 on Cold Creek Rd (near Southern Desert Correction Center); 36.51153 -115.570812 ^4^, 3454ft., [APH_0183, 12/9/07, 1♂, Tim Filson, AUMNH]; [APH_0184, 8/8/07, 1♀, Tim Filson, AUMNH]; Muddy Mountains, 36.36485 -114.70364 ^2^, 2851ft., [APH_1634, 10/11/12, 1 juv, Matt Graham, AUMNH]; Pahrump Rd, 17 to 19 miles SE of Pahrump, 36.04757 -115.726851 ^5^, 3375ft., [AUMS_2608, 11/10/74, 1♀, W. Icenogle, AUMNH]; SE of Pahrump, Trout Canyon Rd, 36.12301 -115.82007 ^1^, 3441ft., [APH_1075-1077, 2/10/10, 1♂, 2♀, Chris A. Hamilton, AUMNH]; Searchlight, 3 miles south on Hwy 95, 35.418906 -114.907457 ^5^, 3176ft., [APH_2245, 2/10/81, 1♂, W. Icenogle, AMNH]; Christmas Tree Pass, Spirit Mtn, Lake Mead NRA, 35.26086 -114.73926 ^1^, 3887ft., [APH_0313, 12/10/07, 1♀, Rick C. West, AUMNH]; [APH_0517, 24/5/2009, 1♀, Brent E. Hendrixson, Rick C. West, Bernadette DeRussy, AUMNH]; [APH_0996-0998, 6/5/10, 3♀, Chris A. Hamilton, Rick West, AUMNH]; Searchlight, 8.2 miles west on Hwy 164 in the foothills of highlands, 35.49844 -115.056633 ^4^, 4108ft., [APH_2261, 7/10/89, 1♀, T.R. Prentice, AMNH]; **Lincoln**: 8.3 miles S Alamo Canyon Rd on Hwy-93, 37.224912 -115.082582 ^1^, 3160ft., [APH_0782, 9/10/09, 1 juv, Brent E. Hendrixson, Thomas Martin, AUMNH]; Pioche, 37.929818 -114.451689 ^5^, 6063ft., [APH_2728, 10/1941, 1♂, Mrs. Dibble, AMNH]; **Nye**: 0.8 miles S Hwy-95 along Rd-552, 36.582225 -115.944177 ^1^, 3728ft., [APH_1547, 23/10/2012, 1 juv, Brent E. Hendrixson, AUMNH]; Mercury, 36.66051 -115.994475 ^5^, 3796ft., [APH_2724, 1962, 1♂, Gertsch, AMNH]; [APH_2726, 16/10/1961, 1♂, unknown, AMNH]; [APH_2729, 10/11/60, 1♂, Gertsch, AMNH]; [APH_2732, 9/10/61, 1♂, unknown, AMNH]; **White Pine**: Ely, 39.247265 -114.88863 ^5^, 6437ft., [APH_2619, 24/8/1952, 1♂, unknown, AMNH]; **Utah: Beaver**: Wah Wah Springs, 38.503218 -113.472862 ^5^, 5233ft., [APH_2270, 07/1940, 1♂, unknown, AMNH]; unknown, 38.326633 -113.28705 ^7^, 6214ft., [APH_2272, 25/8/1946, 1♂, Chamberlin and Ivie, AMNH]; **Box Elder**: Brigham City, 41.510213 -112.015502 ^6^, 4439ft., [APH_2283, 1927, 2♂, unknown, AMNH]; Honeyville, approx. 2 miles E I-15 at foothills of mtns, 41.63333 -112.04894 ^4^, 5516ft., [APH_0006, 29/8/2004, 1♂, Andrea Strange, AUMNH]; **Cache**: Sardine Canyon, 41.581309 -111.932847 ^5^, 4993ft., [APH_2734, 9/1947, 1♂, unknown, AMNH]; **Davis**: Centerville, 40.918 -111.87216 ^5^, 4393ft., [APH_2269, 3/10/36, 1♂, unknown, AMNH]; **Duchesne**: Lower part of City Creek Canal, 40.202984 -110.413946 ^5^, 5640ft., [APH_2280, 21/9/1942, 1♂, Wilton Ivie, AMNH]; **Millard**: W of Delta on Hwy 6/50, E of House Range Mtns around Antelope Springs Rd and Steamboat Springs Rd, 39.05897 -113.24963 ^1^, 4784ft., [APH_2015, 23/9/2010, 1♂, Kelly Parr, AUMNH]; [APH_2016, 14/10/2010, 1♀, Chris A. Hamilton, AUMNH]; White Valley, 38.378831 -113.706023 ^5^, 5682ft., [APH_2271, 12/8/40, 1♂, Chamberlin and Ivie, AMNH]; **Piute**: Marysvale, 38.44942 -112.2302 ^5^, 5892ft., [APH_2736, 8/8/48, 1♂, Edw. Cormolly, AMNH]; **Salt Lake**: Big Cottonwood Canyon, 0.9 miles E Wasatch Blvd on UT-190, 40.62078 -111.77369 ^1^, 5200ft., [APH_0050, 14/7/2006, 1♀, Brent E. Hendrixson, AUMNH]; Bluffdale, ~3 miles W I-15 on 14600 S, 40.48621 -111.91835 ^4^, 4466ft., [APH_0004, 10/10/04, 1♂, Tiffany Blanchard, AUMNH]; East of Holladay, 40.671407 -111.782393 ^6^, 5650ft., [APH_2267, 25/8/1941, 1♂, Chamberlin and Ivie, AMNH]; Mill Creek Canyon, 40.69001 -111.777132 ^5^, 5240ft., [APH_2738, 08/1928, 1♂, R.V. Chamberlin, AMNH]; Mill Creek Canyon, Wasatch Mountains, 40.690695 -111.773522 ^5^, 5249ft., [APH_2268, 5/8/42, 1♂, Chamberlin and Ivie, AMNH]; [APH_2277, 12/9/45, 1♂, Willis M. Creer, AMNH]; N Salt Lake City , 40.86167 -111.892803 ^6^, 4902ft., [APH_2263, 210/2008, 2♂, unknown, AMNH]; Salt Lake City, 40.727231 -112.039815 ^6^, 4245ft., [APH_2207, unknown, 2♂, unknown, AMNH]; [APH_2265, 22/9/1941, 4♂, Chamberlin and Ivie, AMNH]; [APH_2266, 08/1939, 2♂, unknown, AMNH]; [APH_2273, 10/1933, 1♂, unknown, AMNH]; Salt Lake City, Bonneville Shoreline Trail, 40.763 -111.82 ^2^, 5210ft., [APH_0788-0789, 28/8/2009, 2♂, Will Black, AUMNH]; unknown, 40.644188 -111.952249 ^7^, 4409ft., [APH_2262, 1934, 3♂, unknown, AMNH]; **San Juan**: San Juan Bulge, 1/2 mile below Mexican Hat, 37.147965 -109.866298 ^5^, 4114ft., [APH_2737, 18/7/1947, 1♀, Harold Higgins, AMNH]; **Sevier**: Oak Creek Canyon, 38.526922 -111.85269 ^5^, 6998ft., [APH_2735, 24/8/1968, 1♂, G.F. Knowetn, AMNH]; **Tooele**: S of Delle, off I-80, 40.68329 -112.88853 ^2^, 4920ft., [APH_0790, 17/10/2009, 1♂, DeAnn Neal, AUMNH]; South Mountain, 40.4651111 -112.4826666 ^5^, 5406ft., [APH_0163, 19/8/2007, 1♂, Brian Nielsen, AUMNH]; South Willow Canyon, 40.5093342 -112.5349121 ^5^, 5679ft., [APH_0161, 2/8/07, 1♂, Brian Nielsen, AUMNH]; [APH_0162, 9/8/07, 1♂, Brian Nielsen, AUMNH]; Stansbury Mountains, 40.5097653 -112.5324224 ^5^, 5797ft., [APH_0155, 13/7/2007, 1♂, Brian Nielsen, AUMNH]; Stockton, 40.452722 -112.360782 ^6^, 5121ft., [APH_2264, 1918, 2♂, unknown, AMNH]; Wendover, 40.737095 -114.03751 ^5^, 4295ft., [APH_2733, 15/9/1942, 1♂, Don Nieleson, AMNH]; **Utah**: Box Elder Canyon, 40.473006 -111.750208 ^5^, 5548ft., [APH_2739, 2/10/47, 2♂, S. H. Jenaera, AMNH]; **Washington**: 1 mile N of La Verkin, 37.230736 -113.271966 ^4^, 3245ft., [APH_2282, 12/10/39, 1♂, unknown, AMNH]; 10 miles south of Enterprise, 37.450725 -113.63881 ^5^, 5791ft., [APH_2279, 14/10/1939, 1♂, unknown, AMNH]; Beaver Dam Mountains, 37.0764081 -113.8702196 ^5^, 4026ft., [APH_0154, 13/7/2007, 1♂, Brian Nielsen, AUMNH]; Beaver Dam Mountains, 0.5 miles W, 37.077649 -113.872749 ^6^, 4135ft., [AUMS_2468, 1993, 1♀, T.R. Prentice, AUMNH]; Beaver Dam Mountains, near Bulldog Knolls, 37.0184842 -113.8854744 ^5^, 3086ft., [APH_0157, 13/7/2007, 1♀, Brian Nielsen, AUMNH]; Beaver Dam Mountains, Old Highway-91, 37.05399 -113.894229 ^5^, 3445ft., [APH_0453, 11/10/06, 1♂, Brian Nielsen, AUMNH]; Beaver Dam Mountains, Old Hwy 91, 1.2 miles N of Utah/AZ line, 37.016981 -113.909332 ^4^, 2960ft., [AUMS_2347, 12/10/93, 1♀, T.R. Prentice, AUMNH]; Beaver Dam Mountains, Rd to Summit Springs, Old Hwy 91, 37.028247 -113.90558 ^5^, 3038ft., [APH_2217, 6/10/93, 1♂, T.R. Prentice, AMNH]; [APH_2225, 19/10/1993, 1♂, T.R. Prentice, AMNH]; Beaver Dam Mountains, Rd to Summit Springs, Old Hwy 91, .2 miles north of UT/AZ line, 37.003736 -113.914265 ^5^, 2635ft., [APH_2249, 19/10/1993, 1♂, T.R. Prentice, AMNH]; Beaver Dam Mountains, Rd to Summit Springs, Old Hwy 91, 1.7 miles north of UT/AZ line, 37.018438 -113.908722 ^5^, 2884ft., [APH_2244, 19/10/1993, 1♂, T.R. Prentice, AMNH]; Beaver Dam Mountains, Rd to Summit Springs, Old Hwy 91, 2.5 miles north of UT/AZ line, 37.017035 -113.909593 ^5^, 2520ft., [APH_2259, 12/10/93, 1♂, T.R. Prentice, AMNH]; Beaver Dam Mountains, Rd to Summit Springs, Old Hwy 91, 2.6 miles north of UT/AZ line, 37.036118 -113.902906 ^5^, 3169ft., [APH_2257, 19/10/1993, 1♂, T.R. Prentice, AMNH]; Beaver Dam Mountains, Summit Spring, near Old Hwy-91, 37.088438 -113.871915 ^1^, 4297ft., [APH_0779, 8/10/09, 1 juv, Brent E. Hendrixson, Thomas Martin, AUMNH]; Beaver Dam Mountains, Summit Springs, 37.066177 -113.885856 ^5^, 3865ft., [APH_2254, 4/7/89, 1♂, T.R. Prentice, AMNH]; [APH_2260, 16/9/1989, 1♂, T.R. Prentice, AMNH]; [AUMS_3320, 5/10/95, 1♂, T.R. Prentice, AUMNH]; Enterprise, 37.573587 -113.719133 ^5^, 5322ft., [APH_2284, 20/9/1939, 1♂, Ronn Hardy, AMNH]; Entrance to Zion National Park, 37.200215 -112.990135 ^5^, 4006ft., [APH_2278, 16/8/1925, 1♂, A.M. Woodbury, AMNH]; just outside Zion National Park, Smith Mesa Road, 37.290592 -113.112587 ^5^, 5230ft., [AUMS_2696, 22/8/1991, 1♀, 1♂, T.R. Prentice, AUMNH]; just outside Zion National Park, Smith Mesa Road, 37.289355 -113.114065 ^5^, 5230ft., [AUMS_2698, 23/8/1991, 1♂, T.R. Prentice, AUMNH]; near Beaver Dam Mountains, 37.168672 -113.964461 ^6^, 4852ft., [APH_2214, 10/1931, 1♂, A.M. Woodbury, AMNH]; near Gunlock, 37.2465941 -113.7766128 ^5^, 3664ft., [APH_0153, 13/7/2007, 1♂, Brian Nielsen, AUMNH]; on Smith Mesa Rd., 1.2 miles W of junction with Kolob Reservoir Rd., 37.290556 -113.113463 ^4^, 5225ft., [AUMS_2201, 23/9/1989, 1♂, T.R. Prentice, AUMNH]; Road to Summit Spring, Old Hwy 91, 2 miles north of UT/AZ line, 37.030356 -113.904284 ^4^, 3074ft., [APH_2232, 6/10/93, 1♂, T.R. Prentice, AMNH]; Smith Mesa campground, just outside Zion National Park boundary, 37.241343 -113.202808 ^5^, 5002ft., [AUMS_2332, 22/8/1991, 1♂, T.R. Prentice, AUMNH]; Smith Mesa, .5 miles W of Zion National Park boundary, 37.204674 -112.993735 ^5^, 3899ft., [AUMS_2580, 22/9/1989, 1♂, T.R. Prentice, AUMNH]; St. George, 37.095278 -113.578056 ^6^, 2638ft., [APH_2219, 25/10/1939, 1♂, unknown, AMNH]; Summit Spring, Old Hwy 91, 37.066904 -113.895177 ^5^, 3773ft., [APH_2226, 5/10/93, 1♀, T.R. Prentice, AMNH]; Summit Springs, Beaver Dam Mtns, 37.080788 -113.871741 ^6^, 4140ft., [AUMS_2367, 3/7/89, 1♂, T.R. Prentice, AUMNH]; Welcome Spring Rd, Beaver Dam Mountains, 37.090134 -113.945487 ^5^, 3835ft., [APH_2215, 23/11/1989, 1♂, T.R. Prentice, AMNH]; Zion National Park, 37.208573 -112.982125 ^5^, 3990ft., [APH_2275, unknown, 4♂, A.M. Woodbury, AMNH]; [APH_2281, 1927, 1♂, A.M. Woodbury, AMNH]; [AUMS_3300, unknown, 1♀, T.R. Prentice, AUMNH]; Zion National Park, at Temple Sinawava site/trail, 37.285417 -112.94755 ^1^, 3865ft., [APH_2018, 18/10/2010, 1♀, Chris A. Hamilton, AUMNH]; Zion National Park, field next to Watchman employee housing neighborhood, 37.20527 -112.97655 ^1^, 4004ft., [APH_2019-2020, 18/10/2010, 2♀, Chris A. Hamilton, AUMNH]; Zion National Park, near administrative buildings, 37.20841 -112.98188 ^1^, 4010ft., [APH_2017, 18/10/2010, 1♂, Chris A. Hamilton, AUMNH]; Zion National Park, road to maintenance bldg. and Oak Creek employee housing neighborhood, 37.2099 -112.98579 ^1^, 4031ft., [APH_2021, 18/10/2010, 1♂, Chris A. Hamilton, AUMNH]; [APH_2022, 19/10/2010, 1♂, Chris A. Hamilton, AUMNH]; Zion National Park, Smith-Mesa Rd, 37.290185 -113.113675 ^5^, 5230ft., [AUMS_4194, 17/9/1989, 1♂, T.R. Prentice, AUMNH].

#### Distribution and natural history.

*Aphonopelma iodius* has a wide distribution that stretches across several diverse habitats from the Bay Area of California, south along the Coast Ranges across the Transverse Ranges, into the Mojave Desert (Fig. 1H), and north throughout the Great Basin Desert of Nevada, northwestern Arizona, and Utah (Fig. 67). *Aphonopelma iodius* has been found in the following Level III Ecoregions: Sierra Nevada, Central California Foothills and Coastal Mountains, Central California Valley, Southern California Mountains, Mojave Basin and Range, Sonoran Basin and Range, Central Basin and Range, Arizona/New Mexico Plateau, Northern Basin and Range, Wasatch and Uinta Mountains, and Colorado Plateaus. *Aphonopelma iodius* can be found in syntopy with a number of species across its distribution including *Aphonopelma atomicum* , *Aphonopelma eutylenum*, *Aphonopelma icenoglei* , *Aphonopelma johnnycashi* , *Aphonopelma joshua*, *Aphonopelma mojave*, *Aphonopelma prenticei* , and *Aphonopelma steindachneri*. The breeding season, when mature males abandon their burrows in search of females, occurs from late summer to early fall (generally August–November).

#### Conservation status.

*Aphonopelma iodius* is one of the most widespread and abundant species in the United States. The species is secure.

#### Remarks.

*Aphonopelma iodius* is a problematic species. As presently defined, there are no major morphological features that can be used to distinguish *Aphonopelma iodius* from its sister lineages, and there is not enough evidence at this time to allow us to recognize cryptic diversity within the *Aphonopelma iodius* lineage (except for the geographically, genetically, and morphologically distinct *Aphonopelma johnnycashi*). During evaluation of traditional two-dimensional PCA morphospace and three-dimensional PCA morphospace (PC1~PC2~PC3), males and females of *Aphonopelma iodius* do not separate from any of the other species in the *iodius* species group (*Aphonopelma chalcodes*, *Aphonopelma eutylenum*, *Aphonopelma johnnycashi*), but do separate from *Aphonopelma steindachneri* and their syntopic miniature tarantulas (*Aphonopelma atomicum*, *Aphonopelma icenoglei*, *Aphonopelma joshua*, *Aphonopelma mojave*, and *Aphonopelma prenticei*) (see Suppl. material 2). PC1, PC2, and PC3 explain ≥95% of the variation in all analyses.

We examined the holotypes and freshly collected topotypic material of *Aphonopelma brunnius*, *Aphonopelma chamberlini*, *Aphonopelma iviei*, *Aphonopelma lithodomum*, *Aphonopelma smithi*, and *Aphonopelma zionis*. We also examined the holotypes and freshly collected topotypic material of three previously synonymized species: *Aphonopelma angusi*, *Aphonopelma melanium*, and *Aphonopelma nevadanum*. Our morphological and molecular analyses fail to recognize these nine species as separate, independently evolving lineages. As a consequence, we consider *Aphonopelma brunnius*, *Aphonopelma chamberlini*, *Aphonopelma iviei*, *Aphonopelma lithodomum*, *Aphonopelma smithi*, and *Aphonopelma zionis* junior synonyms of *Aphonopelma iodius* and confirm the synonymizations of *Aphonopelma angusi*, *Aphonopelma melanium*, and *Aphonopelma nevadanum* proposed by Prentice (1997). With additional sampling, however, it is possible that *Aphonopelma nevadanum* will be removed from synonymy.

Prentice formally changed the spelling of *Aphonopelma iodius* to *Aphonopelma iodium* because the gender is neuter. But, Chamberlin & Ivie (1939, pgs. 5–7) described Delopelma (Aphonopelma) iodius as “...easily distinguishable from *melanius*, which it apparently resembles most closely, by its rich rust color as contrasted with the dark metallic color of *melanius*.” It is also noted that in Chamberlin (1940), the key uses the word “blackish” to describe *Aphonopelma melanius* (since synonymized). Because *Aphonopelma iodius* (the more rust colored of the two) and *Aphonopelma melanius* (the more blackish of the two) were described next to each other (Chamberlin 1939), *Aphonopelma iodius* is the correct neuter comparative (*pers. comm.* H.D. Cameron).

Mitochondrial DNA (*CO1*) identifies *Aphonopelma iodius*, as presently defined, as a polyphyletic species with individuals grouping together with members of *Aphonopelma chalcodes* and *Aphonopelma johnnycashi* (Fig. 7). A number of these lineages were previously identified as putative novel species (Hamilton et al. 2014). While nuclear DNA also identifies *Aphonopelma iodius* as a polyphyletic group, it is providing more insight into the evolutionary history of the *Aphonopelma iodius* lineages (Fig. 8). *Aphonopelma iodius* comprises two monophyletic groups (one comprising nominotypical *Aphonopelma iodius* material from Utah and another comprising all remaining material). More specimens need to be sequenced for future coalescent species-tree analyses and gene flow evaluation before taxonomic change can be recommended. These results once again highlight how *CO1* is not effective at accurately delimiting species boundaries within this group.

### Aphonopelma johnnycashi

Taxon classificationAnimaliaAraneaeTheraphosidae

Hamilton sp. n.

http://zoobank.org/27F1F13C-F8A2-4FEF-A258-98C62F0F7139

**Figure 68.**
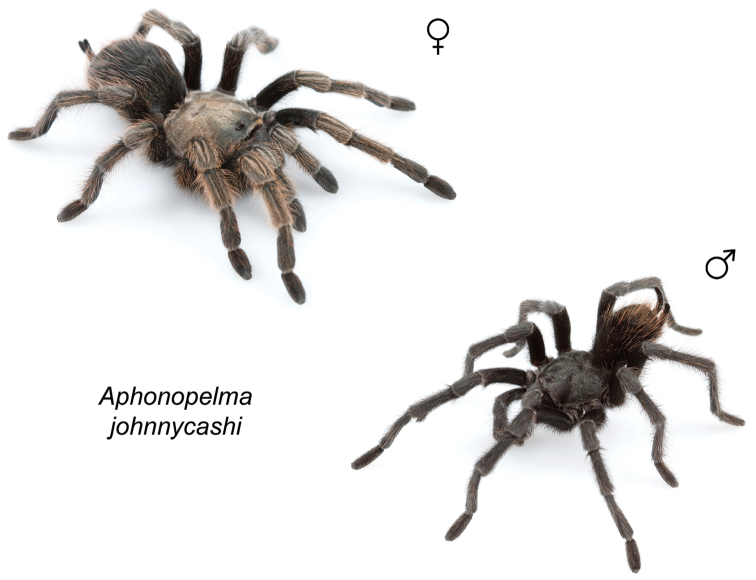
*Aphonopelma johnnycashi* sp. n. live photographs. Female (L) - APH_3073; Male (R) - APH_3063.

**Figure 69.**
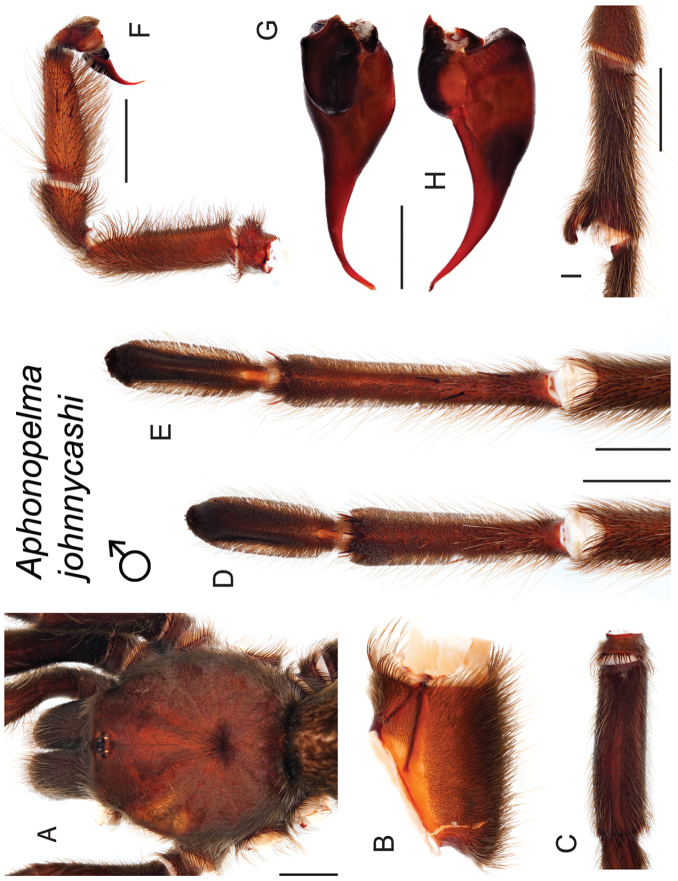
*Aphonopelma johnnycashi* sp. n. **A–I** male holotype, APH_2007 **A** dorsal view of carapace, scale bar = 4mm **B** prolateral view of coxa I **C** dorsal view of femur III **D** ventral view of metatarsus III, scale bar = 4mm **E** ventral view of metatarsus IV, scale bar = 3.5mm **F** prolateral view of L pedipalp and palpal tibia, scale bar = 4.5mm **G** dorsal view of palpal bulb **H** retrolateral view of palpal bulb, scale bar = 1mm **I** prolateral view of tibia I (mating clasper), scale bar = 5.5mm.

**Figure 70.**
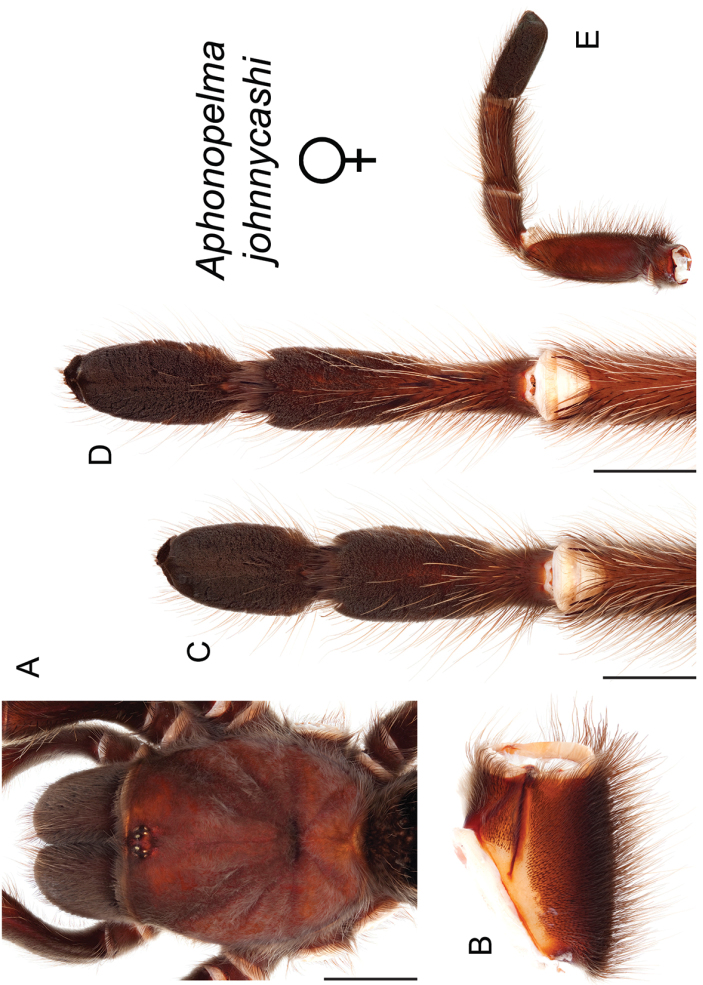
*Aphonopelma johnnycashi* sp. n. **A–E** female paratype, APH_3080 **A** dorsal view of carapace, scale bar = 7mm **B** prolateral view of coxa I **C** ventral view of metatarsus III, scale bar = 3.5mm **D** ventral view of metatarsus IV, scale bar = 4mm **E** prolateral view of L pedipalp and palpal tibia.

**Figure 71.**
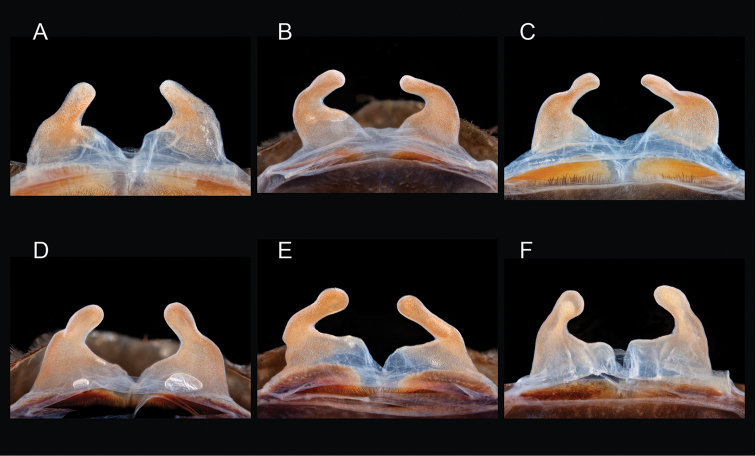
*Aphonopelma johnnycashi* sp. n. **A–F** cleared spermathecae **A** APH_2006 **B** APH_3064 **C** APH_3073 **D** APH_3080 **E** APH_3090 **F** APH_3094.

**Figure 72.**
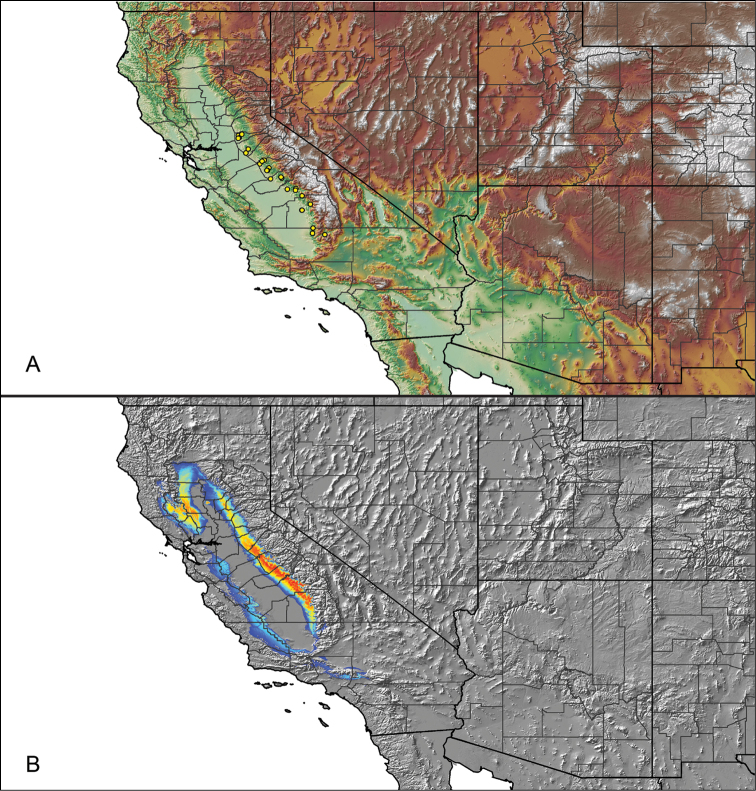
*Aphonopelma johnnycashi* sp. n. **A** distribution of known specimens **B** predicted distribution; warmer colors (red, orange, yellow) represent areas of high probability of occurrence, cooler colors (blue shades) represent areas of low probability of occurrence.

Figures 68 , 69 , 70 , 71 , 72

#### Types.

Male holotype (APH_2007) from NE of Ione, on Sutter Creek/Ione Rd, Amador Co., California, 38.37543 -120.90288 ^1^, elev. 570ft., 12.x.10, coll. Chris Hamilton; deposited in AUMNH. Paratype female (APH_3080) from Coarsegold, off Hwy 41, behind CAL Fire station, Madera Co., California, 37.2507 -119.70392 ^1^, elev. 2226ft., 10.vii.12, coll. Chris Hamilton; deposited in AUMNH. Paratype male (APH_2032) from Ione, Amador Co., California, 38.363017 -120.92695 ^1^, elev. 430ft., 1.x.10, coll. Mike Dame; deposited in AMNH. Paratype female (APH_3090) from off Rocky Hill Dr., E of Exeter - off 65, Rocky Hill, Tulare Co., California, 36.29807 -119.09829 ^1^, elev. 732ft., 12.vii.12, coll. Chris Hamilton; deposited in AMNH.

#### Etymology.

The specific epithet is in honor of the country music legend, Johnny Cash. This species can be found near the area of Folsom Prison in California, and like Cash’s distinctive style of dress (where he was referred to as “the man in black”), mature males of this species are generally black in color.

#### Diagnosis.

*Aphonopelma johnnycashi* (Fig. 68) is a member of the *iodius* species group and can be identified by a combination of morphological, molecular, and geographic characteristics. Nuclear DNA identifies *Aphonopelma johnnycashi* as a strongly supported monophyletic lineage within the *iodius* species group that is the sister lineage to *Aphonopelma eutylenum* and the paraphyletic *Aphonopelma iodius* (Fig. 8). There are no pronounced measurements or characters that help differentiate male or female *Aphonopelma johnnycashi* from *Aphonopelma iodius* (except that adult males of *Aphonopelma johnnycashi* are generally black whereas males of *Aphonopelma iodius* are brown or tan). Males and females of *Aphonopelma johnnycashi* can easily be differentiated from *Aphonopelma steindachneri* by color and the extent of scopulation on metatarsi III and IV.

Significant measurements that distinguish male *Aphonopelma johnnycashi* from its closely related phylogenetic and syntopic species are T3 and the extent of scopulation on metatarsus IV. Male *Aphonopelma johnnycashi* can be distinguished by possessing a larger T1/T3 (≥1.25; 1.25–1.31) than *Aphonopelma eutylenum* (≤1.23; 1.16–1.23); by possessing a larger A1/T3 (≥0.81; 0.81–0.91) than *Aphonopelma chalcodes* (≤0.79; 0.67–0.79); and by possessing a larger L4 scopulation extent (70%-76%) than *Aphonopelma steindachneri* (21%-31%). There are no significant measurements that separate male *Aphonopelma johnnycashi* from *Aphonopelma iodius*. The most significant measurement that distinguishes female *Aphonopelma johnnycashi* from *Aphonopelma steindachneri* is extent of scopulation on metatarsus IV. There are no significant measurements that separate female *Aphonopelma johnnycashi* from the other members of the *iodius* species group. Female *Aphonopelma johnnycashi* can be distinguished by possessing a larger L4 scopulation extent (67%-82%) than *Aphonopelma steindachneri* (24%-34%).

#### Description of male holotype

(APH_2007; Fig. 69). *Specimen preparation and condition*: Specimen collected live crossing road, preserved in 80% ethanol; original coloration faded due to preservation. Left legs I, III, IV, and left pedipalp removed for measurements and photographs; stored in vial with specimen. Right leg III removed for DNA and stored at -80°C in the AUMNH (Auburn, AL). *General coloration*: Black and faded black/brown. *Cephalothorax*: Carapace 13.71 mm long, 13.19 mm wide; Hirsute; densely clothed with dark brown/black iridescent pubescence mostly appressed to surface; fringe covered in long setae not closely appressed to surface; foveal groove medium deep and straight; pars cephalica region rises gradually from foveal groove, gently arching anteriorly toward ocular area; AER slightly procurved, PER very slightly recurved; normal sized chelicerae; clypeus very slightly extends forward on a curve; LBl 1.67, LBw 2.07; sternum hirsute, clothed with black, densely packed setae. *Abdomen*: Densely clothed in short black/brown pubescence with numerous longer red/orange setae interspersed; possessing a dense dorsal patch of black Type I urticating bristles (Cooke et al. 1972). *Legs*: Hirsute; densely clothed in a mix of black or faded black pubescence, femurs are darker. Metatarsus I slightly curved. F1 13.29; F1w 3.52; P1 5.04; T1 10.58; M1 10.57; A1 7.60; F3 11.05; F3w 3.55; P3 4.69; T3 8.31; M3 10.24; A3 7.04; F4 12.84; F4w 3.29; P4 4.90; T4 10.90; M4 13.72; A4 7.46; femur III is normal - not noticeably swollen or wider than other legs. All tarsi fully scopulate. Extent of metatarsal scopulation: leg III (SC3) = 75.8%; leg IV (SC4) = 70.9%. One ventral and one prolateral spinose seta on metatarsus III; three ventral spinose setae on metatarsus IV; one prolateral spinose seta on tibia I; one large megaspine present on the retrolateral tibia at the apex of the mating clasper - this can be seen when viewing the prolateral face of the mating clasper. *Coxa I*: Prolateral surface a mix of fine, hair-like and tapered/thin tapered setae. *Pedipalps*: Hirsute; densely clothed in the same setal color as the other legs, with numerous longer ventral setae; one spinose seta on the apical, prolateral femur and four spinose setae on the prolateral tibia; PTl 6.646, PTw 2.31. When extended, embolus tapers and gently curves to the retrolateral side near apex; embolus very slender, no keels.

**Variation (5).** Cl 13.42–13.71 (13.565±0.09), Cw 13.15–13.19 (13.17±0.01), LBl 1.54–1.67 (1.605±0.04), LBw 1.96–2.07 (2.015±0.03), F1 13.07–13.29 (13.18±0.07), F1w 3.23–3.52 (3.375±0.09), P1 5.04–5.69 (5.365±0.21), T1 10.58–11.23 (10.905±0.21), M1 10.57–10.74 (10.655±0.05), A1 7.6–7.69 (7.645±0.03), L1 length 47.08–48.42 (47.75±0.42), F3 11.05–11.16 (11.105±0.03), F3w 3.43–3.55 (3.49±0.04), P3 4.47–4.69 (4.58±0.07), T3 8.31–8.55 (8.43±0.08), M3 10.24–10.74 (10.49±0.16), A3 6.81–7.04 (6.925±0.07), L3 length 41.33–41.73 (41.53±0.13), F4 12.77–12.84 (12.805±0.02), F4w 3.16–3.29 (3.225±0.04), P4 4.7–4.9 (4.8±0.06), T4 10.9–11.33 (11.115±0.14), M4 13.72–14.53 (14.125±0.26), A4 7.46–7.85 (7.655±0.12), L4 length 49.82–51.18 (50.5±0.43), PTl 6.646–8.429 (7.538±0.56), PTw 2.31–2.801 (2.556±0.16), SC3 ratio 0.71–0.758 (0.734±0.02), SC4 ratio 0.709–0.762 (0.736±0.02), Coxa I setae = tapered/thin tapered, F3 condition = normal.

#### Description of female paratype

(APH_3080; Figs 70–71). *Specimen preparation and condition*: Specimen collected live from burrow, preserved in 80% ethanol; original coloration faded due to preservation. Left legs I, III, IV, and pedipalp removed for photographs and measurements; stored in vial with specimen. Right leg III removed for DNA and stored at -80°C in the AUMNH (Auburn, AL). Genital plate with spermathecae removed and cleared, stored in vial with specimen. *General coloration*: Faded brown/black. *Cephalothorax*: Carapace 16.96 mm long, 14.93 mm wide; Hirsute, densely clothed with brown pubescence closely appressed to surface; fringe densely covered in longer setae; foveal groove medium deep and straight; pars cephalica region gently rises from thoracic furrow, arching anteriorly toward ocular area; AER procurved, PER very slightly recurved; large chelicerae, clypeus mostly straight; LBl 2.16, LBw 2.48; sternum very hirsute, clothed with dark brown setae. *Abdomen*: Densely clothed dorsally in short black setae with numerous longer, lighter setae interspersed (generally red or orange *in situ*); dense dorsal patch of black Type I urticating bristles (Cooke et al. 1972); ventral side with shorter black/dark brown setae. *Spermathecae*: Paired and separate with wide bases, tapering and curving medially towards capitate bulbs. *Legs*: Very hirsute, particularly ventrally; densely clothed in medium and long brown pubescence, femurs darker. F1 12.88; F1w 4.03; P1 5.82; T1 10.26; M1 8.10; A1 6.49; F3 11.38; F3w 3.74; P3 5.14; T3 7.90; M3 8.27; A3 6.31; F4 13.58; F4w 3.89; P4 5.63; T4 10.18; M4 11.53; A4 7.09. All tarsi fully scopulate. Extent of metatarsal scopulation: leg III (SC3) = 88.1%; leg IV (SC4) = 67.4%. One ventral spinose seta on metatarsus III; five ventral spinose setae on metatarsus IV. *Coxa I*: Prolateral surface a mix of fine, hair-like and tapered setae. *Pedipalps*: Densely clothed in the same setal color as the other legs; one spinose seta on the apical, prolateral femur, three spinose setae on the prolateral patella, and six spinose setae on the prolateral tibia.

**Variation (6).** Cl 15.01–15.81 (15.41±0.23), Cw 13.89–14.01 (13.95±0.03), LBl 1.94–2.05 (1.995±0.03), LBw 2.47–2.48 (2.475±0), F1 11.56–12.64 (12.1±0.31), F1w 3.63–3.64 (3.635±0), P1 5.39–5.84 (5.615±0.13), T1 8.76–9.82 (9.29±0.31), M1 7.36–8.22 (7.79±0.25), A1 5.95–6.29 (6.12±0.1), L1 length 39.36–42.47 (40.915±0.9), F3 9.58–10.20 (9.89±0.18), F3w 3.16–3.18 (3.17±0.01), P3 4.63–4.64 (4.635±0), T3 6.91–7.52 (7.215±0.18), M3 7.61–7.94 (7.775±0.1), A3 5.84–5.86 (5.85±0.01), L3 length 34.57–36.16 (35.365±0.46), F4 11.92–12.28 (12.1±0.1), F4w 3.31–3.39 (3.35±0.02), P4 4.97–5.10 (5.035±0.04), T4 9.23–9.58 (9.405±0.1), M4 10.28–11.11 (10.695±0.24), A4 6.49–6.57 (6.53±0.02), L4 length 42.97–44.56 (43.765±0.46), SC3 ratio 0.774–0.917 (0.846±0.04), SC4 ratio 0.741–0.817 (0.779±0.02), Coxa I setae = tapered/thin tapered, F3 condition = normal. Spermathecae variation can be seen in Figure 71.

#### Material examined.

**United States: California: Amador**: N of Ione/SW of Plymouth, on Muller Rd, off Carbondale Rd and Hwy 6, 38.44191 -120.92164 ^1^, 678ft., [APH_2004-2006, 12/10/10, 1♀, 2 juv, Chris Hamilton, AUMNH]; NE of Ione, on Sutter Creek/Ione Rd, 38.37543 -120.90288 ^1^, 570ft., [APH_2007-2009, 12/10/10, 3♂, Chris Hamilton, AUMNH]; Ione, 38.363017 -120.92695 ^1^, 430ft., [APH_2032-2034, 1/10/10, 2♂, 1♀, Mike Dame, AUMNH & AMNH]; on Burke Dr, off Fiddletown Rd, E of Plymouth, 38.47597 -120.82309 ^1^, 1302ft., [APH_2038, 16/9/11, 1♂, Molly Taylor, AUMNH]; **Calaveras**: in between Angel’s Camp & Copperopolis; off Hwy ^4^, 38.04767 -120.64017 ^1^, 1517ft., [APH_3062-3064, 7/7/12, 2♂, 1♀, Chris Hamilton, AUMNH]; off Hwy 4; SW of Copperopolis, 37.94251 -120.71042 ^1^, 1150ft., [APH_3065-3066, 7/7/12, 1♀, 1♂, Chris Hamilton, AUMNH]; **Fresno**: western foothills of the Sierra Nevada mountains, 36.917882 -119.276034 ^8^, 1710ft., [APH_0032, 2005, 1♂, Amy Stockman, AUMNH]; Edison Point trailhead; near Pine Flat Reservoir; Trimmer Springs Rd., 36.8702 -119.2854 ^1^, 1177ft., [APH_3060-3061, 5/5/12, 1♀, 1 juv, Marshal Hedin, Jim Starrett, Dean Leavitt, AUMNH]; off Hwy 168 (Tollhouse Rd); near Tollhouse Rd & Sample Rd intersection, NE of Clovis & Fresno, 36.90073 -119.52076 ^1^, 598ft., [APH_3081-3084, 11/7/12, 3♀, 1♂, Chris Hamilton, AUMNH]; off Dunlap Rd. from Hwy 180; E of Squaw Valley, 36.71181 -119.09888 ^1^, 2333ft., [APH_3085-3086, 11/7/12, 2 juv, Chris Hamilton, AUMNH]; **Kern**: NE of Bakersfield ~20 miles; off Granite Rd, 35.63867 -118.80021 ^1^, 2858ft., [APH_3093-3095, 13/7/2012, 2♂, 1♀, Chris Hamilton, AUMNH]; off Whiteriver Rd/Old Stage Coach Rd; very close to Kern/Tulare county line, 35.785199 -118.783135 ^1^, 2410ft., [APH_3120, 5/4/13, 1 juv, Marshal Hedin, Jim Starrett, AUMNH]; Erskine Creek (Canyon), Erskine Creek road, 35.593637 -118.445971 ^5^ 2933ft., [AUMS_3322, 10/1970, 2♂, 1♀, J. Anderson, AUMNH]; **Madera**: 31901 Cherokee Rd, Coarsegold, 37.211624 -119.680988 ^2^, 2240ft., [APH_0197, 2/10/07, 1♂, Lowell Christie, AUMNH]; near entrance to Eastman Lake and Day Fee station; off Rd 29, 37.1971 -120.00248 ^1^, 441ft., [APH_3078-3079, 10/7/12, 1♀, 1♂, Chris Hamilton, AUMNH]; Coarsegold, off Hwy 41, behind CAL Fire station, 37.2507 -119.70392 ^1^, 2226ft., [APH_3080, 10/7/12, 1♀, Chris Hamilton, AUMNH]; **Mariposa**: off Hwy 49; N of Coulterville; near Don Pedro Reservoir, 37.7259 -120.20672 ^1^, 1842ft., [APH_3067-3069, 8/7/12, 2♂, 1♀, Chris Hamilton, AUMNH]; 5 miles S on Merced Falls Rd., on Hwy 132 - W of Hwy 49, intersection with Coronado Dr., 37.62829 -120.30836 ^1^, 1298ft., [APH_3070-3072, 8/7/12, 3♀, Chris Hamilton, AUMNH]; off J132; NE of Coulterville ~2 1/2 miles, 37.73476 -120.18259 ^1^, 2196ft., [APH_3073, 8/7/12, 1♀, Chris Hamilton, AUMNH]; off Hwy 49, N side of Mt. Bullion, 37.51229 -120.04727 ^1^, 2163ft., [APH_3074, 9/7/12, 1♀, Chris Hamilton, AUMNH]; off Hwy 140, Catheys Valley, Mariposa County Catheys Valley Park, 37.43761 -120.08519 ^1^, 1420ft., [APH_3075, 9/7/12, 1♀, Chris Hamilton, AUMNH]; off Hornitos Rd from Hwy 140; Catheys Valley, 37.44179 -120.10249 ^1^, 1350ft., [APH_3076-3077, 9/7/12, 1♂, 1♀, Chris Hamilton, AUMNH]; Horseshoe Bend Campground; just S of Hwy 132, 37.701605 -120.243205 ^1^, 948ft., [APH_3119, 1/4/13, 1♀, Marshal Hedin, Jim Starrett, AUMNH]; **Tulare**: Rocky Hill Rd, E of intersection with Rd 210; crossing road, 36.29664 -119.10703 ^1^, 485ft., [APH_0192, 14/9/2007, 1♂, Lorenzo Prendini, Jeremy Huff, AUMNH]; N of Three Rivers; off Hwy 198; near southern entrance to Sequoia N.P., 36.47036 -118.85903 ^1^, 1201ft., [APH_3087-3089, 12/7/12, 3♀, Chris Hamilton, AUMNH]; off Rocky Hill Dr., E of Exeter - off 65, Rocky Hill, 36.29807 -119.09829 ^1^, 732ft., [APH_3090-3092, 12/7/12, 1♂, 2♀, Chris Hamilton, AUMNH & AMNH].

#### Distribution and natural history.

*Aphonopelma johnnycashi* has a distribution running along the western foothills of the Sierra Nevada Mountains in California (Fig. 72) and can be found inhabiting the following Level III Ecoregions: Sierra Nevada, Central California Foothills and Coastal Mountains, and Central California Valley. *Aphonopelma johnnycashi* can be found in syntopy with *Aphonopelma steindachneri* across the most southern part of its distribution and there may be areas in the Tehachapi Mountains where *Aphonopelma johnnycashi* and *Aphonopelma iodius* co-occur. The breeding season, when mature males abandon their burrows in search of females, occurs during the fall (generally September–November).

#### Conservation status.

*Aphonopelma johnnycashi* is common across its distribution along the foothills and lower elevations of the western Sierra Nevada (Fig. 1D). The species is likely secure.

#### Remarks.

*Aphonopelma johnnycashi* is a member of the problematic *iodius* species group. As of now, we only have evidence to split *Aphonopelma johnnycashi* from the remaining populations of *Aphonopelma iodius*. Other important ratios that distinguish males: *Aphonopelma johnnycashi* possess a smaller T3/A3 (≤1.27; 1.18–1.27) than *Aphonopelma eutylenum* (≥1.30; 1.30–1.35) and *Aphonopelma steindachneri* (≥1.33; 1.33–1.52); by possessing a smaller F3/A3 (≤1.63; 1.56–1.63) than *Aphonopelma chalcodes* (≥1.63; 1.63–1.88); by possessing a larger L3 scopulation extent (71%–82%) than *Aphonopelma steindachneri* (40%–54%). Other important ratios that distinguish females: *Aphonopelma johnnycashi* possess a larger M1/M4 (≥0.70; 0.70–0.74) than *Aphonopelma steindachneri* (≤0.67; 0.62–0.67). For both males and females, certain morphometrics have potential to be useful, though due to the amounts of variation, small number of specimens, and the small differences between species, no others are claimed to be significant at this time (see Suppl. material 2). During evaluation of traditional two-dimensional PCA morphospace, male *Aphonopelma johnnycashi* separate from their syntopic species *Aphonopelma steindachneri*, but do not separate from the other species in the *iodius* species group (*chalcodes*, *eutylenum*, and *iodius*). Interestingly, when evaluating three-dimensional PCA morphospace (PC1~PC2~PC3), male *Aphonopelma johnnycashi* separates from *Aphonopelma eutylenum*, as well as *Aphonopelma steindachneri*. Female *Aphonopelma johnnycashi* separate in two-dimensional and three-dimensional morphospace from their syntopic species *Aphonopelma steindachneri*, but do not separate from the other species in the *iodius* species group. PC1, PC2, and PC3 explain ≥95% of the variation in all analyses. It is important to note the tremendous variation in spermathecae shape that can be seen across *Aphonopelma johnnycashi* populations (Fig. 71). Previous taxonomic work considered this variation enough to split and describe separate species; this is clearly not an effective character due to the large amounts of subtle variation that is possible.

Mitochondrial DNA (*CO1*) identifies *Aphonopelma johnnycashi* as a polyphyletic species with individuals grouping variously together with different lineages of *Aphonopelma iodius* (Fig. 7), likely indicative of past mitochondrial introgression. These results highlight how *CO1* is not effective at accurately delimiting species boundaries within this group.

### Aphonopelma joshua

Taxon classificationAnimaliaAraneaeTheraphosidae

Prentice, 1997

**Figure 73.**
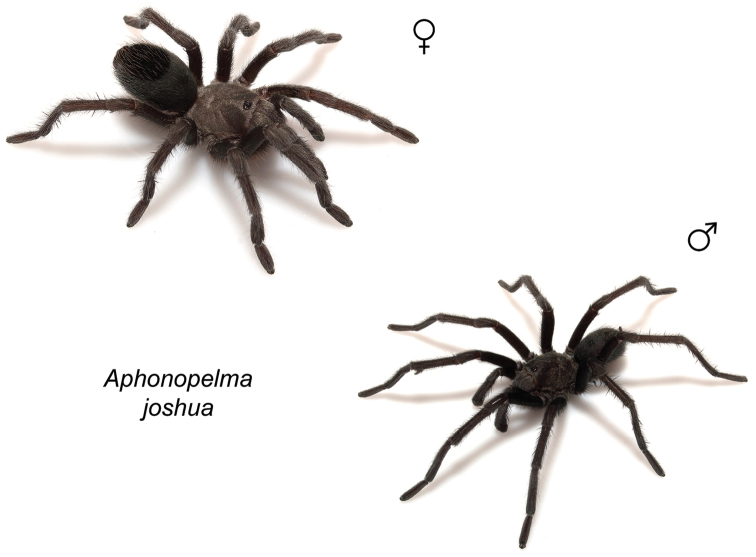
*Aphonopelma joshua* Prentice, 1997 specimens, live photographs. Female (L) - APH_1475; Male (R) - APH_1476.

**Figure 74.**
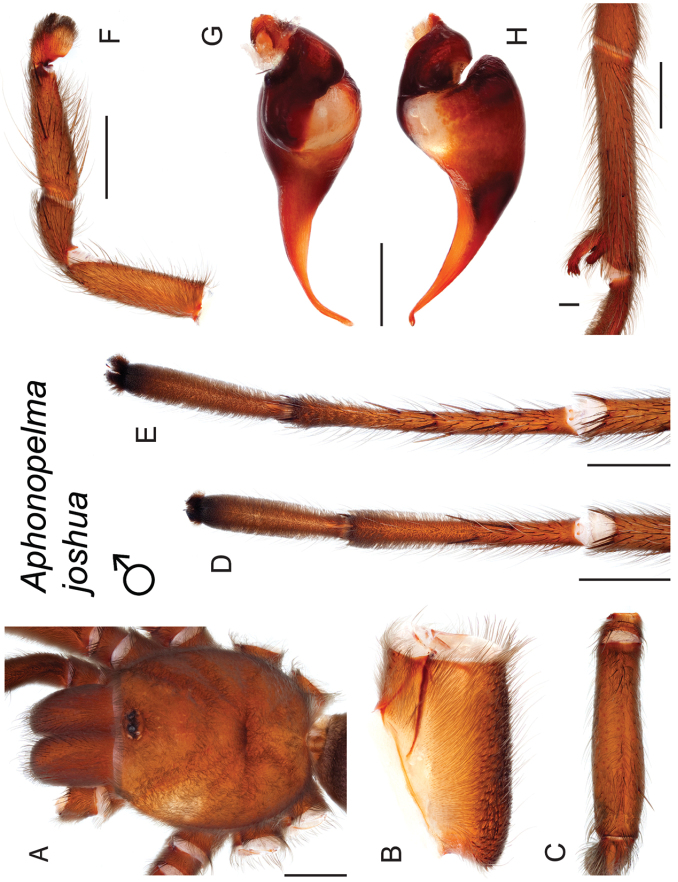
*Aphonopelma joshua* Prentice, 1997. **A–I** male specimen, APH_1476 **A** dorsal view of carapace, scale bar = 3mm **B** prolateral view of coxa I **C** dorsal view of femur III **D** ventral view of metatarsus III, scale bar = 3mm **E** ventral view of metatarsus IV, scale bar = 3mm **F** prolateral view of L pedipalp and palpal tibia, scale bar = 3mm **G** dorsal view of palpal bulb **H** retrolateral view of palpal bulb, scale bar = 0.5mm **I** prolateral view of tibia I (mating clasper), scale bar = 2.5mm.

**Figure 75.**
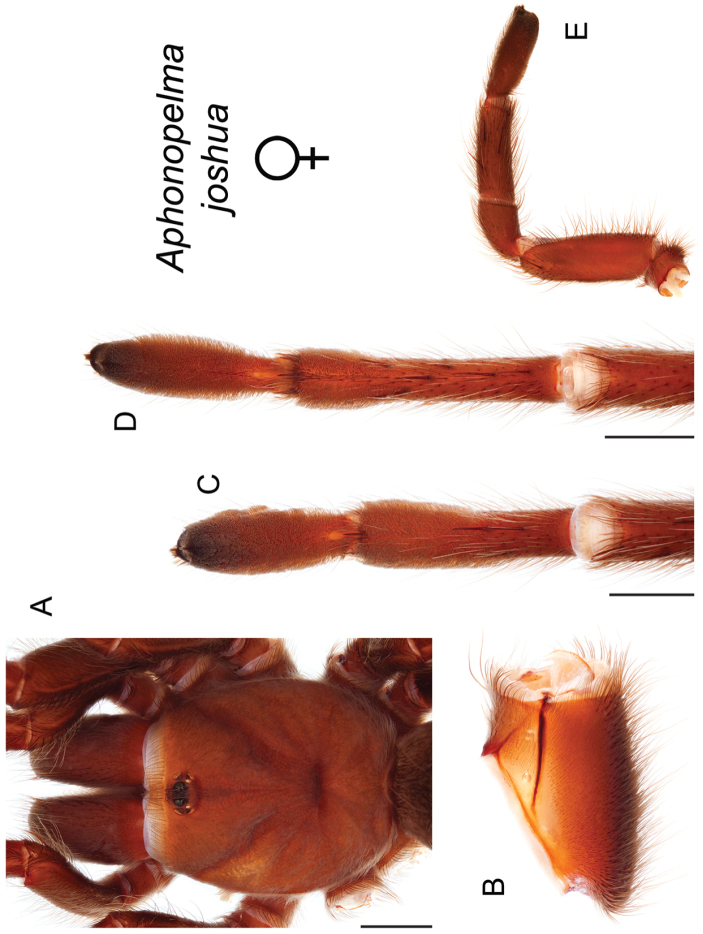
*Aphonopelma joshua* Prentice, 1997. **A–E** female specimen, APH_2306 **A** dorsal view of carapace, scale bar = 3mm **B** prolateral view of coxa I **C** ventral view of metatarsus III, scale bar = 2mm **D** ventral view of metatarsus IV, scale bar = 2mm **E** prolateral view of L pedipalp and palpal tibia.

**Figure 76.**
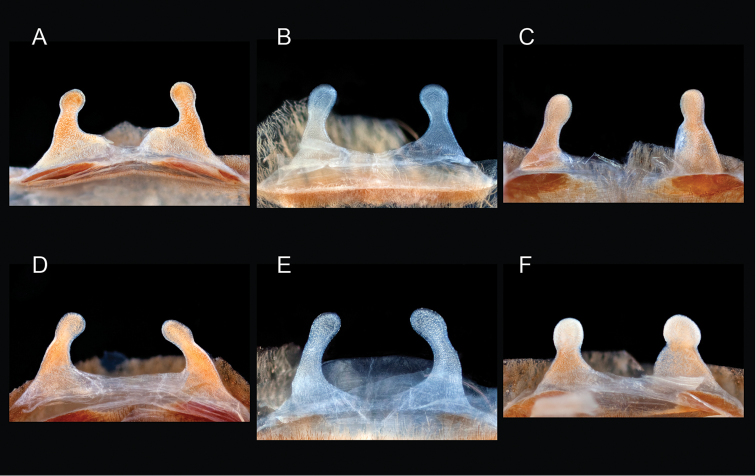
*Aphonopelma joshua* Prentice, 1997. **A–F** cleared spermathecae **A** *joshua* allotype **B** APH_2307 **C** APH_1007 **D** APH_2306 **E** APH_2285 **F** APH_2289.

**Figure 77.**
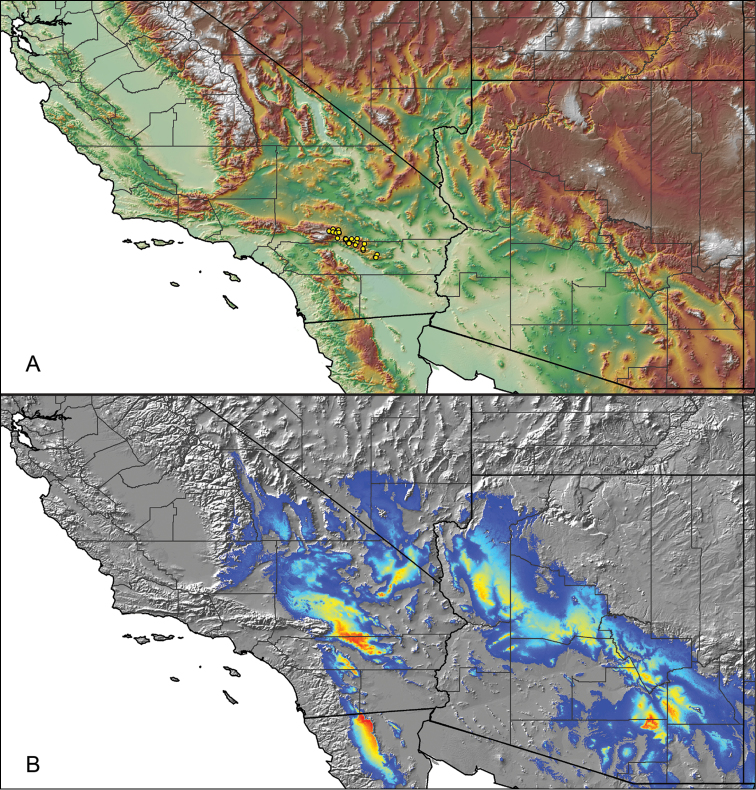
*Aphonopelma joshua* Prentice, 1997. **A** distribution of known specimens **B** predicted distribution; warmer colors (red, orange, yellow) represent areas of high probability of occurrence, cooler colors (blue shades) represent areas of low probability of occurrence.

Figures 73 , 74 , 75 , 76 , 77

Aphonopelma joshua Prentice, 1997: 150; male holotype from 2.3 mi. below the Covington Flats entrance to Joshua Tree National Park, San Bernardino Co., California, 34.045652 -116.315566^4^, elev. 4683ft., 6.ix.1992, coll. Thomas R. Prentice; deposited in AMNH. Allotype female from 5.6 miles into Joshua Tree National Park, in the Upper Covington Flat area, Riverside Co., California, 34.017254 -116.318644^4^, elev. 5027ft., 21.x.1989, coll. Thomas R. Prentice; deposited in AMNH. [examined]

#### Diagnosis.

*Aphonopelma joshua* (Fig. 73) is a member of the *paloma* species group and can be identified by a combination of morphological, molecular, and geographic characteristics. Nuclear and mitochondrial DNA identifies *Aphonopelma joshua* as a strongly supported monophyletic lineage (Figs 7–8) embedded within the turret-building group (see Hendrixson et al. 2013 and Graham et al. 2015) that includes (*Aphonopelma atomicum* sp. n., *Aphonopelma mojave*, *Aphonopelma icenoglei* sp. n., and *Aphonopelma prenticei* sp. n.). *Aphonopelma joshua* can easily be differentiated from other turret-building species by possessing stout sternal setae, from *Aphonopelma iodius* due to its smaller size and reduced extent of scopulation on metatarsus III and IV, and from other members of the *paloma* species group by their locality. The most significant measurement that distinguishes male *Aphonopelma joshua* from its closely related phylogenetic and syntopic species is A1. Male *Aphonopelma joshua* can be distinguished by possessing a smaller Cl/A1 (≤1.57; 1.42–1.57) than *Aphonopelma atomicum* (≥1.70; 1.70–1.93), *Aphonopelma iodius* (≥1.64; 1.64–2.23), *Aphonopelma icenoglei* (≥1.64; 1.64–1.97), *Aphonopelma mojave* (≥1.58; 1.58–1.94), *Aphonopelma prenticei* (≥1.68; 1.68–1.92), and *Aphonopelma xwalxwal* sp. n. (≥1.64; 1.64–1.75). The most significant measurements that distinguish female *Aphonopelma joshua* from its closely related phylogenetic and syntopic species are F1, A1, and the extent of scopulation on metatarsus IV. Female *Aphonopelma joshua* can be distinguished by possessing a larger A1/F3 (≥0.57; 0.57–0.64) than *Aphonopelma atomicum* (≤0.56; 0.53–0.56; by possessing a smaller F1/M4 (≤1.04; 0.98–1.04) than *Aphonopelma icenoglei* (≥1.07; 1.07–1.23) and *Aphonopelma prenticei* (≥1.04; 1.04–1.25); by possessing a smaller F1/T3 (≤1.57; 1.47–1.57) than *Aphonopelma mojave* (≥1.57; 1.57–1.92); and a smaller L4 scopulation extent (34%-40%) than *Aphonopelma iodius* (59%-83%). Females of *Aphonopelma xwalxwal* are unknown at this time and cannot be compared.

#### Description.

Male and female originally described by Prentice (1997).

#### Redescription of male exemplar

(APH_1476; Fig. 74). *Specimen preparation and condition*: Specimen collected live wandering, preserved in 80% ethanol; deposited in AUMNH; original coloration faded due to preservation. Left legs I, III, IV, and left pedipalp removed for measurements and photographs; stored in vial with specimen. Right legs III & IV removed for DNA and stored at -80°C in the AUMNH (Auburn, AL). *General coloration*: Black or faded black. *Cephalothorax*: Carapace 8.48 mm long, 7.26 mm wide; Hirsute; densely clothed with black or faded black pubescence mostly appressed to surface; fringe covered in long setae not closely appressed to surface; foveal groove medium deep and straight; pars cephalica region rises gradually from foveal groove to ocular area; AER procurved, PER slightly recurved; normal sized chelicerae; clypeus extends forward on a curve; LBl 1.153, LBw 1.557; sternum hirsute, clothed with short black or faded black, densely packed setae. *Abdomen*: Densely clothed in short black pubescence with numerous longer red/orange setae interspersed; possessing a distinct, dense dorsal patch of black Type I urticating bristles (Cooke et al. 1972) - smaller and distinct from large species. *Legs*: Hirsute; densely clothed in black/faded black pubescence. Metatarsus I straight. F1 8.73; F1w 1.81; P1 2.84; T1 8.05; M1 7.29; A1 5.41; F3 8.11; F3w 1.97; P3 3.20; T3 6.76; M3 8.37; A3 5.42; F4 9.42; F4w 1.34; P4 3.12; T4 8.44; M4 10.39; A4 6.30; femur III is swollen. All tarsi fully scopulate. Extent of metatarsal scopulation: leg III (SC3) = 68.3%; leg IV (SC4) = 39.3%. Three ventral and two prolateral spinose setae on metatarsus III, with numerous medium stout setae throughout; eight ventral spinose setae and one prolateral on metatarsus IV, with numerous medium stout setae throughout; three prolateral and three ventral spinose setae on tibia I; one large megaspine is present at the apex on the retrolateral tibia of the mating clasper; one megaspine on the prolateral branch of the mating clasper; two megaspines on either side of the apex of the retrolateral branch of the mating clasper, with numerous thickened setae throughout. *Coxa I*: Prolateral surface covered by fine, hair-like setae. *Pedipalps*: Very hirsute, particularly ventrally; densely clothed in the same setal color as the other legs; one spinose seta near the anterior margin of the prolateral palpal femur; one spinose seta on prolateral patella; four spinose setae and two ventral spinose setae on the prolateral palpal tibia, with numerous medium stout setae throughout; PTl 4.846, PTw 1.548. When extended, embolus tapers and gently curves to the retrolateral side; embolus slender, no keels.

**Variation (6).** Cl 7.549–8.48 (7.931±0.14), Cw 6.92–7.62 (7.264±0.11), LBl 1.101–1.339 (1.181±0.04), LBw 1.208–1.709 (1.417±0.08), F1 8.656–9.24 (8.941±0.1), F1w 1.81–2.145 (1.964±0.05), P1 2.84–3.48 (3.158±0.1), T1 7.899–8.96 (8.302±0.17), M1 7.135–7.868 (7.461±0.11), A1 5.13–5.41 (5.266±0.05), L1 length 31.749–34.445 (33.127±0.42), F3 8.028–8.527 (8.23±0.07), F3w 1.97–2.678 (2.5±0.11), P3 2.655–3.20 (2.978±0.1), T3 6.76–7.597 (7.071±0.14), M3 8.213–9.248 (8.707±0.16), A3 5.208–6.21 (5.542±0.14), L3 length 30.918–34.04 (32.527±0.51), F4 9.225–9.93 (9.565±0.11), F4w 1.34–2.084 (1.846±0.12), P4 2.82–3.14 (3.004±0.06), T4 8.172–9.04 (8.676±0.15), M4 10.39–11.45 (11.005±0.19), A4 5.23–6.30 (5.862±0.16), L4 length 36.896–39.52 (38.112±0.41), PTl 4.469–5.021 (4.786±0.08), PTw 1.433–1.58 (1.506±0.03), SC3 ratio 0.532–0.706 (0.628±0.03), SC4 ratio 0.242–0.393 (0.342±0.02), Coxa I setae = very thin tapered, F3 condition = swollen.

#### Description of female exemplar

(APH_2306; Figs 75–76). *Specimen preparation and condition*: Specimen collected live from burrow, preserved in 80% ethanol; deposited in AUMNH; original coloration faded due to preservation. Left legs I, III, IV, and pedipalp removed for photographs and measurements; stored in vial with specimen. No tissue for DNA. Genital plate with spermathecae removed and cleared, stored in vial with specimen. *General coloration*: Black or faded black. *Cephalothorax*: Carapace 9.15 mm long, 8.02 mm wide; Hirsute, densely clothed with short black or faded black pubescence closely appressed to surface; fringe densely covered in slightly longer setae; foveal groove medium deep and straight; pars cephalica region rises gradually from foveal groove to ocular area; AER slightly procurved, PER recurved; normal chelicerae, clypeus extends forward on a slight curve; LBl 1.36, LBw 1.51; sternum hirsute, clothed with short black or faded black setae. *Abdomen*: Densely clothed dorsally in short black or faded black setae with longer, lighter setae (generally red or orange *in situ*) focused near the urticating patch; small but dense dorsal patch of black Type I urticating bristles (Cooke et al. 1972) - smaller and distinct from large species. *Spermathecae*: Paired and separate, very simple, with capitate bulbs and wide bases that are not fused. *Legs*: Hirsute; densely clothed in short black or faded black pubescence; F1 7.23; F1w 2.05; P1 2.72; T1 6.30; M1 4.48; A1 3.74; F3 6.54; F3w 1.90; P3 2.82; T3 4.88; M3 5.62; A3 4.13; F4 8.11; F4w 2.07; P4 2.76; T4 6.58; M4 7.34; A4 4.28. All tarsi fully scopulate. Extent of metatarsal scopulation: leg III (SC3) = 66.9%; leg IV (SC4) = 39.9%. Three ventral spinose setae on metatarsus III; six ventral spinose setae and one prolateral on metatarsus IV, with numerous thicker setae throughout. *Coxa I*: Prolateral surface covered by fine, hair-like and very thin tapered setae. *Pedipalps*: Densely clothed in the same setal color as the other legs; one spinose seta on the apical, prolateral femur, six prolateral (two at the apical, prolateral border with the tarsus) and two ventral spinose setae (one at the apical, ventral border with the tarsus) on the tibia.

**Variation (6).** Cl 6.05–9.82 (8.238±0.59), Cw 5.13–8.02 (7.057±0.48), LBl 0.901–1.36 (1.188±0.07), LBw 1.133–1.52 (1.383±0.07), F1 5.048–8.45 (6.805±0.51), F1w 1.436–2.256 (1.869±0.14), P1 2.07–3.322 (2.712±0.22), T1 4.231–6.91 (5.813±0.4), M1 2.853–5.35 (4.235±0.4), A1 2.597–4.16 (3.541±0.22), L1 length 16.909–28.16 (23.106±1.72), F3 4.163–7.21 (5.864±0.45), F3w 1.319–2.154 (1.732±0.12), P3 1.839–2.92 (2.453±0.2), T3 3.241–5.49 (4.486±0.32), M3 3.484–5.82 (4.887±0.4), A3 2.98–4.147 (3.718±0.2), L3 length 15.707–25.52 (21.409±1.55), F4 5.668–8.55 (7.329±0.47), F4w 1.265–2.182 (1.818±0.14), P4 2.175–3.54 (2.745±0.22), T4 4.788–7.34 (6.271±0.38), M4 5.019–8.15 (6.732±0.48), A4 3.488–4.73 (4.195±0.22), L4 length 21.138–32.31 (27.766±1.99), SC3 ratio 0.627–0.696 (0.657±0.01), SC4 ratio 0.345–0.399 (0.368±0.01), Coxa I setae = very thin tapered. Spermathecae variation can be seen in Figure 76.

#### Material examined.

**United States: California: Riverside**: Joshua Tree National Park, 34.028757 -116.315665 ^6^, 4880ft., [APH_0883, 2007, 1♀, Josh Richards, AUMNH]; Joshua Tree National Park, 1 mile off Keys View Road, 33.87529 -116.038777 ^5^, 3589ft., [APH_2306, 3/5/1989, 1♀, T.R. Prentice, AMNH]; Joshua Tree National Park, Cottonwood Springs area, 33.743197 -115.811558 ^1^, 3116ft., [APH_1498-1499, 5/9/2012, 1♂, 1 juv, Brent E. Hendrixson, AUMNH]; [APH_2292, 23/8/1989, 1♂, T.R. Prentice, AMNH]; [APH_2300, 23/8/1993, 1♂, T.R. Prentice, AMNH]; [APH_2315, 23/8/1989, 1♂, T.R. Prentice, AMNH]; Joshua Tree National Park, Covington Flats area, 34.00862 -116.304733 ^1^, 4831ft., [APH_1475-1477, 29/7/2012, 1♂, 1♀, 1 juv, Brent E. Hendrixson, Brendon Barnes, Austin Deskewies, AUMNH]; [APH_2293, 27/7/1992, 1♂, T.R. Prentice, AMNH]; [APH_2301, 2/8/1962, 1♂, Jim Ortez, AMNH]; [AUMS_2567, 2/8/1962, 1♂, unknown, AUMNH]; Joshua Tree National Park, Hidden Valley Campground, 34.01651 -116.16179 ^1^, 4200ft., [APH_0335-0337, 8/5/2008, 3 juv, Brent E. Hendrixson, Zach Valois, AUMNH]; [APH_1200, 29/7/2010, 1♂, Brent E. Hendrixson, Brendon Barnes, Nate Davis, AUMNH]; Joshua Tree National Park, off El Dorado Mine Rd, Belle Campground, 33.99305 -116.0213 ^1^, 3872ft., [APH_1007, 10/5/2010, 1♀, Chris A. Hamilton, AUMNH]; Joshua Tree National Park, picnic area, Covington Flats area, 34.027454 -116.30194 ^1^, 4656ft., [APH_0492-0494, 16/5/2009, 3 juv, Brent E. Hendrixson, Bernadette DeRussy, Sloan Click, Jason Bond, AUMNH]; [APH_1199, 29/7/2010, 1 juv, Brent E. Hendrixson, Brendon Barnes, Nate Davis, AUMNH]; Joshua Tree National Park, Upper Covington Flats Area, near back country hiking trail parking area, 34.0085 -116.30694 ^1^, 4800ft., [APH_0329-0331, 8/5/2008, 3 juv, Brent E. Hendrixson, Zach Valois, AUMNH]; Lower Covington Flats, Joshua Tree National Monument, 34.004968 -116.307553 ^5^, 4861ft., [AUMS_2636, 31/7/1972, 1♂, Marqua, AUMNH]; [AUMS_2639, 31/7/1972, 1♂, D.G. Marqua, AUMNH]; Pleasant Valley, Joshua Tree National Park, 33.903782 -116.043557 ^5^, 4180ft., [APH_2291, 27/4/1965, 1♀, E.L. Sleeper and S.L. Jenkins, AMNH]; [APH_2316, 23/9/1967, 1♂, E.L. Sleeper and S.L. Jenkins, AMNH]; Smoke Tree Wash, In Joshua Tree National Park, 33.796978 -115.787031 ^5^, 2812ft., [APH_2296, 31/8/1989, 1♂, T.R. Prentice, AMNH]; Squaw Tank, Joshua Tree National Park, 34.087561 -116.153877 ^5^, 3442ft., [APH_2317, 9/9/1966, 1♂, E.L. Sleeper and S.L. Jenkins, AMNH]; **San Bernardino**: Burns Canyon, 8.3 miles west of Hwy 247 , 34.206942 -116.596597 ^4^, 5400ft., [APH_2290, 2/11/1991, 1♀, T.R. Prentice, AMNH]; [APH_2295, 2/11/1991, 1♀, T.R. Prentice, AMNH]; Burns Canyon, 8.3 miles west of Pipes Canyon Rd. , 34.227029 -116.668521 ^4^, 6096ft., [APH_2289, 2/11/1989, 1♀, T.R. Prentice, AMNH]; Covington Flats, 34.077473 -116.357061 ^5^, 4587ft., [APH_2302, 10/8/1989, 1♂, T.R. Prentice, AMNH]; [APH_2305, 10/8/1989, 1♂, T.R. Prentice, AMNH]; [APH_2308, 27/7/1990, 1♂, T.R. Prentice, AMNH]; [APH_2311, 27/12/1990, 1♂, T.R. Prentice, AMNH]; [APH_2313, 24/7/1989, 1♂, T.R. Prentice, AMNH]; [APH_2314, 3/8/1989, 1♂, T.R. Prentice, AMNH]; [AUMS_2479, 26/7/1990, 1♂, T.R. Prentice, AUMNH]; Covington Flats, below Joshua Tree National Park entrance, 34.051039 -116.342447 ^5^, 4577ft., [APH_2307, 30/7/1992, 1♀, T.R. Prentice, AMNH]; [APH_2312, 27/7/1992, 1♂, T.R. Prentice, AMNH]; [APH_2304, 28/7/1992, 1♂, T.R. Prentice, AMNH]; Covington Flats, area before the entrance, 34.077473 -116.357061 ^5^, 4587ft., [APH_2303, 28/7/1992, 1♂, T.R. Prentice, AMNH]; Covington Flats, in Joshua Tree National Monument, .5 miles north of split in the road, 34.071626 -116.367566 ^5^, 4206ft., [APH_2310, 12/8/1992, 1♂, T.R. Prentice, AMNH]; Joshua Tree National Monument, 4.5 miles SE of entrance on Quail Springs Road., 34.05728 -116.224505 ^4^, 3878ft., [APH_2286, 28/3/1989, 1♂, T.R. Prentice, AMNH]; Joshua Tree National Monument, below the entrance, 34.081762 -116.254111 ^5^, 3996ft., [APH_2309, 27/7/1992, 1♂, T.R. Prentice, AMNH]; Joshua Tree National Monument, Lost Horse Valley, 1.1 miles south of Quail Springs Rd on Keys View Rd, then 1 mile west, 33.968748 -116.184906 ^4^, 4469ft., [APH_2287, 3/5/1989, 1♂, T.R. Prentice, AMNH]; New Dixie Mine Rd, 6.3 miles west of Hwy 247, then .5 miles south, 34.264951 -116.5958 ^4^, 5331ft., [APH_2288, 11/11/1992, 1♀, T.R. Prentice, AMNH]; Pipes Canyon Road, 34.199004 -116.492984 ^5^, 4065ft., [APH_2294, 4/11/1989, 1♂, T.R. Prentice, AMNH]; Pipes Canyon, 2.5 miles west of Hwy 247 towards Burns Canyon, 34.194486 -116.479814 ^4^, 4377ft., [APH_2298, 18/4/1990, 1♂, T.R. Prentice, AMNH]; Pipes Canyon, 4.3 miles north of Hwy 247, 34.246525 -116.493804 ^4^, 4042ft., [APH_2297, 1/8/1992, 1♂, T.R. Prentice, AMNH]; Pipes Canyon, 5.9 miles west of Hwy 247 , 34.190832 -116.536894 ^4^, 4288ft., [APH_2299, 13/11/1992, 1♀, T.R. Prentice, AMNH]; Upper Morongo Valley, 5.1 miles north of the Post Office off Hwy 62., 34.093965 -116.512293 ^4^, 2799ft., [APH_2285, 6/9/1993, 1♀, T.R. Prentice, AMNH]; San Bernardino Mtns., Pipes Canyon road, 3.2 miles W of jct with Hwy 247, 34.198916 -116.486393 ^5^, 4000ft., [AUMS_3323, 6/9/1978, 1♂, W. Icenogle, AUMNH].

#### Distribution and natural history.

*Aphonopelma joshua* has a rather limited distribution within Joshua Tree National Park and surrounding areas in south-central San Bernardino and north-central Riverside Counties, California (Fig. 77). These spiders have been collected from elevations between 850 and 1500 meters in habitats characteristic of the Mojave and Sonoran deserts including the Eastern Mojave Low Ranges and Arid Footslopes Level III Ecoregion. The species distribution model suggests that suitable habitat may be present well beyond the boundaries of Joshua Tree National Park (Fig. 77B), but many of these regions are either unrealistically discontinuous (e.g., Arizona and northern Baja California) or already occupied by close relatives (e.g., *Aphonopelma xwalxwal* near the San Jacinto and Santa Rosa Mountains and *Aphonopelma prenticei* in Mojave National Preserve). Consequently, we feel that our sampling of *Aphonopelma joshua* provides a reasonable estimate of the extent of its distribution. This species is syntopic with *Aphonopelma iodius* (burrows of both species have been located within several meters of each other) and probably shares portions of its range with *Aphonopelma icenoglei* in the vicinity of Yucca Valley. Burrow entrances are generally surrounded by a distinct mound or turret made of excavated soil and silk (Fig. 2D–E). Mating is nocturnal and occurs during the summer (July-September, earlier in the northwestern portion of its range and later in the southeastern portion). A more extensive account of the natural history of *Aphonopelma joshua* is provided by Prentice (1997).

#### Conservation status.

Despite its narrow distribution, *Aphonopelma joshua* is largely protected by Joshua Tree National Park. Recreational activities may pose some concern but this species is common throughout the park and is likely secure.

#### Remarks.

*Aphonopelma joshua* is one of the larger species of miniature *Aphonopelma* and is morphologically very similar to the new species *Aphonopelma xwalxwal*. Other important ratios that distinguish males: *Aphonopelma joshua* possess a smaller F1/M4 (≤0.84; 0.78–0.84) than *Aphonopelma atomicum* (≥0.89; 0.89–0.91), *Aphonopelma iodius* (≥0.89; 0.89–1.04), *Aphonopelma icenoglei* (≥0.90; 0.90–1.02), *Aphonopelma mojave* (≥0.87; 0.87–0.94), *Aphonopelma prenticei* (≥0.91; 0.91–0.95); by possessing a smaller L1/L3 (≤1.03; 0.99–1.03) than *Aphonopelma iodius* (≥1.05; 1.05–1.20), *Aphonopelma mojave* (≥1.06; 1.06–1.11), *Aphonopelma prenticei* (≥1.05; 1.05–1.11), *Aphonopelma xwalxwal* (≥1.07; 1.07–1.12); by possessing a smaller L3 scopulation extent (53%-71%) than *Aphonopelma iodius* (78%-96%); by possessing a smaller L4 scopulation extent (24%-39%) than *Aphonopelma iodius* (62%-88%); by possessing a smaller M1/F3 (<0.92; 0.88–0.92) than *Aphonopelma xwalxwal* (>0.98; 0.98–1.02). Other important ratios that distinguish females: *Aphonopelma joshua* possess a larger Cl/P1 (≥2.77; 2.77–3.53) than *Aphonopelma atomicum* (≤2.50; 2.43–2.50); by possessing a smaller L1/Cl (≤2.87; 2.67–2.87) than *Aphonopelma icenoglei* (≥2.92; 2.92–3.13); by possessing a smaller Cl/M3 (≤1.74; 1.62–1.74) than *Aphonopelma prenticei* (≥1.77; 1.77–2.20); by possessing a smaller A1/T4 (≤0.59; 0.54–0.59) than *Aphonopelma mojave* (≥0.58; 0.58–0.67; small overlap). For both males and females, certain morphometrics have potential to be useful, though due to the amounts of variation, small number of specimens, and the small differences between species, no others are claimed to be significant at this time (see Suppl. material 2). During evaluation of traditional PCA morphospace, males of *Aphonopelma joshua* separate in PCA morphological space from *Aphonopelma atomicum*, *Aphonopelma iodius*, *Aphonopelma icenoglei*, *Aphonopelma mojave*, and *Aphonopelma prenticei* but do not separate from *Aphonopelma xwalxwal*. Female *Aphonopelma joshua* separate from *Aphonopelma iodius* and *Aphonopelma prenticei* in morphological space, but do not separate from *Aphonopelma atomicum*, *Aphonopelma icenoglei*, and *Aphonopelma mojave*. Interestingly, *Aphonopelma joshua* males separate from *Aphonopelma iodius* and *Aphonopelma xwalxwal*, as well as *Aphonopelma atomicum*, *Aphonopelma icenoglei*, *Aphonopelma mojave*, and *Aphonopelma prenticei* in three-dimensional PCA morphospace (PC1~PC2~PC3). *Aphonopelma joshua* females separate from *Aphonopelma iodius* and *Aphonopelma prenticei* but do not separate from *Aphonopelma atomicum*, *Aphonopelma icenoglei*, or *Aphonopelma mojave*. PC1, PC2, and PC3 explain ≥97% of the variation in all analyses.

### Aphonopelma madera

Taxon classificationAnimaliaAraneaeTheraphosidae

Hamilton, Hendrixson & Bond sp. n.

http://zoobank.org/E1E09A39-EA97-4C9B-931A-58EC5978C259

**Figure 78.**
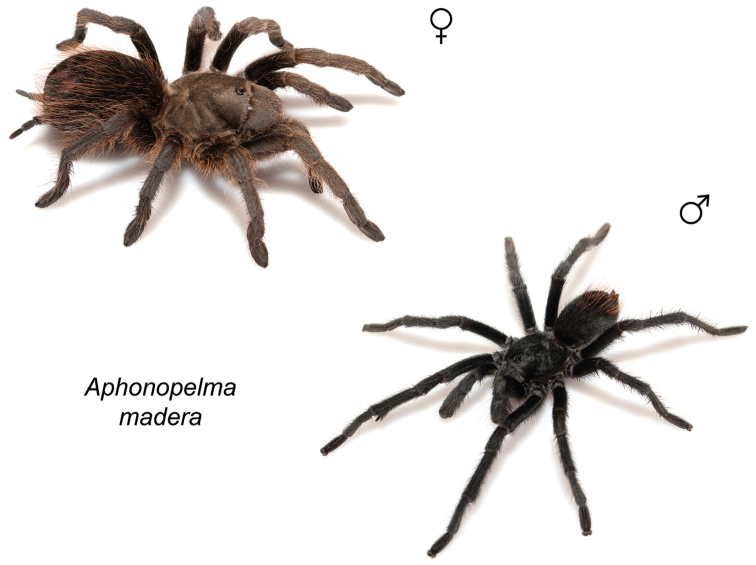
*Aphonopelma madera* sp. n. live photographs. Female paratype (L) - APH_1393; Male (R) - APH_1434.

**Figure 79.**
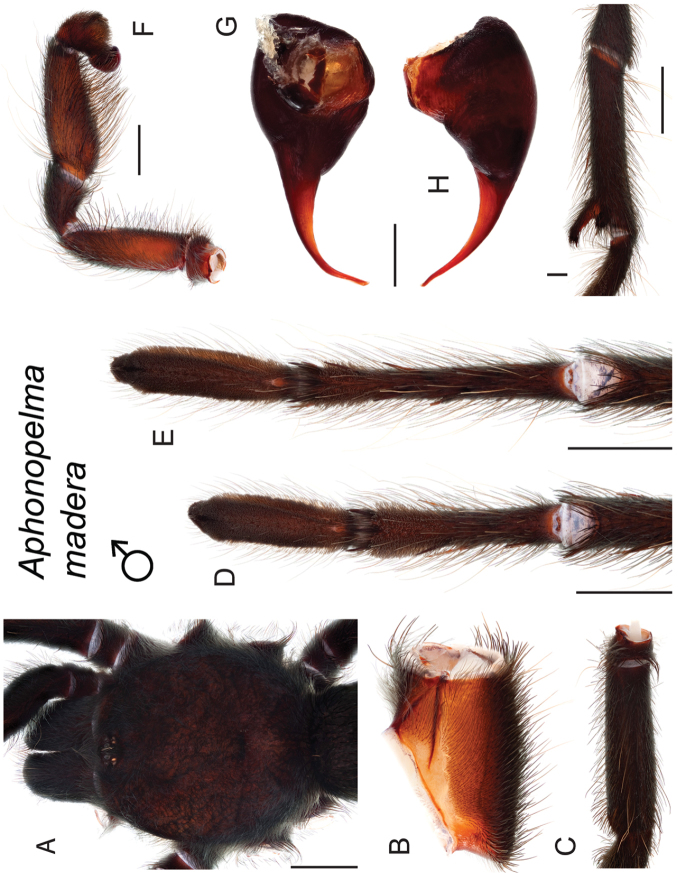
*Aphonopelma madera* sp. n. **A–I** male holotype, APH_3177 **A** dorsal view of carapace, scale bar = 2.5mm **B** prolateral view of coxa I **C** dorsal view of femur III **D** ventral view of metatarsus III, scale bar = 2.5mm **E** ventral view of metatarsus IV, scale bar = 3mm **F** prolateral view of L pedipalp and palpal tibia, scale bar = 2mm **G** dorsal view of palpal bulb **H** retrolateral view of palpal bulb, scale bar = 0.5mm **I** prolateral view of tibia I (mating clasper), scale bar = 3mm.

**Figure 80.**
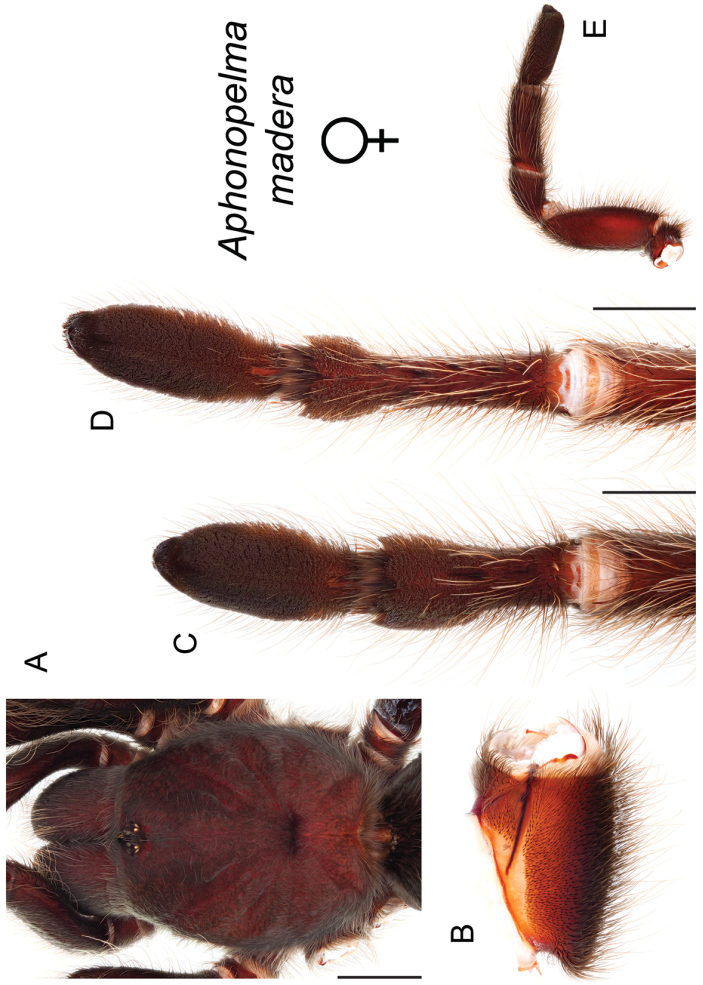
*Aphonopelma madera* sp. n. **A–E** female paratype, APH_1393 **A** dorsal view of carapace, scale bar = 5.5mm **B** prolateral view of coxa I **C** ventral view of metatarsus III, scale bar = 2.5mm **D** ventral view of metatarsus IV, scale bar = 3mm **E** prolateral view of L pedipalp and palpal tibia.

**Figure 81.**
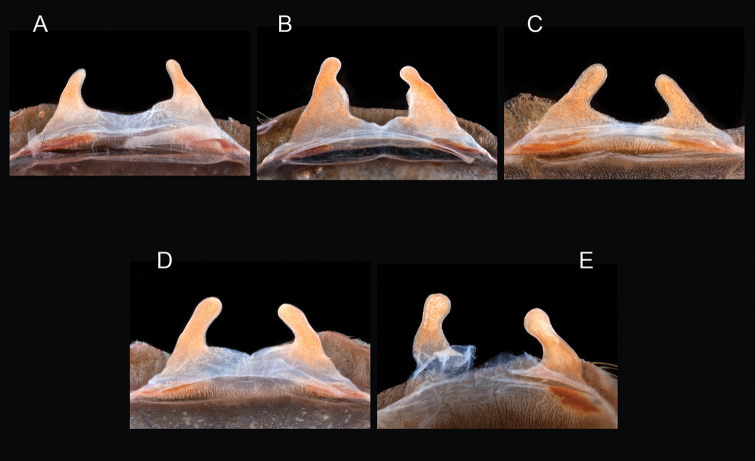
*Aphonopelma madera* sp. n. **A–E** cleared spermathecae **A** APH_1393 **B** APH_0136 **C** APH_0881 **D** APH_1571 **E** APH_1624.

**Figure 82.**
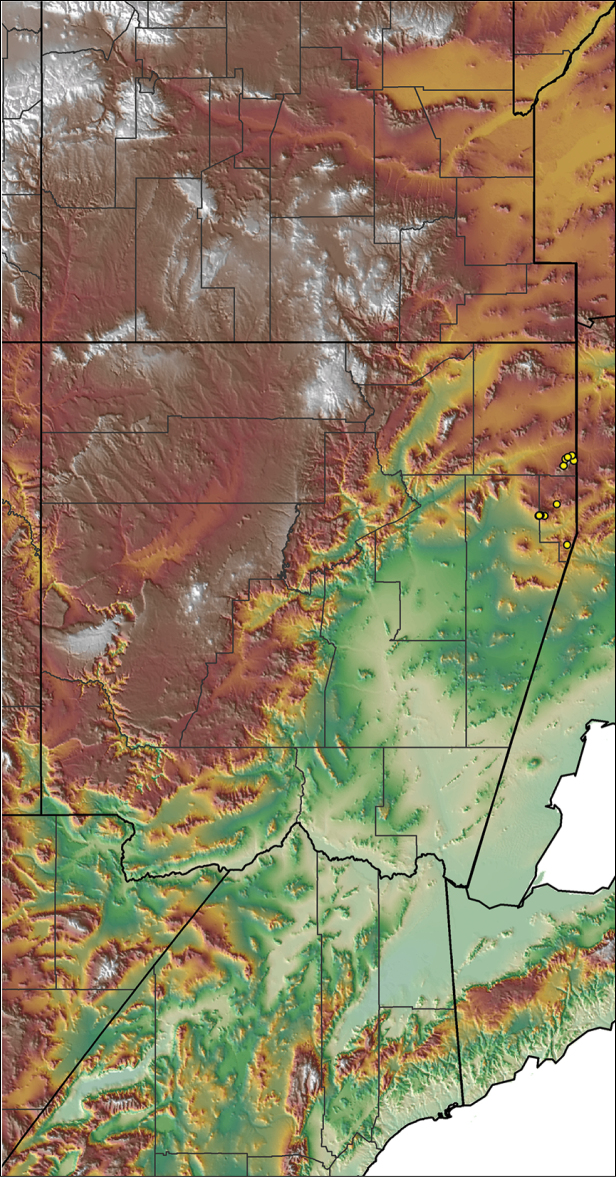
*Aphonopelma madera* sp. n. distribution of known specimens. There is no predicted distribution map due to the limited number of sampling localities and restricted distribution this species possesses.

Figures 78 , 79 , 80 , 81 , 82

#### Types.

Male holotype (APH_3177) from Madera Canyon, Coronado National Forest, on road to Bog Springs Campground, Pima Co., Arizona, 31.72812 -110.878818 ^1^, elev. 4809ft., 12.xi.2013, coll. Chris A. Hamilton and Brent E. Hendrixson; deposited in AUMNH. Paratype female (APH_1393) from Madera Canyon, Mt. Wrightson picnic area, Santa Cruz Co., Arizona, 31.71273 -110.874936 ^1^, elev. 5401ft., 5.x.2011, coll. Brent E. Hendrixson and Thomas Martin; deposited in AUMNH. Paratype male (APH_1627) from Madera Canyon, Madera Picnic Area, Cochise Co., Arizona, 31.72722 -110.880812 ^1^, elev. 4809ft., 15.xi.2012, coll. Brent E. Hendrixson; deposited in AMNH. Paratype female (APH_1571) from Madera Canyon, Bog Springs Campground, Santa Cruz Co., Arizona, 31.72733 -110.875018 ^1^, elev. 5051ft., 27.x.2012, coll. Brent E. Hendrixson; deposited in AMNH.

#### Etymology.

The specific epithet is a noun in apposition taken from type locality, Madera Canyon, in the Santa Rita Mountains where this species was first discovered.

#### Diagnosis.

*Aphonopelma madera* (Fig. 78) is a member of the *Marxi* species group and can be distinguished by a combination of morphological, molecular, and geographic characteristics. Nuclear and mitochondrial DNA identifies *Aphonopelma madera* as a phylogenetically distinct monophyletic lineage (Figs 7–8), supported as the sister lineage to *Aphonopelma catalina* sp. n. (a species endemic to the Santa Catalina Mountains) and *Aphonopelma chiricahua* sp. n. (a species endemic to the Chiricahua Mountains). The significant measurements that distinguish male *Aphonopelma madera* from its closely related phylogenetic and syntopic species are Cl and A3. Male *Aphonopelma madera* can be distinguished by possessing a larger Cl/M3 (≥1.51; 1.51–1.60) than *Aphonopelma catalina* (≤1.42; 1.26–1.42), *Aphonopelma peloncillo* sp. n. (≤1.40; 1.20–1.40), *Aphonopelma vorhiesi* (≤1.43; 1.24–1.43), and *Aphonopelma chalcodes* (≤1.44; 1.15–1.44); and a larger Cl/A3 (≥1.86; 1.86–2.18) than *Aphonopelma chiricahua* (≤1.74; 1.53–1.74). Significant measurements that distinguish female *Aphonopelma madera* from its closely related phylogenetic and syntopic species are M3 and A4. Female *Aphonopelma madera* can be distinguished by possessing a smaller M3/A4 (≤1.07; 0.96–1.07) than *Aphonopelma catalina* (≥1.07; 1.07–1.10), Aphonopelma chalcodes (≥1.12; 1.12–1.49), and *Aphonopelma peloncillo* (≥1.11; 1.11–1.23), but larger than *Aphonopelma chiricahua* (0.80 ± (only 1 specimen).

#### Description of male holotype

(APH_3177; Fig. 79). *Specimen preparation and condition*: Specimen collected live crossing road, preserved in 80% ethanol; original coloration faded due to preservation. Left legs I, III, IV, and left pedipalp removed for measurements and photographs; stored in vial with specimen. Right legs II & III removed for DNA and stored at -80°C in the AUMNH (Auburn, AL). *General coloration*: Generally black or faded black. *Cephalothorax*: Carapace 7.81 mm long, 7.48 mm wide; densely clothed with black/faded black pubescence, slightly appressed to surface and longer than lower elevation species; fringe covered in long setae not closely appressed to surface; foveal groove medium deep and straight; pars cephalica region rises gradually from foveal groove, gently arching anteriorly toward ocular area; AER slightly procurved, PER very slightly recurved; normal sized chelicerae; clypeus slightly extends forward on a curve; LBl 0.99, LBw 1.14; sternum hirsute, clothed with short black, densely packed setae. *Abdomen*: Densely clothed in short black pubescence with numerous longer, lighter setae interspersed (generally red or orange *in situ*); dense dorsal patch of black Type I urticating bristles (Cooke et al. 1972); ventral setae same as dorsal. *Legs*: Hirsute; densely clothed with short, similar length black setae, and longer setae interspersed. Metatarsus I very slightly curved. F1 8.37; F1w 1.92; P1 3.28; T1 7.32; M1 5.01; A1 3.85; F3 6.21; F3w 1.96; P3 2.58; T3 4.98; M3 5.04; A3 4.14; F4 7.52; F4w 1.83; P4 2.60; T4 6.76; M4 7.04; A4 4.83; femur III is normal - not noticeably swollen or wider than other legs. All tarsi fully scopulate. Extent of metatarsal scopulation: leg III (SC3) = 45.9%; leg IV (SC4) = 28.4%. Three ventral spinose setae, two prolateral spinose setae and one retrolateral spinose seta on metatarsus III; eight ventral spinose setae, and one prolateral spinose seta on metatarsus IV; three ventral spinose setae on tibia I; one large megaspine is present on the retrolateral tibia at the apex of the mating clasper - this can be seen when viewing the prolateral face of the mating clasper. *Coxa I*: Prolateral surface a mix of fine, hair-like and very thin tapered setae. *Pedipalps*: Hirsute; densely clothed in the same setal color as the other legs, with numerous longer ventral setae; one spinose seta at the apical, prolateral femur and three spinose setae on the prolateral tibia; PTl 4.947, PTw 2.158. Embolus shorter and stockier than lower elevation species; when extended, embolus gently and quickly tapers and curves to the retrolateral side near apex; embolus slender, no keels.

**Variation (6).** Cl 7.815–9.43 (8.488±0.22), Cw 7.478–8.57 (7.861±0.16), LBl 0.986–1.138 (1.065±0.03), LBw 1.139–1.449 (1.321±0.06), F1 8.374–9.78 (8.918±0.22), F1w 1.917–2.11 (2.012±0.03), P1 3.016–3.59 (3.302±0.08), T1 7.321–8.89 (7.813±0.23), M1 4.977–5.52 (5.213±0.1), A1 3.542–4.84 (4.135±0.22), L1 length 27.831–32.29 (29.38±0.7), F3 6.215–7.06 (6.611±0.12), F3w 1.9–2.13 (1.991±0.03), P3 2.581–3.136 (2.851±0.09), T3 4.618–5.70 (5.029±0.15), M3 5.038–5.89 (5.467±0.12), A3 3.945–4.56 (4.29±0.1), L3 length 22.958–26.13 (24.247±0.46), F4 7.521–8.92 (8.154±0.21), F4w 1.82–1.99 (1.905±0.03), P4 2.603–3.234 (2.983±0.09), T4 6.379–7.51 (6.92±0.16), M4 7.043–8.20 (7.58±0.2), A4 4.288–5.89 (5.027±0.25), L4 length 28.706–33.63 (30.663±0.83), PTl 4.947–6.201 (5.447±0.17), PTw 1.79–2.158 (2.043±0.06), SC3 ratio 0.459–0.702 (0.548±0.03), SC4 ratio 0.238–0.481 (0.339±0.03), Coxa I setae = very thin tapered, F3 condition = normal.

#### Description of female paratype

(APH_1393; Figs 80–81). *Specimen preparation and condition*: Specimen collected live from burrow, preserved in 80% ethanol. Left legs I, III, IV, and pedipalp removed for photographs and measurements; stored in vial with specimen. Right leg III removed for DNA and stored at -80°C in the AUMNH (Auburn, AL). Genital plate with spermathecae removed and cleared, stored in vial with specimen. *General coloration*: Black/faded black and brown. *Cephalothorax*: Carapace 15.72 mm long, 13.73 mm wide; Hirsute, densely clothed with black/faded black, pubescence closely appressed to surface; fringe densely covered in longer setae; foveal groove medium deep and straight; pars cephalica region gently rises from thoracic furrow, arching anteriorly toward ocular area; AER very slightly procurved, PER slightly recurved; robust chelicerae, clypeus very slightly extends forward on a curve; LBl 1.76, LBw 2.19; sternum very hirsute, clothed with longer black/faded black setae. *Abdomen*: Densely clothed dorsally in short black setae with numerous longer, lighter setae interspersed (generally red or orange *in situ*); dense dorsal patch of black Type I urticating bristles (Cooke et al. 1972); ventral setae same as dorsal. *Spermathecae*: Paired and separate, very basic, very slight taper curve medially towards capitate bulbs, with wide bases that appear fused. *Legs*: Very hirsute; densely clothed with longer setae colored similarly as the long abdominal setae; F1 12.62; F1w 3.82; P1 5.31; T1 9.22; M1 6.56; A1 5.58; F3 8.79; F3w 3.34; P3 4.52; T3 6.84; M3 6.81; A3 5.73; F4 12.38; F4w 3.58; P4 4.87; T4 9.55; M4 9.91; A4 6.47. All tarsi fully scopulate. Extent of metatarsal scopulation: leg III (SC3) = 59.9%; leg IV (SC4) = 33.2%. Three ventral spinose setae on metatarsus III; eight ventral spinose setae, two retrolateral spinose setae (one near the basal border with the tibia), and one prolateral spinose seta on metatarsus IV. *Coxa I*: Prolateral surface a mix of fine, hair-like and tapered/thin tapered setae. *Pedipalps*: Densely clothed in the same setal color as the other legs; one spinose seta on the apical, prolateral femur, six prolateral spinose setae and one ventral spinose seta on the tibia (three at the apical border with the tarsus).

**Variation (5).** Cl 9.17–15.72 (12.176±1.11), Cw 8.43–13.73 (10.914±0.95), LBl 1.18–1.76 (1.475±0.09), LBw 1.51–2.19 (1.781±0.11), F1 7.332–12.62 (9.914±0.86), F1w 2.342–3.82 (3.088±0.25), P1 3.046–5.31 (4.265±0.39), T1 5.999–9.22 (7.798±0.57), M1 3.416–6.56 (5.147±0.55), A1 3.951–5.58 (4.83±0.27), L1 length 23.744–39.29 (31.955±2.62), F3 7.36–8.79 (8.203±0.34), F3w 2.46–3.34 (2.845±0.19), P3 3.34–4.52 (3.768±0.27), T3 5.07–6.84 (5.898±0.37), M3 5.11–6.81 (6±0.38), A3 4.69–5.73 (5.253±0.22), L3 length 25.67–32.69 (29.12±1.5), F4 7.514–12.38 (9.841±0.8), F4w 2.188–3.58 (2.816±0.25), P4 3.234–4.87 (4.003±0.29), T4 6.137–9.55 (7.823±0.59), M4 6.197–9.91 (8.183±0.66), A4 4.842–6.47 (5.694±0.29), L4 length 27.924–43.18 (35.545±2.61), SC3 ratio 0.483–0.684 (0.595±0.04), SC4 ratio 0.296–0.348 (0.33±0.01), Coxa I setae = tapered/thin tapered. Spermathecae variation can be seen in Figure 81.

#### Material examined.

**United States: Arizona: Cochise**: Carr Canyon Rd, 31.449658 -110.282028 ^1^, 5257ft., [APH_1595, 8/11/2012, 1♂, Brent E. Hendrixson, AUMNH]; Copper Canyon, Huachuca Mtns, 31.363172 -110.29979 ^5^, 6082ft., [APH_0881, 2007, 1 juv, Josh Richards, AUMNH]; [APH_0980, 2007, 1♀, Josh Richards, AUMNH]; Huachuca Mtns, Ash Canyon, 31.38339 -110.24486 ^2^, 5290ft., [APH_1249, 11/2010, 1♂, Jim Murray, AUMNH]; Huachuca Mtns, Garden Canyon Rd, 31.47306 -110.35111 ^2^, 5360ft., [APH_1250-1251, 4/12/2010, 2♂, AMNH]; Huachuca Mountains, Miller Canyon, primitive campsite, 31.42476 -110.26082 ^2^, 5238ft., [APH_3218, 20/5/2014, 1 juv, Brent E. Hendrixson, Dustin Garig, Harrison Olinger, AUMNH]; **Pima**: 0.67 miles N Santa Cruz County line on Madera Canyon Rd, 31.73588 -110.88232 ^2^, 4601ft., [APH_1434, 8/11/2011, 1♂, June Olberding, AUMNH]; [APH_1435, 11/11/2011, 1♂, June Olberding, AUMNH]; Madera Canyon Rd, 31.728908 -110.880462 ^1^, 4803ft., [APH_1624, 15/11/2012, 1♀, Brent E. Hendrixson, AUMNH]; [APH_1626, 15/11/2012, 1♂, Brent E. Hendrixson, AUMNH]; Madera Canyon, Bog Springs Campground, 31.72733 -110.875018 ^1^, 5051ft., [APH_1571, 27/10/2012, 1♀, Brent E. Hendrixson, AMNH]; [APH_1628, 15/11/2012, 1♂, Brent E. Hendrixson, AUMNH]; [APH_1630, 15/11/2012, 1♂, Brent E. Hendrixson, AUMNH]; Madera Canyon, Coronado National Forest, on road to Bog Springs Campground, 31.72812 -110.878818 ^1^, 4809ft., [APH_3177, 12/11/2013, 1♂, Chris A. Hamilton, Brent E. Hendrixson, AUMNH]; Madera Canyon, Madera Picnic Area, 31.72722 -110.880812 ^1^, 4809ft., [APH_1625, 15/11/2012, 1♀, Brent E. Hendrixson, AUMNH]; [APH_1627, 15/11/2012, 1♂, Brent E. Hendrixson, AMNH]; [APH_1629, 15/11/2012, 1♂, Brent E. Hendrixson, AUMNH]; Madera Canyon, Madera Trailhead Picnic Area, 31.726945 -110.8804 ^1^, 4758ft., [APH_0618, 10/7/2009, 1 juv, Brent E. Hendrixson, Jon Davenport, Nate Davis, AUMNH]; Madera Canyon, Whitehouse Picnic Area, 31.733397 -110.88249 ^1^, 4640ft., [APH_1631, 15/11/2012, 1♂, Brent E. Hendrixson, AUMNH]; **Santa Cruz**: Madera Canyon, Bog Springs Campground, 31.72633 -110.87473 ^2^, 5088ft., [APH_1436, 11/11/2011, 1♂, June Olberding, AUMNH]; Madera Canyon, Mt. Wrightson picnic area, 31.71273 -110.874936 ^1^, 5401ft., [APH_1223, 5/8/2010, 1♀, Brent E. Hendrixson, Ashley Bailey, Andrea Reed, AUMNH]; [APH_1393, 5/10/2011, 1♀, Brent E. Hendrixson, Thomas Martin, AUMNH]; Madera Canyon, on road across from Santa Rita Gift Shop, 31.72527 -110.880051 ^1^, 4851ft., [APH_1594, 8/11/2012, 1♂, Brent E. Hendrixson, AUMNH]; Madera Canyon, picnic area, 31.72662 -110.879835 ^2^, 4886ft., [APH_1523, 10/9/2012, 1 juv, Brent E. Hendrixson, AUMNH]; Pajarito Mtns, 31.43263 -111.18977 ^6^, 4050ft., [APH_0136, unknown, 1♀, David Kandeyeli, AUMNH]; Patagonia, Pennsylvania Avenue, 31.54024 -110.758528 ^2^, 4046ft., [APH_1442, 18/12/2011, 1♂, Brent E. Hendrixson, Thomas Martin, AUMNH]; Santa Rita Mtns, along Mt. Hopkins Rd, 31.676321 -110.883409 ^1^, 6904ft., [APH_1197, 28/7/2010, 1 juv, Brent E. Hendrixson, Brendon Barnes, Nate Davis, AUMNH]; Upper Madera Canyon picnic area, 31.712488 -110.876839 ^1^, 5467ft., [APH_1342-1343, 4/8/2011, 2 juv, Brent E. Hendrixson, Brendon Barnes, Nate Davis, Jake Storms, AUMNH].

#### Distribution and natural history.

*Aphonopelma madera* is known from the Huachuca, Pajarito, and Santa Rita Mountains in southeastern Arizona at elevations ranging from 1230 to 2110 meters, inhabiting the Madrean Archipelago Level III Ecoregion. The species has been collected from riparian, oak-grassland, oak woodland, and pine-oak woodland communities (Figs 1C, 82). Like other sky island endemics (e.g., *Aphonopelma catalina* and *Aphonopelma chiricahua*), *Aphonopelma madera* is thought to be the only species found at higher elevations within these mountain ranges but might be syntopic with *Aphonopelma chalcodes* and *Aphonopelma vorhiesi* at lower elevations. Despite several field trips to the type locality to search for adults of this species, only a single burrow (of an adult female, APH_1393, Fig. 78) was ever observed. The burrow entrance was located along the base of a slope and had a thin layer of silk along its edges; the burrow itself was fairly shallow and was sheltered by a large rock that was removed to reveal the spider inside its retreat. All other adults (males and females) were found walking along canyon roads during daylight hours. All immature specimens were located underneath large rocks with no obvious burrow entrances. The breeding period for this species is similar to other high-elevation species in the region (late autumn, early winter). Adult males examined in this study were found during the months of November and December; unconfirmed adult males (no voucher specimens available, identification tentatively assigned based on a photograph and locality data) have been found in Madera Canyon in October (http://bugguide.net/node/view/154224/bgimage; http://bugguide.net/node/view/665106/bgpage).

#### Conservation status.

Of the three sky island endemics, *Aphonopelma madera* is represented by the most specimens, has the largest distribution, and is the only species that is not restricted to a single mountain range. The species appears to be fairly common in Madera Canyon but we would not argue that the conservation status for this species is secure at the moment. Our sampling of mitochondrial haplotypes (Hendrixson et al. 2015) indicates that each mountain range is genetically unique and should be further evaluated for the presence of evolutionary significant units for conservation purposes. Additional sampling throughout the region (e.g., Canelo Hills, Patagonia Mountains, northern Sonora) is required to gain a better assessment of the potential for gene flow between populations. These mountain ranges are fairly rugged and benefit from management by the federal government (Coronado National Forest, Nogales and Sierra Vista Ranger Districts, United States Army), but have also been subjected to habitat degradation from recent urban encroachment (e.g., Nogales, Sierra Vista, Tucson), human-caused forest fires, off-road driving, recreational activities, human immigrants, and illegal drug trafficking (Coronado Planning Partnership 2008). Climate change in the sky island region (Brusca et al. 2013, Mitchell and Ober 2013, Moore et al. 2013, Hendrixson et al. 2015) also poses a potential threat to the survival of *Aphonopelma madera*.

#### Remarks.

*Aphonopelma madera* is morphologically similar to other high-elevation species in the *Marxi* species group, especially *Aphonopelma catalina* (see Hendrixson et al. 2015). Other important ratios that distinguish males: *Aphonopelma madera* possess a larger PTl/M3 (≥0.94; 0.94–1.05) than *Aphonopelma peloncillo* (≤0.82; 0.71–0.82), *Aphonopelma vorhiesi* (≤0.87; 0.71–0.87), and *Aphonopelma chalcodes* (≤0.75; 0.67–0.75); by possessing a larger A3/M4 (≥0.54; 0.54–0.60) than *Aphonopelma catalina* (≤0.52; 0.47–0.52) and *Aphonopelma chalcodes* (≤0.50; 0.43–0.50), but smaller than *Aphonopelma chiricahua* (≥0.65; 0.65–0.72). Other important ratios that distinguish females: *Aphonopelma madera* possess a larger F1/T3 (≥1.74; 1.74–1.84) than *Aphonopelma catalina* (≤1.66; 1.63–1.66); by possessing a larger Cl/P1 (≥2.71; 2.71–3.01) than *Aphonopelma chiricahua* (2.21 ± (only 1 specimen)); by possessing a larger A3/T4 (≥0.60; 0.60–0.68) than *Aphonopelma peloncillo* (≤0.60; 0.57–0.60), but smaller than *Aphonopelma chiricahua* (0.71 ± (only 1 specimen)); by possessing a smaller L3 scopulation extent (48%–68%) than *Aphonopelma chalcodes* (78%–93%); by possessing a smaller L4 scopulation extent (29%–35%) than *Aphonopelma chalcodes* (63%–81%) and *Aphonopelma catalina* (37%–46%). For both males and females, certain morphometrics have potential to be useful, though due to the amounts of variation, small number of specimens, and the small differences between species, no other are claimed to be significant at this time (see Suppl. material 2). During evaluation of traditional PCA morphospace, males of *Aphonopelma madera* separate from *Aphonopelma chalcodes*, *Aphonopelma peloncillo*, and *Aphonopelma vorhiesi* along PC1~2, but do not separate from *Aphonopelma catalina* and *Aphonopelma chiricahua*. Females of *Aphonopelma madera* separate from *Aphonopelma chiricahua* and *Aphonopelma chalcodes* along PC1~2, but do not separate from *Aphonopelma catalina*, *Aphonopelma peloncillo*, and *Aphonopelma vorhiesi*. Interestingly, *Aphonopelma madera* males separate from *Aphonopelma chalcodes*, *Aphonopelma peloncillo*, and *Aphonopelma vorhiesi* in three-dimensional PCA morphospace (PC1~PC2~PC3), but do not separate from *Aphonopelma catalina*, *Aphonopelma chiricahua*, and *Aphonopelma marxi*. *Aphonopelma madera* females separate from *Aphonopelma chalcodes*, *Aphonopelma chiricahua*, and *Aphonopelma marxi*, but do not separate from *Aphonopelma catalina*, *Aphonopelma peloncillo*, and *Aphonopelma vorhiesi*. PC1, PC2, and PC3 explain ≥96% of the variation in all analyses. An important note on morphology, when viewing the *Aphonopelma madera* types and investigating the amount of variation across the measured specimens, one can see the large size differences possible between mature males and mature females of certain *Aphonopelma* species.

### Aphonopelma mareki

Taxon classificationAnimaliaAraneaeTheraphosidae

Hamilton, Hendrixson & Bond sp. n.

http://zoobank.org/22CB089B-6B44-47AC-BAE4-46894A10C02A

**Figure 83.**
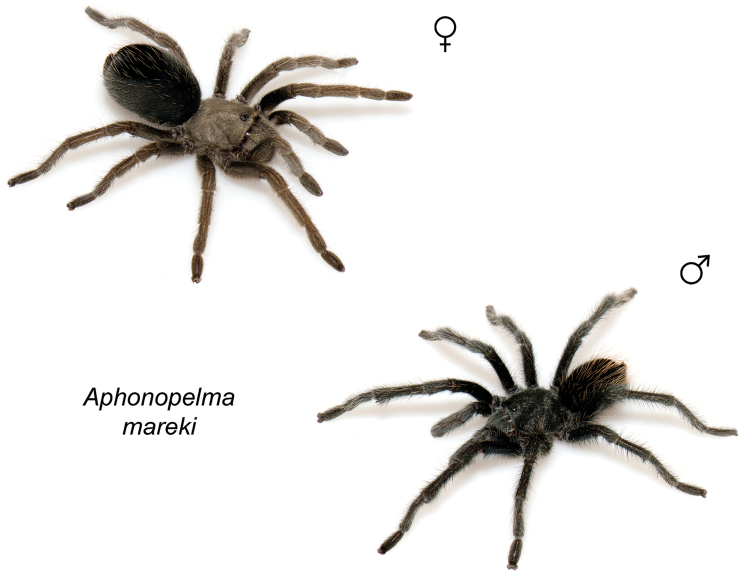
*Aphonopelma mareki* sp. n. live photographs. Female (L) - APH_1617; Male holotype (R) - APH_1615.

**Figure 84.**
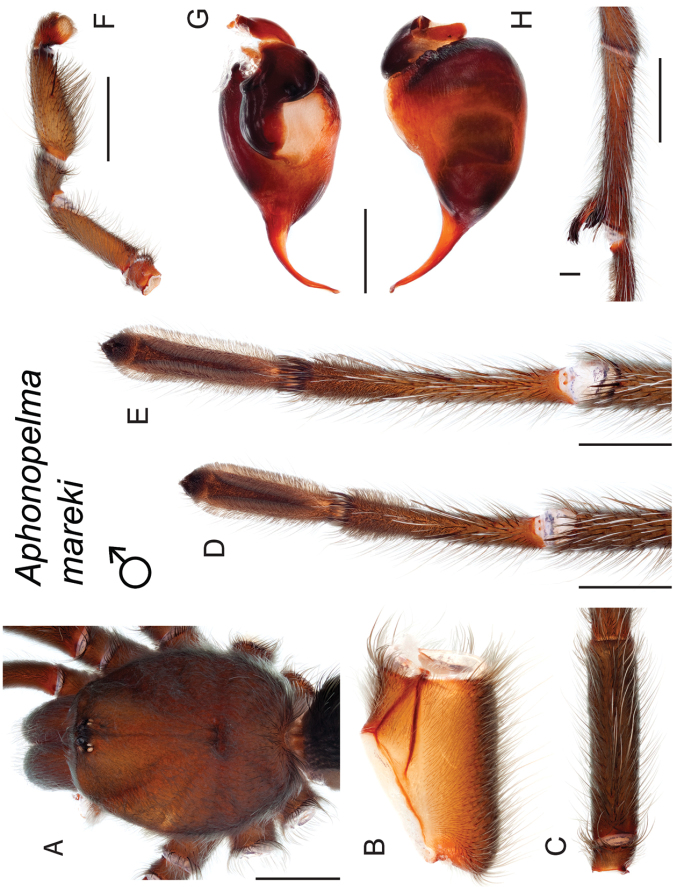
*Aphonopelma mareki* sp. n. **A–I** male holotype, APH_1615 **A** dorsal view of carapace, scale bar = 2.5mm **B** prolateral view of coxa I **C** dorsal view of femur III **D** ventral view of metatarsus III, scale bar = 2mm **E** ventral view of metatarsus IV, scale bar = 2mm **F** prolateral view of L pedipalp and palpal tibia, scale bar = 3mm **G** dorsal view of palpal bulb **H** retrolateral view of palpal bulb, scale bar = 0.5mm **I** prolateral view of tibia I (mating clasper), scale bar = 2.5mm.

**Figure 85.**
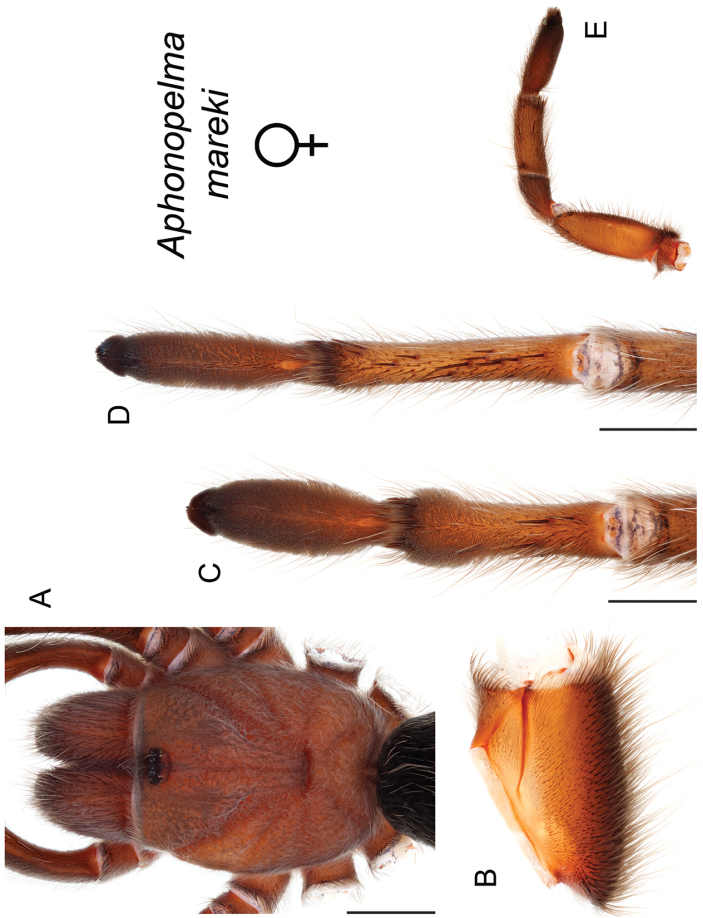
*Aphonopelma mareki* sp. n. **A–E** female paratype, APH_1590 **A** dorsal view of carapace, scale bar = 3mm **B** prolateral view of coxa I **C** ventral view of metatarsus III, scale bar = 1.5mm **D** ventral view of metatarsus IV, scale bar = 2mm **E** prolateral view of L pedipalp and palpal tibia.

**Figure 86.**
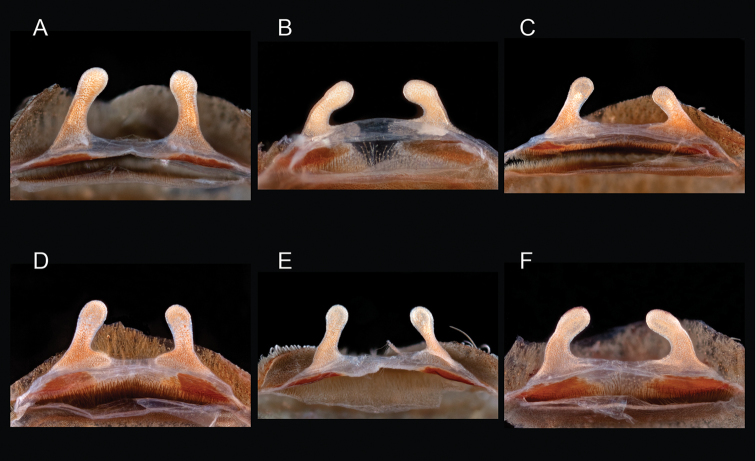
*Aphonopelma mareki* sp. n. **A–E** cleared spermathecae **A** APH_1590 **B** APH_0297 **C** APH_0941 **D** APH_1589 **E** APH_1617.

**Figure 87.**
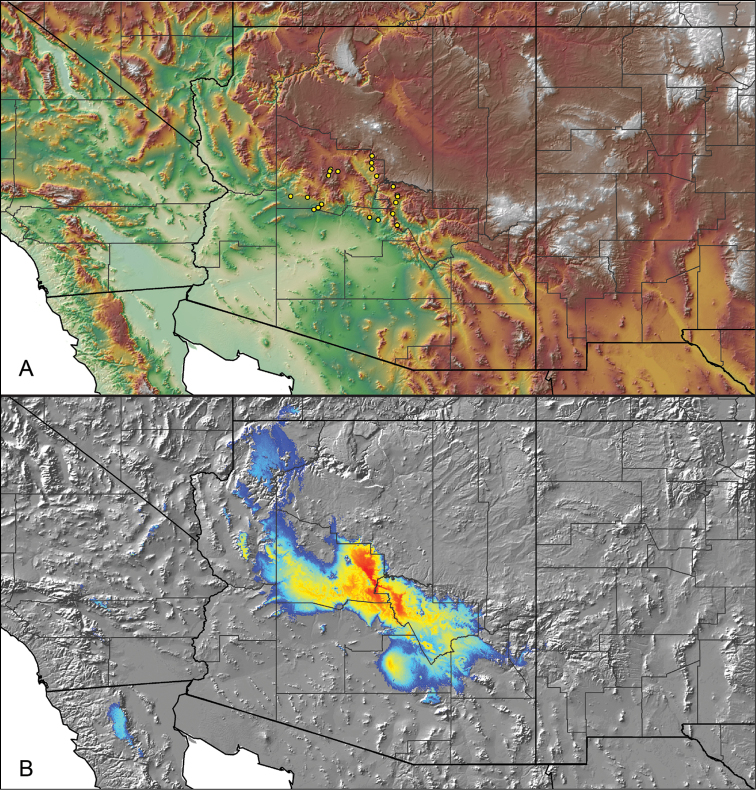
*Aphonopelma mareki* sp. n. **A** distribution of known specimens **B** predicted distribution; warmer colors (red, orange, yellow) represent areas of high probability of occurrence, cooler colors (blue shades) represent areas of low probability of occurrence.

Figures 83 , 84 , 85 , 86 , 87 ; Suppl. material 4

#### Types.

Male holotype (APH_1615) collected 0.4 miles E Hwy-89 on Stanton Rd, Yavapai Co., Arizona, 34.18203 -112.818862 ^1^, elev. 2852ft., 12.xi.2012, coll. Brent E. Hendrixson; deposited in AUMNH. Paratype female (APH_1590) from Bartlett Dam Rd, Maricopa Co., Arizona, 33.847258 -111.793291 ^1^, elev. 2793ft., 7.xi.2012, coll. Brent E. Hendrixson; deposited in AUMNH. Paratype male (APH_1569) from just outside N boundary of Montezuma Well National Monument, along Beaver Creek Rd, Yavapai Co., Arizona, 34.653811 -111.757497 ^1^, elev. 3596ft., 27.x.2012, coll. Brent E. Hendrixson; deposited in AMNH. Paratype female (APH_0297) from Mazatzal Mtns, Four Peaks, Pigeon Spring Rd. junction, Gila Co., Arizona, 33.72156 -111.33753 ^1^, elev. 5780ft., 6.x.2007, coll. Zach Valois, deposited in AMNH.

#### Etymology.

The specific epithet is a patronym in recognition of our friend, colleague, and myriapod biologist Paul Marek, who collected several important specimens examined as part of this revision.

#### Diagnosis.

*Aphonopelma mareki* (Fig. 83) is a member of the *paloma* species group and can be distinguished by a combination of morphological, molecular, and geographic characteristics. Nuclear DNA identifies *Aphonopelma mareki* as a strongly supported, phylogenetically-distinct monophyletic lineage (Fig. 8) that is a sister lineage to *Aphonopelma parvum* sp. n., *Aphonopelma saguaro* sp. n., and *Aphonopelma superstitionense* sp. n. *Aphonopelma mareki* can easily be differentiated from syntopic populations of *Aphonopelma chalcodes* and *Aphonopelma marxi* by their smaller size, and can be differentiated from other members of the *paloma* species group (*Aphonopelma parvum*, *Aphonopelma paloma*, *Aphonopelma prenticei* sp. n. and *Aphonopelma saguaro*) by locality. *Aphonopelma mareki* males can possess either a normal or slightly swollen femur III (different from than the swollen femur III in *Aphonopelma paloma* and *Aphonopelma saguaro*). The most significant measurements that distinguish male *Aphonopelma mareki* from its closely related phylogenetic and syntopic species are F1 and the extent of scopulation on metatarsus III. Male *Aphonopelma mareki* can be distinguished by possessing a larger F1/M1 (≥1.41; 1.41–1.58) than *Aphonopelma chalcodes* (≤1.36; 1.11–1.36) and *Aphonopelma prenticei* (≤1.34; 1.25–1.34), and smaller than *Aphonopelma marxi* (≥1.69; 1.69–1.94); a larger L3 scopulation extent (50%-56%) than *Aphonopelma paloma* (21%-41%), and smaller than *Aphonopelma chalcodes* (65%-86%), *Aphonopelma parvum* (60%-65%), *Aphonopelma prenticei* (60%-73%), and *Aphonopelma superstitionense* (40%-48%). There are no significant measurements that separate *Aphonopelma mareki* and *Aphonopelma saguaro*. The most significant measurements that distinguish female *Aphonopelma mareki* from its closely related phylogenetic and syntopic species are F1L/W and the extent of scopulation on metatarsus IV. Female *Aphonopelma mareki* can be distinguished by possessing a larger F1L/W (≥2.94; 2.94–3.17) than *Aphonopelma saguaro* (2.58 ± (only 1 specimen)); a smaller L4 scopulation extent (21%–27%) than *Aphonopelma chalcodes* (56%–81%), *Aphonopelma marxi* (33%–51%), *Aphonopelma parvum* (33%–42%), and *Aphonopelma prenticei* (27%–38%). There are no significant measurements that separate female *Aphonopelma mareki* from *Aphonopelma paloma* or *Aphonopelma superstitionense*.

#### Description of male holotype

(APH_1615; Fig. 84). *Specimen preparation and condition*: Specimen collected wandering and preserved in 80% ethanol; original coloration faded due to preservation. Left legs I, III, IV, and left pedipalp removed for measurements and photographs; stored in vial with specimen. Right legs III & IV removed for DNA and stored at -80°C in the AUMNH (Auburn, AL). *General coloration*: Faded black/brown. *Cephalothorax*: Carapace 6.394 mm long, 5.504 mm wide; densely clothed with faded pubescence, appressed to surface; fringe covered in longer setae not closely appressed to surface; foveal groove medium deep and straight; pars cephalica region rises very gradually from foveal groove on a straight plane towards the ocular area; AER very slightly procurved - mostly straight, PER very slightly recurved - mostly straight; normal sized chelicerae; clypeus extends forward on a slight curve; LBl 0.777, LBw 1.16; sternum hirsute, clothed with faded, densely packed, short setae. *Abdomen*: Densely clothed in short black/brown pubescence with numerous longer, lighter setae interspersed (generally red or orange *in situ*); dense dorsal patch of black Type I urticating bristles (Cooke et al. 1972) - smaller and distinct from large species. *Legs*: Hirsute; densely clothed in faded pubescence. Metatarsus I curved. F1 6.534; F1w 1.306; P1 2.406; T1 5.473; M1 4.137; A1 3.196; F3 5.046; F3w 1.451; P3 1.869; T3 4.008; M3 4.271; A3 3.358; F4 6.121; F4w 1.268; P4 1.928; T4 5.333; M4 5.494; A4 3.808; femur III is normal - not noticeably swollen or wider than other legs. All tarsi fully scopulate. Extent of metatarsal scopulation: leg III (SC3) = 54.1%; leg IV (SC4) = 42.1%. Three ventral spinose setae on metatarsus III; four ventral and four retrolateral spinose setae on metatarsus IV; two prolateral spinose setae and two ventral spinose setae on tibia I; one megaspine present on the retrolateral tibia, at the apex of the mating clasper; two megaspines on the apex on the retrolateral branch of the tibial apophyses, one megaspine on the apex of the prolateral branch, and one megaspine on the prolateral base of the prolateral branch of the tibial apophyses. *Coxa I*: Prolateral surface covered by fine, hair-like setae. *Pedipalps*: Hirsute; densely clothed in the same setal color as the other legs, with numerous longer ventral setae; one spinose seta at the apical, prolateral femur and two prolateral spinose setae on the palpal tibia; PTl 3.915, PTw 1.416. Palpal bulb is very short and stout. When extended, embolus tapers with a curve to the retrolateral side; embolus slender, no keels; distinct dorsal and ventral transition from bulb to embolus.

**Variation (5).** Cl 5.41–6.411 (5.981±0.19), Cw 4.751–5.844 (5.348±0.18), LBl 0.711–0.858 (0.78±0.02), LBw 0.857–1.16 (0.981±0.06), F1 6.017–6.875 (6.501±0.14), F1w 1.306–1.622 (1.449±0.05), P1 1.995–2.609 (2.382±0.1), T1 5.392–5.782 (5.62±0.08), M1 4.137–4.531 (4.352±0.07), A1 2.729–3.457 (3.183±0.13), L1 length 20.39–22.933 (22.039±0.47), F3 4.776–5.561 (5.248±0.15), F3w 1.451–1.875 (1.655±0.07), P3 1.726–1.975 (1.86±0.04), T3 3.732–4.434 (4.098±0.12), M3 4.271–5.129 (4.66±0.16), A3 3.092–3.607 (3.39±0.09), L3 length 17.686–20.706 (19.256±0.53), F4 5.849–6.639 (6.296±0.14), F4w 1.268–1.589 (1.399±0.06), P4 1.757–2.207 (2.022±0.08), T4 4.729–5.931 (5.475±0.21), M4 5.494–6.687 (6.158±0.24), A4 3.463–4.148 (3.858±0.12), L4 length 21.575–25.57 (23.808±0.74), PTl 3.542–4.044 (3.793±0.09), PTw 1.285–1.499 (1.392±0.03), SC3 ratio 0.502–0.563 (0.533±0.01), SC4 ratio 0.186–0.421 (0.286±0.04), Coxa I setae = very thin tapered, F3 condition = normal & slightly swollen.

#### Description of female paratype

(APH_1590; Figs 85–86). *Specimen preparation and condition*: Specimen collected live from burrow, preserved in 80% ethanol; original coloration faded due to preservation. Left legs I, III, IV, and pedipalp removed for photographs and measurements; stored in vial with specimen. Right legs III & IV removed for DNA and stored at -80°C in the AUMNH (Auburn, AL). Genital plate with spermathecae removed and cleared, stored in vial with specimen. *General coloration*: Faded black/brown. *Cephalothorax*: Carapace 7.744 mm long, 6.839 mm wide; Hirsute, densely clothed with short faded black/brown pubescence closely appressed to surface; fringe densely covered in slightly longer setae; foveal groove medium deep and straight; pars cephalica region gently rises from thoracic furrow, arching anteriorly toward ocular area; AER very slightly procurved, PER very slightly recurved; chelicerae robust, clypeus extends on a slight curve; LBl 1.153, LBw 1.364; sternum hirsute, clothed with short faded setae. *Abdomen*: Densely clothed dorsally in short faded black setae with longer, lighter setae (generally red or orange *in situ*) focused near the urticating patch; dense dorsal patch of black Type I urticating bristles (Cooke et al. 1972) - smaller and distinct from large species. *Spermathecae*: Paired and separate, with capitate bulbs, narrow necks widening towards the bases; not fused. *Legs*: Hirsute; densely clothed in short faded black/brown pubescence; F1 6.467; F1w 2.193; P1 3.075; T1 5.425; M1 4.04; A1 3.742; F3 5.576; F3w 1.855; P3 2.736; T3 3.906; M3 3.986; A3 3.504; F4 6.708; F4w 1.959; P4 2.786; T4 5.56; M4 5.788; A4 4.188. All tarsi fully scopulate. Extent of metatarsal scopulation: leg III (SC3) = 56.9%; leg IV (SC4) = 25.1%. Four ventral spinose setae and one prolateral spinose seta on metatarsus III; sixteen ventral spinose setae on metatarsus IV (though five are clustered near the row of spinose setae that line the margin with the tarsus). *Coxa I*: Prolateral surface covered by a mix of thin tapered/very thin tapered and fine, hair-like setae. *Pedipalps*: Densely clothed in the same setal color as the other legs; one spinose seta on the apical, prolateral femur, six prolateral (two at the apical, prolateral border with the tarsus) spinose setae and one ventral spinose seta on the tibia.

**Variation (6).** Cl 7.315–8.778 (7.717±0.22), Cw 6.021–7.405 (6.649±0.19), LBl 1.012–1.195 (1.116±0.03), LBw 1.171–1.364 (1.281±0.03), F1 5.83–7.175 (6.362±0.18), F1w 1.839–2.276 (2.067±0.07), P1 2.412–3.416 (2.8±0.16), T1 4.712–5.646 (5.159±0.14), M1 3.348–4.089 (3.684±0.13), A1 2.981–3.742 (3.318±0.11), L1 length 19.528–23.553 (21.323±0.62), F3 4.494–5.928 (5.185±0.21), F3w 1.669–2.058 (1.794±0.06), P3 1.856–2.736 (2.341±0.13), T3 3.194–4.041 (3.647±0.12), M3 3.336–4.525 (3.859±0.16), A3 3.127–3.606 (3.455±0.07), L3 length 16.007–20.751 (18.487±0.66), F4 5.886–7.124 (6.504±0.17), F4w 1.679–2.053 (1.842±0.06), P4 2.285–3.222 (2.617±0.14), T4 4.687–5.565 (5.247±0.14), M4 4.705–5.878 (5.463±0.18), A4 3.636–4.188 (3.958±0.09), L4 length 21.384–25.866 (23.789±0.64), SC3 ratio 0.429–0.617 (0.554±0.03), SC4 ratio 0.218–0.273 (0.241±0.01), Coxa I setae = thin tapered. Spermathecae variation can be seen in Figure 86.

#### Material examined.

**United States: Arizona: Coconino**: Schnebly Hill Road, Sedona, 34.86533 -111.753003 ^5^, 4328ft., [AUMS_2272, 15/5/1992, 1♂, unknown, AUMNH]; **Gila**: 1.1 miles NW AZ-87 on Barnhardt Trail Rd (FR 419), 34.09815 -111.36165 ^1^, 3200ft., [APH_0345, 10/5/2008, 1 juv, Brent E. Hendrixson, Zach Valois, Josh Richards, AUMNH]; 1.4 miles E US-87 on Control Rd, 34.35949 -111.40302 ^1^, 5430ft., [APH_0347, 10/5/2008, 1 juv, Brent E. Hendrixson, Zach Valois, Josh Richards, AUMNH]; Mazatzal Mtns, Four Peaks, Pigeon Spring Rd. junction, 33.72156 -111.33753 ^1^, 5780ft., [APH_0297, 6/10/2007, 1♀, Zach Valois, AMNH]; Payson, Payson rodeo grounds at southern edge of town, 34.2189 -111.33695 ^1^, 5000ft., [APH_0299, 6/10/2007, 1♀, Zach Valois, AUMNH]; S side of Payson, 34.194017 -111.330517 ^5^, 6000ft., [APH_0941, 2007, 1♀, Josh Richards, AUMNH]; **Maricopa**: 2.5 miles E North Cave Creek Rd on Bartlett Dam Rd, 33.84746 -111.79369 ^1^, 2700ft., [APH_0343, 9/5/2008, 1 juv, Brent E. Hendrixson, Zach Valois, Josh Richards, AUMNH]; [APH_0433-0440, 16/11/2008, 6♀, 2 juv, Brent E. Hendrixson, AUMNH]; Bartlett Dam Rd, 33.847258 -111.793291 ^1^, 2793ft., [APH_1570, 27/10/2012, 1♀, Brent E. Hendrixson, AUMNH]; [APH_1588-1593, 7/11/2012, 6♀, Brent E. Hendrixson, AUMNH]; Bartlett Dam Rd, past Cave Creek ranger station, N of Carefree, Tonto National Forest, 33.84689 -111.79397 ^1^, 2703ft., [APH_3161-3165, 11/11/2013, 4♀, 1♂, Chris A. Hamilton, Brent E. Hendrixson, Molly Taylor, AUMNH]; Bartlett Dam, Bartlett Dam road, 33.809017 -111.65097 ^5^, 1637ft., [APH_0884, 2007, 1♀, Josh Richards, AUMNH]; Constellation Road near Rodeo Grounds northeast of Wickenburg, 33.980107 -112.71139 ^5^, 2221ft., [APH_2554, 14/2/1982, 1♂, John Rowley, AMNH]; Four Peaks Area, 33.72099 -111.33759 ^1^, 5769ft., [APH_0138, 15/6/2007, 1 juv, Mike Dame, Zach Valois, AUMNH]; Mazatzal Mtns, Tonto National Forest, Mount Ord, 33.91131 -111.40861 ^1^, 6735ft., [APH_0190, 6/9/2007, 1♂, Lorenzo Prendini, Jeremy Huff, AUMNH]; **Yavapai**: 0.4 miles E Hwy-89 on Stanton Rd, 34.18203 -112.818862 ^1^, 2852ft., [APH_1615-1617, 12/11/2012, 1♂, 2♀, Brent E. Hendrixson, AUMNH]; 5.4 miles NE Hwy 93, 0.6 miles S of cattle guard in Hieroglyphic Mtns., Constellation, 34.008657 -112.646455 ^4^, 2900ft., [AUMS_2572, 1/4/1990, 1♂, T.R. Prentice, AUMNH]; 5.4 miles NE Hwy 93, Hieroglyphic Mtns., Constellation, 34.017615 -112.635409 ^4^, 3060ft., [AUMS_2577, 1/4/1990, 1♂, T.R. Prentice, AUMNH]; 8 miles S of Sedona, 34.753621 -111.761997 ^5^, 3895ft., [AUMS_2534, 29/10/1983, 1♂, B. and M.W. Sanderson, AUMNH]; Constellation, 34.065643 -112.581652 ^5^, 3404ft., [AUMS_2573, 24/11/1991, 1♂, T.R. Prentice, AUMNH]; [AUMS_2574, 10/11/1990, 1♂, T.R. Prentice, AUMNH]; [AUMS_2575, 17/11/unknown, 1♂, T.R. Prentice, AUMNH]; [AUMS_2551, 18/3/1995, 1♀, T.R. Prentice, AUMNH]; Constellation Rd., 5.4 miles NE of Wickenburg, 34.0145 -112.645736 ^4^, 2950ft., [AUMS_2570, 1/4/1990, 1♂, T.R. Prentice, AUMNH]; [AUMS_2578, 10/11/1990, 1♂, T.R. Prentice, AUMNH]; Joshua Forest Parkway, ~31 miles N of Wickenburg, 34.199956 -113.094405 ^5^, 2700ft., [AUMS_2565, 19/1/1992, 1♂, T.R. Prentice, AUMNH]; just outside N boundary of Montezuma Well National Monument, along Beaver Creek Rd, 34.653811 -111.757497 ^1^, 3596ft., [APH_1569, 27/10/2012, 1♂, Brent E. Hendrixson, AMNH]; Prescott, 34.540024 -112.468502 ^5^, 5394ft., [AUMS_2525, 1999, 1♀, Lee Trout, AUMNH]; Prescott Valley, 34.610024 -112.315721 ^5^, 5005ft., [AUMS_2248, 11/1998, 2♂, Denis Watson, AUMNH]; Prescott, Antelope Villa Circle, 34.647138 -112.434386 ^2^, 5042ft., [APH_1568, 12/10/2012, 1♂, Timothy Tilney, AUMNH]; Prescott, on rock next to Hazy Library at Embry-Riddle Aeronautical University, 34.615964 -112.448841 ^2^, 5170ft., [APH_1618, 9/11/2012, 1♂, Greg Rice, AUMNH]; West Clear Creek Wilderness, 34.53555 -111.67659 ^1^, 3600ft., [APH_1441, 27/10/2011, 1♂, Paul E. Marek, Charity Hall, AUMNH].

#### Distribution and natural history.

*Aphonopelma mareki* can be found inhabiting the Arizona/New Mexico Mountains and Sonoran Basin and Range Level III Ecoregions of central Arizona (Fig. 87). This species is syntopic with *Aphonopelma chalcodes* and *Aphonopelma marxi*, and flanks the distributions of *Aphonopelma prenticei* and *Aphonopelma superstitionense*. The breeding season, when mature males abandon their burrows in search of females, occurs during the fall (generally September–November).

#### Conservation status.

*Aphonopelma mareki* is very common throughout its distribution but can be difficult to find due to the cryptic nature of its burrows and small window of activity during the year. The species is secure.

#### Remarks.

Other important ratios that distinguish *Aphonopelma mareki* males: *Aphonopelma mareki* possess a larger T1/M1 (≥1.26; 1.26–1.33) than *Aphonopelma chalcodes* (≤1.13; 0.99–1.13) and *Aphonopelma prenticei* (≤1.16; 1.07–1.16), and smaller than *Aphonopelma marxi* (≥1.39; 1.39–1.52); a larger PTl/M1 (≥0.83; 0.83–0.95) than *Aphonopelma chalcodes* (≤0.79; 0.67–0.79), *Aphonopelma prenticei* (≤0.82; 0.72–0.82), and *Aphonopelma superstitionense* (≤0.82; 0.79–0.82); a larger T3/M3 (≥0.85; 0.85–0.94) than *Aphonopelma paloma* (≤0.85; 0.79–0.85) and *Aphonopelma prenticei* (≤0.85; 0.80–0.85); a smaller PTl/F1 (≤0.61; 0.53–0.61) than *Aphonopelma saguaro* (≥0.61; 0.61–0.64). Other important ratios that distinguish *Aphonopelma mareki* females: *Aphonopelma mareki* possess a larger F3L/W (≥2.66; 2.66–3.00) than *Aphonopelma saguaro* (2.41 ± (only 1 specimen)); a larger M3/M4 (≥0.66; 0.66–0.77) than *Aphonopelma superstitionense* (0.62 ± (only 1 specimen)); a smaller M4/A4 (≤1.48; 1.23–1.48) than *Aphonopelma chalcodes* (≥1.61; 1.61–1.98); a smaller F1/M3 (≤1.75; 1.58–1.75) than *Aphonopelma marxi* (≥1.76; 1.76–1.91). Certain morphometrics have potential to be useful, though due to the amounts of variation, small number of specimens, and the small differences between species, no other are claimed to be significant at this time (see Suppl. material 2). During evaluation of traditional two-dimensional PCA morphospace and three-dimensional PCA morphospace (PC1~PC2~PC3), males of *Aphonopelma mareki* separate from *Aphonopelma chalcodes* and *Aphonopelma marxi* along PC1~2, but do not separate from *Aphonopelma paloma*, *Aphonopelma parvum*, *Aphonopelma prenticei*, *Aphonopelma saguaro*, or *Aphonopelma superstitionense*. Male *Aphonopelma mareki* separate from all compared species except *Aphonopelma parvum* and *Aphonopelma saguaro* in three-dimensional morphospace. Female *Aphonopelma mareki* separate from *Aphonopelma chalcodes*, *Aphonopelma marxi*, and *Aphonopelma paloma*, but do not separate from *Aphonopelma parvum*, *Aphonopelma prenticei*, *Aphonopelma saguaro*, or *Aphonopelma superstitionense* in two-dimensional morphological space, yet when three-dimensional morphospace is plotted, *Aphonopelma mareki* separates from *Aphonopelma chalcodes*, *Aphonopelma marxi*, and *Aphonopelma parvum*, but not *Aphonopelma paloma*, *Aphonopelma prenticei*, *Aphonopelma saguaro* or *Aphonopelma superstitionense*. PC1, PC2, and PC3 explain ≥98% of the variation in all analyses.

Mitochondrial DNA (*CO1*) identifies *Aphonopelma mareki* as a polyphyletic group with some members grouping with the *Aphonopelma superstitionense* lineage (Fig. 7). Both species were previously identified as putative novel species (Hamilton et al. 2014). Nuclear DNA identifies what we feel is a more accurate evolutionary history of the *Aphonopelma mareki* lineage and highlights how *CO1* is not effective at accurately delimiting species boundaries within this group possibly due to mitochondrial introgression.

### Aphonopelma marxi

Taxon classificationAnimaliaAraneaeTheraphosidae

(Simon, 1891)

**Figure 88.**
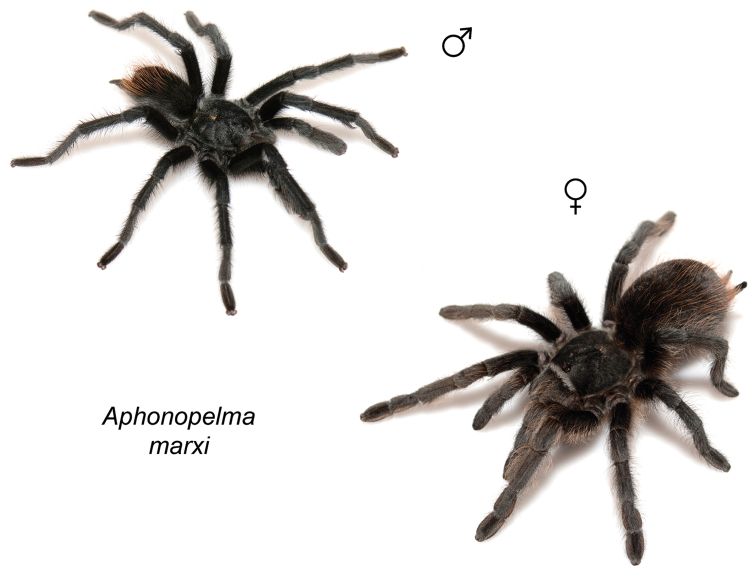
*Aphonopelma marxi* (Simon, 1891) specimens, live photographs. Male (L) - APH_0769; Female (R) - APH_1418.

**Figure 89.**
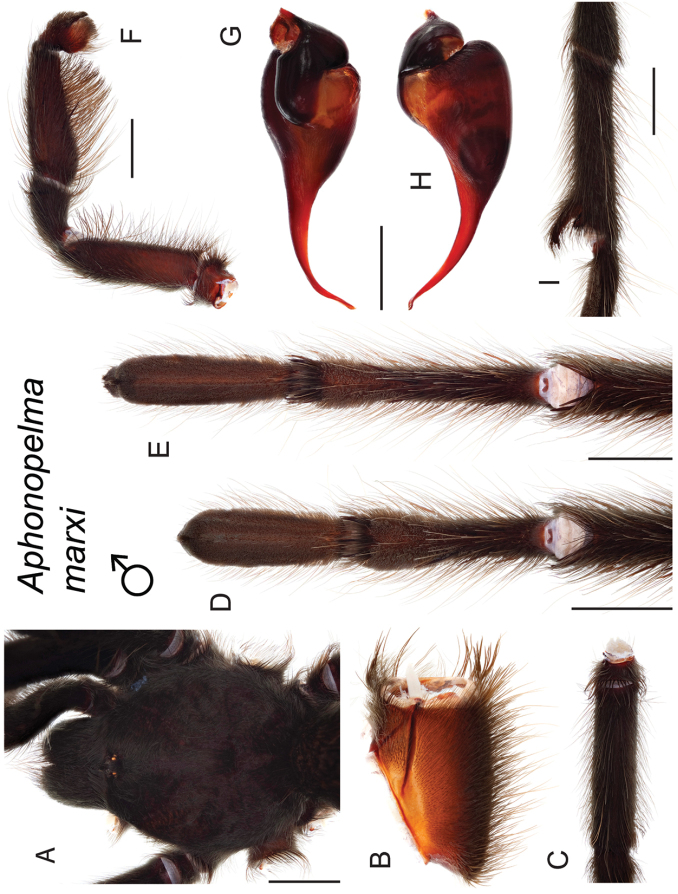
*Aphonopelma marxi* (Simon, 1891). **A–I** male specimen, APH_1396 **A** dorsal view of carapace, scale bar = 3.5mm **B** prolateral view of coxa I **C** dorsal view of femur III **D** ventral view of metatarsus III, scale bar = 3.5mm **E** ventral view of metatarsus IV, scale bar = 3mm **F** prolateral view of L pedipalp and palpal tibia, scale bar = 3mm **G** dorsal view of palpal bulb **H** retrolateral view of palpal bulb, scale bar = 1mm **I** prolateral view of tibia I (mating clasper), scale bar = 3.5mm.

**Figure 90.**
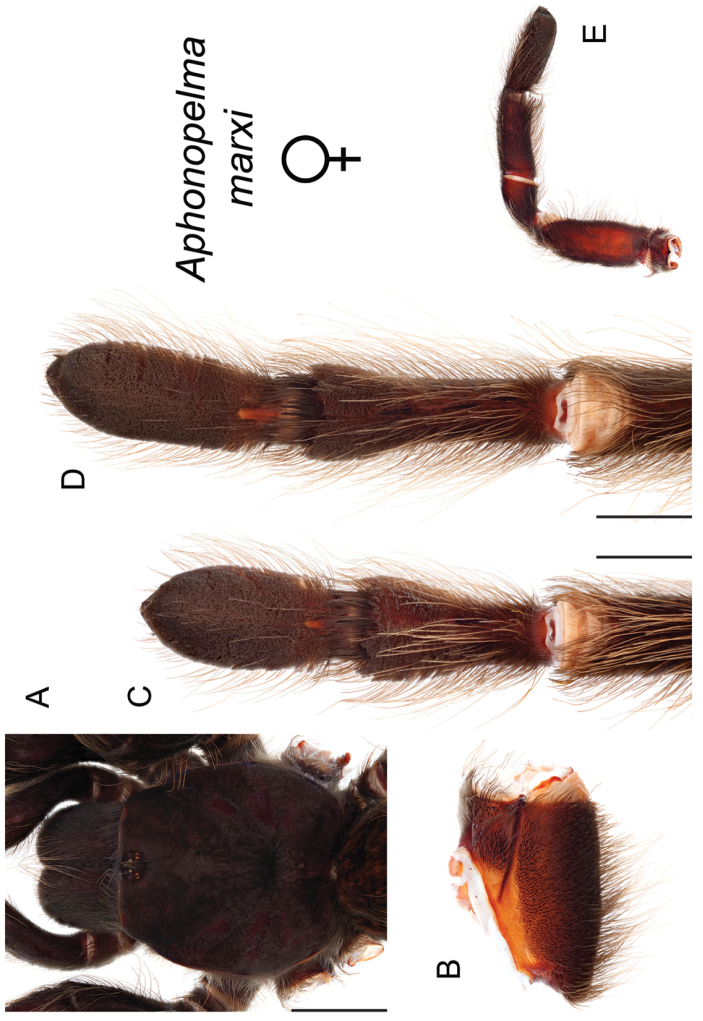
*Aphonopelma marxi* (Simon, 1891). **A–E** female specimen, APH_0452 **A** dorsal view of carapace, scale bar = 7mm **B** prolateral view of coxa I **C** ventral view of metatarsus III, scale bar = 3mm **D** ventral view of metatarsus IV, scale bar = 3mm **E** prolateral view of L pedipalp and palpal tibia.

**Figure 91.**
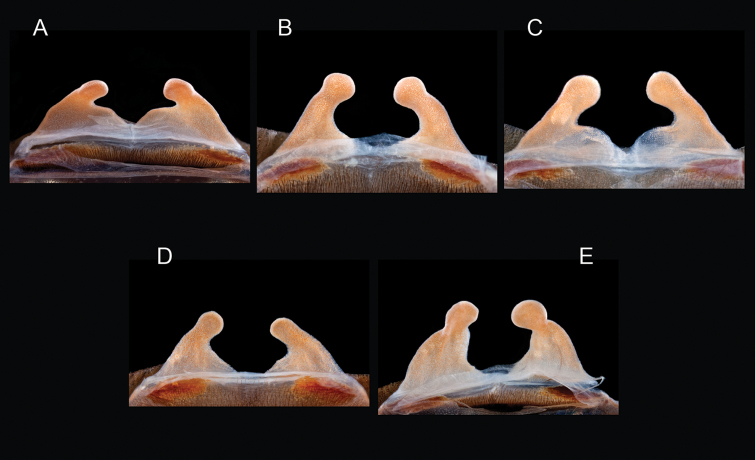
*Aphonopelma marxi* (Simon, 1891). **A–E** cleared spermathecae **A** APH_0452 **B** APH_1540 **C** APH_1541 **D** APH_2249 **E** APH_2266.

**Figure 92.**
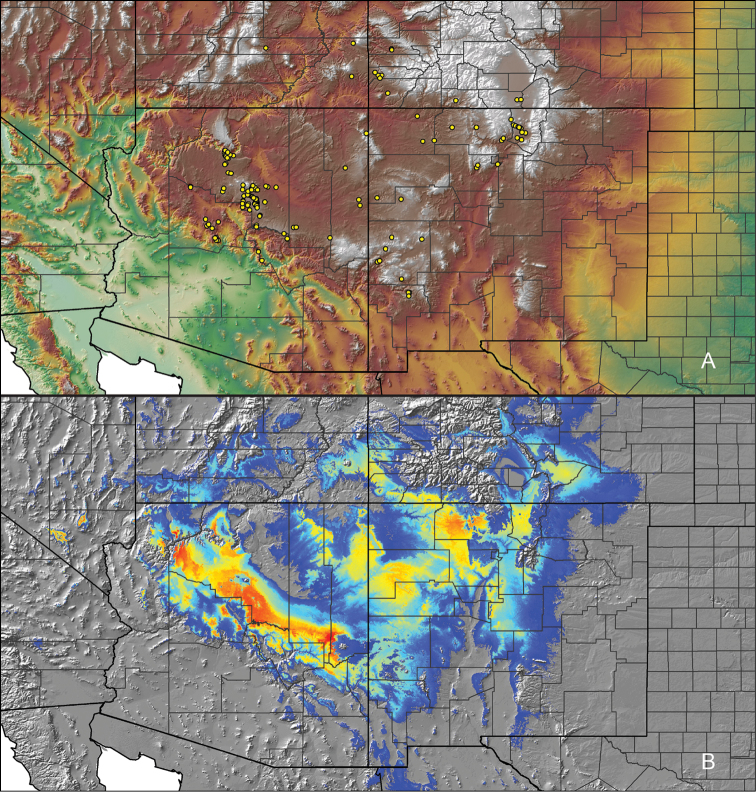
*Aphonopelma marxi* (Simon, 1891). **A** distribution of known specimens **B** predicted distribution; warmer colors (red, orange, yellow) represent areas of high probability of occurrence, cooler colors (blue shades) represent areas of low probability of occurrence.

Figures 88 , 89 , 90 , 91 , 92

Eurypelma marxi Simon, 1891: 324; no original labeled types known to exist; male neotype designated (Prentice 1997) from Punta del Aqua, Torrance Co., New Mexico, 34.600061 -106.283907^5^, elev. 6576ft., unknown collecting date, coll. Geo. Marx; deposited in NMNH. [not examined]
Delopelma marxi Petrunkevitch, 1939: 252.
Rhechostica marxi Raven, 1985: 149.
Aphonopelma marxi Smith, 1995: 119.
Aphonopelma marxi Prentice, 1997: 147.
Delopelma simulatum Chamberlin & Ivie, 1939: 8; male holotype from Fruita, Wayne Co., Utah, 38.285536 -111.246836^6^, elev. 5442ft., 14.vii.1931, coll. Gertsch and Johnson; deposited in AMNH. [examined]
Aphonopelma (Delopelma) simulatum Chamberlin, 1940: 26.
Rhechostica simulatum Raven, 1985: 149.
Aphonopelma simulatum Smith, 1995: 144. **previously synonymized by Prentice, 1997: 147.**
Aphonopelma behlei Chamberlin, 1940: 26; male holotype and male paratype from Grand Canyon Village, Coconino Co., Arizona, 36.054444 -112.140111^4^, elev. 6882ft., 15.ix.1939, coll. Dr. W.H. Behle; deposited in AMNH. [examined]
Rhechostica behlei Raven, 1985: 149.
Aphonopelma behlei Smith, 1995: 76. **syn. n.**
Aphonopelma vogeli Smith, 1995: 154; male holotype from Aztec, San Juan Co., New Mexico, 36.822226 -107.992846^6^, elev. 5657ft., 20.x.1982, coll. W.A. Drew; deposited in Oklahoma State University collection [presumed lost]
Aphonopelma vogelae - male neotype designated (APH_1431) from 0.4 miles E. CR-7635 along dirt road, San Juan Co., New Mexico, 36.28073 -107.8757^2^, elev. 6721ft., 4.xi.2011, coll. Amber Williams; deposited in AUMNH. [examined] **syn. n.**

#### Diagnosis.

*Aphonopelma marxi* (Fig. 88) is a member of the *Marxi* species group and can be identified by a combination of morphological, molecular, and geographic characteristics. Nuclear and mitochondrial DNA identifies *Aphonopelma marxi* as a strongly supported monophyletic lineage (Figs 7–8). *Aphonopelma marxi* is morphologically most similar to species in the Madrean sky island but has a different distribution. This species can easily be differentiated from syntopic species due to its black (or faded black) color, overall hirsute appearance, size, and habitat. The most significant measurement that distinguishes male *Aphonopelma marxi* from its closely related phylogenetic and syntopic species is M1. Male *Aphonopelma marxi* can be distinguished by possessing a larger F1/M1 (≥1.69; 1.69–1.94) than *Aphonopelma chalcodes* (≤1.36; 1.11–1.36), *Aphonopelma hentzi* (≤1.47; 1.32–1.47), *Aphonopelma mareki* sp. n. (≤1.58; 1.41–1.58), and *Aphonopelma vorhiesi* (≤1.63; 1.25–1.63). The most significant measurement that distinguishes female *Aphonopelma marxi* from its closely related phylogenetic and syntopic species is F1. Female *Aphonopelma marxi* can be distinguished by possessing a larger F1/M3 (≥1.76; 1.76–1.91) than *Aphonopelma chalcodes* (≤1.66; 1.41–1.66), *Aphonopelma hentzi* (≤1.61; 1.44–1.61), and *Aphonopelma mareki* (≤1.75; 1.58–1.75); and a larger F1/T4 (≥1.32; 1.32–1.43) than *Aphonopelma vorhiesi* (≤1.25; 1.17–1.25).

#### Description.

Male originally described by Simon (1891). No labeled types were known to exist. Two specimens from the Geo. Marx collection are now deposited in the USNM: a male non-type from the San Bernardino Mountains, California and a male non-type from Punta del Aqua, Torrance Co., New Mexico. Prentice (1997) examined the non-types and designated the New Mexico male as the neotype.

#### Redescription of male exemplar

(APH_1396; Fig. 89). *Specimen preparation and condition*: Specimen collected live crossing road, preserved in 80% ethanol; deposited in AUMNH; original coloration faded due to preservation. Left legs I, III, IV, and left pedipalp removed for measurements and photographs; stored in vial with specimen. Right leg III removed for DNA and stored at -80°C in the AUMNH (Auburn, AL). *General coloration*: Black and faded black. *Cephalothorax*: Carapace 9.50 mm long, 8.98 mm wide; Very hirsute; densely clothed with black, slightly iridescent, pubescence mostly appressed to surface; fringe covered in long setae not closely appressed to surface; foveal groove medium deep and straight; pars cephalica region rises gradually from foveal groove, gently arching anteriorly, reaches peak before ocular area; AER very slightly procurved - mostly straight, PER recurved; normal sized chelicerae; clypeus slightly extends forward on a curve; LBl 1.228, LBw 1.376; sternum very hirsute, clothed with black, densely packed setae. *Abdomen*: Densely clothed in short black pubescence with numerous longer red/orange setae interspersed; possessing a dense dorsal patch of black Type I urticating bristles (Cooke et al. 1972). *Legs*: Very hirsute, particularly ventrally; densely clothed in black or faded black pubescence. Metatarsus I straight. F1 10.683; F1w 2.563; P1 3.868; T1 8.649; M1 6.048; A1 4.776; F3 7.862; F3w 2.353; P3 3.401; T3 5.858; M3 6.311; A3 5.758; F4 9.913; F4w 2.349; P4 3.494; T4 8.04; M4 8.761; A4 6.426; femur III is normal - not noticeably swollen or wider than other legs. All tarsi fully scopulate. Extent of metatarsal scopulation: leg III (SC3) = 66.3%; leg IV (SC4) = 46.5%. Five ventral spinose setae on metatarsus III; eleven ventral spinose setae on metatarsus IV; one large megaspine is present on the retrolateral tibia at the apex of the mating clasper; one large megaspine at the apex of the retrolateral branch of the mating clasper. *Coxa I*: Prolateral surface a mix of fine, hair-like and thin tapered setae. *Pedipalps*: Very hirsute, particularly ventrally; densely clothed in the same setal color as the other legs; two spinose setae on the prolateral tibia; PTl 6.409, PTw 2.11. When extended, embolus tapers and gently curves to the retrolateral side.

**Variation (8).** Cl 8.32–10.53 (9.348±0.24), Cw 8.03–10.55 (8.854±0.28), LBl 1.087–1.397 (1.235±0.03), LBw 1.222–1.64 (1.446±0.05), F1 9.556–12.45 (10.532±0.36), F1w 1.957–2.89 (2.444±0.11), P1 3.546–4.81 (3.896±0.15), T1 7.366–10.21 (8.463±0.3), M1 4.912–7.33 (5.847±0.26), A1 4.61–6.05 (5.051±0.17), L1 length 30.157–40.85 (33.79±1.2), F3 7.184–9.23 (7.864±0.24), F3w 2.12–2.67 (2.315±0.06), P3 2.768–3.66 (3.179±0.11), T3 5.386–7.37 (5.985±0.22), M3 5.247–7.27 (6.183±0.23), A3 4.902–5.964 (5.421±0.14), L3 length 26.005–33.32 (28.632±0.86), F4 8.677–11.04 (9.536±0.28), F4w 2.04–2.79 (2.268±0.09), P4 2.943–4.11 (3.398±0.13), T4 7.058–9.39 (8.073±0.25), M4 7.548–9.74 (8.539±0.24), A4 5.573–6.80 (6.247±0.15), L4 length 32.388–41.08 (35.791±1), PTl 5.663–6.774 (6.152±0.13), PTw 1.94–2.49 (2.134±0.06), SC3 ratio 0.524–0.668 (0.605±0.02), SC4 ratio 0.38–0.465 (0.411±0.01), Coxa I setae = thin tapered, F3 condition = normal.

#### Description of female exemplar

(APH_0452; Figs 90–91). *Specimen preparation and condition*: Specimen collected live from burrow, preserved in 80% ethanol; deposited in AUMNH; original coloration faded due to preservation. Left legs I, III, IV, and pedipalp removed for photographs and measurements; stored in vial with specimen. Right leg III removed for DNA and stored at -80°C in the AUMNH (Auburn, AL). Genital plate with spermathecae removed and cleared, stored in vial with specimen. *General coloration*: Black/faded black. *Cephalothorax*: Carapace 15.30 mm long, 14.04 mm wide; Very hirsute, densely clothed with black/faded black, slightly iridescent, pubescence closely appressed to surface; fringe densely covered in longer setae; foveal groove medium deep and straight; pars cephalica region gently rises from thoracic furrow, arching anteriorly toward ocular area; AER very slightly procurved, PER very slightly recurved; robust chelicerae, clypeus straight; LBl 1.74, LBw 2.09; sternum very hirsute, clothed with long black/faded black setae. *Abdomen*: Densely clothed dorsally in short black setae with numerous longer, lighter setae interspersed (generally red or orange *in situ*); dense dorsal patch of black Type I urticating bristles (Cooke et al. 1972); ventral side with shorter black setae. *Spermathecae*: Paired and separate, short, tapering and curving medially towards capitate bulbs, with wide bases that are not fused. *Legs*: Very hirsute, particularly ventrally; densely clothed in medium and long black pubescence, with longer setae colored similarly as the long abdominal setae; F1 12.84; F1w 4.33; P1 6.29; T1 10.29; M1 6.88; A1 6.58; F3 10.16; F3w 3.56; P3 5.04; T3 7.18; M3 6.72; A3 6.54; F4 11.94; F4w 3.77; P4 5.21; T4 9.54; M4 9.66; A4 7.51. All tarsi fully scopulate. Extent of metatarsal scopulation: leg III (SC3) = 70.0%; leg IV (SC4) = 50.6%. Three ventral spinose setae on metatarsus III; seven ventral spinose setae on metatarsus IV. *Coxa I*: Prolateral surface a mix of fine, hair-like and tapered/medium tapered setae. *Pedipalps*: Densely clothed in the same setal color as the other legs; one spinose seta on the apical, prolateral femur and two spinose setae on the prolateral tibia.

**Variation (5).** Cl 13.49–15.30 (14.454±0.34), Cw 12.85–15.02 (13.714±0.38), LBl 1.49–2.27 (1.86±0.13), LBw 1.95–2.31 (2.148±0.07), F1 11.48–13.62 (12.498±0.36), F1w 3.68–4.47 (4.004±0.16), P1 5.24–6.76 (5.95±0.26), T1 9.17–10.91 (9.914±0.31), M1 6.22–7.23 (6.76±0.16), A1 5.58–7.13 (6.276±0.27), L1 length 37.69–44.70 (41.398±1.17), F3 8.87–10.83 (9.832±0.32), F3w 2.94–4.06 (3.39±0.2), P3 4.36–5.54 (4.912±0.19), T3 6.36–7.93 (6.958±0.28), M3 6.49–7.30 (6.818±0.14), A3 5.45–6.66 (6.182±0.23), L3 length 31.67–38.0 (34.702±1.04), F4 10.85–13.28 (11.936±0.4), F4w 3.36–3.81 (3.566±0.1), P4 4.35–5.50 (4.936±0.21), T4 8.64–9.54 (9.146±0.18), M4 8.71–10.69 (9.734±0.32), A4 6.18–7.61 (7.124±0.28), L4 length 38.73–46.55 (42.876±1.26), SC3 ratio 0.597–0.74 (0.674±0.02), SC4 ratio 0.339–0.51 (0.438±0.04), Coxa I setae = tapered/medium tapered. Spermathecae variation can be seen in Figure 91.

#### Material examined.

**United States: Arizona: Apache**: 0.75 miles NE Hwy-191 on Hwy-61, 34.908575 -109.227341 ^1^, 6251ft., [APH_1408, 9/10/2011, 1♂, Brent E. Hendrixson, Thomas Martin, AUMNH]; 9.5 miles N Hwy-61 on Hwy-191, 35.030934 -109.254585 ^1^, 6415ft., [APH_1423, 12/10/2011, 1 juv, Brent E. Hendrixson, Thomas Martin, AUMNH]; Ganada, 35.711279 -109.542049 ^5^, 6398ft., [APH_2576, 5/10/1962, 1♂, Charles Supplee, AMNH]; Navajo Indian Reservation, Chuska Mtns, Roof Butte, 2.8 miles on IR-68/road to Roof Butte lookout tower from IR-13, 36.45784722 -109.0942889 ^1^, 9304ft., [APH_0452, 30/8/2008, 1♀, Zach Valois, AUMNH]; **Coconino**: 1 mile north of Cosimo and east of Flagstaff, 35.237379 -111.465593 ^5^, 6348ft., [APH_2514, 1/11/1978, 1♂, B. and M.W. Sanderson, AMNH]; 5 miles south of Lindbergh Spt. And south of Flagstaff on 89A, 35.010414 -111.738008 ^5^, 5587ft., [APH_2520, 19/10/1977, 1♂, B. and M.W. Sanderson, AMNH]; FH-3 (Lake Mary Road) south of Flagstaff, 35.132647 -111.612736 ^5^, 6873ft., [APH_2516, 4/10/1977, 1♂, B. and M.W. Sanderson, AMNH]; Grand Canyon - river side, 36.091658 -112.131436 ^7^, 3668ft., [APH_2522, 25/10/1982, 1♂, V. Roth, AMNH]; Leupp Road (510) east of Flagstaff toward Doney Peak, 35.304918 -111.20856 ^5^, 5469ft., [APH_2519, 5/10/1977, 1♂, B. Sanderson, AMNH]; Northeast of Flagstaff about 1/4 mile east of Highway 89A on Leupp Road, 35.238083 -111.42633 ^5^, 6207ft., [APH_2530, 30/9/1977, 1♂, F.B. and M.W. Sanderson, AMNH]; Northeast of Flagstaff in pine-oak forest, 35.241395 -111.555125 ^6^, 6824ft., [APH_2515, 24/9/1977, 1♂, B. and M.W. Sanderson, AMNH]; [APH_2517, 10/10/1977, 1♂, B. and M.W. Sanderson, AMNH]; On road north of Mormon Lake, 34.997156 -111.453915 ^5^, 7175ft., [APH_2518, 9/10/1978, 1♂, B. and M.W. Sanderson, AMNH]; Schnebly Hill Road northeast of Sedona in pines north Highway 17, 34.914247 -111.640255 ^5^, 6506ft., [APH_2523, 21/10/1976, 1♂, M.W. Sanderson, AMNH]; 0.1 miles NW Mormon Lake Rd on FS Rd 240, 34.946366 -111.490839 ^1^, 7175ft., [APH_0764, 6/10/2009, 1♂, Brent E. Hendrixson, Thomas Martin, AUMNH]; 0.54 miles E Spruce St on Hwy-180, 35.604503 -112.040946 ^1^, 6126ft., [APH_0772, 7/10/2009, 1♂, Brent E. Hendrixson, Thomas Martin, AUMNH]; 0.63 miles W Spruce St on Hwy-180, 35.609818 -112.060954 ^1^, 6095ft., [APH_0773, 7/10/2009, 1♂, Brent E. Hendrixson, Thomas Martin, AUMNH]; 0.9 miles E Hwy-64/180 on FS Rd 320, 35.804115 -112.115103 ^1^, 6185ft., [APH_0769, 7/10/2009, 1♂, Brent E. Hendrixson, Thomas Martin, AUMNH]; 1.1 miles S FS Rd 320 on Hwy-64/180, 35.787285 -112.129924 ^1^, 6095ft., [APH_0770-0771, 7/10/2009, 2♂, Brent E. Hendrixson, Thomas Martin, AUMNH]; 1.5 miles S Mormon Lake Rd on Lake Mary Rd, 34.962886 -111.437062 ^1^, 7298ft., [APH_1403-1404, 9/10/2011, 2♂, Brent E. Hendrixson, Thomas Martin, AUMNH]; 1.55 miles N FR-234 on Lake Mary Rd, 34.673922 -111.380134 ^1^, 6893ft., [APH_1406, 9/10/2011, 1♂, Brent E. Hendrixson, Thomas Martin, AUMNH]; 1.66 miles W Lake Mary Rd on Mormon Lake Rd, 34.983545 -111.474974 ^1^, 7251ft., [APH_0765, 6/10/2009, 1♂, Brent E. Hendrixson, Thomas Martin, AUMNH]; 2.33 miles N Mormon Lake Rd on Lake Mary Rd, 35.014623 -111.451737 ^1^, 6910ft., [APH_0766-0767, 6/10/2009, 1♂, 1♀, Brent E. Hendrixson, Thomas Martin, AUMNH]; 3.5 miles north of Flagstaff on 89A, then 1 mile northeast on Land Fill road, 35.286659 -111.537408 ^5^, 6726ft., [APH_2556, 21/10/1977, 1♂, M.W. Sanderson, AMNH]; 5.4 miles N Mormon Lake Rd on Lake Mary Rd, 35.056801 -111.462549 ^1^, 6913ft., [APH_0768, 6/10/2009, 1♂, Brent E. Hendrixson, Thomas Martin, AUMNH]; 6 miles north of Flagstaff-east of Highway 89A, 35.286052 -111.534536 ^5^, 6739ft., [APH_2558, 4/10/1977, 1♂, B. and M.W. Sanderson, AMNH]; 8 miles north of Flagstaff on 89A, 35.270113 -111.545071 ^5^, 6732ft., [APH_2545, 5/11/1977, 1♂, B. and M.W. Sanderson, AMNH]; along FR-47 near Kaibab Lake, 35.276468 -112.156744 ^1^, 6885ft., [APH_1427, 15/10/2011, 1♂, Brent E. Hendrixson, Krissy E. Rehm, AUMNH]; along FS-126, 34.982005 -111.27115 ^2^, 6382ft., [APH_0750, 20/9/2009, 1♂, Tyler P. McKee, AUMNH]; Disc Cave, 35.246593 -111.746956 ^5^, 7690ft., [APH_2054, 1/11/1953, 1♂, R. deSaussure, AMNH]; E of junction of I-17 and Rocky Park Road, 34.808426 -111.598321 ^5^, 6463ft., [AUMS_2274, 12/1991, 1♀, Chuck Kristensen, AUMNH]; east side of Upper Lake Mary, 35.048834 -111.456544 ^5^, 6860ft., [APH_2563, 9/10/1978, 1♂, B. and M.W. Sanderson, AMNH]; Grand Canyon, 36.090161 -112.123683 ^7^, 3540ft., [APH_2592, 3/10/1935, 1♂, unknown, AMNH]; Grand Canyon National Park, South Rim Trail, 36.060569 -112.124309 ^1^, 6987ft., [APH_1425-1426, 14/10/2011, 1♂, 1♀, Brent E. Hendrixson, Krissy E. Rehm, AUMNH]; Grand Canyon Village, 36.054444 -112.140111 ^5^, 6880ft., [APH_2047, 15/9/1939, 1♂, W.H. Behle, AMNH]; Grand Canyon - Colorado river side, 36.090969 -112.128235 ^7^, 3675ft., [APH_2564, 23/10/1982, 1♂, V. Roth, AMNH]; Grand Canyon, S rim, 5.5 miles E of Hwy 64/180 off Hwy 64, 36.006329 -112.03996 ^5^, 7160ft., [AUMS_2270, 7/10/1995, 1♀, T.R. Prentice, AUMNH]; Grand Canyon, S. Rim - 7.7 E of Hwy 64/180 jct off Hwy 64, 35.993894 -112.02127 ^4^, 7370ft., [AUMS_2393, 6/10/1996, 1♂, T.R. Prentice, AUMNH]; Grand Canyon, S. Rim 5.7 E of Hwy 64/180 jct, 35.996859 -112.032781 ^5^, 7295ft., [AUMS_2291, 6/10/1996, 1♂, T.R. Prentice, AUMNH]; Highway 180, 0.1 miles E of marker 258, 35.59363 -112.000625 ^4^, 6280ft., [AUMS_2271, 17/10/1998, 1♂, T.R. Prentice, AUMNH]; Highway 180, 1.6 miles E of marker 258, 35.594031 -112.000933 ^5^, 6280ft., [AUMS_2269, 17/10/1998, 1♂, T.R. Prentice, AUMNH]; Hwy 64, 1.5 miles S of Hwy 64 and 180 junction, 36.050233 -112.067007 ^4^, 6033ft., [AUMS_3290, 18/10/1998, 1♀, T.R. Prentice, AUMNH]; just east of Lake Mary Rd on unnamed Forest Rd, 34.695292 -111.372356 ^1^, 6908ft., [APH_1405, 9/10/2011, 1♀, Brent E. Hendrixson, Thomas Martin, AUMNH]; Kaibab Lake, 0.5 miles W of Hwy 64, 0.75 miles N of I-40, 35.27411 -112.157105 ^4^, 6933ft., [AUMS_2275, 22/10/1999, 1♂, T.R. Prentice, AUMNH]; Kaibab National Forest, along Benham Trail, 35.203242 -112.179256 ^1^, 7307ft., [APH_1428, 15/10/2011, 1♂, Brent E. Hendrixson, Krissy E. Rehm, AUMNH]; Lake Mary Rd, along Lower Lake Mary, 35.086973 -111.533713 ^1^, 6773ft., [APH_1529, 4/10/2012, 1♂, Brent E. Hendrixson, AUMNH]; Lake Mary Rd, near Jct with CR-209, 34.976885 -111.446262 ^1^, 7126ft., [APH_1530, 4/10/2012, 1♂, Brent E. Hendrixson, AUMNH]; Lakeview Trail at Double Springs Campground, 34.940291 -111.493418 ^1^, 7213ft., [APH_0509-0510, 20/5/2009, 2 juv, Brent E. Hendrixson, Bernadette DeRussy, Sloan Click, AUMNH]; Leupp Road (510) west of Doney Park, 35.23797 -111.426361 ^5^, 6207ft., [APH_2562, 9/10/1977, 1♂, B. and M.W. Sanderson, AMNH]; [APH_2571, 9/10/1977, 1♂, B. Sanderson, AMNH]; Leupp Road east of Flagstaff, 4 miles west of turnoff to Leupp, 35.299775 -111.028763 ^5^, 4767ft., [APH_2569, 8/10/1977, 1♂, B. and M.W. Sanderson, AMNH]; Lower Lake Mary, Lake Mary Rd., 0.75-1 mile N of dividing dam, 35.094464 -111.542495 ^4^, 6840ft., [AUMS_2280, 8/10/1995, 1♀, T.R. Prentice, AUMNH]; Lower Lake Mary, Lake Mary Rd., S of dividing dam, 35.08059 -111.5309 ^6^, 6826ft., [AUMS_2282, 22/10/1995, 1♂, T.R. Prentice, AUMNH]; Mogollon Rim, along Hwy-260, E of Payson, 34.29877 -110.86005 ^5^, 7574ft., [APH_0171-0172, unknown, 2 juv, Brandon Anderson, AUMNH]; Munds Park, along Pinewood Blvd between Maverick Circle and Stallion Circle, 34.939587 -111.629052 ^1^, 6639ft., [APH_0763, 6/10/2009, 1♂, Brent E. Hendrixson, Thomas Martin, AUMNH]; NE Flagstaff in Ponderosa pine-oak forest, 35.33044 -111.52799 ^6^, 6801ft., [AUMS_3350, 5/10/1978, 1♂, Mary Lou Olivier, AUMNH]; near entrance to Lindbergh Spring on Highway 89A - a few miles south of Flagstaff, 35.108374 -111.722211 ^5^, 6919ft., [APH_2512, 28/9/1977, 1♂, B. and M.W. Sanderson, AMNH]; NE of Flagstaff in pine-oak forest, 35.254193 -111.566811 ^6^, 7005ft., [APH_2541, 4/11/1977, 1♂, Mary Lou Olivier, AMNH]; NE of Flagstaff on Leupp road between 89A and Lindsey Road, 35.318653 -111.250668 ^5^, 5607ft., [APH_2557, 4/10/1977, 1♂, B. and M.W. Sanderson, AMNH]; NE of Flagstaff on Lindsey Road off of Leupp Road, 35.241864 -111.553488 ^5^, 6811ft., [APH_2572, 28/9/1977, 1♂, Palmer Child, AMNH]; Oak Creek Canyon at Slide Rock, 34.944142 -111.752462 ^5^, 4944ft., [APH_2574, 7/10/1977, 1♂, B. and M.W. Sanderson, AMNH]; Oak Creek Canyon- 3 miles south of Pine Flat campground, 34.994615 -111.737614 ^5^, 5443ft., [APH_2573, 9/10/1977, 1♂, B. and M.W. Sanderson, AMNH]; off SR-260, unmarked forest road, 34.48289 -111.45219 ^1^, 7043ft., [APH_0302, 7/10/2007, 1♀, Zach Valois, AUMNH]; on FH-3, 0.5 miles south of Upper Lake Mary dam, 35.078386 -111.529309 ^5^, 6844ft., [APH_2560, 9/10/1978, 1♂, B. and M.W. Sanderson, AMNH]; on FH-3, 1/2 mile south of curve to Mormon Lake Village, 34.897979 -111.438392 ^5^, 7310ft., [APH_2546, 9/11/1978, 1♂, B. and M.W. Sanderson, AMNH]; on FH-3, 13.5 miles north of Highway 87, 34.858355 -111.437038 ^5^, 7392ft., [APH_2566, 31/10/1978, 1♂, B. and M.W. Sanderson, AMNH]; on FH-3, 4 miles north of Upper Lake Mary dam, 35.095012 -111.544621 ^5^, 6864ft., [APH_2537, 9/11/1978, 1♂, B. and M.W. Sanderson, AMNH]; on FH-3, 5 miles south of Upper Lake Mary dam, 35.06553 -111.506179 ^5^, 6844ft., [APH_2536, 9/11/1978, 1♂, B. and M.W. Sanderson, AMNH]; on FH-3, east of Upper Lake Mary, north of Mormon Lake Village, 35.022747 -111.452029 ^5^, 6909ft., [APH_2561, 12/11/1977, 1♂, B. and M.W. Sanderson, AMNH]; [APH_2570, 12/11/1977, 1♂, B. and M.W. Sanderson, AMNH]; on FH-3, near Upper Lake Mary, 35.032127 -111.453127 ^5^, 6890ft., [APH_2542, 9/11/1978, 1♂, B. and M.W. Sanderson, AMNH]; on road north of Flagstaff near Buffalo Park, 35.21067 -111.633677 ^5^, 7090ft., [APH_2568, 4/10/1977, 1♂, M.W. Sanderson, AMNH]; on road to Mormon Lake Village-west of FH-3, 34.901168 -111.444089 ^5^, 7234ft., [APH_2565, 9/10/1978, 1♂, B. and M.W. Sanderson, AMNH]; on road west of Mormon Lake, 34.937277 -111.48767 ^5^, 7198ft., [APH_2544, 9/11/1978, 1♂, B. and M.W. Sanderson, AMNH]; Ponderosa pine-oak forest northeast of Flagstaff, 35.291744 -111.487715 ^6^, 6598ft., [APH_2559, 10/10/1978, 1♂, B. and M.W. Sanderson, AMNH]; S Rim of Grand Canyon, 4.2 miles E of Hwy 180 and Ring Rd junction, 36.005413 -112.046599 ^4^, 7258ft., [AUMS_2288, 5/10/1996, 1♂, T.R. Prentice, AUMNH]; S rim of Grand Canyon, BLM road between Grandview and Moran Points, 35.975297 -111.94644 ^6^, 7124ft., [AUMS_2266, 21/9/1989, 1♂, T.R. Prentice, AUMNH]; Schnebly Hill Road, 34.867375 -111.746206 ^5^, 4495ft., [APH_2538, 27/11/1978, 1♂, B. and M.W. Sanderson, AMNH]; south of Flagstaff on FH-3 (Lake Mary Road), 35.137983 -111.644481 ^5^, 6969ft., [APH_2547, 4/11/1977, 1♂, B. and M.W. Sanderson, AMNH]; Tusayan - jct. rd. 302, 35.926944 -112.048164 ^5^, 6840ft., [AUMS_2392, 21/10/1999, 1♂, T.R. Prentice, AUMNH]; Tusayan, 0.7 miles E of Hwy 64/180 on FR302, 35.967188 -112.115484 ^4^, 6701ft., [AUMS_2279, 21/10/1999, 1♀, T.R. Prentice, AUMNH]; Tusayan, 2.1 miles E of Hwy 64/180 on FR302, 35.954709 -112.091883 ^4^, 6754ft., [AUMS_2283, 6/10/1996, 1 juv, T.R. Prentice, AUMNH]; Tusayan, 4.6 miles SE on Rd 302, 1 mile W off 302 on dirt road, 35.9315 -112.076389 ^4^, 6805ft., [AUMS_2618, 13/10/2001, 2♂, T.R. Prentice, AUMNH]; Tusayan, campsite 1/2 mile N, 35.980154 -112.121266 ^5^, 6660ft., [AUMS_2286, 6/10/1996, 1♀, T.R. Prentice, AUMNH]; Tusayan, campsite 5 miles E of town, 35.930788 -112.056647 ^4^, 6812ft., [AUMS_2284, 5/10/1996, 1♂, T.R. Prentice, AUMNH]; Tusayan, Hwy 180/64, 1.2 miles E of Tusayan on Rd. 302, 35.963569 -112.106672 ^4^, 6729ft., [AUMS_2292, 7/10/1995, 1♂, T.R. Prentice, AUMNH]; Upper Lake Mary, Lake Mary Road- 0.2 miles S of Marshall Lake Road, 35.090485 -111.53677 ^4^, 6842ft., [AUMS_2273, 22/10/1995, 1♀, T.R. Prentice, AUMNH]; [AUMS_2287, 22/10/1995, 1♀, T.R. Prentice, AUMNH]; Upper Lake Mary, Lake Mary Road- 0.25 miles S of Marshall Lake Road, 35.089915 -111.535904 ^4^, 6845ft., [AUMS_2277, 22/10/1995, 1♀, T.R. Prentice, AUMNH]; [AUMS_2290, 22/10/1995, 1♀, T.R. Prentice, AUMNH]; Upper Lake Mary, Lake Mary Road- 2 miles S of boat landing, 35.05728 -111.471341 ^4^, 6840ft., [AUMS_2276, 8/10/1995, 1♂, T.R. Prentice, AUMNH]; Upper Lake Mary, Lake Mary Road- directly across rd. L.M. boat landing, 35.072195 -111.522194 ^4^, 6847ft., [AUMS_2289, 22/10/1995, 1♂, T.R. Prentice, AUMNH]; Upper Lake Mary, Lake Mary Road, 0.5 miles S of northern boat landing, 35.067884 -111.513956 ^4^, 6851ft., [AUMS_3292, 8/10/1995, 1♂, T.R. Prentice, AUMNH]; Upper Lake Mary, Lake Mary Road, just S of northern L. M. boat landing, 35.070238 -111.520417 ^5^, 6843ft., [AUMS_2466, 8/10/1995, 1♂, T.R. Prentice, AUMNH]; Upper Lake Mary, S end of lake (Tusayan), 35.043271 -111.465698 ^5^, 6860ft., [AUMS_2659, 17/10/1998, 1♂, T.R. Prentice, AUMNH]; W. side of San Francisco Mountain (near Sugarloaf Mtn), 35.342816 -111.737946 ^6^, 8510ft., [APH_2049, 4/10/1970, 1♂, R.H. Russell, AMNH]; West of Mormon Lake on road, 34.915288 -111.470848 ^5^, 7123ft., [APH_2502, 9/10/1978, 1♂, W. S and BBS, AMNH]; **Gila**: along FR-512, 34.19 -110.7925 ^2^, 6600ft., [APH_1239-1241, 24/10/2010, 3♂, Tyler P. McKee, AUMNH]; [APH_1246-1247, 24/10/2010, 1♀, 1♂, Tyler P. McKee, AUMNH]; Mazatzal Mtns, Four Peaks, Pigeon Spring Rd. junction, 33.72156 -111.33753 ^1^, 5780ft., [APH_0296, 6/10/2007, 1 juv, Zach Valois, AUMNH]; Mount Ord, 33.908542 -111.406922 ^1^, 6864ft., [APH_1533, 4/10/2012, 1 juv, Brent E. Hendrixson, AUMNH]; Strawberry, 34.444717 -111.453017 ^6^, 7079ft., [APH_0878-0879, 2007, 2 juv, Josh Richards, AUMNH]; [APH_0882, 2007, 1♀, Josh Richards, AUMNH]; **Maricopa**: Mount Ord, 33.91722 -111.40972 ^5^, 6338ft., [APH_0137, unknown, 1 juv, David Kandeyeli, AUMNH]; **Navajo**: 0.55 miles W Hwy-260 on unmarked Forest Rd, 34.430555 -110.652986 ^1^, 6827ft., [APH_1407, 9/10/2011, 1♂, Brent E. Hendrixson, Thomas Martin, AUMNH]; 1.35 miles SW Apache County Line on Elmer’s Way, 34.212947 -109.869137 ^1^, 7020ft., [APH_1424, 12/10/2011, 1♂, Brent E. Hendrixson, Thomas Martin, AUMNH]; Heber, 34.435921 -110.599701 ^5^, 6575ft., [APH_2053, 18/10/1950, 1♂, G.M. Bradt, AMNH]; **Yavapai**: 1005 N Zuni Ln, Prescott, 34.55229 -112.26907 ^2^, 4738ft., [APH_0030, 23/10/2005, 1♂, Shane Yoder, AUMNH]; 2.1 miles of junction Copper Basin Rd./89a S along Hwy 89, 34.499381 -112.480383 ^4^, 5801ft., [AUMS_2522, 17/11/1987, 1♂, F.C. Baptista, AUMNH]; Bradshaw Mtns, 34.41407 -112.403407 ^6^, 7480ft., [AUMS_3294, unknown, 1♀, unknown, AUMNH]; Bradshaw Mtns, above Crown King, 34.223851 -112.319382 ^6^, 6074ft., [AUMS_2268, 22/9/1990, 1♂, T.R. Prentice, AUMNH]; Bradshaw Mtns, Coal Camp Spring, 34.152809 -112.273775 ^6^, 6192ft., [AUMS_2249, 19/9/1992, 1♂, T.R. Prentice, AUMNH]; Bradshaw Mtns, Lane Mtn. area, eastern slope - power line, 34.156773 -112.316443 ^6^, 6703ft., [AUMS_2389, 21/9/1990, 1♂, T.R. Prentice, AUMNH]; Crown King by Ranger Station, 34.210431 -112.336055 ^6^, 5721ft., [AUMS_2281, 25/10/1992, 1♀, Fred Jenson, AUMNH]; [AUMS_2634, 24/10/1992, 1♂, Fred Jenson, AUMNH]; Crown King ranger station, 34.206888 -112.34151 ^5^, 5885ft., [AUMS_3276, 19/10/1992, 1♀, Fred Jenson, AUMNH]; [AUMS_3287, 18/9/1992, 1♂, unknown, AUMNH]; Granite Basin Rd, near Prescott, 34.613078 -112.543108 ^2^, 5624ft., [APH_1608-1611, 12/10/2012, 4♂, Tim Cota, AUMNH]; Ranger Station, Bradshaw Mtns, 34.468075 -112.514445 ^7^, 5884ft., [AUMS_2207, 18/9/1992, 1♂, T.R. Prentice, AUMNH]; Seligman, 0.75 miles from I-40 BI on CR-5, 35.313373 -112.86772 ^1^, 5225ft., [APH_1429, 15/10/2011, 1♂, Brent E. Hendrixson, Krissy E. Rehm, AUMNH]; Seligman, along CR-5, 35.295749 -112.867876 ^1^, 5211ft., [APH_1544, 23/10/2012, 1♂, Brent E. Hendrixson, AUMNH]; **Colorado: Archuleta**: 6 miles S Hwy 160 on Cat Creek Rd, 37.15957 -107.16641 ^5^, 6953ft., [APH_0009, 8/10/2004, 1♂, Rich Fuller, AUMNH]; **Conejos**: along Hwy-142, 37.17546 -105.849954 ^1^, 7750ft., [APH_1526-1527, 2/10/2012, 2♂, Brent E. Hendrixson, AUMNH]; **Dolores**: 0.25 miles NW CR-N on Hwy-491 (Hwy-666), 37.700308 -108.836649 ^1^, 6673ft., [APH_1412, 10/10/2011, 1♂, Brent E. Hendrixson, Thomas Martin, AUMNH]; 0.6 miles E CR-15 on Rd N, 37.70015 -108.76392 ^2^, 7005ft., [APH_1238, 17/10/2010, 1♂, Bob De Groff, AUMNH]; 10 miles SE Dove Creek, 0.5 miles E CR 15 on Rd N, 37.69978 -108.76347 ^4^, 7028ft., [APH_0787, 8/10/2009, 1♂, Robert De Groff, AUMNH]; 11 miles SE Dove Creek, 37.639983 -108.80134 ^2^, 6744ft., [APH_1433, 4/11/2011, 1♂, Bob De Groff, AUMNH]; Dove Creek, 37.7661 -108.905939 ^5^, 6861ft., [AUMS_2632, 8/10/2002, 1♂, Dessie Watson and La Veta Martin, AUMNH]; [AUMS_2640, 28/10/2001, 1♂, Emullest, AUMNH]; [AUMS_2657, 4/10/2002, 1♂, E & R Summers, AUMNH]; [AUMS_2658, 30/9/2002, 1♀, E & J Crawford, AUMNH]; [AUMS_2661, 25/10/2001, 1♂, Jake Holley, AUMNH]; [AUMS_2662, unknown, 1♂, unknown, AUMNH]; [AUMS_2663, 7/10/2001, 1♂, unknown, AUMNH]; [AUMS_2665, 9/10/2002, 1♂, J. Fury, AUMNH]; [AUMS_2666, 28/10/2001, 1♂, Glenda Holley, AUMNH]; [AUMS_2656, 9/10/2002, 1♀, Jeff Martinez and Buddy Banks, AUMNH]; **Montezuma**: Town of Cortez, 0.4 miles N CR-G on Creek 22.6, 37.31811 -108.62973 ^2^, 5930ft., [APH_1237, 3/10/2010, 1♂, Bob De Groff, AUMNH]; **Montrose**: Nucla, 38.24624 -108.545593 ^5^, 5840ft., [APH_2604, 8/10/1964, 1♂, L. Anderson, AMNH]; Nucla, 5 miles N CO-97, 38.26991 -108.54767 ^5^, 5816ft., [APH_0008, 9/10/2004, 1♂, Peggy Case, AUMNH]; **New Mexico: Catron**: 0.4 miles E Hwy-180 on Hwy-12, 33.694408 -108.855012 ^1^, 6344ft., [APH_1396, 6/10/2011, 1♂, Brent E. Hendrixson, Thomas Martin, AUMNH]; 0.7 miles E Hwy-180 on Hwy-12, 33.69624 -108.849978 ^1^, 6312ft., [APH_1397, 6/10/2011, 1♂, Brent E. Hendrixson, Thomas Martin, AUMNH]; 11.4 miles N Hwy-12 on Hwy-32, 33.975757 -108.674004 ^1^, 7560ft., [APH_1399, 6/10/2011, 1♂, Brent E. Hendrixson, Thomas Martin, AUMNH]; 3.8 miles E Hwy-180 on Hwy-12, 33.717351 -108.803131 ^1^, 6086ft., [APH_1398, 6/10/2011, 1 juv, Brent E. Hendrixson, Thomas Martin, AUMNH]; 9.1 miles S Hwy-60 on Hwy-32, 34.222511 -108.541725 ^1^, 7299ft., [APH_1400, 6/10/2011, 1♂, Brent E. Hendrixson, Thomas Martin, AUMNH]; Swingle Canyon Rd, 34.179194 -107.896824 ^1^, 7639ft., [APH_1534, 5/10/2012, 1♂, Brent E. Hendrixson, AUMNH]; **Cibola**: El Morro National Monument, along Hwy-53, 35.043388 -108.343568 ^1^, 7182ft., [APH_1410, 9/10/2011, 1♂, Brent E. Hendrixson, Thomas Martin, AUMNH]; **Grant**: 3.6 miles E Hwy-15 on Hwy-35 (Ruins Vista Trail), 33.034239 -108.163433 ^1^, 6165ft., [APH_1401, 7/10/2011, 1 juv, Brent E. Hendrixson, Thomas Martin, AUMNH]; Gila National Forest, 33.32663 -108.334031 ^6^, 7802ft., [APH_2575, 20/8/1975, 1♂, Riectert, AMNH]; Meadow Creek, 33.034779 -108.184903 ^5^, 5928ft., [APH_2621, 16/10/1978, 1♂, MH♂, AMNH]; [APH_2634, 4/12/1977, 1♂, M.H. Muma, AMNH]; **Los Alamos**: 0.45 miles SE Bandelier National Monument entrance on Hwy-4, 35.791144 -106.267812 ^1^, 6614ft., [APH_1422, 11/10/2011, 1♂, Brent E. Hendrixson, Thomas Martin, AUMNH]; **McKinley**: Zuni, 0.44 miles W CR-8 on Hwy-53, 35.068318 -108.859058 ^1^, 6272ft., [APH_1409, 9/10/2011, 1♂, Brent E. Hendrixson, Thomas Martin, AUMNH]; **Rio Arriba**: 2.75 miles E CR-247 on Hwy-111/554, 36.349489 -106.146003 ^1^, 7122ft., [APH_1536, 6/10/2012, 1♂, Brent E. Hendrixson, AUMNH]; 2.8 miles S El Rito on Hwy-554, 36.304638 -106.183638 ^1^, 6741ft., [APH_1535, 6/10/2012, 1♂, Brent E. Hendrixson, AUMNH]; Chama Canyon Road, 8 miles west of Hwy 84, 36.585467 -106.725334 ^5^, 6821ft., [APH_2051, 6/10/1963, 1♂, William A. Shear, AMNH]; Valencia Canyon, Carson National Forest, 36.5850096 -107.2461544 ^5^, 6745ft., [APH_2050, 25/10/1963, 1♂, William A. Shear, AMNH]; [APH_2052, 26/10/1963, 1♂, William A. Shear, AMNH]; **San Juan**: 0.4 miles E CR-7635 along dirt road, 36.28073 -107.8757 ^2^, 6721ft., [APH_1431-1432, 4/11/2011, 2♂, Amber Williams, AUMNH]; along dirt road just W of Rio Arriba County line, 36.29967 -107.63205 ^2^, 7236ft., [APH_1430, 1/11/2011, 1♂, Amber Williams, AUMNH]; **Sandoval**: Jemez Springs, 35.768636 -106.692258 ^5^, 6204ft., [APH_2320, unknown, 1♀, 1♂, Gertsch, AMNH]; Santa Fe National Forest, River’s Bend Rec Area, off Hwy-4, 35.713267 -106.723601 ^1^, 5827ft., [APH_1524, 1/10/2012, 1♀, Brent E. Hendrixson, AUMNH]; [APH_1542, 7/10/2012, 1 juv, Brent E. Hendrixson, AUMNH]; **Taos**: 10.4 miles SE Hwy-285 on Hwy-64, 36.582832 -105.798461 ^1^, 7388ft., [APH_1417, 11/10/2011, 1♀, Brent E. Hendrixson, Thomas Martin, AUMNH]; 2.4 miles SE Hwy-285 on Hwy-64, 36.632836 -105.927053 ^1^, 7942ft., [APH_1413, 11/10/2011, 1♂, Brent E. Hendrixson, Thomas Martin, AUMNH]; 2.87 miles E Hwy-285 on Hwy-567, 36.361557 -105.846664 ^1^, 7057ft., [APH_1537, 6/10/2012, 1♂, Brent E. Hendrixson, AUMNH]; 3.7 miles E Rio Grande Gorge on Hwy-64, 36.465284 -105.66766 ^1^, 7087ft., [APH_1421, 11/10/2011, 1♂, Brent E. Hendrixson, Thomas Martin, AUMNH]; 5.2 miles SE Hwy-285 on Hwy-64, 36.615257 -105.881934 ^1^, 7708ft., [APH_1414, 11/10/2011, 1♂, Brent E. Hendrixson, Thomas Martin, AUMNH]; 7.5 miles N Hwy-64 on Hwy-285, 36.75308 -105.982277 ^1^, 8229ft., [APH_1525, 2/10/2012, 1♂, Brent E. Hendrixson, AUMNH]; 8.6 miles SE Hwy-285 on Hwy-64, 36.594161 -105.827864 ^1^, 7502ft., [APH_1415-1416, 11/10/2011, 2♂, Brent E. Hendrixson, Thomas Martin, AUMNH]; along Hwy-64, 36.479505 -105.748914 ^1^, 6906ft., [APH_1539-1541, 6/10/2012, 3♀, Brent E. Hendrixson, AUMNH]; along Hwy-64, Taos Plateau Access Road, 36.561091 -105.769216 ^1^, 7356ft., [APH_1528, 2/10/2012, 1♀, Brent E. Hendrixson, AUMNH]; Hwy-570 at the bottom of the Rio Grande Gorge, 36.319072 -105.755713 ^1^, 6388ft., [APH_1538, 6/10/2012, 1♂, Brent E. Hendrixson, AUMNH]; near jct. Sheep Herder Rd and Upper Rim Rd (0.9 miles W Rio Grande Gorge on Hwy-64), 36.480253 -105.747771 ^1^, 7031ft., [APH_1418-1420, 11/10/2011, 1♀, 2♂, Brent E. Hendrixson, Thomas Martin, AUMNH]; **Utah: San Juan**: along Mustang Rd, near Blanding, 37.676633 -109.403537 ^1^, 6478ft., [APH_1411, 10/10/2011, 1♂, Brent E. Hendrixson, Thomas Martin, AUMNH]; Black Ridge area, south of Moab, 38.389 -109.372 ^6^, 6706ft., [APH_1444, 2/9/2011, 1♂, Corina Smith, AUMNH].

#### Distribution and natural history.

*Aphonopelma marxi* has a wide distribution across higher elevation areas of northern Arizona (Fig. 1B), northwestern New Mexico, southwestern Colorado, and southeastern Utah (Fig. 92) in the following Level III Ecoregions: Arizona/New Mexico Plateau, Arizona/New Mexico Mountains, Colorado Plateaus, and Southern Rockies. These tarantulas have been found in a variety of habitats including mixed conifer forests and sagebrush steppe. *Aphonopelma marxi* can be found syntopic with *Aphonopelma chalcodes* and *Aphonopelma mareki*. The breeding season, when mature males abandon their burrows in search of females, occurs during the fall (generally September–November). Additional details about the natural history of this species (discussed under the section on *Aphonopelma behlei*) are provided by Smith (1995).

#### Conservation status.

*Aphonopelma marxi* has a wide distribution across higher elevation habitats centered on the Four Corners region of Arizona, Colorado, New Mexico, and Utah. Some populations appear to be restricted to isolated mountains (e.g., Mount Ord) but are otherwise very common (but can be quite difficult to find due to the cryptic nature of their burrows). The species is likely secure.

#### Remarks.

Because no labeled types existed, Prentice (1997) examined additional material at the NMNH and designated a neotype, a New Mexico male, based on measurements matching Simon’s (1891) original description. We use this designation as the basis for our definition of *Aphonopelma marxi*. Efforts to locate the holotype of *Aphonopelma vogelae* (from Aztec, New Mexico) in the Oklahoma State University collection were unsuccessful and the specimen is presumed lost (Richard Grantham 2013, pers. comm.); the identity of this species, however, is not in question so we have designated a neotype from northwestern New Mexico (see above; deposited in AUMNH). We examined the holotype and freshly collected topotypic material of *Aphonopelma behlei* as well as the neotype of *Aphonopelma vogelae*. Our morphological and molecular analyses fail to recognize these two species as separate, independently evolving lineages. As a consequence, we consider *Aphonopelma behlei* and *Aphonopelma vogelae* junior synonyms of *Aphonopelma marxi*.

Other important ratios that distinguish males: *Aphonopelma marxi* possess a larger T1/M1 (≥1.39; 1.39–1.52) than *Aphonopelma chalcodes* (≤1.13; 0.99–1.13), *Aphonopelma hentzi* (≤1.24; 1.08–1.24), *Aphonopelma mareki* (≤1.33; 1.26–1.33), *Aphonopelma vorhiesi* (≤1.36; 1.09–1.36); by possessing a larger PTl/M3 (≥0.92; 0.92–1.16) than *Aphonopelma chalcodes* (≤0.76; 0.65–0.76), *Aphonopelma hentzi* (≤0.81; 0.71–0.81), *Aphonopelma mareki* (≤0.92; 0.76–0.92), and *Aphonopelma vorhiesi* (≤0.87; 0.71–0.87). Other important ratios that distinguish females: *Aphonopelma marxi* possess a larger L1/L3 (≥1.17; 1.17–1.21) than *Aphonopelma hentzi* (≤1.15; 1.07–1.15); by possessing a larger T1/T4 (≥1.06; 1.06–1.14) than *Aphonopelma hentzi* (≤1.05; 0.85–1.05), *Aphonopelma mareki* (≤1.01; 0.94–1.01), and *Aphonopelma vorhiesi* (≤0.98; 0.93–0.98); by possessing a larger L4 scopulation extent (33%-51%) than *Aphonopelma mareki* (21%-27%) and smaller than *Aphonopelma chalcodes* (56%-81%). For both males and females, certain morphometrics have potential to be useful, though due to the amounts of variation, small number of specimens, and the small differences between species, no others are claimed to be significant at this time (see Suppl. material 2). During evaluation of traditional PCA morphospace, males of *Aphonopelma marxi* separate in PCA morphological space from *Aphonopelma chalcodes*, *Aphonopelma hentzi*, *Aphonopelma mareki*, *Aphonopelma peloncillo* sp. n., and *Aphonopelma vorhiesi* but do not separate from the sky island species *Aphonopelma catalina* sp. n., *Aphonopelma chiricahua* sp. n., and *Aphonopelma madera* sp. n.. Female *Aphonopelma marxi* separate from *Aphonopelma chalcodes*, *Aphonopelma chiricahua*, and *Aphonopelma mareki* in morphological space, but do not separate from *Aphonopelma catalina*, *Aphonopelma madera*, *Aphonopelma hentzi*, *Aphonopelma peloncillo*, and *Aphonopelma vorhiesi*. Interestingly, *Aphonopelma marxi* males separate from *Aphonopelma catalina*, *Aphonopelma chalcodes*, *Aphonopelma hentzi*, *Aphonopelma peloncillo*, and *Aphonopelma vorhiesi* in three-dimensional PCA morphospace (PC1~PC2~PC3), but do not separate from *Aphonopelma chiricahua* or *Aphonopelma madera*. *Aphonopelma marxi* females separate from *Aphonopelma catalina*, *Aphonopelma chalcodes*, *Aphonopelma chiricahua*, *Aphonopelma hentzi*, *Aphonopelma mareki*, *Aphonopelma peloncillo*, but do not separate from *Aphonopelma madera* or *Aphonopelma vorhiesi*. PC1, PC2, and PC3 explain ≥96% of the variation in all analyses.

### Aphonopelma moderatum

Taxon classificationAnimaliaAraneaeTheraphosidae

(Chamberlin & Ivie, 1939)

**Figure 93.**
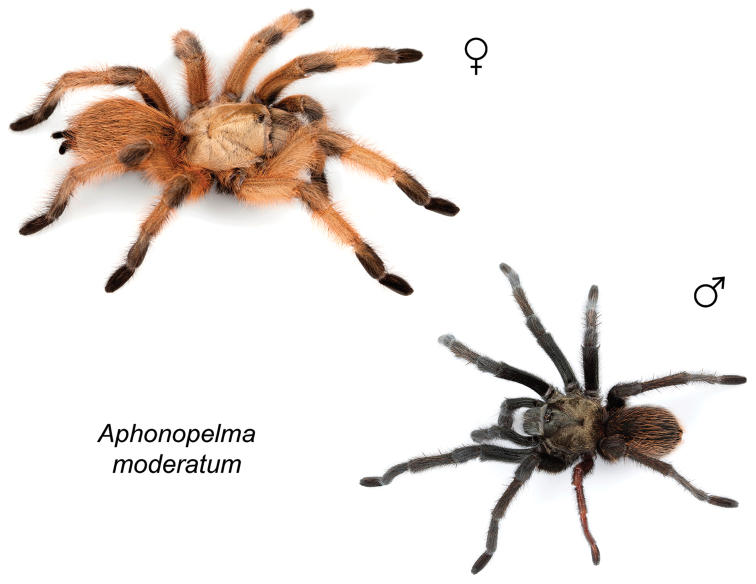
*Aphonopelma moderatum* (Chamberlin & Ivie, 1939) specimens, live photographs. Female (L) - APH_0532; Male (R) - APH_0890.

**Figure 94.**
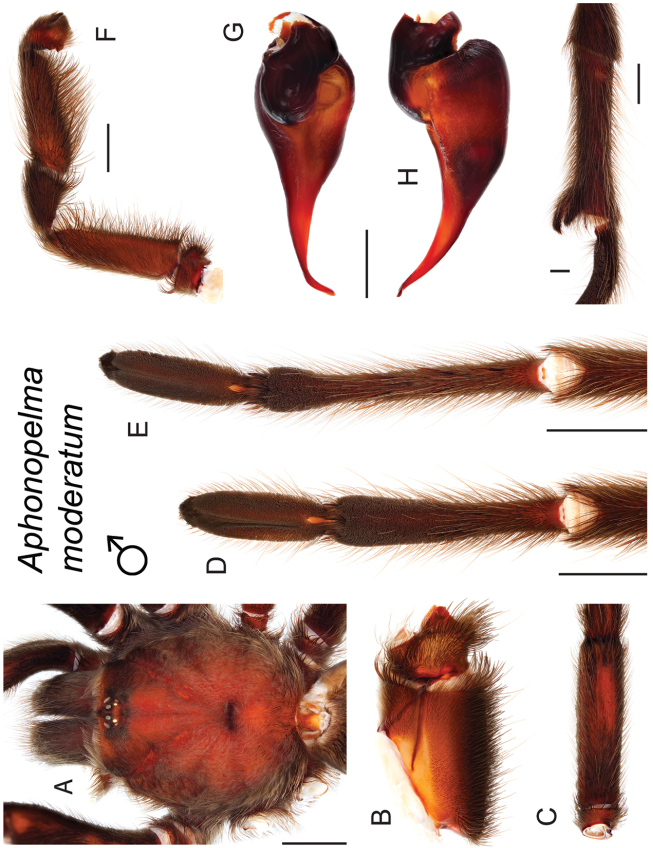
*Aphonopelma moderatum* (Chamberlin & Ivie, 1939). **A–I** male specimen, APH_0890 **A** dorsal view of carapace, scale bar = 5mm **B** prolateral view of coxa I **C** dorsal view of femur III **D** ventral view of metatarsus III, scale bar = 4.5mm **E** ventral view of metatarsus IV, scale bar = 5.5mm **F** prolateral view of L pedipalp and palpal tibia, scale bar = 3mm **G** dorsal view of palpal bulb **H** retrolateral view of palpal bulb, scale bar = 1mm **I** prolateral view of tibia I (mating clasper), scale bar = 3.5mm.

**Figure 95.**
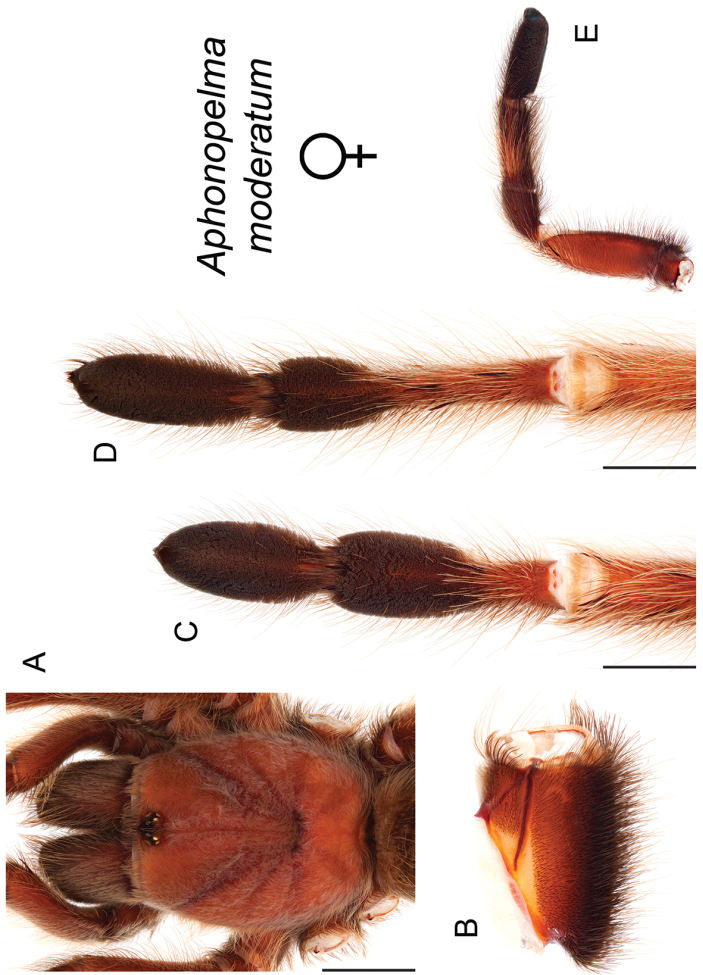
*Aphonopelma moderatum* (Chamberlin & Ivie, 1939). **A–E** female specimen, APH_0532 **A** dorsal view of carapace, scale bar = 6.5mm **B** prolateral view of coxa I **C** ventral view of metatarsus III, scale bar = 3.5mm **D** ventral view of metatarsus IV, scale bar = 3.5mm **E** prolateral view of L pedipalp and palpal tibia.

**Figure 96.**
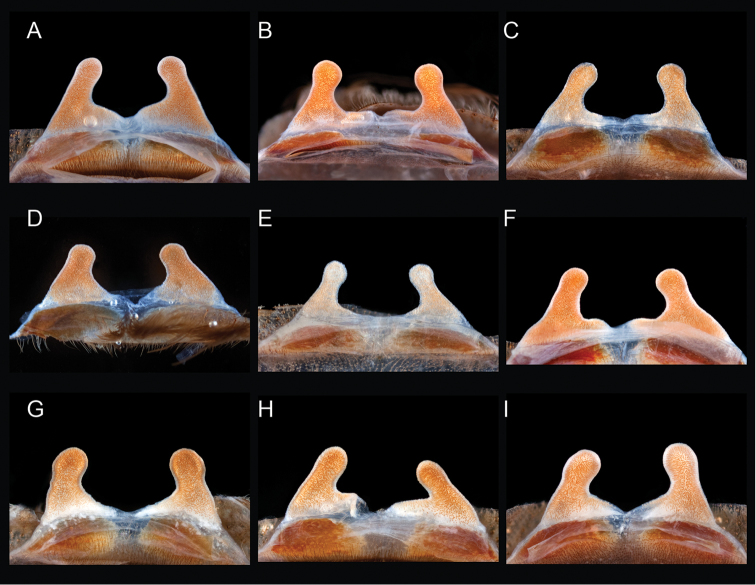
*Aphonopelma moderatum* (Chamberlin & Ivie, 1939). **A–I** cleared spermathecae **A** APH_0057 **B** APH_0532 **C** APH_0877 **D** APH_0891 **E** APH_0892 **F** APH_0894 **G** APH_1123 **H** APH_1136 **I** APH_1283.

**Figure 97.**
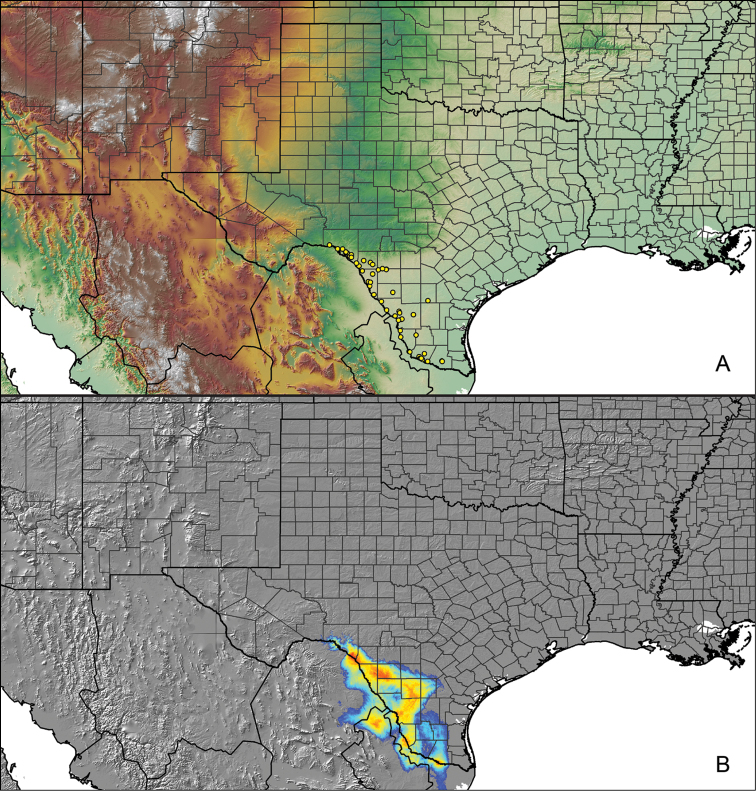
*Aphonopelma moderatum* (Chamberlin & Ivie, 1939). **A** distribution of known specimens **B** predicted distribution; warmer colors (red, orange, yellow) represent areas of high probability of occurrence, cooler colors (blue shades) represent areas of low probability of occurrence.

Figures 93 , 94 , 95 , 96 , 97 ; Suppl. material 4

Delopelma moderatum Chamberlin & Ivie, 1939: 9; male holotype from 5 miles E of Rio Grande City, Starr Co., Texas, 26.342436 -98.728749^5^, elev. 231ft., 1.v.1937, coll. S. Mulaik; deposited in AMNH. Paratype male from 32 miles SW of Laredo, Webb Co., Texas, 27.305342 -100.013223^7^, elev. 636ft., 10.iv.1936, coll. S. Mulaik; deposited in AMNH. [examined]
Rhechostica moderatum Raven, 1985: 149.
Aphonopelma moderatum Smith, 1995: 122.
Aphonopelma heterops Chamberlin, 1940: 29; female syntypes from Edinburg, Hidalgo, Co., Texas, 26.30173 -98.16335^5^, elev. 96ft., ix-xii.1933, coll. S. Mulaik; deposited in the AMNH. [examined] **syn. n.**

#### Diagnosis.

*Aphonopelma moderatum* (Fig. 93) is a member of the *moderatum* species group can be identified by a combination of morphological, molecular, and geographic characteristics. Nuclear DNA identifies *Aphonopelma moderatum* as a phylogenetically distinct monophyletic lineage (Fig. 8), supported as the sister lineage to *Aphonopelma gabeli* and *Aphonopelma moellendorfi* sp. n. Females and subadult males are morphologically unique and can be readily identified by possessing an orange body disrupted by distinctly darkened (black or dark brown) banding on the patellae, metatarsi, and tarsi (Fig. 93). *Aphonopelma moderatum* can be distinguished from *Aphonopelma anax* by the shapes of its spermathecae and palpal bulbs; from *Aphonopelma armada* by lacking flared metatarsal scopulae and the unique pattern of setae on coxa I; and from *Aphonopelma gabeli* and *Aphonopelma hentzi* by phenotypic appearance. The most important measurement that distinguishes male *Aphonopelma moderatum* from its closely related phylogenetic and syntopic species is the article M1. Male *Aphonopelma moderatum* can be distinguished by possessing a larger M1/M4 (≥0.76; 0.76–0.81) than *Aphonopelma armada* (≤0.73; 0.63–0.73), *Aphonopelma gabeli* (≤0.74; 0.70–0.74), and *Aphonopelma hentzi* (≤0.75; 0.67–0.75); by possessing a smaller CL/M1 (≤1.30; 1.13–1.30) than *Aphonopelma anax* (≥1.36; 1.36–1.63) and *Aphonopelma armada* (≥1.36; 1.36–1.47); and a smaller PTl/M1 (≤0.69; 0.61–0.69) than *Aphonopelma armada* (≥0.73; 0.73–0.83) and *Aphonopelma hentzi* (≥0.74; 0.74–0.88). There are no significant measurements that separate male *Aphonopelma moderatum* from *Aphonopelma moellendorfi*. Significant measurements that distinguish female *Aphonopelma moderatum* from its closely related phylogenetic and syntopic species are Cl and T3. Female *Aphonopelma moderatum* can be distinguished by possessing a smaller Cl/A3 (≤2.46; 2.36–2.46) than *Aphonopelma armada* (≥2.48; 2.48–2.64); a smaller T3/T4 (≤0.70; 0.63–0.70) than *Aphonopelma gabeli* (≥0.73; 0.73–0.79); and a smaller P1/T3 (≤13.12; 8.14–13.12) than *Aphonopelma anax* (≥13.88; 13.88–19.15). There are no significant measurements that separate female *Aphonopelma moderatum* from *Aphonopelma hentzi*. Females of *Aphonopelma moellendorfi* are unknown and cannot be compared.

#### Description.

Male originally described by Chamberlin and Ivie (1939).

#### Redescription of male exemplar

(APH_0890; Fig. 94). *Specimen preparation and condition*: Specimen collected live from burrow, kept alive until mature, preserved in 80% ethanol; deposited in AUMNH; original coloration faded due to preservation. Left legs I, III, IV, and left pedipalp removed for measurements and photographs; stored in vial with specimen. Right leg III removed for DNA and stored at -80°C in the AUMNH (Auburn, AL). *General coloration*: Generally black or faded to brown. *Cephalothorax*: Carapace 15.55 mm long, 13.92 mm wide; densely clothed with black/brown iridescent pubescence appressed to surface; fringe covered in long setae not closely appressed to surface; foveal groove medium deep and straight; pars cephalica region rises gradually from foveal groove, gently arching anteriorly toward ocular area; AER slightly procurved, PER slightly recurved; normal sized chelicerae; clypeus extends forward on a curve; LBl 1.93, LBw 2.31; sternum hirsute, clothed with short length black, densely packed setae. *Abdomen*: Densely clothed in short black/brown pubescence with numerous longer, lighter setae interspersed (generally red or orange *in situ*); possessing a dense dorsal patch of black Type I urticating bristles (Cooke et al. 1972). *Legs*: Hirsute, particularly ventrally; densely clothed in a mix of short black/brown setae. Metatarsus I slightly curved. F1 16.48; F1w 3.33; P1 6.42; T1 13.45; M1 12.91; A1 7.57; F3 13.5; F3w 3.72; P3 5.52; T3 10.78; M3 12.89; A3 7.51; F4 15.26; F4w 3.45; P4 5.83; T4 13.53; M4 16.91; A4 8.18; femur III is normal - not noticeably swollen or wider than other legs. All tarsi fully scopulate. Extent of metatarsal scopulation: leg III (SC3) = 65.6%; leg IV (SC4) = 31.3%. Two ventral spinose setae on metatarsus III; seven ventral spinose setae on metatarsus IV; one spinose seta on the anterior margin of retrolateral tibia I. *Coxa I*: Prolateral surface a mix of fine, hair-like and tapered/thin tapered setae. *Pedipalps*: Hirsute; densely clothed in the same setal color as the other legs, with numerous longer ventral setae; one spinose seta at the apical, prolateral femur; one spinose seta on the prolateral patella; five spinose setae on the prolateral tibia; PTl 7.919, PTw 2.53. When extended, embolus tapers with a gentle curve to the retrolateral side near apex; embolus very slender, no keels; distinct dorsal groove where the bulb transitions to the embolus.

**Variation (9).** Cl 11.46–16.06 (14.409±0.52), Cw 9.49–14.51 (12.722±0.57), LBl 1.52–1.99 (1.811±0.05), LBw 1.7–2.36 (2.019±0.09), F1 12.35–17.39 (15.218±0.51), F1w 2.66–3.84 (3.453±0.12), P1 4.99–7.03 (6.133±0.23), T1 10.23–14.28 (12.43±0.43), M1 10.1–13.38 (11.754±0.35), A1 6.29–7.76 (6.957±0.18), L1 length 43.96–59.84 (52.491±1.65), F3 10.11–13.82 (12.24±0.43), F3w 2.88–4.21 (3.611±0.14), P3 4.17–6.13 (5±0.25), T3 7.62–10.88 (9.55±0.41), M3 9.45–13.75 (11.829±0.44), A3 5.36–7.51 (6.729±0.24), L3 length 36.71–52.05 (45.348±1.72), F4 11.54–16.23 (14.024±0.47), F4w 2.67–3.84 (3.347±0.14), P4 4.27–6.10 (5.234±0.24), T4 9.97–13.53 (12.232±0.38), M4 12.82–17.54 (15.129±0.52), A4 6.62–8.18 (7.478±0.21), L4 length 45.64–61.46 (54.098±1.77), PTl 6.258–8.692 (7.7±0.23), PTw 2.033–2.70 (2.441±0.07), SC3 ratio 0.481–0.782 (0.646±0.03), SC4 ratio 0.313–0.503 (0.411±0.02), Coxa 1 setae = tapered/thin tapered, F3 condition = normal/slightly swollen.

#### Description of female exemplar

(APH_0532; Figs 95–96). *Specimen preparation and condition*: Specimen collected live from burrow, preserved in 80% ethanol; deposited in AUMNH; original coloration faded due to preservation. Left legs I, III, IV, and pedipalp removed for photographs and measurements; stored in vial with specimen. Right leg III removed for DNA and stored at -80°C in the AUMNH (Auburn, AL). Genital plate with spermathecae removed and cleared, stored in vial with specimen. *General coloration*: Mostly appearing orange/brown with a mix of medium and longer length setae covering body, with black banding on the coxa, patella and metatarsi, and black tarsi. *Cephalothorax*: Carapace 15.6 mm long, 13.01 mm wide; densely clothed with brown/orange pubescence closely appressed to surface; fringe densely covered in long setae; foveal groove medium deep and slightly procurved; pars cephalica region rises from thoracic furrow more steeply than male, gently arching anteriorly toward ocular area; AER slightly procurved, PER recurved; clypeus extends forward on a curve; LBl 1.98, LBw 2.37; sternum hirsute, clothed with short, black/brown setae. *Abdomen*: Densely clothed in short black pubescence and longer gold/orange setae; dense dorsal patch of black Type I urticating bristles (Cooke et al. 1972); ventral transition to black setae. *Spermathecae*: Paired and separate, quickly tapering to capitate bulbs, with wide bases that are fused. *Legs*: Hirsute, particularly ventrally; densely clothed in a mix of yellow/orange pubescence with numerous longer setae, and black setae forming bands (either complete or incomplete) around the patella, metatarsus, and tarsus. *Coxa I*: Prolateral surface a mix of fine, hair-like and tapered/thin tapered setae. F1 13.14; F1w 3.86; P1 5.74; T1 10.17; M1 8.26; A1 6.13; F3 10.15; F3w 3.62; P3 5.45; T3 7.38; M3 8.57; A3 6.41; F4 12.66; F4w 3.64; P4 5.6; T4 10.42; M4 11.91; A4 6.69. All tarsi fully scopulate. Extent of metatarsal scopulation: leg III (SC3) = 70.1%; leg IV (SC4) = 42.9%. One ventral spinose seta on metatarsus III; four ventral spinose setae on metatarsus IV. *Pedipalps*: Densely clothed in the same setal color and patterns as the other legs; one spinose seta on the apical, prolateral femur, one spinose seta on the prolateral patella, and five spinose setae on the prolateral tibia.

**Variation (9).** Cl 9.98–15.62 (12.719±0.67), Cw 8.33–13.47 (10.64±0.61), LBl 1.37–2.23 (1.701±0.1), LBw 1.53–2.37 (1.914±0.1), F1 7.936–13.14 (10.457±0.6), F1w 2.497–3.98 (3.17±0.17), P1 3.662–6.09 (4.812±0.28), T1 6.485–10.17 (8.135±0.4), M1 4.719–8.26 (6.4±0.4), A1 3.974–6.27 (5.317±0.25), L1 length 26.776–43.44 (35.122±1.9), F3 5.974–10.15 (8.053±0.51), F3w 2.164–3.62 (2.766±0.19), P3 2.982–5.45 (3.686±0.33), T3 4.486–7.38 (5.541±0.35), M3 5.15–8.57 (6.447±0.45), A3 4.086–6.41 (5.171±0.32), L3 length 22.678–37.96 (28.898±1.92), F4 7.761–12.66 (10.041±0.54), F4w 2.287–3.66 (2.976±0.16), P4 3.37–5.76 (4.291±0.29), T4 6.417–10.42 (8.373±0.43), M4 7.353–11.91 (9.486±0.52), A4 4.808–6.91 (5.836±0.26), L4 length 29.709–47.28 (38.028±2.01), SC3 ratio 0.657–0.736 (0.699±0.01), SC4 ratio 0.334–0.486 (0.413±0.02), Coxa 1 setae = tapered/thin tapered. Spermathecae variation can be seen in Figure 96.

#### Material examined.

**United States: Texas: Dimmit**: Picnic area 3 miles NW Catarina on US-83, 28.375443 -99.64811 ^1^, 566ft., [APH_0471-0472, 11/4/2009, 2 juv, Brent E. Hendrixson, AUMNH]; **Hidalgo**: Edinberg, 26.290053 -98.155073 ^5^, 92ft., [APH_2208, 12/1937, 1♀, S. Mulaik, AMNH]; **Jim Hogg**: 1.4 miles W FM-649 on TX-16, 27.087044 -98.946036 ^1^, 594ft., [APH_1132, 16/3/2010, 1 juv, Brent E. Hendrixson, Gerri Wilson, Thomas Martin, AUMNH]; **Kinney**: 0.59 miles E US-277 on FM-693, 29.17044 -100.673259 ^1^, 972ft., [APH_1163, 17/3/2010, 1 juv, Brent E. Hendrixson, Gerri Wilson, Thomas Martin, AUMNH]; 4.6 miles SW US-90 on FM-1572, 29.21789 -100.260058 ^1^, 1002ft., [APH_1157-1160, 17/3/2010, 4 juv, Brent E. Hendrixson, Gerri Wilson, Thomas Martin, AUMNH]; 7.8 miles W Hwy-131 on Hwy-90 (W of Brackettville), 29.33578 -100.53469 ^2^, 1075ft., [APH_1448, 28/1/2012, 1 juv, Stanley A. Schultz, AUMNH]; Picnic area, 7.9 miles W FM-1572 on US-90, 29.280556 -100.323946 ^1^, 1074ft., [APH_0474-0475, 11/4/2009, 2 juv, Brent E. Hendrixson, AUMNH]; **Maverick**: 11.1 miles N Webb County Line on FM-1021, 28.303931 -100.219328 ^1^, 775ft., [APH_1140-1142, 16/3/2010, 3 juv, Brent E. Hendrixson, Gerri Wilson, Thomas Martin, AUMNH]; 4.55 miles S Kinney County Line on FM-1908, 29.020109 -100.569423 ^1^, 913ft., [APH_1161-1162, 17/3/2010, 2 juv, Brent E. Hendrixson, Gerri Wilson, Thomas Martin, AUMNH]; 4.6 miles ESE Eagle Pass (jct US-57) on US-277, 28.69567 -100.39303 ^1^, 833ft., [APH_0054, 17/7/2006, 1♀, Brent E. Hendrixson, AUMNH]; [APH_0057, 17/7/2006, 1♀, Brent E. Hendrixson, AUMNH]; [APH_0149, 17/7/2006, 1♀, Brent E. Hendrixson, AUMNH]; 5.2 miles N FM-2644 on FM-1021, 28.572348 -100.347434 ^1^, 751ft., [APH_1143-1146, 16/3/2010, 4 juv, Brent E. Hendrixson, Gerri Wilson, Thomas Martin, AUMNH]; 9.0 miles NE US-57 on FM-481, 28.928198 -100.262798 ^1^, 787ft., [APH_1147-1148, 17/3/2010, 2 juv, Brent E. Hendrixson, Gerri Wilson, Thomas Martin, AUMNH]; off dirt road near Hwy-277, 28.67713 -100.32325 ^2^, 830ft., [APH_1452-1453, 24/1/2012, 2 juv, Stanley A. Schultz, AUMNH]; **McMullen**: 0.7 miles S FM-624 on TX-16, 28.123878 -98.59089 ^1^, 389ft., [APH_1118-1121, 14/3/2010, 4 juv, Brent E. Hendrixson, Gerri Wilson, Thomas Martin, AUMNH]; **Starr**: 0.4 miles SW Ranch Rd on FM-755, 26.52287 -98.69055 ^1^, 482ft., [APH_1276, 12/5/2011, 1 juv, Brent E. Hendrixson, Kate Hall, Austin Deskewies, Alexis Guice, AUMNH]; 3.2 miles E FM-2360 on US-83, 26.289361 -98.593355 ^1^, 177ft., [APH_0464-0465, 10/4/2009, 2 juv, Brent E. Hendrixson, AUMNH]; 4.1 miles N US-83 on FM-3167, 26.429482 -98.850094 ^1^, 279ft., [APH_1126-1128, 15/3/2010, 3 juv, Brent E. Hendrixson, Gerri Wilson, Thomas Martin, AUMNH]; 5 miles east of Rio Grande City, 26.377617 -98.789218 ^5^, 151ft., [APH_2649, 29/4/1939, 1 juv, D. Mulaik, AMNH]; Falcon Reservoir, 26.574767 -99.128433 ^5^, 370ft., [APH_0890, 2006, 1♂, Dave Moellendorf, AUMNH]; Rio Grande City, 26.379787 -98.820305 ^5^, 161ft., [APH_2321, 29/9/1939, 1♀, 4♂, S. Mulaik, AMNH]; **Uvalde**: 1.93 miles NE Zavala County Line on FM-481, 29.095431 -99.961871 ^1^, 855ft., [APH_1153-1156, 17/3/2010, 4 juv, Brent E. Hendrixson, Gerri Wilson, Thomas Martin, AUMNH]; Uvalde, near Nueces River, 29.067017 -99.84935 ^5^, 783ft., [APH_0930, 2006, 1♂, Dave Moellendorf, AUMNH]; **Val Verde**: 0.25 miles N Kinney County Line on Hwy-277, 29.25733 -100.75319 ^2^, 897ft., [APH_1447, 26/1/2012, 1 juv, Stanley A. Schultz, AUMNH]; [APH_1456-1457, 26/1/2012, 2 juv, Stanley A. Schultz, AUMNH]; Amistad National Recreation Area, US-277 North Campground, 29.511963 -100.907314 ^1^, 1135ft., [APH_1168-1170, 17/3/2010, 3 juv, Brent E. Hendrixson, Gerri Wilson, Thomas Martin, AUMNH]; Comstock, 29.685333 -101.170783 ^1^, 1550ft., [APH_0891, 15/7/2008, 1♀, Chris A. Hamilton, AUMNH]; [APH_0893-0894, 15/7/2008, 1♂, 1♀, Chris A. Hamilton, AUMNH]; Comstock Cemetery, 29.685451 -101.170515 ^2^, 1565ft., [APH_0535, 4/6/2009, 1♀, Brent E. Hendrixson, Courtney Dugas, Sloan Click, AUMNH]; Del Rio - Hwy 90 W of Lake Amistad, 29.56805 -101.0745 ^5^, 1293ft., [APH_0929, 2006, 1♂, Dave Moellendorf, AUMNH]; Del Rio - Lake Amistad, 29.561667 -101.03155 ^1^, 1149ft., [APH_0892, 15/7/2008, 1♀, Chris A. Hamilton, AUMNH]; Del Rio - Lake Amistad, 29.4704 -100.952283 ^5^, 1112ft., [APH_0877, 7/2008, 1♀, Chris A. Hamilton, AUMNH]; Del Rio, 0.3 miles N Hwy-90 on Hwy-377, 29.4315 -100.908 ^2^, 1104ft., [APH_1445-1146, 31/1/2012, 2 juv, Stanley A. Schultz, AUMNH]; Lake Amistad Rec Area, Spur 406, 29.59828 -101.06573 ^1^, 1380ft., [APH_1291, 15/5/2011, 1 juv, Brent E. Hendrixson, Kate Hall, Austin Deskewies, Alexis Guice, AUMNH]; Lake Amistad Rec Area, Spur 454, 29.469723 -100.950532 ^1^, 1127ft., [APH_0534, 4/6/2009, 1 juv, Brent E. Hendrixson, Courtney Dugas, Sloan Click, AUMNH]; Lake Amistad Rec Area, US-277N campground, 29.51043 -100.90765 ^1^, 1125ft., [APH_1292, 15/5/2011, 1♂, Brent E. Hendrixson, Kate Hall, Austin Deskewies, Alexis Guice, AUMNH]; Langtry, along road that leads to dump, 29.81062 -101.56441 ^1^, 1325ft., [APH_1290, 15/5/2011, 1 juv, Brent E. Hendrixson, Kate Hall, Austin Deskewies, Alexis Guice, AUMNH]; Seminole Canyon State Park, N of campground, 29.6962 -101.32285 ^1^, 1400ft., [APH_1286-1287, 13/5/2011, 2 juv, Brent E. Hendrixson, Kate Hall, Austin Deskewies, Alexis Guice, AUMNH]; **Webb**: 0.64 miles E FM-2895 on US-59, 27.699808 -99.027114 ^1^, 562ft., [APH_1122-1124, 14/3/2010, 3 juv, Brent E. Hendrixson, Gerri Wilson, Thomas Martin, AUMNH]; 1.45 miles W FM-3338 on FM-1472, 27.661125 -99.584458 ^1^, 562ft., [APH_1133-1134, 16/3/2010, 2 juv, Brent E. Hendrixson, Gerri Wilson, Thomas Martin, AUMNH]; 10.15 miles SE Maverick County Line on Eagle Pass Rd, 28.100947 -99.993316 ^1^, 615ft., [APH_1138-1139, 16/3/2010, 2 juv, Brent E. Hendrixson, Gerri Wilson, Thomas Martin, AUMNH]; 3.6 miles NW I-35 on US-83, 27.80051 -99.46291 ^1^, 760ft., [APH_1283-1284, 13/5/2011, 2 juv, Brent E. Hendrixson, Kate Hall, Austin Deskewies, Alexis Guice, AUMNH]; 4.3 miles E Loop-20 on US-59 at Los Tios Creek, 27.561599 -99.390339 ^1^, 467ft., [APH_0467-0470, 11/4/2009, 4 juv, Brent E. Hendrixson, AUMNH]; 7.35 miles NW Mines Rd on FM-1472, 27.838017 -99.832079 ^1^, 531ft., [APH_1135-1137, 16/3/2010, 3 juv, Brent E. Hendrixson, Gerri Wilson, Thomas Martin, AUMNH]; Laredo, 27.530614 -99.480215 ^5^, 453ft., [APH_2319, 10/3/1936, 1♂, S. Mulaik, AMNH]; near Jct. Hwy-83 and I-35, 27.75556 -99.43975 ^2^, 711ft., [APH_1450-1451, 19/1/2012, 2 juv, Stanley A. Schultz, AUMNH]; roadside park, 5.7 miles E Loop-20 on Hwy-59, 27.5684 -99.36855 ^2^, 549ft., [APH_1454-1455, 18/1/2012, 2 juv, Stanley A. Schultz, AUMNH]; **Zapata**: 12.3 miles N FM-3169 (San Ygnacio) on Hwy-83, 27.21819 -99.41825 ^2^, 339ft., [APH_1449, 21/1/2012, 1 juv, Stanley A. Schultz, AUMNH]; 2.0 miles SE FM-3169 on US-83, 27.025086 -99.411585 ^1^, 364ft., [APH_0466, 10/4/2009, 1 juv, Brent E. Hendrixson, AUMNH]; Falcon State Park, 26.59031 -99.15428 ^1^, 306ft., [APH_0031, 15/3/2005, 1♂, Brent E. Hendrixson, AUMNH]; **Zavala**: 1.5 miles E Maverick County Line on FM-481, 29.054763 -100.087256 ^1^, 809ft., [APH_1151-1152, 17/3/2010, 2 juv, Brent E. Hendrixson, Gerri Wilson, Thomas Martin, AUMNH]; just S of Rest Area near Nueces River, 29.070874 -99.842183 ^1^, 844ft., [APH_0532-0533, 3/6/2009, 1♀, 1♂, Brent E. Hendrixson, Courtney Dugas, Sloan Click, AUMNH].

#### Distribution and natural history.

*Aphonopelma moderatum* is widely distributed in counties bordering the Rio Grande in Texas from Langtry (Val Verde County) to Edinburg (Hidalgo County) to more interior areas such as McMullen County (Figs 1I, 97). The species distribution model predicts suitable habitat for this species along the Rio Grande throughout northeastern Mexico in Coahuila, Nuevo Leon, and Tamaulipas (Fig. 97). Specimens collected by the authors were found at elevations from 50 to 475 meters in the Southern Texas Plains (particularly in Texas-Tamaulipas Thornscrub), Western Gulf Coast Plains, and Chihuahuan Deserts Level III Ecoregions. This species has been found in syntopy with *Aphonopelma anax* throughout much of its range and probably overlaps with *Aphonopelma armada*, *Aphonopelma hentzi*, and *Aphonopelma moellendorfi* in the extreme northwestern portion of its distribution near Amistad Reservoir in Val Verde County. Burrows are typical of that for North American tarantulas (i.e., circular and generally covered by a thin veil of silk) and specimens can be readily collected by pouring a small amount of water into their burrows. In areas with shallow soils (e.g., Chihuahuan Desert habitats west of Amistad Reservoir), these spiders have been found under large flat rocks. Adult males wander during the spring (March-June) and have been observed shortly before sunset and during overcast afternoons.

#### Conservation status.

The type specimens of *Aphonopelma heterops* (considered a synonym of *Aphonopelma moderatum*) were collected from Edinburg (Hidalgo County) in 1933. In the 80+ years since their collection, much of the land in this section of the Lower Rio Grande Valley has been converted for agricultural use. It is no surprise then that we failed to find these spiders near Edinburg despite traveling to the area numerous times. Consequently, *Aphonopelma moderatum* may be extirpated from the Edinburg-McAllen-Mission Metropolitan area, but these spiders may have never been particularly abundant in these parts in the first place given that the location is near the easternmost portion of their range. These tarantulas are among the most attractive species in the United States (Fig. 93) and are highly sought after by collectors for the pet trade. There is no evidence, however, that collectors have had any discernable impact on the numbers of this species. *Aphonopelma moderatum* is the most frequently encountered tarantula species at roadside picnic areas and along highway shoulders from Amistad Reservoir (Val Verde County) to Falcon Reservoir (Starr County), a distance of nearly 400 kilometers. Much of this area is surrounded and protected by privately owned ranches so the species appears to be secure.

#### Remarks.

*Aphonopelma moderatum* exhibits considerable sexual dimorphism in coloration. Adult females and immature specimens of both sexes possess perhaps the most distinctive color pattern of any species in the United States (i.e., tan to orange body with distinctly darker leg patellae, metatarsi, and tarsi). Adult males, on the other hand, are often solid black or dark brown. We suspect that Chamberlin and Ivie (1939) and Chamberlin (1940) described *Aphonopelma moderatum* and *Aphonopelma heterops*, respectively, due to these incredible differences in coloration (i.e., the description of *Aphonopelma moderatum* was based on adult male material whereas the description of *Aphonopelma heterops* was based on female or immature material). Our examination of the types and our extensive fieldwork throughout South Texas demonstrate that these two species are indeed conspecific. As a consequence, we consider *Aphonopelma heterops* a junior synonym of *Aphonopelma moderatum*.

Mitochondrial DNA (*CO1*) identifies *Aphonopelma moderatum* as a polyphyletic species with two divergent lineages spread across the tree (Fig. 7); both lineages were previously identified as separate species, one of which was cryptic, by Hamilton et al. (2014). Results from the AE analysis demonstrate that *CO1* is not effective at accurately delimiting species boundaries within this group due to deep mitochondrial divergence. Interestingly, the mtDNA genetic break between these two divergent lineages coincides with the area near Amistad Reservoir, at the convergence of the Tamaulipan Scrublands, Chihuahuan Desert, and Edwards Plateau. The apparent biogeographical shift between mtDNA lineages may be indicative of past Pleistocene fragmentation as proposed by Hamilton et al. (2011).

Other important ratios that distinguish males: *Aphonopelma moderatum* possess a larger L1/L4 (≥0.95; 0.95–0.99) than *Aphonopelma gabeli* (≤0.95; 0.90–0.95). For both males and females, certain morphometrics have potential to be useful but due to the amounts of variation, small number of specimens, and/or the small differences between species no others are claimed to be significant at this time (see Suppl. material 2). During evaluation of traditional PCA morphospace, males of *Aphonopelma moderatum* separate from *Aphonopelma anax*, *Aphonopelma armada*, and *Aphonopelma hentzi* along PC1~2, but do not separate in PCA morphological space from *Aphonopelma gabeli* or *Aphonopelma moellendorfi*. Females separate from *Aphonopelma anax* along PC1~2, but do not separate from *Aphonopelma armada*, *Aphonopelma gabeli*, or *Aphonopelma hentzi*. Interestingly, *Aphonopelma moderatum* males separate from *Aphonopelma anax*, *Aphonopelma armada*, and *Aphonopelma hentzi* in three-dimensional PCA morphospace (PC1~PC2~PC3), but do not separate from *Aphonopelma gabeli* and *Aphonopelma moellendorfi*. *Aphonopelma moderatum* females separate from *Aphonopelma anax*, but do not separate from *Aphonopelma armada*, *Aphonopelma gabeli*, and *Aphonopelma hentzi*. PC1, PC2, and PC3 explain ≥87% of the variation in male analyses and ≥96% of the variation in female analyses. It is important to note the tremendous variation in spermathecae shape that can be seen across *moderatum* populations (Fig. 96). Previous taxonomic work considered this variation enough to split and describe separate species; this is clearly not an effective character due to the large amounts of subtle variation that is possible.

### Aphonopelma moellendorfi

Taxon classificationAnimaliaAraneaeTheraphosidae

Hamilton sp. n.

http://zoobank.org/4BB4EC30-71DA-4352-8FE4-0845D67EAA0C

Figures 98 , 99

#### Types.

Male holotype (APH_0925) from N of Del Rio on Hwy 277, Val Verde Co., Texas, 29.488717 -100.907633 ^5^, elev. 1190ft., 2006, coll. Dave Moellendorf; deposited in AUMNH. Paratype male (APH_0928) from Del Rio - Hwy 90 W of Lake Amistad, Val Verde Co., Texas, 29.618868 -101.104385 ^5^, elev. 1406ft., 2006, coll. Dave Moellendorf; deposited in AMNH.

#### Etymology.

The specific epithet is a patronym in recognition of Dave Moellendorf, a friend and mentor to CAH. Moellendorf introduced CAH to the tarantula fauna of Texas and encouraged and fostered CAH’s early research on the genus *Aphonopelma*. Moellendorf has also spent countless hours educating the public on the importance of spiders on our planet.

#### Diagnosis.

*Aphonopelma moellendorfi* belongs to the *moderatum* species complex and can be distinguished by a combination of morphological, molecular, and geographic characteristics. Nuclear and mitochondrial DNA identifies *Aphonopelma moellendorfi* as a phylogenetically distinct monophyletic lineage (Figs 7–8), supported as the sister lineage to *Aphonopelma gabeli* and closely related to *Aphonopelma moderatum*. Immature and penultimate males of *Aphonopelma moellendorfi* can be distinguished from *Aphonopelma moderatum* and *Aphonopelma gabeli* due to their overall phenotypic appearance (i.e., *Aphonopelma moellendorfi* closely resemble *Aphonopelma hentzi*; D. Moellendorf, *pers. comm.*). Mature male *Aphonopelma moellendorfi* can be distinguished from *Aphonopelma anax* by the shape of their palpal bulbs. The significant measurement that distinguishes male *Aphonopelma moellendorfi* from its closely related phylogenetic and syntopic species is M1. Male *Aphonopelma moellendorfi* can be distinguished by possessing a larger M1/M3 (≥0.95; 0.95–1.00) than *Aphonopelma gabeli* (≤0.94; 0.90–0.94); by possessing a smaller PTl/M1 (≤0.69; 0.60–0.69) than *Aphonopelma armada* (≥0.73; 0.73–0.83) and *Aphonopelma hentzi* (≥0.74; 0.74–0.88); and a smaller CL/M1 (≤1.31; 1.10–1.31) than *Aphonopelma anax* (≥1.36; 1.36–1.63) and *Aphonopelma armada* (≥1.36; 1.36–1.47). There are no significant measurements that separate male *Aphonopelma moellendorfi* from *Aphonopelma moderatum*. Females of *Aphonopelma moellendorfi* are unknown at this time.

#### Description of male holotype

**Figure 98.**
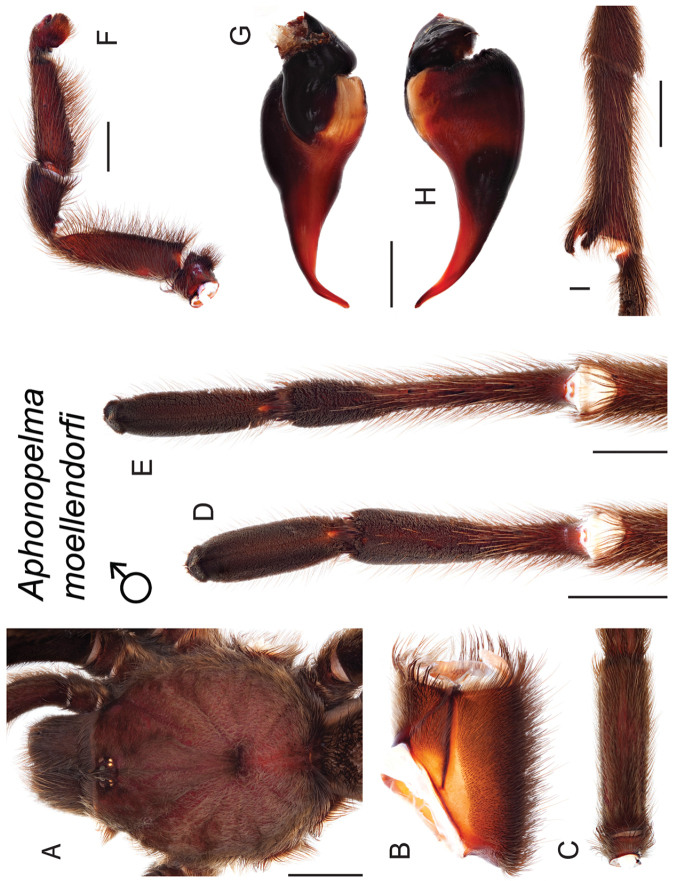
*Aphonopelma moellendorfi* sp. n. **A–I** male holotype, APH_0925 **A** dorsal view of carapace, scale bar = 6mm **B** prolateral view of coxa I **C** dorsal view of femur III **D** ventral view of metatarsus III, scale bar = 4.5mm **E** ventral view of metatarsus IV, scale bar = 3mm **F** prolateral view of L pedipalp and palpal tibia, scale bar = 4mm **G** dorsal view of palpal bulb **H** retrolateral view of palpal bulb, scale bar = 1mm **I** prolateral view of tibia I (mating clasper), scale bar = 5.5mm.

(APH_0925; Fig. 98). *Specimen preparation and condition*: Specimen collected live crossing road, preserved in 80% ethanol; original coloration faded due to preservation. Left legs I, III, IV, and left pedipalp removed for measurements and photographs; stored in vial with specimen. Right leg III removed for DNA and stored at -80°C in the AUMNH (Auburn, AL). *General coloration*: Generally black or faded to brown. *Cephalothorax*: Carapace 17.23 mm long, 15.48 mm wide; densely clothed with black/brown pubescence, with slight iridescence, appressed to surface; fringe covered in long setae not closely appressed to surface; foveal groove medium deep and straight; pars cephalica region rises gradually from foveal groove, gently arching anteriorly toward ocular area; AER slightly procurved, PER slightly recurved; normal sized chelicerae; clypeus straight; LBl 1.96, LBw 2.21; sternum hirsute, clothed with short black/brown, densely packed setae. *Abdomen*: Densely clothed in short black/brown pubescence with numerous longer, lighter setae interspersed (generally red or orange *in situ*); dense dorsal patch of black Type I urticating bristles (Cooke et al. 1972). *Legs*: Hirsute; densely clothed with short, similar length black/brown setae, and longer setae dorsally. Metatarsus I slightly curved. F1 17.53; F1w 4.15; P1 7.25; T1 13.73; M1 13.11; A1 8.20; F3 14.27; F3w 4.52; P3 5.79; T3 11.18; M3 13.14; A3 7.48; F4 15.96; F4w 4.35; P4 5.88; T4 13.78; M4 16.62; A4 8.77; femur III is normal - not noticeably swollen or wider than other legs. All tarsi fully scopulate. Extent of metatarsal scopulation: leg III (SC3) = 67.3%; leg IV (SC4) = 40.2%. One ventral spinose seta on metatarsus III; five ventral spinose setae on metatarsus IV. The prolateral branch of the tibial apophyses possesses a very large megaspine that projects anteriorly. *Coxa I*: Prolateral surface a mix of fine, hair-like and thin tapered setae. *Pedipalps*: Hirsute; densely clothed in the same setal color as the other legs, with numerous longer ventral setae; one spinose seta at the apical, prolateral femur; one spinose seta on the prolateral patella; two spinose setae on the prolateral tibia; PTl 9.125, PTw 2.051. When extended, embolus tapers with a gentle curve to the retrolateral side near apex; embolus slender (i.e., more stout than *hentzi*), no keels.

**Variation (5).** Cl 15.05–17.56 (16.192±0.51), Cw 13.27–15.48 (14.308±0.39), LBl 1.65–2.04 (1.9±0.07), LBw 1.84–2.28 (2.124±0.09), F1 16.36–18.10 (17.338±0.32), F1w 3.42–4.15 (3.81±0.14), P1 6.31–7.25 (6.698±0.17), T1 13.57–15.75 (14.396±0.41), M1 13.11–15.27 (13.814±0.42), A1 7.68–8.49 (8.126±0.14), L1 length 57.15–64.29 (60.372±1.25), F3 13.56–15.47 (14.28±0.34), F3w 3.7–4.52 (4.034±0.14), P3 4.84–5.79 (5.36±0.18), T3 10.02–11.79 (10.972±0.38), M3 13.11–15.48 (14.032±0.45), A3 7.04–8.47 (7.678±0.24), L3 length 49.71–56.88 (52.322±1.34), F4 17.29–15.38 (16.23±0.31), F4w 4.35–3.51 (3.846±0.15), P4 6.02–5.14 (5.536±0.17), T4 12.52–14.12 (13.38±0.31), M4 16.04–18.88 (17.518±0.54), A4 8.14–9.17 (8.616±0.17), L4 length 58.64–64.39 (61.28±1.15), PTl 8.6–9.175 (8.898±0.12), PTw 2.051–2.67 (2.418±0.11), SC3 ratio 0.571–0.785 (0.674±0.03), SC4 ratio 0.341–0.481 (0.409±0.03), Coxa I setae = thin tapered, F3 condition = normal.

#### Material examined.

**United States: Texas: Presidio**: Hwy 169, 29.938343 -104.034011 ^6^, 3992ft., [APH_0948, 2006, 1♂, Dave Moellendorf, AUMNH]; Sierra Vieja Mtns, 13 miles west Valentine, 30.723313 -104.669391 ^4^, 4127ft., [APH_0938, 2006, 1♂, Dave Moellendorf, AUMNH]; [APH_0969, 2006, 1♂, Dave Moellendorf, AUMNH]; **Val Verde**: Del Rio - Hwy 90 W of Lake Amistad, 29.618868 -101.104385 ^5^, 1406ft., [APH_0928, 2006, 1♂, Dave Moellendorf, AMNH]; N of Del Rio on Hwy 277, 29.488717 -100.907633 ^5^, 1190ft., [APH_0925, 2006, 1♂, Dave Moellendorf, AUMNH].

#### Distribution and natural history.

**Figure 99.**
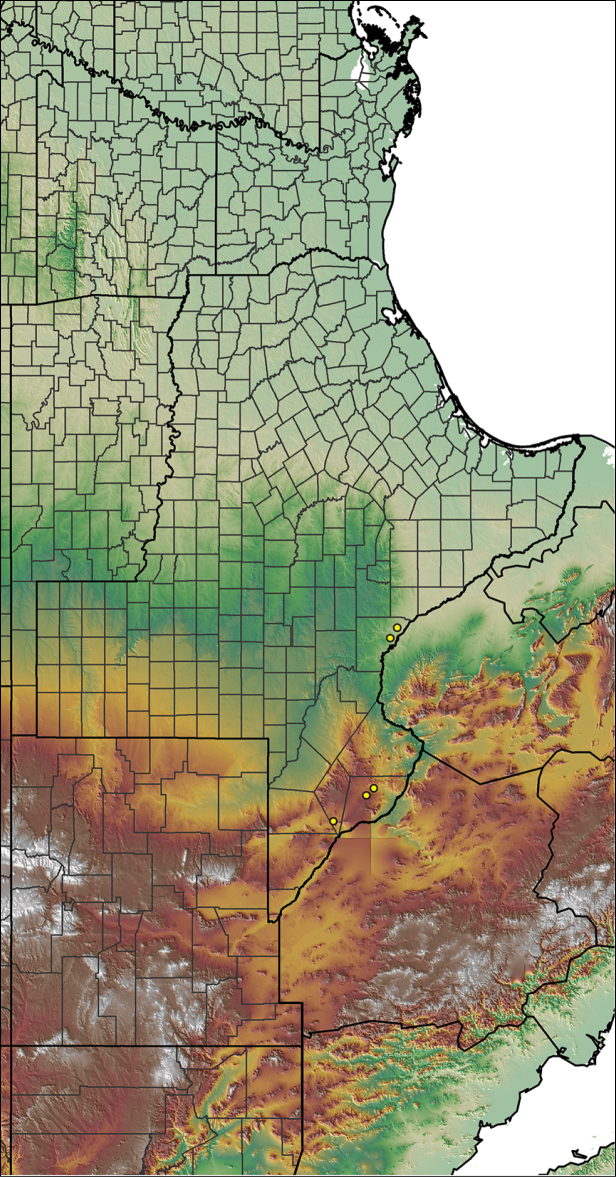
*Aphonopelma moellendorfi* sp. n. distribution of known specimens. There is no predicted distribution map due to the limited number of sampling localities and restricted distribution this species possesses.

*Aphonopelma moellendorfi* is presently known from only a handful of localities in southwestern Texas, along the border with Mexico from Del Rio west to the Sierra Vieja Mountains (Fig. 99), where they can be found inhabiting the following Level III Ecoregions: Southern Texas Plains and Chihuahuan Deserts. *Aphonopelma moellendorfi* can be found in syntopy with a handful of other species across its distribution including *Aphonopelma armada*, *Aphonopelma hentzi*, and *Aphonopelma moderatum*. The breeding season, when mature males abandon their burrows in search of females, seems to be limited to late spring and summer (May–July). Extensive sampling throughout extreme West Texas and northern Mexico will be necessary to fully understand the distribution of this species.

#### Conservation status.

This enigmatic species is only known from a handful of localities in the United States so it would be premature to comment on its conservation status. Fieldwork in West Texas and northern Mexico is necessary to more fully understand the abundance and extent of this species.

#### Remarks.

*Aphonopelma moellendorfi* males are morphologically similar to *Aphonopelma gabeli* and *Aphonopelma moderatum*, and lack the characteristic shiny copper or bronze carapace found in *Aphonopelma hentzi*. But interestingly, while females of *Aphonopelma moellendorfi* remain unknown, it should be noted that immature males closely resemble *Aphonopelma hentzi* (D. Moellendorf, *pers. comm.*, collected and raised immature males to maturity). Other important ratios that distinguish males: *Aphonopelma moellendorfi* possess a larger L1/L4 (≥0.96; 0.96–1.00) than *Aphonopelma gabeli* (≤0.95; 0.90–0.95) and *Aphonopelma anax* (≤0.94; 0.88–0.94); by possessing a larger M1/F4 (≥0.81; 0.81–0.88) than *Aphonopelma anax* (≤0.75; 0.69–0.75) and *Aphonopelma hentzi* (≤0.77; 0.68–0.77). Certain morphometrics have potential to be useful, though due to the amounts of variation, small number of specimens, and the small differences between species, no other are claimed to be significant at this time (see Suppl. material 2). During evaluation of traditional PCA morphospace, males of *Aphonopelma moellendorfi* separate from *Aphonopelma armada*, *Aphonopelma hentzi*, and *Aphonopelma anax* along PC1~2, but do not separate from *Aphonopelma gabeli* or *Aphonopelma moderatum*. Interestingly, *Aphonopelma moellendorfi* males separate from *Aphonopelma anax*, *Aphonopelma armada*, and *Aphonopelma hentzi* in three-dimensional PCA morphospace (PC1~PC2~PC3), but do not separate from *Aphonopelma gabeli* and *Aphonopelma moderatum*. Females of *Aphonopelma moellendorfi* are unknown at this time. PC1, PC2, and PC3 explain ≥96% of the variation in male analyses. Unfortunately, at this time we do not have photos of live specimens.

### Aphonopelma mojave

Taxon classificationAnimaliaAraneaeTheraphosidae

Prentice, 1997

**Figure 100.**
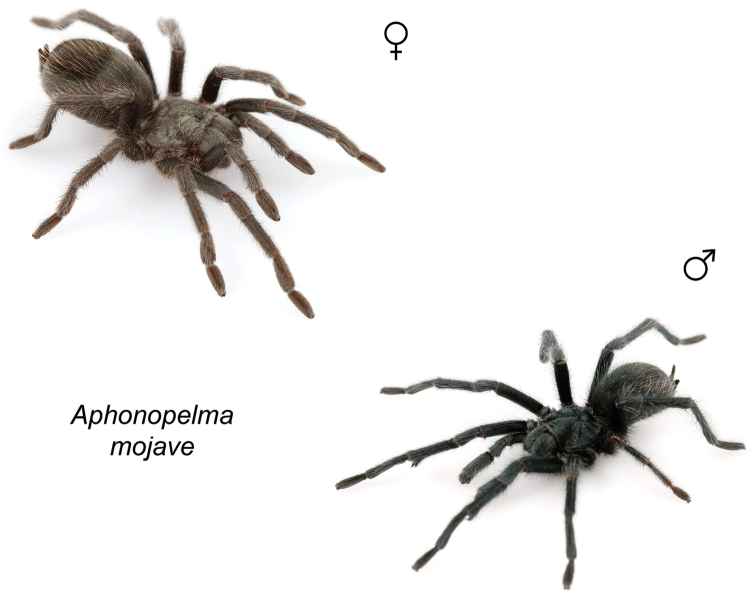
*Aphonopelma mojave* Prentice, 1997 specimens, live photographs. Female (L) - APH_3101; Male (R) - APH_3102.

**Figure 101.**
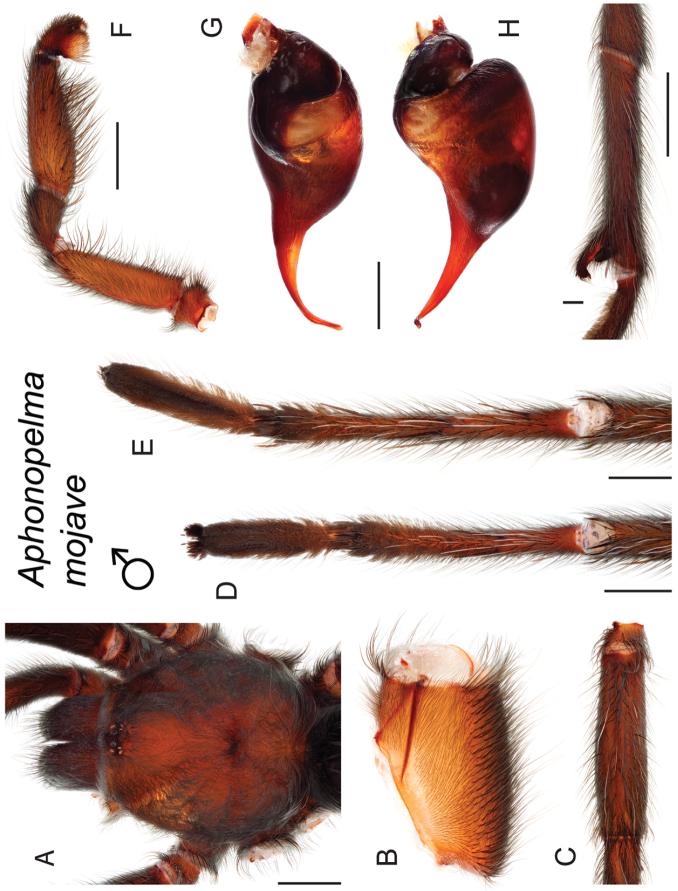
*Aphonopelma mojave* Prentice, 1997. **A–I** male specimen, APH_1561 **A** dorsal view of carapace, scale bar = 2.5mm **B** prolateral view of coxa I **C** dorsal view of femur III **D** ventral view of metatarsus III, scale bar = 2mm **E** ventral view of metatarsus IV, scale bar = 2mm **F** prolateral view of L pedipalp and palpal tibia, scale bar = 2.5mm **G** dorsal view of palpal bulb **H** retrolateral view of palpal bulb, scale bar = 0.5mm **I** prolateral view of tibia I (mating clasper), scale bar = 3mm.

**Figure 102.**
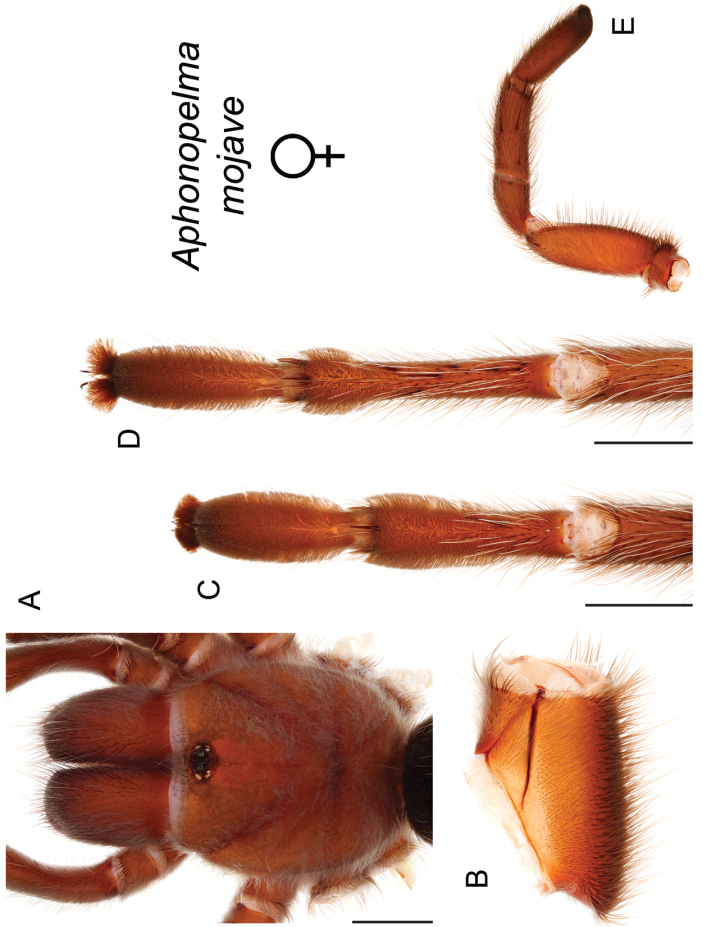
*Aphonopelma mojave* Prentice, 1997. **A–E** female specimen, APH_1558 **A** dorsal view of carapace, scale bar = 3mm **B** prolateral view of coxa I **C** ventral view of metatarsus III, scale bar = 2.5mm **D** ventral view of metatarsus IV, scale bar = 2.5mm **E** prolateral view of L pedipalp and palpal tibia.

**Figure 103.**
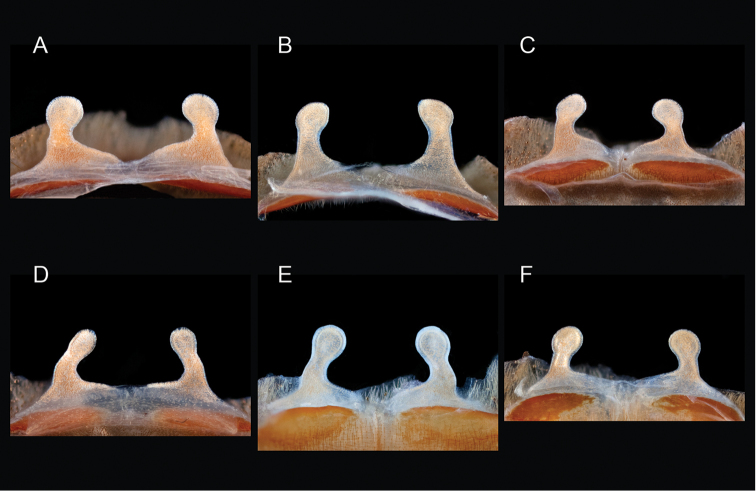
*Aphonopelma mojave* Prentice, 1997. **A–F** cleared spermathecae **A** *mojave* allotype **B** APH_1355 **C** APH_1558 **D** APH_3101 **E** AUMS_2364 **F** AUMS_2512.

**Figure 104.**
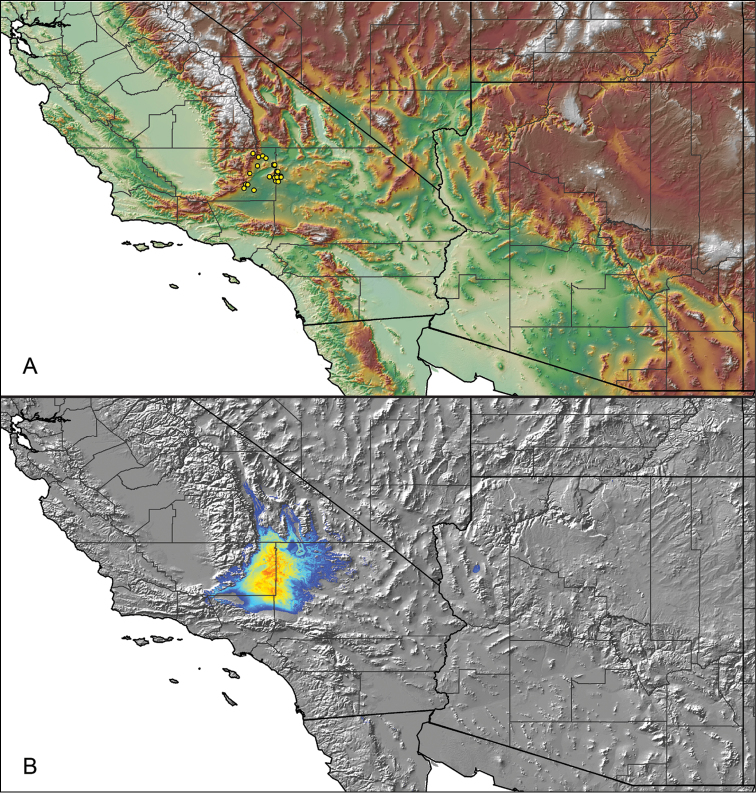
*Aphonopelma mojave* Prentice, 1997. **A** distribution of known specimens **B** predicted distribution; warmer colors (red, orange, yellow) represent areas of high probability of occurrence, cooler colors (blue shades) represent areas of low probability of occurrence.

Figures 100 , 101 , 102 , 103 , 104

Aphonopelma mojave Prentice, 1997: 157; male holotype and female allotype from S of Red Mountain, 20 miles N of Kramer Jct. on Hwy 395, 1–2 miles W of Hwy 395, San Bernardino and Kern Co. line, California, 35.310573 -117.644350^4^, elev. 3405ft., 28.x.1989 (male), 20.x.1991 (female), coll. Thomas R. Prentice; deposited in AMNH. [examined]

#### Diagnosis.

*Aphonopelma mojave* (Fig. 100) is a member of the *paloma* species group and can be identified by a combination of morphological, molecular, and geographic characteristics. Nuclear and mitochondrial DNA identifies *Aphonopelma mojave* as a strongly supported monophyletic lineage (Figs 7–8) that is a sister lineage to *Aphonopelma atomicum* sp. n., *Aphonopelma icenoglei* sp. n., *Aphonopelma prenticei* sp. n., and *Aphonopelma joshua*. *Aphonopelma mojave* can easily be distinguished from syntopic populations of *Aphonopelma iodius* by their smaller size and limited extent of scopulation on metatarsi III and IV, and from other members of the *paloma* species group by locality. The most significant measurements that distinguish male *Aphonopelma mojave* from its closely related phylogenetic and syntopic species are F4, PTl, and the extent of scopulation on metatarsus IV. Male *Aphonopelma mojave* can be distinguished by possessing a larger PTl/F4 (≥0.57; 0.57–0.62) than *Aphonopelma joshua* (≤0.51; 0.48–0.51); and by possessing a smaller L4 scopulation extent (30%-44%) than *Aphonopelma iodius* (62%-88%). There are no significant measurements that separate male *Aphonopelma mojave* from *Aphonopelma atomicum*, *Aphonopelma icenoglei*, or *Aphonopelma prenticei*, although *Aphonopelma mojave* males do not possess the swollen femur III condition that is known in *Aphonopelma atomicum* and *Aphonopelma prenticei*). The most significant measurements that distinguish female *Aphonopelma mojave* from its closely related phylogenetic and syntopic species are Cl, F3, T3, and the extent of scopulation on metatarsus IV. Female *Aphonopelma mojave* can be distinguished by possessing a larger A1/F3 (≥0.56; 0.56–0.67) than *Aphonopelma atomicum* (≤0.56; 0.53–0.56); a larger F1/T3 (≥1.57; 1.57–1.92) than *Aphonopelma joshua* (≤1.57; 1.47–1.57); by possessing a smaller Cl/M4 (≤1.31; 1.20–1.31) than *Aphonopelma prenticei* (≥1.31; 1.31–1.52); and a smaller L4 scopulation extent (34%-52%) than *Aphonopelma iodius* (59%-83%). There are no significant measurements that separate female *Aphonopelma mojave* from *Aphonopelma icenoglei*. *Aphonopelma mojave* and *Aphonopelma icenoglei* are morphologically very similar but are not sister lineages (Fig. 8) and have allopatric distributions.

#### Description.

Male and female originally described by Prentice (1997).

#### Redescription of male exemplar

(APH_1561; Fig. 101). *Specimen preparation and condition*: Specimen collected live wandering, preserved in 80% ethanol; deposited in AUMNH; original coloration faded due to preservation. Left legs I, III, IV, and left pedipalp removed for measurements and photographs; stored in vial with specimen. Right legs III & IV removed for DNA and stored at -80°C in the AUMNH (Auburn, AL). *General coloration*: Faded black or brown. *Cephalothorax*: Carapace 7.454 mm long, 6.979 mm wide; Very hirsute; densely clothed with faded black pubescence mostly appressed to surface; fringe covered in long setae not closely appressed to surface; posterior region of carapace with long setae; foveal groove medium deep and straight; pars cephalica region rises gradually from foveal groove to ocular area; AER very slightly procurved, PER recurved; normal sized chelicerae; clypeus extends forward on a curve; LBl 1.03, LBw 1.266; sternum hirsute, clothed with faded black, densely packed, short setae. *Abdomen*: Densely clothed in short black pubescence with numerous longer red/orange setae interspersed; possessing a dense dorsal patch of black Type I urticating bristles (Cooke et al. 1972) - smaller and distinct from large species. *Legs*: Hirsute; densely clothed in faded black pubescence. Metatarsus I straight. F1 8.246; F1w 1.77; P1 2.873; T1 7.339; M1 6.254; A1 4.705; F3 7.408; F3w 2.013; P3 2.564; T3 5.684; M3 7.373; A3 4.672; F4 8.639; F4w 1.754; P4 2.468; T4 7.362; M4 9.201; A4 4.984; femur III is normal. All tarsi fully scopulate. Extent of metatarsal scopulation: leg III (SC3) = 64.3%; leg IV (SC4) = 34%. Two ventral and one prolateral spinose setae on metatarsus III; four ventral spinose setae and one prolateral on metatarsus IV; two prolateral spinose setae on tibia I; one large megaspine is present at the apex on the retrolateral tibia of the mating clasper. *Coxa I*: Prolateral surface covered by fine, hair-like setae. *Pedipalps*: Very hirsute, particularly ventrally; densely clothed in the same setal color as the other legs; one spinose seta near the anterior margin of the prolateral palpal femur and five spinose setae on the prolateral palpal tibia; PTl 5.132, PTw 1.612. Palpal bulb is short and stout; distinct transition between bulb and embolus; embolus tapers and gently curves to the retrolateral side, no keels.

**Variation (6).** Cl 7.374–8.988 (7.862±0.25), Cw 6.689–8.035 (7.185±0.2), LBl 1.03–1.206 (1.079±0.03), LBw 1.22–1.371 (1.272±0.02), F1 7.352–9.266 (8.251±0.25), F1w 1.764–2.218 (1.908±0.07), P1 2.873–3.421 (3.16±0.08), T1 6.69–8.041 (7.323±0.19), M1 6.01–7.277 (6.492±0.17), A1 3.87–4.994 (4.451±0.17), L1 length 27.868–32.999 (29.677±0.76), F3 6.577–7.938 (7.23±0.19), F3w 1.915–2.449 (2.081±0.08), P3 2.537–2.931 (2.686±0.07), T3 5.162–6.539 (5.683±0.19), M3 6.462–7.865 (7.072±0.2), A3 4.293–5.387 (4.685±0.17), L3 length 25.19–30.66 (27.355±0.77), F4 8.283–9.518 (8.682±0.18), F4w 1.754–2.14 (1.874±0.06), P4 2.468–3.188 (2.791±0.11), T4 6.99–8.241 (7.506±0.17), M4 8.358–9.946 (8.998±0.23), A4 4.446–5.929 (4.984±0.24), L4 length 30.958–36.657 (32.961±0.83), PTl 4.866–5.584 (5.153±0.1), PTw 1.536–2.029 (1.75±0.08), SC3 ratio 0.601–0.792 (0.666±0.03), SC4 ratio 0.303–0.438 (0.377±0.02), Coxa I setae = very thin tapered, F3 condition = normal.

#### Description of female exemplar

(APH_1558; Figs 102–103). *Specimen preparation and condition*: Specimen collected live from burrow, preserved in 80% ethanol; deposited in AUMNH; original coloration faded due to preservation. Left legs I, III, IV, and pedipalp removed for photographs and measurements; stored in vial with specimen. Right legs III & IV removed for DNA and stored at -80°C in the AUMNH (Auburn, AL). Genital plate with spermathecae removed and cleared, stored in vial with specimen. *General coloration*: Faded black or brown. *Cephalothorax*: Carapace 7.687 mm long, 7.094 mm wide; Hirsute, densely clothed with short faded black or brown pubescence closely appressed to surface; fringe densely covered in slightly longer setae; foveal groove medium deep and slightly procurved; pars cephalica region gently rises from thoracic furrow, arching anteriorly toward ocular area; AER very slightly procurved - mostly straight, PER recurved; chelicerae slightly robust, clypeus extends forward on a curve; LBl 1.281, LBw 1.41; sternum hirsute, clothed with short faded black or brown setae. *Abdomen*: Densely clothed dorsally in short black, faded black setae with longer, lighter setae (generally red or orange *in situ*) focused near the urticating patch; dense dorsal patch of black Type I urticating bristles (Cooke et al. 1972) - smaller and distinct from large species. *Spermathecae*: Paired and separate, short, with capitate bulbs and wide bases that are not fused. *Legs*: Hirsute; densely clothed in short faded black or brown pubescence; F1 6.846; F1w 1.957; P1 2.985; T1 5.697; M1 4.34; A1 3.591; F3 5.573; F3w 1.837; P3 2.536; T3 4.24; M3 4.45; A3 4.218; F4 7.245; F4w 1.888; P4 2.433; T4 5.975; M4 6.167; A4 4.753. All tarsi fully scopulate. Extent of metatarsal scopulation: leg III (SC3) = 76.8%; leg IV (SC4) = 34.3%. One ventral spinose seta on metatarsus III; seven ventral spinose setae on metatarsus I. *Coxa I*: Prolateral surface covered by fine, hair-like setae. *Pedipalps*: Densely clothed in the same setal color as the other legs; one spinose seta on the apical, prolateral femur, seven prolateral (three at the apical, prolateral border with the tarsus) and two ventral spinose setae on the tibia (one at the apical, ventral border with the tarsus).

**Variation (7).** Cl 6.911–8.471 (7.803±0.21), Cw 6.203–7.648 (6.858±0.2), LBl 1.071–1.48 (1.24±0.05), LBw 1.263–1.70 (1.453±0.06), F1 5.84–7.52 (6.877±0.21), F1w 1.66–1.962 (1.873±0.04), P1 2.15–3.163 (2.774±0.12), T1 4.72–6.53 (5.642±0.21), M1 3.73–4.66 (4.242±0.12), A1 3.16–4.18 (3.588±0.12), L1 length 19.6–25.75 (23.122±0.72), F3 5.311–6.34 (5.747±0.13), F3w 1.37–1.849 (1.666±0.07), P3 2.06–2.774 (2.426±0.1), T3 3.38–4.76 (4.153±0.17), M3 4.05–4.87 (4.477±0.12), A3 3.08–4.218 (3.737±0.13), L3 length 18.14–22.45 (20.54±0.56), F4 6.68–7.76 (7.196±0.14), F4w 1.29–1.942 (1.771±0.09), P4 2.433–2.93 (2.673±0.08), T4 5.37–6.26 (5.767±0.12), M4 5.68–6.65 (6.192±0.14), A4 3.56–4.753 (4.238±0.15), L4 length 23.73–28.17 (26.067±0.56), SC3 ratio 0.653–0.768 (0.706±0.02), SC4 ratio 0.343–0.525 (0.407±0.03), Coxa I setae = very thin tapered. Spermathecae variation can be seen in Figure 103.

#### Material examined.

**United States: California: Kern**: 7 miles north of Johannesburg, 35.4804 -117.654683 ^5^, 3412ft., [APH_2669, 9/10/1968, 1♂, W. Icenogle, AMNH]; 8 miles NW San Bernardino County Line along Hwy 395, 35.469394 -117.65434 ^1^, 3482ft., [APH_0754-0755, 4/10/2009, 2 juv, Brent E. Hendrixson, Thomas Martin, AUMNH]; 8.1 miles into Kern county on Hwy 395, 35.480904 -117.67768 ^4^, 4003ft., [APH_2390, 20/10/1991, 1♂, T.R. Prentice, AMNH]; 8.2 miles E Hwy-14 on Hwy-58, 35.016745 -118.035457 ^1^, 2596ft., [APH_1323-1325, 30/7/2011, 3♀, Brent E. Hendrixson, Brendon Barnes, Nate Davis, Jake Storms, AUMNH]; 9.7 miles W US-395 on Randsburg-Mojave Rd (Osdick Rd becomes Randsburg-Mojave Rd), 35.26131 -117.75031 ^1^, 3287ft., [APH_0326-0327, 7/5/2008, 2 juv, Brent E. Hendrixson, Zach Valois, AUMNH]; along power line road west of Hwy-14, 35.462553 -117.971773 ^1^, 3199ft., [APH_1204-1207, 31/7/2010, 4 juv, Brent E. Hendrixson, Brendon Barnes, Nate Davis, AUMNH]; E of Mojave, near Hwy 58, off Cache Creek Rd, 35.1261 -118.18484 ^1^, 3252ft., [APH_3101, 18/7/2012, 1♀, Chris A. Hamilton, Amy Skibiel, AUMNH]; E of Mojave, near Hwy 58, off Randsburg Cutoff Rd, 35.11755 -118.15001 ^1^, 2974ft., [APH_3102, 19/7/2012, 1♂, Chris A. Hamilton, Amy Skibiel, AUMNH]; Jawbone Canyon area, 35.32137 -118.11149 ^5^, 2900ft., [APH_1355, 21/8/2011, 1♀, Warren Burke, AUMNH]; Ransburg-Inyokern Rd., 1 mile W of Hwy 395, 35.641784 -117.874597 ^5^, 2855ft., [AUMS_2530, 20/10/1991, 1♂, T.R. Prentice, AUMNH]; Sage canyon, 35.600649 -117.814267 ^5^, 2554ft., [AUMS_2576, 26/10/1975, 1♂, Ray Jillison, AUMNH]; SE of Walker’s Pass, NE side of SR-178/Walker’s Pass Rd, 35.62323 -117.95274 ^1^, 3985ft., [APH_0399, 4/8/2008, 1♀, Zach Valois, AUMNH]; Walker Pass, Hwy 178, 35.687958 -118.051992 ^5^, 4605ft., [AUMS_2526, 20/10/1990, 1♂, T.R. Prentice, AUMNH]; west Mojave, 35.04916 -118.217596 ^7^, 2985ft., [AUMS_2462, 1997, 1♂, T.R. Prentice, AUMNH]; **San Bernardino**: 0.25 miles E Hwy-395 on Cuddeback Rd, 35.255317 -117.608121 ^1^, 3016ft., [APH_1555-1556, 25/10/2012, 1♀, 1 juv, Brent E. Hendrixson, AUMNH]; 10 to 17 miles S of Johannesburg, 35.172459 -117.59183 ^5^, 2787ft., [AUMS_3317, 10/10/1974, 1♂, W. Icenogle, AUMNH]; [APH_2403, 10/10/1974, 1♀, W. Icenogle, AMNH]; [APH_2406, 10/10/1974, 1♂, W. Icenogle, AMNH]; 8.1 miles south of Kelso on Kelbaker Rd., 34.899855 -115.649338 ^4^, 2825ft., [APH_2439, 1/11/1992, 1♂, T.R. Prentice, AMNH]; Red Mountain, 35.358297 -117.616726 ^6^, 3609ft., [AUMS_2513, 14/10/1990, 1♀, T.R. Prentice, AUMNH]; [AUMS_2527, 20/10/1994, 1♀, Geogr, AUMNH]; Red Mountain, 1 mile west of Powerline Rd. , 35.128369 -116.794985 ^4^, 1972ft., [APH_2413, 26/10/1991, 1♂, T.R. Prentice, AMNH]; Red Mountain, 1.2 miles west of 20 Mule Team Rd., 35.247194 -117.652967 ^4^, 3005ft., [APH_2412, 26/10/1991, 1♂, T.R. Prentice, AMNH]; Red Mountain, 19 miles N of Kramer Junction, 35.254038 -117.553643 ^5^, 3262ft., [AUMS_2514, 3/10/1992, 1♂, T.R. Prentice, AUMNH]; [AUMS_2535, 13/10/1991, 1♂, T.R. Prentice, AUMNH]; [APH_2388, 28/10/1989, 1♂, T.R. Prentice, AMNH]; [APH_2405, 25/1/1992, 1♀, T.R. Prentice, AMNH]; [APH_2407, 13/10/1997, 1♀, T.R. Prentice, AMNH]; [APH_2408, 26/10/1991, 1♂, T.R. Prentice, AMNH]; [APH_2410, 14/10/1991, 1♂, T.R. Prentice, AMNH]; [APH_2411, 20/10/1991, 1♀, T.R. Prentice, AMNH]; [APH_2414, 26/10/1991, 1♂, T.R. Prentice, AMNH]; Red Mountain, 20 miles N of Kramer Junction, 35.301692 -117.614683 ^5^, 3220ft., [AUMS_2510, 16/10/1992, 1 juv, T.R. Prentice, AUMNH]; Red Mountain, 20 miles N of Kramer Junction, Hwy 395, W side, 35.305756 -117.615541 ^5^, 3256ft., [AUMS_2512, 4/10/1992, 1♂, T.R. Prentice, AUMNH]; Red Mountain, campsite W of power line road, 35.331378 -117.612816 ^5^, 3389ft., [AUMS_2364, 20/10/1991, 1♂, T.R. Prentice, AUMNH]; Red Mountain, campsite, 19 miles north , 35.358297 -117.616726 ^5^, 3602ft., [APH_2404, 4/10/1992, 1♀, T.R. Prentice, AMNH]; Red Mountain, junction of 20 Mule Team Rd. and PL Rd., 35.25197 -117.611916 ^5^, 2999ft., [APH_2409, 26/10/1991, 1♂, T.R. Prentice, AMNH]; Red Mountain, South of Power Line Road, 35.321067 -117.611791 ^5^, 3409ft., [AUMS_2531, 28/10/1989, 1♂, T.R. Prentice, AUMNH]; W of Hwy 395 along Twenty Mule Team Pkwy, 35.253597 -117.615238 ^1^, 3013ft., [APH_1558-1561, 25/10/2012, 2♀, 2♂, Brent E. Hendrixson, AUMNH].

#### Distribution and natural history.

The redefined *Aphonopelma mojave* has a narrow distribution centered on the western Mojave Desert and extreme southeastern foothills of the Sierra Nevada Mountains in eastern Kern and northwestern San Bernardino Counties, California (Fig. 104), inhabiting the Western Mojave Basins Level III Ecoregion. Accurately georeferenced specimens have been collected at elevations between 775 and 1225 meters. The species distribution model suggests that suitable habitat may be present in adjacent sections of southwestern Inyo and northeastern Los Angeles Counties (Fig. 104B). Additional sampling is required to assess the northern extent of the distribution of *Aphonopelma mojave* along the Highway-395 corridor. *Aphonopelma mojave* is syntopic with *Aphonopelma iodius* (burrows of both species have been located within one meter of each other) and may overlap its range with *Aphonopelma icenoglei* in the vicinity of Kramer Junction. Burrow entrances are generally surrounded by a distinct mound or turret made of excavated soil and silk (Fig. 2D–E). Mating takes place during daylight hours in the autumn (October-November). The natural history of *Aphonopelma mojave* and its close relatives is discussed in more detail in Prentice (1997).

#### Conservation status.

In addition to having a relatively narrow distribution, habitat degradation due to Off-Highway Vehicle (OHV) recreational activity probably poses the greatest threat to *Aphonopelma mojave*. However, these spiders are abundant (especially near the type locality), with several areas within the western Mojave Desert that are managed by the State of California or Bureau of Land Management (e.g., West Mojave Desert Ecological Reserve, Fremont Valley Ecological Reserve, Red Rock Canyon State Park, Desert Tortoise Natural Area). This species is likely secure.

#### Remarks.

Prentice (1997) noted that *Aphonopelma mojave* comprised two different geographic “races” (i.e., western and eastern lineages) but he did not describe two different species. Our results (also see Hendrixson et al. 2013, Graham et al. 2015) demonstrate that these two populations are indeed distinct species, but perhaps even more interesting is that the western and eastern groups comprise additional species-level diversity. We now recognize four different species that were all previously considered *Aphonopelma mojave* (*Aphonopelma atomicum*, *Aphonopelma icenoglei*, *Aphonopelma mojave*, and *Aphonopelma prenticei*). For both males and females, certain morphometrics have potential to be useful, though due to the amounts of variation, small number of specimens, and the small differences between species, no others are claimed to be significant at this time (see Suppl. material 2). During evaluation of traditional PCA morphospace and three-dimensional PCA morphospace (PC1~PC2~PC3), males of *Aphonopelma mojave* separate in morphological space from *Aphonopelma iodius*, *Aphonopelma joshua*, and *Aphonopelma xwalxwal*, but do not separate from *Aphonopelma atomicum*, *Aphonopelma icenoglei*, and *Aphonopelma prenticei*. Female *Aphonopelma mojave* separate from *Aphonopelma iodius* in morphological space, but do not separate from *Aphonopelma atomicum*, *Aphonopelma icenoglei*, and *Aphonopelma prenticei*. There are no known female *Aphonopelma xwalxwal* at this time to compare. PC1, PC2, and PC3 explain ≥96% of the variation in all analyses.

### Aphonopelma paloma

Taxon classificationAnimaliaAraneaeTheraphosidae

Prentice, 1993

**Figure 105.**
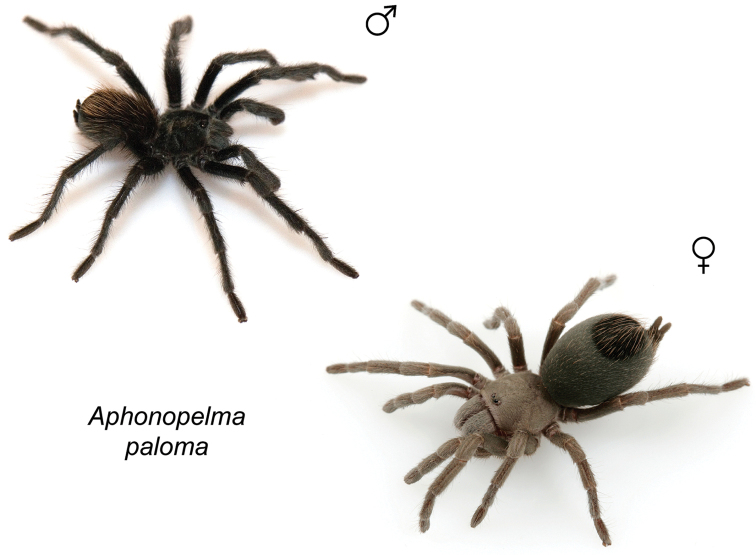
*Aphonopelma paloma* Prentice, 1993 specimens, live photographs. Male (L) - APH_1254; Female (R) - APH_3166.

**Figure 106.**
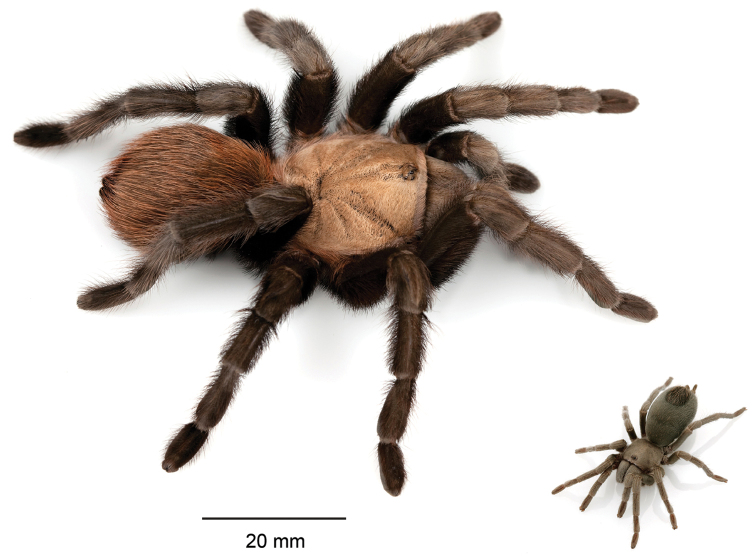
A generalized comparison of the largest species in the United States, an adult female *Aphonopelma anax*, and the smallest species in the United States, an adult female *Aphonopelma paloma*.

**Figure 107.**
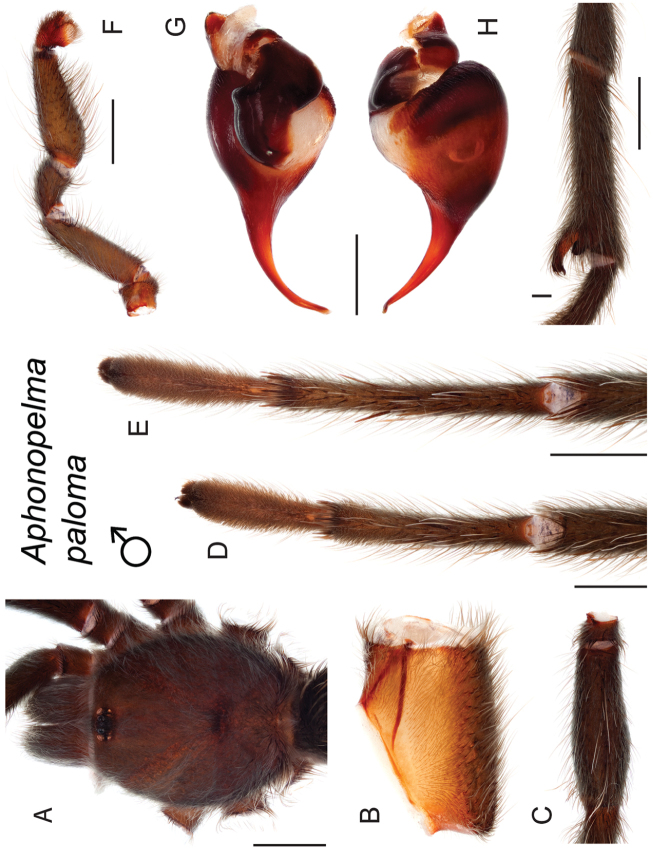
*Aphonopelma paloma* Prentice, 1993. **A–I** male specimen, APH_1603 **A** dorsal view of carapace, scale bar = 2mm **B** prolateral view of coxa I **C** dorsal view of femur III **D** ventral view of metatarsus III, scale bar = 1.5mm **E** ventral view of metatarsus IV, scale bar = 2mm **F** prolateral view of L pedipalp and palpal tibia, scale bar = 2mm **G** dorsal view of palpal bulb **H** retrolateral view of palpal bulb, scale bar = 0.5mm **I** prolateral view of tibia I (mating clasper), scale bar = 1.5mm.

**Figure 108.**
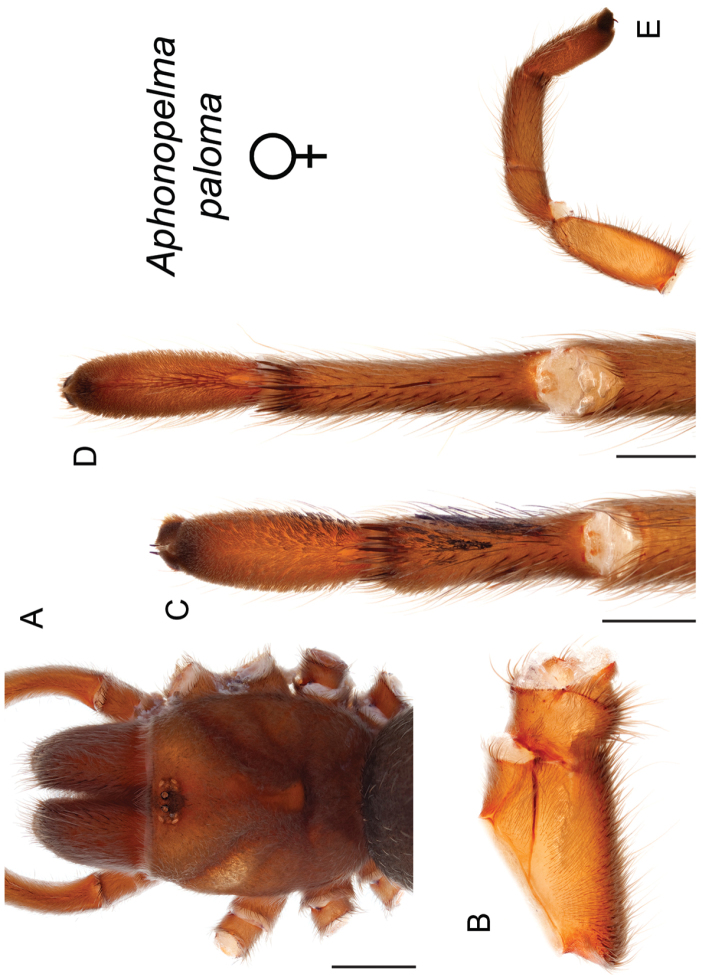
*Aphonopelma paloma* Prentice, 1993. **A–E** female specimen, APH_1255 **A** dorsal view of carapace, scale bar = 2mm **B** prolateral view of coxa I **C** ventral view of metatarsus III, scale bar = 1mm **D** ventral view of metatarsus IV, scale bar = 1mm **E** prolateral view of L pedipalp and palpal tibia.

**Figure 109.**
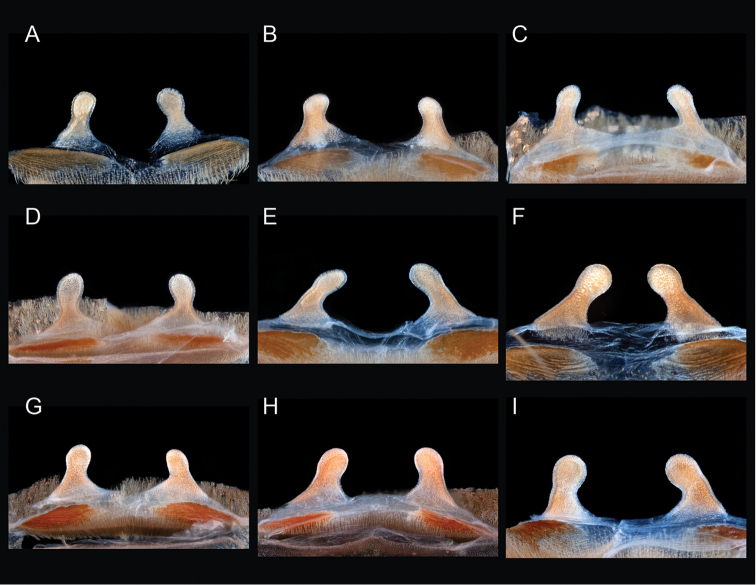
*Aphonopelma paloma* Prentice, 1993. **A–I** cleared spermathecae **A** *paloma* allotype **B** APH_0422 **C** APH_1102 **D** APH_1114 **E** APH_1115 **F** APH_1255 **G** APH_3170 **H** APH_3171 **I** APH_3172.

**Figure 110.**
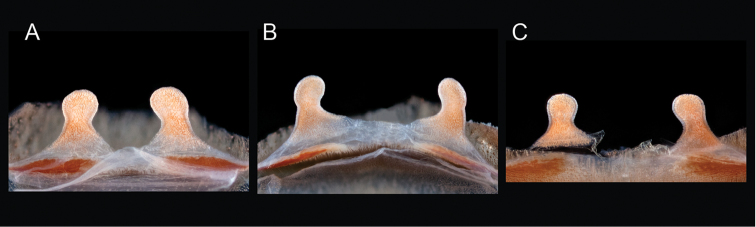
*Aphonopelma paloma* Prentice, 1993, cleared spermathecae. **A** APH_3189 **B** APH_3190 **C** APH_3194.

**Figure 111.**
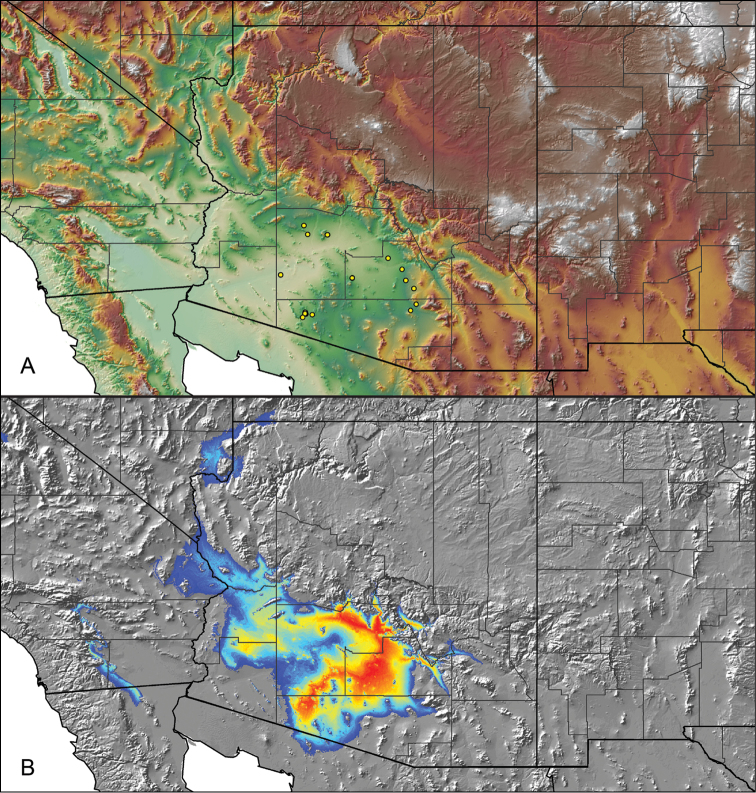
*Aphonopelma paloma* Prentice, 1993. **A** distribution of known specimens **B** predicted distribution; warmer colors (red, orange, yellow) represent areas of high probability of occurrence, cooler colors (blue shades) represent areas of low probability of occurrence.

Figures 105 , 106 , 107 , 108 , 109 , 110 , 111

Aphonopelma paloma Prentice, 1993: 189; male holotype and female allotype from 3 miles NE exit 151 off I-8 (jct with Hwy 84), Pinal Co., Arizona, 32.856167 -112.086609^2^, elev. 4310ft., 17-18.xi.1989, coll. Tom Prentice; deposited in AMNH. [examined]
Apachepelma paloma Smith, 1995: 45.
Aphonopelma paloma Prentice, 1997: 140.

#### Diagnosis.

*Aphonopelma paloma* (Fig. 105) is a member of the *paloma* species group and can be identified by a combination of morphological, molecular, and geographic characteristics. Nuclear and mitochondrial DNA identifies *Aphonopelma paloma* as a strongly supported monophyletic lineage (Figs 7–8) supported as the sister lineage to all other members of the *paloma* species group. *Aphonopelma paloma* is the smallest species in the United States (Fig. 106) and can easily be differentiated from syntopic species by its much smaller size and reduced extent of scopulation on metatarsi III and IV; it can be distinguished from other members of the *paloma* species group by locality. The most significant measurements that distinguish male *Aphonopelma paloma* from its closely related phylogenetic and syntopic species are M1 and the extent of scopulation of metatarsus III. Male *Aphonopelma paloma* can be distinguished by possessing a larger M1/T3 (≥1.11; 1.11–1.16) than *Aphonopelma parvum* sp. n. (≤1.09; 0.99–1.09), *Aphonopelma saguaro* sp. n. (≤1.05; 0.99–1.05), *Aphonopelma superstitionense* sp. n. (≤1.04; 0.98–1.04), and *Aphonopelma xwalxwal* sp. n. (≤1.11; 1.09–1.11); and by possessing a smaller L3 scopulation extent (21%-41%) than *Aphonopelma chalcodes* (75%-86%), *Aphonopelma mareki* sp. n. (50%–56%), and *Aphonopelma vorhiesi* (44%-62%). The most significant measurements that distinguish female *Aphonopelma paloma* from its closely related phylogenetic and syntopic species are Cl and the extent of scopulation on metatarsus IV. Female *Aphonopelma paloma* can be distinguished by possessing a smaller L4 scopulation extent (0–25%) than *Aphonopelma chalcodes* (63%–81%), *Aphonopelma parvum* (33%–42%), and *Aphonopelma vorhiesi* (26%–37%); and by possessing a larger Cl/M4 (≥1.44; 1.44–1.60) than *Aphonopelma superstitionense* (1.25 ± (only 1 specimen)). There are no significant measurements that separate female *Aphonopelma paloma* from *Aphonopelma mareki* or *Aphonopelma saguaro*.

#### Description.

Male and female originally described by Prentice (1993).

#### Redescription of male exemplar

(APH_1603; Fig. 107). *Specimen preparation and condition*: Specimen collected live wandering, preserved in 80% ethanol; deposited in AUMNH; original coloration faded due to preservation. Left legs I, III, IV, and left pedipalp removed for measurements and photographs; stored in vial with specimen. Right legs III & IV removed for DNA and stored at -80°C in the AUMNH (Auburn, AL). *General coloration*: Faded black or grey. *Cephalothorax*: Carapace 5.13 mm long, 4.73 mm wide; Very hirsute; densely clothed with grey pubescence mostly appressed to surface; fringe covered in long setae not closely appressed to surface; posterior region of carapace with long, stout setae; foveal groove medium deep and straight; pars cephalica region rises gradually from foveal groove to ocular area; AER very slightly procurved - mostly straight, PER straight; normal sized chelicerae; clypeus extends forward on a curve; LBl 0.732, LBw 0.878; sternum hirsute, clothed with grey, densely packed setae. *Abdomen*: Densely clothed in short black pubescence with numerous longer red/orange setae interspersed; possessing a dense dorsal patch of black Type I urticating bristles (Cooke et al. 1972) - smaller and distinct from large species. *Legs*: Hirsute; densely clothed in grey or faded black pubescence. Metatarsus I curved. F1 5.417; F1w 1.246; P1 2.083; T1 4.034; M1 4.058; A1 2.838; F3 4.565; F3w 1.74; P3 1.648; T3 3.51; M3 4.367; A3 2.884; F4 5.508; F4w 1.386; P4 1.733; T4 4.72; M4 5.454; A4 3.264; femur III is swollen. All tarsi fully scopulate. Extent of metatarsal scopulation: leg III (SC3) = 36.8%; leg IV (SC4) = 9.4%. Two ventral and two prolateral spinose setae on metatarsus III, with numerous medium stout setae throughout; five ventral spinose setae and one prolateral on metatarsus IV, with numerous medium stout setae throughout; two prolateral spinose setae on tibia I, one ventral spinose seta at the posterior margin of tibia I; one large megaspine is present at the apex on the retrolateral tibia of the mating clasper; one large megaspine at the apex of the retrolateral branch of the mating clasper. *Coxa I*: Prolateral surface covered by fine, hair-like setae. *Pedipalps*: Very hirsute, particularly ventrally; densely clothed in the same setal color as the other legs; one spinose seta near the anterior margin of the prolateral palpal femur; one spinose seta on the ventral palpal tibia, with numerous medium stout setae throughout; PTl 3.619, PTw 1.329. Palpal bulb is very short and stout; embolus tapers and gently curves to the retrolateral side, no keels; distinct dorsal and ventral transition from bulb to embolus.

**Variation (6).** Cl 5.126–5.502 (5.343±0.06), Cw 4.468–5.124 (4.824±0.1), LBl 0.675–0.797 (0.741±0.02), LBw 0.868–0.898 (0.878±0), F1 5.289–6.208 (5.723±0.15), F1w 1.176–1.402 (1.27±0.04), P1 1.741–2.44 (2.047±0.1), T1 4.034–5.248 (4.839±0.18), M1 3.62–4.297 (4.017±0.11), A1 2.785–2.876 (2.827±0.01), L1 length 18.374–20.949 (19.453±0.47), F3 4.466–4.958 (4.737±0.08), F3w 1.456–1.831 (1.673±0.06), P3 1.528–1.804 (1.694±0.04), T3 3.244–3.795 (3.532±0.09), M3 4.019–4.569 (4.332±0.09), A3 2.658–3.047 (2.837±0.06), L3 length 15.988–17.891 (17.131±0.33), F4 5.308–5.984 (5.703±0.11), F4w 1.112–1.445 (1.313±0.05), P4 1.532–2.102 (1.829±0.08), T4 4.578–5.096 (4.847±0.08), M4 5.412–6.102 (5.71±0.13), A4 3.042–3.374 (3.28±0.05), L4 length 20.357–22.272 (21.37±0.4), PTl 3.273–3.706 (3.53±0.07), PTw 1.159–1.431 (1.263±0.04), SC3 ratio 0.219–0.41 (0.309±0.03), SC4 ratio 0.056–0.245 (0.12±0.03), Coxa I setae = very thin tapered, F3 condition = swollen/slightly swollen.

#### Description of female exemplar

(APH_1255; Figs 108–110). *Specimen preparation and condition*: Specimen collected live from burrow, preserved in 80% ethanol; deposited in AUMNH; original coloration faded due to preservation. Left legs I, III, IV, and pedipalp removed for photographs and measurements; stored in vial with specimen. Right legs III & IV removed for DNA and stored at -80°C in the AUMNH (Auburn, AL). Genital plate with spermathecae removed and cleared, stored in vial with specimen. *General coloration*: Grey. *Cephalothorax*: Carapace 5.141 mm long, 4.393 mm wide; Hirsute, densely clothed with short grey pubescence closely appressed to surface, with short, stout setae throughout; fringe densely covered in slightly longer setae; foveal groove medium deep and procurved; pars cephalica region gently rises from thoracic furrow, arching anteriorly toward ocular area; AER very procurved, PER very slightly recurved; anterior margin of carapace broad, robust chelicerae, clypeus extends forward on a curve; LBl 0.807, LBw 0.963; sternum hirsute, clothed with short grey setae. *Abdomen*: Densely clothed dorsally in short black, faded black, and grey setae with longer, lighter setae (generally red or orange *in situ*) focused near the urticating patch; small but dense dorsal patch of black Type I urticating bristles (Cooke et al. 1972) - smaller and distinct from large species. *Spermathecae*: Paired and separate, short, very simple, with capitate bulbs and wide bases that are not fused. *Legs*: Hirsute; densely clothed in short grey pubescence; F1 4.192; F1w 1.303; P1 1.748; T1 3.323; M1 2.151; A1 2.074; F3 3.249; F3w 1.123; P3 1.463; T3 2.154; M3 2.25; A3 2.102; F4 4.301; F4w 1.181; P4 1.634; T4 3.248; M4 3.27; A4 2.408. All tarsi fully scopulate. Extent of metatarsal scopulation: leg III (SC3) = 29.1%; leg IV (SC4) = 9.7%. Three ventral spinose setae and one prolateral on metatarsus III, with numerous thicker setae throughout; three ventral spinose setae and one prolateral on metatarsus IV, with numerous thicker setae throughout. *Coxa I*: Prolateral surface covered by fine, hair-like setae. *Pedipalps*: Densely clothed in the same setal color as the other legs; two spinose setae on the anterior margin of prolateral/ventral tibia, with numerous thicker setae throughout.

**Variation (12).** Cl 4.635–7.058 (5.66±0.24), Cw 4.192–6.092 (5.023±0.21), LBl 0.763–1.197 (0.904±0.04), LBw 0.903–1.346 (1.074±0.04), F1 3.597–5.573 (4.52±0.19), F1w 1.186–1.884 (1.508±0.07), P1 1.494–2.461 (1.971±0.09), T1 2.968–4.516 (3.726±0.14), M1 1.911–3.189 (2.499±0.12), A1 1.927–2.705 (2.295±0.08), L1 length 11.984–18.261 (15.01±0.61), F3 2.973–4.507 (3.635±0.16), F3w 1.037–1.809 (1.346±0.07), P3 1.318–2.141 (1.687±0.08), T3 2.035–3.057 (2.523±0.1), M3 2.067–3.361 (2.607±0.13), A3 2.031–2.976 (2.401±0.09), L3 length 10.438–15.884 (12.853±0.55), F4 3.883–5.675 (4.735±0.18), F4w 1.088–1.864 (1.374±0.07), P4 1.521–2.33 (1.911±0.09), T4 3.135–4.529 (3.799±0.14), M4 3.083–4.612 (3.718±0.16), A4 2.308–3.263 (2.741±0.1), L4 length 13.93–20.232 (16.904±0.65), SC3 ratio 0.26–0.496 (0.392±0.02), SC4 ratio 0–0.247 (0.169±0.02), Coxa I setae = very thin tapered. Spermathecae variation can be seen in Figures 109–110.

#### Material examined.

**United States: Arizona: Maricopa**: 1.2 miles S Aqueduct on Aguila-Wickenburg Rd, 33.571504 -112.818108 ^1^, 1306ft., [APH_0432, 16/11/2008, 1 juv, Brent E. Hendrixson, AUMNH]; 4.65 miles N I-8 (Sentinel) on Agua Caliente Rd, 32.908339 -113.262179 ^1^, 584ft., [APH_0430, 16/11/2008, 1 juv, Brent E. Hendrixson, AUMNH]; just NW of Jct. Vulture Mine Rd and Aguila Rd, 33.719699 -112.8824 ^1^, 1716ft., [APH_1612-1613, 12/11/2012, 2♂, Brent E. Hendrixson, AUMNH]; just S of White Tank Mtn Rd, 33.56397 -112.49147 ^2^, 1360ft., [APH_1101-1103, 30/11/2009, 3 juv, June Olberding, AUMNH]; **Pima**: 0.75 miles S Ball Rd in Why along Hwy-85, 32.25235 -112.745206 ^1^, 1765ft., [APH_0424, 15/11/2008, 1 juv, Brent E. Hendrixson, AUMNH]; [APH_0426-0427, 15/11/2008, 1♀, 1♂, Brent E. Hendrixson, AUMNH]; Bates Well Rd, S of Ajo, 32.27002 -112.86087 ^2^, 1673ft., [APH_1114-1116, 15/12/2009, 2♀, 1♂, June Olberding, AUMNH]; in desert east of Bates Well Rd, about 7 miles W of Why, AZ, 32.28612 -112.85435 ^4^, 1720ft., [APH_0442-0449, 12/2008, 3♀, 5 juv, June Olberding, AUMNH]; Tucson area, near Catalina State Park, N side of Tangerine Rd and E of Tangerine Crossing, E of Marana, 32.42413 -111.03427 ^1^, 2631ft., [APH_3170-3172, 12/11/2013, 3♀, Chris A. Hamilton, Brent E. Hendrixson, AUMNH]; W of Why, 32.26683 -112.847 ^2^, 1650ft., [APH_1253-1258, 1/12/2010, 2♂, 4♀, June Olberding, AUMNH]; Saguaro National Park (West) - western portion, 32.32247 -111.12608 ^2^, 2790ft., [APH_1675, 1/12/14, 1♂, Derrick Smith, AUMNH; **Pinal**: 0.7 miles S Skyline Dr. on Quail Run Ln, 33.180864 -111.496516 ^2^, 1552ft., [APH_1671-1674, 6/12/2012, 4♀, June Olberding, Tim Cota, AUMNH]; in desert NW jct of Hwy-84 and Amarillo Valley Rd (3.2 miles NW I-8), 32.856511 -112.085564 ^1^, 1541ft., [APH_0421-0423, 15/11/2008, 3 juv, Brent E. Hendrixson, Paul Marek, Charity Hall, Kojun, AUMNH]; [APH_1603-1606, 10/11/2012, 3♂, 1♀, Brent E. Hendrixson, AUMNH]; off Florence-Kelvin Hwy, BLM land E of Florence, 32.99957 -111.26484 ^1^, 1968ft., [APH_3195, 15/11/2013, 1♀, Chris A. Hamilton, Brent E. Hendrixson, AUMNH]; [APH_3197-3198, 15/11/2013, 2♀, Chris A. Hamilton, Brent E. Hendrixson, AUMNH]; off Hwy 79 around 1/2 way between 77 split and Florence at Tom Mix Rest Area, 32.818271 -111.204751 ^1^, 2338ft., [APH_3194, 15/11/2013, 1♀, Brent E. Hendrixson, Chris A. Hamilton, AUMNH]; off Hwy 79, E side of road, N of Tucson past Hwy 77 split, near Ninety Six hills, 32.682347 -111.072531 ^1^, 2972ft., [APH_3189-3190, 14/11/2013, 2♀, Chris A. Hamilton, Brent E. Hendrixson, AUMNH]; W of Chuck’s Corner off S. Amarillo Valley Rd, off Hwy 84, N of I-8, 32.85665 -112.08419 ^1^, 1501ft., [APH_3166, 11/11/2013, 1♀, Brent E. Hendrixson, Chris A. Hamilton, AUMNH]; [APH_3168, 11/11/2013, 1 juv, Chris A. Hamilton, Brent E. Hendrixson, AUMNH].

#### Distribution and natural history.

*Aphonopelma paloma* is widely distributed across lower elevation sections of southern and southwestern Arizona (Figs 1G, 111) in the Sonoran Basin and Range Level III Ecoregion. *Aphonopelma paloma* can be found in syntopy with *Aphonopelma chalcodes* throughout its entire distribution and with *Aphonopelma vorhiesi* north of Tucson. The breeding season, when mature males abandon their burrows in search of females, is limited to late fall and early winter (November–December). Burrow entrances of *Aphonopelma paloma* are unique among species in the United States in that individuals frequently create a distinct crescent-shaped mound made from excavated soil and silk (Fig. 2F); this behavior has not been observed in other similar species (e.g., *Aphonopelma mareki*, *Aphonopelma parvum*, *Aphonopelma saguaro*, and *Aphonopelma superstitionense*). The natural history of *Aphonopelma paloma* is discussed in more detail by Prentice (1993).

#### Conservation status.

*Aphonopelma paloma* is abundant throughout its distribution but can be difficult to find due to the cryptic nature of their burrows and narrow window of activity during the year. This species is secure.

#### Remarks.

Other important ratios that distinguish *Aphonopelma paloma* males: possess a smaller L3 scopulation extent (21%–41%) than *Aphonopelma parvum* (60%–65%) and *Aphonopelma xwalxwal* (65%–95%); by possessing a larger PTl/M1 (≥0.81; 0.81–0.92) than *Aphonopelma chalcodes* (≤0.75; 0.67–0.75) and *Aphonopelma xwalxwal* (≤0.64; 0.57–0.64); by possessing a larger M3/A3 (≥1.46; 1.46–1.61) than *Aphonopelma mareki* (≤1.42; 1.27–1.42) and *Aphonopelma parvum* (≤1.41; 1.21–1.41), but smaller than *Aphonopelma xwalxwal* (≥1.66; 1.66–1.91); by possessing a larger Cl/M3 (≥1.17; 1.17–1.31) than *Aphonopelma superstitionense* (≤1.12; 1.05–1.12) and *Aphonopelma xwalxwal* (≤1.01; 0.91–1.01). Other important ratios that distinguish females: *Aphonopelma paloma* possess a larger Cl/A4 (≥1.92; 1.92–2.21) than *Aphonopelma parvum* (≤1.91; 1.63–1.91), but smaller than *Aphonopelma chalcodes* (≥2.32; 2.32–2.64); by possessing a smaller L3 scopulation extent (25%–50%) than *Aphonopelma chalcodes* (78%-93%), *Aphonopelma parvum* (62–67%), *Aphonopelma superstitionense* (64% ± (only 1 specimen)), and *Aphonopelma vorhiesi* (49%–69%, with slight overlap). For both males and females, certain morphometrics have potential to be useful, though due to the amounts of variation, small number of specimens, and the small differences between species, no others are claimed to be significant at this time (see Suppl. material 2). During evaluation of traditional PCA morphospace, males of *Aphonopelma paloma* separate in PCA morphological space from *Aphonopelma chalcodes*, *Aphonopelma vorhiesi*, *Aphonopelma parvum*, and *Aphonopelma xwalxwal* but do not separate from *Aphonopelma mareki*, *Aphonopelma saguaro*, and *Aphonopelma superstitionense*. Female *Aphonopelma paloma* separate from *Aphonopelma chalcodes*, *Aphonopelma mareki*, *Aphonopelma parvum*, *Aphonopelma saguaro*, *Aphonopelma superstitionense*, and *Aphonopelma vorhiesi* in morphological space. Interestingly, *Aphonopelma paloma* males separate from *Aphonopelma chalcodes*, *Aphonopelma parvum*, *Aphonopelma superstitionense*, and *Aphonopelma vorhiesi* in three-dimensional PCA morphospace (PC1~PC2~PC3), but do not separate from *Aphonopelma mareki* or *Aphonopelma saguaro*. *Aphonopelma paloma* females separate from *Aphonopelma chalcodes*, *Aphonopelma mareki*, *Aphonopelma parvum*, *Aphonopelma saguaro*, *Aphonopelma superstitionense*, and *Aphonopelma vorhiesi*. PC1, PC2, and PC3 explain ≥96% of the variation in all analyses.

### Aphonopelma parvum

Taxon classificationAnimaliaAraneaeTheraphosidae

Hamilton, Hendrixson & Bond sp. n.

http://zoobank.org/36D039B8-1D65-4312-A5CD-0A96F0C77061

**Figure 112.**
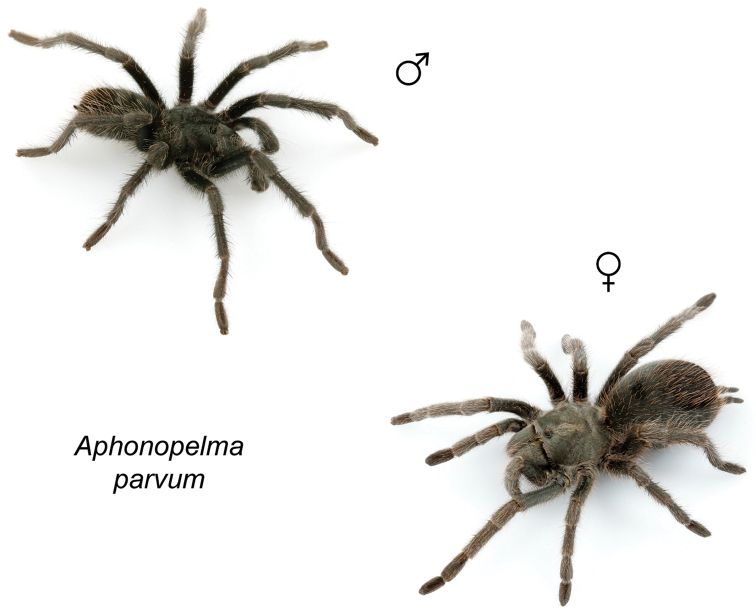
*Aphonopelma parvum* sp. n. live photographs. Male (L) - APH_3181; Female (R) - APH_3185.

**Figure 113.**
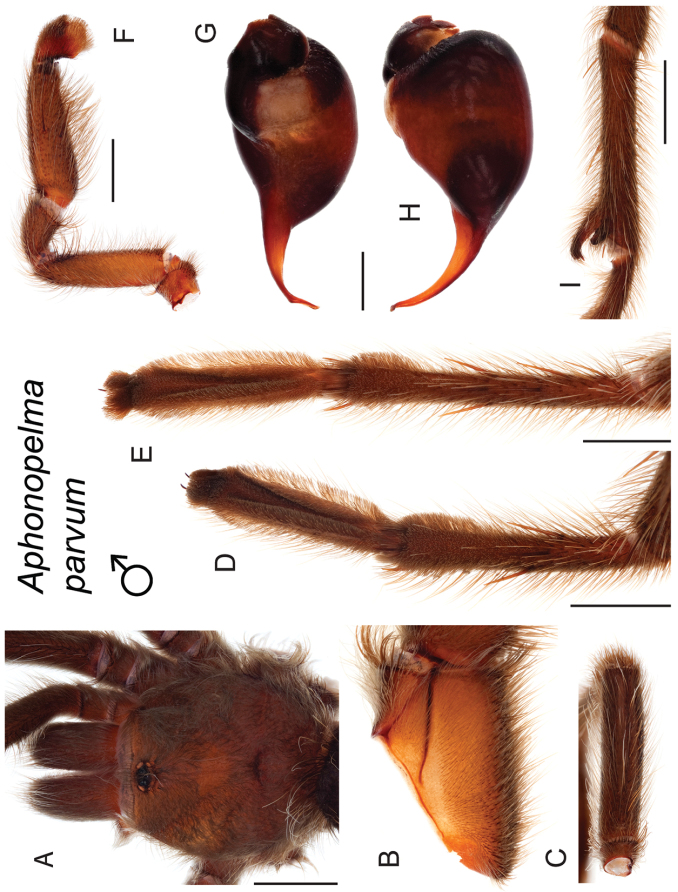
*Aphonopelma parvum* sp. n. **A–I** male holotype, APH_1264 **A** dorsal view of carapace, scale bar = 3mm **B** prolateral view of coxa I **C** dorsal view of femur III **D** ventral view of metatarsus III, scale bar = 2mm **E** ventral view of metatarsus IV, scale bar = 2.5mm **F** prolateral view of L pedipalp and palpal tibia, scale bar = 2mm **G** dorsal view of palpal bulb **H** retrolateral view of palpal bulb, scale bar = 0.5mm **I** prolateral view of tibia I (mating clasper), scale bar = 3mm.

**Figure 114.**
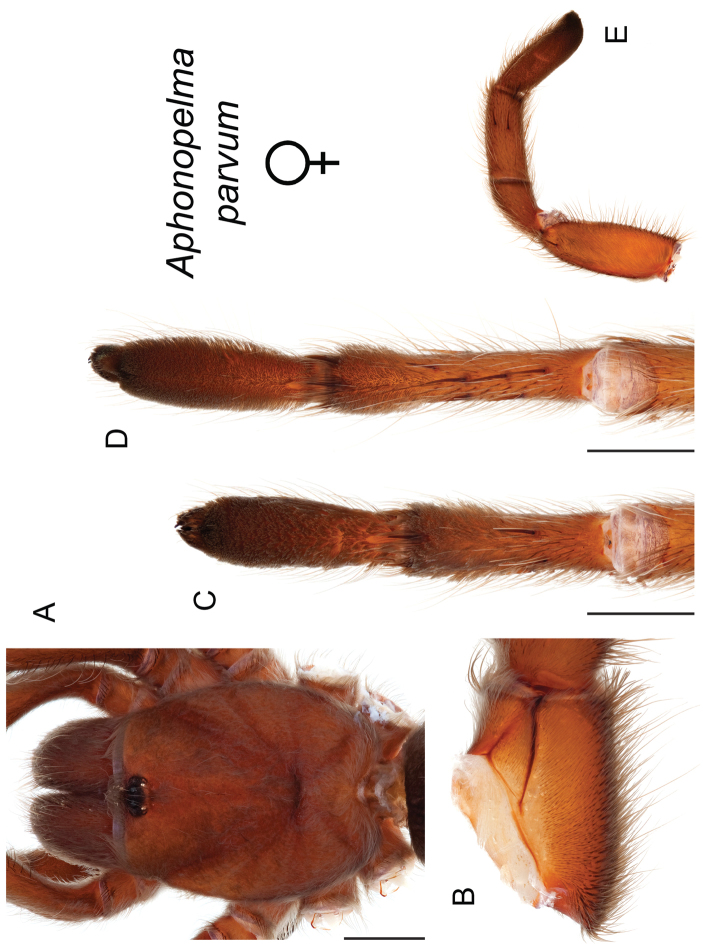
*Aphonopelma parvum* sp. n. **A–E** female paratype, APH_1622 **A** dorsal view of carapace, scale bar = 2.5mm **B** prolateral view of coxa I **C** ventral view of metatarsus III, scale bar = 1.5mm **D** ventral view of metatarsus IV, scale bar = 2mm **E** prolateral view of L pedipalp and palpal tibia.

**Figure 115.**
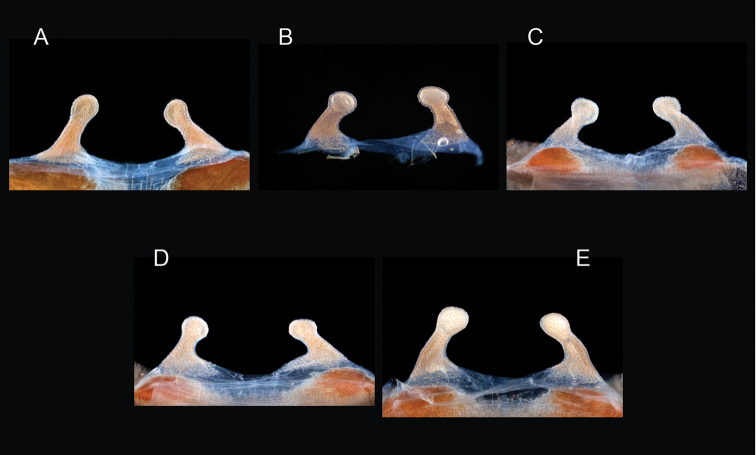
*Aphonopelma parvum* sp. n. **A–E** cleared spermathecae **A** APH_1104 **B** APH_1109 **C** APH_1599 **D** APH_1600 **E** APH_1622.

**Figure 116.**
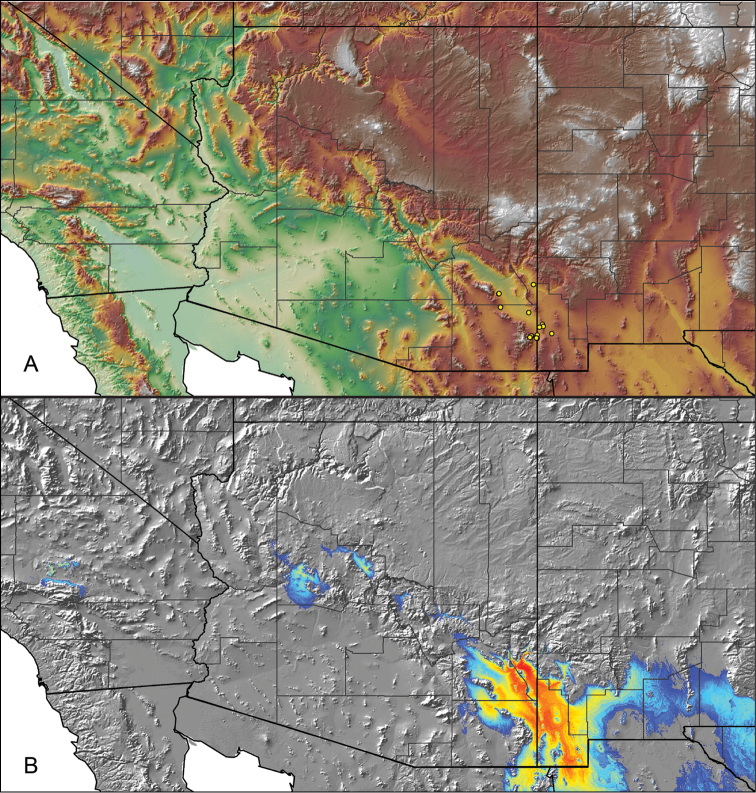
*Aphonopelma parvum* sp. n. **A** distribution of known specimens **B** predicted distribution; warmer colors (red, orange, yellow) represent areas of high probability of occurrence, cooler colors (blue shades) represent areas of low probability of occurrence.

Figures 112 , 113 , 114 , 115 , 116

#### Types.

Male holotype (APH_1264) collected along CR-C078, Hidalgo Co., New Mexico, 32.0679 -108.94608 ^2^, elev. 4296ft., 6.xii.2010, coll. June Olberding; deposited in AUMNH. Paratype female (APH_1622) from 0.2 miles N Hwy-533 (Portal Rd) on Stateline Rd., Hidalgo Co., New Mexico, 31.874121 -109.047377 ^1^, elev. 4107ft., 14.xi.2012, coll. Brent E. Hendrixson; deposited in AUMNH. Paratype male (APH_1106) from 0.11 miles N Portal Rd (Hwy 533) on State Line Rd, Hidalgo Co., New Mexico, 31.87232 -109.04787 ^2^, elev. 4127ft., 28.xi.2009, coll. June Olberding; deposited in AMNH. Paratype female (APH_1600) from Tanque Rd., Graham Co., Arizona, 32.606785 -109.680286 ^1^, elev. 4158ft., 9.xi.2012, coll. Brent E. Hendrixson; deposited in AMNH.

#### Etymology.

The specific epithet is a neuter adjective for “very small” or “very little” and is in reference to the diminutive size of this new tarantula species.

#### Diagnosis.

*Aphonopelma parvum* (Fig. 112) is a member of the *paloma* species group and can be distinguished by a combination of morphological, molecular, and geographic characteristics. Nuclear and mitochondrial DNA identifies *Aphonopelma parvum* as a strongly supported monophyletic lineage (Figs 7–8) that is a sister lineage to *Aphonopelma mareki* sp. n., *Aphonopelma saguaro* sp. n., and *Aphonopelma superstitionense* sp. n. *Aphonopelma parvum* can easily be differentiated from syntopic populations of *Aphonopelma chalcodes*, *Aphonopelma gabeli*, *Aphonopelma hentzi*, and *Aphonopelma vorhiesi* by their much smaller size, and can be differentiated from other members of the *paloma* species group by locality. Importantly, *Aphonopelma parvum* males do not possess a swollen femur III as is seen in *Aphonopelma paloma*, *Aphonopelma saguaro*, *Aphonopelma superstitionense*, and some specimens of *Aphonopelma mareki*. The most significant measurements that distinguish male *Aphonopelma parvum* from its closely related phylogenetic and syntopic species are Cl and the extent of scopulation on metatarsi III and IV. Male *Aphonopelma parvum* can be distinguished by possessing a smaller Cl/PTw (≤4.22; 3.79–4.22) than *Aphonopelma chalcodes* (≥5.06; 5.06–6.05), *Aphonopelma gabeli* (≥5.93; 5.93–6.56), *Aphonopelma hentzi* (≥4.87; 4.87–6.16), and *Aphonopelma vorhiesi* (≥4.61; 4.61–5.58); a larger L3 scopulation extent (60%–65%) than *Aphonopelma mareki* (50%–56%); and a larger L4 scopulation extent (36%-44%) than *Aphonopelma paloma* (5%–24%), *Aphonopelma saguaro* (14%–26%), and *Aphonopelma superstitionense* (14%–20%). The most significant measurements that distinguish female *Aphonopelma parvum* from its closely related phylogenetic and syntopic species are F3 and the extent of scopulation on metatarsus IV. Female *Aphonopelma parvum* can be distinguished by possessing a smaller F3/A4 (≤1.28; 1.21–1.28) than *Aphonopelma chalcodes* (≥1.44; 1.44–1.80), *Aphonopelma gabeli* (≥1.37; 1.37–1.65), *Aphonopelma hentzi* (≥1.36; 1.36–1.66), and *Aphonopelma vorhiesi* (≥1.37; 1.37–1.63); and a larger L4 scopulation extent (33%–42%) than *Aphonopelma chiricahua* sp. n. (28% ± (only 1 specimen)), *Aphonopelma mareki* (21%–27%), *Aphonopelma paloma* (0–25%), *Aphonopelma saguaro* (21% ± (only 1 specimen)), and *Aphonopelma superstitionense* (26% ± (only 1 specimen)).

#### Description of male holotype

(APH_1264; Fig. 113). *Specimen preparation and condition*: Specimen collected wandering and preserved in 80% ethanol; original coloration faded due to preservation. Left legs I, III, IV, and left pedipalp removed for measurements and photographs; stored in vial with specimen. Right legs III & IV removed for DNA and stored at -80°C in the AUMNH (Auburn, AL). *General coloration*: Faded black/brown. *Cephalothorax*: Carapace 6.544 mm long, 6.019 mm wide; densely clothed with faded pubescence, appressed to surface; fringe covered in long setae not closely appressed to surface, hirsute appearance; foveal groove medium deep and slightly recurved; pars cephalica region rises very gradually from foveal groove on a straight plane towards the ocular area; AER slightly procurved, PER recurved; normal sized chelicerae; clypeus extends forward on a slight curve; LBl 0.829, LBw 0.989; sternum hirsute, clothed with faded, densely packed, short setae. *Abdomen*: Densely clothed in short black/brown pubescence with numerous longer, lighter setae interspersed (generally red or orange *in situ*); dense dorsal patch of black Type I urticating bristles (Cooke et al. 1972) - larger than other miniature species. *Legs*: Hirsute; densely clothed in faded pubescence. Metatarsus I curved. F1 7.966; F1w 1.529; P1 2.427; T1 6.919; M1 4.861; A1 4.182; F3 6.228; F3w 1.649; P3 2.157; T3 4.869; M3 5.41; A3 4.143; F4 7.436; F4w 1.461; P4 2.297; T4 6.65; M4 6.86; A4 4.963; femur III is normal - not noticeably swollen or wider than other legs. All tarsi fully scopulate. Extent of metatarsal scopulation: leg III (SC3) = 61.4%; leg IV (SC4) = 40.4%. Three ventral spinose setae on metatarsus III; six ventral spinose setae on metatarsus IV; one prolateral and two ventral spinose setae on tibia I; one megaspine present on the retrolateral tibia, at the apex of the mating clasper; one megaspine on the apex on the retrolateral branch of the tibial apophyses. *Coxa I*: Prolateral surface covered by fine, hair-like setae. *Pedipalps*: Hirsute; densely clothed in the same setal color as the other legs, with numerous longer ventral setae; one spinose seta at the apical, prolateral femur and three prolateral spinose setae on the palpal tibia; PTl 4.618, PTw 1.664. Palpal bulb is very short and stout. When extended, embolus tapers with a curve to the retrolateral side; embolus slender, no keels; distinct dorsal and ventral transition from bulb to embolus.

**Variation (7).** Cl 5.702–7.057 (6.333±0.19), Cw 5.141–6.376 (5.836±0.2), LBl 0.764–0.889 (0.829±0.02), LBw 0.888–0.989 (0.958±0.01), F1 6.212–7.966 (7.063±0.25), F1w 1.312–1.704 (1.54±0.05), P1 1.967–2.597 (2.35±0.09), T1 5.269–6.919 (6.012±0.21), M1 3.837–4.863 (4.483±0.16), A1 3.282–4.182 (3.593±0.13), L1 length 20.633–26.355 (23.501±0.76), F3 4.964–6.228 (5.631±0.2), F3w 1.305–1.865 (1.637±0.08), P3 1.806–2.173 (2.025±0.05), T3 3.791–4.869 (4.318±0.14), M3 4.408–5.41 (4.817±0.14), A3 3.141–4.143 (3.758±0.13), L3 length 18.197–22.807 (20.548±0.63), F4 6.055–7.436 (6.741±0.2), F4w 1.226–1.634 (1.453±0.06), P4 2.085–2.476 (2.214±0.05), T4 5.104–6.65 (5.772±0.2), M4 5.78–6.86 (6.336±0.18), A4 3.915–4.963 (4.374±0.13), L4 length 23.105–28.206 (25.438±0.71), PTl 3.619–4.618 (4.139±0.13), PTw 1.377–1.673 (1.567±0.05), SC3 ratio 0.601–0.649 (0.63±0.01), SC4 ratio 0.368–0.442 (0.399±0.01), Coxa I setae = very thin tapered, F3 condition = normal.

#### Description of female paratype

(APH_1622; Figs 114–115). *Specimen preparation and condition*: Specimen collected live from burrow, preserved in 80% ethanol; original coloration faded due to preservation. Left legs I, III, IV, and pedipalp removed for photographs and measurements; stored in vial with specimen. Right legs III & IV removed for DNA and stored at -80°C in the AUMNH (Auburn, AL). Genital plate with spermathecae removed and cleared, stored in vial with specimen. *General coloration*: Faded black/brown. *Cephalothorax*: Carapace 7.147 mm long, 6.51 mm wide; Hirsute, densely clothed with short faded black/brown pubescence closely appressed to surface; fringe densely covered in slightly longer setae; foveal groove medium deep and slightly procurved; pars cephalica region gently rises from thoracic furrow, arching anteriorly toward ocular area; AER very slightly procurved - mostly straight, PER slightly recurved; chelicerae robust, clypeus extends on a slight curve; LBl 1.136, LBw 1.271; sternum hirsute, clothed with short faded setae. *Abdomen*: Densely clothed dorsally in short faded black setae with longer, lighter setae (generally red or orange *in situ*) focused near the urticating patch; dense dorsal patch of black Type I urticating bristles (Cooke et al. 1972) - larger than other miniature species. *Spermathecae*: Paired and separate, with capitate bulbs, narrow necks widening towards the bases; not fused. *Legs*: Hirsute; densely clothed in short faded black/brown pubescence; F1 6.016; F1w 1.935; P1 2.504; T1 4.616; M1 2.927; A1 3.144; F3 4.951; F3w 1.674; P3 2.168; T3 3.466; M3 3.703; A3 3.027; F4 6.277; F4w 1.733; P4 2.396; T4 5.001; M4 4.984; A4 4.017. All tarsi fully scopulate. Extent of metatarsal scopulation: leg III (SC3) = 64.2%; leg IV (SC4) = 40.4%. Two ventral spinose setae on metatarsus III; six ventral spinose setae and one retrolateral spinose seta on metatarsus IV. *Coxa I*: Prolateral surface covered by very thin tapered and fine, hair-like setae. *Pedipalps*: Densely clothed in the same setal color as the other legs; one spinose seta on the apical, prolateral femur, four prolateral (two at the apical, prolateral border with the tarsus) spinose setae and one ventral spinose seta on the tibia.

**Variation (5).** Cl 6.181–7.326 (6.974±0.21), Cw 5.827–6.86 (6.28±0.2), LBl 0.964–1.136 (1.074±0.03), LBw 1.072–1.295 (1.206±0.04), F1 5.344–6.154 (5.849±0.14), F1w 1.777–2.043 (1.896±0.05), P1 2.291–2.693 (2.453±0.07), T1 4.492–5.026 (4.721±0.1), M1 2.927–3.487 (3.099±0.1), A1 2.777–3.283 (3.027±0.09), L1 length 18.064–20.322 (19.149±0.4), F3 4.621–5.077 (4.851±0.1), F3w 1.515–1.742 (1.646±0.04), P3 2.127–2.267 (2.168±0.03), T3 3.192–3.467 (3.369±0.05), M3 3.221–3.703 (3.44±0.09), A3 3.027–3.573 (3.227±0.1), L3 length 16.258–17.782 (17.056±0.29), F4 5.735–6.537 (6.147±0.15), F4w 1.598–1.764 (1.696±0.03), P4 2.255–2.544 (2.383±0.05), T4 4.539–5.438 (4.98±0.16), M4 4.592–5.153 (4.942±0.1), A4 3.603–4.035 (3.886±0.08), L4 length 21.215–23.343 (22.339±0.46), SC3 ratio 0.629–0.672 (0.651±0.01), SC4 ratio 0.338–0.422 (0.397±0.02), Coxa I setae = very thin tapered. Spermathecae variation can be seen in Figure 115.

#### Material examined.

**United States: Arizona: Cochise**: 0.8 miles SE Hwy-191 on connecting road, 32.384654 -109.654269 ^1^, 4231ft., [APH_1619, 14/11/2012, 1♀, Brent E. Hendrixson, AUMNH]; Cave Creek, 1 mile south of Ranger Station, 31.888981 -109.16895 ^4^, 5067ft., [AUMS_2247, 28/11/1990, 1♂, Barney Tomberlin, AUMNH]; on Portal Rd going towards Cave Creek Canyon, 31.91472 -109.146262 ^1^, 4763ft., [APH_3187, 13/11/2013, 1♂, Chris A. Hamilton, Brent E. Hendrixson, AUMNH]; on Portal Rd, just past the state line, 31.87769 -109.060587 ^1^, 4195ft., [APH_3186, 13/11/2013, 1♂, Chris A. Hamilton, Brent E. Hendrixson, AUMNH]; Portal, 31.913703 -109.14145 ^5^, 4770ft., [APH_2209, 20/11/1963, 1♂, V. Roth, AMNH]; [APH_2210, 1/12/1964, 1♂, V. Roth, AMNH]; San Simon, E of Kennedy Rd, 32.296735 -109.18536 ^1^, 3703ft., [APH_1620, 14/11/2012, 1♀, Brent E. Hendrixson, AUMNH]; **Graham**: 0.25 miles E Hwy-191 on Tanque Rd, 32.605235 -109.682418 ^1^, 3873ft., [APH_1350, 5/8/2011, 1 juv, Brent E. Hendrixson, Brendon Barnes, Nate Davis, Jake Storms, AUMNH]; 0.4 miles E Hwy-191 on Tanque Rd, 32.606204 -109.681524 ^1^, 3891ft., [APH_1183, 25/7/2010, 1 juv, Brent E. Hendrixson, Brendon Barnes, Nate Davis, AUMNH]; off Tanque Rd, off Hwy 191 to Safford, near Swift Trail Junction, 32.60887 -109.678738 ^1^, 3833ft., [APH_3178-3180, 13/11/2013, 2♀, 1♂, Chris A. Hamilton, Brent E. Hendrixson, AUMNH]; Tanque Rd, 32.606785 -109.680286 ^1^, 4158ft., [APH_1599-1601, 9/11/2012, 3♀, Brent E. Hendrixson, AUMNH & AMNH]; [APH_1623, 14/11/2012, 1♀, Brent E. Hendrixson, AUMNH]; **Greenlee**: 0.4 miles N Hwy-75 on Goat Camp Rd, 32.755403 -109.110492 ^1^, 3726ft., [APH_1354, 5/8/2011, 1 juv, Brent E. Hendrixson, Brendon Barnes, Nate Davis, Jake Storms, AUMNH]; **New Mexico: Hidalgo**: 0.11 miles N Portal Rd (Hwy-533) on State Line Rd, 31.87232 -109.04787 ^2^, 4127ft., [APH_1106, 28/11/2009, 1♂, June Olberding, AMNH]; [APH_1109, 28/11/2009, 1♀, June Olberding, AUMNH]; 0.18 miles N Portal Rd (Hwy-533) on State Line Rd, 31.87333 -109.04758 ^2^, 4129ft., [APH_1105, 28/11/2009, 1 juv, June Olberding, AUMNH]; 0.19 miles N Portal Rd (Hwy-533) on State Line Rd, 31.87347 -109.04763 ^2^, 4130ft., [APH_1111, 28/11/2009, 1 juv, June Olberding, AUMNH]; 0.2 miles N Hwy-533 (Portal Rd) on Stateline Rd, 31.874121 -109.047377 ^1^, 4107ft., [APH_1596-1598, 8/11/2012, 3♀, Brent E. Hendrixson, AUMNH]; [APH_1621-1622, 14/11/2012, 1♂, 1♀, Brent E. Hendrixson, AUMNH]; 0.2 miles NW Hwy-80 on CR-C078, 32.1046 -108.96062 ^2^, 4493ft., [APH_1260-1261, 4/12/2010, 2♂, June Olberding, AUMNH]; [APH_1265-1266, 4/12/2010, 2♀, June Olberding, AUMNH]; 0.20 miles N Portal Rd (Hwy-533) on State Line Rd, 31.8737 -109.04763 ^2^, 4130ft., [APH_1108, 28/11/2009, 1♀, June Olberding, AUMNH]; 0.21 miles N Portal Rd (Hwy-533) on State Line Rd, 31.8738 -109.04768 ^2^, 4130ft., [APH_1110, 28/11/2009, 1♀, June Olberding, AUMNH]; 0.22 miles N Portal Rd (Hwy-533) on State Line Rd, 31.87392 -109.04793 ^2^, 4131ft., [APH_1104, 28/11/2009, 1♀, June Olberding, AUMNH]; [APH_1107, 28/11/2009, 1 juv, June Olberding, AUMNH]; 0.5 miles W Hwy-80 on road near Rusty’s RV Ranch, 31.92755 -109.0443 ^2^, 4170ft., [APH_1259, 5/12/2010, 1♂, June Olberding, AUMNH]; [APH_1262-1263, 5/12/2010, 2♂, June Olberding, AUMNH]; [APH_1267, 5/12/2010, 1♀, June Olberding, AUMNH]; along CR-C078, 32.0679 -108.94608 ^2^, 4296ft., [APH_1264, 6/12/2010, 1♂, June Olberding, AUMNH]; [APH_1268-1269, 6/12/2010, 2♀, June Olberding, AUMNH]; Animas, 31.949 -108.8073 ^2^, 4400ft., [APH_0794, 14/10/2009, 1♀, Clyde Mahan, AUMNH]; off State Line Rd, off Portal Rd, 31.873052 -109.048122 ^1^, 4134ft., [APH_3183-3185, 13/11/2013, 1♂, 2♀, Brent E. Hendrixson, Chris A. Hamilton, AUMNH]; S on Hwy 80, S of Granite Gap, 32.054494 -109.009828 ^1^, 4038ft., [APH_3181-3182, 13/11/2013, 2♂, Chris A. Hamilton, Brent E. Hendrixson, AUMNH].

#### Distribution and natural history.

*Aphonopelma parvum* can be found inhabiting the Chihuahuan Desert and Madrean Archipelago Level III Ecoregions of southeastern Arizona and southwestern New Mexico (Figs 1F & 116). Due to its distribution that is in close proximity to the Cochise Filter Barrier (an important biogeographic region where biota from different ecoregions converge), *Aphonopelma parvum* is syntopic with several other tarantula species including *Aphonopelma chalcodes*, *Aphonopelma chiricahua*, *Aphonopelma gabeli*, *Aphonopelma hentzi*, and *Aphonopelma vorhiesi*. The breeding season, when mature males abandon their burrows in search of females, is limited to late fall and early winter (November–December). Excavated soil near burrow entrances is generally haphazardly scattered and not arranged into a distinct crescent-shaped mound or turret.

#### Conservation status.

*Aphonopelma parvum* is very common throughout their distribution but can be difficult to find due to the cryptic nature of their burrows and narrow window of activity during the year. This species is likely secure.

#### Remarks.

Other important ratios that distinguish males: *Aphonopelma parvum* possess a larger F1/M3 (≥1.40; 1.40–1.53) than *Aphonopelma chalcodes* (≤1.31; 1.13–1.31), *Aphonopelma gabeli* (≤1.24; 1.18–1.24), *Aphonopelma hentzi* (≤1.40; 1.20–1.40), *Aphonopelma paloma* (≤1.38; 1.24–1.38), *Aphonopelma saguaro* (≤1.37; 1.22–1.37), and *Aphonopelma superstitionense* (≤1.33; 1.22–1.33). Other important ratios that distinguish *Aphonopelma parvum* females: *Aphonopelma parvum* possess a larger F3L/W (≥2.85; 2.85–3.05) than *Aphonopelma saguaro* (2.41 ± (only 1 specimen)); a smaller P1/F3 (≤0.54; 0.47–0.54) than *Aphonopelma chiricahua* (0.69 ± (only 1 specimen)) and *Aphonopelma superstitionense* (0.58 ± (only 1 specimen)); a smaller Cl/A4 (≤1.91; 1.63–1.91) than *Aphonopelma paloma* (≥1.92; 1.92–2.21); a smaller Cl/F4 (≤1.17; 1.07–1.17) than *Aphonopelma gabeli* (≥1.20; 1.20–1.32), *Aphonopelma hentzi* (≥1.20; 1.20–1.40), and *Aphonopelma vorhiesi* (≥1.23; 1.23–1.37); a smaller L4 scopulation extent than *Aphonopelma chalcodes* (56%–81%). Certain morphometrics have potential to be useful, though due to the amounts of variation, small number of specimens, and the small differences between species, no other are claimed to be significant at this time (see Suppl. material 2). During evaluation of traditional two-dimensional PCA morphospace and three-dimensional PCA morphospace (PC1~PC2~PC3), males of *Aphonopelma parvum* separate from *Aphonopelma chalcodes*, *Aphonopelma gabeli*, *Aphonopelma hentzi*, *Aphonopelma paloma*, *Aphonopelma saguaro*, *Aphonopelma superstitionense*, and *Aphonopelma vorhiesi* along PC1~2, but do not separate from *Aphonopelma chiricahua* or *Aphonopelma mareki*. Male *Aphonopelma parvum* separate from all compared species, except *Aphonopelma mareki*, in three-dimensional morphospace. Female *Aphonopelma parvum* separate from *Aphonopelma chalcodes*, *Aphonopelma gabeli*, *Aphonopelma hentzi*, *Aphonopelma paloma*, and *Aphonopelma vorhiesi*, but do not separate from *Aphonopelma chiricahua*, *Aphonopelma mareki*, *Aphonopelma saguaro*, or *Aphonopelma superstitionense* in two-dimensional morphological space, yet when three-dimensional morphospace is plotted, *Aphonopelma parvum* separates from all compared species. PC1, PC2, and PC3 explain ≥97% of the variation in all analyses.

### Aphonopelma peloncillo

Taxon classificationAnimaliaAraneaeTheraphosidae

Hamilton, Hendrixson & Bond sp. n.

http://zoobank.org/697A075D-4358-4D48-B2F4-507618BD9E29

**Figure 117.**
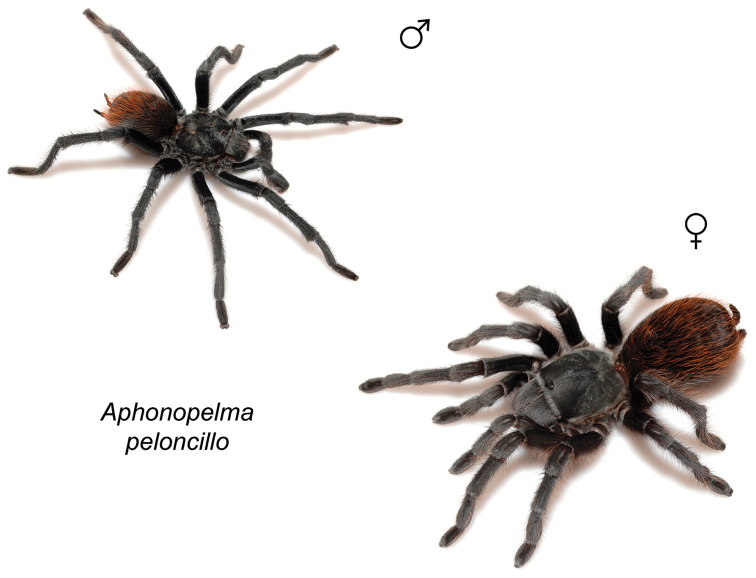
*Aphonopelma peloncillo* sp. n. live photographs. Male (L) - APH_1300; Female (R) - APH_1296.

**Figure 118.**
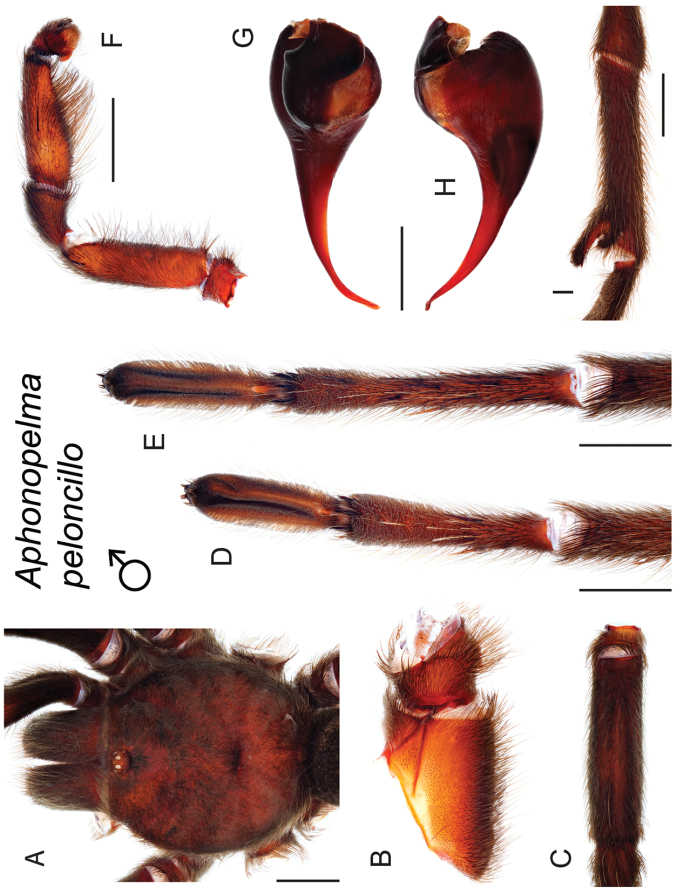
*Aphonopelma peloncillo* sp. n. **A–I** male holotype, APH_0672 **A** dorsal view of carapace, scale bar = 4mm **B** prolateral view of coxa I **C** dorsal view of femur III **D** ventral view of metatarsus III, scale bar = 3.5mm **E** ventral view of metatarsus IV, scale bar = 4mm **F** prolateral view of L pedipalp and palpal tibia, scale bar = 5mm **G** dorsal view of palpal bulb **H** retrolateral view of palpal bulb, scale bar = 1mm **I** prolateral view of tibia I (mating clasper), scale bar = 4mm.

**Figure 119.**
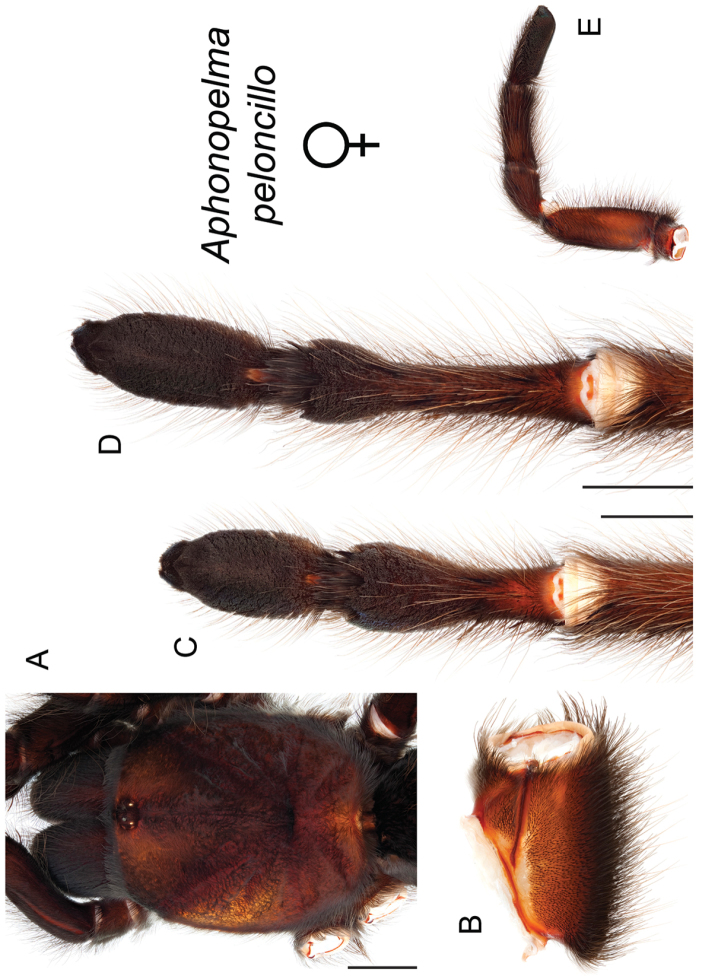
*Aphonopelma peloncillo* sp. n. **A–E** female paratype, APH_1296 **A** dorsal view of carapace, scale bar = 5.5mm **B** prolateral view of coxa I **C** ventral view of metatarsus III, scale bar = 3.5mm **D** ventral view of metatarsus IV, scale bar = 4mm **E** prolateral view of L pedipalp and palpal tibia.

**Figure 120.**
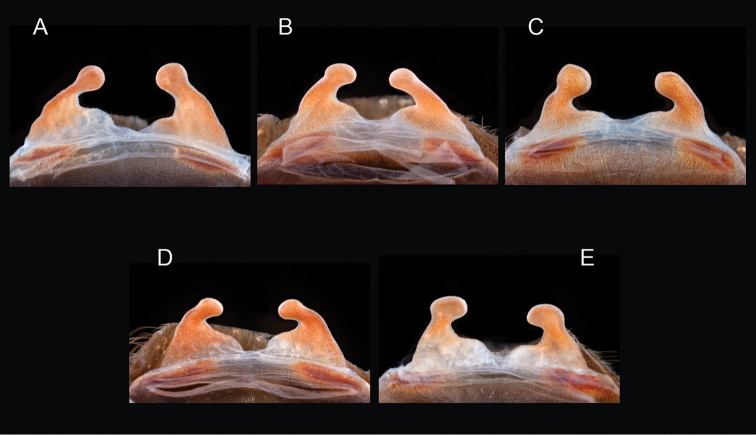
*Aphonopelma peloncillo* sp. n. **A–E** cleared spermathecae **A** APH_1296 **B** APH_0681 **C** APH_0683 **D** APH_1181 **E** APH_1297.

**Figure 121.**
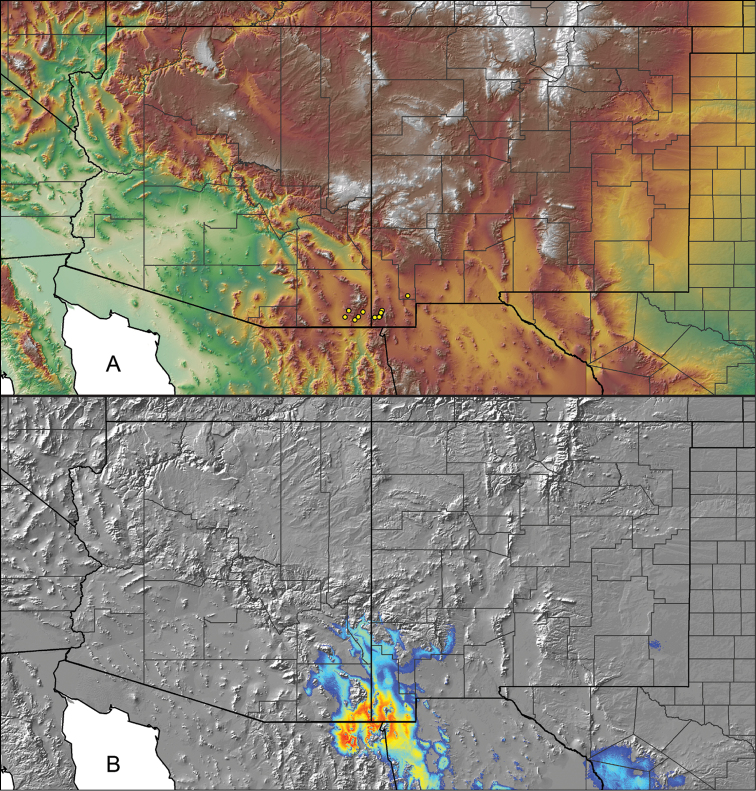
*Aphonopelma peloncillo* sp. n. **A** distribution of known specimens **B** predicted distribution; warmer colors (red, orange, yellow) represent areas of high probability of occurrence, cooler colors (blue shades) represent areas of low probability of occurrence.

Figures 117 , 118 , 119 , 120 , 121 ; Suppl. material 4

#### Types.

Male holotype (APH_0672) from along Hwy-338, Hidalgo Co., New Mexico, 31.607079 -108.867081 ^1^, elev. 4934ft., 14.vii.2009, coll. Brent E. Hendrixson and Nate Davis; deposited in AUMNH. Paratype female (APH_1296) from Coronado National Forest, Peloncillo Mtns, Clanton Draw Area, Hidalgo Co., New Mexico, 31.518665 -108.982597 ^1^, elev. 5435ft., 25.vii.2011, coll. Brent E. Hendrixson, Brendon Barnes, Nate Davis, Jake Storms; deposited in AUMNH. Paratype male (APH_1190) from along Hwy-338, Hidalgo Co., New Mexico, 31.607079 -108.867081 ^1^, elev. 4934ft., 26.vii.2010, coll. Brent E. Hendrixson, Brendon Barnes, Nate Davis; deposited in AMNH. Paratype female (APH_1181) from Coronado National Forest, Peloncillo Mtns, Hidalgo Co., New Mexico, 31.518665 -108.982597 ^1^, elev. 5435ft., 26.vii.2010, coll. Brent E. Hendrixson, Brendon Barnes, Nate Davis, Jake Storms; deposited in AMNH.

#### Etymology.

The specific epithet is a noun in apposition taken from type locality, the Peloncillo Mountains in southwestern New Mexico, where this species was first discovered.

#### Diagnosis.

*Aphonopelma peloncillo* (Fig. 117) is a member of the *Marxi* species group and can be distinguished by a combination of morphological, molecular, and geographic characteristics. Nuclear and mitochondrial DNA identifies *Aphonopelma peloncillo* as a phylogenetically distinct monophyletic lineage (Figs 7–8), supported as a sister lineage to the clade that includes *Aphonopelma catalina* sp. n., *Aphonopelma chiricahua* sp. n., and *Aphonopelma madera* sp. n. The significant measurements that distinguish male *Aphonopelma peloncillo* from its closely related phylogenetic and syntopic species are Cl, PTl, and the extent of scopulation on metatarsus III. Male *Aphonopelma peloncillo* can be distinguished by possessing a larger Cl/PTw (≥4.67; 4.67–5.59) than *Aphonopelma catalina* (≤4.26; 3.96–4.26), *Aphonopelma chiricahua* (≤4.05; 3.32–4.05), and *Aphonopelma parvum* sp. n. (≤4.22; 3.79–4.22), but smaller than *Aphonopelma gabeli* (≥5.93; 5.93–6.56); by possessing a smaller PTl/M3 (≤0.82; 0.71–0.82) than *Aphonopelma madera* (≥0.94; 0.94–1.05); and a smaller L3 scopulation extent (52%–68%) than *Aphonopelma chalcodes* (75%–86%) and *Aphonopelma hentzi* (69%–86%). There are no significant measurements that separate male *Aphonopelma peloncillo* from *Aphonopelma vorhiesi*. Significant measurements that distinguish female *Aphonopelma peloncillo* from its closely related phylogenetic and syntopic species are Cl, A4, and the extent of scopulation on metatarsus IV. Female *Aphonopelma peloncillo* can be distinguished by possessing a larger Cl/T1 (≥1.56; 1.56–1.77) than *Aphonopelma catalina* (≤1.51; 1.44–1.51) and *Aphonopelma parvum* (≤1.55; 1.45–1.55); a larger M3/A4 (≥1.11; 1.11–1.23) than *Aphonopelma chiricahua* (0.80 ± (only 1 specimen)) and *Aphonopelma madera* (≤1.07; 0.96–1.07); and by possessing a smaller L4 scopulation extent (32%-39%) than *Aphonopelma chalcodes* (63%–81%), *Aphonopelma hentzi* (42%–72%), and *Aphonopelma gabeli* (39%–53%). There are no significant measurements that separate female *Aphonopelma peloncillo* from *Aphonopelma vorhiesi*.

#### Description of male holotype

(APH_0672; Fig. 118). *Specimen preparation and condition*: Specimen collected live crossing road, preserved in 80% ethanol; original coloration faded due to preservation. Left legs I, III, IV, and left pedipalp removed for measurements and photographs; stored in vial with specimen. Right leg III removed for DNA and stored at -80°C in the AUMNH (Auburn, AL). *General coloration*: Generally black/faded black or brown. *Cephalothorax*: Carapace 12.27 mm long, 11.42 mm wide; densely clothed with black, faded black or brown pubescence, appressed to surface; fringe covered in long setae not closely appressed to surface; foveal groove medium deep and straight; pars cephalica region rises gradually from foveal groove, gently arching anteriorly toward ocular area; ocular quadrangle distinct and round; AER procurved, PER strongly recurved; normal sized chelicerae; clypeus slightly extends forward on a curve; LBl 1.51, LBw 1.84; sternum hirsute, clothed with short black, densely packed setae. *Abdomen*: Densely clothed in short black pubescence with numerous longer, lighter setae interspersed (generally red or orange *in situ*); dense dorsal patch of black Type I urticating bristles (Cooke et al. 1972). *Legs*: Hirsute; densely clothed with short, similar length black setae, and longer setae interspersed. Metatarsus I slightly curved. F1 13.45; F1w 3.02; P1 4.88; T1 11.17; M1 8.31; A1 5.87; F3 10.01; F3w 3.16; P3 4.43; T3 7.87; M3 9.06; A3 6.04; F4 12.19; F4w 3.04; P4 4.52; T4 10.45; M4 12.53; A4 7.11; femur III is normal - not noticeably swollen or wider than other legs. All tarsi fully scopulate. Extent of metatarsal scopulation: leg III (SC3) = 58.4%; leg IV (SC4) = 34.4%. Two ventral and two prolateral spinose setae on metatarsus III; eleven ventral spinose setae, and one prolateral spinose seta on metatarsus IV. *Coxa I*: Prolateral surface a mix of fine, hair-like and thin tapered setae. *Pedipalps*: Hirsute; densely clothed in the same setal color as the other legs, with numerous longer ventral setae; one spinose seta at the apical, prolateral femur and three spinose setae on the prolateral tibia; PTl 7.36, PTw 2.47. When extended, embolus tapers and gently curves to the retrolateral side; embolus slender, no keels.

**Variation (9).** Cl 10.23–14.90 (12.567±0.44), Cw 9.15–14.10 (11.582±0.46), LBl 1.32–1.95 (1.46±0.06), LBw 1.4–2.07 (1.651±0.07), F1 11.148–14.86 (13.352±0.4), F1w 2.423–3.795 (3.059±0.12), P1 4.151–5.594 (4.945±0.18), T1 9.699–12.83 (11.482±0.36), M1 7.331–10.436 (8.77±0.29), A1 5.179–6.889 (6.104±0.18), L1 length 37.508–50.297 (44.654±1.31), F3 9.27–12.369 (10.739±0.32), F3w 2.766–4.012 (3.286±0.12), P3 3.629–4.891 (4.129±0.14), T3 6.674–9.573 (8.172±0.3), M3 7.465–10.86 (9.588±0.34), A3 5.712–7.983 (6.445±0.25), L3 length 33.029–45.454 (39.073±1.25), F4 10.549–14.471 (12.599±0.38), F4w 2.469–3.765 (2.996±0.12), P4 3.44–4.991 (4.353±0.16), T4 9.249–12.388 (10.832±0.35), M4 10.683–14.17 (12.768±0.4), A4 6.65–8.656 (7.43±0.24), L4 length 40.656–54.24 (47.981±1.42), PTl 6.119–8.133 (7.382±0.24), PTw 2.027–2.91 (2.507±0.1), SC3 ratio 0.526–0.685 (0.594±0.02), SC4 ratio 0.274–0.442 (0.338±0.02), Coxa 1 setae = tapered/thin tapered, F3 condition = normal.

#### Description of female paratype

(APH_1296; Figs 119–120). *Specimen preparation and condition*: Specimen collected live from burrow, preserved in 80% ethanol. Left legs I, III, IV, and pedipalp removed for photographs and measurements; stored in vial with specimen. Right leg III removed for DNA and stored at -80°C in the AUMNH (Auburn, AL). Genital plate with spermathecae removed and cleared, stored in vial with specimen. *General coloration*: Black/faded black and brown. *Cephalothorax*: Carapace 19.32 mm long, 16.76 mm wide; Hirsute, densely clothed with black/faded black, pubescence closely appressed to surface; fringe densely covered in longer setae; foveal groove medium deep and straight; pars cephalica region gently rises from thoracic furrow, arching anteriorly toward ocular area; AER very slightly procurved, PER slightly recurved; robust chelicerae, clypeus slightly extends forward on a curve; LBl 1.74, LBw 2.02; sternum very hirsute, clothed with longer black/faded black setae. *Abdomen*: Densely clothed dorsally in short black setae with numerous longer, lighter setae interspersed (generally red or orange *in situ*); dense dorsal patch of black Type I urticating bristles (Cooke et al. 1972). *Spermathecae*: Paired and separate, basic, slightly tapers and curves medially towards capitate bulbs, with wide bases that do not appear fused. *Legs*: Very hirsute; densely clothed with longer setae colored similarly as the long abdominal setae; F1 14.51; F1w 4.60; P1 6.72; T1 10.91; M1 8.45; A1 6.34; F3 11.88; F3w 3.98; P3 5.73; T3 8.62; M3 8.94; A3 6.68; F4 14.42; F4w 4.13; P4 6.40; T4 11.27; M4 11.61; A4 7.56. All tarsi fully scopulate. Extent of metatarsal scopulation: leg III (SC3) = 67.7%; leg IV (SC4) = 38.5%. Two ventral and two prolateral spinose setae on metatarsus III; five ventral spinose setae and one prolateral spinose seta on metatarsus IV. *Coxa I*: Prolateral surface a mix of fine, hair-like and tapered setae. *Pedipalps*: Densely clothed in the same setal color as the other legs; two spinose setae on the apical, prolateral femur, one spinose seta on the prolateral patella, six prolateral spinose setae and one ventral spinose seta on the tibia (two at the apical border with the tarsus).

**Variation (5).** Cl 15.33–19.32 (16.912±0.69), Cw 13.1–16.76 (14.756±0.65), LBl 1.74–2.33 (1.992±0.1), LBw 2.02–2.65 (2.224±0.11), F1 12.25–14.51 (13.252±0.43), F1w 3.81–4.60 (4.086±0.14), P1 5.3–6.72 (5.9±0.28), T1 9.47–10.97 (10.376±0.27), M1 6.68–8.45 (7.686±0.31), A1 5.95–6.34 (6.2±0.07), L1 length 39.65–46.93 (43.414±1.28), F3 9.48–11.88 (10.484±0.43), F3w 3.14–3.98 (3.51±0.14), P3 4.54–5.73 (4.956±0.21), T3 7.01–8.62 (7.748±0.31), M3 7.51–8.94 (8.262±0.3), A3 5.7–6.68 (6.234±0.17), L3 length 34.5–41.85 (37.684±1.26), F4 12.26–14.42 (13.224±0.41), F4w 3.5–4.13 (3.718±0.11), P4 5.09–6.40 (5.444±0.25), T4 9.66–11.27 (10.602±0.26), M4 9.91–12.05 (11.294±0.39), A4 6.63–7.56 (7.118±0.18), L4 length 43.84–51.26 (47.682±1.31), SC3 ratio 0.586–0.676 (0.64±0.02), SC4 ratio 0.324–0.386 (0.357±0.01), Coxa I setae = tapered. Spermathecae variation can be seen in Figure 120.

#### Material examined.

**United States: Arizona: Cochise**: 0.03 miles N Hunt Canyon Rd on Leslie Canyon Rd, 31.64689064 -109.4732291 ^2^, 4927ft., [APH_0718, 17/8/2009, 1♂, Alice Abela, AUMNH]; 0.51 miles N Davis Ranch Rd on Leslie Canyon Rd, 31.52685642 -109.5446671 ^2^, 4389ft., [APH_0719, 17/8/2009, 1♂, Alice Abela, AUMNH]; 0.83 miles SW Price Canyon Rd on Hwy-80, 31.62561536 -109.2064459 ^2^, 4656ft., [APH_0724, 18/8/2009, 1♂, Alice Abela, AUMNH]; 19.8 miles NE of Douglas along Highway 80, 31.53231 -109.294192 ^5^, 4562ft., [AUMS_2246, 23/7/1989, 1♂, F.C. Baptista, AUMNH]; along Hwy-80, 31.46999359 -109.3591602 ^2^, 4346ft., [APH_0723, 18/8/2009, 1♂, Alice Abela, AUMNH]; **New Mexico: Hidalgo**: along CR C001, 31.608773 -108.86636 ^1^, 4966ft., [APH_1227, 7/8/2010, 1♂, Brent E. Hendrixson, Ashley Bailey, Andrea Reed, AUMNH]; along CR C004, 31.527331 -108.905731 ^1^, 5262ft., [APH_1226, 7/8/2010, 1♂, Brent E. Hendrixson, Ashley Bailey, Andrea Reed, AUMNH]; along Geronimo Trail, 31.528707 -108.900331 ^1^, 5032ft., [APH_1516, 8/9/2012, 1♀, Brent E. Hendrixson, AUMNH]; along Hwy-338, 31.607079 -108.867081 ^1^, 4934ft., [APH_0671-0673, 14/7/2009, 2♂, 1 juv, Brent E. Hendrixson, Nate Davis, AUMNH]; [APH_1188-1191, 26/7/2010, 4♂, Brent E. Hendrixson, Brendon Barnes, Nate Davis, AUMNH & AMNH]; Clanton Draw area, 31.522379 -108.98037 ^1^, 5406ft., [APH_0666-0669, 14/7/2009, 1♀, 3 juv, Brent E. Hendrixson, Nate Davis, AUMNH]; [APH_0681-0685, 16/7/2009, 3♀, 1♂, 1 juv, Brent E. Hendrixson, Nate Davis, AUMNH]; Coronado National Forest, Peloncillo Mtns, 31.518665 -108.982597 ^1^, 5435ft., [APH_1181-1182, 26/7/2010, 2♀, Brent E. Hendrixson, Brendon Barnes, Nate Davis, AUMNH & AMNH]; Coronado National Forest, Peloncillo Mtns, Clanton Draw Area, 31.518665 -108.982597 ^1^, 5435ft., [APH_1296-1297, 25/7/2011, 2♀, Brent E. Hendrixson, Brendon Barnes, Nate Davis, Jake Storms, AUMNH]; CR-C004, 0.8 miles SW Hwy-338, 31.544499 -108.882007 ^1^, 5112ft., [APH_0670, 14/7/2009, 1♂, Brent E. Hendrixson, Nate Davis, AUMNH]; NM-9 at Little Hatchet Mtns Rd, W of Hachita, 31.926633 -108.363083 ^5^, 4480ft., [APH_0411, 11/10/2008, 1♂, Kari McWest, Keisha Hendricks, AUMNH].

#### Distribution and natural history.

*Aphonopelma peloncillo* can be found inhabiting the Madrean Archipelago Level III Ecoregion where it resides in lower to mid elevation habitats in and around the Peloncillo Mountains in southeastern Arizona and southwestern New Mexico (Fig. 121). *Aphonopelma peloncillo* can be found in syntopy at the lower elevations of its distribution with *Aphonopelma chalcodes*, *Aphonopelma gabeli*, *Aphonopelma hentzi*, *Aphonopelma parvum*, and *Aphonopelma vorhiesi*. The breeding season, when mature males abandon their burrows in search of females, occurs during the summer (July–August) coinciding with the summer monsoon season.

#### Conservation status.

*Aphonopelma peloncillo* has a somewhat narrow distribution in the United States that is restricted to middle and lower elevations in and around the Peloncillo Mountains of Arizona and New Mexico. These tarantulas are fairly common but this region is subject to grazing, illegal immigration, and other modes of habitat degradation. Future climate change throughout the sky island region is also a concern (Brusca et al. 2013).

#### Remarks.

This species was originally recognized by Hendrixson et al. (2015). *Aphonopelma peloncillo* males and females are similar looking to other species in the sky islands, though generally possessing a more hirsute appearance. *Aphonopelma peloncillo* can be differentiated from *Aphonopelma chalcodes*, *Aphonopelma gabeli*, *Aphonopelma hentzi*, and *Aphonopelma vorhiesi* by phenotypic appearance, and from *Aphonopelma parvum* by appearance and size. An interesting note, in both mtDNA and nuDNA datasets, *Aphonopelma peloncillo* is inferred as sister to a lineage in the Huachuca Mountains (that is not *Aphonopelma madera*), putatively identified as a novel species. Unfortunately, at this time we do not have additional support for this single specimen to be recognized and formally described.

Other important ratios that distinguish males: *Aphonopelma peloncillo* possess a smaller PTl/M3 (≤0.82; 0.71–0.82) than *Aphonopelma catalina* (≥0.86; 0.86–0.98), *Aphonopelma chiricahua* (≥0.94; 0.94–1.10) and *Aphonopelma parvum* (≥0.81; 0.81–0.88, with slight overlap), but larger than *Aphonopelma gabeli* (≤0.63; 0.57–0.63); by possessing a larger L3 scopulation extent (52%-68%) than *Aphonopelma catalina* (48%–53%, with slight overlap); by possessing a smaller F1/M4 (≤1.11; 0.99–1.11) than *Aphonopelma catalina* (≥1.15; 1.15–1.22) and *Aphonopelma madera* (≥1.12; 1.12–1.22), but larger than *Aphonopelma chalcodes* (≤0.99; 0.92–0.99) and *Aphonopelma gabeli* (≤0.97; 0.92–0.97); by possessing a larger T1/P4 (≥2.47; 2.47–2.94) than *Aphonopelma hentzi* (≤2.46; 1.96–2.46). Other important ratios that distinguish females: *Aphonopelma peloncillo* possess a larger M3/A4 (≥1.11; 1.11–1.23) than *Aphonopelma catalina* (≤1.10; 1.07–1.10) and *Aphonopelma parvum* (≤0.92; 0.86–0.92); by possessing a smaller L3 scopulation extent (58%–68%) than *Aphonopelma chalcodes* (78%-93%), *Aphonopelma hentzi* (67%–95%, with slight overlap), and *Aphonopelma gabeli* (72%–80%). For both males and females, certain morphometrics have potential to be useful, though due to the amounts of variation, small number of specimens, and the small differences between species, no other are claimed to be significant at this time (see Suppl. material 2). During evaluation of traditional PCA morphospace, males of *Aphonopelma peloncillo* separate from *Aphonopelma chalcodes*, *Aphonopelma gabeli*, *Aphonopelma madera*, and *Aphonopelma parvum* along PC1~2, but do not separate from *Aphonopelma catalina*, *Aphonopelma chiricahua*, *Aphonopelma hentzi*, and *Aphonopelma vorhiesi*. Females of *Aphonopelma peloncillo* separate from *Aphonopelma chiricahua* and *Aphonopelma parvum* along PC1~2, but do not separate from *Aphonopelma catalina*, *Aphonopelma chalcodes*, *Aphonopelma gabeli*, *Aphonopelma hentzi*, *Aphonopelma madera*, and *Aphonopelma vorhiesi*. Interestingly, *Aphonopelma peloncillo* males separate from *Aphonopelma catalina*, *Aphonopelma chiricahua*, *Aphonopelma madera*, and *Aphonopelma parvum* in three-dimensional PCA morphospace (PC1~PC2~PC3), but do not separate from *Aphonopelma chalcodes*, *Aphonopelma gabeli*, *Aphonopelma hentzi*, and *Aphonopelma vorhiesi*. *Aphonopelma peloncillo* females separate from *Aphonopelma chiricahua*, *Aphonopelma madera*, and *Aphonopelma parvum*, but do not separate from *Aphonopelma catalina*, *Aphonopelma chalcodes*, *Aphonopelma gabeli*, *Aphonopelma hentzi*, and *Aphonopelma vorhiesi*. PC1, PC2, and PC3 explain ≥96% of the variation in all analyses.

### Aphonopelma phasmus

Taxon classificationAnimaliaAraneaeTheraphosidae

Chamberlin, 1940

Figures 122 , 123

Aphonopelma (Delopelma) phasmus Chamberlin, 1940: 28; male holotype from Phantom Ranch, Grand Canyon, Coconino Co., Arizona, 36.105315 -112.095201, elev. 2536ft., 26.vii.1934, coll. Dr. Lutz; deposited in AMNH. [examined]
Rhechostica phasmus Raven, 1985: 149.
Aphonopelma phasmus Smith, 1995: 129.

#### Diagnosis.

**Figure 122.**
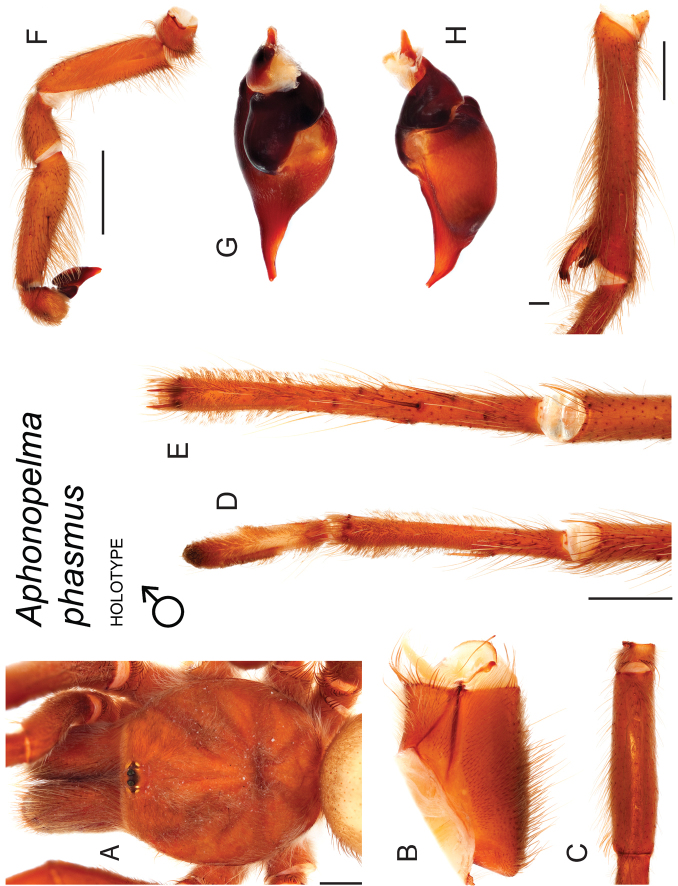
*Aphonopelma phasmus* Chamberlin, 1940. **A–I** male holotype **A** dorsal view of carapace, scale bar = 1mm **B** prolateral view of coxa I **C** dorsal view of femur III **D** ventral view of metatarsus III, scale bar = 2.5mm **E** ventral view of metatarsus IV **F** prolateral view of L pedipalp and palpal tibia, scale bar = 3.5mm **G** dorsal view of palpal bulb (damaged) **H** retrolateral view of palpal bulb (damaged) **I** prolateral view of tibia I (mating clasper), scale bar = 2mm.

Based on carapace length (a proxy for body size) and evaluation of morphospace, *Aphonopelma phasmus* probably belongs to the *paloma* species group but molecular data is needed to confirm its phylogenetic placement. Although the tip of the embolus is broken, the palpal bulb is unique (Fig. 122) compared to other species found in the region (i.e., *Aphonopelma iodius*, *Aphonopelma marxi*, and *Aphonopelma prenticei*). The carapace is distinctly more round than *Aphonopelma prenticei*; *Aphonopelma phasmus* possess a larger F3L/W ratio (3.59 ± (only 1 specimen)) than *Aphonopelma prenticei* (≤3.27; 2.76–3.27), and larger L3 scopulation extent (81.7% ± (only 1 specimen)) than *Aphonopelma prenticei* (60%-73%). *Aphonopelma phasmus* can be differentiated from *Aphonopelma marxi* by comparing the PTl, Cl, and metatarsus measurements; *Aphonopelma phasmus* possess a smaller PTl/M1 (0.79 ± (only 1 specimen)) than *Aphonopelma marxi* (≥0.92: 0.92–1.23) and a smaller Cl/M1 (1.21 ± (only 1 specimen)) than *Aphonopelma marxi* (≥1.43; 1.43–1.89).

#### Description.

Male originally described by Chamberlin (1940).

#### Redescription of male holotype

(Fig. 122). *Specimen preparation and condition*: unknown collecting information; deposited in AMNH; original coloration faded due to preservation. Specimen badly fragmented, with legs stored in vial with specimen; most setae have fallen off over time. Specimen was pieced together for measurements. *General coloration*: Faded brown. *Cephalothorax*: Carapace 7.89 mm long, 6.84 mm wide; foveal groove medium deep and straight; pars cephalica region rises gradually from foveal groove to ocular area; AER very slightly procurved, PER straight; normal sized chelicerae; clypeus extends forward on a curve; LBl 1.072, LBw 1.315. *Abdomen*: Devoid of setae - lost over time. *Legs*: Metatarsus I very slightly curved. F1 8.88; F1w 1.917; P1 3.598; T1 8.124; M1 6.523; A1 4.773; L1 length 31.898; F3 7.338; F3w 2.046; P3 2.635; T3 5.979; M3 6.902; A3 4.369; L3 length 27.223; F4 8.59; F4w 1.79; P4 2.93; T4 *missing/damaged*; M4 *missing/damaged*; A4 *missing/damaged*; L4 *incomplete*; femur III is slightly swollen. All tarsi fully scopulate. Extent of metatarsal scopulation: leg III (SC3) = 81.7%; leg IV (SC4) = *missing/damaged*. Two ventral spinose setae and one prolateral spinose seta on metatarsus III; five ventral spinose setae and one prolateral spinose seta on metatarsus IV; one prolateral spinose seta on tibia I; one large megaspine is present at the apex on the retrolateral tibia of the mating clasper. *Coxa I*: Prolateral surface covered by thin tapered and fine, hair-like setae. *Pedipalps*: One spinose seta near the anterior margin of the prolateral palpal femur; three spinose setae on the prolateral palpal tibia (one near the anterior/ventral margin), one ventral spinose seta towards the anterior margin; PTl 5.188, PTw 1.768. Palpal bulb is unique; embolus is broken; distinct ventral transition from bulb to embolus; unique process extending posteriorly.

#### Distribution and natural history.

**Figure 123.**
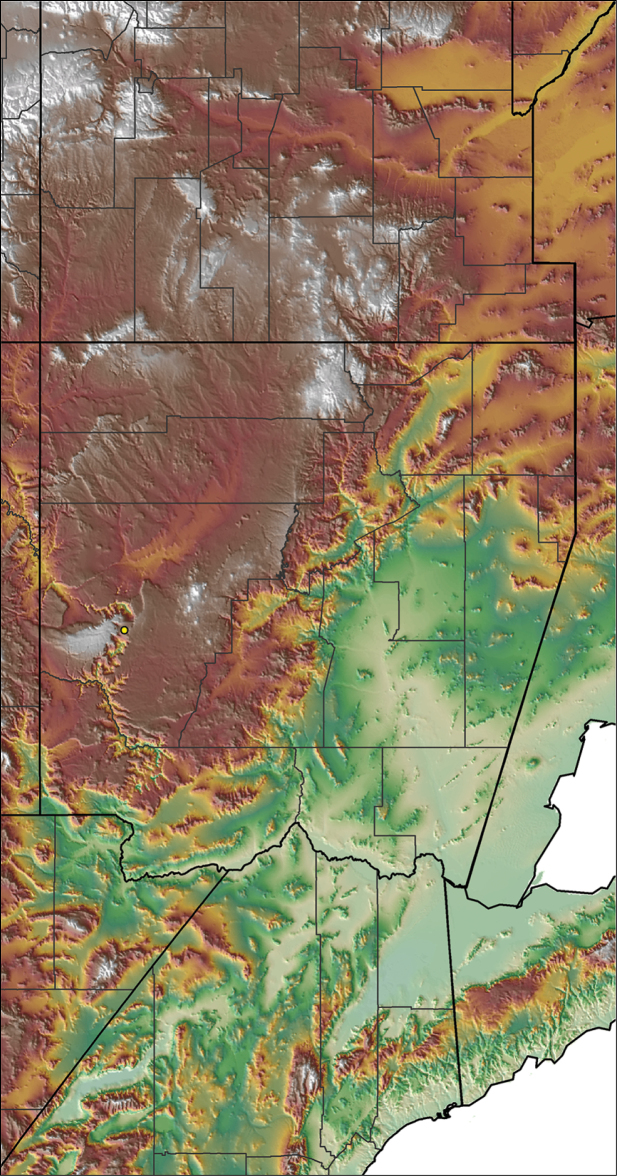
*Aphonopelma phasmus* Chamberlin, 1940 distribution of known specimens. There is no predicted distribution map due to the limited number of sampling localities and restricted distribution this species possesses.

*Aphonopelma phasmus* is known from a single adult male collected at Phantom Ranch (770 meters) near the Colorado River in Grand Canyon National Park in Coconino County, Arizona (Fig. 123); Phantom Ranch is characterized by riparian habitat surrounded by desert. The July collection date for the holotype indicates a summer breeding period for this species. *Aphonopelma phasmus* is the only species known to inhabit the bottom of Grand Canyon; *Aphonopelma marxi* is abundant along the South Rim in ponderosa pine forest, some 1300 meters higher in elevation and *Aphonopelma prenticei* can be found in desert habitats north and west of Grand Canyon.

#### Conservation status.

*Aphonopelma phasmus* probably has a very restricted distribution along the Colorado River in Grand Canyon National Park. Although the species is protected by the boundaries of the park, the Phantom Ranch area is prone to habitat degradation due to foot traffic and other recreational activities. New collecting efforts to find this species need to be taken before its conservation status can be assessed.

#### Species concept applied.

Morphological Species Concept.

#### Remarks.

Very little is known about this enigmatic species. Despite our extensive fieldwork throughout Arizona and surrounding areas, we have not encountered any other specimens that appear to be conspecific with *Aphonopelma phasmus*. Focused collection efforts at Phantom Ranch and other locations along the Colorado River are necessary to fully grasp the validity of this species and to determine its phylogenetic placement. A handful of measurements demonstrate that *Aphonopelma phasmus* likely is not conspecific with other species in the surrounding area (i.e., *Aphonopelma iodius*, *Aphonopelma marxi*, and *Aphonopelma prenticei*) but new material is needed to confirm this hypothesis. Unfortunately, the holotype is badly damaged, fragmented, and missing multiple pieces, and because we lack more specimens, we are not able to understand the possible morphological variation that exists within this species. During evaluation of traditional PCA morphospace and three-dimensional PCA morphospace (PC1~PC2~PC3), *Aphonopelma phasmus* groups with the other species in the *paloma* species group (see Suppl. material 2).

### Aphonopelma prenticei

Taxon classificationAnimaliaAraneaeTheraphosidae

Hamilton, Hendrixson & Bond sp. n.

http://zoobank.org/320D8826-1CCD-46EE-9248-34ABDB9C9B26

**Figure 124.**
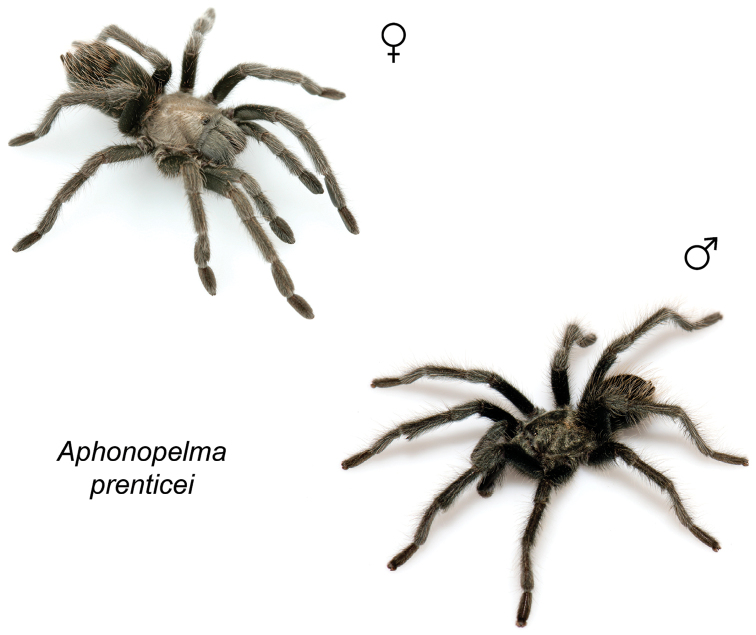
*Aphonopelma prenticei* sp. n. live photographs. Female (L) - APH_3202; Male (R) - APH_1569.

**Figure 125.**
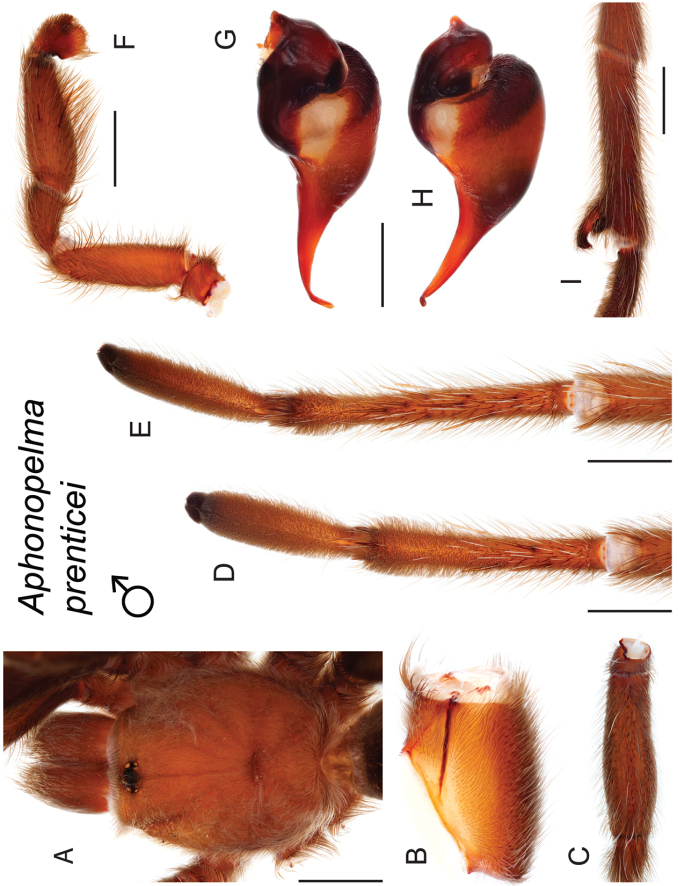
*Aphonopelma prenticei* sp. n. **A–I** male holotype, AUMS_2557 **A** dorsal view of carapace, scale bar = 3mm **B** prolateral view of coxa I **C** dorsal view of femur III **D** ventral view of metatarsus III, scale bar = 2mm **E** ventral view of metatarsus IV, scale bar = 2mm **F** prolateral view of L pedipalp and palpal tibia, scale bar = 2.5mm **G** dorsal view of palpal bulb **H** retrolateral view of palpal bulb, scale bar = 0.5mm **I** prolateral view of tibia I (mating clasper), scale bar = 2.5mm.

**Figure 126.**
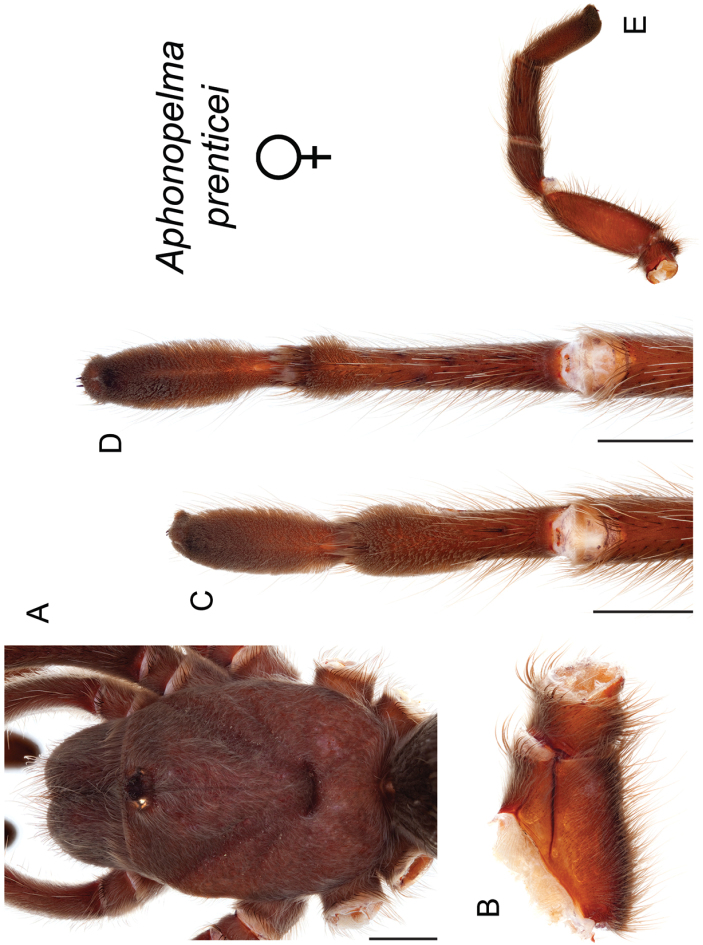
*Aphonopelma prenticei* sp. n. **A–E** female paratype, APH_0319 **A** dorsal view of carapace, scale bar = 2mm **B** prolateral view of coxa I **C** ventral view of metatarsus III, scale bar = 2mm **D** ventral view of metatarsus IV, scale bar = 2mm **E** prolateral view of L pedipalp and palpal tibia.

**Figure 127.**
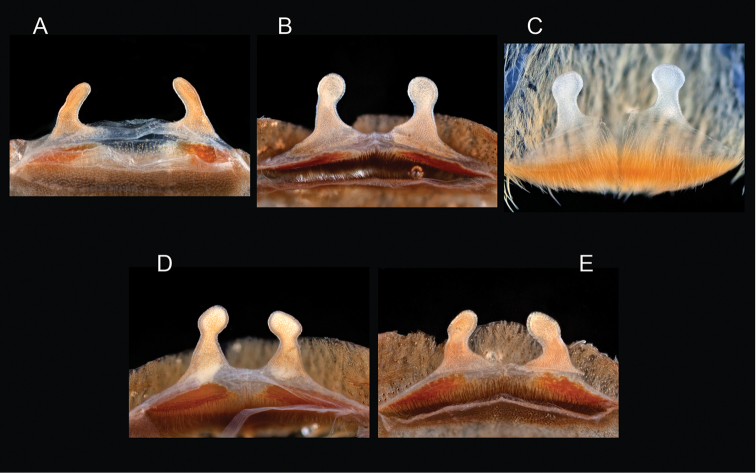
*Aphonopelma prenticei* sp. n. **A–E** cleared spermathecae **A** APH_0319 **B** APH_0786 **B** AUMS_2554 **D** AUMS_3270 **E** AUMS_3279.

**Figure 128.**
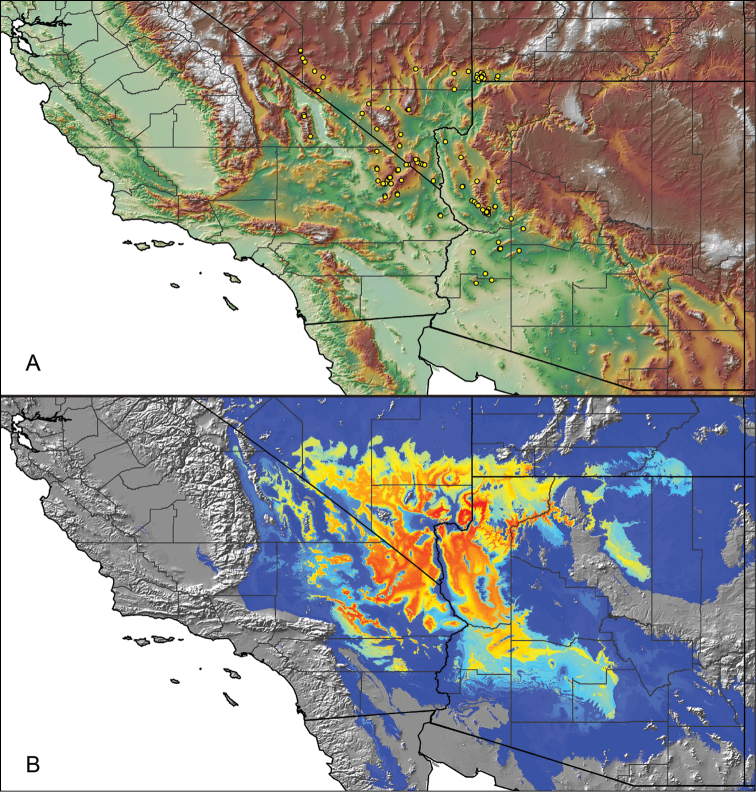
*Aphonopelma prenticei* sp. n. **A** distribution of known specimens **B** predicted distribution; warmer colors (red, orange, yellow) represent areas of high probability of occurrence, cooler colors (blue shades) represent areas of low probability of occurrence.

Figures 124 , 125 , 126 , 127 , 128

Aphonopelma mojave Prentice, 1997 (in part): 161.

#### Types.

Male holotype (AUMS_2557) collected Alamo Rd., off I-40 and Yucca, 0.4 miles SE of campsite, Mohave Co., Arizona, 34.604435 -113.751233 ^4^, elev. 3300ft., 13.x.1993, coll. T.R. Prentice; deposited in AUMNH. Paratype female (APH_0319) from 0.4 miles S of Kelso Dunes Rd on Kelbaker Rd, San Bernardino Co., California, 34.89574 -115.65016 ^1^, elev. 2825ft., 6.v.2008, coll. Brent E. Hendrixson and Zach Valois; deposited in AUMNH. Paratype male (APH_0415) from Mojave National Preserve, San Bernardino Co., California, 35.173875 -115.609553 ^1^, elev. 4130ft., 25.x.2008, coll. Anette Pillau; deposited in AMNH. Paratype female (AUMS_2554) from Chicken Springs Rd., 2 miles N of Alamo rd. crossing, Mohave Co., Arizona, 34.609856 -113.77401 ^5^, elev. 3250ft., 20.ix.1992, coll. T.R. Prentice; deposited in AMNH.

#### Etymology.

The specific epithet is a patronym in recognition of arachnologist Thomas R. Prentice for his work on the genus *Aphonopelma*. Prentice’s (1993, 1997) studies inspired our interest in the genus and greatly influenced the way we approached this revision. This project benefited tremendously from his help collecting specimens, donating his personal collection to the AUMNH, and sharing his encyclopedic knowledge of North American *Aphonopelma*.

#### Diagnosis.

*Aphonopelma prenticei* (Fig. 124) is a member of the *paloma* species group and can be distinguished by a combination of morphological, molecular, and geographic characteristics. Nuclear and mitochondrial DNA identifies *Aphonopelma prenticei* as a strongly supported monophyletic lineage (Figs 7–8) that is the sister lineage to *Aphonopelma atomicum* sp. n., *Aphonopelma icenoglei* sp. n., *Aphonopelma mojave*, and *Aphonopelma joshua*. *Aphonopelma prenticei* can easily be differentiated from syntopic populations of *Aphonopelma iodius* and *Aphonopelma chalcodes* due to their smaller size and limited extent of scopulation on metatarsus IV. *Aphonopelma prenticei* can also be differentiated from other turret-building members of the *paloma* species group by locality. The most significant measurements that distinguish male *Aphonopelma prenticei* from its closely related phylogenetic and syntopic species are F3 and the extent of scopulation on metatarsus IV. Male *Aphonopelma prenticei* can be distinguished by possessing a larger PTl/F3 (≥0.63; 0.63–0.73) than *Aphonopelma joshua* (≤0.61; 0.55–0.61) and *Aphonopelma xwalxwal* sp. n. (≤0.63; 0.59–0.63); a larger A1/F3 (≥0.58; 0.58–0.65) than *Aphonopelma atomicum* (≤0.56; 0.53–0.56); a smaller F3L/W (≤3.27; 2.76–3.27) than *Aphonopelma icenoglei* (≥3.51; 3.51–3.90); and a smaller L4 scopulation extent (30%-42%) than *Aphonopelma iodius* (62%–88%) and *Aphonopelma chalcodes* (42%–76%). There are no significant measurements that separate male *Aphonopelma prenticei* from *Aphonopelma mojave*, although *Aphonopelma mojave* males do not possess a swollen femur III (as seen in *Aphonopelma atomicum* and *Aphonopelma prenticei*). The most significant measurements that distinguish female *Aphonopelma prenticei* from its closely related phylogenetic and syntopic species are Cl, M4, and the extent of scopulation on metatarsus IV. Female *Aphonopelma prenticei* can be distinguished by possessing a larger Cl/M4 (≥1.31; 1.31–1.52) than *Aphonopelma atomicum* (≤1.28; 1.21–1.28); a larger P4/M4 (≥0.46; 0.46–0.58) than *Aphonopelma mojave* (≤0.45; 0.39–0.45) and *Aphonopelma joshua* (≤0.43; 0.37–0.43); a larger Cl/F1 (≥1.17; 1.17–1.33) than *Aphonopelma icenoglei* (≤1.16; 1.07–1.16); and a smaller L4 scopulation extent (27%–38%) than *Aphonopelma chalcodes* (56%–81%) and *Aphonopelma iodius* (59%–83%).

#### Description of male holotype

(AUMS_2557; Fig. 125). *Specimen preparation and condition*: Specimen originally collected wandering and preserved in unknown percentage of ethanol; original coloration faded due to preservation. Left legs I, III, IV, and left pedipalp removed for measurements and photographs; stored in vial with specimen. No tissue for DNA. *General coloration*: Faded black/brown. *Cephalothorax*: Carapace 7.177 mm long, 6.592 mm wide; densely clothed with faded pubescence, appressed to surface; fringe covered in long setae not closely appressed to surface, hirsute appearance; foveal groove medium deep and very slightly recurved; pars cephalica region rises very gradually from foveal groove on a straight plane towards the ocular area; AER very slightly procurved, PER mostly straight; normal sized chelicerae; clypeus mostly straight; LBl 0.948, LBw 0.933; sternum hirsute, clothed with faded, densely packed, short setae. *Abdomen*: Densely clothed in short black/brown pubescence with numerous longer, lighter setae interspersed (generally red or orange *in situ*); dense dorsal patch of black Type I urticating bristles (Cooke et al. 1972) - smaller and distinct from large species. *Legs*: Hirsute; densely clothed in faded pubescence. Metatarsus I straight. F1 7.655; F1w 1.721; P1 3.184; T1 6.597; M1 5.7; A1 4.08; F3 6.623; F3w 2.298; P3 3.007; T3 5.36; M3 6.482; A3 4.205; F4 7.724; F4w 1.884; P4 2.909; T4 6.59; M4 8.022; A4 4.271; femur III is swollen. All tarsi fully scopulate. Extent of metatarsal scopulation: leg III (SC3) = 60.4%; leg IV (SC4) = 35.4%. Three ventral spinose setae and one retrolateral spinose seta on metatarsus III; eight ventral spinose setae and one retrolateral spinose seta on metatarsus IV; two prolateral spinose setae on tibia I; one megaspine present on the retrolateral tibia, at the apex of the mating clasper; one megaspine on the apex on the retrolateral branch of the tibial apophyses. *Coxa I*: Prolateral surface covered by fine, hair-like setae. *Pedipalps*: Hirsute; densely clothed in the same setal color as the other legs, with numerous longer ventral setae; one spinose seta at the apical, prolateral femur and three prolateral spinose setae on the palpal tibia; PTl 4.426, PTw 1.41. When extended, embolus tapers with a curve to the retrolateral side; embolus slender, no keels; distinct dorsal and ventral transition from bulb to embolus.

**Variation (5).** Cl 6.478–7.787 (7.244±0.24), Cw 5.42–6.948 (6.448±0.27), LBl 0.848–1.106 (0.992±0.05), LBw 0.933–1.204 (1.058±0.05), F1 6.693–8.289 (7.534±0.27), F1w 1.547–1.883 (1.744±0.06), P1 2.612–3.597 (3.022±0.17), T1 6.131–6.931 (6.536±0.17), M1 5.269–6.296 (5.817±0.18), A1 3.654–4.532 (4.094±0.14), L1 length 24.359–29.645 (27.003±0.87), F3 5.915–7.23 (6.599±0.22), F3w 1.806–2.612 (2.24±0.13), P3 2.104–3.078 (2.612±0.19), T3 4.631–5.693 (5.237±0.17), M3 5.727–6.952 (6.322±0.2), A3 3.64–4.396 (4.125±0.13), L3 length 22.017–27.275 (24.896±0.87), F4 6.814–8.616 (7.66±0.31), F4w 1.367–2.119 (1.778±0.12), P4 2.314–3.262 (2.688±0.17), T4 6.244–7.306 (6.722±0.2), M4 7.223–8.964 (8.075±0.28), A4 4.037–4.64 (4.326±0.11), L4 length 26.632–32.291 (29.471±0.93), PTl 4.305–4.746 (4.485±0.08), PTw 1.367–1.754 (1.53±0.07), SC3 ratio 0.604–0.728 (0.647±0.02), SC4 ratio 0.308–0.416 (0.355±0.02), Coxa I setae = thin/very thin tapered, F3 condition = swollen.

#### Description of female paratype

(APH_0319; Figs 126–127). *Specimen preparation and condition*: Specimen collected live from burrow, preserved in 80% ethanol; original coloration faded due to preservation. Left legs I, III, IV, and pedipalp removed for photographs and measurements; stored in vial with specimen. Right legs III & IV removed for DNA and stored at AUMNH (Auburn, AL). Genital plate with spermathecae removed and cleared, stored in vial with specimen. *General coloration*: Faded black/brown. *Cephalothorax*: Carapace 7.85 mm long, 6.56 mm wide; Hirsute, densely clothed with short faded black/brown pubescence closely appressed to surface; fringe densely covered in slightly longer setae; foveal groove medium deep and procurved; pars cephalica region gently rises from thoracic furrow, arching anteriorly toward ocular area; AER very slightly procurved, PER slightly recurved; chelicerae robust, clypeus mostly straight; LBl 1.256, LBw 1.224; sternum hirsute, clothed with short faded setae. *Abdomen*: Densely clothed dorsally in short faded black setae with longer, lighter setae (generally red or orange *in situ*) focused near the urticating patch; dense dorsal patch of black Type I urticating bristles (Cooke et al. 1972) - smaller and distinct from large species. *Spermathecae*: Paired and separate, with bulbs widening towards the bases; not fused. *Legs*: Hirsute; densely clothed in short faded black/brown pubescence; F1 6.135; F1w 1.931; P1 2.555; T1 5.051; M1 3.732; A1 3.092; F3 5.587; F3w 1.843; P3 2.489; T3 3.996; M3 4.414; A3 3.447; F4 6.967; F4w 1.937; P4 2.702; T4 5.796; M4 5.845; A4 4.04. All tarsi fully scopulate. Extent of metatarsal scopulation: leg III (SC3) = 64.1%; leg IV (SC4) = 27.3%. Two ventral spinose setae and one retrolateral spinose seta on metatarsus III; five ventral spinose setae on metatarsus IV. *Coxa I*: Prolateral surface covered by very thin tapered and fine, hair-like setae. *Pedipalps*: Densely clothed in the same setal color as the other legs; one spinose seta on the apical, prolateral femur, five prolateral (three at the apical, prolateral border with the tarsus) spinose setae on the tibia.

**Variation (6).** Cl 7.186–8.426 (7.795±0.18), Cw 6.143–6.991 (6.582±0.14), LBl 1.115–1.415 (1.225±0.04), LBw 1.216–1.564 (1.308±0.05), F1 5.971–6.94 (6.296±0.15), F1w 1.803–2.093 (1.955±0.04), P1 2.51–3.35 (2.884±0.13), T1 4.764–5.732 (5.26±0.16), M1 3.692–4.337 (3.936±0.11), A1 3.018–3.856 (3.353±0.12), L1 length 20.434–23.665 (21.728±0.57), F3 4.845–5.662 (5.262±0.16), F3w 1.706–2.31 (1.868±0.09), P3 2.211–2.595 (2.458±0.05), T3 3.47–4.381 (3.878±0.13), M3 3.385–4.414 (4.008±0.17), A3 3.246–3.751 (3.44±0.07), L3 length 17.427–20.562 (19.047±0.52), F4 6.25–7.168 (6.744±0.17), F4w 1.736–1.993 (1.878±0.04), P4 2.474–3.124 (2.852±0.1), T4 5.215–6.022 (5.586±0.15), M4 4.96–5.997 (5.531±0.16), A4 3.519–4.473 (3.874±0.14), L4 length 22.644–26.604 (24.587±0.58), SC3 ratio 0.641–0.711 (0.671±0.01), SC4 ratio 0.273–0.384 (0.332±0.02), Coxa I setae = thin tapered. Spermathecae variation can be seen in Figure 127.

#### Material examined.

**United States: Arizona: La Paz**: 0.4 miles NNW Alamo Rd on Low Mtn Rd, 33.92798 -113.54573 ^2^, 2321ft., [APH_1113, 2/11/2009, 1♂, June Olberding, AUMNH]; 0.6 miles NE Alamo Rd on Low Mountain Rd, 33.91962 -113.53957 ^2^, 2262ft., [APH_1112, 3/11/2009, 1♂, June Olberding, AUMNH]; 0.7 miles E Alamo Rd on 77th St, 7 miles N Wenden, 33.928133 -113.530933 ^2^, 2319ft., [APH_1586, 10/4/2011, 1♂, June Olberding, AUMNH]; 16 miles N US-60 on Alamo Rd (Mile Marker #16), 34.04226 -113.566527 ^1^, 1948ft., [APH_0495, 17/5/2009, 1 juv, Brent E. Hendrixson, Bernadette DeRussy, Sloan Click, AUMNH]; 5.5 miles S La Posa Rd on Plomosa Rd (S of Bouse), 33.85678 -114.02833 ^2^, 1160ft., [APH_0798-0799, 2/11/2009, 2♀, June Olberding, AUMNH]; [APH_1100, 17/10/2009, 1♀, June Olberding, AUMNH]; 6.9 miles N US-60 on Alamo Rd, 33.9214 -113.5416 ^2^, 2280ft., [APH_0797, 1/11/2009, 1♂, June Olberding, AUMNH]; 6.9 miles N US-60 on Alamo Rd (near Base of Harcuvar Mtns N of Wenden), 33.92479 -113.54419 ^1^, 2285ft., [APH_0338-0339, 9/5/2008, 2 juv, Brent E. Hendrixson, Zach Valois, AUMNH]; Harcuvar Mtns, 1.6 miles W Low Mtn Rd along powerline road, 33.938266 -113.530166 ^1^, 2462ft., [APH_1563, 26/10/2012, 1♀, Brent E. Hendrixson, June Olberding, Doug Olberding, AUMNH]; [APH_1565-1567, 23/10/2012, 3♀, June Olberding, AUMNH]; Harcuvar Mtns, Low Mountain Rd, 33.91962 -113.53957 ^2^, 2261ft., [APH_1252, 18/11/2010, 1♂, June Olberding, AUMNH]; Kofa National Wildlife Refuge, 33.4672 -113.80513 ^2^, 1686ft., [APH_1659-1661, 23/11/2012, 1♀, 2 juv, June Olberding, AUMNH]; **Maricopa**: 3.7 miles S US-60 (Aguila) on Eagle Eye Rd, 33.891289 -113.184713 ^1^, 2323ft., [APH_0496-0497, 17/5/2009, 2 juv, Brent E. Hendrixson, Bernadette DeRussy, Sloan Click, AUMNH]; **Mohave**: 12 miles NW Chicken Springs Rd, 34.593079 -113.813486 ^5^, 3020ft., [AUMS_2552, 18/10/1992, 1♀, T.R. Prentice, AUMNH]; 21 miles NW Chicken Springs Rd on Alamo Rd, 34.80901 -114.04903 ^1^, 2350ft., [APH_0350, 11/5/2008, 1 juv, Brent E. Hendrixson, Zach Valois, AUMNH]; 4.7 miles SE US-93/AZ-66 on Hualapai Mountain Rd, 35.162771 -113.961704 ^1^, 4285ft., [APH_0521, 24/5/2009, 1 juv, Brent E. Hendrixson, Bernadette DeRussy, AUMNH]; 5.6 miles NE Alamo Rd on Chicken Springs Rd, 34.66005 -113.74667 ^1^, 3860ft., [APH_0348-0349, 11/5/2008, 2 juv, Brent E. Hendrixson, Zach Valois, AUMNH]; 7.4 miles NE Hwy-93 on CR-25 in Dolan Springs, 35.60418 -114.253563 ^1^, 3638ft., [APH_1310-1312, 28/7/2011, 3♀, Brent E. Hendrixson, Brendon Barnes, Nate Davis, Jake Storms, AUMNH]; Alamo Crossing Rd. 12 miles north of Chicken Springs Rd. jct, 34.716434 -113.941424 ^5^, 3001ft., [AUMS_2549, 24/10/1992, 1♀, T.R. Prentice, AUMNH]; Alamo Rd., 34.547893 -113.754441 ^6^, 2852ft., [APH_3115, unknown, 1♀, T.R. Prentice, AUMNH]; [AUMS_2544, 25/10/1994, 1♀, T.R. Prentice, AUMNH]; [AUMS_2545, 27/10/1994, 1♀, T.R. Prentice, AUMNH]; [AUMS_2548, 29/9/1994, 1♀, T.R. Prentice, AUMNH]; [AUMS_2561, 25/10/1994, 1♂, T.R. Prentice, AUMNH]; [AUMS_2562, 29/9/1994, 1♂, T.R. Prentice, AUMNH]; [AUMS_2568, unknown, 1♀, T.R. Prentice, AUMNH]; [AUMS_2569, 25/10/1994, 1♂, T.R. Prentice, AUMNH]; Alamo Rd., 0.5 miles NW of Alamo Rd., 34.614423 -113.794758 ^4^, 3220ft., [AUMS_2555, 13/10/1993, 1♂, T.R. Prentice, AUMNH]; Alamo Rd., 0.5 miles S of campsite, 34.580474 -113.785733 ^6^, 3083ft., [AUMS_2559, 29/9/1994, 1♂, T.R. Prentice, AUMNH]; Alamo Rd., off I-40 and Yucca, 0.4 miles SE of campsite, 34.604435 -113.751233 ^4^, 3300ft., [AUMS_2557, 13/10/1993, 1♂, T.R. Prentice, AUMNH]; Alamo Road, 12 miles W of Chicken Springs jct, 34.593874 -113.787242 ^5^, 3120ft., [AUMS_2563, 30/3/1993, 1♂, T.R. Prentice, AUMNH]; along Hualapai Mountain Rd, 35.162658 -113.961419 ^1^, 4315ft., [APH_0775-0778, 7/10/2009, 4 juv, Brent E. Hendrixson, Thomas Martin, AUMNH]; campsite, Alamo rd. crossing, 34.597711 -113.796962 ^5^, 3100ft., [AUMS_2564, 29/3/1993, 1♂, T.R. Prentice, AUMNH]; Chicken Springs Rd., 34.607061 -113.767613 ^6^, 3250ft., [AUMS_2566, 23/6/1992, 1♂, T.R. Prentice, AUMNH]; Chicken Springs Rd., 1.4 miles W of Alamo rd. crossing, 34.582903 -113.813953 ^4^, 3060ft., [AUMS_2558, 20/9/1992, 1♂, T.R. Prentice, AUMNH]; Chicken Springs Rd., 1.5 miles E of jct, 34.6148 -113.769604 ^5^, 3300ft., [AUMS_2560, 23/6/1992, 1♂, T.R. Prentice, AUMNH]; Chicken Springs Rd., 1.7 miles N of Alamo Rd. jct., 34.607695 -113.771849 ^4^, 3250ft., [AUMS_2556, 23/6/1992, 1♂, T.R. Prentice, AUMNH]; Chicken Springs Rd., 2 miles N of Alamo rd. crossing, 34.609856 -113.77401 ^5^, 3250ft., [AUMS_2553-2554, 20/9/1992, 2♀, T.R. Prentice, AUMNH & AMNH]; East Mojave, Alamo Rd, 34.605396 -113.811652 ^6^, 3028ft., [AUMS_2550, unknown, 1♀, unknown, AUMNH]; just N I-40 on Silver Springs Rd, 35.161716 -113.564676 ^1^, 3927ft., [APH_1306-1308, 28/7/2011, 1♀, 2 juv, Brent E. Hendrixson, Brendon Barnes, Nate Davis, Jake Storms, AUMNH]; Mt Wilson Wilderness, W of Hwy-143, 35.8885 -114.53848 ^2^, 2074ft., [APH_1635-1638, 24/11/2012, 4 juv, Matt Graham, AUMNH]; Secret Pass Wash, along CR-10, 35.060369 -114.231182 ^1^, 2238ft., [APH_0518-0520, 24/5/2009, 1♀, 2 juv, Brent E. Hendrixson, Rick C. West, Bernadette DeRussy, AUMNH]; Summit Springs, 0.9 miles S. of UT/AZ line, 36.99089 -113.940466 ^4^, 2433ft., [AUMS_2350, unknown, 1♀, unknown, AUMNH]; SW of Kingman in Black Mtns along Rt. 66, 35.06994 -114.21444 ^1^, 2287ft., [APH_0314, 11/10/2007, 1♀, Rick C. West, AUMNH]; Wikieup, S of Kingman on Chicken Springs Rd, 34.70215 -113.622317 ^5^, 2123ft., [APH_0942, 2007, 1♀, Josh Richards, AUMNH]; [APH_0978-0979, 2007, 2 juv, Josh Richards, AUMNH]; **Yavapai**: 12.1 miles SE AZ-97 on US-93, 34.29077 -113.11067 ^1^, 2625ft., [APH_0139-0140, 11/6/2007, 2 juv, Brent E. Hendrixson, AUMNH]; 6.4 miles NW AZ-97 on US-93, 34.47677 -113.32877 ^1^, 3348ft., [APH_0141, 11/6/2007, 1 juv, Brent E. Hendrixson, AUMNH]; **Yuma**: Kofa National Wildlife Refuge, 33.2939 -113.9764 ^2^, 1858ft., [APH_1656-1658, 27/11/2012, 3 juv, June Olberding, AUMNH]; Little Horn Mtns near Hovatter Ranch, 33.352408 -113.691248 ^6^, 1624ft., [APH_2500, 8/5/1980, 1♀, V. Roth, AMNH]; **California: Inyo**: Death Valley National Park, 0.45 miles NE Hwy-178 on Skidoo Rd, 36.387424 -117.144511 ^1^, 4924ft., [APH_0487-0490, 14/5/2009, 4 juv, Brent E. Hendrixson, Bernadette DeRussy, Sloan Click, Jason Bond, AUMNH]; Death Valley National Park, Harrisburg Flats jct with Skidoo Rd, Panamint Mtns, 36.354921 -117.130963 ^5^, 5000ft., [AUMS_2678, 18/10/1963, 1♂, R. Hardy, AUMNH]; Panamint Mtns, Butte Valley Rd, 35.98438 -117.01727 ^2^, 3959ft., [APH_1640, 18/11/2012, 1 juv, Matt Graham, AUMNH]; **San Bernardino**: 0.25 miles W NV State Line on Nipton Rd, 35.475186 -115.229851 ^1^, 3557ft., [APH_0752, 3/10/2009, 1 juv, Brent E. Hendrixson, Thomas Martin, AUMNH]; 0.35 miles W Morning Star Mine Rd on Morning Star Mine Cutoff (dirt road), 35.36247 -115.42249 ^1^, 3200ft., [APH_0356, 11/5/2008, 1 juv, Brent E. Hendrixson, Zach Valois, AUMNH]; 0.4 miles S of Kelso Dunes Rd on Kelbaker Rd, 34.89574 -115.65016 ^1^, 2825ft., [APH_0319-0321, 6/5/2008, 3 juv, Brent E. Hendrixson, Zach Valois, AUMNH]; 100 yards S Havasu Lake Road, 2 miles SE of jct with Hwy 95, 34.529018 -114.627926 ^1^, 1640ft., [APH_3204, 16/11/2013, 1♀, W. Icenogle, AUMNH]; 2.5 miles north of Essex Rd. junction on Black Canyon Rd., 34.937357 -115.424716 ^4^, 3202ft., [APH_2438, 11/10/1992, 1♀, T.R. Prentice, AMNH]; 2.9 miles west on Powerline Rd. from Cima Rd. junction, 35.233597 -115.558759 ^4^, 4636ft., [APH_2452, 11/10/1992, 1♀, T.R. Prentice, AMNH]; 7.6-7.9 miles N of Kelso, 35.116271 -115.652408 ^4^, 2984ft., [AUMS_2528, 22/10/1994, 1♂, T.R. Prentice, AUMNH]; [AUMS_2538, 22/10/1994, 1♂, T.R. Prentice, AUMNH]; [AUMS_2542-2543, 22/10/1994, 2♀, T.R. Prentice, AUMNH]; [AUMS_2546-2547, 22/10/1994, 2♀, T.R. Prentice, AUMNH]; Black Canyon Road- 2.8 miles north of Essex Road Junction, 34.917059 -115.42027 ^4^, 3005ft., [APH_2442, 25/10/1992, 1♀, T.R. Prentice, AMNH]; Cima - 2 miles north of Cedar Canyon Rd., 35.18127 -115.348115 ^5^, 5125ft., [APH_2441, 31/10/1992, 1♂, T.R. Prentice, AMNH]; Cima-Kelso Rd., 10 miles north of Kelso, 35.121413 -115.540532 ^5^, 3232ft., [APH_2447, 1/11/1992, 1♂, T.R. Prentice, AMNH]; East of Nipton - Nevada border, 35.468365 -115.219492 ^5^, 3665ft., [APH_2426, 1/11/1992, 1♂, T.R. Prentice, AMNH]; Halloran Summit Road, 0.3 miles N I-15, 35.407038 -115.795374 ^1^, 4079ft., [APH_0516, 21/5/2009, 1♀, Brent E. Hendrixson, Bernadette DeRussy, Sloan Click, AUMNH]; Halloran Summit, I-15, 35.387482 -115.792505 ^5^, 4170ft., [APH_2430, 24/9/1989, 1♀, T.R. Prentice, AMNH]; Highway 164-1.2 miles north of Nipton, 35.46981 -115.260352 ^4^, 2999ft., [APH_2444, 1/11/1992, 1♂, T.R. Prentice, AMNH]; just S of Havasu Lake Rd, 1 mile SE of jct with Hwy 95, 34.543104 -114.638538 ^1^, 1680ft., [APH_3203, 16/11/2013, 1♀, W. Icenogle, AUMNH]; Kelbaker Rd. 9.4 miles south of Kelso, 34.874762 -115.64445 ^4^, 3123ft., [APH_2449, 31/10/1992, 1♂, T.R. Prentice, AMNH]; Kingston Range, Excelsior Mine Rd, 35.71268 -115.79702 ^2^, 3601ft., [APH_1641, 26/11/2012, 1 juv, Matt Graham, AUMNH]; Mojave National Preserve, 35.173875 -115.609553 ^1^, 4130ft., [APH_0415, 25/10/2008, 1♂, Anette Pillau, AMNH]; Mojave National Preserve, Kelbaker Rd, 35.1728 -115.7756 ^2^, 3231ft., [APH_1564, 21/10/2012, 1♂, June Olberding, Anette Pillau, AUMNH]; Morning Star Mine Rd., 3.1 miles south of Ivanpah Rd., 35.375084 -115.405867 ^4^, 3018ft., [APH_2423, 1/11/1992, 1♂, T.R. Prentice, AMNH]; Powerline Rd. west of Cima, 35.235418 -115.551832 ^5^, 4606ft., [APH_2448, 11/10/1992, 1♀, T.R. Prentice, AMNH]; Powerline Road- 4.6 miles west of Cima, CA, 35.225338 -115.574018 ^4^, 4570ft., [APH_2443, 10/10/1992, 1♂, T.R. Prentice, AMNH]; **Nevada: Clark**: 0.3 miles N Hwy-161 along dirt road, between Jean and Goodsprings, 35.81268 -115.38099 ^1^, 3370ft., [APH_0357, 12/5/2008, 1 juv, Brent E. Hendrixson, Zach Valois, AUMNH]; 0.5 to 3 miles west of Searchlight, Highway 164, 35.474673 -114.953305 ^4^, 3530ft., [APH_2425, 23/10/1976, 1♂, W. Icenogle, AMNH]; [APH_2437, 23/10/1976, 1♂, W. Icenogle, AMNH]; 0.75 miles S Blue Diamond Rd near Bonnie Springs, 36.020622 -115.356856 ^4^, 3300ft., [APH_0304, 20/10/2007, 1♂, Deric Murcer, AUMNH]; 1.5 miles W Searchlight (US-95) on NV-164, 35.472017 -114.944686 ^1^, 3480ft., [APH_0511-0514, 21/5/2009, 4♀, Brent E. Hendrixson, Bernadette DeRussy, Sloan Click, AUMNH]; 2.1 miles W Searchlight (US-95) on Hwy-164 (Nipton Rd), 35.4753 -114.95528 ^1^, 3500ft., [APH_0354-0355, 11/5/2008, 2 juv, Brent E. Hendrixson, Zach Valois, AUMNH]; 2.9 miles SW Hwy-95 on Cold Creek Rd, 36.500528 -115.592032 ^1^, 3790ft., [APH_1216-1218, 1/8/2010, 3♀, Brent E. Hendrixson, Brendon Barnes, Nate Davis, AUMNH]; 3.1 miles E Hwy-160 on Trout Canyon Rd, 36.12435 -115.796265 ^1^, 3646ft., [APH_1211-1212, 1/8/2010, 2♀, Brent E. Hendrixson, Brendon Barnes, Nate Davis, AUMNH]; 4.6 miles W US-95 (Searchlight) on Hwy-164, 35.488073 -114.996602 ^1^, 3690ft., [APH_1219, 2/8/2010, 1♀, Brent E. Hendrixson, Brendon Barnes, Nate Davis, AUMNH]; 5.7 miles E US-95 on Hwy-163, 35.1708 -114.75875 ^1^, 2850ft., [APH_0351-0353, 11/5/2008, 3 juv, Brent E. Hendrixson, Zach Valois, AUMNH]; 8.2 miles W of SL (Searchlight), 4.1 miles N of Hwy 164, 35.562049 -115.084771 ^4^, 4436ft., [AUMS_3270, 7/10/1989, 1♂, T.R. Prentice and W. Icenogle, AUMNH]; 8.2 miles west of Searchlight, on Hwy 164 - road to Highland Spring and Highland Range, 35.510402 -115.055526 ^4^, 4157ft., [APH_2422, 7/10/1989, 1♀, T.R. Prentice, AMNH]; Desert National Wildlife Refuge, near Mormon Well Rd, 36.47675 -115.22656 ^2^, 4963ft., [APH_1579-1581, 4/11/2012, 2♀, 1 juv, Matt Graham, AUMNH]; Searchlight - 8.2 miles west of Searchlight Highway 164, 0.2 miles north of 164, 35.509718 -115.056348 ^4^, 4163ft., [APH_2434, 12/10/1990, 1♀, T.R. Prentice, AMNH]; Searchlight - 8.2 miles west of Searchlight Highway 164, 2.1 miles north toward Highland Range, 35.548501 -115.049262 ^4^, 4551ft., [APH_2432, 12/10/1991, 1♀, T.R. Prentice, AMNH]; Searchlight - 8.2 miles west off highway 164, 35.507713 -115.057128 ^4^, 4163ft., [APH_2424, 12/10/1991, 1♀, T.R. Prentice, AMNH]; [APH_2436, 12/10/1991, 1♀, T.R. Prentice, AMNH]; Searchlight, 8.2 miles west of Searchlight, 1.1 mile north of highway 164, 35.523072 -115.055713 ^4^, 4196ft., [APH_2428, 7/10/1989, 1♀, T.R. Prentice, AMNH]; Searchlight, 35.465268 -114.9197 ^5^, 3551ft., [AUMS_2536, unknown, 1♂, unknown, AUMNH]; **Esmeralda**: 7.7 miles NW Nye County line on US-95, 37.551762 -117.197071 ^1^, 4960ft., [APH_0482-0483, 13/5/2009, 2 juv, Brent E. Hendrixson, Bernadette DeRussy, Sloan Click, Jason Bond, AUMNH]; Highway 95, 8 miles south of Goldfield Summit, 37.565959 -117.200255 ^5^, 5075ft., [APH_2431, 28/10/1978, 1♂, W. Icenogle, AMNH]; **Lincoln**: 0.6 miles N Clark County Line on Carp Rd, 36.852419 -114.374019 ^1^, 3072ft., [APH_1318, 29/7/2011, 1 juv, Brent E. Hendrixson, Brendon Barnes, Nate Davis, Jake Storms, AUMNH]; 8.3 miles S Alamo Canyon Rd on Hwy-93, 37.224912 -115.082582 ^1^, 3160ft., [APH_0783-0786, 9/10/2009, 1♂, 3 juv, Brent E. Hendrixson, Thomas Martin, AUMNH]; along Carp Rd, just south of jct. with Snow Valley Rd, 37.138416 -114.379434 ^1^, 3361ft., [APH_1316-1317, 29/7/2011, 2♀, Brent E. Hendrixson, Brendon Barnes, Nate Davis, Jake Storms, AUMNH]; along Snow Valley Rd, 37.180151 -114.117812 ^1^, 3562ft., [APH_1315, 29/7/2011, 1 juv, Brent E. Hendrixson, Brendon Barnes, Nate Davis, Jake Storms, AUMNH]; **Nye**: 0.8 miles S Hwy-95 along Rd-552, 36.582173 -115.944696 ^1^, 3735ft., [APH_1545-1546, 23/10/2012, 1♀, 1 juv, Brent E. Hendrixson, AUMNH]; 22 miles SW jct. Hwy-267 on US-95, 37.073519 -116.783474 ^1^, 3994ft., [APH_0478-0480, 13/5/2009, 3 juv, Brent E. Hendrixson, Bernadette DeRussy, Sloan Click, Jason Bond, AUMNH]; 6.6 miles SE Esmeralda County Line along Hwy-95, 37.351878 -117.119118 ^1^, 4336ft., [APH_1554, 24/10/2012, 1♀, Brent E. Hendrixson, AUMNH]; along dirt road just E of Hwy-160, 36.409822 -116.066403 ^1^, 3237ft., [APH_1213-1214, 1/8/2010, 1♀, 1 juv, Brent E. Hendrixson, Brendon Barnes, Nate Davis, AUMNH]; Highway 95, 10 miles south of Scotty’s Junction, 37.186208 -116.93858 ^5^, 4039ft., [APH_2429, 28/10/1978, 1♂, W. Icenogle, AMNH]; [APH_2433, 28/10/1978, 1♂, W. Icenogle, AMNH]; Highway 95, 5.5 miles south Lida Junction (Highway 266 turnoff), 37.424395 -117.158462 ^4^, 4629ft., [APH_2453, 28/10/1978, 1♂, W. Icenogle, AMNH]; just NE Death Valley National Park boundary along Hwy-374, 36.832413 -116.8774 ^1^, 3531ft., [APH_1552, 24/10/2012, 1 juv, Brent E. Hendrixson, AUMNH]; Searchlight, 0.5 to 3 miles west on highway 164, 35.474673 -114.953305 ^4^, 3530ft., [APH_2421, 23/10/1976, 1♂, W. Icenogle, AMNH]; [APH_2435, 23/10/1976, 1♂, W. Icenogle, AMNH]; **Utah: Washington**: 3.5 miles north of Utah-Arizona state line - road to Beaver Dam and Summit Springs, 37.04535 -113.593792 ^4^, 2526ft., [APH_2420, 20/10/1993, 1♂, T.R. Prentice, AMNH]; Beaver Dam Mtns, 2.1 miles W Old Hwy-91 on Lytle Ranch Rd, 37.070155 -113.924059 ^1^, 3570ft., [APH_0780-0781, 8/10/2009, 2 juv, Brent E. Hendrixson, Thomas Martin, AUMNH]; Beaver Dam Mtns, Summit Springs, 37.089996 -113.872526 ^5^, 4380ft., [AUMS_2539, 27/8/1992, 1♀, T.R. Prentice, AUMNH]; Beaver Dam Mtns, Summit Springs campsite, 37.152929 -113.886935 ^5^, 6332ft., [APH_2446, 12/10/1993, 1♀, T.R. Prentice, AMNH]; Beaver Dam Mtns, Summit Springs campsite- 1.5 miles west of highway, 37.152929 -113.886935 ^4^, 6332ft., [APH_2445, 12/10/1993, 1♀, T.R. Prentice, AMNH]; Beaver Dam Mtns, Summit Springs, .25 miles below Summit Springs tank, 37.07197 -113.874595 ^4^, 3960ft., [AUMS_3279, 12/10/1993, 1♂, T.R. Prentice, AUMNH]; Beaver Dam Slope, 37.119205 -113.847796 ^6^, 5335ft., [APH_2740, 23/11/1939, 2♂, A.M. Woodbury, AMNH]; Road to Summit Spring - campsite of Summit Spring, Beaver Dam Mtns, 37.115133 -113.864841 ^5^, 5299ft., [APH_2440, 6/10/1993, 1♀, T.R. Prentice, AMNH]; Road to Summit Springs - Beaver Dam Mtns, 3 miles north of Utah-Arizona line, 37.044575 -113.835773 ^5^, 5013ft., [APH_2450, 19/10/1993, 1♂, T.R. Prentice, AMNH]; Road to Summit Springs, 2.7 miles north of UT/AZ line, 37.042419 -113.830789 ^4^, 5276ft., [APH_2451, 19/10/1993, 1♂, T.R. Prentice, AMNH]; Summit Springs off old Hwy 91, 1.86 miles W of Hwy, 37.045214 -113.918874 ^5^, 3181ft., [AUMS_2529, 19/10/1993, 1♂, T.R. Prentice, AUMNH]; vicinity of St. George, 37.095278 -113.578059 ^6^, 2654ft., [APH_2741, 19/10/1939, 1♂, Harold Higgins, AMNH]; Welcome Spring Rd. - 2.35 miles west of Welcome Spring Rd. turnoff, 37.071925 -113.961751 ^4^, 3284ft., [APH_2427, 19/10/1993, 1♂, T.R. Prentice, AMNH]; western portion of Beaver Dam Mtns, 37.120212 -113.954005 ^1^, 3803ft., [APH_1313-1314, 29/7/2011, 2 juv, Brent E. Hendrixson, Brendon Barnes, Nate Davis, Jake Storms, AUMNH].

#### Distribution and natural history.

*Aphonopelma prenticei* has a large range throughout the eastern Mojave (southeastern California, southern Nevada, southwestern Utah, northwestern Arizona) and northwestern Sonoran (western Arizona) deserts at elevations between 350 and 1525 meters. Much of the distribution is east of the Death Valley drainage system with the exception of a few populations along the Panamint Range (Fig. 128). *Aphonopelma prenticei* can be found inhabiting the following Level III Ecoregions: Mojave Basin and Range, Eastern Mojave Basins, Eastern Mojave Low Ranges and Arid Footslopes, Arizona/New Mexico Mountains, and Sonoran Basin and Range. The species distribution model suggests that the habitat is largely unsuitable for these spiders in the surrounding Great Basin Desert, Las Vegas metropolitan area, Amargosa Desert, and Lower Colorado River Valley (Fig. 128B). Additional fieldwork in northwestern Arizona, particularly along the extreme western end of Grand Canyon National Park, is needed to fully assess the distribution of this species. *Aphonopelma prenticei* is syntopic with *Aphonopelma iodius* (California, Nevada, and Utah) and *Aphonopelma chalcodes* (Arizona); its distribution may overlap with *Aphonopelma atomicum* in southern Nye County, Nevada and southeastern Inyo County, California. Burrow entrances are generally surrounded by a distinct mound or turret made of excavated soil and silk (Fig. 2D–E). Mating occurs during daylight hours in autumn (September-November).

#### Conservation status.

*Aphonopelma prenticei* is the most abundant and widespread turret-building tarantula species in the United States and has recently expanded its range into the more northern portions of its distribution (Graham et al. 2015). The species does exhibit high levels of phylogeographic structuring (Hendrixson et al. 2013, Graham et al. 2015) and should be evaluated for the presence of evolutionary significant units, but the conservation status of *Aphonopelma prenticei* is doubtlessly secure.

#### Remarks.

Other important ratios that distinguish females: *Aphonopelma prenticei* possess a larger Cl/M4 (≥1.31; 1.31–1.52) than *Aphonopelma mojave* (≤1.31; 1.20–1.31) and *Aphonopelma joshua* (≤1.25; 1.20–1.25). Certain morphometrics have potential to be useful, though due to the amounts of variation, small number of specimens, and the small differences between species, no other are claimed to be significant at this time (see Suppl. material 2). During evaluation of traditional two-dimensional PCA morphospace and three-dimensional PCA morphospace (PC1~PC2~PC3), males of *Aphonopelma prenticei* separate from *Aphonopelma chalcodes*, *Aphonopelma iodius*, *Aphonopelma joshua*, and *Aphonopelma xwalxwal* along PC1~2, but do not separate from *Aphonopelma atomicum*, *Aphonopelma mojave*, or *Aphonopelma icenoglei*. Female *Aphonopelma prenticei* separate from *Aphonopelma chalcodes*, but do not separate from *Aphonopelma iodius* or any other miniature species (*Aphonopelma atomicum*, *Aphonopelma mojave*, and *Aphonopelma icenoglei*) in two-dimensional morphological space. Females do separate from *Aphonopelma iodius* in three-dimensional morphospace. There are no known female *Aphonopelma xwalxwal* at this time to compare. PC1, PC2, and PC3 explain ≥97% of the variation in all analyses.

### Aphonopelma saguaro

Taxon classificationAnimaliaAraneaeTheraphosidae

Hamilton sp. n.

http://zoobank.org/077A3731-424F-4272-AA31-7617EF896DFB

**Figure 129.**
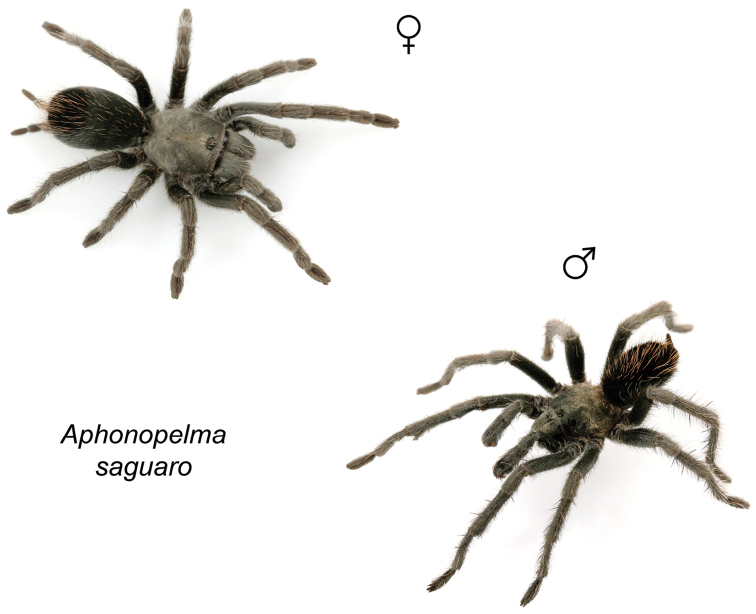
*Aphonopelma saguaro* sp. n. live photographs. Female paratype (L) - APH_3176; Male holotype (R) - APH_3220.

**Figure 130.**
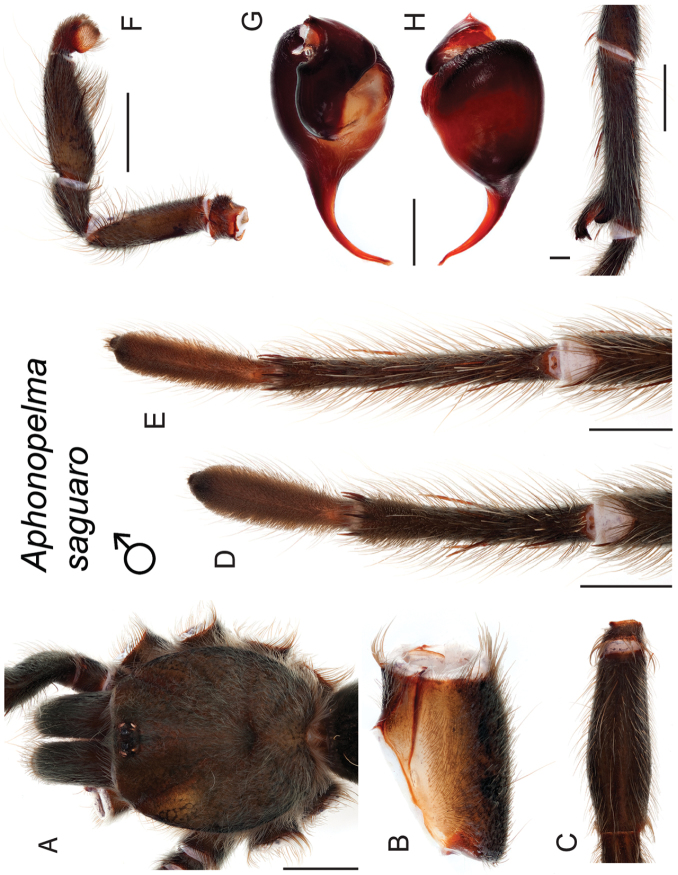
*Aphonopelma saguaro* sp. n. **A–I** male holotype, APH_3220 **A** dorsal view of carapace, scale bar = 2.5mm **B** prolateral view of coxa I **C** dorsal view of femur III **D** ventral view of metatarsus III, scale bar = 2mm **E** ventral view of metatarsus IV, scale bar = 2mm **F** prolateral view of L pedipalp and palpal tibia, scale bar = 2.5mm **G** dorsal view of palpal bulb **H** retrolateral view of palpal bulb, scale bar = 0.5mm **I** prolateral view of tibia I (mating clasper), scale bar = 2mm.

**Figure 131.**
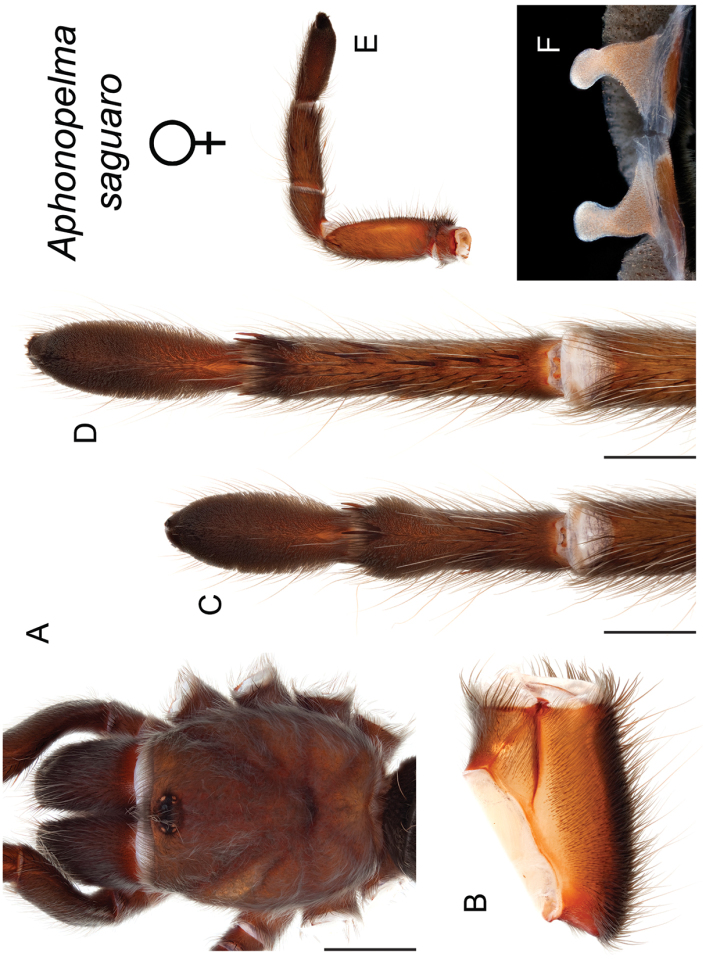
*Aphonopelma saguaro* sp. n. **A–F** female paratype, APH_3176 **A** dorsal view of carapace, scale bar = 3mm **B** prolateral view of coxa I **C** ventral view of metatarsus III, scale bar = 1.5mm **D** ventral view of metatarsus IV, scale bar = 1.5mm **E** prolateral view of L pedipalp and palpal tibia **F** cleared spermathecae.

**Figure 132.**
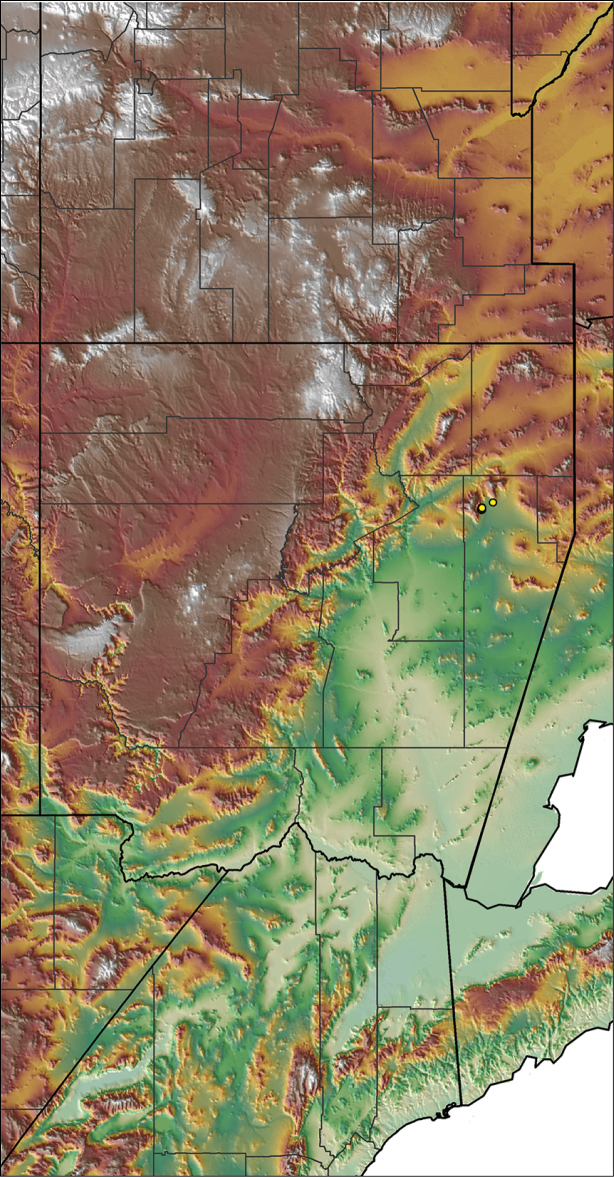
*Aphonopelma saguaro* sp. n. distribution of known specimens. There is no predicted distribution map due to the limited number of sampling localities and restricted distribution this species possesses.

Figures 129 , 130 , 131 , 132 ; Suppl. material 4

#### Types.

Male holotype (APH_3220) collected from Sabino Canyon, Tucson, Pima Co., Arizona, 32.322533 -110.809561 ^2^, elev. 2774ft., 23.xi.2014, coll. Philip MacDuff; deposited in AUMNH. Paratype female (APH_3176) from Saguaro National Park - Saguaro East (Rincon Mountain District), off Cactus Forest Loop Dr., before the Mica View picnic area, Pima Co., Arizona, 32.198568 -110.735614 ^1^, elev. 2944ft., 12.xi.2013, coll. Brent E. Hendrixson and Chris A. Hamilton; deposited in AUMNH. Paratype male (APH_2540) from Sabino Canyon, Tucson, Pima Co., Arizona, 32.316614 -110.81745 ^5^, elev. 2818ft., date unknown, coll. O. Bryant; deposited in AMNH.

#### Etymology.

The specific epithet is a noun in apposition taken from type locality, Saguaro National Park, where this new species can be found in and around the foothills of the Santa Catalina and Rincon Mountains.

#### Diagnosis.

*Aphonopelma saguaro* (Fig. 129) is a member of the *paloma* species group and can be distinguished by a combination of morphological, molecular, and geographic characteristics. Nuclear DNA identifies *Aphonopelma saguaro* as a strongly supported, phylogenetically-distinct lineage (Fig. 8) that is a sister lineage to *Aphonopelma mareki* sp. n., *Aphonopelma parvum* sp. n., and *Aphonopelma superstitionense* sp. n. *Aphonopelma saguaro* can easily be differentiated from syntopic populations of *Aphonopelma chalcodes*, *Aphonopelma catalina*, and *Aphonopelma vorhiesi* by their smaller size, and can be differentiated from other members of the *paloma* species group by locality. Importantly, *Aphonopelma saguaro* males either possess a swollen or slightly swollen femur III, which is different from the normal femur found in *Aphonopelma parvum*. The most significant measurements that distinguish male *Aphonopelma saguaro* from its closely related phylogenetic and syntopic species are F1, Cl, and the extent of scopulation on metatarsus IV. Male *Aphonopelma saguaro* can be distinguished by possessing a larger Cl/M3 (≥1.17; 1.17–1.25) than *Aphonopelma superstitionense* (≤1.12; 1.05–1.12); a smaller F1/T3 (≤1.51; 1.42–1.51) than *Aphonopelma catalina* (≥1.69; 1.69–1.97) and *Aphonopelma paloma* (≥1.54; 1.54–1.69); a smaller Cl/PTw (≤4.27; 3.13–4.27) than *Aphonopelma vorhiesi* (≥4.61; 4.61–5.58); and a smaller L4 scopulation extent (14%–26%) than *Aphonopelma chalcodes* (42%–76%) and *Aphonopelma parvum* (36%–44%). There are no significant measurements that separate male *Aphonopelma saguaro* and *Aphonopelma mareki*. The most significant measurement that distinguishes female *Aphonopelma saguaro* from its closely related phylogenetic and syntopic species is T1. Female *Aphonopelma saguaro* can be distinguished by possessing a larger T1/M4 (1.02 ± (only 1 specimen)) than *superstitionense* (0.84 ± (only 1 specimen)); a smaller F1/T1 (1.13 ± (only 1 specimen)) than *Aphonopelma catalina* (≥1.21; 1.21–1.22), *Aphonopelma chalcodes* (≥1.25; 1.25–1.42), *Aphonopelma marxi* (≥1.19; 1.19–1.27), *Aphonopelma parvum* (≥1.18; 1.18–1.30), *Aphonopelma superstitionense* (1.30 ± (only 1 specimen)), and *Aphonopelma vorhiesi* (≥1.24; 1.24–1.32). There are no significant measurements that separate female *Aphonopelma saguaro* and *Aphonopelma paloma*.

#### Description of male holotype

(APH_3220; Fig. 130). *Specimen preparation and condition*: Specimen collected wandering and preserved in 80% ethanol; original coloration faded due to preservation. Left legs I, III, IV, and left pedipalp removed for measurements and photographs; stored in vial with specimen. Right legs I-IV removed for DNA and stored at -80°C in the AUMNH (Auburn, AL). *General coloration*: Faded black/brown. *Cephalothorax*: Carapace 6.165 mm long, 5.719 mm wide; densely clothed with faded pubescence, appressed to surface; fringe covered in longer setae not closely appressed to surface; foveal groove medium deep and recurved; pars cephalica region rises very gradually from foveal groove on a straight plane towards the ocular area; AER slightly procurved, PER very slightly recurved - mostly straight; normal sized chelicerae; clypeus extends forward on a slight curve; LBl 0.961, LBw 1.148; sternum hirsute, clothed with faded, densely packed, short setae. *Abdomen*: Densely clothed in short black/brown pubescence with numerous longer, lighter setae interspersed (generally red or orange *in situ*); dense dorsal patch of black Type I urticating bristles (Cooke et al. 1972) - smaller and distinct from large species. *Legs*: Hirsute; densely clothed in faded pubescence. Metatarsus I slightly curved. F1 6.232; F1w 1.593; P1 2.444; T1 5.62; M1 4.346; A1 3.053; F3 5.31; F3w 1.985; P3 2.071; T3 4.241; M3 4.952; A3 3.449; F4 6.314; F4w 1.736; P4 2.216; T4 5.541; M4 6.215; A4 3.704; femur III is swollen. All tarsi fully scopulate. Extent of metatarsal scopulation: leg III (SC3) = 38.0%; leg IV (SC4) = 19.2%. Three ventral spinose setae, two prolateral, and one retrolateral spinose seta on metatarsus III; seven ventral spinose setae and one retrolateral on metatarsus IV; two prolateral spinose setae and three ventral spinose setae on tibia I; one ventral spinose seta on patella I; one megaspine present on the retrolateral tibia, at the apex of the mating clasper; two megaspines on the apex on the retrolateral branch of the tibial apophyses. *Coxa I*: Prolateral surface covered by fine, hair-like setae. *Pedipalps*: Hirsute; densely clothed in the same setal color as the other legs, with numerous longer ventral setae; one spinose seta at the apical, prolateral femur; one spinose seta on the prolateral patella; four prolateral spinose setae and one ventral on the palpal tibia; PTl 3.953, PTw 1.445. Palpal bulb is very short and stout. When extended, embolus tapers with a curve to the retrolateral side; embolus slender, no keels; distinct dorsal and ventral transition from bulb to embolus.

**Variation (6).** Cl 4.554–6.165 (5.362±0.25), Cw 4.074–5.719 (4.998±0.23), LBl 0.576–0.961 (0.745±0.06), LBw 0.816–1.148 (0.997±0.05), F1 4.764–6.232 (5.717±0.21), F1w 1.121–1.593 (1.387±0.07), P1 1.732–2.444 (2.106±0.1), T1 4.33–5.674 (5.158±0.2), M1 3.394–4.346 (3.964±0.14), A1 2.542–3.241 (2.949±0.1), L1 length 16.76–21.695 (19.894±0.74), F3 3.941–5.31 (4.772±0.21), F3w 1.268–1.985 (1.666±0.11), P3 1.481–2.071 (1.777±0.09), T3 3.289–4.241 (3.892±0.15), M3 3.643–4.952 (4.449±0.21), A3 2.633–3.581 (3.156±0.15), L3 length 14.985–20.023 (18.046±0.79), F4 4.683–6.314 (5.591±0.23), F4w 1.037–1.736 (1.306±0.09), P4 1.541–2.216 (1.917±0.09), T4 4.435–5.664 (5.152±0.19), M4 4.675–6.393 (5.661±0.25), A4 3.212–3.993 (3.497±0.12), L4 length 18.545–23.99 (21.817±0.82), PTl 3.036–3.953 (3.612±0.14), PTw 1.238–1.644 (1.449±0.06), SC3 ratio 0.306–0.521 (0.402±0.03), SC4 ratio 0.146–0.263 (0.202±0.02), Coxa I setae = fine & thin hair-like, F3 condition = slightly swollen/swollen.

#### Description of female paratype

(APH_3176; Fig. 131). *Specimen preparation and condition*: Specimen collected live from burrow, preserved in 80% ethanol; original coloration faded due to preservation. Left legs I, III, IV, and pedipalp removed for photographs and measurements; stored in vial with specimen. Right legs I-IV removed for DNA and stored at -80°C in the AUMNH (Auburn, AL). Genital plate with spermathecae removed and cleared, stored in vial with specimen. *General coloration*: Faded black/brown. *Cephalothorax*: Carapace 6.86 mm long, 6.033 mm wide; Hirsute, densely clothed with short faded black/brown pubescence closely appressed to surface; fringe densely covered in slightly longer setae; foveal groove medium deep and recurved; pars cephalica region gently rises from thoracic furrow, arching anteriorly toward ocular area; AER slightly procurved, PER very slightly recurved; chelicerae robust, clypeus extends on a curve; LBl 1.087, LBw 1.239; sternum hirsute, clothed with short faded setae. *Abdomen*: Densely clothed dorsally in short faded black setae with longer, lighter setae (generally red or orange *in situ*) focused near the urticating patch; dense dorsal patch of black Type I urticating bristles (Cooke et al. 1972) - smaller and distinct from large species. *Spermathecae*: Paired and separate, with capitate bulbs, short, widening towards the bases; not fused. *Legs*: Hirsute; densely clothed in short faded black/brown pubescence; F1 5.361; F1w 2.073; P1 2.34; T1 4.718; M1 3.253; A1 3.049; L1 length 18.721; F3 4.431; F3w 1.838; P3 2.042; T3 3.361; M3 3.261; A3 3.124; L3 length 16.219; F4 5.828; F4w 1.782; P4 2.412; T4 4.814; M4 4.607; A4 3.521; L4 length 21.182. All tarsi fully scopulate. Extent of metatarsal scopulation: leg III (SC3) = 49.0%; leg IV (SC4) = 21.8%. Two ventral spinose setae and two prolateral spinose setae on metatarsus III; seven ventral spinose setae and one prolateral spinose seta on metatarsus IV. *Coxa I*: Prolateral surface covered by very thin tapered and fine, hair-like setae. *Pedipalps*: Densely clothed in the same setal color as the other legs; one spinose seta on the apical, prolateral femur; five prolateral spinose setae (two at the apical, prolateral border with the tarsus) and one ventral spinose seta on the tibia.

#### Material examined.

**United States: Arizona: Pima**: Sabino Canyon, Tucson, 32.316614 -110.81745 ^5^, 2818ft., [APH_2540, date unknown, 1♂, O. Bryant, AMNH]; 32.3132 -110.811875 ^2^, 2718ft., [APH_3219, 22/11/2014, 1♂, Philip MacDuff, AUMNH]; 32.322533 -110.809561 ^2^, 2774ft., [APH_3220, 23/11/2014, 1♂, Philip MacDuff, AUMNH]; Bear Canyon, Tucson, 32.315814 -110.79015 ^2^, 2857ft., [APH_3221, 3/12/2014, 1♂, Philip MacDuff, AUMNH]; 32.31655 -110.788878 ^2^, 2871ft., [APH_3222, 3/12/2014, 1♂, Philip MacDuff, AUMNH]; 32.314594 -110.794006 ^2^, 2814ft., [APH_3223, 3/12/2014, 1♂, Philip MacDuff, AUMNH]; Saguaro National Park, off Cactus Forest Loop Dr, before Mica View picnic area, 32.198568 -110.735614 ^1^, 2944ft., [APH_3176, 12/11/2013, 1♀, Brent E. Hendrixson, Chris A. Hamilton, AUMNH].

#### Distribution and natural history.

*Aphonopelma saguaro* can be found inhabiting the Madrean Archipelago and Sonoran Basin and Range Level III Ecoregions in southeastern Arizona where their distribution occurs in the foothills and lower canyons of the Santa Catalina and Rincon Mountains (Fig. 132). *Aphonopelma saguaro* can be found in syntopy with *Aphonopelma catalina*, *Aphonopelma chalcodes* and *Aphonopelma vorhiesi*. The breeding season, when mature males abandon their burrows in search of females, occurs during the late fall and early winter (November–December).

#### Conservation status.

*Aphonopelma saguaro* appears to have a limited distribution restricted to the foothills and lower canyons in and around the Santa Catalina and Rincon Mountains. As evidenced by only one specimen previously collected and deposited in a museum collection prior to this study, *Aphonopelma saguaro* can be difficult to find due to the cryptic nature of their burrows and narrow window of activity during the year. The Tucson Metropolitan area is experiencing rapid growth and recreational activities in the Santa Catalina and Rincon Mountains threaten suitable habitat for this species. The status of *Aphonopelma saguaro* seems secure (e.g., protection in Saguaro National Park) but should be monitored.

#### Remarks.

Other important ratios that distinguish males: *Aphonopelma saguaro* possess a larger T3/F4 (≥0.67; 0.67–0.71) than *Aphonopelma paloma* (≤0.64; 0.57–0.64) and *Aphonopelma parvum* (≤0.66; 0.61–0.66); a larger PTl/F4 (≥0.62; 0.62–0.66) than *Aphonopelma chalcodes* (≤0.62; 0.52–0.62), *Aphonopelma superstitionense* (≤0.59; 0.55–0.59), and *Aphonopelma vorhiesi* (≤0.62; 0.55–0.62); a smaller Cl/M3 (≤1.25; 1.17–1.25) than *Aphonopelma catalina* (≥1.26; 1.26–1.42); a smaller F1/T3 (≤1.51; 1.42–1.51) than *Aphonopelma parvum* (≥1.56; 1.56–1.70); a smaller Cl/PTw (≤4.27; 3.13–4.27) than *Aphonopelma chalcodes* (≥5.06; 5.06–6.05) and *Aphonopelma superstitionense* (≥4.40; 4.40–4.72); a smaller L3 scopulation extent (30%–52%) than *Aphonopelma chalcodes* (65%–86%), *Aphonopelma parvum* (60%–65%), and *Aphonopelma vorhiesi* (53%–62%). Other important ratios that distinguish females: *Aphonopelma saguaro* possess a smaller F3L/W (2.41 ± (only 1 specimen)) than *Aphonopelma catalina* (≥2.93; 2.93–2.99), *Aphonopelma chalcodes* (≥2.84; 2.84–3.15), *Aphonopelma mareki* (≥2.66; 2.66–3.00), *Aphonopelma parvum* (≥2.85; 2.85–3.05), *Aphonopelma superstitionense* (2.75 ± (only 1 specimen)), and *Aphonopelma vorhiesi* (≥2.77; 2.77–3.11). Certain morphometrics have potential to be useful, though due to the amounts of variation, small number of specimens, and the small differences between species, no other are claimed to be significant at this time (see Suppl. material 2). During evaluation of traditional two-dimensional PCA morphospace and three-dimensional PCA morphospace (PC1~PC2~PC3), males of *Aphonopelma saguaro* separate from *Aphonopelma catalina*, *Aphonopelma chalcodes*, *Aphonopelma vorhiesi* and *Aphonopelma parvum*, but do not separate from *Aphonopelma paloma*, *Aphonopelma mareki*, or *Aphonopelma superstitionense*. Female *Aphonopelma saguaro* separate from *Aphonopelma catalina*, *Aphonopelma chalcodes*, and *Aphonopelma vorhiesi*, but do not separate from *Aphonopelma mareki*, *Aphonopelma paloma*, *Aphonopelma parvum*, or *Aphonopelma superstitionense* in two-dimensional morphological space, yet when three-dimensional morphospace is plotted, *Aphonopelma saguaro* will separate from *Aphonopelma parvum*. PC1, PC2, and PC3 explain ≥98% of the variation in all analyses. While mtDNA (*CO1*) identifies *saguaro* as a unique and independent lineage, its placement with regards to its sister lineages is not well supported.

### Aphonopelma steindachneri

Taxon classificationAnimaliaAraneaeTheraphosidae

(Ausserer, 1875)

**Figure 133.**
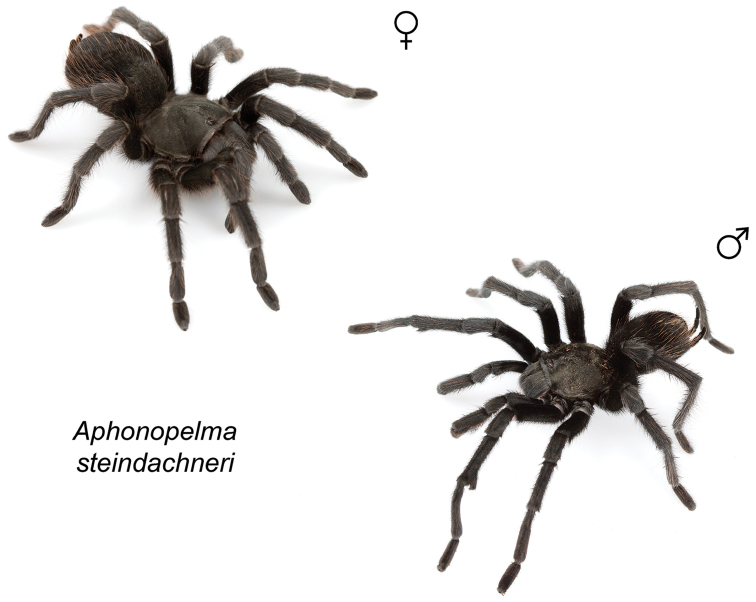
*Aphonopelma steindachneri* (Ausserer, 1875), live photographs. Female (L) - APH_3105; Male neotype (R) - APH_1023.

**Figure 134.**
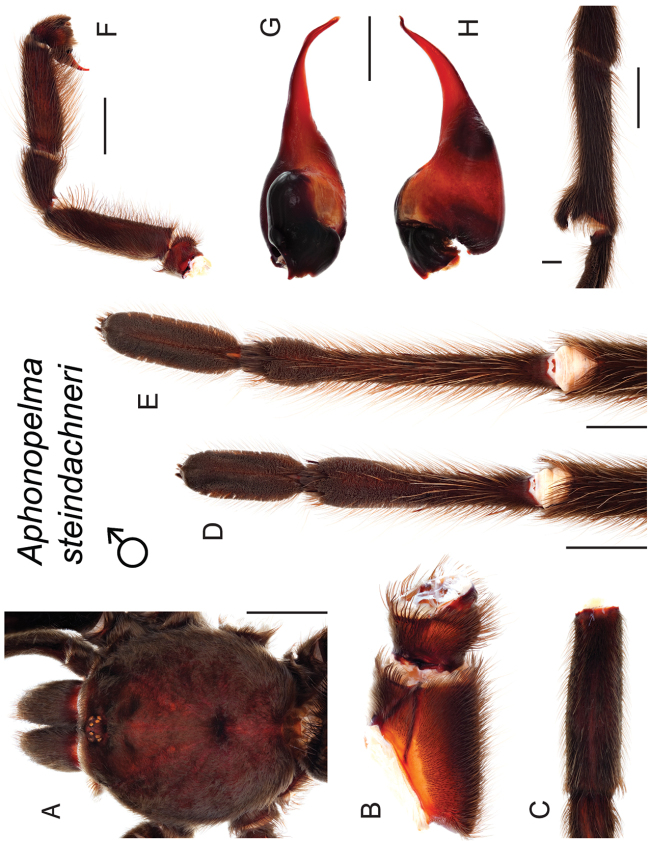
*Aphonopelma steindachneri* (Ausserer, 1875). **A–I** male neotype, APH_1023 **A** dorsal view of carapace, scale bar = 7mm **B** prolateral view of coxa I **C** dorsal view of femur III **D** ventral view of metatarsus III, scale bar = 5mm **E** ventral view of metatarsus IV, scale bar = 3.5mm **F** prolateral view of L pedipalp and palpal tibia, scale bar = 5mm **G** dorsal view of palpal bulb **H** retrolateral view of palpal bulb, scale bar = 1mm **I** prolateral view of tibia I (mating clasper), scale bar = 5.5mm.

**Figure 135.**
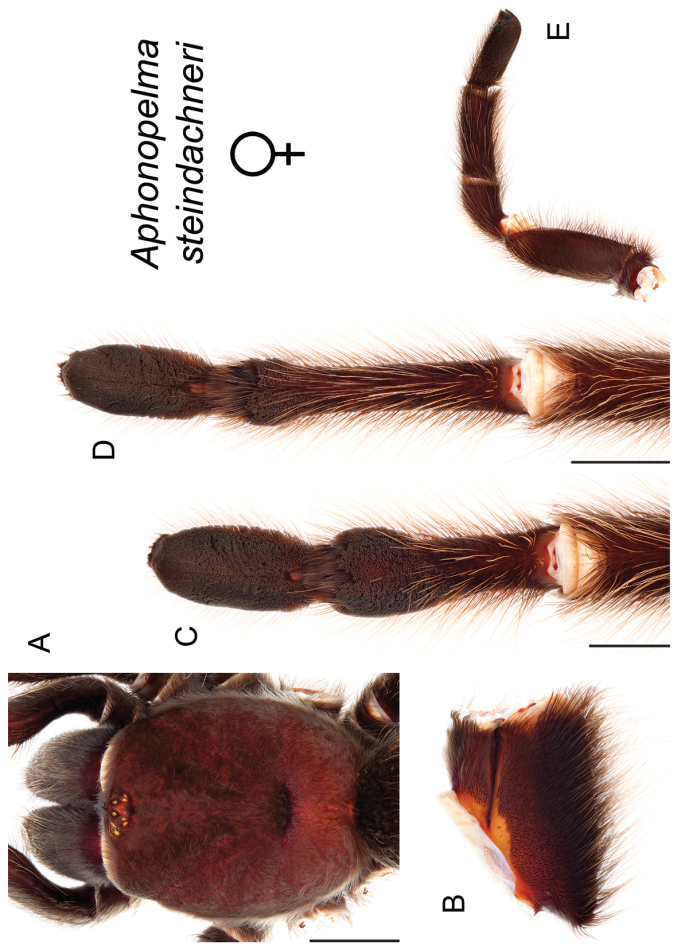
*Aphonopelma steindachneri* (Ausserer, 1875). **A–E** female specimen, APH_1022 **A** dorsal view of carapace, scale bar = 7.5mm **B** prolateral view of coxa I **C** ventral view of metatarsus III, scale bar = 3mm **D** ventral view of metatarsus IV, scale bar = 4.5mm **E** prolateral view of L pedipalp and palpal tibia. Notice the eye deformity, a feature seen in the original species description.

**Figure 136.**
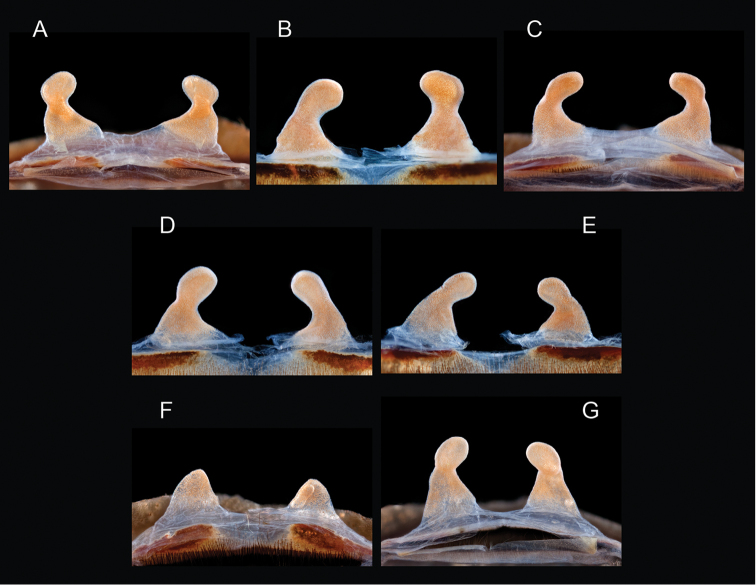
*Aphonopelma steindachneri* (Ausserer, 1875). **A–G** cleared spermathecae **A** *reversum* allotype **B** APH_1022 **C** APH_1030 **D** APH_1034 **E** APH_3104 **F** APH_3105 **G** APH_3098.

**Figure 137.**
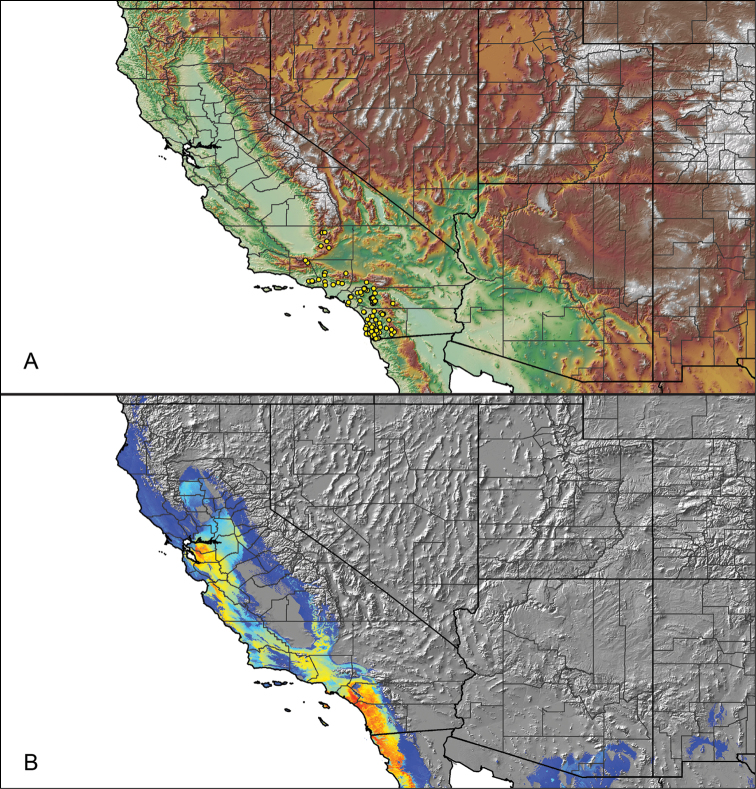
*Aphonopelma steindachneri* (Ausserer, 1875). **A** distribution of known specimens **B** predicted distribution; warmer colors (red, orange, yellow) represent areas of high probability of occurrence, cooler colors (blue shades) represent areas of low probability of occurrence.

Figures 133 , 134 , 135 , 136 , 137

Eurypelma steindachneri Ausserer, 1875: 199; male holotype from Wruck Canyon, SE San Ysidro, San Diego Co., California, 32.548946 -117.006135^4^, elev. 237ft., unknown collection date, coll. Dr. Steindachner; deposited in NHMW. [examined by Prentice *pers. comm*. and see Prentice (1997)]
Delopelma steindachneri Petrunkevitch, 1939: 253.
Rhechostica steindachneri Raven, 1985: 160.
Aphonopelma steindachneri Smith, 1995: 147.
Aphonopelma steindachneri - male neotype designated (APH_1023) from Wruck Canyon, San Ysidro, off Cactus Rd., San Diego Co., California, 32.55002 -116.99192^1^, elev. 398ft., 16.v.2010, coll. Chris A. Hamilton, Xavier Atkinson, Jordan Satler; deposited in AUMNH.
Aphonopelma phanus Chamberlin, 1940: 24; male holotype from Laguna Beach, Orange Co., California, 33.542248 -117.783110^5^; elev. 18ft., vii.1931, coll. unknown; deposited in AMNH. [examined]
Rhechostica phanus Raven, 1985: 149.
Aphonopelma phanus Smith, 1995: 128.
Aphonopelma phanum . **syn. n.**
Aphonopelma reversum Chamberlin, 1940: 8; male holotype, male paratype, three female paratypes from San Diego, San Diego Co., California, 32.715738 -117.161085^6^, elev. 54ft., 1935, coll. unknown; deposited in AMNH. [examined]
Rhechostica reversum Raven, 1985: 149.
Aphonopelma reversum Smith, 1995: 133. **syn. n.**

#### Diagnosis.

*Aphonopelma steindachneri* (Fig. 133) is the only member of the *Steindachneri* species group in the Unite States and can be identified by a combination of morphological, molecular, and geographic characteristics. Nuclear DNA identifies *Aphonopelma steindachneri* as a strongly supported phylogenetically distinct monophyletic lineage, supported as the sister lineage to all other species of *Aphonopelma* in the United States (Fig. 8). *Aphonopelma steindachneri* males and females are easily differentiated from all other species in California by the combination of their reduced scopulation on metatarsi three and four, their black color, and size. The most significant measurement that distinguishes male and female *Aphonopelma steindachneri* from its closely related phylogenetic and syntopic species is the extent scopulation on metatarsus IV. Male *Aphonopelma steindachneri* possess a smaller L4 scopulation extent (21%–31%) than *Aphonopelma eutylenum* (62%–74%), *Aphonopelma iodius* (62%–88%), and *Aphonopelma johnnycashi* sp. n. (70%–76%). Female *Aphonopelma steindachneri* possess a smaller L4 scopulation extent (24%–34%) than *Aphonopelma eutylenum* (62%–75%), *Aphonopelma iodius* (59%–83%), and *Aphonopelma johnnycashi* (67%–82%).

#### Description.

Originally described by Ausserer (1875).

#### Description of male neotype

(APH_1023; Fig. 134). *Specimen preparation and condition*: Specimen collected live from burrow, kept alive to mature, preserved in 80% ethanol; deposited in AUMNH; original coloration faded due to preservation. Left legs I, III, IV, and left pedipalp removed for measurements and photographs; stored in vial with specimen. Right leg III removed for DNA and stored at -80°C in the AUMNH (Auburn, AL). *General coloration*: Black and faded black. *Cephalothorax*: Carapace 17.21 mm long, 16.01 mm wide; Hirsute; densely clothed with faded black iridescent pubescence mostly appressed to surface; fringe covered in long setae not closely appressed to surface; foveal groove medium deep and straight; pars cephalica region rises gradually from foveal groove toward ocular area; AER slightly procurved, PER strongly recurved; normal sized chelicerae; carapace much more round than syntopic species; clypeus slightly extends forward, but mostly straight; LBl 2.01, LBw 2.29; sternum hirsute, clothed with black, densely packed setae. *Abdomen*: Densely clothed in short black pubescence with numerous longer red/orange setae interspersed; possessing a dense dorsal patch of black Type I urticating bristles (Cooke et al. 1972). *Legs*: Hirsute, particularly ventrally; densely clothed in a mix of black or faded black pubescence, femurs are darker. Metatarsus I slightly curved. F1 17.01; F1w 4.03; P1 6.89; T1 14.86; M1 12.95; A1 7.88; F3 13.76; F3w 4.47; P3 6.11; T3 11.59; M3 13.73; A3 7.60; F4 16.28; F4w 4.08; P4 6.25; T4 14.12; M4 18.43; A4 8.42; femur III is slightly swollen. All tarsi fully scopulate. Extent of metatarsal scopulation: leg III (SC3) = 54.2%; leg IV (SC4) = 31.4%. Three ventral spinose setae on metatarsus III; eight ventral spinose setae on metatarsus IV. *Coxa I*: Prolateral surface a mix of fine, hair-like and tapered/thin tapered setae. *Pedipalps*: Hirsute; densely clothed in the same setal color as the other legs, with numerous longer ventral setae; one spinose seta on the apical, prolateral femur and two spinose setae on the prolateral tibia; PTl 9.855, PTw 2.95. When extended, embolus tapers and gently curves to the retrolateral side near apex; embolus very slender, no keels.

**Variation (7).** Cl 12.74–19.08 (15.197±0.84), Cw 12.19–17.33 (14.029±0.75), LBl 1.53–2.20 (1.77±0.09), LBw 1.77–2.91 (2.123±0.15), F1 13.84–17.76 (15.367±0.59), F1w 3.1–4.31 (3.621±0.17), P1 5.69–7.38 (6.231±0.25), T1 12.58–15.12 (13.684±0.4), M1 10.6–14.12 (11.837±0.5), A1 6.64–8.71 (7.49±0.26), L1 length 49.48–63.09 (54.61±1.96), F3 11.58–14.89 (12.794±0.47), F3w 3.43–4.89 (4.053±0.2), P3 4.66–6.22 (5.437±0.23), T3 9.39–11.77 (10.309±0.39), M3 11.31–14.94 (12.511±0.52), A3 6.58–8.72 (7.356±0.27), L3 length 43.72–56.54 (48.407±1.83), F4 13.38–17.36 (14.827±0.58), F4w 3.06–4.52 (3.624±0.2), P4 4.96–6.38 (5.604±0.21), T4 11.63–15.45 (12.823±0.54), M4 15.04–20.16 (16.714±0.73), A4 7.31–9.01 (7.997±0.23), L4 length 52.75–68.36 (57.966±2.26), PTl 8.359–10.514 (9.091±0.31), PTw 2.45–3.05 (2.708±0.08), SC3 ratio 0.401–0.542 (0.479±0.02), SC4 ratio 0.22–0.314 (0.282±0.01), Coxa I setae = tapered/thin tapered, F3 condition = slightly swollen.

#### Description of female exemplar

(APH_1022; Figs 135–136). *Specimen preparation and condition*: Specimen collected live from burrow, preserved in 80% ethanol; deposited in AUMNH; original coloration faded due to preservation. Left legs I, III, IV, and pedipalp removed for photographs and measurements; stored in vial with specimen. Right leg III removed for DNA and stored at -80°C in the AUMNH (Auburn, AL). Genital plate with spermathecae removed and cleared, stored in vial with specimen. *General coloration*: Black/faded black. *Cephalothorax*: Carapace 20.22 mm long, 17.96 mm wide; Hirsute, densely clothed with black/grey pubescence closely appressed to surface; fringe densely covered in longer setae; foveal groove medium deep and slightly procurved; pars cephalica region rises from thoracic furrow more strongly than syntopic species, arching anteriorly toward ocular area; AER and PER not normal due to apparent developmental issues, with what appears to be an extra ALE merged with the ALE on the left side (see Fig. 135A); broad anterior margin of carapace; large chelicerae, clypeus extends forward on a curve; LBl 2.33, LBw 2.77; sternum very hirsute, clothed with dark black setae. *Abdomen*: Densely clothed dorsally in short black setae with numerous longer, lighter setae interspersed (generally red or orange *in situ*); dense dorsal patch of black Type I urticating bristles (Cooke et al. 1972); ventral side with shorter black setae. *Spermathecae*: Paired and separate, tapering and curving medially towards capitate bulbs, relatively shorter than other syntopic species, with wide bases that are not fused. *Legs*: Very hirsute, particularly ventrally; densely clothed in medium and long black/faded black pubescence, femurs darker. F1 14.61; F1w 4.73; P1 7.18; T1 11.86; M1 8.74; A1 6.32; F3 12.18; F3w 4.35; P3 5.78; T3 8.37; M3 9.44; A3 6.33; F4 14.79; F4w 4.49; P4 5.93; T4 11.13; M4 13.10; A4 7.09. All tarsi fully scopulate. Extent of metatarsal scopulation: leg III (SC3) = 60.8%; leg IV (SC4) = 30.7%. Three ventral spinose setae on metatarsus III; ten ventral spinose setae on metatarsus IV. *Coxa I*: Prolateral surface a mix of fine, hair-like and tapered/thin tapered setae. *Pedipalps*: Densely clothed in the same setal color as the other legs; three spinose setae on the prolateral tibia.

**Variation (6).** Cl 16.59–20.22 (18.295±0.53), Cw 14.55–17.96 (16.215±0.52), LBl 1.97–2.43 (2.178±0.09), LBw 2.27–2.77 (2.458±0.09), F1 11.91–14.61 (13.75±0.39), F1w 3.98–4.73 (4.41±0.12), P1 5.57–7.18 (6.538±0.24), T1 8.95–12.03 (11.19±0.47), M1 7.46–8.74 (8.207±0.23), A1 6.24–7.33 (6.608±0.16), L1 length 40.13–48.99 (46.293±1.35), F3 10.46–12.21 (11.417±0.29), F3w 3.71–4.35 (3.967±0.09), P3 4.79–6.50 (5.662±0.23), T3 7.62–8.91 (8.312±0.17), M3 7.87–9.44 (8.897±0.24), A3 6.33–6.93 (6.595±0.1), L3 length 37.25–43.66 (40.882±0.89), F4 12.85–14.79 (13.858±0.29), F4w 3.94–4.49 (4.157±0.08), P4 5.08–6.89 (5.93±0.25), T4 10.37–11.44 (11.083±0.16), M4 11.71–13.73 (12.688±0.32), A4 6.86–7.54 (7.175±0.1), L4 length 47.11–52.78 (50.735±0.89), SC3 ratio 0.513–0.608 (0.574±0.01), SC4 ratio 0.245–0.344 (0.3±0.01), C1 setae = tapered/thin tapered. Spermathecae variation can be seen in Figure 136.

#### Material examined.

**United States: California: Kern**: above Frazier Park Ranger Station, 34.800403 -118.996582 ^5^, 5700ft., [AUMS_3298, 18/7/1987, 1♀, T.R. Prentice, AUMNH]; Caliente Creek, 20 miles N Hwy 58 + 2.5 mils N of Twin Oaks country store, Piute Mtns, 35.345195 -118.386352 ^5^, 3348ft., [AUMS_2603, 11/6/1989, 1♀, T.R. Prentice, AUMNH]; Erskine Creek (Canyon), Erskine Creek road, 35.593637 -118.445971 ^5^, 2933ft., [AUMS_3322, 10/1970, 1♀, J. Anderson, AUMNH]; N of Tehachapi, off Tehachapi Loop (Woodford-Tehachapi Rd), 35.20873 -118.55061 ^1^, 2821ft., [APH_3100, 17/7/2012, 1♀, Chris A. Hamilton, Amy Skibiel, AUMNH]; near Lake Isabella, off Kern River Canyon Rd, near Bodfish and Lake Isabella Blvd intersection, 35.5912 -118.51563 ^1^, 2662ft., [APH_3096-3098, 14/7/2012, 3♀, Chris A. Hamilton, Jim Starrett, Amy Skibiel AUMNH]; S of Tehachapi, off Sand Canyon Rd - off Hwy 58, 35.16025 -118.32507 ^1^, 4144ft., [APH_3099, 17/7/2012, 1♂, Chris A. Hamilton, Amy Skibiel, AUMNH]; **Los Angeles**: 27832 Stonehill Way, Canyon Country, 34.42464 -118.45257 ^2^, 1525ft., [APH_0362, 6/7/2008, 1♂, Anette Pillau, AUMNH]; [APH_0413, 23/8/2008, 1♀, Anette Pillau, AUMNH]; Azusa, 3 miles west in Fish Canyon, 34.135573 -117.940972 ^5^, 581ft., [APH_2694, 23/9/1956, 1♂, V. Roth, AMNH]; Benedict Canyon, Santa Monica Mtns, 34.092508 -118.42897 ^5^, 548ft., [APH_2722, 1967, 3♂, unknown, AMNH]; Canyon Country, Fitch Ave, 34.44963 -118.43426 ^2^, 1820ft., [APH_1632, unknown, 1♀, Anette Pillau, AUMNH]; Chatsworth, 34.250636 -118.61481 ^5^, 1007ft., [APH_2342, 24/7/1962, 1♂, Chamberlin, AMNH]; [APH_2350, 14/8/1966, 1♀, Chamberlin, AMNH]; [APH_2353, 25/9/1966, 1♀, W. Icenogle, AMNH]; corner of Avenue 50 and Figueroa Street, Los Angeles, 34.104913 -118.202204 ^4^, 502ft., [APH_2673, 10/10/1962, 1♂, C. Feldmeth, AMNH]; E of Valyermo (Angeles National Forest), junction of Big Pines Hwy and Big Rock Creek Rd, 34.43123 -117.83439 ^1^, 4074ft., [APH_1044, 30/5/2010, 1♀, Chris A. Hamilton, AUMNH]; Placerita Canyon, 34.373899 -118.450673 ^5^, 2188ft., [APH_2670, 23/9/1965, 1♂, J. Kelley, AMNH]; San Fernando Valley, 34.182785 -118.43996 ^6^, 702ft., [APH_2715, 8/1959, 1♂, unknown, AMNH]; Sierra Madre, 34.161673 -118.052846 ^7^, 955ft., [APH_2465, unknown, 1♂, Chas Camp, AMNH]; **Orange**: Laguna Beach, 0.5 miles from Hwy-133 and CA-1, 33.54846 -117.78093 ^4^, 84ft., [APH_0043, 31/8/2003, 1 juv, Felix Martin, AUMNH]; Limestone Canyon, Limestone Canyon Reserve, 33.764025 -117.710106 ^5^, 806ft., [AUMS_2296, 22/7/1997, 1♀, unknown, AUMNH]; N of Laguna Beach, off Hwy 133, Laguna Coast Wilderness Park, James Dilley Greenbelt Preserve, off Mariposa Trail, 33.59678 -117.75917 ^1^, 350ft., [APH_3104-3105, 20/7/2012, 2♀, Chris A. Hamilton, Amy Skibiel, AUMNH]; San Juan ranger station, up trail toward Chiquito Spring, 33.647262 -117.433319 ^6^, 2537ft., [AUMS_2595, 25/6/1989, 1♂, T.R. Prentice, AUMNH]; Santiago Canyon Road at Limestone SPVR gate, 33.752467 -117.691955 ^6^, 971ft., [AUMS_2630, 22/7/1997, 1♂, unknown, AUMNH]; **Riverside**: 1 mile W of Winchester, Icenogle residence, 33.707049 -117.101768 ^4^, 1461ft., [AUMS_2304, 17/7/1989, 1♂, W. Icenogle, AUMNH]; 2163 Mt. Vernon Ave, Riverside, 33.990875 -117.316626 ^2^, 1372ft., [AUMS_2589, 7/1991, 1♂, T.R. Prentice, AUMNH]; 3231 October Ct, Corona, 33.875017 -117.505452 ^2^, 790ft., [APH_1369, 7/9/2011, 1♂, Alan Bond, AUMNH]; Box Spring Mtns- backside toward 215 Freeway, 33.956553 -117.285374 ^6^, 1923ft., [AUMS_2311, 19/7/1992, 1♂, T.R. Prentice, AUMNH]; Box Spring Mtns, North Side, 34.005987 -117.302515 ^6^, 1773ft., [AUMS_2584, 6/8/1989, 1♂, T.R. Prentice, AUMNH]; Myrtle Rd. and California, Calimesa, 33.9973 -117.03894 ^5^, 2535ft., [AUMS_2648, 8/2002, 1♂, unknown, AUMNH]; Calvert St, Winchester, Green Acres community, W of Hemet, 1/2 mile NE of Hwy 79 and 74 junction, 33.7425 -117.067883 ^4^, 1619ft., [APH_1047, 15/7/2009, 1♀, W. Icenogle, AUMNH]; floor of Icenogle shop (at his home), 33.713802 -117.091508 ^4^, 1545ft., [AUMS_2592, 7/7/1997, 1♂, W. Icenogle, AUMNH]; Gavilon Hills-Amalfi Ave and Loonsberry, 33.82904 -117.379834 ^5^, 1559ft., [AUMS_2307, 25/8/1998, 1♀, M. Blva, AUMNH]; Lake Skinner-below U5 toward lake, 33.603475 -117.056291 ^5^, 2040ft., [AUMS_2312 , 28/9/1997, 1♂, T.R. Prentice, AUMNH]; Lake Skinner, 0.3 miles S of loop road to B7, 33.620919 -117.014227 ^4^, 2068ft., [AUMS_2313, 17/7/1997, 1♂, T.R. Prentice, AUMNH]; Lake Skinner, 0.3 miles W of road to Ultz, 33.571286 -117.067755 ^5^, 1500ft., [AUMS_2305, 17/7/1997, 1♀, T.R. Prentice, AUMNH]; Lake Skinner, 0.4 miles south of Loop Road, 33.569122 -117.060326 ^4^, 1475ft., [AUMS_2253, 18/8/1999, 1♂, T.R. Prentice, AUMNH]; Lake Skinner, just W of B9, No Shore Crossing Road, 33.580192 -117.036497 ^5^, 1516ft., [AUMS_2306, 4/8/1997, 1♀, T.R. Prentice, AUMNH]; Lake Skinner, Rawson Canyon, 0.4 miles S of Loop Rd, 33.596439 -117.022658 ^4^, 1550ft., [AUMS_3302, 4/8/1997, 1♀, unknown, AUMNH]; [AUMS_2303, 4/8/1997, 1♀, T.R. Prentice, AUMNH]; Lake Skinner, Rawson Canyon, 0.45 miles S of loop rd., N side of Black Mtn., 33.631487 -117.004912 ^4^, 2136ft., [AUMS_3314, 21/7/1997, 1♂, T.R. Prentice, AUMNH]; Lake Skinner, Rawson Rd., on way out to Hwy 79, 33.628617 -117.062839 ^5^, 1597ft., [AUMS_2596, 28/9/1997, 1♂, T.R. Prentice, AUMNH]; Lamb Canyon off Hwy 79, ~1 mile N junction Gilman Springs Road, 33.859831 -117.006355 ^5^, 2188ft., [AUMS_2318, 21/3/1989, 1♂, T.R. Prentice, AUMNH]; Mockingbird Canyon (Mockingbird reservoir), 33.891344 -117.412145 ^5^, 981ft., [AUMS_2664, unknown, 1♂, unknown, AUMNH]; Morena Valley, 33.943057 -117.229709 ^6^, 1627ft., [AUMS_2295, 4/8/1997, 1♂, Rob Vellu, AUMNH]; Mt. Vernon Ave, 33.991438 -117.316665 ^5^, 1387ft., [AUMS_2591, 20/8/1991, 1♂, Lisa Fry, AUMNH]; Mt. Vernon area of Riverside, 34.004119 -117.313596 ^5^, 1139ft., [AUMS_2308, 6/7/1997, 1♂, Michael Adams, AUMNH]; S of Hemet, NE of Lake Skinner, off De Portola Rd and Crown Valley Rd, 33.64249 -116.99219 ^1^, 2329ft., [APH_1039, 27/5/2010, 1♀, Chris A. Hamilton, Tom Prentice, AUMNH]; San Jacinto Mtns, .5 miles W of Pinyon Pines Fire Station, .5 miles S Hwy 74 (Ribbonwood CP GR), 33.569305 -116.493135 ^4^, 4469ft., [AUMS_2621, 9/7/2003, 1♂, W. Icenogle, AUMNH]; W side Box Spring Mtns, Mt. Vernon Ave-2163 M. Adams, 33.991205 -117.316559 ^5^, 1380ft., [AUMS_2316, 28/7/1992, 1♂, unknown, AUMNH]; Winchester, 33.706966 -117.084473 ^5^, 1486ft., [APH_2325, 9/8/1968, 1♂, W. Icenogle, AMNH]; [APH_2351, 4/8/1968, 1♂, Chamberlin, AMNH]; [AUMS_2586, 7/1991, 1♂, W. Icenogle, AUMNH]; Winchester, Double Butte area, 33.713817 -117.0916 ^4^, 1558ft., [APH_1048, 16/6/2009, 1♀, W. Icenogle, AUMNH]; Winchester, Grand Ave., .5 miles E of jct of Leon Rd, 33.71436 -117.111327 ^4^, 1508ft., [AUMS_2597, 9/8/1999, 1♂, T.R. Prentice, AUMNH]; Winchester, Icenogle home, 33.706838 -117.084548 ^4^, 1475ft., [AUMS_2299, 5/5/1992, 1♂, W. Icenogle, AUMNH]; [AUMS_2309, 26/7/1997, 1♂, W. Icenogle, AUMNH]; [AUMS_2310, 3/7/1985, 1♀, W. Icenogle, AUMNH]; [AUMS_2598, 17/8/1999, 1♀, W. Icenogle, AUMNH]; [AUMS_2601, 1/9/1997, 1♀, W. Icenogle, AUMNH]; [AUMS_2645, 22/7/1996, 1♂, W. Icenogle, AUMNH]; **San Bernardino**: San Bernardino (San Bernardino National Forest), in hills N of city, off Quail Canyon Rd and Del Rosa, 34.17506 -117.24981 ^1^, 2402ft., [APH_1046, 1/6/2010, 1♀, Chris A. Hamilton, AUMNH]; **San Diego**: 4670 Vista St., San Diego, 32.762575 -117.103569 ^4^, 367ft., [APH_2354, 23/6/1931, 1♀, Major Duncan, AMNH]; 7808 Vista Lazanja, San Diego, 32.978869 -117.161645 ^2^, 327ft., [APH_0441, 18/11/2008, 1 juv, Sandy Lamb, AUMNH]; Camp Pendleton, 33.313998 -117.314552 ^6^, 359ft., [AUMS_2298, 1999, 1♀, Dan, AUMNH]; Camp Pendleton, Santa Margarita Rd., 33.32354 -117.325895 ^5^, 107ft., [AUMS_2599, 17/8/1999, 1♂, T.R. Prentice, AUMNH]; Daley Ranch, Escondido, 33.20605 -117.070667 ^1^, 1550ft., [APH_0987, 9/2009, 1♀, Kyle Dickerson, AUMNH]; E of Alta Rd on Otay Mountain Truck Rd, 32.575022 -116.893226 ^4^, 1524ft., [APH_0178, 8/2007, 1♂, Dorian LaPaglia, AUMNH]; El Cajon, 32.794773 -116.962527 ^5^, 436ft., [APH_2332, 18/7/1930, 1♂, E. Pearson, AMNH]; El Monte County Park, NE of Flinn Springs, off El Monte Park Rd, 32.88992 -116.84796 ^1^, 540ft., [APH_1030, 19/5/2010, 1♀, Chris A. Hamilton, Xavier Atkinson, AUMNH]; Elliot Reserve, Elliot Chaparral Reserve, 32.891876 -117.095075 ^5^, 651ft., [AUMS_2297, 8/8/1997, 1♂, unknown, AUMNH]; Encanto, 32.711739 -117.061755 ^5^, 318ft., [APH_2326, unknown, 1♀, unknown, AMNH]; Escondido, at jct I-15 and Deer Springs Rd, 33.19611 -117.12774 ^5^, 1006ft., [APH_0047, 2/2/2002, 1♀, Garth Hansen, AUMNH]; Jamul, off Jamul Dr, 32.72953 -116.87247 ^1^, 882ft., [APH_0991-0992, 4/5/2010, 2♀, Chris A. Hamilton, AUMNH]; Jamul, off Millar Ranch Rd, next to San Diego National Wildlife Refuge, 32.72892 -116.93993 ^1^, 374ft., [APH_0993, 4/5/2010, 1♀, Chris A. Hamilton, AUMNH]; La Jolla, 32.84722 -117.27333 ^5^, 105ft., [APH_2340, 8/11/1930, 1♂, unknown, AMNH]; [APH_2689, 26/8/1945, 1♂, Gardell Marshall, AMNH]; Lake Morena County Park, off Buckman Springs Rd, 32.684 -116.52038 ^1^, 3091ft., [APH_0994-0995, 5/5/2010, 2♀, Chris A. Hamilton, AUMNH]; MNAS - 6C8, 33.093381 -116.608165 ^7^, 3414ft., [AUMS_2492, 12/8/1997, 1♀, T.R. Prentice, AUMNH]; MNAS - 6C8, W exposure hill slope, 33.093381 -116.608165 ^7^, 3414ft., [AUMS_2593, 6/6/1997, 1♂, unknown, AUMNH]; N of I-8 and E of Alpine, on Viejas Grade Rd, 32.86061 -116.6763 ^1^, 2966ft., [APH_1072, 12/8/2010, 1♂, Peter Scott, AUMNH]; near Lake Henshaw, on Co. Hwy 57, E of Palomar Mtn and W of Lake Henshaw, 33.26411 -116.76653 ^1^, 3347ft., [APH_1074, 14/8/2010, 1♂, Peter Scott, AUMNH]; near Palomar Mountain, on Co. Hwy 57, 33.29249 -116.82854 ^1^, 4559ft., [APH_1073, 14/8/2010, 1♂, Peter Scott, AUMNH]; off Kitchen Creek Rd, N of I-8 (N of Pacific Crest Natl. Scenic Trail), 32.75287 -116.45063 ^1^, 3896ft., [APH_1026-1027, 18/5/2010, 2♀, Chris A. Hamilton, Xavier Atkinson, AUMNH]; Otay, 32.559473 -116.973468 ^6^, 486ft., [APH_2333, 5/7/1930, 1♀, J.L. Keltner, AMNH]; Otay Mesa, San Ysidro, 32.559473 -116.973468 ^5^, 488ft., [AUMS_4192, 30/7/1994, 1♂, T.R. Prentice, AUMNH]; Pine Valley, 32.82144 -116.529184 ^5^, 3763ft., [APH_2347, 25/7/1927, 1♂, Kelsey, AMNH]; Point Loma, 32.70003 -117.246684 ^5^, 318ft., [APH_2323, 1/8/1928, 1♀, Fred L. Keltuer, AMNH]; [APH_2338, 13/4/1936, 1♀, N.L. Johnson, AMNH]; Poway, 32.94456 -117.04873 ^1^, 745ft., [APH_1011-1012, 12/5/2010, 2♀, Chris A. Hamilton, Xavier Atkinson, AUMNH]; Ramona, Eben-Haezer Nevarez chicken farm, 33.00327 -116.87647 ^1^, 1574ft., [APH_1013, 13/5/2010, 1♀, Chris A. Hamilton, Xavier Atkinson, AUMNH]; San Diego, 32.715329 -117.157255 ^6^, 59ft., [APH_2324, 1932, 1♀, unknown, AMNH]; [APH_2327, 17/10/1934, 2♀, unknown, AMNH]; [APH_2330, 1935, 2♂, unknown, AMNH]; [APH_2331, 16/8/1926, 1♂, unknown, AMNH]; [APH_2334, 16/6/1932, 1♀, unknown, AMNH]; [APH_2335, 2/1/1925, 1♀, unknown, AMNH]; [APH_2336, 20/8/1927, 1♀, unknown, AMNH]; [APH_2337, 1/6/1927, 1♀, unknown, AMNH]; [APH_2339, unknown, 2♂, unknown, AMNH]; [APH_2344, 7/8/1932, 1♂, unknown, AMNH]; [APH_2345, unknown, 4♀, unknown, AMNH]; [APH_2346, 27/9/1920, 1♀, unknown, AMNH]; [APH_2348, unknown, 1♂, E. Denton, AMNH]; [APH_2349, 21/5/1932, 1♀, unknown, AMNH]; San Diego Wild Animal Park, 33.09325 -116.98494 ^1^, 642ft., [APH_0396-0397, 31/7/2008, 2♂, Zach Valois, AUMNH]; 54th and Montezuma, San Diego, 32.771159 -117.079432 ^5^, 400ft., [APH_2343, 23/8/1960, 1♂, D. Irwin, AMNH]; San Diego, Mission Trails Regional Park, 32.84078 -117.04901 ^1^, 585ft., [APH_1032, 20/5/2010, 1♂, Chris A. Hamilton, AUMNH]; San Luis Rey picnic area off 76, across San Luis Rey river on E hill slope, 33.249722 -116.7892 ^5^, 2578ft., [AUMS_2300, unknown, 1♂, T.R. Prentice, AUMNH]; San Ysidro area, just W of Cactus Rd., N of Calle la Linea, Wruck Canyon, SW slope, 32.550384 -116.99306 ^5^, 387ft., [AUMS_2600, 14/9/1997, 1♂, T.R. Prentice, AUMNH]; San Ysidro area, Wruck Canyon, W of Cactus Rd, 32.550352 -116.998292 ^5^, 359ft., [AUMS_2293, 16/9/1997, 1♀, T.R. Prentice, AUMNH]; [AUMS_2302, 16/9/1997, 1♀, T.R. Prentice, AUMNH]; San Ysidro, Otay Mesa, 0.5 miles E of school, 32.559473 -116.973468 ^5^, 488ft., [AUMS_2646, 30/7/1994, 1♂, unknown, AUMNH]; Santee, in hills N of where Carlton Hills Blvd ends, 32.86146 -116.99485 ^4^, 780ft., [APH_0145, 9/7/2007, 1 juv, Gilbert Quintana, AUMNH]; [APH_0146, 5/2007, 1♀, Gilbert Quintana, AUMNH]; [APH_0147, 7/7/2007, 1♂, Gilbert Quintana, AUMNH]; [APH_0148, 9/7/2007, 1♂, Gilbert Quintana, AUMNH]; South Coronado Island, 32.685885 -117.183089 ^5^, 7ft., [APH_2328, 19/8/1928, 1♂, C. Searl, AMNH]; SW of San Marcos (Lake San Marcos area), off Rancho Santa Fe Rd and Melrose Dr, 33.1095 -117.22002 ^1^, 538ft., [APH_1033, 21/5/2010, 1♀, Chris A. Hamilton, Xavier Atkinson, AUMNH]; Sweetwater, 32.633742 -117.079092 ^5^, 81ft., [AUMS_2301, 8/unknown, 1♀, unknown, AUMNH]; Sweetwater Reserve/Sweetwater Regional Park, 32.672235 -117.021893 ^5^, 96ft., [AUMS_2294, 9/97, 1♂, unknown, AUMNH]; [AUMS_2602, 13/8/1997, 1♂, unknown, AUMNH]; Torrey Pines State Preserve, 32.91788 -117.25008 ^1^, 352ft., [APH_1034, 21/5/2010, 1♀, Chris A. Hamilton, Xavier Atkinson, AUMNH]; W of Alta Rd, S of Donovan State Prison Rd, 32.5755 -116.920052 ^4^, 625ft., [APH_0179, 31/8/2007, 1♂, Dorian LaPaglia, AUMNH]; Wilderness Gardens Preserve, E of Pala, off Hwy 76 E of I-15, 33.35043 -117.0287 ^1^, 557ft., [APH_1036, 24/5/2010, 1♀, Chris A. Hamilton, AUMNH]; Wruck Canyon, San Ysidro, 32.550352 -116.998292 ^5^, 359ft., [AUMS_3347, 10/8/1998, 1♂, T.R. Prentice, AUMNH]; Wruck Canyon, San Ysidro, off Cactus Rd, 32.55002 -116.99192 ^1^, 398ft., [APH_1022-1024, 16/5/2010, 2♀, 1♂, Chris A. Hamilton, Xavier Atkinson, Jordan Satler, AUMNH]; **Ventura**: 1958 Smokey Ridge Ave, Westlake Village, 34.19675 -118.78803 ^2^, 1349ft., [APH_0012, 14/7/2005, 1♂, Roy Dunn, AUMNH]; Los Padres National Forest, Dry Creek, 8 miles off I-5 thru Hungry Valley, 34.726379 -118.933743 ^5^, 4629ft., [AUMS_3295, 1/8/1987, 1♂, T.R. Prentice, AUMNH]; Los Padres National Forest, off Gold Hill Rd, W of Hungry Valley State Vehicle Recreation Area, 34.72644 -118.92672 ^1^, 4766ft., [APH_1043, 29/5/2010, 1♀, Chris A. Hamilton, AUMNH]; Thousand Oaks, 34.21538 -118.80325 ^1^, 1402ft., [APH_0317, 7/10/2007, 1♀, Anette Pillau, AUMNH]; 1640 Calle Yucca, Thousand Oaks, 34.20203 -118.90031 ^2^, 751ft., [APH_0011, 25/7/2005, 1♂, Tracy Kolnick, AUMNH].

#### Distribution and natural history.

*Aphonopelma steindachneri* is distributed from northern Baja California, north into California along the Southern Californian Coast Ranges, bounded by the Mojave Desert, across the Tehachapi Mountains, and into the southern portions of the Sierra Nevada Mountains (Fig. 137). This species is found in the following Level III Ecoregions: Southern California/Northern Baja Coast, Southern California Mountains, and Sierra Nevada. *Aphonopelma steindachneri* can be found in syntopy with *Aphonopelma eutylenum*, *Aphonopelma iodius*, and *Aphonopelma johnnycashi* across its distribution. The breeding season, when mature males abandon their burrows in search of females, occurs during the summer (July–August).

#### Conservation status.

*Aphonopelma steindachneri* is widely distributed across Southern California and is very common. The species is likely secure although some localized populations in urbanized areas (e.g., Los Angeles and San Diego) are likely threatened by human encroachment and development.

#### Remarks.

Other important ratios that distinguish males: *Aphonopelma steindachneri* possess a smaller L3 scopulation extent (40%–54%) than *Aphonopelma eutylenum* (72%–93%), *Aphonopelma iodius* (78%–96%) and *Aphonopelma johnnycashi* (71%–82%); by possessing a larger T1/F3 (≥1.01; 1.01–1.11) than *Aphonopelma eutylenum* (≤1.00; 0.93–1.00) and *Aphonopelma johnnycashi* (≤1.02; 0.95–1.02, with slight overlap). Other important ratios that distinguish females: *Aphonopelma steindachneri* possess a smaller L3 scopulation extent (51%–61%) than *Aphonopelma eutylenum* (77%–91%), *Aphonopelma iodius* (72%–95%), and *Aphonopelma johnnycashi* (69%–92%); by possessing a smaller M1/M4 (≤0.67; 0.62–0.67) than *Aphonopelma eutylenum* (≥0.67; 0.67–0.78) and *Aphonopelma johnnycashi* (0.70–0.74). For both males and females, certain morphometrics have potential to be useful, though due to the amounts of variation, small number of specimens, and the small differences between species, no others are claimed to be significant at this time (see Suppl. material 2). During evaluation of traditional two-dimensional PCA morphospace and three-dimensional PCA morphospace (PC1~PC2~PC3), male and female *Aphonopelma steindachneri* separate from all of their syntopic species in the *iodius* species group (*Aphonopelma eutylenum*, *Aphonopelma iodius*, and *Aphonopelma johnnycashi*). PC1, PC2, and PC3 explain ≥95% of the variation in all analyses.

Prentice (1997) determined the type locality of *Aphonopelma steindachneri* by examining Dr. Steindachner’s collection notes and through communication with Dr. Jürgen Gruber (NHMW). *Aphonopelma steindachneri* is the oldest name available for this species but the types were apparently lost at some point following Prentice’s (1997) rediscovery and confirmation of them as *Aphonopelma steindachneri*. Prentice examined these types and recorded various measurements, spine patterns, and made note of the deformed eye pattern of the female specimen in the Austria collection. The specimens had been mislabeled, but Prentice was informed that the museum records indicated they had been the types of *Aphonopelma steindachneri*. According to Prentice, every measurement and other descriptive value given by Ausserer (1875) matched precisely those of the specimens in his possession. Prentice had Ausserer’s work translated which indicated that the types collected by Dr. Steindachner were from a site near the Mexican border in San Diego (perhaps Otay Mesa or Wruck Canyon). Based on this information, we have designated a neotype for *Aphonopelma steindachneri* from APH_1023 (see above). *Aphonopelma steindachneri* has been mentioned in the literature numerous times but interestingly, always from localities outside of California. Specimens from New Mexico, redescribed by Smith (1995), were apparently part of the Koch collection now residing at the BMNH but these specimens are not *Aphonopelma steindachneri*. Of particular note, the original *Aphonopelma steindachneri* female described by Ausserer (1875) had an ocular deformity, a condition also present in our female exemplar (APH_1022; see Fig. 135A). This is an uncommon feature in *Aphonopelma* and suggests that perhaps both Ausserer’s and our specimens originated from the same population (Otay Mesa/Wruck Canyon). Additionally, we examined the holotypes and freshly collected topotypic material of *Aphonopelma phanum* and *Aphonopelma reversum*. Our morphological and molecular analyses fail to recognize these two species as separate, independently evolving lineages. As a consequence, we consider *Aphonopelma phanum* and *Aphonopelma reversum* junior synonyms of *Aphonopelma steindachneri*.

### Aphonopelma superstitionense

Taxon classificationAnimaliaAraneaeTheraphosidae

Hamilton, Hendrixson & Bond sp. n.

http://zoobank.org/D475F136-F10C-48D1-9C3D-391816B2BB46

**Figure 138.**
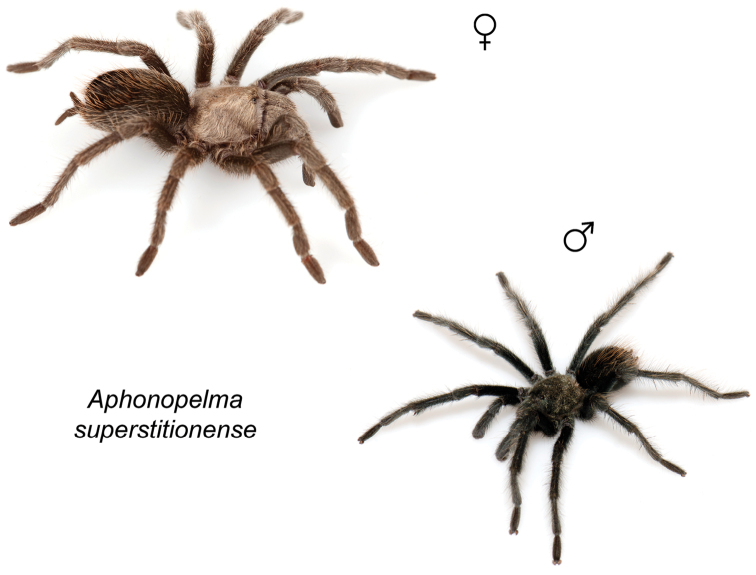
*Aphonopelma superstitionense* sp. n. live photographs. Female paratype (L) - APH_0504; Male (R) - APH_1607.

**Figure 139.**
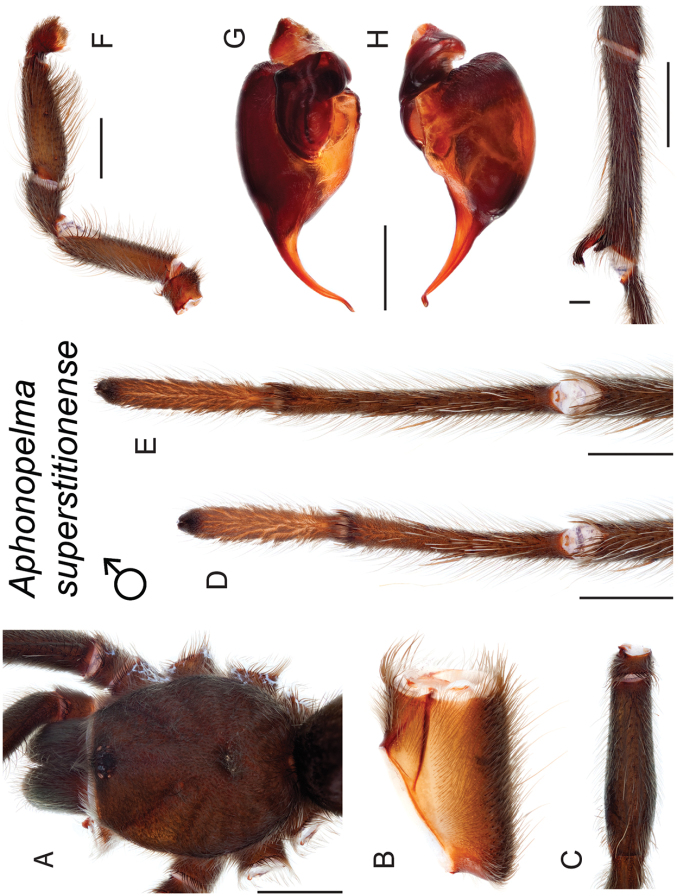
*Aphonopelma superstitionense* sp. n. **A–I** male holotype, APH_1443 **A** dorsal view of carapace, scale bar = 2.5mm **B** prolateral view of coxa I **C** dorsal view of femur III **D** ventral view of metatarsus III, scale bar = 2mm **E** ventral view of metatarsus IV, scale bar = 2mm **F** prolateral view of L pedipalp and palpal tibia, scale bar = 2mm **G** dorsal view of palpal bulb **H** retrolateral view of palpal bulb, scale bar = 0.5mm **I** prolateral view of tibia I (mating clasper), scale bar = 2.5mm.

**Figure 140.**
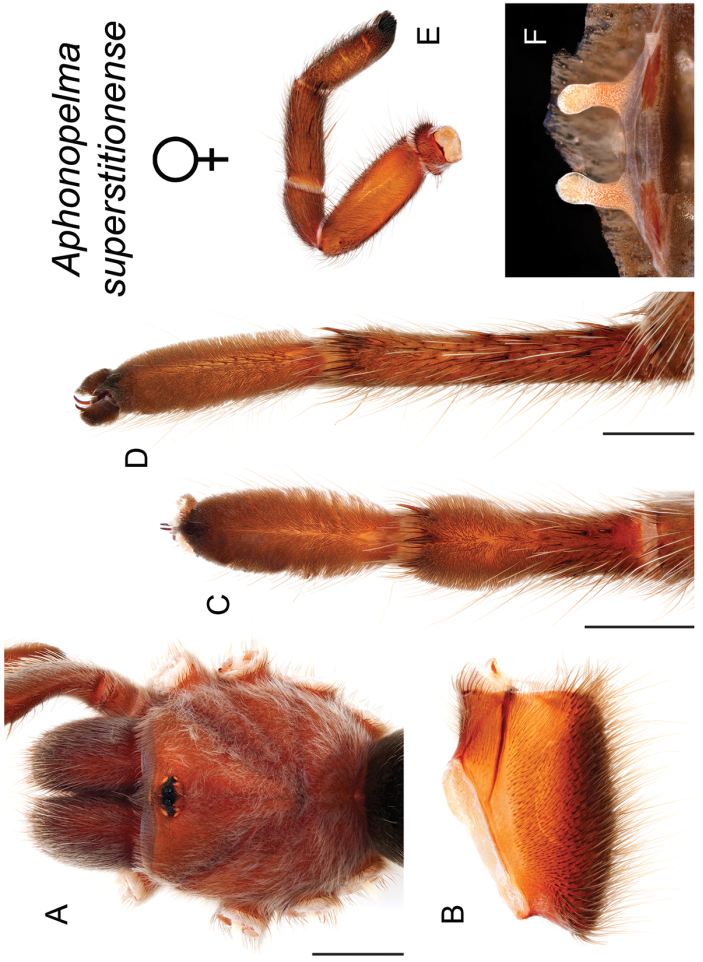
*Aphonopelma superstitionense* sp. n. **A–F** female paratype, APH_0504 **A** dorsal view of carapace, scale bar = 3mm **B** prolateral view of coxa I **C** ventral view of metatarsus III, scale bar = 1.5mm **D** ventral view of metatarsus IV, scale bar = 1.5mm **E** prolateral view of L pedipalp and palpal tibia **F** cleared spermathecae.

**Figure 141.**
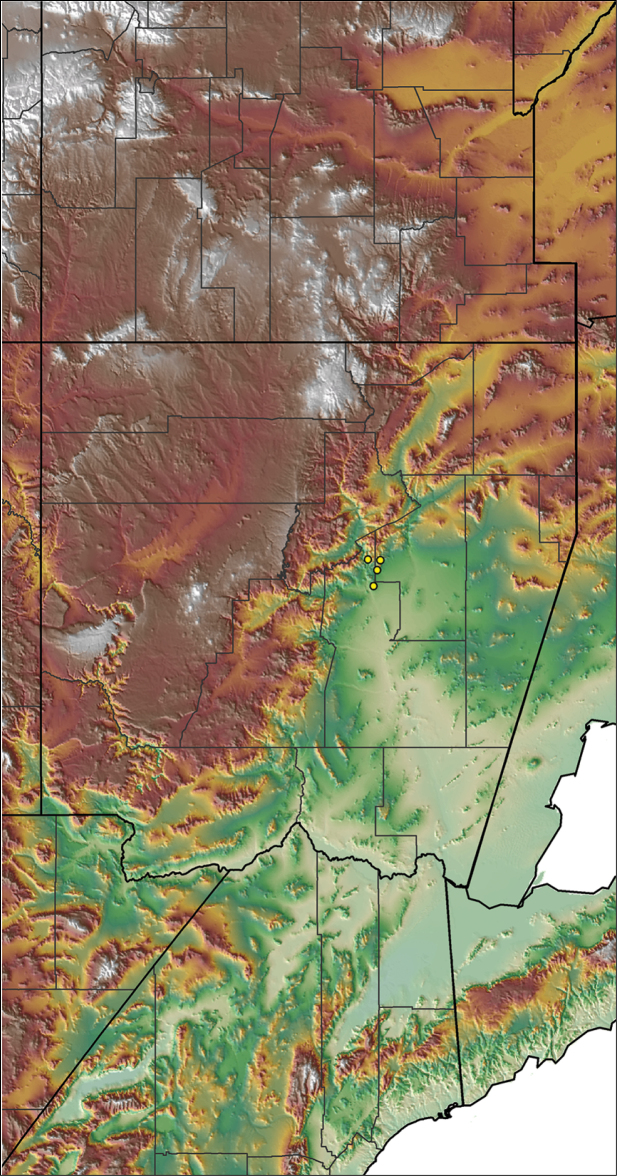
*Aphonopelma superstitionense* sp. n. distribution of known specimens. There is no predicted distribution map due to the limited number of sampling localities and restricted distribution this species possesses.

Figures 138 , 139 , 140 , 141

#### Types.

Male holotype (APH_1443) collected from the Superstition Mtns, Flat Iron, just above Flat Bowl Siphon Draw, 33.440386 -111.457602 ^1^, elev. 3450ft., 1.xii.2011, coll. Tim Cota; deposited in AUMNH. Paratype female (APH_0504) from 0.25 miles S Crimson Rd on Usery Pass Rd., 33.479188 -111.625608 ^1^, elev. 2009ft., 19.v.2009, coll. Brent E. Hendrixson, Bernadette DeRussy, Sloan Click; deposited in AUMNH. Paratype male (APH_1607) from the Superstition Mtns, Peralta Canyon Trail, 33.408937 -111.354086 ^1^, elev. 2982ft., 11.xi.2012, coll. Brent E. Hendrixson, Tim Cota, Molly Taylor; deposited in AMNH.

#### Etymology.

The specific epithet is a neuter adjective taken from type locality, the Superstition Mountains, where this species was first discovered.

#### Diagnosis.

*Aphonopelma superstitionense* (Fig. 138) is a member of the *paloma* species group and can be distinguished by a combination of morphological, molecular, and geographic characteristics. Nuclear DNA identifies *Aphonopelma superstitionense* as a strongly supported, phylogenetically-distinct lineage (Fig. 8) that is a sister lineage to *Aphonopelma mareki* sp. n., *Aphonopelma parvum* sp. n., and *Aphonopelma saguaro* sp. n. *Aphonopelma superstitionense* can easily be differentiated from syntopic populations of *Aphonopelma chalcodes* by its much smaller size, and can be differentiated from other members of the *paloma* species group by locality. Importantly, *Aphonopelma superstitionense* males possess a slightly swollen femur III, which is different from the normal condition of the femur in *Aphonopelma parvum*). The most significant measurements that distinguish male *Aphonopelma superstitionense* from its closely related phylogenetic and syntopic species are Cl and the extent of scopulation on metatarsus IV. Male *Aphonopelma superstitionense* can be distinguished by possessing a smaller Cl/M3 (≤1.12; 1.05–1.12) than *Aphonopelma mareki* (≥1.18; 1.18–1.50), *Aphonopelma paloma* (≥1.17; 1.17–1.32), *Aphonopelma parvum* (≥1.20; 1.20–1.39), and *Aphonopelma saguaro* (≥1.17; 1.17–1.25); and a smaller L4 scopulation extent (14%–20%) than *Aphonopelma chalcodes* (42%–76%) and *Aphonopelma parvum* (36%–44%). The most significant measurements that distinguish female *Aphonopelma superstitionense* from its closely related phylogenetic and syntopic species are T1 and the extent of scopulation on metatarsus IV. Female *Aphonopelma superstitionense* can be distinguished by possessing a smaller T1/M4 (0.84 ± (only 1 specimen)) than *Aphonopelma paloma* (≥0.93; 0.93–1.10), *Aphonopelma parvum* (≥0.91; 0.91–1.00), and *Aphonopelma saguaro* (1.02 ± (only 1 specimen)); and a smaller L4 scopulation extent (26% ± (only 1 specimen)) than *Aphonopelma chalcodes* (56%–81%) and *Aphonopelma parvum* (33%–42%). There are no significant measurements that separate female *Aphonopelma superstitionense* and *Aphonopelma mareki*.

#### Description of male holotype

(APH_1443; Fig. 139). *Specimen preparation and condition*: Specimen collected wandering and preserved in 80% ethanol; original coloration faded due to preservation. Left legs I, III, IV, and left pedipalp removed for measurements and photographs; stored in vial with specimen. Right legs II-IV removed for DNA and stored at -80°C in the AUMNH (Auburn, AL). *General coloration*: Faded black/brown. *Cephalothorax*: Carapace 5.594 mm long, 5.171 mm wide; densely clothed with faded pubescence, appressed to surface; fringe covered in longer setae not closely appressed to surface; foveal groove medium deep and straight; pars cephalica region rises very gradually from foveal groove on a straight plane towards the ocular area; AER slightly procurved, PER very slightly recurved - mostly straight; normal sized chelicerae; clypeus extends forward on a slight curve; LBl 0.824, LBw 1.028; sternum hirsute, clothed with faded, densely packed, short setae. *Abdomen*: Densely clothed in short black/brown pubescence with numerous longer, lighter setae interspersed (generally red or orange *in situ*); dense dorsal patch of black Type I urticating bristles (Cooke et al. 1972) - smaller and distinct from large species. *Legs*: Hirsute; densely clothed in faded pubescence. Metatarsus I slightly curved. F1 6.804; F1w 1.308; P1 2.234; T1 6.002; M1 4.755; A1 3.455; F3 5.475; F3w 1.698; P3 1.878; T3 4.567; M3 5.127; A3 3.571; F4 6.418; F4w 1.335; P4 2.061; T4 5.902; M4 6.571; A4 3.871; femur III is swollen. All tarsi fully scopulate. Extent of metatarsal scopulation: leg III (SC3) = 47.7%; leg IV (SC4) = 20.4%. Three ventral spinose setae and one prolateral spinose seta on metatarsus III; nine ventral spinose setae and one prolateral spinose seta on metatarsus IV (though two are clustered near the row of spinose setae that line the margin with the tarsus); two prolateral spinose setae and two ventral spinose setae on tibia I; one ventral spinose seta on patella I; one megaspine present on the retrolateral tibia, at the apex of the mating clasper; two megaspines on the apex on the retrolateral branch of the tibial apophyses; one megaspine at the base of the prolateral branch of the tibial apophyses. *Coxa I*: Prolateral surface covered by fine, hair-like setae. *Pedipalps*: Hirsute; densely clothed in the same setal color as the other legs, with numerous longer ventral setae; one spinose seta at the apical, prolateral femur; two spinose setae on the prolateral patella; four prolateral spinose setae and one ventral on the palpal tibia; PTl 3.787, PTw 1.236. Palpal bulb is very short and stout. When extended, embolus tapers with a curve to the retrolateral side; embolus slender, no keels; distinct dorsal and ventral transition from bulb to embolus.

**Variation (3).** Cl 5.594–6.586 (5.961±0.31), Cw 5.171–6.302 (5.592±0.36), LBl 0.824–0.93 (0.869±0.03), LBw 1.006–1.044 (1.026±0.01), F1 6.624–7.28 (6.903±0.2), F1w 1.308–1.469 (1.37±0.05), P1 2.234–2.382 (2.32±0.04), T1 5.731–6.501 (6.078±0.23), M1 4.719–4.839 (4.771±0.04), A1 3.387–3.764 (3.535±0.12), L1 length 22.843–24.729 (23.607±0.57), F3 5.475–6.065 (5.708±0.18), F3w 1.592–1.923 (1.738±0.1), P3 1.878–2.021 (1.957±0.04), T3 4.545–4.916 (4.676±0.12), M3 5.127–5.883 (5.473±0.22), A3 3.398–3.622 (3.53±0.07), L3 length 20.618–22.234 (21.345±0.47), F4 6.418–6.966 (6.753±0.17), F4w 1.294–1.538 (1.389±0.08), P4 2.061–2.265 (2.149±0.06), T4 5.902–6.666 (6.193±0.24), M4 6.571–7.264 (6.983±0.21), A4 3.871–3.929 (3.902±0.02), L4 length 24.823–27.09 (25.98±0.65), PTl 3.786–3.951 (3.841±0.05), PTw 1.236–1.394 (1.308±0.05), SC3 ratio 0.404–0.477 (0.429±0.02), SC4 ratio 0.147–0.204 (0.185±0.02), Coxa I setae = very thin tapered, F3 condition = slightly swollen/swollen.

#### Description of female paratype

(APH_0504; Fig. 140). *Specimen preparation and condition*: Specimen collected live from burrow, preserved in 80% ethanol; original coloration faded due to preservation. Left legs I-IV, and pedipalp removed for photographs and measurements; stored in vial with specimen. Right legs III & IV removed for DNA and stored at -80°C in the AUMNH (Auburn, AL). Genital plate with spermathecae removed and cleared, stored in vial with specimen. *General coloration*: Faded black/brown. *Cephalothorax*: Carapace 6.972 mm long, 6.823 mm wide; Very hirsute, densely clothed with short faded black/brown pubescence closely appressed to surface; fringe densely covered in slightly longer setae; foveal groove medium deep and slightly recurved; pars cephalica region gently rises from thoracic furrow, arching anteriorly toward ocular area; AER procurved, PER mostly straight; chelicerae robust, clypeus extends on a curve; LBl 1.139, LBw 1.384; sternum hirsute, clothed with short faded setae. *Abdomen*: Densely clothed dorsally in short faded black setae with longer, lighter setae (generally red or orange *in situ*) focused near the urticating patch; dense dorsal patch of black Type I urticating bristles (Cooke et al. 1972) - smaller and distinct from large species. *Spermathecae*: Paired and separate, with capitate bulbs, short, widening towards the bases; not fused. *Legs*: Hirsute; densely clothed in short faded black/brown pubescence; F1 6.151; F1w 1.943; P1 2.792; T1 4.708; M1 3.561; A1 3.15; L1 length 20.362; F3 4.789; F3w 1.741; P3 2.13; T3 3.566; M3 3.458; A3 3.065; L3 length 17.008; F4 6.308; F4w 1.826; P4 2.677; T4 4.939; M4 5.544; A4 3.807; L4 length 23.275. All tarsi fully scopulate. Extent of metatarsal scopulation: leg III (SC3) = 64.2%; leg IV (SC4) = 26.3%. Three ventral spinose setae and one prolateral spinose seta on metatarsus III; nine ventral spinose setae on metatarsus IV (though two are clustered near the row of spinose setae that line the margin with the tarsus). *Coxa I*: Prolateral surface covered by tapered, thin tapered, and fine, hair-like setae. *Pedipalps*: Densely clothed in the same setal color as the other legs; one spinose seta on the apical, prolateral femur; five prolateral spinose setae (two at the apical, prolateral border with the tarsus) and two ventral spinose setae on the tibia (one at the apical, prolateral border with the tarsus).

#### Material examined.

**United States: Arizona: Maricopa**: 0.25 miles S Crimson Rd on Usery Pass Rd, 33.479188 -111.625608 ^1^, 2009ft., [APH_0504, 19/5/2009, 1♀, Brent E. Hendrixson, Bernadette DeRussy, Sloan Click, AUMNH]; Apache Trail, 33.540608 -111.345427 ^2^, 2651ft., [APH_1643, 29/11/2012, 1♂, Tim Cota, AUMNH]; **Pinal**: Superstition Mtns, Flat Iron, just above Flat Bowl Siphon Draw, 33.440386 -111.457602 ^1^, 3450ft., [APH_1443, 1/12/2011, 1♂, Tim Cota, AUMNH]; Superstition Mtns, Peralta Canyon Trail, 33.408937 -111.354086 ^1^, 2982ft., [APH_1607, 11/11/2012, 1♂, Brent E. Hendrixson, Tim Cota, Molly Taylor, AMNH].

#### Distribution and natural history.

*Aphonopelma superstitionense* can be found inhabiting the Arizona/New Mexico Mountains and Sonoran Basin and Range Level III Ecoregions of central Arizona east of the Phoenix Metropolitan Area (Fig. 141), specifically in and around the foothills of the Superstition Mountains and may be present in or near the Pinal Mountains. *Aphonopelma superstitionense* can be found in syntopy with *Aphonopelma chalcodes* and perhaps *Aphonopelma marxi* at higher elevation. The breeding season, when mature males abandon their burrows in search of females, occurs during the late fall and early winter (November–December).

#### Conservation status.

*Aphonopelma superstitionense* appears to have a limited distribution restricted to the foothills and area in or around the Superstition Mountains. These diminutive tarantulas are probably common but can be incredibly difficult to find due to the cryptic nature of their burrows and narrow window of activity during the year. The Phoenix Metropolitan area is experiencing rapid growth and recreational activities in the Superstition Mountains threaten suitable habitat for this species. The status of *Aphonopelma superstitionense* seems secure (e.g., it is probably common in remote sections of the Superstition Wilderness Area) but should be monitored.

#### Remarks.

Other important ratios that distinguish males: *Aphonopelma superstitionense* possess a smaller PTl/M3 (≤0.74; 0.67–0.74) than *Aphonopelma mareki* (≥0.76; 0.76–0.92), *Aphonopelma paloma* (≥0.77; 0.77–0.85), *Aphonopelma parvum* (≥0.81; 0.81–0.88), and *Aphonopelma saguaro* (≥0.78; 0.78–0.84); a smaller L3 scopulation extent (40%–48%) than *Aphonopelma chalcodes* (65%-86%), *Aphonopelma mareki* (50%–56%), and *Aphonopelma parvum* (60%–65). Other important ratios that distinguish females: *Aphonopelma superstitionense* possess a smaller M3/M4 (0.62 ± (only 1 specimen)) than *Aphonopelma mareki* (≥0.66; 0.66–0.77), *Aphonopelma parvum* (≥0.67; 0.67–0.74), and *Aphonopelma saguaro* (0.70 ± (only 1 specimen)); a smaller Cl/M4 (1.25 ± (only 1 specimen)) than *Aphonopelma paloma* (≥1.44; 1.44–1.60); a larger L1/L3 (1.19 ± (only 1 specimen)) than *Aphonopelma parvum* (≥1.09; 1.09–1.14); a larger F1/T1 (1.30 ± (only 1 specimen)) than *Aphonopelma saguaro* (1.13 ± (only 1 specimen)). Certain morphometrics have potential to be useful, though due to the amounts of variation, small number of specimens, and the small differences between species, no other are claimed to be significant at this time (see Suppl. material 2). During evaluation of traditional two-dimensional PCA morphospace and three-dimensional PCA morphospace (PC1~PC2~PC3), males of *Aphonopelma superstitionense* separate from *Aphonopelma chalcodes* and *Aphonopelma parvum*, but do not separate from *Aphonopelma mareki*, *Aphonopelma paloma*, or *Aphonopelma saguaro*. Female *Aphonopelma superstitionense* separate from *Aphonopelma chalcodes*, but do not separate from *Aphonopelma mareki*, *Aphonopelma paloma*, *Aphonopelma parvum*, or *Aphonopelma saguaro* in two-dimensional morphological space, yet when three-dimensional morphospace is plotted, *Aphonopelma superstitionense* appears to separate from *Aphonopelma parvum* (though we only have 1 female *Aphonopelma superstitionense* to compare). PC1, PC2, and PC3 explain ≥99% of the variation in all analyses. Mitochondrial DNA (*CO1*) identifies *Aphonopelma superstitionense* as a polyphyletic group with some members grouping with the *Aphonopelma mareki* lineage (Fig. 7). Both species were previously identified as putative novel species (Hamilton et al. 2014). Nuclear DNA identifies what we feel is a more accurate evolutionary history of the *Aphonopelma superstitionense* lineage and highlights how *CO1* is not effective at accurately delimiting species boundaries within this group possibly due to mitochondrial introgression.

### Aphonopelma vorhiesi

Taxon classificationAnimaliaAraneaeTheraphosidae

(Chamberlin & Ivie, 1939)

**Figure 142.**
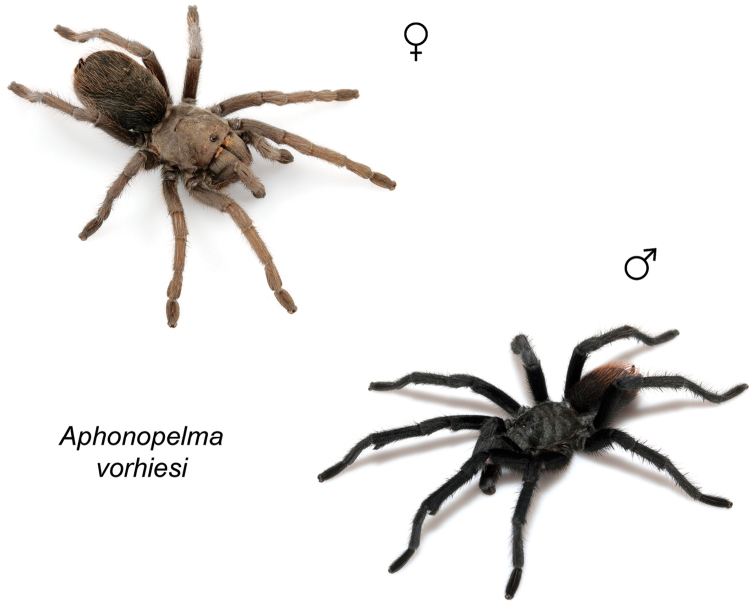
*Aphonopelma vorhiesi* (Chamberlin & Ivie, 1939), live photographs. Female (L) - APH_3188; Male (R) - APH_1520.

**Figure 143.**
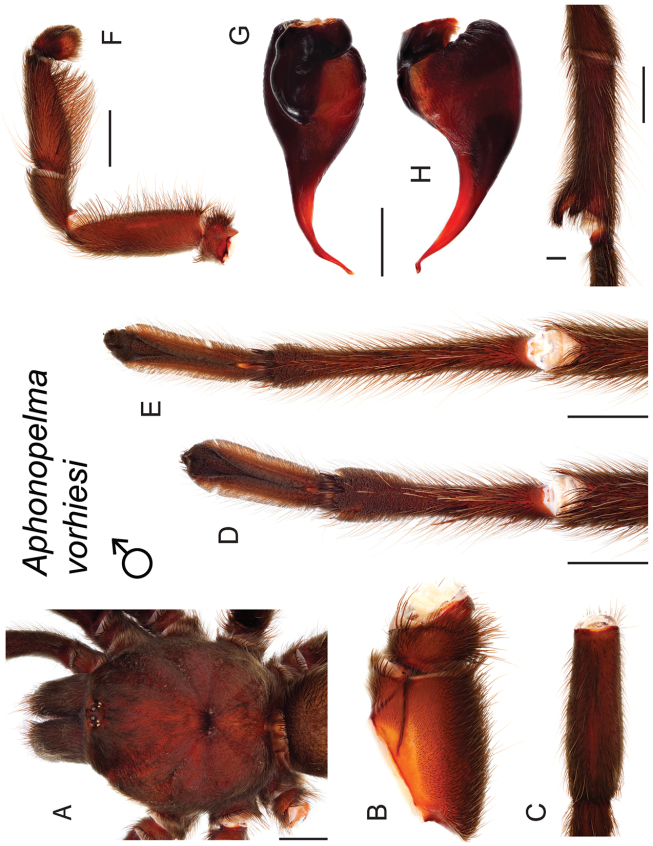
*Aphonopelma vorhiesi* (Chamberlin & Ivie, 1939). **A–I** male specimen, APH_0177 **A** dorsal view of carapace, scale bar = 3.5mm **B** prolateral view of coxa I **C** dorsal view of femur III **D** ventral view of metatarsus III, scale bar = 4mm **E** ventral view of metatarsus IV, scale bar = 4mm **F** prolateral view of L pedipalp and palpal tibia, scale bar = 4mm **G** dorsal view of palpal bulb **H** retrolateral view of palpal bulb, scale bar = 1mm **I** prolateral view of tibia I (mating clasper), scale bar = 4mm.

**Figure 144.**
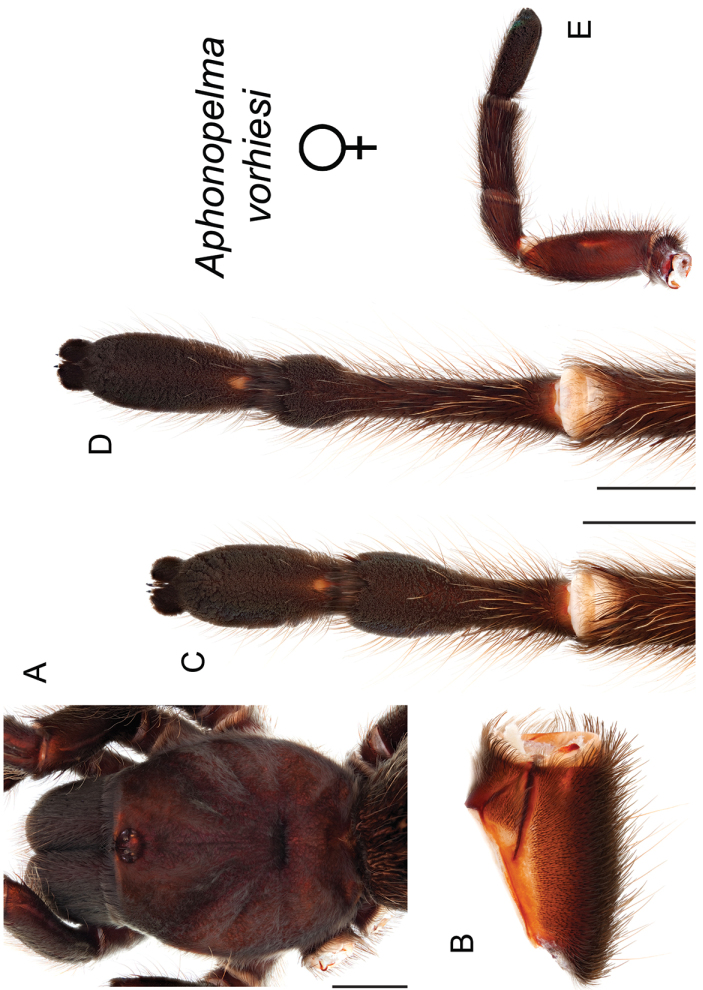
*Aphonopelma vorhiesi* (Chamberlin & Ivie, 1939). **A–E** female specimen, APH_1488 **A** dorsal view of carapace, scale bar = 5mm **B** prolateral view of coxa I **C** ventral view of metatarsus III, scale bar = 4mm **D** ventral view of metatarsus IV, scale bar = 3.5mm **E** prolateral view of L pedipalp and palpal tibia.

**Figure 145.**
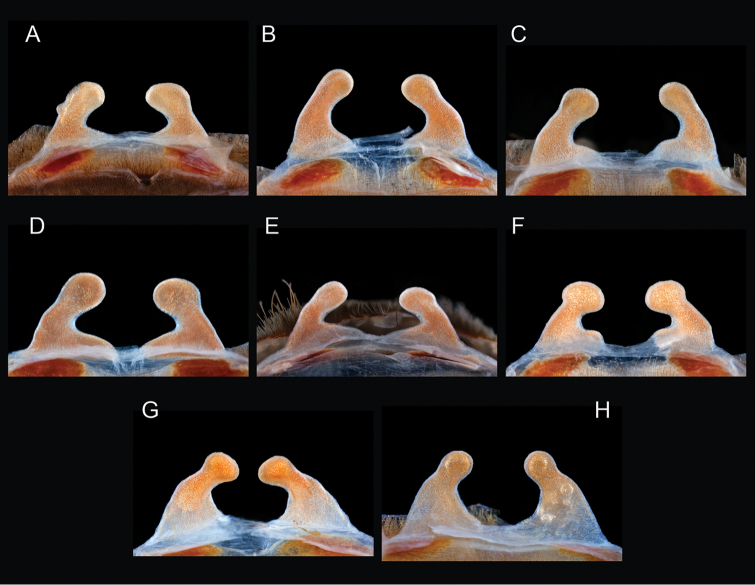
*Aphonopelma vorhiesi* (Chamberlin & Ivie, 1939). **A–H** cleared spermathecae **A** APH_0639 **B** APH_0640 **C** APH_0674 **D** APH_0886 **E** APH_1488 **F** APH_1506 **G** Jung’s “*cochise*” female paratype **H** APH_2137, a Jung “*cochise*” specimen.

**Figure 146.**
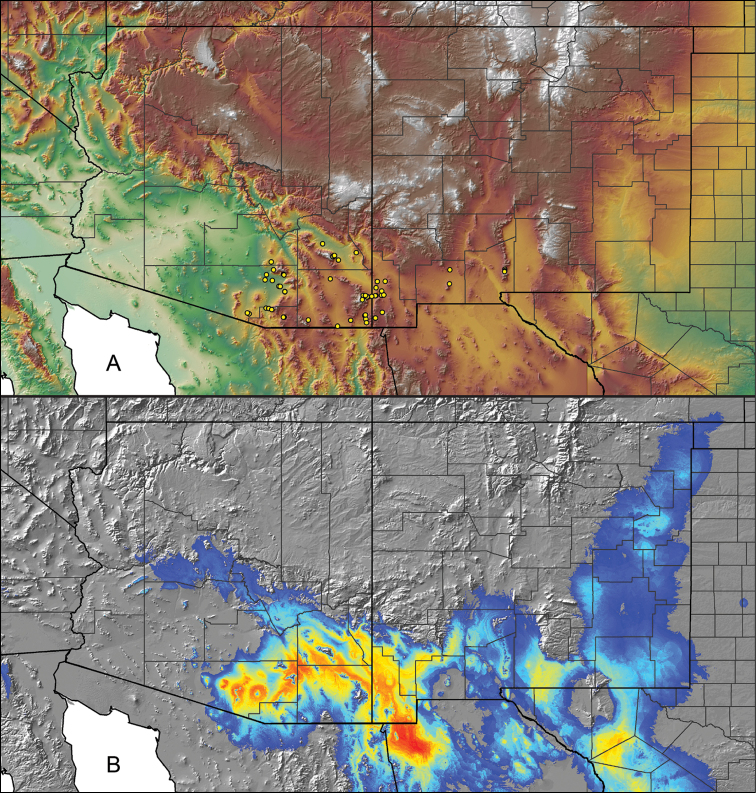
*Aphonopelma vorhiesi* (Chamberlin & Ivie, 1939). **A** distribution of known specimens **B** predicted distribution; warmer colors (red, orange, yellow) represent areas of high probability of occurrence, cooler colors (blue shades) represent areas of low probability of occurrence.

Figures 142 , 143 , 144 , 145 , 146 ; Suppl. material 4

Delopelma vorhiesi Chamberlin & Ivie, 1939: 7; male holotype and male paratype from Tucson, Pima Co., Arizona, 32.221743 -110.926479^6^, elev. 2473ft., no collecting date, coll. Prof. C.T. Vorhies; deposited in AMNH. [examined]
Rhechostica vorhiesi Raven, 1985: 149.
Aphonopelma vorhiesi Smith, 1995: 155.
Aphonopelma jungi Smith, 1995: 116; male holotype from Portal Rd., Portal, Cochise Co., Arizona, 31.914142 -109.141676^5^, elev. 4761ft., viii.1992, coll. A. Smith and Michael Sullivan; deposited in BMNH. [examined] **syn. n.**
Aphonopelma punzoi Smith, 1995: 131; male holotype from Swift Trail (Jct), 5 miles S of Safford, Graham Co., Arizona, 32.729897 -109.713921^5^, elev. 3161ft., mid-August, coll. A. Smith and Michael Sullivan; deposited in BMNH. [examined] **syn. n.**

#### Diagnosis.

*Aphonopelma vorhiesi* (Fig. 142) belongs to the *Marxi* species group and can be identified by a combination of morphological, molecular, and geographic characteristics. Nuclear DNA identifies *Aphonopelma vorhiesi* as a strongly supported monophyletic lineage (Fig. 8). It can easily be distinguished from syntopic populations of *Aphonopelma parvum* sp. n., *Aphonopelma paloma*, and *Aphonopelma saguaro* sp. n. by its larger size. The most significant measurements that distinguish male *Aphonopelma vorhiesi* from its closely related phylogenetic and syntopic species are PTl and the extent of scopulation on metatarsus III. Male *Aphonopelma vorhiesi* can be distinguished by possessing a smaller PTl/M1 (≤0.90; 0.76–0.90) than *Aphonopelma catalina* sp. n. (≥0.90; 0.90–1.03), *Aphonopelma chiricahua* sp. n. (≥0.96; 0.96–1.18), and *Aphonopelma madera* sp. n. (≥0.98; 0.98–1.12), and larger than *Aphonopelma chalcodes* (≤0.75; 0.67–0.75) and *Aphonopelma gabeli* (≤0.68; 0.61–0.68); and a smaller L3 scopulation extent (44%-62%) than *Aphonopelma chalcodes* (76%–86%) and *Aphonopelma hentzi* (69%–86%). There are no significant measurements that separate male *Aphonopelma vorhiesi* from *Aphonopelma peloncillo* sp. n. Significant measurements that distinguish female *Aphonopelma vorhiesi* from its closely related phylogenetic and syntopic species are Cl and the extent of scopulation on metatarsus IV. Female *Aphonopelma vorhiesi* can be distinguished by possessing a larger Cl/Cw (≥1.11; 1.11–1.21) than *Aphonopelma catalina* (≤1.09; 1.07–1.09) and *Aphonopelma chiricahua* (1.02 ± (only 1 specimen)); and by possessing a smaller L4 scopulation extent (26%–37%) than *Aphonopelma chalcodes* (63%–81%), *Aphonopelma gabeli* (39%–53%) and *Aphonopelma hentzi* (42%–72%). There are no significant measurements that separate female *Aphonopelma vorhiesi* from *Aphonopelma peloncillo* and *Aphonopelma madera*.

#### Description.

Male originally described by Chamberlin and Ivie (1939).

#### Redescription of male exemplar

(APH_0177; Fig. 143). *Specimen preparation and condition*: Specimen collected live crossing road, preserved in 80% ethanol; deposited in AUMNH; original coloration faded due to preservation. Left legs I, III, IV, and left pedipalp removed for measurements and photographs; stored in vial with specimen. Right leg III removed for DNA and stored at -80°C in the AUMNH (Auburn, AL). *General coloration*: Black and faded brown. *Cephalothorax*: Carapace 13.61 mm long, 12.95 mm wide; Hirsute; densely clothed with black, slightly iridescent, pubescence mostly appressed to surface; fringe covered in long setae not closely appressed to surface; foveal groove medium deep and slightly recurved; pars cephalica region rises gradually from foveal groove, gently arching anteriorly toward ocular area; AER slightly procurved, PER slightly recurved; normal sized chelicerae; clypeus extends forward on a curve; LBl 1.48, LBw 1.88; sternum hirsute, clothed with short black, densely packed setae. *Abdomen*: Densely clothed in short black pubescence with numerous longer red/orange setae interspersed; possessing a dense dorsal patch of black Type I urticating bristles (Cooke et al. 1972). *Legs*: Hirsute; densely clothed in a mix of short and medium length black or faded black pubescence, slightly longer ventrally. Metatarsus I mostly straight. F1 13.70; F1w 3.62; P1 5.85; T1 11.31; M1 10.20; A1 6.65; F3 11.52; F3w 4.09; P3 4.41; T3 9.25; M3 10.89; A3 6.97; F4 13.94; F4w 3.64; P4 4.56; T4 11.74; M4 14.66; A4 7.83; femur III is swollen. All tarsi fully scopulate. Extent of metatarsal scopulation: leg III (SC3) = 55.9%; leg IV (SC4) = 28.2%. Two ventral spinose setae on metatarsus III; seven ventral spinose setae on metatarsus IV; one large ventral spinose seta at the base of the tibia I near the patella; one large megaspine is present on the retrolateral tibia at the apex of the mating clasper - this can be seen when viewing the prolateral face of the mating clasper. *Coxa I*: Prolateral surface a mix of fine, hair-like and thin tapered setae. *Pedipalps*: Hirsute; densely clothed in the same setal color as the other legs, with numerous longer ventral setae; one spinose seta on the apical, prolateral femur; one spinose seta on the prolateral patella; four spinose setae on the prolateral tibia; PTl 7.791, PTw 2.61. When extended, embolus tapers and gently curves to the retrolateral side; bulb is shorter and more stout than *chalcodes*.

**Variation (11).** Cl 10.018–13.98 (11.663±0.4), Cw 9.18–12.95 (10.722±0.4), LBl 1.11–1.658 (1.404±0.06), LBw 1.24–1.921 (1.62±0.07), F1 10.15–14.51 (11.867±0.42), F1w 2.51–3.62 (2.917±0.1), P1 4.1–5.88 (4.885±0.18), T1 8.69–12.39 (10.09±0.36), M1 6.877–10.20 (8.296±0.3), A1 4.74–6.70 (5.584±0.21), L1 length 35.23–49.06 (40.541±1.51), F3 8.37–11.57 (9.858±0.34), F3w 2.65–4.09 (3.18±0.14), P3 3.3–4.47 (3.794±0.12), T3 6.49–9.25 (7.619±0.28), M3 7.717–10.89 (8.927±0.34), A3 5.35–6.97 (6.075±0.17), L3 length 31.4–43.04 (36.647±1.26), F4 10.09–13.94 (11.696±0.41), F4w 2.32–3.64 (2.831±0.13), P4 3.46–4.56 (4.129±0.11), T4 8.996–11.74 (10.128±0.31), M4 10.33–14.66 (12.131±0.44), A4 5.43–7.83 (6.606±0.25), L4 length 38.39–52.73 (44.469±1.57), PTl 6.167–8.125 (6.983±0.18), PTw 1.919–2.611 (2.334±0.06), SC3 ratio 0.45–0.619 (0.554±0.01), SC4 ratio 0.201–0.358 (0.3±0.01), Coxa 1 setae = tapered/thin tapered, F3 condition = slightly swollen.

#### Description of female exemplar

(APH_1488; Figs 144–145). *Specimen preparation and condition*: Specimen collected live from burrow, preserved in 80% ethanol; deposited in AUMNH; original coloration faded due to preservation. Left legs I, III, IV, and pedipalp removed for photographs and measurements; stored in vial with specimen. Right leg III removed for DNA and stored at -80°C in the AUMNH (Auburn, AL). Genital plate with spermathecae removed and cleared, stored in vial with specimen. *General coloration*: Black/faded black. *Cephalothorax*: Carapace 16.38 mm long, 14.63 mm wide; Hirsute, densely clothed with black/faded black, slightly iridescent, pubescence closely appressed to surface; fringe densely covered in longer setae; foveal groove medium deep and straight; pars cephalica region gently rises from thoracic furrow, arching anteriorly toward ocular area; AER procurved, PER recurved; robust chelicerae, clypeus extends forward on a curve; LBl 1.99, LBw 2.10; sternum hirsute, clothed with shorter black/faded black setae. *Abdomen*: Densely clothed dorsally in short black setae with numerous longer, lighter setae interspersed (generally red or orange *in situ*); dense dorsal patch of black Type I urticating bristles (Cooke et al. 1972); ventral side with shorter black setae. *Spermathecae*: Paired and separate, tapering and slightly curving medially towards capitate bulbs, with wide bases that are not fused. *Legs*: Hirsute, particularly ventrally; densely clothed in short and medium black pubescence, with longer setae colored similarly as the long abdominal setae; F1 12.61; F1w 4.06; P1 6.22; T1 9.97; M1 8.17; A1 6.37; F3 10.52; F3w 3.79; P3 4.53; T3 7.70; M3 8.13; A3 6.72; F4 12.91; F4w 3.81; P4 5.19; T4 10.69; M4 11.47; A4 7.32. All tarsi fully scopulate. Extent of metatarsal scopulation: leg III (SC3) = 69.0%; leg IV (SC4) = 33.9%. One ventral spinose seta on metatarsus III; six ventral spinose setae on metatarsus IV. *Coxa I*: Prolateral surface a mix of fine, hair-like and tapered setae. *Pedipalps*: Densely clothed in the same setal color as the other legs; one spinose seta on the apical, prolateral femur, one spinose seta on the prolateral patella, and four spinose setae on the prolateral tibia.

**Variation (8).** Cl 11.23–16.38 (13.636±0.64), Cw 9.77–14.63 (11.819±0.59), LBl 1.48–1.99 (1.778±0.08), LBw 1.77–2.42 (2.06±0.09), F1 8.86–12.61 (10.74±0.52), F1w 2.86–4.06 (3.401±0.16), P1 4.17–6.22 (5.001±0.24), T1 7.09–9.97 (8.447±0.38), M1 5.08–8.17 (6.301±0.38), A1 4.46–6.37 (5.274±0.23), L1 length 29.66–43.34 (35.763±1.72), F3 7.54–10.52 (8.761±0.42), F3w 2.45–3.79 (2.978±0.15), P3 3.26–4.93 (3.99±0.22), T3 4.87–7.70 (6.312±0.38), M3 5.24–8.13 (6.611±0.39), A3 4.65–6.72 (5.367±0.25), L3 length 25.56–37.60 (31.041±1.6), F4 8.93–12.91 (10.773±0.56), F4w 2.67–3.81 (3.076±0.14), P4 3.39–5.35 (4.438±0.22), T4 7.54–10.69 (8.885±0.4), M4 7.87–11.47 (9.504±0.46), A4 5.44–7.32 (6.122±0.25), L4 length 33.76–47.58 (39.721±1.85), SC3 ratio 0.495–0.69 (0.604±0.02), SC4 ratio 0.269–0.369 (0.332±0.01), Coxa I setae = tapered. Spermathecae variation can be seen in Figure 145.

#### Material examined.

**United States: Arizona: Cochise**: 0.45 miles NNE Cazador Trl on Hwy 80, 31.4724344 -109.4502641 ^2^, 4554ft., [APH_0720-0721, 17/8/2009, 2♂, Alice Abela, AUMNH]; 1 mile west of Portal, 31.914744 -109.162506 ^5^, 5026ft., [APH_2130, 24/9/1963, 1♂, V. Roth, AMNH]; [APH_2135, 30/10/1963, 1♀, V. Roth, AMNH]; 10 miles south of Apache, 31.572141 -109.17493 ^5^, 4669ft., [APH_2132, 10/9/1965, 1♂, W.J. Gertsch, AMNH]; 10 miles west of Douglas, 31.363766 -109.696872 ^5^, 4150ft., [APH_2108, 8/9/1964, 1♂, R. Hastings and M. Hastings, AMNH]; 10-20 mile south of Apache, 31.494756 -109.166548 ^5^, 4459ft., [APH_2123, 11/10/1963, 1♀, V. Roth, AMNH]; 12 miles south of Apache, 31.55928 -109.183506 ^5^, 4590ft., [APH_2106, 8/10/1964, 3♂, V. Roth , AMNH]; 12-16 miles south of Apache, 31.42937 -109.152669 ^5^, 4383ft., [APH_2125, 23/7/1963, 3♂, V. Roth, AMNH]; 2 miles northeast of Portal, 31.938743 -109.172137 ^5^, 5938ft., [APH_2140, 10/2/1962, 1♀, Cazier and Mortenson, AMNH]; 2 miles south of Rodeo, 31.921206 -109.050959 ^5^, 4209ft., [APH_2141, 20/6/1962, 1♀, N.M. , AMNH]; 2 miles south of Southwestern Research Station, 31.862638 -109.228246 ^5^, 6499ft., [APH_2137, 20/7/1959, 1♀, G.M. Happ and F.A. McKittrick, AMNH]; Deer Creek Ranch, 32.252852 -109.832012 ^5^, 4173ft., [APH_2126, 19/7/1956, 1♀, J. Anderson, AMNH]; Galeyville, 31.943827 -109.213586 ^5^, 5469ft., [APH_2124, 28/8/unknown, 1♂, Scotty Anderson, AMNH]; Portal, 31.913703 -109.14145 ^5^, 4770ft., [APH_2110, 23/10/1972, 1♂, W.J. Gertsch, AMNH]; [APH_2112, 24/8/1964, 2♂, R. Hastings and M. Hastings, AMNH]; [APH_2116, 15/11/1962, 1♂, V. Roth, AMNH]; [APH_2118, 1965, 1♂, W.J. Gertsch, AMNH]; [APH_2129, 15/7/1963, 1♀, V. Roth, AMNH]; [APH_2136, 8/1965, 1♀, W.J. Gertsch, AMNH]; Sierra Vista, just outside city limits, near and around residential areas near Ft. Huachuca Military Base, 31.47973 -110.24666 ^1^, 4643ft., [APH_0187, 24/8/2007, 1♂, Dean Pittman, AUMNH]; [APH_0188, 3/8/2007, 1♀, Dean Pittman, AUMNH]; **Graham**: 0.25 miles E Hwy-191 on Tanque Rd, 32.605235 -109.682418 ^1^, 3873ft., [APH_1331-1335, 1/8/2011, 1♀, 4 juv, Brent E. Hendrixson, Brendon Barnes, Nate Davis, Jake Storms, AUMNH]; [APH_1346, 5/8/2011, 1♀, Brent E. Hendrixson, Brendon Barnes, Nate Davis, Jake Storms, AUMNH]; [APH_1348, 5/8/2011, 1 juv, Brent E. Hendrixson, Brendon Barnes, Nate Davis, Jake Storms, AUMNH]; [APH_1480, 1/8/2012, 1♀, Brent E. Hendrixson, Brendon Barnes, Austin Deskewies, AUMNH]; [APH_1490-1491, 4/9/2012, 1♀, 1 juv, Brent E. Hendrixson, AUMNH]; [APH_1506-1508, 7/9/2012, 1♀, 2♂, Brent E. Hendrixson, AUMNH]; 0.4 miles E Hwy-191 on Tanque Rd, 32.606204 -109.681524 ^1^, 3891ft., [APH_1185-1187, 25/7/2010, 1♀, 2 juv, Brent E. Hendrixson, Brendon Barnes, Nate Davis, AUMNH]; dirt road S of US-70, 32.744869 -109.344099 ^1^, 4088ft., [APH_0639-0640, 12/7/2009, 2♀, Brent E. Hendrixson, Nate Davis, AUMNH]; Klondyke Rd, SW of Hwy-70, 32.914146 -109.975734 ^1^, 3110ft., [APH_0700, 18/7/2009, 1 juv, Brent E. Hendrixson, Nate Davis, AUMNH]; Swift Trail, along Hwy-366, 32.67433 -109.78367 ^2^, 4885ft., [APH_1361-1364, 3/9/2011, 4♂, Brandon La Forest, AUMNH]; Tanque Rd, near Hwy-191, 32.604126 -109.681695 ^1^, 3887ft., [APH_0624-0625, 11/7/2009, 2 juv, Brent E. Hendrixson, Nate Davis, AUMNH]; **Pima**: 4405 W Speedway Blvd, Tucson, 32.236347 -111.059977 ^2^, 2600ft., [APH_1359, 4/9/2011, 1♂, Lee and Denise Roberts, AUMNH]; 6928 N. Northpoint Dr, Tucson, 32.33242 -111.03403 ^2^, 2320ft., [APH_0400, 7/9/2008, 1♂, Linda Lanan, AUMNH]; 9095 E. Arbab Ct., Tucson, 32.11585 -110.79889 ^2^, 2907ft., [APH_0177, 29/8/2007, 1♂, Sandi Sowers, AUMNH]; Arivaca Creek (AZ-289/Ruby Rd), 31.59577 -111.36337 ^4^, 3560ft., [APH_0294-0295, 3/10/2007, 2♂, Manny Rubio, AUMNH]; Catalina State Park, Romero Canyon Trail, 32.4253 -110.8994 ^2^, 2850ft., [APH_1488, 22/7/2011, 1♀, Ken Macneil, AUMNH]; jct. I-10 and Hwy 83, 32.004689 -110.689365 ^5^, 3448ft., [AUMS_2212, 19/8/1995, 1♂, Jeremy Huff, AUMNH]; Santa Catalina Mountains, Mt. Lemmon Highway, Gordon Hirabayashi Recreation Area, 32.339509 -110.717456 ^2^, 4845ft., [APH_3217, 1/7/14, 1 juv, Brent E. Hendrixson, AUMNH]; Mt. Lemmon Hwy, 0.3 miles S of Molino Basin, S of Catalina Mtns, 32.326961 -110.702536 ^4^, 4101ft., [AUMS_3278, 16/9/1974, 1♂, W. Icenogle, AUMNH]; SE of Tucson, 3.5 miles N I-10 on Houghton Rd, 32.1125 -110.77611 ^2^, 2950ft., [APH_0791, unknown, 1♀, Jon Camp, AUMNH]; SE of Tucson, just N of I-10 and Hwy 83 jct., 32.012461 -110.690131 ^5^, 3433ft., [AUMS_2205, 19/8/1995, 2♂, J. Huff and Thomas R. Prentice, AUMNH]; [AUMS_2213, 19/8/1995, 1♂, R. Portillo and Thomas R. Prentice, AUMNH]; [AUMS_2225, 19/8/1995, 1♂, Thomas Mason, AUMNH]; Tucson, 32.221743 -110.926479 ^6^, 2470ft., [APH_2318, 1♂, T. Vorhies, AMNH]; off Hwy 79, N of Tucson just past Hwy 77 split, E side of road, 32.57446 -110.94732 ^1^, 3445ft., [APH_3188, 14/11/2013, 1♀, Chris A. Hamilton, Brent E. Hendrixson, AUMNH]; **Santa Cruz**: 0.1 miles SE FS-184 on Mt. Hopkins Rd, 31.69358253 -110.9889079 ^2^, 3684ft., [APH_0732, 20/8/2009, 1♂, Alice Abela, AUMNH]; 0.13 miles NW FS-184 on Mt. Hopkins Rd, 31.69571757 -110.9927435 ^2^, 3630ft., [APH_0731, 20/8/2009, 1♂, Alice Abela, AUMNH]; 0.37 miles NE FR-4099 on Mt. Hopkins Rd, 31.67437255 -110.9370608 ^2^, 4406ft., [APH_0734, 20/8/2009, 1♂, Alice Abela, AUMNH]; 1.6 miles E I-19 Frontage Rd along Amado-Mentosa Rd (FS Rd 184), 31.70242578 -111.0435914 ^2^, 3156ft., [APH_0382, 28/7/2008, 1 juv, Alice Abela, AUMNH]; Patagonia Mtns, Harshaw Road, 31.528093 -110.711410 ^4^, 4215ft., [APH_0185, 9/9/2007, 1♂, Manny Rubio, AUMNH]; Patagonia Mtns, Harshaw Road, 31.531246 -110.718179 ^4^, 4231ft., [APH_0186, late/8/2007, 1♂, Manny Rubio, AUMNH]; **New Mexico: Dona Ana**: Aguirre Springs Rd, 32.399191 -106.548438 ^1^, 4905ft., [APH_0656-0658, 13/7/2009, 3♂, Brent E. Hendrixson, Nate Davis, AUMNH]; [APH_0660-0661, 13/7/2009, 2♂, Brent E. Hendrixson, Nate Davis, AUMNH]; Just outside Las Cruces, on Aguirre Spring Road, 32.431202 -106.54921 ^5^, 5306ft., [APH_0001, 8/2003, 1♂, Roy Thibodeau, AUMNH]; Organ Mtns, Aguirre Springs Rec Area, 32.387276 -106.551429 ^1^, 5061ft., [APH_1176, 22/7/2010, 1♂, Brent E. Hendrixson, Brendon Barnes, Nate Davis, AUMNH]; **Hidalgo**: 1 mile west of Animas, 31.948038 -108.828004 ^5^, 4403ft., [APH_2119, 26/8/1960, 1♂, unknown, AMNH]; 11 miles south of Road Forks, 32.208574 -108.798363 ^5^, 4341ft., [APH_2128, 26/8/1960, 1♂, unknown, AMNH]; 3 miles W of Animas on Hwy-9, 31.942609 -108.856771 ^1^, 4400ft., [APH_0292, 10/2007, 1♂, Kari McWest, AUMNH]; 3.1 miles E Hwy-80 on Hwy-9, 31.93239033 -108.9869393 ^2^, 4135ft., [APH_0725, 18/8/2009, 1♂, Alice Abela, AUMNH]; 4.1 miles E Hwy-80 on Hwy-9, 31.936704 -108.970448 ^1^, 4187ft., [APH_0679, 16/7/2009, 1 juv, Brent E. Hendrixson, Nate Davis, AUMNH]; along Hwy-338, 32.0242 -108.866316 ^1^, 4297ft., [APH_1512, 8/9/2012, 1♂, Brent E. Hendrixson, AUMNH]; [APH_1517, 8/9/2012, 1♂, Brent E. Hendrixson, AUMNH]; along Hwy-80 at Granite Gap, 32.088477 -108.973445 ^1^, 4450ft., [APH_1484, 1/8/2012, 1♀, Brent E. Hendrixson, Brendon Barnes, Austin Deskewies, AUMNH]; along Hwy-80, 2.3 miles S I-10, 32.204378 -108.949722 ^1^, 4227ft., [APH_0674-0675, 15/7/2009, 1♀, 1 juv, Brent E. Hendrixson, Nate Davis, AUMNH]; along Hwy-9, W of Animas, 31.94331229 -108.8738359 ^2^, 4386ft., [APH_0727, 18/8/2009, 1♂, Alice Abela, AUMNH]; Clanton Draw, Geronimo Gap - Peloncillo Mtns, 31.517505 -108.984343 ^4^, 5441ft., [APH_0886, 2006, 1♀, Dave Moellendorf, AUMNH]; **Luna**: Cookes Canyon Rd, A019, 0.7 miles NW NM-26, 32.424 -107.583806 ^1^, 4545ft., [APH_0394, 28/7/2008, 1 juv, Kari, Hunter McWest, AUMNH]; Little Florida Mtns, 1 miles SE on Bonita Rd from turnoff on Gap Rd, 32.15817 -107.58858 ^1^, 4480ft., [APH_0195-0196, 3/9/2007, 2♂, Lorenzo Prendini, Jeremy Huff, AUMNH].

#### Distribution and natural history.

*Aphonopelma vorhiesi* is widely distributed across southeastern Arizona and southern New Mexico (Fig. 146) and can be found in the following Level III Ecoregions: Madrean Archipelago, Sonoran Basin and Range, Chihuahuan Deserts, and Arizona/New Mexico Mountains. *Aphonopelma vorhiesi* can be found in syntopy with a large number of different species across its distribution including *Aphonopelma catalina*, *Aphonopelma chalcodes*, *Aphonopelma chiricahua*, *Aphonopelma gabeli*, *Aphonopelma hentzi*, *Aphonopelma madera*, *Aphonopelma paloma*, *Aphonopelma parvum*, *Aphonopelma peloncillo*, and *Aphonopelma saguaro*. The breeding season, when mature males abandon their burrows in search of females, occurs during the summer and fall (July–October).

#### Conservation status.

*Aphonopelma vorhiesi* is very common throughout its distribution in southeastern Arizona and southern New Mexico. The species is secure.

#### Remarks.

Mitochondrial DNA identifies *Aphonopelma vorhiesi* as a paraphyletic group with respect to *Aphonopelma chalcodes* (Fig. 7). The secondary lineages outside of the *Aphonopelma vorhiesi* clade were previously identified as putative cryptic species by Hamilton et al. (2014), due to deep mitochondrial divergence. Results from the AE analysis demonstrate that *CO1* is not effective at accurately delimiting species boundaries within this group. Other important ratios that distinguish males: *Aphonopelma vorhiesi* possess a smaller L4 scopulation extent (20%-36%) than *Aphonopelma chalcodes* (66%–76%) and *Aphonopelma gabeli* (36%–47%). Other important ratios that distinguish females: *Aphonopelma vorhiesi* possess a larger T1/M3 (≥1.22; 1.22–1.39) than *Aphonopelma gabeli* (≤1.20; 1.13–1.20) and smaller than *Aphonopelma chiricahua* (1.53 ± (only 1 specimen)). For both males and females, certain morphometrics have potential to be useful, though due to the amounts of variation, small number of specimens, and the small differences between species, no others are claimed to be significant at this time (see Suppl. material 2). During evaluation of PCA morphospace, males of *Aphonopelma vorhiesi* separate from their syntopic species *Aphonopelma catalina*, *Aphonopelma chalcodes*, *Aphonopelma chiricahua*, *Aphonopelma gabeli*, *Aphonopelma madera*, and members of the miniature tarantulas (*Aphonopelma paloma*, *Aphonopelma parvum*, and *Aphonopelma saguaro*), but do not separate from *Aphonopelma hentzi* or *Aphonopelma peloncillo*. Female *Aphonopelma vorhiesi* separate in two-dimensional morphological space from *Aphonopelma chalcodes*, *Aphonopelma chiricahua*, and members of the miniature tarantulas, but do not separate from *Aphonopelma catalina*, *Aphonopelma gabeli*, *Aphonopelma hentzi*, *Aphonopelma madera*, or *Aphonopelma peloncillo*. Interestingly, *Aphonopelma vorhiesi* males separate from *Aphonopelma catalina*, *Aphonopelma chalcodes*, *Aphonopelma chiricahua*, *Aphonopelma gabeli*, *Aphonopelma hentzi*, *Aphonopelma madera*, *Aphonopelma parvum*, *Aphonopelma paloma*, and *Aphonopelma saguaro* in three-dimensional PCA morphospace (PC1~PC2~PC3), but do not separate from *Aphonopelma peloncillo*. *Aphonopelma vorhiesi* females separate from *Aphonopelma chiricahua*, *Aphonopelma parvum*, *Aphonopelma paloma*, and *Aphonopelma saguaro*, but do not separate from *Aphonopelma catalina*, *Aphonopelma chalcodes*, *Aphonopelma gabeli*, *Aphonopelma hentzi*, *Aphonopelma madera*, and *Aphonopelma peloncillo*. PC1, PC2, and PC3 explain ≥98% of the variation in all analyses.

We examined the holotypes and freshly collected topotypic material of *Aphonopelma jungi* and *Aphonopelma punzoi*. Our morphological and molecular analyses fail to recognize these two species as separate, independently evolving lineages. As a consequence, we consider *Aphonopelma jungi* and *Aphonopelma punzoi* junior synonyms of *Aphonopelma vorhiesi*.

### Aphonopelma xwalxwal

Taxon classificationAnimaliaAraneaeTheraphosidae

Hamilton sp. n.

http://zoobank.org/EDADFC4B-1113-4FD2-9BB5-DCC7329D435C

**Figure 147.**
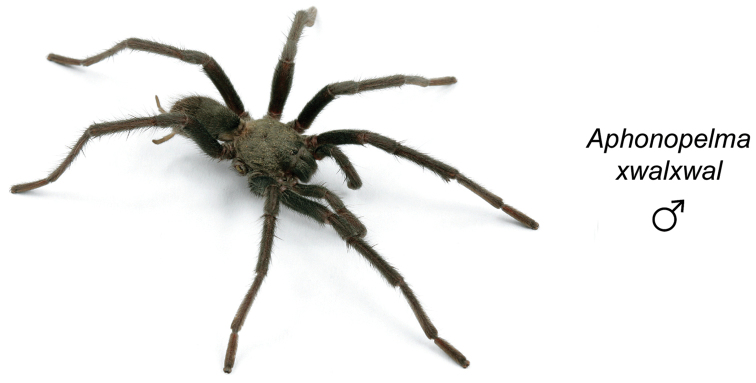
*Aphonopelma xwalxwal* sp. n. live photograph. Male paratype - APH_3133.

**Figure 148.**
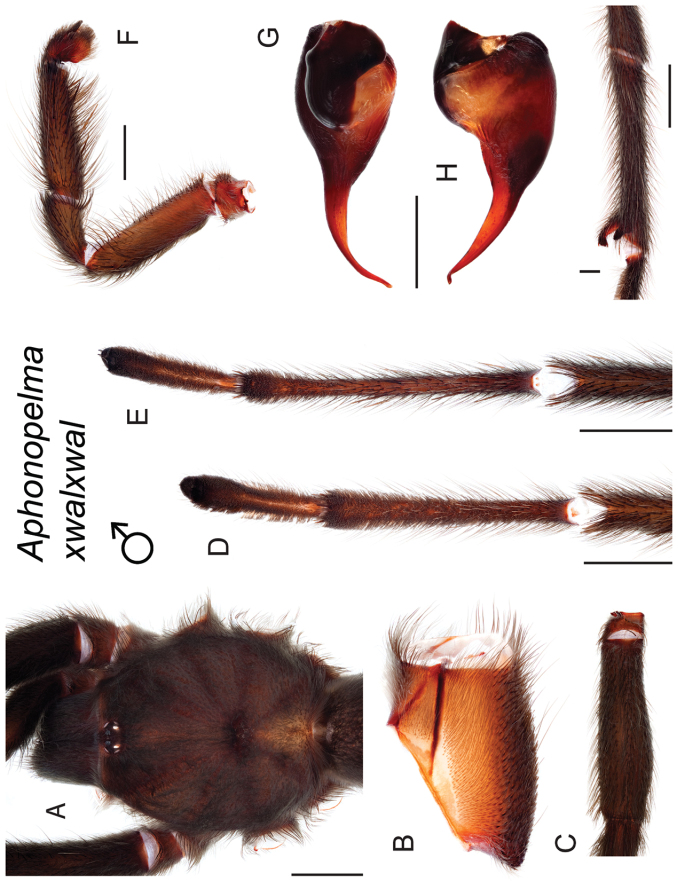
*Aphonopelma xwalxwal* sp. n. **A–I** male holotype, APH_3134 **A** dorsal view of carapace, scale bar = 3.5mm **B** prolateral view of coxa I **C** dorsal view of femur III **D** ventral view of metatarsus III, scale bar = 4mm **E** ventral view of metatarsus IV, scale bar = 4.5mm **F** prolateral view of L pedipalp and palpal tibia, scale bar = 2.5mm **G** dorsal view of palpal bulb **H** retrolateral view of palpal bulb, scale bar = 1mm **I** prolateral view of tibia I (mating clasper), scale bar = 4mm.

**Figure 149.**
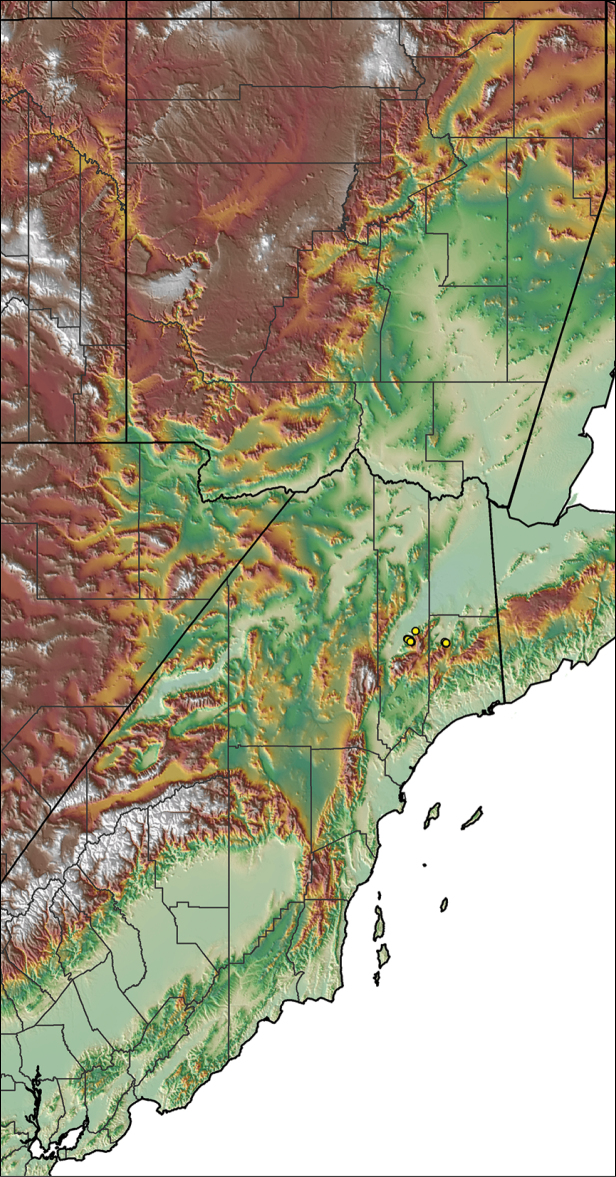
*Aphonopelma xwalxwal* sp. n. distribution of known specimens. There is no predicted distribution map due to the limited number of sampling localities and restricted distribution this species possesses.

Figures 147 , 148 , 149

#### Types.

Male holotype (APH_3134) collected on Hwy S22 (Montezuma Valley Rd), 3.3 miles S junction with Palm Canyon Dr. (Borrego Springs), Anza-Borrego State Park, San Diego Co., California, 33.225633 -116.414633 ^1^, elev. 1710ft., 4.x.2013, coll. Wendell Icenogle; deposited in AUMNH. Paratype male (APH_3133) from Hwy S22 (Montezuma Valley Rd), 4 miles S junction with Palm Canyon Dr. (Borrego Springs), Anza-Borrego State Park, San Diego Co., California, 33.224618 -116.424232 ^1^, elev. 1935ft., 5.x.2013, coll. Wendell Icenogle; deposited in AMNH.

#### Etymology.

The specific epithet is a noun in apposition, referencing the word for “a type of small spider” in the language of the Cahuilla Native Americans, whose traditional territory includes the distribution of this species (from the Coachella Valley to Borrego Springs). Pronounced “hwalhwal”; *xw* - sounds like a light raspy sound when blowing out a candle; *a* - sounds like “a” in father; *l* - sounds like “l” in light.

#### Diagnosis.

*Aphonopelma xwalxwal* (Fig. 147) is a member of the *paloma* species group and can be distinguished by a combination of morphological, molecular, and geographic characteristics. Nuclear and mitochondrial DNA identifies *Aphonopelma xwalxwal* as a phylogenetically distinct monophyletic lineage, supported as the sister lineage to the remaining members of the *paloma* species group (Figs 7–8). *Aphonopelma xwalxwal* is the largest of the miniature *Aphonopelma*, and while they are very similar looking to *Aphonopelma joshua*, *Aphonopelma xwalxwal* are slightly larger and has a different mating period (fall versus summer). *Aphonopelma xwalxwal* can be distinguished from *Aphonopelma eutylenum* by its smaller size, general phenotypic appearance, and the extent of scopulation on metatarsus IV, and from all other members of the *paloma* species group by locality. Like *Aphonopelma joshua*, *Aphonopelma xwalxwal* possess unique stout setae on the sternum. The significant measurement that distinguishes male *Aphonopelma xwalxwal* from its closely related phylogenetic and syntopic species is F4. Male *Aphonopelma xwalxwal* can be distinguished by possessing a smaller F4/T4 (≤1.05; 1.00–1.05) than *Aphonopelma eutylenum* (≥1.14; 1.14–1.22), *Aphonopelma icenoglei* sp. n. (≥1.14; 1.14–1.18), *Aphonopelma joshua* (≥1.07; 1.07–1.15), and *Aphonopelma paloma* (≥1.14; 1.14–1.24). Females of *Aphonopelma xwalxwal* are unknown at this time, though juvenile males look very similar to *Aphonopelma joshua*.

#### Description of male holotype

(APH_3134; Fig. 148). *Specimen preparation and condition*: Specimen collected live crossing road, preserved in 80% ethanol; original coloration faded due to preservation. Left legs I, III, IV, and left pedipalp removed for measurements and photographs; stored in vial with specimen. Right legs II & III removed for DNA and stored at -80°C in the AUMNH (Auburn, AL); missing leg IV right side. *General coloration*: Black. *Cephalothorax*: Carapace 10.49 mm long, 9.92 mm wide; densely clothed with black/faded black pubescence, slight iridescence, appressed to surface; fringe covered in long setae not closely appressed to surface; foveal groove medium deep and straight; pars cephalica region rises gradually from foveal groove on a straight plane towards the ocular area; AER procurved, PER slightly recurved; normal sized chelicerae; clypeus slightly extends forward on a curve; LBl 1.45, LBw 1.50; sternum hirsute, clothed with black, unique setae; setae are stout like *joshua*. *Abdomen*: Densely clothed in short black pubescence with numerous longer, lighter setae interspersed (generally red or orange *in situ*); dense dorsal patch of black Type I urticating bristles (Cooke et al. 1972) - smaller and distinct from large species; ventral setae same as dorsal. *Legs*: Hirsute; densely clothed with mostly short black setae, and numerous long, stout spines throughout. Metatarsus I straight. F1 11.85; F1w 2.67; P1 4.80; T1 11.72; M1 10.68; A1 6.36; F3 10.49; F3w 3.30; P3 3.74; T3 9.92; M3 11.21; A3 6.05; F4 12.65; F4w 2.81; P4 4.19; T4 12.01; M4 14.37; A4 6.80; femur III is swollen. All tarsi fully scopulate. Extent of metatarsal scopulation: leg III (SC3) = 94.7%; leg IV (SC4) = 34.8%. No distinct ventral spinose setae on metatarsus III, though numerous medium stout setae throughout; five ventral, one prolateral, and two retrolateral spinose setae - one near the margin with the tibia, on metatarsus IV; three prolateral spinose setae and one ventral on tibia I; two megaspines present on the retrolateral tibia, at the apex of the mating clasper; two megaspines on either side of the apex on the retrolateral branch of the tibial apophyses. *Coxa I*: Prolateral surface covered by fine, hair-like setae. *Pedipalps*: Hirsute; densely clothed in the same setal color as the other legs, with numerous longer ventral setae; one spinose seta at the apical, prolateral femur; one spinose seta on the prolateral patella; six prolateral spinose setae and two ventral on the palpal tibia, along with numerous medium stout setae throughout; PTl 6.382, PTw 1.744. When extended, embolus tapers with a curve to the retrolateral side; embolus slender, no keels; distinct dorsal and ventral transition from bulb to embolus.

**Variation (5).** Cl 8.389–10.491 (9.24±0.36), Cw 7.387–9.92 (8.664±0.45), LBl 1.142–1.454 (1.24±0.06), LBw 1.211–1.503 (1.361±0.06), F1 9.357–11.849 (10.398±0.43), F1w 2.233–2.672 (2.407±0.09), P1 3.656–4.797 (4.099±0.21), T1 8.907–11.723 (10.242±0.49), M1 7.681–10.68 (9.069±0.51), A1 4.786–6.363 (5.39±0.27), L1 length 34.436–45.412 (39.197±1.9), F3 7.71–10.492 (9.045±0.47), F3w 2.537–3.297 (2.854±0.13), P3 3.217–3.743 (3.398±0.1), T3 7.011–9.921 (8.28±0.49), M3 8.272–11.209 (9.72±0.5), A3 4.884–6.055 (5.419±0.22), L3 length 31.169–41.42 (35.861±1.74), F4 9.182–12.649 (10.644±0.59), F4w 2.179–2.81 (2.357±0.11), P4 3.471–4.192 (3.708±0.14), T4 9.03–12.006 (10.39±0.51), M4 11.074–14.367 (12.661±0.57), A4 5.047–6.798 (5.724±0.31), L4 length 38.048–50.012 (43.127±2.02), PTl 4.878–6.382 (5.53±0.25), PTw 1.309–1.744 (1.532±0.07), SC3 ratio 0.651–0.947 (0.773±0.07), SC4 ratio 0.348–0.485 (0.405±0.02), Coxa I setae = very thin tapered, F3 condition = swollen.

#### Material examined.

**United States: California: Riverside**: Carrizo Creek, .25 miles S of Hwy 74 bridge, 4 miles S of Palm Desert or Hwy 111, 33.643993 -116.400317 ^4^, 2572ft., [AUMS_3319, 27/3/1972, 1♂, W. Icenogle, AUMNH]; Deep Canyon, 33.669056 -116.367683 ^5^, 699ft., [AUMS_2668, 23/10/1963, 1♂, E. Schlinger, AUMNH]; on Hwy 74, 2.5 miles S of Carrizo Creek bridge, 6.5 miles S of Palm Desert or Hwy 111, 33.627668 -116.40353 ^4^, 2925ft., [AUMS_3318, 15/10/1976, 1♂, W. Icenogle, AUMNH]; P.L. Boyd Deep Canyon Reserve Center, 3.5 miles S of Palm Desert, 33.674866 -116.371261 ^4^, 639ft., [AUMS_2670, 13/10/1969, 1♂, Saul Frommer, AUMNH]; P.L. Boyd Deep Canyon Reserve, 0.4 miles N of reserve station, 33.653477 -116.37419 ^4^, 891ft., [AUMS_2348, 22/9/1995, 1♂, T.R. Prentice, AUMNH]; P.L. Boyd Deep Canyon Reserve, 0.5 miles below reserve gate, 33.677408 -116.370109 ^4^, 613ft., [AUMS_2345, 27/9/1995, 1♂, T.R. Prentice, AUMNH]; P.L. Boyd Deep Canyon Reserve, 1.1 miles below reserve gate, 33.684368 -116.364817 ^4^, 558ft., [AUMS_2352, 21/9/1995, 1♂, T.R. Prentice, AUMNH]; P.L. Boyd Deep Canyon Reserve, at reserve gate - North, 33.670726 -116.372949 ^4^, 684ft., [AUMS_2344, 21/9/1995, 1♂, T.R. Prentice, AUMNH]; P.L. Boyd-Deep Canyon Reserve, 0.2 miles below reserve gate, 33.586027 -116.276451 ^4^, 613ft., [AUMS_3280, 27/9/1995, 1♂, T.R. Prentice, AUMNH]; **San Diego**: Hwy S22 (Montezuma Valley Rd), 1.6 miles S junction with Palm Canyon Dr. (Borrego Springs), Anza-Borrego State Park, 33.2409 -116.403283 ^1^, 1148ft., [APH_3132, 5/10/2013, 1♂, W. Icenogle, AUMNH]; Hwy S22 (Montezuma Valley Rd), 2.8 miles S junction with Palm Canyon Dr. (Borrego Springs), Anza-Borrego State Park, 33.231167 -116.411606 ^1^, 1474ft., [APH_3130, 4/10/2013, 1♂, W. Icenogle, AUMNH]; Hwy S22 (Montezuma Valley Rd), 3.2 miles S junction with Palm Canyon Dr. (Borrego Springs), Anza-Borrego State Park, 33.225583 -116.413067 ^1^, 1673ft., [APH_3131, 4/10/2013, 1♂, W. Icenogle, AUMNH]; Hwy S22 (Montezuma Valley Rd), 3.3 miles S junction with Palm Canyon Dr. (Borrego Springs), Anza-Borrego State Park, 33.225633 -116.414633 ^1^, 1710ft., [APH_3134, 4/10/2013, 1♂, W. Icenogle, AUMNH]; Hwy S22 (Montezuma Valley Rd), 4 miles S junction with Palm Canyon Dr. (Borrego Springs), Anza-Borrego State Park, 33.224618 -116.424232 ^1^, 1935ft., [APH_3133, 5/10/2013, 1♂, W. Icenogle, AMNH].

#### Distribution and natural history.

*Aphonopelma xwalxwal* is presently known from only two areas along the foothills and mountains west of Palm Springs and Borrego Springs (Fig. 149). The species can be found inhabiting the Sonoran Basin and Range Level III Ecoregion, in particular this species appears to be restricted to the Western Sonoran Mountains and Western Sonoran Mountain Woodland and Shrubland. *Aphonopelma xwalxwal* can be found in syntopy with *Aphonopelma eutylenum*. The breeding season, when mature males abandon their burrows in search of females, occurs during the fall (October).

#### Conservation status.

The distribution of *Aphonopelma xwalxwal* appears to be restricted to the mountains and foothills on the eastern side of the Peninsular Ranges and San Jacinto Mountains. In historical collections, this species appears to have been collected somewhat infrequently. Based on our own fieldwork in search of females of this species, we found burrows to be incredibly difficult to find due to the steep, rocky nature of their habitat, and the harsh, dry environments that likely keeps them inactive for most of the year. The species is likely secure, but populations should be monitored due to the continued expansion of human activity and development in Southern California.

#### Remarks.

Other important ratios that distinguish males: *Aphonopelma xwalxwal* possess a larger T3/A3 (≥1.41; 1.41–1.64) than *Aphonopelma eutylenum* (≤1.35; 1.30–1.35), *Aphonopelma icenoglei* (≤1.37; 1.24–1.37), *Aphonopelma joshua* (≤1.37; 1.19–1.37), and *Aphonopelma paloma* (≤1.31; 1.21–1.31); by possessing a larger L4 scopulation extent (34%–48%) than *Aphonopelma paloma* (5%–24%) and smaller than *Aphonopelma eutylenum* (62%–77%). Certain morphometrics have potential to be useful, though due to the amounts of variation, small number of specimens, and the small differences between species, no other are claimed to be significant at this time (see Suppl. material 2). During evaluation of PCA morphospace, males of *Aphonopelma xwalxwal* separate from *Aphonopelma eutylenum* and all other miniature species along PC1~2, except for *Aphonopelma joshua*. Interestingly, *Aphonopelma xwalxwal* males also separate from *Aphonopelma eutylenum* and all other miniature species, except for *Aphonopelma joshua*, in three-dimensional PCA morphospace (PC1~PC2~PC3). PC1, PC2, and PC3 explain ≥97% of the variation in all analyses.

## Supplementary Material

XML Treatment for Aphonopelma

XML Treatment for Aphonopelma anax

XML Treatment for Aphonopelma armada

XML Treatment for Aphonopelma atomicum

XML Treatment for Aphonopelma catalina

XML Treatment for Aphonopelma chalcodes

XML Treatment for Aphonopelma chiricahua

XML Treatment for Aphonopelma eutylenum

XML Treatment for Aphonopelma gabeli

XML Treatment for Aphonopelma hentzi

XML Treatment for Aphonopelma icenoglei

XML Treatment for Aphonopelma iodius

XML Treatment for Aphonopelma johnnycashi

XML Treatment for Aphonopelma joshua

XML Treatment for Aphonopelma madera

XML Treatment for Aphonopelma mareki

XML Treatment for Aphonopelma marxi

XML Treatment for Aphonopelma moderatum

XML Treatment for Aphonopelma moellendorfi

XML Treatment for Aphonopelma mojave

XML Treatment for Aphonopelma paloma

XML Treatment for Aphonopelma parvum

XML Treatment for Aphonopelma peloncillo

XML Treatment for Aphonopelma phasmus

XML Treatment for Aphonopelma prenticei

XML Treatment for Aphonopelma saguaro

XML Treatment for Aphonopelma steindachneri

XML Treatment for Aphonopelma superstitionense

XML Treatment for Aphonopelma vorhiesi

XML Treatment for Aphonopelma xwalxwal

## Supplementary materials

Supplementary material 1 Compressed file (.zip) containing two separate locality datasets of *Aphonopelma* specimens. Data type: Microsoft Excel spreadsheet (.xls) and Microsoft Excel comma delimited (.csv). Explanation note: Locality data for all *Aphonopelma* specimens examined over the course of this study and listed in the material examined section that accompanies each species, as well as locality data with Darwin Core headers. This dataset is made available under the Open Database License (http://opendatacommons.org/licenses/odbl/1.0/). The Open Database License (ODbL) is a license agreement intended to allow users to freely share, modify, and use this Dataset while maintaining this same freedom for others, provided that the original source and author(s) are credited.Chris A. Hamilton, Brent E. Hendrixson, Jason E. Bond

Supplementary material 2 Compressed files (.zip) containing *Aphonopelma* boxplot and PCA morphospace data. Data type: Microsoft Excel spreadsheet (.xls), Microsoft Excel comma delimited (.csv), Portable Document Format (.pdf), Simple Text (.txt), and Graphics Interchange Format (.gif). Explanation note: All boxplots and PCA morphospace files used in *Aphonopelma* species boundary determination. This dataset is made available under the Open Database License (http://opendatacommons.org/licenses/odbl/1.0/). The Open Database License (ODbL) is a license agreement intended to allow users to freely share, modify, and use this Dataset while maintaining this same freedom for others, provided that the original source and author(s) are credited.Chris A. Hamilton, Brent E. Hendrixson, Jason E. Bond

Supplementary material 3 Compressed file (.zip) containing simple R scripts for analyzing *Aphonopelma* morphological space. Data type: R scripts (.R). Explanation note: R scripts to run boxplot, 2D and 3D PCA morphospace analyses of *Aphonopelma* morphological space. These scripts will allow the user to include their own specimen morphological data to compare with known *Aphonopelma* species morphological space. We are hoping to provide a mechanism for species identification based on specimen data from natural history collections or specimens where molecular data is not possible or practical in gathering. Directions for using the scripts are included within the scripts. This dataset is made available under the Open Database License (http://opendatacommons.org/licenses/odbl/1.0/). The Open Database License (ODbL) is a license agreement intended to allow users to freely share, modify, and use this Dataset while maintaining this same freedom for others, provided that the original source and author(s) are credited.Chris A. Hamilton, Brent E. Hendrixson, Jason E. Bond

Supplementary material 4 Compressed files (.zip) containing supplemental morphology figures. Data type: Microsoft Word (.docx), Portable Document Format (.pdf), and Simple Text (.txt). Explanation note: Figures (like those provided in the species descriptions) of additional specimens (for species where additional photographs were available), to provide visual reference of the variation possible within *Aphonopelma* species. This dataset is made available under the Open Database License (http://opendatacommons.org/licenses/odbl/1.0/). The Open Database License (ODbL) is a license agreement intended to allow users to freely share, modify, and use this Dataset while maintaining this same freedom for others, provided that the original source and author(s) are credited.Chris A. Hamilton, Brent E. Hendrixson, Jason E. Bond
