# Supplementary material for: Taxonomic revision of the tarantula genus Aphonopelma Pocock, 1901 (Araneae, Mygalomorphae, Theraphosidae) within the United States
Source: Zookeys. 2016 Feb 4;(560):1–340. doi: 10.3897/zookeys.560.6264 (PMC4768370; doi:10.3897/zookeys.560.6264)
Supplement: Supplementary material 4 — Compressed files (.zip) containing supplemental morphology figures. [file zookeys-560-001-s004.zip › Supp4_0.zip!/Appendix 4/Appendix 4.docx]

**Appendix 4.**

Supplemental Morphology Figures.

1. *Aphonopelma anax* (Chamberlin, 1940), (A-I) male specimen, APH_0055. A - dorsal view of carapace, scale bar = 5mm; B - prolateral view of coxa I; C - dorsal view of femur III; D - ventral view of metatarsus III, scale bar - 3.5mm; E - ventral view of metatarsus IV, scale bar = 4.5mm; F - prolateral view of L pedipalp and palpal tibia, scale bar = 4mm; G - dorsal view of palpal bulb; H - retrolateral view of palpal bulb, scale bar = 1mm; I - prolateral view of tibia I (mating clasper), scale bar = 5mm.

2. *Aphonopelma anax* (Chamberlin, 1940), (A-E) female specimen, APH_0871. A - dorsal view of carapace, scale bar = 6mm; B - prolateral view of coxa I; C - ventral view of metatarsus III, scale bar - 3.5mm; D - ventral view of metatarsus IV, scale bar = 3.5mm; E - prolateral view of L pedipalp and palpal tibia.

3. *Aphonopelma chalcodes* Chamberlin, 1940, (A-I) male specimen, APH_0013. A - dorsal view of carapace, scale bar = 6mm; B - prolateral view of coxa I; C - dorsal view of femur III; D - ventral view of metatarsus III, scale bar - 4.5mm; E - ventral view of metatarsus IV, scale bar = 5mm; F - prolateral view of L pedipalp and palpal tibia, scale bar = 5mm; G - dorsal view of palpal bulb; H - retrolateral view of palpal bulb, scale bar = 1mm; I - prolateral view of tibia I (mating clasper), scale bar = 4.5mm.

4. *Aphonopelma chalcodes* Chamberlin, 1940, (A-E) female specimen, AUMS_2692. A - dorsal view of carapace, scale bar = 5mm; B - prolateral view of coxa I; C - ventral view of metatarsus III, scale bar - 4mm; D - ventral view of metatarsus IV, scale bar = 5mm; E - prolateral view of L pedipalp and palpal tibia.

5. *Aphonopelma hentzi* (Girard, 1854), (A-I) male specimen, APH_0936. A - dorsal view of carapace, scale bar = 6.5mm; B - prolateral view of coxa I; C - dorsal view of femur III; D - ventral view of metatarsus III, scale bar - 3.5mm; E - ventral view of metatarsus IV, scale bar = 4mm; F - prolateral view of L pedipalp and palpal tibia, scale bar = 3mm; G - dorsal view of palpal bulb; H - retrolateral view of palpal bulb, scale bar = 1mm; I - prolateral view of tibia I (mating clasper), scale bar = 3mm.

6. *Aphonopelma hentzi* (Girard, 1854), (A-E) female specimen, APH_0867. A - dorsal view of carapace, scale bar = 6.5mm; B - prolateral view of coxa I; C - ventral view of metatarsus III, scale bar - 4.5mm; D - ventral view of metatarsus IV, scale bar = 4.5mm; E - prolateral view of L pedipalp and palpal tibia.

7. *Aphonopelma iodius* (Chamberlin & Ivie, 1939), (A-I) male specimen, APH_1093. A - dorsal view of carapace, scale bar = 6.5mm; B - prolateral view of coxa I; C - dorsal view of femur III; D - ventral view of metatarsus III, scale bar - 4mm; E - ventral view of metatarsus IV, scale bar = 6mm; F - prolateral view of L pedipalp and palpal tibia, scale bar = 5mm; G - dorsal view of palpal bulb; H - retrolateral view of palpal bulb, scale bar = 1mm; I - prolateral view of tibia I (mating clasper), scale bar = 4.5mm.

8. *Aphonopelma iodius* (Chamberlin & Ivie, 1939), (A-I) male specimen, APH_1084. A - dorsal view of carapace, scale bar = 6.5mm; B - prolateral view of coxa I; C - dorsal view of femur III; D - ventral view of metatarsus III, scale bar - 5mm; E - ventral view of metatarsus IV, scale bar = 5mm; F - prolateral view of L pedipalp and palpal tibia, scale bar = 5mm; G - dorsal view of palpal bulb; H - retrolateral view of palpal bulb, scale bar = 1mm; I - prolateral view of tibia I (mating clasper), scale bar = 3.5mm.

9. *Aphonopelma iodius* (Chamberlin & Ivie, 1939), (A-I) male specimen, AUMS_2349. A - dorsal view of carapace, scale bar = 6mm; B - prolateral view of coxa I; C - dorsal view of femur III; D - ventral view of metatarsus III, scale bar - 4.5mm; E - ventral view of metatarsus IV, scale bar = 4.5mm; F - prolateral view of L pedipalp and palpal tibia, scale bar = 5mm; G - dorsal view of palpal bulb; H - retrolateral view of palpal bulb, scale bar = 1mm; I - prolateral view of tibia I (mating clasper), scale bar = 3mm.

10. *Aphonopelma iodius* (Chamberlin & Ivie, 1939), (A-E) female specimen, APH_2030. A - dorsal view of carapace, scale bar = 6mm; B - prolateral view of coxa I; C - ventral view of metatarsus III, scale bar - 3.5mm; D - ventral view of metatarsus IV, scale bar = 2.5mm; E - prolateral view of L pedipalp and palpal tibia.

11. *Aphonopelma iodius* (Chamberlin & Ivie, 1939), (A-E) female specimen, APH_0996. A - dorsal view of carapace, scale bar = 7mm; B - prolateral view of coxa I; C - ventral view of metatarsus III, scale bar - 4.5mm; D - ventral view of metatarsus IV, scale bar = 4mm; E - prolateral view of L pedipalp and palpal tibia.

12. *Aphonopelma mareki* **sp. nov.** (A-E) female specimen, APH_0297. A - dorsal view of carapace, scale bar = 2.5mm; B - prolateral view of coxa I; C - ventral view of metatarsus III, scale bar - 2mm; D - ventral view of metatarsus IV, scale bar = 2mm; E - prolateral view of L pedipalp and palpal tibia.

13. *Aphonopelma moderatum* (Chamberlin & Ivie, 1939), (A-I) male specimen, APH_0893. A - dorsal view of carapace, scale bar = 5mm; B - prolateral view of coxa I; C - dorsal view of femur III; D - ventral view of metatarsus III, scale bar - 4.5mm; E - ventral view of metatarsus IV, scale bar = 4mm; F - prolateral view of L pedipalp and palpal tibia, scale bar = 4mm; G - dorsal view of palpal bulb; H - retrolateral view of palpal bulb, scale bar = 1mm; I - prolateral view of tibia I (mating clasper), scale bar = 4mm.

14. *Aphonopelma moderatum* (Chamberlin & Ivie, 1939), (A-E) female specimen, APH_0894. A - dorsal view of carapace, scale bar = 5.5mm; B - prolateral view of coxa I; C - ventral view of metatarsus III, scale bar - 2mm; D - ventral view of metatarsus IV, scale bar = 3.5mm; E - prolateral view of L pedipalp and palpal tibia.

15. *Aphonopelma peloncillo* **sp. nov.** (A-I) male specimen, APH_0724. A - dorsal view of carapace, scale bar = 5mm; B - prolateral view of coxa I; C - dorsal view of femur III; D - ventral view of metatarsus III, scale bar - 4.5mm; E - ventral view of metatarsus IV, scale bar = 4mm; F - prolateral view of L pedipalp and palpal tibia, scale bar = 4.5mm; G - dorsal view of palpal bulb; H - retrolateral view of palpal bulb, scale bar = 1mm; I - prolateral view of tibia I (mating clasper), scale bar = 5.5mm.

16. *Aphonopelma saguaro* **sp. nov.** (A-I) male specimen, APH_2540. A - dorsal view of carapace, scale bar = 2mm; B - prolateral view of coxa I; C - dorsal view of femur III; D - ventral view of metatarsus III, scale bar - 1.5mm; E - ventral view of metatarsus IV, scale bar = 2mm; F - prolateral view of L pedipalp and palpal tibia, scale bar = 1.5mm; G - dorsal view of palpal bulb; H - retrolateral view of palpal bulb, scale bar = 0.5mm; I - prolateral view of tibia I (mating clasper), scale bar = 1.5mm.

17. *Aphonopelma vorhiesi* (Chamberlin & Ivie, 1939), (A-I) male specimen, APH_0186. A - dorsal view of carapace, scale bar = 3.5mm; B - prolateral view of coxa I; C - dorsal view of femur III; D - ventral view of metatarsus III, scale bar - 3mm; E - ventral view of metatarsus IV, scale bar = 3.5mm; F - prolateral view of L pedipalp and palpal tibia, scale bar = 4mm; G - dorsal view of palpal bulb; H - retrolateral view of palpal bulb, scale bar = 1mm; I - prolateral view of tibia I (mating clasper), scale bar = 4.5mm.

[NOMEN DUBIUM] *digitized for posterity

18. *Aphonopelma radinum* (Chamberlin & Ivie, 1939), (A-I) male holotype. A - dorsal view of carapace, scale bar = 2mm; B - prolateral view of coxa I; C - dorsal view of femur III; D - ventral view of metatarsus III; E - ventral view of metatarsus IV, scale bar = 2.5mm; F - prolateral view of L pedipalp and palpal tibia, scale bar = 2mm; G - dorsal view of palpal bulb; H - retrolateral view of palpal bulb, scale bar = 0.5mm; I - prolateral view of tibia I (mating clasper), scale bar = 3mm.
