# Supplementary material for: Taxonomic revision of the tarantula genus Aphonopelma Pocock, 1901 (Araneae, Mygalomorphae, Theraphosidae) within the United States
Source: Zookeys. 2016 Feb 4;(560):1–340. doi: 10.3897/zookeys.560.6264 (PMC4768370; doi:10.3897/zookeys.560.6264)
Supplement: Supplementary material 4 — Compressed files (.zip) containing supplemental morphology figures. [file zookeys-560-001-s004.zip › Supp4_2.zip!/Appendix 4-2 chalcodes/Appendix_4_3.pdf]

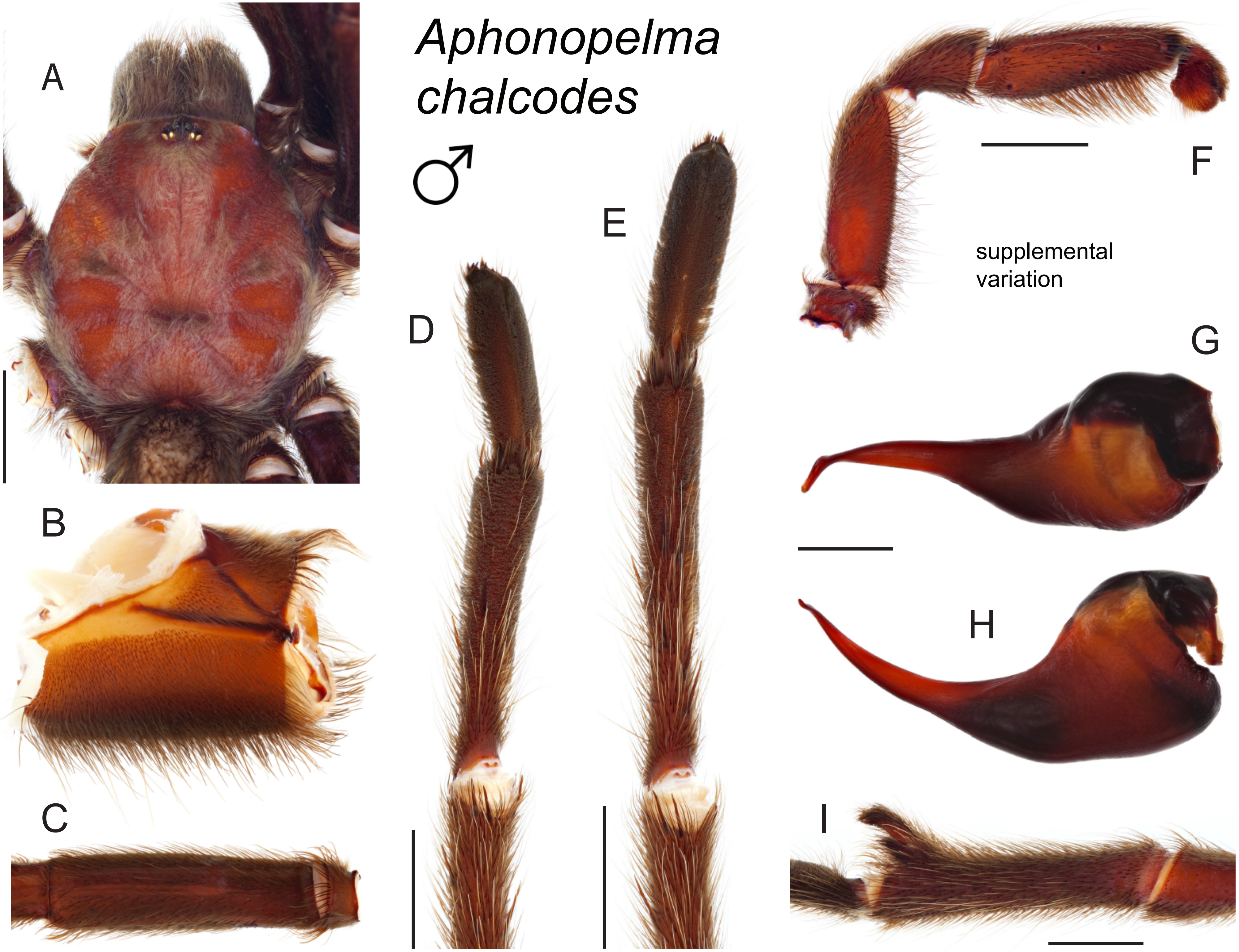

A
B
C
D
E
F
G
H
I
Aphonopelma chalcodes
♂
supplemental variation
