# Supplementary material for: Taxonomic revision of the tarantula genus Aphonopelma Pocock, 1901 (Araneae, Mygalomorphae, Theraphosidae) within the United States
Source: Zookeys. 2016 Feb 4;(560):1–340. doi: 10.3897/zookeys.560.6264 (PMC4768370; doi:10.3897/zookeys.560.6264)
Supplement: Supplementary material 4 — Compressed files (.zip) containing supplemental morphology figures. [file zookeys-560-001-s004.zip › Supp4_6.zip!/Appendix 4-6 moderatum/Appendix_4_13.pdf]

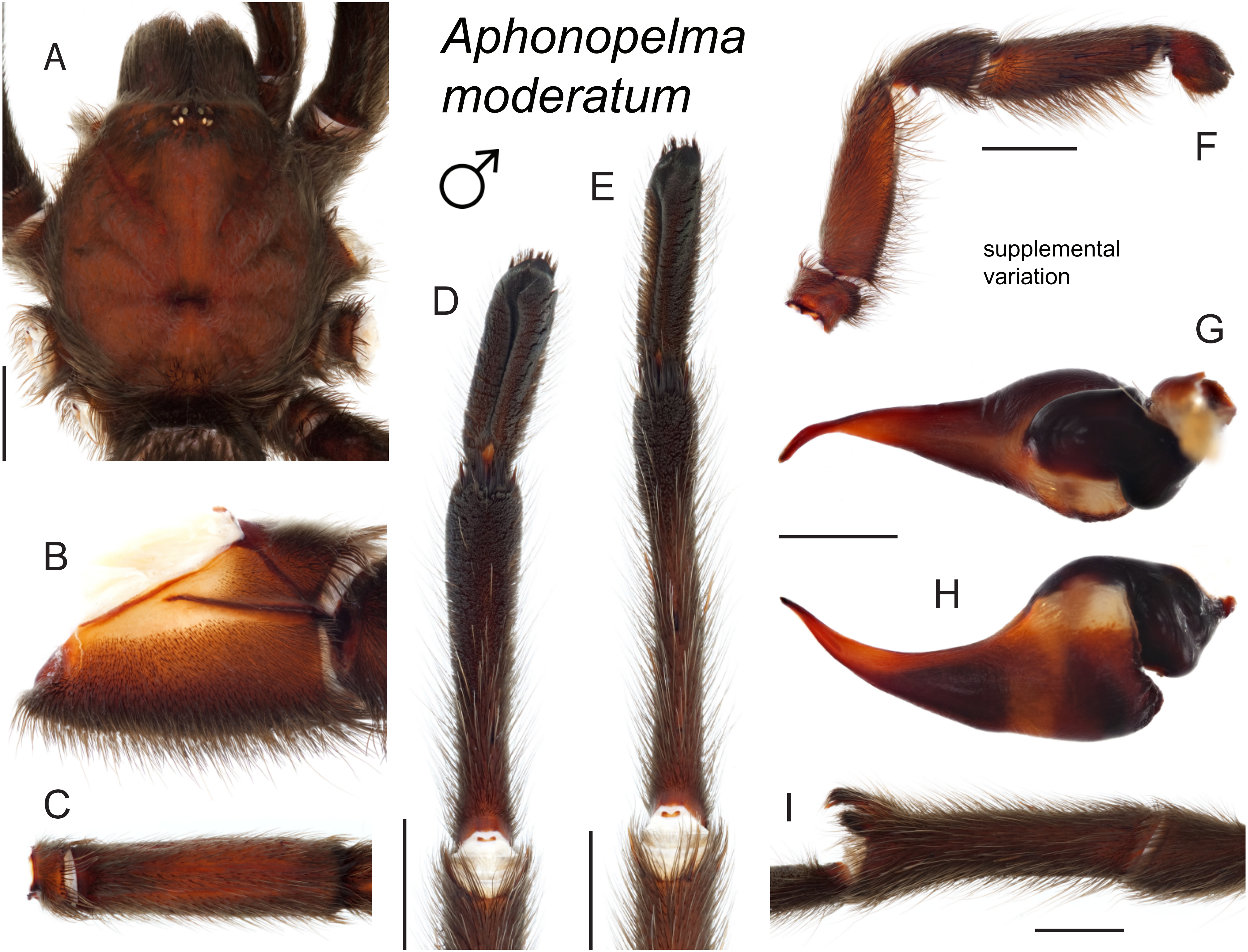

Aphonopelma moderatum
♂
A
B
C
D
E
F
G
H
I
supplemental variation
